# Computational Thermodynamic and Kinetic Analysis of Meisenheimer Intermediate Formation in Aromatic Nucleophilic Addition

**DOI:** 10.3390/molecules31173114

**Published:** 2026-09-05

**Authors:** Cheng Wang, Rui Liu, Shuo-Qing Zhang, Xin Hong

**Affiliations:** 1 Center of Chemistry for Frontier Technologies, Department of Chemistry, Zhejiang University, Hangzhou 310027, China; wangcheng20@zju.edu.cn (C.W.); lrliurui@zju.edu.cn (R.L.); 2 School of Chemistry and Chemical Engineering, Henan Normal University, Xinxiang 453007, China

**Keywords:** Meisenheimer intermediate, aromatic nucleophilic addition, dearomative functionalization, density functional theory, substrate–nucleophile compatibility, thermodynamic and kinetic analysis

## Abstract

Meisenheimer intermediates provide entry points to nucleophilic aromatic substitution and dearomative functionalization, but productive substrate–nucleophile combinations remain difficult to identify beyond strongly activated aromatic systems. We used density functional theory to map Meisenheimer intermediate formation for 95 substituted benzene and pyridine substrates with four carbon nucleophiles, corresponding to 380 combinations. The calculated reaction free energies spanned −90.1 to +33.9 kcal/mol. Electrophilicity and nucleophilicity captured broad thermodynamic trends, although neither descriptor predicted individual reaction energies with sufficient accuracy. A reference-centered thermodynamic window selected 84 combinations for transition-state searches; 44 addition transition states were located, with calculated addition barriers of 0.4–11.4 kcal/mol. Reaction free energy did not rank these barriers, showing that explicit kinetic analysis changed the priorities obtained from thermodynamic screening alone. Sequential application of the thermodynamic window and a barrier no higher than that of the experimental reference (8.0 kcal/mol) identified 32 combinations for experimental evaluation. The results define how aromatic scaffold, substitution topology, and nucleophile strength jointly control Meisenheimer intermediate accessibility and provide a focused set of substrate–nucleophile pairs for experimental testing.

## 1. Introduction

**Figure 1:**
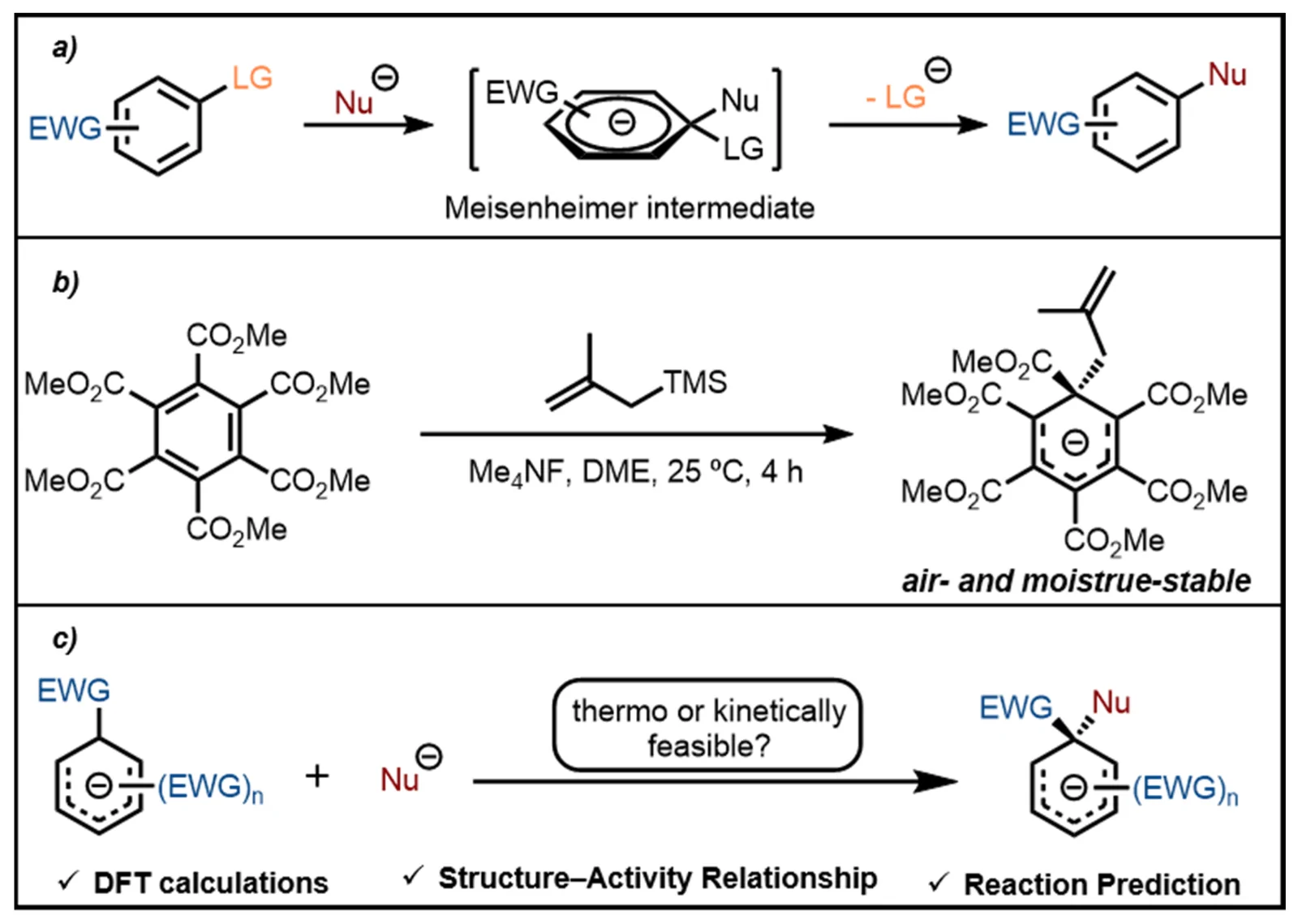
Meisenheimer intermediate formation in aromatic nucleophilic addition and its thermodynamic–kinetic evaluation. (**a**) Classical S_N_Ar addition–elimination pathway involving a Meisenheimer intermediate. (**b**) Bench-stable ester-stabilized Meisenheimer complex as an experimental precedent for persistent dearomatized σ-complexes [31]. (**c**) DFT evaluation of reaction free energies and nucleophilic-addition transition-state barriers for assessing substrate–nucleophile compatibility. EWG denotes electron-withdrawing group.

Aromatic nucleophilic addition is an elementary step in nucleophilic aromatic substitution (S_N_Ar) and dearomative functionalization. In a classical S_N_Ar reaction, nucleophilic attack on an electron-deficient aromatic π system gives the anionic σ-complex known as a Meisenheimer intermediate; leaving-group elimination then restores aromaticity (Figure 1a) [1,2,3,4,5,6,7,8,9]. When rearomatization is suppressed or delayed, the intermediate can instead undergo further bond formation. Its accumulation depends on both the free energy of σ-complex formation and the barrier to nucleophilic addition. These quantities describe different stages of the same reaction sequence and need not vary in parallel.

Traditional Meisenheimer chemistry uses strongly electron-withdrawing substituents, most often nitro groups, to stabilize anionic σ-complexes. Extending this chemistry to less activated aromatic substrates would broaden the range of dearomative transformations that convert planar aromatic feedstocks into non-planar, saturated frameworks [10,11,12,13,14,15,16,17,18,19,20,21,22,23,24,25,26]. Li and co-workers recently isolated bench-stable, ester-stabilized Meisenheimer complexes (Figure 1b) [27]. Their results show that substituents weaker than nitro groups can sustain persistent dearomatized intermediates when the substitution pattern and nucleophile are matched. The isolated complexes participated in 1,4-addition, [2+3] cycloaddition, and [4+n] cycloaddition reactions [27,28,29,30,31,32,33]. This precedent provides an experimental anchor for comparing 95 aromatic substrates with four carbon nucleophiles.

Recent experimental studies have extended nucleophilic dearomatization to quinolines and substituted naphthols, while cascade nucleophilic addition of 1,2,3-triazines provides access to substituted pyridines [34,35,36]. Related computational analysis of N-heteroarene electrocarboxylation further showed that the site and reversibility of nucleophilic C–C bond formation can depend on ring electron distribution and subsequent reaction steps, although the reported intermediates are reduced radical species rather than the closed-shell σ-complexes considered here [37].

For multisubstituted aromatic substrates, rules derived from a single substituent do not capture the combined effects of scaffold, position, and nucleophile identity. Previous calculations have related σ-complex stability to reactivity and site selectivity in nucleophilic aromatic substitution [38,39], and descriptor-based models have been developed for relative reactivity and activation-energy prediction in S_N_Ar chemistry [40,41,42]. DFT studies have also examined reaction pathways and selectivity in related catalytic dearomatizations [43,44]. Here, reaction free energies were calculated for the complete substrate–nucleophile set, followed by transition-state searches within a thermodynamic window defined around an experimental reference. Electrophilicity and nucleophilicity indices describe broad electronic trends; explicit addition barriers distinguish the kinetic behavior of combinations within that window.

## 2. Results and Discussion

### 2.1. Thermodynamic Trends in Meisenheimer Intermediate Formation

**Figure 2:**
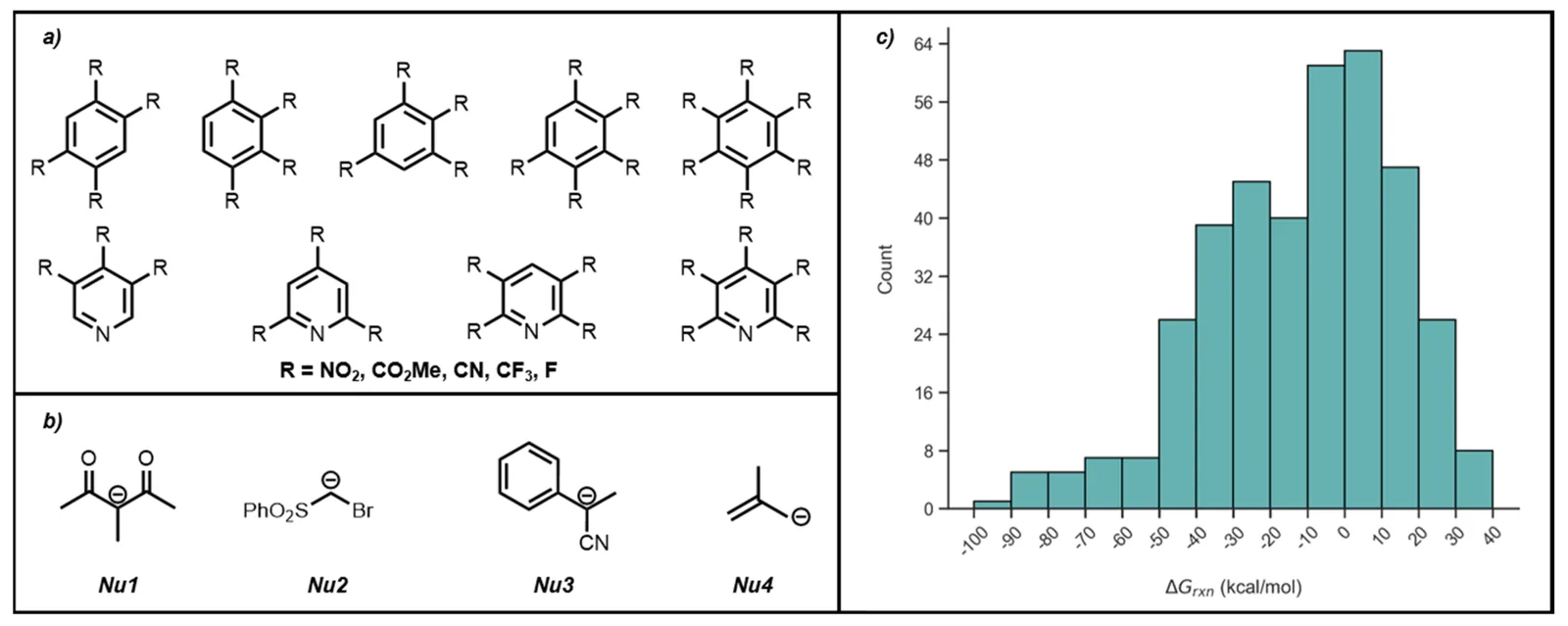
Chemical space and distribution of DFT-computed reaction free energies for Meisenheimer intermediate formation. (**a**) Substituted benzene and pyridine scaffolds considered in this study; EWG denotes electron-withdrawing group. (**b**) Four carbon nucleophiles used for aromatic nucleophilic addition. (**c**) Distribution of Δ*G*_rxn_ for 380 substrate–nucleophile combinations.

To define the reaction space for evaluating Meisenheimer intermediate formation, we constructed substituted benzene and pyridine scaffolds with systematically varied electronic environments. The set contains tetra-, penta-, and hexa-substituted benzenes and tri-, tetra-, and penta-substituted pyridines (Figure 2a). Five electron-withdrawing groups (EWGs), NO_2_, CO_2_Me, CN, CF_3_, and F, were chosen to represent different inductive and resonance contributions. Several are not conventional leaving groups because the present analysis concerns nucleophilic addition and σ-complex stabilization rather than only the elimination step of classical S_N_Ar. The isolation and subsequent reactions of ester-stabilized Meisenheimer complexes provide an experimental basis for including this broader substituent set [31]. The four carbon nucleophiles represent distinct stabilization modes and steric environments: a 1,3-dicarbonyl-stabilized carbanion Nu1, a phenylsulfonyl bromomethyl carbanion Nu2, an α-cyano benzylic carbanion Nu3, and an allylic carbon nucleophile Nu4 (Figure 2b). The full set comprises 95 substrates and 380 aromatic nucleophilic-addition combinations.

Solution-phase reaction free energies were calculated for all 380 substrate–nucleophile combinations. The free energy of Meisenheimer intermediate formation was defined relative to the separated optimized aromatic substrate and nucleophile:

Δ*G*_rxn_ = *G*_MI_ − (*G*_Ar_ + *G*_Nu_) (1)

In Equation (1), MI, Ar, and Nu denote the Meisenheimer intermediate, aromatic substrate, and nucleophile, respectively, and each *G* term is a solution-phase Gibbs free energy. Geometry optimizations and frequency calculations were performed at M06-2X/def2-SVP [45,46] with PCM(DME) solvation [47], and thermal corrections were evaluated at 298.15 K; M06-2X/def2-TZVPP single-point calculations used the same PCM(DME) treatment. DME was implemented as a user-defined PCM solvent; its dielectric constant, optical dielectric constant, solvent radius, and density are specified in the Supporting Information. Table S2 lists the uncorrected Gaussian thermochemical *G* values, and −1.89 kcal/mol was applied to the derived bimolecular reaction and activation free energies for conversion to the 1 mol/L standard state. The M06-2X protocol was selected to compare relative thermochemistry and calculated addition barriers at one consistent level across the full dataset. In a benchmark of carbanion addition to substituted nitroarenes, Błaziak et al. found that M06-2X reproduced the experimental relative-rate trend for the para series (*R*^2^ = 0.91 for activation free energy), matching the performance of PBE1PBE [48]. This literature benchmark supports the use of M06-2X for relative thermochemical and barrier trends in aromatic nucleophilic addition. The calculated Δ*G*_rxn_ values span −90.1 to +33.9 kcal/mol, with a median of −6.8 kcal/mol (Figure 2c). This 124.0 kcal/mol range reflects the combined effects of scaffold, substitution pattern, reactive position, and nucleophile identity.

**Figure 3:**
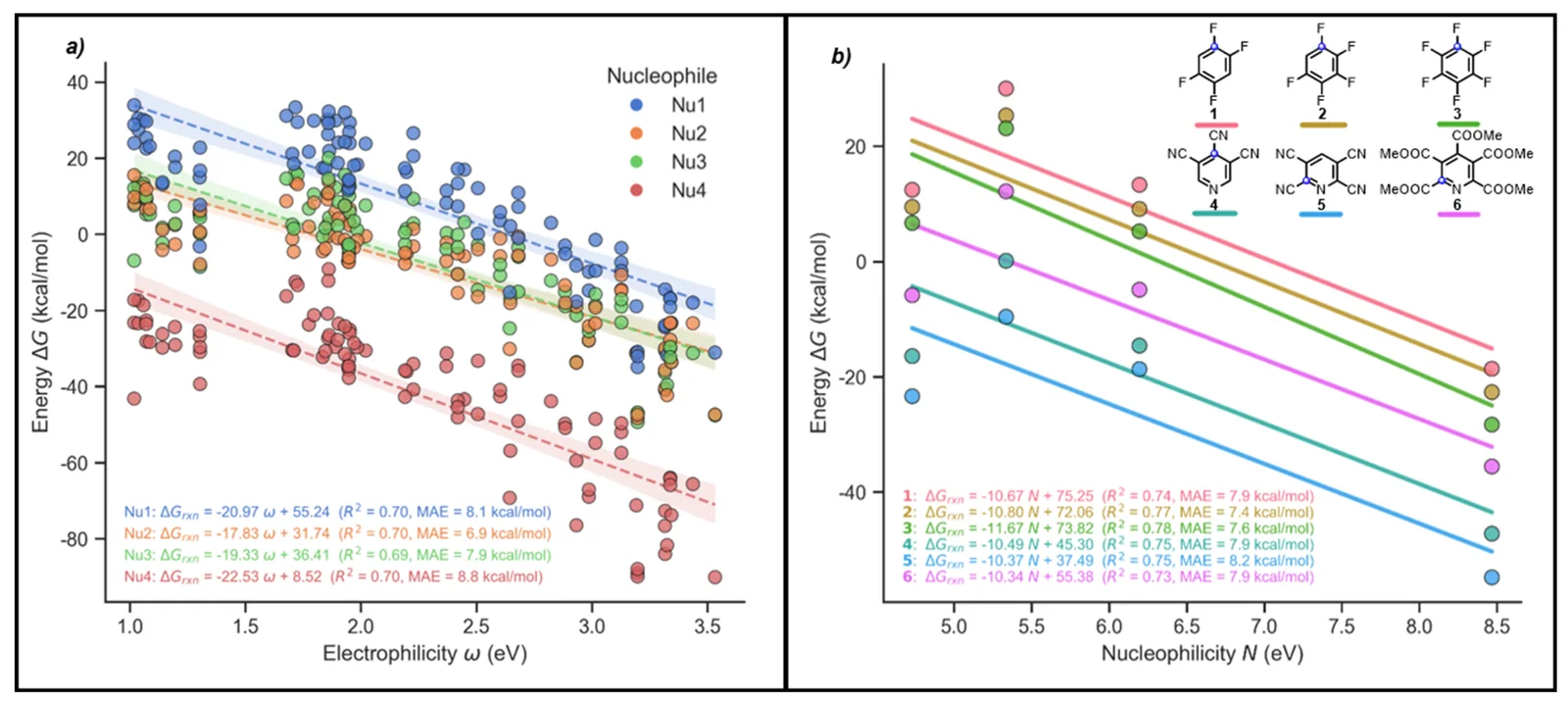
Descriptor relationships for reaction free energies. (**a**) Relationship between Δ*G*_rxn_ and substrate electrophilicity *ω* for fixed nucleophiles. (**b**) Representative relationships between Δ*G*_rxn_ and nucleophilicity index *N*; the four-point fits describe within-dataset trends for the sampled nucleophiles.

For each fixed nucleophile, Δ*G*_rxn_ decreases as substrate electrophilicity *ω* [49] increases (Figure 3a). The four regressions give *R*^2^ values of 0.69–0.70 and mean absolute errors of 6.9–8.8 kcal/mol. Electrophilicity captures the direction of the response and supports coarse triage at the extremes of the reaction space. The observed errors preclude classification near a thermodynamic boundary or prediction of an individual Δ*G*_rxn_. These comparisons use indices calculated consistently within the present electronic-structure protocol.

The complementary effect of nucleophile strength was evaluated for fixed aromatic substrates. One fixed-skeleton series was selected from each substitution-number class of the benzene (Ph) and pyridine (Py) scaffolds, choosing the four-point fit whose *R*^2^ value was closest to the class median. These six series illustrate the relationship between Δ*G*_rxn_ and the nucleophilicity index *N* [50] (Figure 3b), and Figure S2 reports the complete set of fits and the *R*^2^ distribution. Across the sampled fixed-substrate series, increasing *N* produces more favorable addition free energies and therefore captures the expected within-scaffold ordering. Because each regression contains only four nucleophiles, the slopes describe the present chemical set.

The descriptor fits recover the leading electronic response but leave substantial variation associated with scaffold identity, substitution topology, steric environment, and local resonance. They are therefore used to organize the calculated space and identify cases requiring explicit reaction-energy calculations. Positional substituent effects were examined separately through matched Hammett series.

Representative fixed-skeleton series were compared with Hammett constants in the literature [51,52,53,54]. Every fitted slope was negative, consistent with electron-withdrawing substitution favoring σ-complex formation within each matched series. Because classical meta/para constants omit short-range ortho contributions, the Σσ_m,p_ treatment was compared with Σσ_H_ = Σσ_m,p_ + *n_o_* · *σ_I_*, where *n_o_* is the number of symmetry-equivalent ortho positions occupied by the varied substituent in the fixed skeleton and *σ_I_* is the field/inductive constant of the varied substituent. Adding the ortho-inductive term increased *R*^2^ by 0.019–0.144 in all 20 ortho-containing Δ*G*_rxn_ series. We therefore use Σσ_H_ as the positional treatment for these reaction-free-energy series. The adopted constants and full fitted relationships are reported in the Supporting Information (Figures S7 and S8).

### 2.2. Kinetic Accessibility of Meisenheimer Intermediate Formation

Thermodynamic favorability determines whether a σ-complex can be populated but leaves the kinetic accessibility of nucleophilic addition unresolved. We anchored the kinetic subset to the experimentally isolated ester-stabilized Meisenheimer complex rather than selecting only the most exergonic reactions [31]. This reference-centered design compares combinations with similar driving forces before their barriers are evaluated.

**Figure 4:**
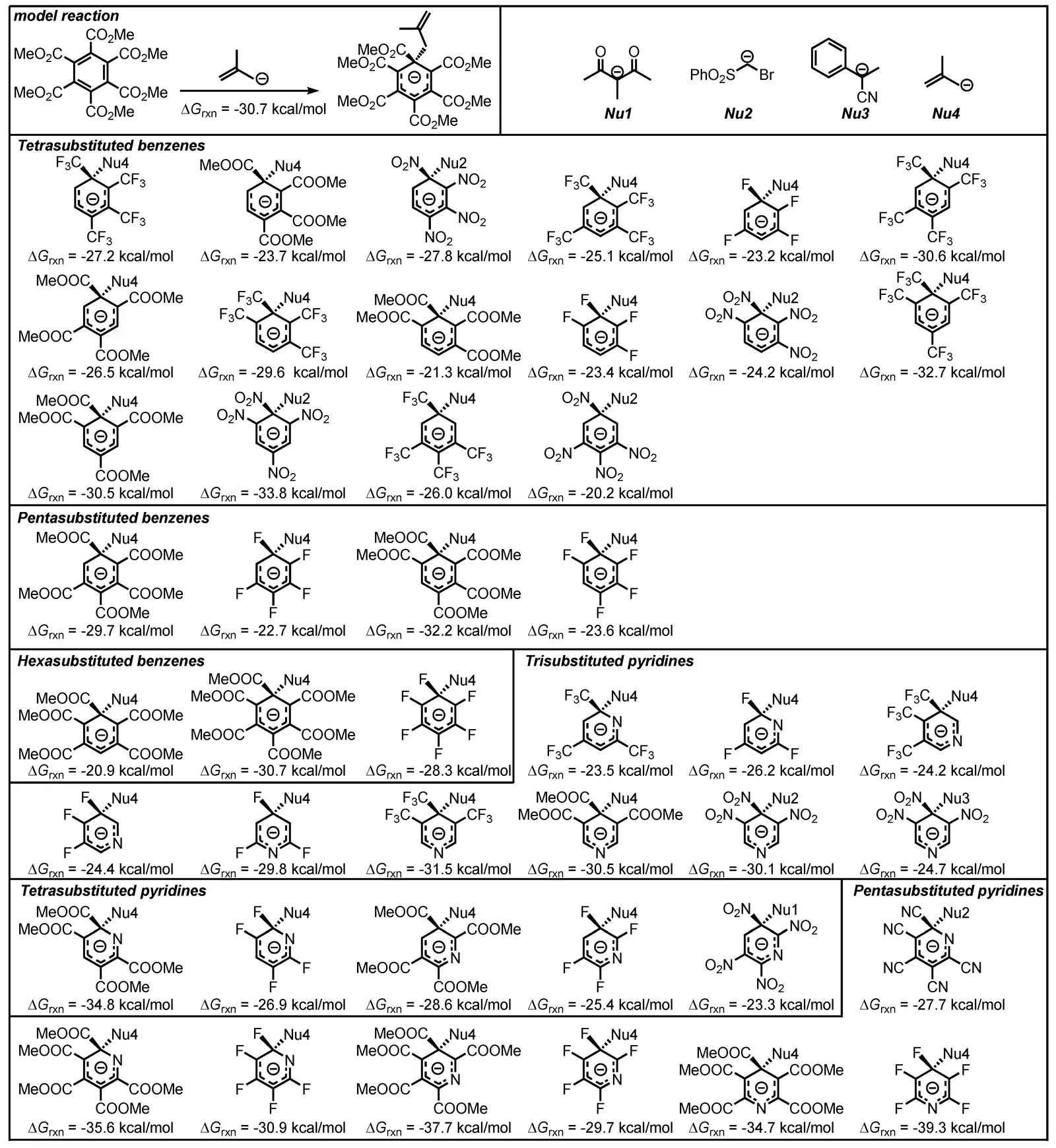
Thermodynamically selected substrate–nucleophile combinations for which nucleophilic-addition transition states were located. The experimentally reported model reaction and the structures of Nu1–Nu4 are shown in the upper panel. The candidate window was Δ*G*_rxn_ = −40 to −20 kcal/mol. Candidates in this window for which a transition state was not located are provided in the Supporting Information.

The calculated reaction free energy of the experimental reference is Δ*G*_rxn_ = −30.7 kcal/mol (Figure 4). We defined a −40 to −20 kcal/mol window that brackets the reference by approximately 10 kcal/mol on either side and used it to select transition-state searches. The window is tied to this reference reaction rather than proposed as a universal cutoff for Meisenheimer chemistry.

The −40 to −20 kcal/mol thermodynamic window contains 84 substrate–nucleophile combinations. Transition-state searches located 44 first-order saddle points and did not locate the intended saddle point for 40 combinations, corresponding to a success rate of 52% (44/84). Each located transition state has one imaginary mode dominated by the formation of the new C–C bond. The kinetic subset is not random: 36 of the 44 located cases involve Nu4, whereas 29 of the 40 unlocated cases involve Nu1–Nu3. The median Δ*G*_rxn_ values are −27.8 and −31.2 kcal/mol for the located and unlocated groups, respectively. These differences show that convergence and nucleophile class affect the membership of the kinetic dataset. No saddle-point energies are available for the unlocated cases, so their kinetic ordering remains unknown. Figure S6 lists the 40 combinations for which the intended transition state was not located.

The activation free energy for nucleophilic addition, relative to the same separated reactants used in Equation (1), was defined as:

(2) $${\Delta G}_{\mathrm{TS}}^{‡}=G_{\mathrm{TS}}-(G_{\mathrm{Ar}}+G_{\mathrm{Nu}})$$

**Figure 5:**
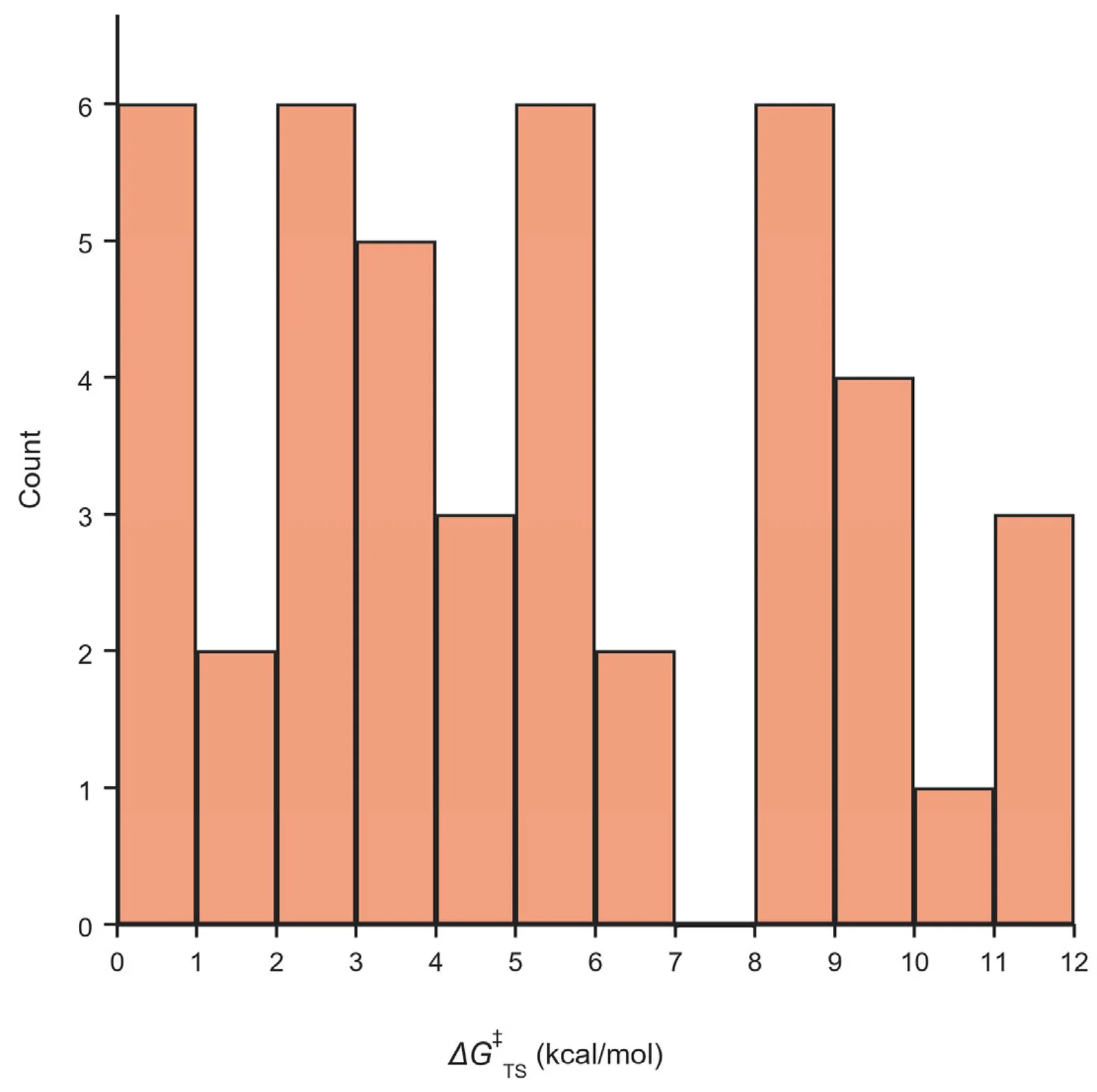
Histogram of calculated nucleophilic-addition barriers ${\Delta G}_{\mathrm{TS}}^{‡}$ for the 44 located transition states in the selected moderate-exergonicity subset.

In Equation (2), TS denotes the nucleophilic-addition transition state. Across the 44 located saddle points, ${\Delta G}_{\mathrm{TS}}^{‡}$ ranges from 0.4 to 11.4 kcal/mol, with a median of 5.1 kcal/mol (Figure 5). Each transition state was confirmed as a first-order saddle point by a single imaginary frequency dominated by formation of the new C–C bond. All 44 located saddle points were included in the kinetic analysis, and the final candidates were selected solely by the thermodynamic window and reference-relative barrier criterion. Additional stationary-point validation is documented in the Supporting Information.

Within the 44-case kinetic dataset, ${\Delta G}_{\mathrm{TS}}^{‡}$ shows no useful linear relationship with Δ*G*_rxn_. The sampled energy window is deliberately narrow, and transition states were located for 52% of its combinations, so this result should not be extrapolated to the full reaction space. For the cases actually compared, however, reaction free energy does not rank the addition barriers; kinetic discrimination requires explicit transition-state calculations.

Reaction free energy defines the thermodynamically admissible set, after which the addition barrier distinguishes kinetic accessibility. Similar reaction free energies can accompany different barriers, so the two quantities serve complementary roles in the screening sequence.

### 2.3. Substrate–Nucleophile Compatibility in Meisenheimer Intermediate Formation

**Figure 6:**
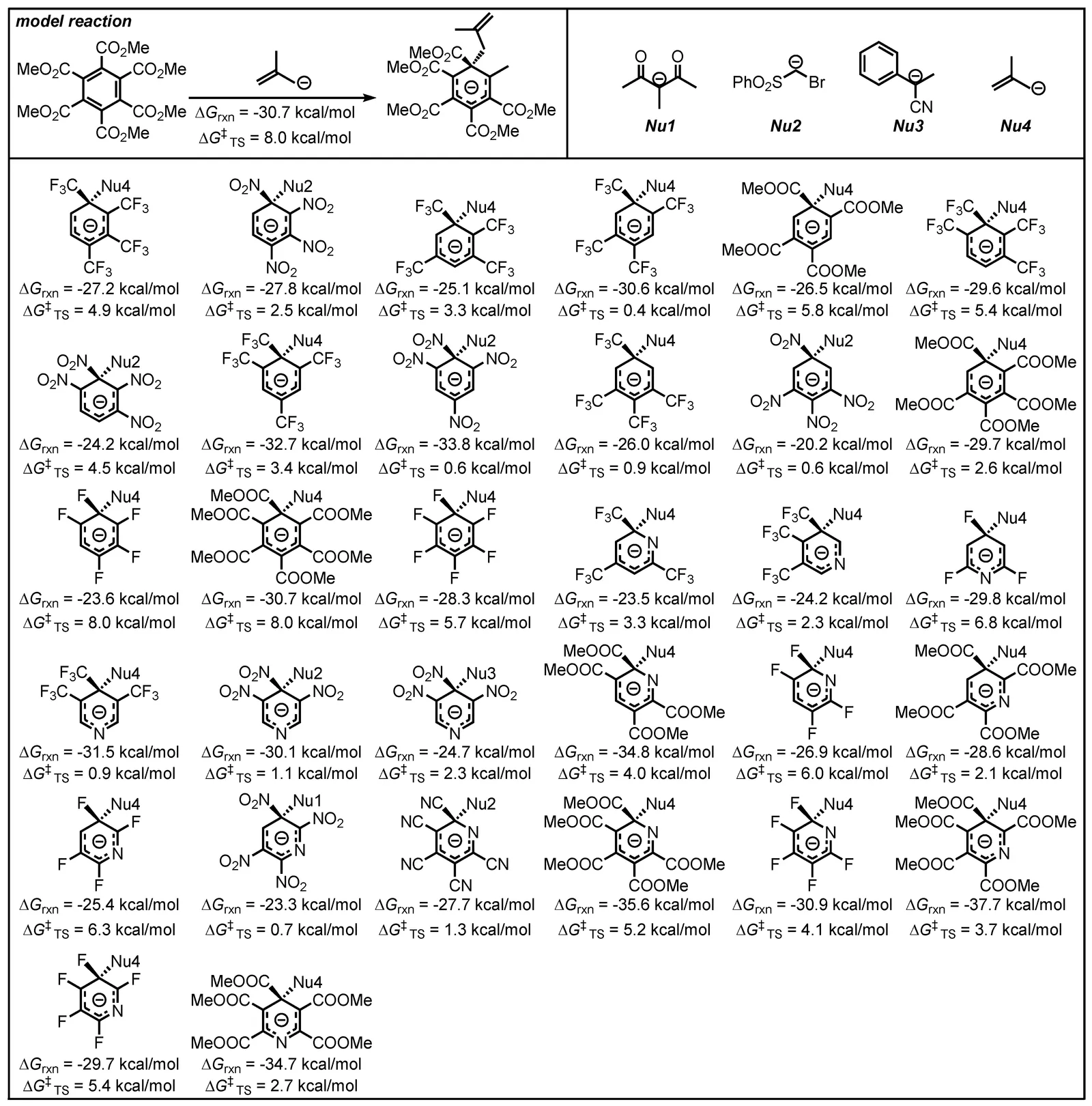
Thirty-two substrate–nucleophile combinations within the thermodynamic window Δ*G*_rxn_ = −40 to −20 kcal/mol and with ${\Delta G}_{\mathrm{TS}}^{‡}$ no higher than that of the experimental reference (8.0 kcal/mol). Candidate selection used unrounded energies; displayed values are reported to one decimal place. The model reaction and the structures of Nu1–Nu4 are shown in the upper panel. The structure-coding convention is defined in Figure S1, and the corresponding energy data are listed in Table S2.

Applying the two energetic criteria identifies 32 combinations with located transition states (Figure 6). Each lies in the Δ*G*_rxn_ = −40 to −20 kcal/mol window and has a ${\Delta G}_{\mathrm{TS}}^{‡}$ no higher than that of the experimental reference (8.0 kcal/mol). Candidate selection used unrounded energies; displayed values are reported to one decimal place. The thermodynamic and kinetic criteria are applied sequentially, with the same experimental reaction serving as their common reference.

For readability, the examples below are described by their aromatic scaffold, substituent at the addition site, and nucleophile; their full structure codes are shown in Figure 6 and Table S2 and defined in Figure S1.

The retained set comprises 32 combinations across the benzene and pyridine scaffolds: 24 with Nu4, 6 with Nu2, and 1 each with Nu1 and Nu3. All four nucleophile classes are represented, although the set is dominated by Nu4, reflecting the composition of the located-transition-state subset. Several entries illustrate different matching regimes. The tetra-substituted benzene with CF_3_ at the addition site in combination with Nu4 combines Δ*G*_rxn_ = −30.6 kcal/mol with ${\Delta G}_{\mathrm{TS}}^{‡}$ = 0.4 kcal/mol, whereas a tetra-substituted benzene with NO_2_ at the addition site in combination with Nu2 gives Δ*G*_rxn_ = −33.8 kcal/mol and ${\Delta G}_{\mathrm{TS}}^{‡}$ = 0.6 kcal/mol. Among the pyridines, a tetra-substituted pyridine with NO_2_ at the addition site in combination with Nu1 (−23.3 and 0.7 kcal/mol) shows that an activated heteroaromatic scaffold can enter the selected window with Nu1, while a penta-substituted pyridine with CN at the addition site in combination with Nu2 (−27.7 and 1.3 kcal/mol) provides a non-nitro example involving Nu2. These examples show how the two energetic criteria retain chemically distinct substrate–nucleophile pairings.

Across the selected set, thermodynamic driving force and the addition barrier must be considered together. Combinations with similar Δ*G*_rxn_ values can have different barriers, and ring nitrogen changes which nucleophile–substituent combinations fall within the reference-centered window. Explicit transition-state calculations therefore alter the priorities obtained from thermodynamic screening alone.

The 32 combinations define an experimental priority set based on Meisenheimer intermediate formation free energy and the calculated addition barrier. Experimental evaluation will determine how speciation and reaction conditions affect intermediate isolation and downstream synthetic utility.

## 3. Conclusions

Across the 380 substrate–nucleophile combinations, aromatic scaffold, substitution topology, reactive site, and nucleophile identity jointly determine the calculated accessibility of Meisenheimer intermediates. Electrophilicity and nucleophilicity reproduce the direction of the main thermodynamic response, whereas explicit reaction-energy calculations are required to distinguish individual combinations. The matched Hammett series further show that short-range ortho induction contributes measurably in multisubstituted substrates.

Transition states were located for 44 of the 84 combinations in the reference-centered thermodynamic window, and their calculated addition barriers span 0.4–11.4 kcal/mol. Within this located subset, reaction free energy does not rank the addition barriers; thermodynamic screening alone therefore cannot identify the kinetically preferred combinations. Applying the thermodynamic window and the reference-relative barrier criterion identifies 32 benzene- and pyridine-based combinations for experimental evaluation. The selected set provides concrete experimental targets and shows that accessible Meisenheimer intermediates arise from a matched combination of scaffold activation, substitution pattern, and nucleophile strength.

## Data Availability

The data supporting the conclusions of this study are provided in the article and Supplementary Materials. Additional calculation input and output files are available from the corresponding authors upon reasonable request.

## Supplementary Materials

The following supporting information can be downloaded at: https://www.mdpi.com/article/10.3390/molecules31173114/s1, Computational details, method scope and literature support, custom PCM parameters for DME, conceptual DFT definitions, complete Δ*G*_rxn_–*N* and Hammett analyses including the ortho-inductive treatment, additional stationary-point validation and descriptor-method checks, unlocated transition-state candidates, calculated energies, and Cartesian coordinates of the computed species are provided in the Supporting Information. References [46,47,48,49,51,52,53,54,55,56,57,58,59,60,61] are cited in the supplementary materials. Figure S1. Nomenclature rules for the substrates and Meisenheimer intermediates. (a) Aromatic skeleton codes. Ph and Py denote benzene- and pyridine-derived cores, respectively. Skeleton labels follow the format core–substitution count–position sequence, with the reactive/substituted site listed first. (b) Codes for substituents (R1–R5) and nucleophiles (Nu1–Nu4). (c) Representative substrate and Meisenheimer intermediate codes. Figure S2. (a) Linear fits of Δ*G*_rxn_ against *N* for all fixed substrate/reactive-site cases. (b) Distribution of the corresponding *R*^2^ values. Figure S3. Representative bidirectional IRC profile for TS-Ph-5-12345-R4-Nu4, calculated at M06-2X/def2-SVP with PCM(DME). The forward direction reaches the reactant side (82 accepted points), and the reverse direction reaches the Meisenheimer intermediate (200 accepted points); both calculations terminated normally. Electronic energies are reported relative to the transition state. Figure S4. Representative bidirectional IRC profile for TS-Py-3-435-R3-Nu4, calculated at M06-2X/def2-SVP with PCM(DME). The forward trajectory extends toward the reactant side for 188 accepted points before stopping during the next point, whereas the reverse trajectory reaches the Meisenheimer intermediate with 200 accepted points and normal termination. Electronic energies are reported relative to the transition state. Figure S5. Representative reactant- and product-side structures obtained by manual displacement and endpoint optimization for Nu1–Nu3 transition states. The transition-state geometries were displaced by a maximum Cartesian amplitude of 0.30 Å in both directions along the imaginary-frequency eigenvector and optimized at M06-2X/def2-SVP with PCM(DME). Panels (a)–(c) show Py-4-3256-R5-Nu1, Ph-4-2135-R5-Nu2, and Py-3-435-R5-Nu3, respectively. Figure S6. The 40 substrate–nucleophile combinations within the −40 to −20 kcal/mol thermodynamic window for which a nucleophilic-addition transition state was not located with the employed search protocol. Together with the 44 located cases, these structures account for all 84 combinations in the window. Figure S7. Fixed-skeleton relationships between Δ*G*_rxn_ and the classical meta/para descriptor Σσ_m,p_ for the six representative benzene and pyridine skeletons. Each line comprises five substituent variants for one nucleophile. Ortho substituents are omitted from Σσ_m,p_. Figure S8. Fixed-skeleton relationships obtained with the ortho-sensitive descriptor Σσ_H_ = Σσ_m,p_ + *n_o_**σ_I_*. Each panel reports the fitted equation, *R*^2^, and mean absolute error. Py-3-246 contains no varied ortho position; its classical and extended fits are therefore identical. Figure S9. Direct comparison of the M06-2X and ωB97X-D electrophilicity indices for 95 substrates. The coefficients of determination and rank correlation are *R*^2^ = 0.995 and Spearman ρ = 0.993, respectively, confirming that the substrate electrophilicity ordering is retained across the two functionals. Figure S10. Comparison of the four global Δ*G*_rxn_-*ω* *R*^2^ values obtained with M06-2X and ωB97X-D. Each paired bar represents one fixed-nucleophile regression over 95 substrates; the vertical axis is expanded to resolve the small differences. The closely clustered values confirm that the global substrate-electrophilicity relationship is retained after the functional is changed. Table S1. Summary of fixed-substrate Δ*G*_rxn_–*N* fit statistics obtained with M06-2X and ωB97X-D. Table S2. Uncorrected Gaussian thermochemical values used to derive the energies in Figure 2, Figure 3, Figure 4, Figure 5 and Figure 6, Figures S2, S7 and S8: zero-point energy (ZPE), thermal correction to enthalpy (TCH), thermal correction to Gibbs free energy (TCG), electronic energy (E), enthalpy (H), and Gibbs free energy (G), all in Hartree, calculated at M06-2X/def2-TZVPP-PCM(DME)//M06-2X/def2-SVP-PCM(DME). The tabulated G values do not include the 1 mol/L standard-state conversion; −1.89 kcal/mol must be applied to each derived bimolecular reaction or activation free energy. ZPE, TCH, and TCG were obtained from M06-2X/def2-SVP/PCM(DME) frequency calculations, whereas E values were obtained from M06-2X/def2-TZVPP/PCM(DME) single-point calculations. Blank imaginary-frequency entries denote minima with no imaginary frequency.

## References

[1] Rohrbach S., Smith A.J., Pang J.H., Poole D.L., Tuttle T., Chiba S., Murphy J.A. (2019). Concerted nucleophilic aromatic substitution reactions. Angew. Chem. Int. Ed..

[2] Kwan E.E., Zeng Y., Besser H.A., Jacobsen E.N. (2018). Concerted nucleophilic aromatic substitutions. Nat. Chem..

[3] Lennox A.J.J. (2018). Meisenheimer complexes in S_N_Ar reactions: Intermediates or transition states?. Angew. Chem. Int. Ed..

[4] Rohrbach S., Murphy J.A., Tuttle T. (2020). Computational study on the boundary between the concerted and stepwise mechanism of bimolecular S_N_Ar reactions. J. Am. Chem. Soc..

[5] Ormazábal-Toledo R., Richter S., Robles-Navarro A., Maulén B., Matute R.A., Gallardo-Fuentes S. (2020). Meisenheimer complexes as hidden intermediates in the aza-S_N_Ar mechanism. Org. Biomol. Chem..

[6] Błaziak K., Danikiewicz W., Mąkosza M. (2016). How does nucleophilic aromatic substitution really proceed in nitroarenes? Computational prediction and experimental verification. J. Am. Chem. Soc..

[7] Błaziak K., Danikiewicz W., Mąkosza M. (2020). How do aromatic nitro compounds react with nucleophiles? Theoretical description using aromaticity, nucleophilicity and electrophilicity indices. Molecules.

[8] Mąkosza M. (2020). Electrophilic and nucleophilic aromatic substitutions are mechanistically similar with opposite polarity. Chem. Eur. J..

[9] Domingo L.R., Ríos-Gutiérrez M., Chamorro E., Pérez P. (2019). Are one-step aromatic nucleophilic substitutions of non-activated benzenes concerted processes?. Org. Biomol. Chem..

[10] Wertjes W.C., Southgate E.H., Sarlah D. (2018). Recent advances in chemical dearomatization of nonactivated arenes. Chem. Soc. Rev..

[11] Zheng C., You S.-L. (2021). Advances in catalytic asymmetric dearomatization. ACS Cent. Sci..

[12] Escolano M., Gaviña D., Alzuet-Piña G., Díaz-Oltra S., Sánchez-Roselló M., del Pozo C. (2024). Recent strategies in the nucleophilic dearomatization of pyridines, quinolines, and isoquinolines. Chem. Rev..

[13] Zhuo C.-X., Zhang W., You S.-L. (2012). Catalytic asymmetric dearomatization reactions. Angew. Chem. Int. Ed..

[14] Bertuzzi G., Bernardi L., Fochi M. (2018). Nucleophilic dearomatization of activated pyridines. Catalysts.

[15] García Mancheño O., Asmus S., Zurro M., Fischer T. (2015). Highly enantioselective nucleophilic dearomatization of pyridines by anion-binding catalysis. Angew. Chem. Int. Ed..

[16] Yang Z.-P., Wu Q.-F., Shao W., You S.-L. (2015). Iridium-catalyzed intramolecular asymmetric allylic dearomatization reaction of pyridines, pyrazines, quinolines, and isoquinolines. J. Am. Chem. Soc..

[17] Wang D., Wang Z., Liu Z., Huang M., Hu J., Yu P. (2019). Strategic C-C bond-forming dearomatization of pyridines and quinolines. Org. Lett..

[18] Zhang H.-J., Yang Z.-P., Gu Q., You S.-L. (2019). Tandem Pd-catalyzed intermolecular allylic alkylation/allylic dearomatization reaction of benzoylmethyl pyridines, pyrazines, and quinolines. Org. Lett..

[19] Wu W.-T., Xu R.-Q., Zhang L., You S.-L. (2016). Construction of spirocarbocycles via gold-catalyzed intramolecular dearomatization of naphthols. Chem. Sci..

[20] Zuo Z., Yang X., Liu J., Nan J., Bai L., Wang Y., Luan X. (2015). Ru(II)-catalyzed oxidative spiroannulation of 2-arylphenols with alkynes via a C-H activation/dearomatization strategy. J. Org. Chem..

[21] Han L., Wang H., Luan X. (2018). Pd(II)-catalyzed [3 + 2] spiroannulation of α-aryl-β-naphthols with alkynes via a C-H activation/dearomatization approach. Org. Chem. Front..

[22] Fang X., Zeng Y., Li Q., Wu Z., Yao H., Lin A. (2018). Redox-neutral atom-economic Pd(0)-catalyzed dearomatization of β-naphthols with alkynes toward naphthalenones. Org. Lett..

[23] An J., Parodi A., Monari M., Reis M.C., López C.S., Bandini M. (2017). Gold-catalyzed dearomatization of 2-naphthols with alkynes. Chem. Eur. J..

[24] Ito T., Harada S., Homma H., Takenaka H., Hirose S., Nemoto T. (2021). Asymmetric intramolecular dearomatization of nonactivated arenes with ynamides for rapid assembly of fused ring system under silver catalysis. J. Am. Chem. Soc..

[25] Mao J.-H., Wang Y.-B., Yang L., Xiang S.-H., Wu Q.-H., Cui Y., Lu Q., Lv J., Li S., Tan B. (2021). Organocatalyst-controlled site-selective arene C-H functionalization. Nat. Chem..

[26] Wang Y., Li R., Guan W., Li Y., Li X., Yin J., Zhang G., Zhang Q., Xiong T., Zhang Q. (2020). Organoborohydride-catalyzed Chichibabin-type C4-position alkylation of pyridines with alkenes assisted by organoboranes. Chem. Sci..

[27] Zeng W.-L., Xia Q., Li C.-Q., Wang M.-Y., Jin W.-Y., Ding H., Li W. (2024). Bench-stable Meisenheimer complexes: Synthesis, characterization, and divergent reactivity for dearomatization. J. Am. Chem. Soc..

[28] Mąkosza M. (2010). Nucleophilic substitution of hydrogen in electron-deficient arenes, a general process of great practical value. Chem. Soc. Rev..

[29] Mąkosza M. (2011). Nucleophilic substitution of hydrogen in nitroarenes: A new chapter of aromatic chemistry. Synthesis.

[30] Mąkosza M., Wojciechowski K., Charushin V., Chupakhin O. (2013). Nucleophilic Substitution of Hydrogen in Arenes and Heteroarenes. Metal Free C-H Functionalization of Aromatics.

[31] Micheletti G., Boga C. (2017). Nucleophile/electrophile combinations in aromatic substitution: From Wheland to Wheland-Meisenheimer intermediates using strongly activated arenes. Synthesis.

[32] Forlani L., Boga C., Mazzanti A., Zanna N. (2012). Trapping and analysing Wheland-Meisenheimer sigma complexes, usually labile and escaping intermediates. Eur. J. Org. Chem..

[33] Mandler M.D., Suss N., Ramirez A., Farley C.A., Aulakh D., Zhu Y., Traeger S.C., Sarjeant A., Davies M.L., Ellsworth B.A. (2022). Amination of nitro-substituted heteroarenes by nucleophilic substitution of hydrogen. Org. Lett..

[34] Zhang Z., Cao X., Song X., Wang G., Shi B., Li X., Ma N., Liu L., Zhang G. (2023). Metal-free nucleophilic 7,8-dearomatization of quinolines: Spiroannulation of aminoquinoline protected amino acids. Chin. Chem. Lett..

[35] Zhang N., Ye Y., Bai L., Liu J., Wang H., Luan X. (2022). Transition metal-free dearomatization of halonaphthols with C(sp^3^)-electrophiles. Chin. Chem. Lett..

[36] Zhang Y., Luo H., Lu Q., An Q., Li Y., Li S., Tang Z., Li B. (2021). Access to pyridines via cascade nucleophilic addition reaction of 1,2,3-triazines with activated ketones or acetonitriles. Chin. Chem. Lett..

[37] Zhong G., Huang Y., He L. (2023). Regioselectivity of N-heteroarene electrocarboxylations: Divided vs. undivided cell. Chem. Synth..

[38] Liljenberg M., Brinck T., Herschend B., Rein T., Tomasi S., Svensson M. (2012). Predicting regioselectivity in nucleophilic aromatic substitution. J. Org. Chem..

[39] Liljenberg M., Brinck T., Rein T., Svensson M. (2013). Utilizing the σ-complex stability for quantifying reactivity in nucleophilic substitution of aromatic fluorides. Beilstein J. Org. Chem..

[40] Lu J., Paci I., Leitch D.C. (2022). A broadly applicable quantitative relative reactivity model for nucleophilic aromatic substitution (S_N_Ar) using simple descriptors. Chem. Sci..

[41] Jorner K., Brinck T., Norrby P.-O., Buttar D. (2021). Machine learning meets mechanistic modelling for accurate prediction of experimental activation energies. Chem. Sci..

[42] Vahl M., Proppe J. (2023). The computational road to reactivity scales. Phys. Chem. Chem. Phys..

[43] Luo X., Zhong K., Lan Y. (2020). Mechanism of palladium-catalyzed spiroannulation of naphthols with alkynes: A density functional theory study. ChemCatChem.

[44] Zhang M., Huang G. (2016). Mechanism and selectivity of Ru(II)- and Rh(III)-catalyzed oxidative spiroannulation of naphthols and phenols with alkynes through a C-H activation/dearomatization strategy. Chem. Eur. J..

[45] Zhao Y., Truhlar D.G. (2008). Density functionals with broad applicability in chemistry. Acc. Chem. Res..

[46] Weigend F., Ahlrichs R. (2005). Balanced basis sets of split valence, triple zeta valence and quadruple zeta valence quality for H to Rn: Design and assessment of accuracy. Phys. Chem. Chem. Phys..

[47] Scalmani G., Frisch M.J. (2010). Continuous surface charge polarizable continuum models of solvation. I. General formalism. J. Chem. Phys..

[48] Błaziak K., Świder P., Mąkosza M., Danikiewicz W. (2025). Relative rates of addition of carbanions to substituted nitroarenes: Can quantum chemical calculations give meaningful predictions?. Phys. Chem. Chem. Phys..

[49] Parr R.G., Szentpály L.V., Liu S. (1999). Electrophilicity index. J. Am. Chem. Soc..

[50] Domingo L.R., Pérez P. (2011). The nucleophilicity N index in organic chemistry. Org. Biomol. Chem..

[51] Hammett L.P. (1937). The effect of structure upon the reactions of organic compounds. Benzene derivatives. J. Am. Chem. Soc..

[52] Jaffé H.H. (1953). A reëxamination of the Hammett equation. Chem. Rev..

[53] Hansch C., Leo A., Taft R.W. (1991). A survey of Hammett substituent constants and resonance and field parameters. Chem. Rev..

[54] Taft R.W. (1952). Polar and steric substituent constants for aliphatic and o-benzoate groups from rates of esterification and hydrolysis of esters. J. Am. Chem. Soc..

[55] Frisch M.J., Trucks G.W., Schlegel H.B., Scuseria G.E., Robb M.A., Cheeseman J.R., Scalmani G., Barone V., Petersson G.A., Nakatsuji H. (2016). Gaussian 16, Revision A.03.

[56] Zhao Y., Truhlar D.G. (2008). The M06 suite of density functionals for main group thermochemistry, thermochemical kinetics, noncovalent interactions, excited states, and transition elements: Two new functionals and systematic testing of four M06-class functionals and 12 other functionals. Theor. Chem. Acc..

[57] Weigend F. (2006). Accurate Coulomb-fitting basis sets for H to Rn. Phys. Chem. Chem. Phys..

[58] Cancés E., Mennucci B., Tomasi J. (1997). A new integral equation formalism for the polarizable continuum model: Theoretical background and applications to isotropic and anisotropic dielectrics. J. Chem. Phys..

[59] Domingo L.R., Chamorro E., Pérez P. (2008). Understanding the reactivity of captodative ethylenes in polar cycloaddition reactions: A theoretical study. J. Org. Chem..

[60] Taft R.W. (1952). Linear free energy relationships from rates of esterification and hydrolysis of aliphatic and ortho-substituted benzoate esters. J. Am. Chem. Soc..

[61] Charton M. (1969). Nature of the ortho effect. V. Ortho-substituent constants. J. Am. Chem. Soc..

