# Supplementary material for: Computational Thermodynamic and Kinetic Analysis of Meisenheimer Intermediate Formation in Aromatic Nucleophilic Addition
Source: Molecules. 2026 Sep 5;31(17):3114. doi: 10.3390/molecules31173114 (PMC13567624; doi:10.3390/molecules31173114)
Supplement: Supplementary file 1 [file molecules-31-03114-s001.zip › molecules-4411721-supplementary.pdf]

Supporting Information

Computational Thermodynamic and Kinetic Analysis of Meisenheimer Intermediate Formation in Aromatic Nucleophilic Addition

Cheng Wang [1], Rui Liu [1], Shuo-Qing Zhang [1,*] and Xin Hong [1,2,*]

1 Center of Chemistry for Frontier Technologies, Department of Chemistry, Zhejiang University, Hangzhou 310027, P. R. China

2 School of Chemistry and Chemical Engineering, Henan Normal University, Xinxiang 453007, P. R. China

* Correspondence: angellasty@zju.edu.cn (S.-Q.Z.); hxchem@zju.edu.cn (X.H.)

Contents

Computational Details 2

Nomenclature Rules for the Structures 3

Calculation of Conceptual DFT Indices 3

Overview of ΔG vs N Relationships 4

IRC Connectivity Analysis 5

Unlocated-Transition-State Candidates 9

Hammett Substituent-Constant Analysis 10

ωB97X-D CDFT Method-Sensitivity Analysis 13

References 16

Table of Energies 17

Cartesian Coordinates of Computed Species 28

Computational Details

All density functional theory (DFT) calculations were carried out with Gaussian 16 (Revision A.03) [1]. Unless noted otherwise, the default Gaussian 16 self-consistent-field convergence criterion (scf=tight), geometry-optimization convergence criteria, and integral-grid settings (int=(ultrafine,acc2e=12)) were used. Geometry optimizations were performed with the M06-2X functional [2] and the def2-SVP basis set [3] for all atoms. Single-point energies were evaluated at the M06-2X/def2-TZVPP level [4] with the same PCM(DME) treatment on the optimized geometries. Solvent effects in 1,2-dimethoxyethane (DME) were included for both optimizations and single-point calculations with the self-consistent reaction-field (SCRF) approach and the polarizable continuum model (PCM) [5,6]. Harmonic frequency calculations were performed at the geometry-optimization level to verify the nature of each stationary point and to obtain the thermal corrections.

Because DME was not included as a predefined solvent in the Gaussian 16 PCM solvent list used here, it was specified as a user-defined solvent. The parameters were set to EPS = 7.20, EPSINF = 1.902, RSOLV = 3.42, and DENSITY = 0.8689.

The G values listed in Table S2 are the uncorrected Gaussian thermochemical values at the 1 atm standard state. Conversion to the 1 mol/L solution standard state adds $RT\ln(c^{\circ}_{\mathrm{sol}}/c^{\circ}_{\mathrm{gas}})$ = 1.89 kcal/mol to each solute species at 298.15 K, where c°_{sol} = 1 mol/L and c°_{gas} = 0.040876 mol/L. Because both the reaction and activation free energies describe association of two separated reactants into one species, the net correction to the derived ΔG_{rxn} and $\Delta G^{\ddagger}_{\mathrm{TS}}$ values is −1.89 kcal/mol. Direct subtraction of the Table S2 G values must therefore be followed by this correction to reproduce the energies reported in the main text.

Nomenclature Rules for the Structures

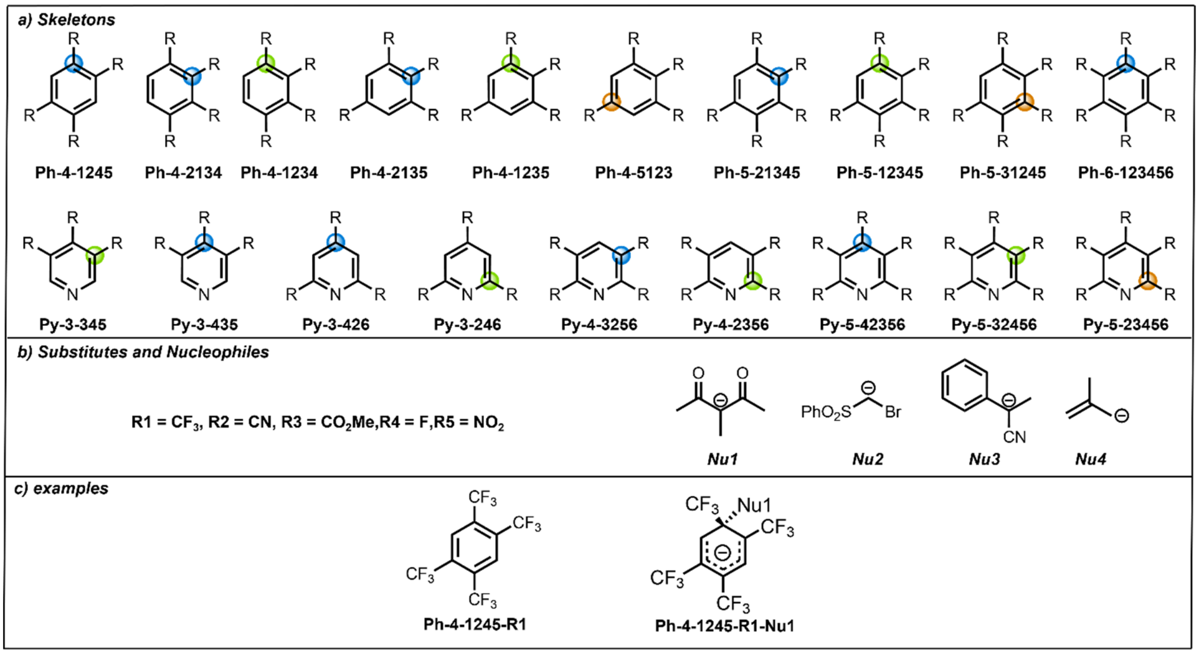

Figure S1. Nomenclature rules for the substrates and Meisenheimer intermediates. (a) Aromatic skeleton codes. Ph and Py denote benzene- and pyridine-derived cores, respectively. Skeleton labels follow the format core–substitution count–position sequence, with the reactive/substituted site listed first. (b) Codes for substituents (R1–R5) and nucleophiles (Nu1–Nu4). (c) Representative substrate and Meisenheimer intermediate codes.

Structures are designated according to the conventions in Figure S1. Aromatic cores are denoted by Ph for benzene-derived frameworks and Py for pyridine-derived frameworks. Each skeleton is identified by the format core–number of substituents–position sequence, with the reactive/substituted site listed first. Substituent and nucleophile codes are assigned separately, as shown in Figure S1b. The resulting substrate and Meisenheimer intermediate names are illustrated in Figure S1c by Ph-4-1245-R1 and Ph-4-1245-R1-Nu1, respectively.

Calculation of Conceptual DFT Indices

Global reactivity descriptors were calculated within the framework of conceptual density functional theory (CDFT) to characterize the electronic properties of the substrates and nucleophiles. Following Parr et al. [8], the global electrophilicity index (ω), which measures the stabilization associated with charge acceptance, was calculated as follows:

$$\omega = \frac{\mu^2}{2\eta}$$

Here, μ and η denote the electronic chemical potential and chemical hardness, respectively. Using the finite-difference approximation and the Koopmans-type frontier-molecular-orbital approximation, they were estimated from the HOMO and LUMO energies as follows:

$$\mu \approx \frac{(E_{\mathrm{HOMO}} + E_{\mathrm{LUMO}})}{2}, \quad \eta \approx E_{\mathrm{LUMO}} - E_{\mathrm{HOMO}}$$

The global nucleophilicity index (N) was obtained from the empirical relative scale introduced by Domingo and co-workers [9]:

$$N = E_{\mathrm{HOMO}}(Nu) - E_{\mathrm{HOMO}}(TCE)$$

Here, E_{HOMO}(Nu) is the HOMO energy of the investigated nucleophile. Consistent with the Domingo scale, tetracyanoethylene (TCE) was used as the reference because of its low HOMO energy. Frontier-orbital energies were taken from the corresponding optimized ground-state calculations. The resulting ω and N values are reported in electron volts (eV). At the M06-2X/def2-SVP/PCM(DME) optimized-geometry level used for the primary N analysis, the TCE HOMO was −0.38410 Hartree (−10.452 eV). A M06-2X/def2-TZVPP/PCM(DME) single-point calculation on the same geometry gave −0.38701 Hartree (−10.531 eV), providing a same-geometry cross-check; this single-point value was not substituted for the optimized-geometry reference in the primary N values.

Overview of ΔG vs N Relationships

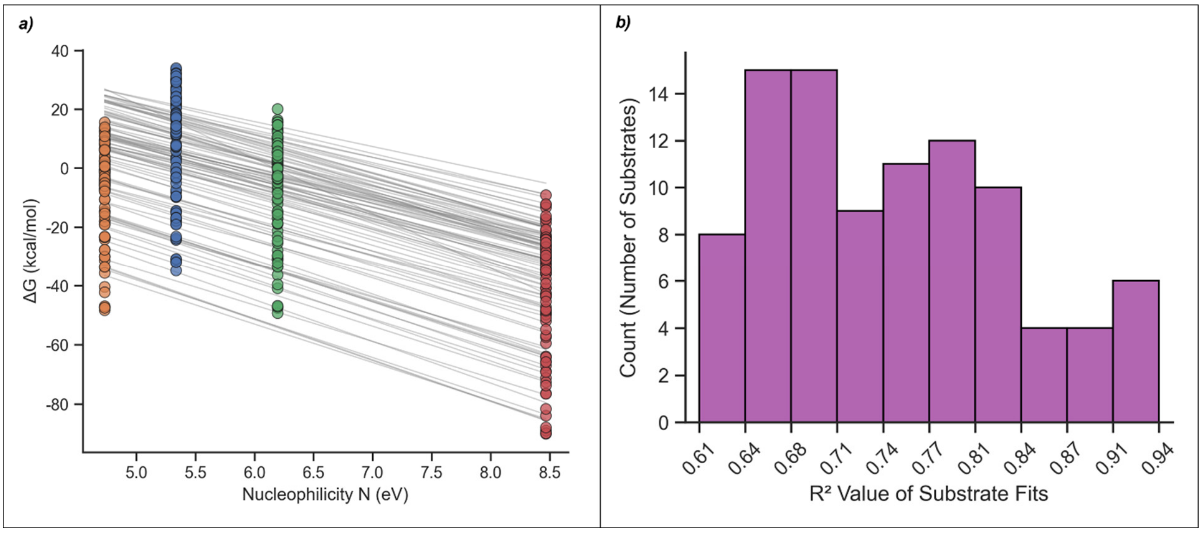

Figure S2. (a) Linear fits of ΔG_{rxn} against N for all fixed substrate/reactive-site cases. (b) Distribution of the corresponding R^2 values.

Figure 3b of the main text shows six representative ΔG_{rxn}–N relationships. One fixed-skeleton series was selected from each substitution-number class of the benzene (Ph) and pyridine (Py) scaffolds, using the four-point fit whose R^2 value was closest to the median for that class. Figure S2a presents the complete set of ΔG_{rxn}–N fits for the fixed substrate/reactive-site cases, and Figure S2b summarizes the

corresponding R^2 values. Each fit contains four nucleophiles and is interpreted only as a within-dataset comparison.

IRC Connectivity Analysis

Bidirectional intrinsic reaction coordinate (IRC) calculations were initiated from all 44 located transition states at the M06-2X/def2-SVP level with PCM(DME), using the same electronic-structure and solvation settings as the transition-state optimizations. Each trajectory was inspected for motion toward the separated substrate–nucleophile pair and the C–C-bonded Meisenheimer intermediate. When an IRC calculation returned too few accepted points, or stopped before both endpoints could be assigned reliably, the transition-state geometry was displaced in both directions along its imaginary-frequency eigenvector and each displaced structure was optimized at the same level. A maximum Cartesian displacement of 0.30 Å was used for the endpoint optimizations reported here. Because the sign of a normal-mode eigenvector is arbitrary, reactant- and product-side assignments were made from the optimized structures.

Figures S3 and S4 show representative extended IRC profiles for two Nu4 transition states. Figure S5 shows complementary endpoint optimizations for representative Nu1, Nu2, and Nu3 cases. One Nu1 trajectory extended over a relatively long segment but did not terminate at an assignable endpoint; it was treated with the same manual-displacement endpoint check. For these three examples, the +d030 displacement relaxed to the separated reactant-side arrangement and the −d030 displacement to the corresponding σ-complex. Manual displacement supplies endpoint evidence when an IRC path is too short or incomplete; it is not equivalent to a fully converged IRC trajectory and was not used as an additional energetic screening criterion.

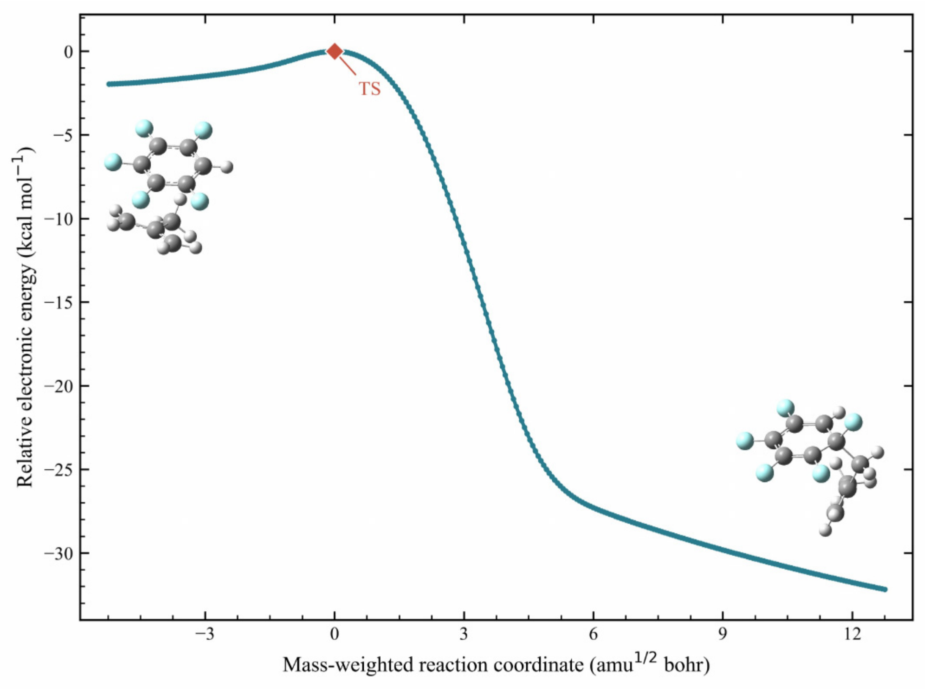

Figure S3. Representative bidirectional IRC profile for TS-Ph-5-12345-R4-Nu4, calculated at M06-2X/def2-SVP with PCM(DME). The forward direction reaches the reactant side (82 accepted points), and the reverse direction reaches the Meisenheimer intermediate (200 accepted points); both calculations terminated normally. Electronic energies are reported relative to the transition state.

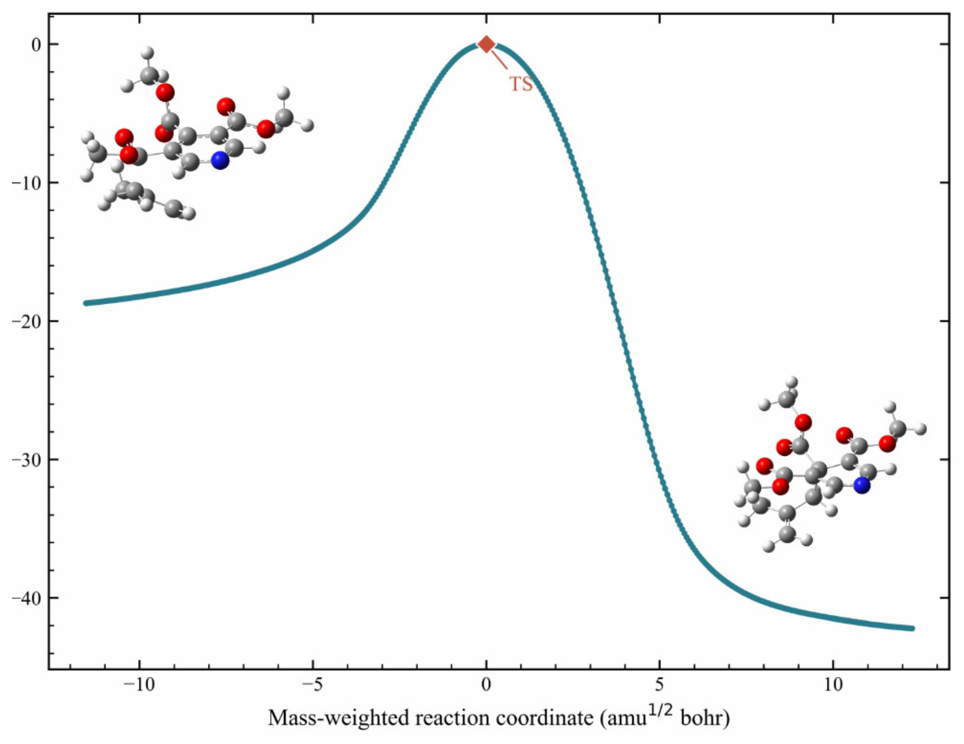

Figure S4. Representative bidirectional IRC profile for TS-Py-3-435-R3-Nu4, calculated at M06-2X/def2-SVP with PCM(DME). The forward trajectory extends toward the reactant side for 188 accepted points before stopping during the next point, whereas the reverse trajectory reaches the Meisenheimer intermediate with 200 accepted points and normal termination. Electronic energies are reported relative to the transition state.

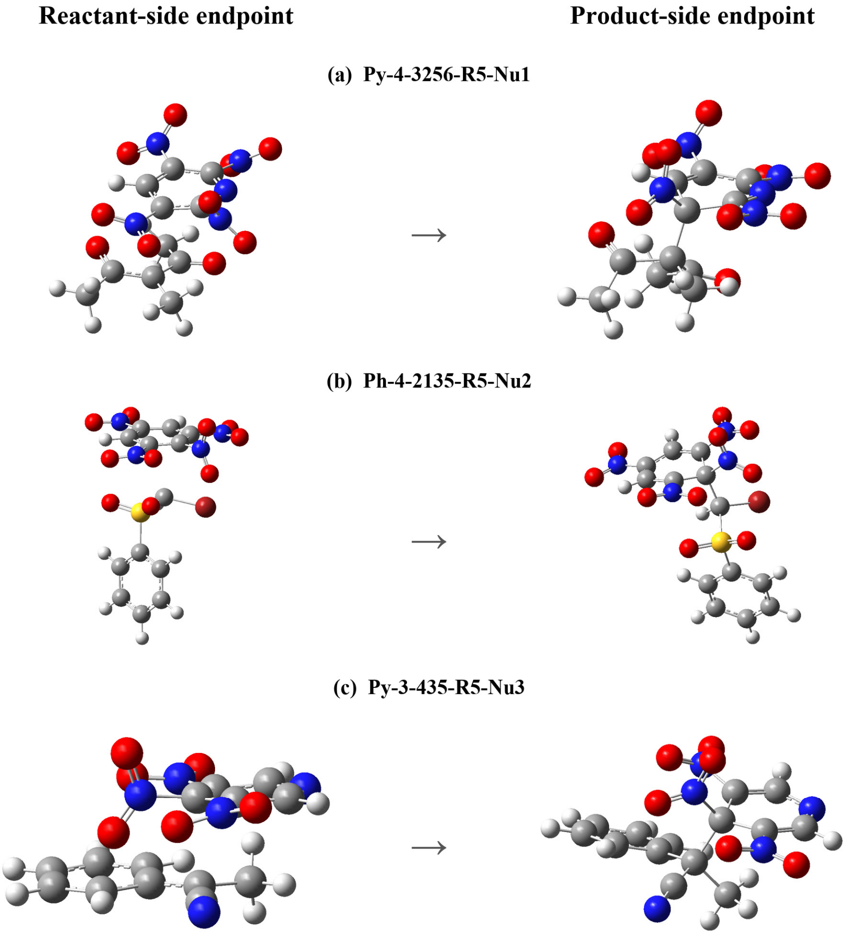

Figure S5. Representative reactant- and product-side structures obtained by manual displacement and endpoint optimization for Nu1–Nu3 transition states. The transition-state geometries were displaced by a maximum Cartesian amplitude of 0.30 Å in both directions along the imaginary-frequency eigenvector and optimized at M06-2X/def2-SVP with PCM(DME). Panels (a)–(c) show Py-4-3256-R5-Nu1, Ph-4-2135-R5-Nu2, and Py-3-435-R5-Nu3, respectively.

Unlocated-Transition-State Candidates

The −40 to −20 kcal/mol thermodynamic window contains 84 combinations. The intended nucleophilic-addition transition state was located for 44 combinations and was not located for 40 with the search protocol used here, corresponding to a success rate of 52% (44/84). The located group contains 36 Nu4, 6 Nu2, 1 Nu3, and 1 Nu1 cases; the unlocated group contains 11 Nu4, 8 Nu2, 14 Nu3, and 7 Nu1 cases. Benzene/pyridine counts are similar in the two groups (23/21 located and 21/19 unlocated), whereas nucleophile composition differs markedly. Median ΔG_{rxn} is −27.8 kcal/mol for the located group and −31.2 kcal/mol for the unlocated group. Thus, the kinetic subset is affected by convergence and nucleophile class and is not representative of the full window. No barrier is assigned to an unlocated case, so these data cannot determine whether its true barrier is higher. The 40 unlocated cases were excluded from the barrier distribution and barrier–reaction-energy analysis.

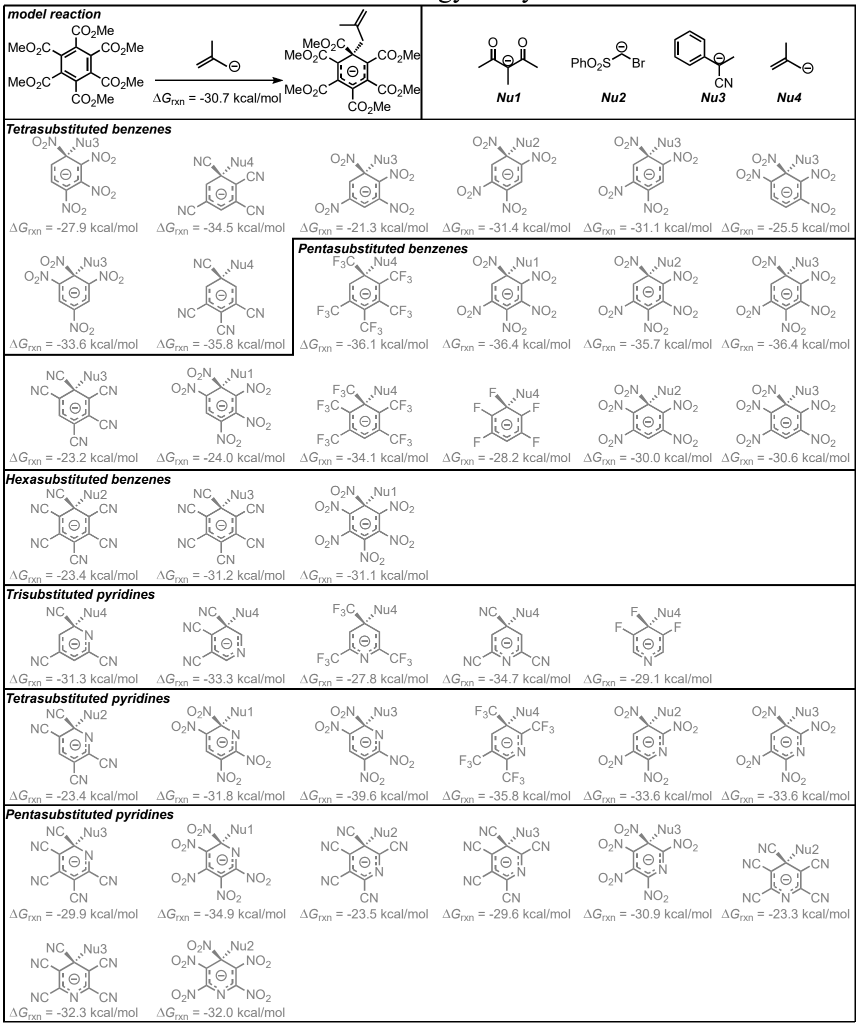

Figure S6. The 40 substrate–nucleophile combinations within the −40 to −20 kcal/mol thermodynamic window for which a nucleophilic-addition transition state was not located with the employed search protocol. Together with the 44 located cases, these structures account for all 84 combinations in the window.

Hammett Substituent-Constant Analysis

Representative fixed-skeleton series were correlated with literature Hammett substituent constants to examine positional substituent effects independently of the global CDFT descriptors. The σ_m, σ_p, and field/inductive constant σ_I values were taken from the compilation of Hansch, Leo, and Taft [12]. For CF_3, CN, CO_2Me, F, and NO_2, the adopted values were 0.43, 0.56, 0.37, 0.34, and 0.71 for σ_m; 0.54, 0.66, 0.45, 0.06, and 0.78 for σ_p; and 0.42, 0.53, 0.34, 0.52, and 0.76 for σ_I, respectively. Meta and para contributions relative to the reactive site were summed to give $\Sigma\sigma_{m,p}$. Classical Hammett constants were developed for meta- and para-substituted aromatic systems and do not explicitly resolve short-range ortho effects [10–12]. The ortho positions were therefore represented by the following field/inductive extension, motivated by the Taft–Charton separation of polar and proximity effects [13–15]:

$$\Sigma\sigma_{\mathrm{H}} = \Sigma\sigma_{\mathrm{m,p}} + n_{\mathrm{o}}\sigma_{\mathrm{I}} \qquad \text{(S1)}$$

In Equation (S1), n_o is the number of symmetry-equivalent ortho positions occupied by the varied substituent in the fixed skeleton and σI is the field/inductive constant of the varied substituent. $\Sigma\sigma_H$ captures an electronic contribution associated with ortho substitution; it does not explicitly parameterize steric crowding, conformational changes, or local solvation. The pyridine ring nitrogen was treated as part of the parent scaffold and was not assigned an additional Hammett constant.

Each fixed-skeleton regression contains five substituent variants and is interpreted only within the corresponding series. Incorporation of the ortho field/inductive term increased R^2 in all 20 ortho-containing ΔG_{rxn} regressions, with ΔR^2 = 0.019–0.144. The corresponding $\Sigma\sigma_H$ fits gave R^2 = 0.917–0.996 for the ortho-containing benzene series and R^2 = 0.931–0.998 for the ortho-containing pyridine series. Thus, the added field/inductive term strengthens the fixed-skeleton reaction-free-energy correlations for the present ΔG_{rxn} dataset. In contrast, the same extension lowered R^2 by 0.031–0.153 in the ωB97X-D substrate-electrophilicity regressions. $\Sigma\sigma_H$ is therefore retained as an ortho-sensitive descriptor for the present ΔG_{rxn} series, not as a property-independent correction.

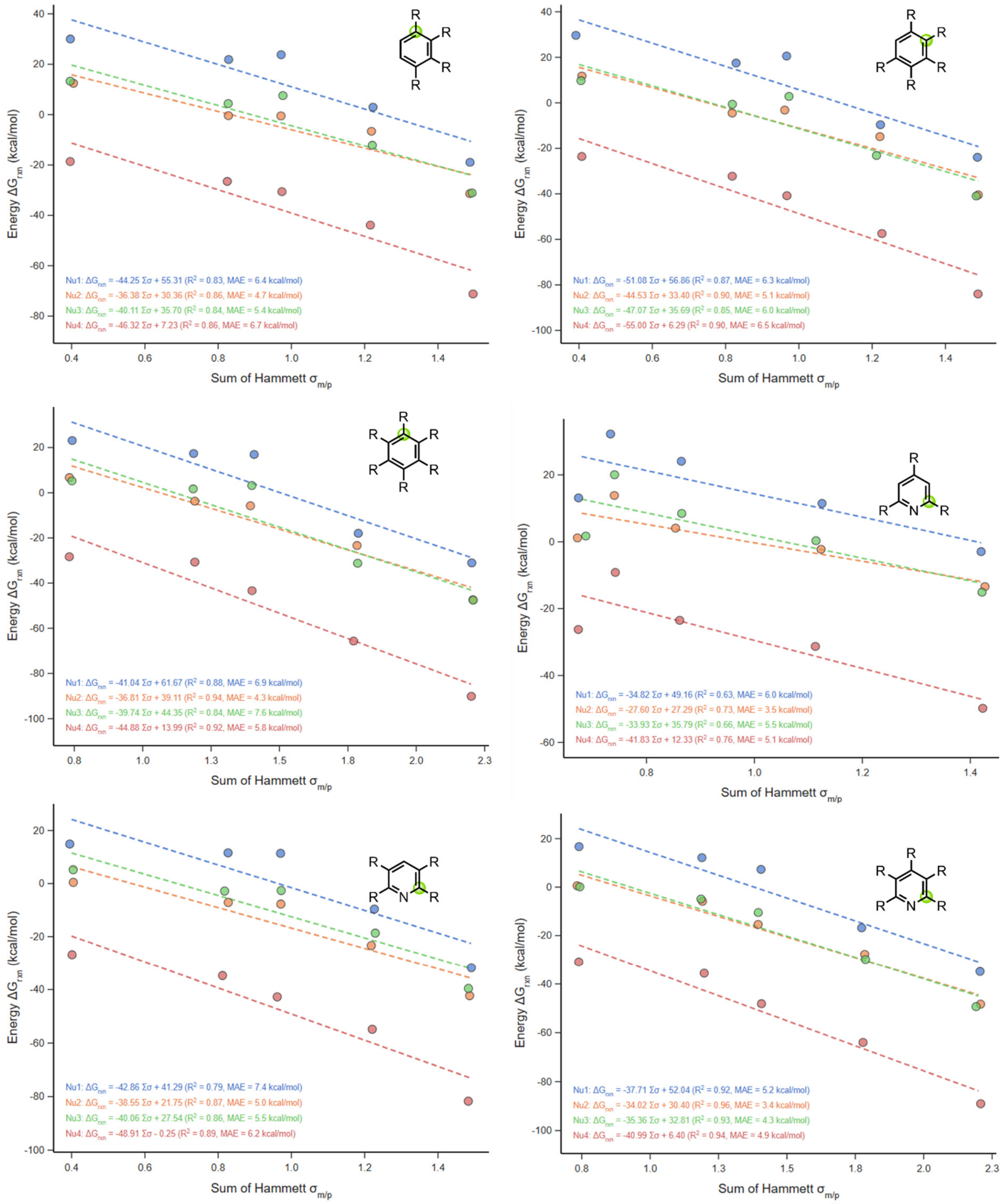

Figure S7. Fixed-skeleton relationships between ΔG_{rxn} and the classical meta/para descriptor $\Sigma\sigma_{\mathrm{m,p}}$ for the six representative benzene and pyridine skeletons. Each line comprises five substituent variants for one nucleophile. Ortho substituents are omitted from $\Sigma\sigma_{\mathrm{m,p}}$.

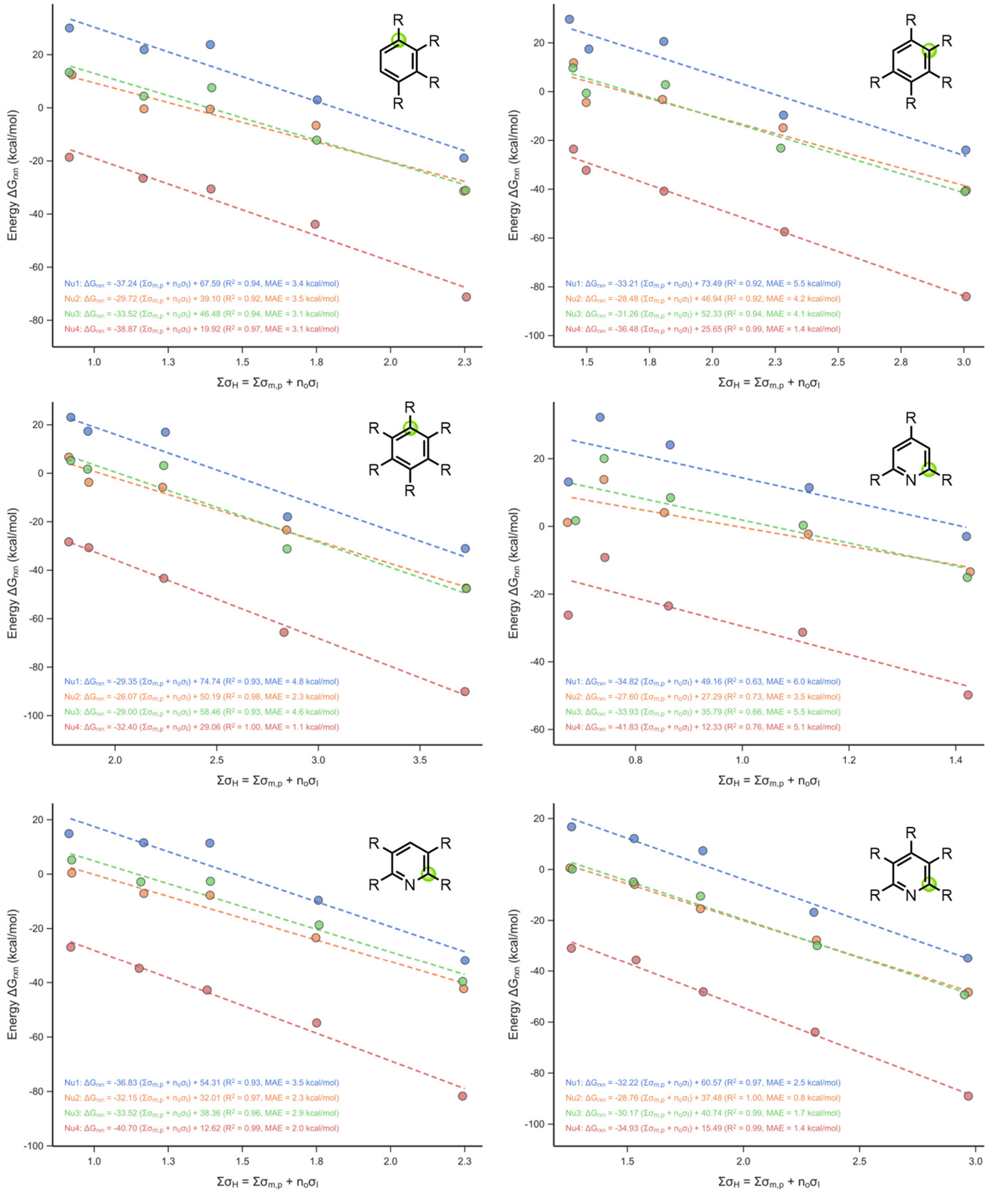

Figure S8. Fixed-skeleton relationships obtained with the ortho-sensitive descriptor $\Sigma\sigma_H = \Sigma\sigma_{m,p} + n_o\sigma_I$. Each panel reports the fitted equation, R^2, and mean absolute error. Py-3-246 contains no varied ortho position; its classical and extended fits are therefore identical.

ωB97X-D CDFT Method-Sensitivity Analysis

The choice of ωB97X-D as the sensitivity method is consistent with the benchmark reported by Błaziak et al. for carbanion addition to substituted nitroarenes [7]. For the para-substituted series in DMF, correlations with experimental relative rates gave R^2 = 0.91 for $\Delta G^‡$ and R^2 = 0.95 for ΔG_R with M06-2X. The corresponding ωB97X-D values were 0.90 and 0.94, while PBE1PBE gave 0.91 and 0.96. M06-2X and ωB97X-D therefore recover closely comparable relative trends in this related nucleophilic-aromatic-addition series, although the same study shows that absolute energies remain method dependent. Here, the comparison is used to test the robustness of the CDFT descriptor relationships, not to define a second barrier model or an absolute rate scale.

ωB97X-D/def2-TZVPP single-point calculations with PCM(DME) were performed for the 95 substrates, four nucleophiles, and the TCE reference on the M06-2X/def2-SVP-PCM(DME) optimized geometries. Frontier-orbital energies were evaluated with the same Koopmans-type definitions used in the primary analysis; the ωB97X-D TCE reference HOMO energy was −11.098 eV. Because CDFT indices depend on the orbital-energy protocol, their absolute values should not be transferred directly between electronic-structure methods. Across the 95 substrates, the M06-2X and ωB97X-D electrophilicity values are strongly correlated (R^2 = 0.995; Spearman ρ = 0.993; Figure S9). The four global ΔG_{rxn}–ω fits give R^2 = 0.690–0.702 with M06-2X and 0.697–0.713 with ωB97X-D (Figure S10), showing that the substrate-electrophilicity relationship is retained. By contrast, the 95 fixed-substrate ΔG_{rxn}–N fits, each based on four nucleophiles, give R^2 = 0.618–0.939 (median 0.745) with M06-2X and 0.412–0.789 (median 0.541) with ωB97X-D. These values are summarized in Table S1; no ωB97X-D fit exceeds its M06-2X counterpart. The global electrophilicity trend is thus method-robust, whereas the four-point nucleophilicity relationships are more method sensitive. M06-2X is retained as the internally consistent primary CDFT protocol for the present dataset.

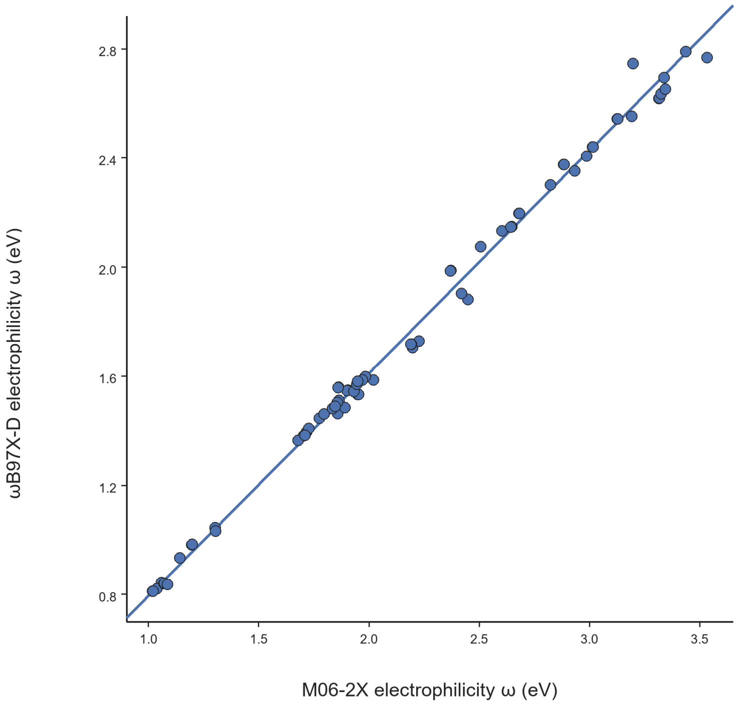

Figure S9. Direct comparison of the M06-2X and ωB97X-D electrophilicity indices for 95 substrates. The coefficients of determination and rank correlation are $R^2 = 0.995$ and Spearman $\rho = 0.993$, respectively, confirming that the substrate electrophilicity ordering is retained across the two functionals.

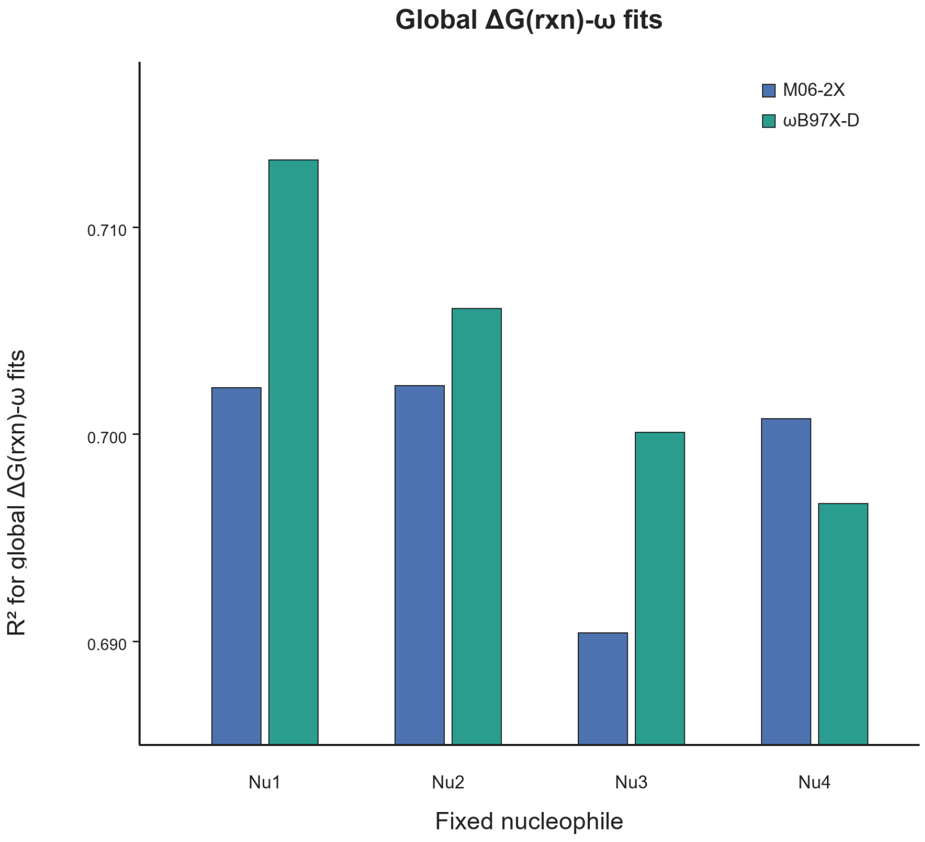

Figure S10. Comparison of the four global ΔG_{rxn}-ω R^2 values obtained with M06-2X and ωB97X-D. Each paired bar represents one fixed-nucleophile regression over 95 substrates; the vertical axis is expanded to resolve the small differences. The closely clustered values confirm that the global substrate-electrophilicity relationship is retained after the functional is changed.

Table S1. Summary of fixed-substrate ΔGrxn–N fit statistics obtained with M06-2X and ωB97X-D.

Analysis	Result
M06-2X, 95 fixed-substrate cases	R^2 = 0.618–0.939; median R^2 = 0.745; four nucleophiles per fit
ωB97X-D, 95 fixed-substrate cases	R^2 = 0.412–0.789; median R^2 = 0.541; four nucleophiles per fit
Pairwise comparison	No ωB97X-D fit exceeds its M06-2X counterpart

References

[1] Gaussian 16, Revision A.03. Frisch, M. J.; Trucks, G. W.; Schlegel, H. B.; Scuseria, G. E.; Robb, M. A.; Cheeseman, J. R.; Scalmani, G.; Barone, V.; Petersson, G. A.; Nakatsuji, H.; Li, X.; Caricato, M.; Marenich, A. V.; Bloino, J.; Janesko, B. G.; Gomperts, R.; Mennucci, B.; Hratchian, H. P.; Ortiz, J. V.; Izmaylov, A. F.; Sonnenberg, J. L.; Williams-Young, D.; Ding, F.; Lipparini, F.; Egidi, F.; Goings, J.; Peng, B.; Petrone, A.; Henderson, T.; Ranasinghe, D.; Zakrzewski, V. G.; Gao, J.; Rega, N.; Zheng, G.; Liang, W.; Hada, M.; Ehara, M.; Toyota, K.; Fukuda, R.; Hasegawa, J.; Ishida, M.; Nakajima, T.; Honda, Y.; Kitao, O.; Nakai, H.; Vreven, T.; Throssell, K.; Montgomery, J. A., Jr.; Peralta, J. E.; Ogliaro, F.; Bearpark, M. J.; Heyd, J. J.; Brothers, E. N.; Kudin, K. N.; Staroverov, V. N.; Keith, T. A.; Kobayashi, R.; Normand, J.; Raghavachari, K.; Rendell, A. P.; Burant, J. C.; Iyengar, S. S.; Tomasi, J.; Cossi, M.; Millam, J. M.; Klene, M.; Adamo, C.; Cammi, R.; Ochterski, J. W.; Martin, R. L.; Morokuma, K.; Farkas, O.; Foresman, J. B.; Fox, D. J. Gaussian, Inc., Wallingford, CT, USA, 2016.

[2] Zhao, Y.; Truhlar, D. G. The M06 suite of density functionals for main group thermochemistry, thermochemical kinetics, noncovalent interactions, excited states, and transition elements: Two new functionals and systematic testing of four M06-class functionals and 12 other functionals. *Theor. Chem. Acc.* 2008, **120**, 215–241.

[3] Weigend, F.; Ahlrichs, R. Balanced basis sets of split valence, triple zeta valence and quadruple zeta valence quality for H to Rn: Design and assessment of accuracy. *Phys. Chem. Chem. Phys.* 2005, **7**, 3297–3305.

[4] Weigend, F. Accurate Coulomb-fitting basis sets for H to Rn. *Phys. Chem. Chem. Phys.* 2006, **8**, 1057–1065.

[5] Cancés, E.; Mennucci, B.; Tomasi, J. A new integral equation formalism for the polarizable continuum model: Theoretical background and applications to isotropic and anisotropic dielectrics. *J. Chem. Phys.* 1997, **107**, 3032–3041.

[6] Scalmani, G.; Frisch, M. J. Continuous surface charge polarizable continuum models of solvation. I. General formalism. *J. Chem. Phys.* 2010, **132**, 114110.

[7] Błaziak, K.; Świder, P.; Mąkosza, M.; Danikiewicz, W. Relative rates of addition of carbanions to substituted nitroarenes: Can quantum chemical calculations give meaningful predictions? *Phys. Chem. Chem. Phys.* 2025, **27**, 5921–5931. https://doi.org/10.1039/d4cp00464g.

[8] Parr, R. G.; Szentpály, L. v.; Liu, S. Electrophilicity index. *J. Am. Chem. Soc.* 1999, **121**, 1922–1924.

[9] Domingo, L. R.; Chamorro, E.; Pérez, P. Understanding the reactivity of captodative ethylenes in polar cycloaddition reactions: A theoretical study. *J. Org. Chem.* 2008, **73**, 4615–4624.

[10] Hammett, L. P. The effect of structure upon the reactions of organic compounds. Benzene derivatives. *J. Am. Chem. Soc.* 1937, **59**, 96–103. https://doi.org/10.1021/ja01280a022.

[11] Jaffé, H. H. A reëxamination of the Hammett equation. *Chem. Rev.* 1953, **53**, 191–261. https://doi.org/10.1021/cr60165a003.

[12] Hansch, C.; Leo, A.; Taft, R. W. A survey of Hammett substituent constants and resonance and field parameters. *Chem. Rev.* 1991, **91**, 165–195. https://doi.org/10.1021/cr00002a004.

[13] Taft, R. W. Linear free energy relationships from rates of esterification and hydrolysis of aliphatic and ortho-substituted benzoate esters. *J. Am. Chem. Soc.* 1952, **74**, 2729–2732. https://doi.org/10.1021/ja01131a010.

[14] Taft, R. W. Polar and steric substituent constants for aliphatic and o-benzoate groups from rates of esterification and hydrolysis of esters. *J. Am. Chem. Soc.* 1952, **74**, 3120–3128. https://doi.org/10.1021/ja01132a049.

[15] Charton, M. Nature of the ortho effect. V. Ortho-substituent constants. *J. Am. Chem. Soc.* 1969, **91**, 6649–6654. https://doi.org/10.1021/ja01052a020.

Table of Energies

Table S2. Uncorrected Gaussian thermochemical values used to derive the energies in Figures 2–6, Figure S2, and Figures S7–S8: zero-point energy (ZPE), thermal correction to enthalpy (TCH), thermal correction to Gibbs free energy (TCG), electronic energy (E), enthalpy (H), and Gibbs free energy (G), all in Hartree, calculated at M06-2X/def2-TZVPP-PCM(DME)//M06-2X/def2-SVP-PCM(DME). The tabulated *G* values do not include the 1 mol/L standard-state conversion; −1.89 kcal/mol must be applied to each derived bimolecular reaction or activation free energy. ZPE, TCH, and TCG were obtained from M06-2X/def2-SVP/PCM(DME) frequency calculations, whereas E values were obtained from M06-2X/def2-TZVPP/PCM(DME) single-point calculations. Blank imaginary-frequency entries denote minima with no imaginary frequency.

Structure	*ZPE*	*TCH*	*TCG*	*E*	*H*	*G*	Imaginary frequency (cm^{-1})
**Nu1**	0.137835	0.147956	0.103920	-384.609141	-384.461185	-384.505221	
**Nu3**	0.142798	0.152464	0.109056	-402.593105	-402.440641	-402.484049	
**Nu2**	0.116679	0.127795	0.078658	-3393.246831	-3393.119036	-3393.168173	
**Nu4**	0.090688	0.097470	0.062894	-156.642571	-156.545101	-156.579677	
**Ph-4-1234-R1**	0.123506	0.141933	0.078229	-1580.560741	-1580.418808	-1580.482512	
**Ph-4-1234-R1-Nu1**	0.264048	0.291810	0.209170	-1965.152056	-1964.860246	-1964.942886	
**Ph-4-1234-R1-Nu3**	0.269232	0.296657	0.214463	-1983.161107	-1982.864450	-1982.946644	
**Ph-4-1234-R1-Nu2**	0.242289	0.271957	0.180826	-4973.824282	-4973.552325	-4973.643456	
**Ph-4-1234-R1-Nu4**	0.221608	0.245531	0.169369	-1737.271956	-1737.026425	-1737.102587	
**Ph-4-1234-R2**	0.096352	0.108789	0.058841	-601.209166	-601.100377	-601.150325	
**Ph-4-1234-R2-Nu1**	0.236753	0.258977	0.187600	-985.834359	-985.575382	-985.646759	
**Ph-4-1234-R2-Nu3**	0.242179	0.263990	0.192722	-1003.841311	-1003.577321	-1003.648589	
**Ph-4-1234-R2-Nu2**	0.215955	0.239178	0.163339	-3994.488047	-3994.248869	-3994.324708	
**Ph-4-1234-R2-Nu4**	0.195091	0.212908	0.150137	-757.945040	-757.732132	-757.794903	
**Ph-4-1234-R3**	0.275183	0.298427	0.222049	-1143.773318	-1143.474891	-1143.551269	
**Ph-4-1234-R3-Nu1**	0.416548	0.448606	0.355979	-1528.374911	-1527.926305	-1528.018932	
**Ph-4-1234-R3-Nu3**	0.421704	0.453338	0.361657	-1546.383247	-1545.929909	-1546.021590	
**Ph-4-1234-R3-Nu2**	0.394916	0.428652	0.329206	-4537.042463	-4536.613811	-4536.713257	
**Ph-4-1234-R3-Nu4**	0.373878	0.401894	0.317327	-1300.483093	-1300.081199	-1300.165766	
**Ph-4-1234-R4**	0.069142	0.077759	0.037204	-629.218886	-629.141127	-629.181682	
**Ph-4-1234-R4-Nu1**	0.208414	0.227295	0.163376	-1013.798815	-1013.571520	-1013.635439	
**Ph-4-1234-R4-Nu3**	0.214531	0.232751	0.170133	-1031.811558	-1031.578807	-1031.641425	
**Ph-4-1234-R4-Nu2**	0.187973	0.207850	0.138936	-4022.465805	-4022.257955	-4022.326869	
**Ph-4-1234-R4-Nu4**	0.166658	0.181055	0.127087	-785.913252	-785.732197	-785.786165	
**Ph-4-1234-R5**	0.113674	0.128991	0.070737	-1050.235889	-1050.106898	-1050.165152	
**Ph-4-1234-R5-Nu1**	0.255724	0.280075	0.203999	-1434.894955	-1434.614880	-1434.690956	
**Ph-4-1234-R5-Nu3**	0.260969	0.284893	0.209501	-1452.900217	-1452.615324	-1452.690716	
**Ph-4-1234-R5-Nu2**	0.234465	0.260185	0.178406	-4443.553056	-4443.292871	-4443.374650	
**Ph-4-1234-R5-Nu4**	0.213277	0.233698	0.164073	-1207.015794	-1206.782096	-1206.851721	
**Ph-4-1235-R1**	0.123228	0.142080	0.075317	-1580.571279	-1580.429199	-1580.495962	
**Ph-4-1235-R1-Nu1**	0.263657	0.291828	0.207485	-1965.158908	-1964.867080	-1964.951423	
**Ph-4-1235-R1-Nu3**	0.268768	0.296791	0.211676	-1983.168511	-1982.871720	-1982.956835	
**Ph-4-1235-R1-Nu2**	0.242135	0.272082	0.179956	-4973.830574	-4973.558492	-4973.650618	

Structure	*ZPE*	*TCH*	*TCG*	*E*	*H*	*G*	Imaginary frequency (cm^{-1})
**Ph-4-1235-R1-Nu4**	0.221209	0.245648	0.166332	-1737.278979	-1737.033331	-1737.112647	
**Ph-4-1235-R2**	0.096371	0.108786	0.058180	-601.211011	-601.102225	-601.152831	
**Ph-4-1235-R2-Nu1**	0.236292	0.258604	0.187023	-985.824738	-985.566134	-985.637715	
**Ph-4-1235-R2-Nu3**	0.241665	0.263550	0.192197	-1003.831602	-1003.568052	-1003.639405	
**Ph-4-1235-R2-Nu2**	0.215333	0.238818	0.161902	-3994.480842	-3994.242024	-3994.318940	
**Ph-4-1235-R2-Nu4**	0.194429	0.212371	0.149143	-757.933635	-757.721264	-757.784492	
**Ph-4-1235-R3**	0.275247	0.298481	0.222093	-1143.780238	-1143.481757	-1143.558145	
**Ph-4-1235-R3-Nu1**	0.416088	0.448377	0.354508	-1528.361738	-1527.913361	-1528.007230	
**Ph-4-1235-R3-Nu3**	0.421180	0.453239	0.358874	-1546.372051	-1545.918812	-1546.013177	
**Ph-4-1235-R3-Nu2**	0.394858	0.428660	0.329229	-4537.030795	-4536.602135	-4536.701566	
**Ph-4-1235-R3-Nu4**	0.373830	0.401919	0.316686	-1300.471374	-1300.069455	-1300.154688	
**Ph-4-1235-R4**	0.069046	0.077594	0.037133	-629.224475	-629.146881	-629.187342	
**Ph-4-1235-R4-Nu1**	0.208554	0.227191	0.164130	-1013.815573	-1013.588382	-1013.651443	
**Ph-4-1235-R4-Nu3**	0.214164	0.232398	0.169216	-1031.825524	-1031.593126	-1031.656308	
**Ph-4-1235-R4-Nu2**	0.187905	0.207579	0.139606	-4022.479227	-4022.271648	-4022.339621	
**Ph-4-1235-R4-Nu4**	0.166717	0.180897	0.127263	-785.928301	-785.747404	-785.801038	
**Ph-4-1235-R5**	0.113726	0.129024	0.071019	-1050.247133	-1050.118109	-1050.176114	
**Ph-4-1235-R5-Nu1**	0.255653	0.280114	0.203241	-1434.894713	-1434.614599	-1434.691472	
**Ph-4-1235-R5-Nu3**	0.260591	0.284835	0.207384	-1452.898477	-1452.613642	-1452.691093	
**Ph-4-1235-R5-Nu2**	0.234109	0.260051	0.177114	-4443.548641	-4443.288590	-4443.371527	
**Ph-4-1235-R5-Nu4**	0.212962	0.233598	0.163181	-1207.010772	-1206.777174	-1206.847591	
**Ph-4-1245-R1**	0.123438	0.142306	0.076386	-1580.575900	-1580.433594	-1580.499514	
**Ph-4-1245-R1-Nu1**	0.264215	0.292307	0.208473	-1965.172295	-1964.879988	-1964.963822	
**Ph-4-1245-R1-Nu3**	0.269242	0.297148	0.212916	-1983.181405	-1982.884257	-1982.968489	
**Ph-4-1245-R1-Nu2**	0.242592	0.272430	0.180899	-4973.846379	-4973.573949	-4973.665480	
**Ph-4-1245-R1-Nu4**	0.221771	0.245950	0.168717	-1737.293599	-1737.047649	-1737.124882	
**Ph-4-1245-R2**	0.096335	0.108777	0.059327	-601.211293	-601.102516	-601.151966	
**Ph-4-1245-R2-Nu1**	0.236939	0.259115	0.187838	-985.837276	-985.578161	-985.649438	
**Ph-4-1245-R2-Nu3**	0.242337	0.264071	0.193167	-1003.845588	-1003.581517	-1003.652421	
**Ph-4-1245-R2-Nu2**	0.215941	0.239226	0.163024	-3994.490727	-3994.251501	-3994.327703	
**Ph-4-1245-R2-Nu4**	0.195254	0.213031	0.150252	-757.948786	-757.735755	-757.798534	
**Ph-4-1245-R3**	0.275075	0.298433	0.221293	-1143.774913	-1143.476480	-1143.553620	
**Ph-4-1245-R3-Nu1**	0.415905	0.448398	0.353590	-1528.374546	-1527.926148	-1528.020956	
**Ph-4-1245-R3-Nu3**	0.421509	0.453521	0.359645	-1546.387322	-1545.933801	-1546.027677	
**Ph-4-1245-R3-Nu2**	0.395390	0.429097	0.329323	-4537.048735	-4536.619638	-4536.719412	
**Ph-4-1245-R3-Nu4**	0.374076	0.402250	0.315991	-1300.488562	-1300.086312	-1300.172571	
**Ph-4-1245-R4**	0.069025	0.077572	0.036454	-629.223865	-629.146293	-629.187411	
**Ph-4-1245-R4-Nu1**	0.208407	0.227138	0.163553	-1013.805347	-1013.578209	-1013.641794	
**Ph-4-1245-R4-Nu3**	0.214270	0.232472	0.169781	-1031.817045	-1031.584573	-1031.647264	
**Ph-4-1245-R4-Nu2**	0.187767	0.207572	0.139009	-4022.471774	-4022.264202	-4022.332765	
**Ph-4-1245-R4-Nu4**	0.166375	0.180795	0.126387	-785.920165	-785.739370	-785.793778	
**Ph-4-1245-R5**	0.113724	0.129057	0.070230	-1050.241349	-1050.112292	-1050.171119	
**Ph-4-1245-R5-Nu1**	0.255745	0.280261	0.203219	-1434.906705	-1434.626444	-1434.703486	
**Ph-4-1245-R5-Nu3**	0.261244	0.285227	0.209228	-1452.910933	-1452.625706	-1452.701705	
**Ph-4-1245-R5-Nu2**	0.234512	0.260369	0.177405	-4443.563669	-4443.303300	-4443.386264	
**Ph-4-1245-R5-Nu4**	0.213601	0.234056	0.164209	-1207.025486	-1206.791430	-1206.861277	

Structure	*ZPE*	*TCH*	*TCG*	*E*	*H*	*G*	Imaginary frequency (cm^{-1})
**Ph-4-2134-R1**	0.123338	0.141895	0.077215	-1580.552945	-1580.411050	-1580.475730	
**Ph-4-2134-R1-Nu1**	0.263897	0.291651	0.209151	-1965.139489	-1964.847838	-1964.930338	
**Ph-4-2134-R1-Nu3**	0.269356	0.296721	0.214758	-1983.151977	-1982.855256	-1982.937219	
**Ph-4-2134-R1-Nu2**	0.242627	0.272066	0.183232	-4973.814584	-4973.542518	-4973.631352	
**Ph-4-2134-R1-Nu4**	0.222253	0.245974	0.170936	-1737.270578	-1737.024604	-1737.099642	
**Ph-4-2134-R2**	0.096352	0.108788	0.058187	-601.209166	-601.100378	-601.150979	
**Ph-4-2134-R2-Nu1**	0.236662	0.258836	0.187976	-985.831863	-985.573027	-985.643887	
**Ph-4-2134-R2-Nu3**	0.242209	0.263860	0.193290	-1003.838034	-1003.574174	-1003.644744	
**Ph-4-2134-R2-Nu2**	0.215595	0.238864	0.162837	-3994.480770	-3994.241906	-3994.317933	
**Ph-4-2134-R2-Nu4**	0.195011	0.212834	0.149680	-757.942498	-757.729664	-757.792818	
**Ph-4-2134-R3**	0.275200	0.298399	0.222457	-1143.773804	-1143.475405	-1143.551347	
**Ph-4-2134-R3-Nu1**	0.417094	0.448701	0.357885	-1528.363920	-1527.915219	-1528.006035	
**Ph-4-2134-R3-Nu3**	0.421230	0.453146	0.359815	-1546.375505	-1545.922359	-1546.015690	
**Ph-4-2134-R3-Nu2**	0.395532	0.428895	0.330907	-4537.034186	-4536.605291	-4536.703279	
**Ph-4-2134-R3-Nu4**	0.373794	0.401762	0.316960	-1300.478844	-1300.077082	-1300.161884	
**Ph-4-2134-R4**	0.069134	0.077750	0.036545	-629.218887	-629.141137	-629.182342	
**Ph-4-2134-R4-Nu1**	0.208792	0.227367	0.163995	-1013.804877	-1013.577510	-1013.640882	
**Ph-4-2134-R4-Nu3**	0.214548	0.232637	0.170394	-1031.817945	-1031.585308	-1031.647551	
**Ph-4-2134-R4-Nu2**	0.188056	0.207684	0.139999	-4022.470054	-4022.262370	-4022.330055	
**Ph-4-2134-R4-Nu4**	0.166909	0.181203	0.127206	-785.923507	-785.742304	-785.796301	
**Ph-4-2134-R5**	0.113675	0.128990	0.070745	-1050.235885	-1050.106895	-1050.165140	
**Ph-4-2134-R5-Nu1**	0.256723	0.280399	0.206664	-1434.889743	-1434.609344	-1434.683079	
**Ph-4-2134-R5-Nu3**	0.261175	0.284943	0.209624	-1452.896415	-1452.611472	-1452.686791	
**Ph-4-2134-R5-Nu2**	0.234553	0.260089	0.179246	-4443.548132	-4443.288043	-4443.368886	
**Ph-4-2134-R5-Nu4**	0.213426	0.233721	0.165058	-1207.013746	-1206.780025	-1206.848688	
**Ph-4-2135-R1**	0.123231	0.142098	0.075238	-1580.571251	-1580.429153	-1580.496013	
**Ph-4-2135-R1-Nu1**	0.264273	0.292281	0.207749	-1965.163190	-1964.870909	-1964.955441	
**Ph-4-2135-R1-Nu3**	0.269297	0.297038	0.213057	-1983.174070	-1982.877032	-1982.961013	
**Ph-4-2135-R1-Nu2**	0.242494	0.272376	0.180255	-4973.836031	-4973.563655	-4973.655776	
**Ph-4-2135-R1-Nu4**	0.222085	0.246174	0.168822	-1737.293606	-1737.047432	-1737.124784	
**Ph-4-2135-R2**	0.096372	0.108788	0.058181	-601.211015	-601.102227	-601.152834	
**Ph-4-2135-R2-Nu1**	0.237196	0.259277	0.188377	-985.845091	-985.585814	-985.656714	
**Ph-4-2135-R2-Nu3**	0.242613	0.264275	0.193469	-1003.851437	-1003.587162	-1003.657968	
**Ph-4-2135-R2-Nu2**	0.216056	0.239157	0.163720	-3994.490997	-3994.251840	-3994.327277	
**Ph-4-2135-R2-Nu4**	0.195524	0.213237	0.150505	-757.958251	-757.745014	-757.807746	
**Ph-4-2135-R3**	0.275178	0.298462	0.221298	-1143.781204	-1143.482742	-1143.559906	
**Ph-4-2135-R3-Nu1**	0.417460	0.449132	0.357630	-1528.386091	-1527.936959	-1528.028461	
**Ph-4-2135-R3-Nu3**	0.422157	0.453796	0.361623	-1546.398576	-1545.944780	-1546.036953	
**Ph-4-2135-R3-Nu2**	0.395781	0.429257	0.329673	-4537.056632	-4536.627375	-4536.726959	
**Ph-4-2135-R3-Nu4**	0.374683	0.402612	0.317347	-1300.502448	-1300.099836	-1300.185101	
**Ph-4-2135-R4**	0.069047	0.077596	0.036478	-629.224471	-629.146875	-629.187993	
**Ph-4-2135-R4-Nu1**	0.208671	0.227168	0.164429	-1013.800619	-1013.573451	-1013.636190	
**Ph-4-2135-R4-Nu3**	0.215332	0.233547	0.170285	-1031.850359	-1031.616812	-1031.680074	
**Ph-4-2135-R4-Nu2**	0.187539	0.207344	0.138855	-4022.467286	-4022.259942	-4022.328431	
**Ph-4-2135-R4-Nu4**	0.167106	0.181818	0.125695	-785.959158	-785.777340	-785.833463	
**Ph-4-2135-R5**	0.113721	0.129022	0.070350	-1050.247132	-1050.118110	-1050.176782	

Structure	*ZPE*	*TCH*	*TCG*	*E*	*H*	*G*	Imaginary frequency (cm^{-1})
**Ph-4-2135-R5-Nu1**	0.256733	0.280563	0.205947	-1434.913155	-1434.632592	-1434.707208	
**Ph-4-2135-R5-Nu3**	0.261477	0.285283	0.209377	-1452.920709	-1452.635426	-1452.711332	
**Ph-4-2135-R5-Nu2**	0.234669	0.260287	0.178418	-4443.574186	-4443.313899	-4443.395768	
**Ph-4-2135-R5-Nu4**	0.213911	0.234158	0.165244	-1207.040526	-1206.806368	-1206.875282	
**Ph-4-5123-R1**	0.123227	0.142080	0.075260	-1580.571278	-1580.429198	-1580.496018	
**Ph-4-5123-R1-Nu1**	0.263469	0.292080	0.205402	-1965.165568	-1964.873488	-1964.960166	
**Ph-4-5123-R1-Nu3**	0.268782	0.296714	0.212769	-1983.180597	-1982.883883	-1982.967828	
**Ph-4-5123-R1-Nu2**	0.241935	0.271910	0.179327	-4973.836738	-4973.564828	-4973.657411	
**Ph-4-5123-R1-Nu4**	0.221132	0.245291	0.169353	-1737.283534	-1737.038243	-1737.114181	
**Ph-4-5123-R2**	0.096369	0.108783	0.058178	-601.211007	-601.102224	-601.152829	
**Ph-4-5123-R2-Nu1**	0.236611	0.258933	0.187033	-985.830670	-985.571737	-985.643637	
**Ph-4-5123-R2-Nu3**	0.241887	0.263766	0.192381	-1003.835677	-1003.571911	-1003.643296	
**Ph-4-5123-R2-Nu2**	0.215784	0.239125	0.162898	-3994.489774	-3994.250649	-3994.326876	
**Ph-4-5123-R2-Nu4**	0.194897	0.212764	0.149847	-757.936459	-757.723695	-757.786612	
**Ph-4-5123-R3**	0.275084	0.298445	0.221447	-1143.779301	-1143.480856	-1143.557854	
**Ph-4-5123-R3-Nu1**	0.415618	0.448248	0.352635	-1528.365766	-1527.917518	-1528.013131	
**Ph-4-5123-R3-Nu3**	0.420636	0.453081	0.357482	-1546.371493	-1545.918412	-1546.014011	
**Ph-4-5123-R3-Nu2**	0.394719	0.428733	0.327767	-4537.029820	-4536.601087	-4536.702053	
**Ph-4-5123-R3-Nu4**	0.373291	0.401826	0.313149	-1300.468964	-1300.067138	-1300.155815	
**Ph-4-5123-R4**	0.069046	0.077594	0.036478	-629.224477	-629.146883	-629.187999	
**Ph-4-5123-R4-Nu1**	0.208419	0.227269	0.163278	-1013.807595	-1013.580326	-1013.644317	
**Ph-4-5123-R4-Nu3**	0.214059	0.232334	0.169152	-1031.818947	-1031.586613	-1031.649795	
**Ph-4-5123-R4-Nu2**	0.187881	0.207760	0.138622	-4022.476261	-4022.268501	-4022.337639	
**Ph-4-5123-R4-Nu4**	0.166462	0.180827	0.126428	-785.918574	-785.737747	-785.792146	
**Ph-4-5123-R5**	0.113721	0.129022	0.070350	-1050.247132	-1050.118110	-1050.176782	
**Ph-4-5123-R5-Nu1**	0.255256	0.280142	0.201146	-1434.893336	-1434.613194	-1434.692190	
**Ph-4-5123-R5-Nu3**	0.260721	0.284990	0.208341	-1452.897024	-1452.612034	-1452.688683	
**Ph-4-5123-R5-Nu2**	0.234349	0.260421	0.176204	-4443.550288	-4443.289867	-4443.374084	
**Ph-4-5123-R5-Nu4**	0.213475	0.233926	0.164419	-1207.003804	-1206.769878	-1206.839385	
**Ph-5-12345-R1**	0.128656	0.150504	0.079345	-1917.634508	-1917.484004	-1917.555163	
**Ph-5-12345-R1-Nu1**	0.269600	0.300785	0.210886	-2302.242185	-2301.941400	-2302.031299	
**Ph-5-12345-R1-Nu3**	0.274977	0.305733	0.216469	-2320.250412	-2319.944679	-2320.033943	
**Ph-5-12345-R1-Nu2**	0.248027	0.280972	0.183582	-5310.911962	-5310.630990	-5310.728380	
**Ph-5-12345-R1-Nu4**	0.227260	0.254415	0.172161	-2074.361451	-2074.107036	-2074.189290	
**Ph-5-12345-R2**	0.094827	0.109180	0.053931	-693.443921	-693.334741	-693.389990	
**Ph-5-12345-R2-Nu1**	0.235836	0.259883	0.184265	-1078.088253	-1077.828370	-1077.903988	
**Ph-5-12345-R2-Nu3**	0.241147	0.264833	0.188940	-1096.090177	-1095.825344	-1095.901237	
**Ph-5-12345-R2-Nu2**	0.214678	0.239841	0.159453	-4086.737871	-4086.498030	-4086.578418	
**Ph-5-12345-R2-Nu4**	0.193926	0.213640	0.146274	-850.195817	-849.982177	-850.049543	
**Ph-5-12345-R3**	0.318145	0.346090	0.258206	-1371.650061	-1371.303971	-1371.391855	
**Ph-5-12345-R3-Nu1**	0.459605	0.496434	0.392251	-1756.257172	-1755.760738	-1755.864921	
**Ph-5-12345-R3-Nu3**	0.465156	0.501488	0.398232	-1774.269314	-1773.767826	-1773.871082	
**Ph-5-12345-R3-Nu2**	0.438856	0.476858	0.368612	-4764.928203	-4764.451345	-4764.559591	
**Ph-5-12345-R3-Nu4**	0.417200	0.449907	0.353987	-1528.369839	-1527.919932	-1528.015852	
**Ph-5-12345-R4**	0.060950	0.070513	0.027118	-728.461253	-728.390740	-728.434135	
**Ph-5-12345-R4-Nu1**	0.200558	0.220367	0.154453	-1113.050439	-1112.830072	-1112.895986	

Structure	*ZPE*	*TCH*	*TCG*	*E*	*H*	*G*	Imaginary frequency (cm^{-1})
**Ph-5-12345-R4-Nu3**	0.206410	0.225659	0.160422	-1131.061092	-1130.835433	-1130.900670	
**Ph-5-12345-R4-Nu2**	0.179873	0.200713	0.129667	-4121.713901	-4121.513188	-4121.584234	
**Ph-5-12345-R4-Nu4**	0.158653	0.174098	0.116389	-885.163343	-884.989245	-885.046954	
**Ph-5-12345-R5**	0.116150	0.134178	0.069478	-1254.723673	-1254.589495	-1254.654195	
**Ph-5-12345-R5-Nu1**	0.259037	0.285951	0.204054	-1639.399323	-1639.113372	-1639.195269	
**Ph-5-12345-R5-Nu3**	0.264320	0.290762	0.209939	-1657.403141	-1657.112379	-1657.193202	
**Ph-5-12345-R5-Nu2**	0.237717	0.266048	0.177696	-4648.053950	-4647.787902	-4647.876254	
**Ph-5-12345-R5-Nu4**	0.216670	0.239662	0.164002	-1411.516929	-1411.277267	-1411.352927	
**Ph-5-21345-R1**	0.128589	0.150522	0.078676	-1917.627384	-1917.476862	-1917.548708	
**Ph-5-21345-R1-Nu1**	0.269892	0.300762	0.212205	-2302.230382	-2301.929620	-2302.018177	
**Ph-5-21345-R1-Nu3**	0.275164	0.305747	0.216858	-2320.242100	-2319.936353	-2320.025242	
**Ph-5-21345-R1-Nu2**	0.248350	0.281050	0.185203	-5310.904195	-5310.623145	-5310.718992	
**Ph-5-21345-R1-Nu4**	0.227917	0.254973	0.172643	-2074.363031	-2074.108058	-2074.190388	
**Ph-5-21345-R2**	0.094828	0.109182	0.053932	-693.443924	-693.334742	-693.389992	
**Ph-5-21345-R2-Nu1**	0.235752	0.259817	0.184190	-1078.091772	-1077.831955	-1077.907582	
**Ph-5-21345-R2-Nu3**	0.241239	0.264839	0.189314	-1096.097286	-1095.832447	-1095.907972	
**Ph-5-21345-R2-Nu2**	0.214540	0.239648	0.159476	-4086.738267	-4086.498619	-4086.578791	
**Ph-5-21345-R2-Nu4**	0.194190	0.213820	0.146401	-850.204594	-849.990774	-850.058193	
**Ph-5-21345-R3**	0.318395	0.346206	0.259483	-1371.657915	-1371.311709	-1371.398432	
**Ph-5-21345-R3-Nu1**	0.460424	0.496684	0.394702	-1756.267570	-1755.770886	-1755.872868	
**Ph-5-21345-R3-Nu3**	0.465355	0.501549	0.399122	-1774.279596	-1773.778047	-1773.880474	
**Ph-5-21345-R3-Nu2**	0.438923	0.476977	0.367947	-4764.938667	-4764.461690	-4764.570720	
**Ph-5-21345-R3-Nu4**	0.418112	0.450401	0.356376	-1528.382859	-1527.932458	-1528.026483	
**Ph-5-21345-R4**	0.060952	0.070515	0.027118	-728.461248	-728.390733	-728.434130	
**Ph-5-21345-R4-Nu1**	0.201219	0.220580	0.156150	-1113.045247	-1112.824667	-1112.889097	
**Ph-5-21345-R4-Nu3**	0.206335	0.225597	0.160521	-1131.060203	-1130.834606	-1130.899682	
**Ph-5-21345-R4-Nu2**	0.180026	0.200720	0.130314	-4121.710845	-4121.510125	-4121.580531	
**Ph-5-21345-R4-Nu4**	0.158890	0.174272	0.118095	-885.166433	-884.992161	-885.048338	
**Ph-5-21345-R5**	0.116151	0.134179	0.069476	-1254.723672	-1254.589493	-1254.654196	
**Ph-5-21345-R5-Nu1**	0.259453	0.285889	0.204965	-1639.399551	-1639.113662	-1639.194586	
**Ph-5-21345-R5-Nu3**	0.264621	0.290808	0.210433	-1657.410890	-1657.120082	-1657.200457	
**Ph-5-21345-R5-Nu2**	0.237828	0.265922	0.178913	-4648.062742	-4647.796820	-4647.883829	
**Ph-5-21345-R5-Nu4**	0.216896	0.239672	0.165020	-1411.529725	-1411.290053	-1411.364705	
**Ph-5-31245-R1**	0.128589	0.150522	0.078677	-1917.627383	-1917.476861	-1917.548706	
**Ph-5-31245-R1-Nu1**	0.269708	0.300575	0.212407	-2302.221007	-2301.920432	-2302.008600	
**Ph-5-31245-R1-Nu3**	0.274979	0.305511	0.217425	-2320.232395	-2319.926884	-2320.014970	
**Ph-5-31245-R1-Nu2**	0.248154	0.280987	0.184818	-5310.896444	-5310.615457	-5310.711626	
**Ph-5-31245-R1-Nu4**	0.227891	0.254936	0.172839	-2074.352488	-2074.097552	-2074.179649	
**Ph-5-31245-R2**	0.094827	0.109181	0.053931	-693.443922	-693.334741	-693.389991	
**Ph-5-31245-R2-Nu1**	0.235263	0.259403	0.183617	-1078.081625	-1077.822222	-1077.898008	
**Ph-5-31245-R2-Nu3**	0.240568	0.264249	0.188799	-1096.087070	-1095.822821	-1095.898271	
**Ph-5-31245-R2-Nu2**	0.214257	0.239487	0.158870	-4086.731094	-4086.491607	-4086.572224	
**Ph-5-31245-R2-Nu4**	0.193800	0.213437	0.146348	-850.192136	-849.978699	-850.045788	
**Ph-5-31245-R3**	0.318229	0.346226	0.258483	-1371.657517	-1371.311291	-1371.399034	
**Ph-5-31245-R3-Nu1**	0.459999	0.496428	0.393934	-1756.247261	-1755.750833	-1755.853327	
**Ph-5-31245-R3-Nu3**	0.464797	0.501107	0.395549	-1774.258581	-1773.757474	-1773.863032	

Structure	*ZPE*	*TCH*	*TCG*	*E*	*H*	*G*	Imaginary frequency (cm^{-1})
**Ph-5-31245-R3-Nu2**	0.438965	0.476826	0.369366	-4764.918967	-4764.442141	-4764.549601	
**Ph-5-31245-R3-Nu4**	0.417545	0.449905	0.355300	-1528.364244	-1527.914339	-1528.008944	
**Ph-5-31245-R4**	0.060948	0.070510	0.027116	-728.461250	-728.390740	-728.434134	
**Ph-5-31245-R4-Nu1**	0.200968	0.220476	0.155410	-1113.055870	-1112.835394	-1112.900460	
**Ph-5-31245-R4-Nu3**	0.206491	0.225614	0.160688	-1131.067507	-1130.841893	-1130.906819	
**Ph-5-31245-R4-Nu2**	0.180060	0.200689	0.130627	-4121.719714	-4121.519025	-4121.589087	
**Ph-5-31245-R4-Nu4**	0.159051	0.174242	0.118148	-885.173857	-884.999615	-885.055709	
**Ph-5-31245-R5**	0.116151	0.134179	0.069476	-1254.723672	-1254.589493	-1254.654196	
**Ph-5-31245-R5-Nu1**	0.259212	0.285739	0.204803	-1639.384340	-1639.098601	-1639.179537	
**Ph-5-31245-R5-Nu3**	0.264038	0.290434	0.209143	-1657.393123	-1657.102689	-1657.183980	
**Ph-5-31245-R5-Nu2**	0.237409	0.265685	0.178036	-4648.045185	-4647.779500	-4647.867149	
**Ph-5-31245-R5-Nu4**	0.216121	0.239116	0.163827	-1411.510503	-1411.271387	-1411.346676	
**Ph-6-123456-R1**	0.134039	0.158723	0.084443	-2254.689492	-2254.530769	-2254.605049	
**Ph-6-123456-R1-Nu1**	0.275109	0.309187	0.214466	-2639.294719	-2638.985532	-2639.080253	
**Ph-6-123456-R1-Nu3**	0.282339	0.315181	0.224113	-2657.305159	-2656.989978	-2657.081046	
**Ph-6-123456-R1-Nu2**	0.253970	0.289533	0.188909	-5647.968337	-5647.678804	-5647.779428	
**Ph-6-123456-R1-Nu4**	0.233165	0.263266	0.174875	-2411.425691	-2411.162425	-2411.250816	
**Ph-6-123456-R2**	0.093257	0.109546	0.049758	-785.673814	-785.564268	-785.624056	
**Ph-6-123456-R2-Nu1**	0.234402	0.260327	0.180673	-1170.335565	-1170.075238	-1170.154892	
**Ph-6-123456-R2-Nu3**	0.239849	0.265300	0.186111	-1188.340957	-1188.075657	-1188.154846	
**Ph-6-123456-R2-Nu2**	0.213353	0.240355	0.156064	-4178.982534	-4178.742179	-4178.826470	
**Ph-6-123456-R2-Nu4**	0.192908	0.214417	0.142797	-942.448121	-942.233704	-942.305324	
**Ph-6-123456-R3**	0.361163	0.393984	0.296119	-1599.536809	-1599.142825	-1599.240690	
**Ph-6-123456-R3-Nu1**	0.503501	0.544319	0.432710	-1984.147989	-1983.603670	-1983.715279	
**Ph-6-123456-R3-Nu3**	0.508735	0.549240	0.437626	-2002.156656	-2001.607416	-2001.719030	
**Ph-6-123456-R3-Nu2**	0.482262	0.524763	0.406891	-4992.818682	-4992.293919	-4992.411791	
**Ph-6-123456-R3-Nu4**	0.461418	0.498165	0.394404	-1756.260676	-1755.762511	-1755.866272	
**Ph-6-123456-R4**	0.052451	0.063250	0.019425	-827.696679	-827.633429	-827.677254	
**Ph-6-123456-R4-Nu1**	0.193404	0.213877	0.146760	-1212.289412	-1212.075535	-1212.142652	
**Ph-6-123456-R4-Nu3**	0.198619	0.218803	0.151750	-1230.301724	-1230.082921	-1230.149974	
**Ph-6-123456-R4-Nu2**	0.172258	0.193987	0.121387	-4220.953175	-4220.759188	-4220.831788	
**Ph-6-123456-R4-Nu4**	0.151295	0.167552	0.109271	-984.408332	-984.240780	-984.299061	
**Ph-6-123456-R5**	0.118143	0.139034	0.068756	-1459.198014	-1459.058980	-1459.129258	
**Ph-6-123456-R5-Nu1**	0.262599	0.291558	0.205281	-1843.886283	-1843.594725	-1843.681002	
**Ph-6-123456-R5-Nu3**	0.267854	0.296426	0.211556	-1861.897677	-1861.601251	-1861.686121	
**Ph-6-123456-R5-Nu2**	0.240693	0.271495	0.178009	-4852.547906	-4852.276411	-4852.369897	
**Ph-6-123456-R5-Nu4**	0.219927	0.245288	0.165084	-1616.014581	-1615.769293	-1615.849497	
**Py-3-246-R1**	0.105491	0.121316	0.057386	-1259.538830	-1259.417514	-1259.481444	
**Py-3-246-R1-Nu1**	0.246274	0.271422	0.190757	-1644.136026	-1643.864604	-1643.945269	
**Py-3-246-R1-Nu3**	0.251492	0.276360	0.196239	-1662.145219	-1661.868859	-1661.948980	
**Py-3-246-R1-Nu2**	0.225446	0.251843	0.166847	-4652.806969	-4652.555126	-4652.640122	
**Py-3-246-R1-Nu4**	0.203549	0.224711	0.152372	-1416.247938	-1416.023227	-1416.095566	
**Py-3-246-R2**	0.085551	0.095989	0.049925	-525.004103	-524.908114	-524.954178	
**Py-3-246-R2-Nu1**	0.225607	0.246095	0.178352	-909.616472	-909.370377	-909.438120	
**Py-3-246-R2-Nu3**	0.231189	0.251088	0.183927	-927.618642	-927.367554	-927.434715	
**Py-3-246-R2-Nu2**	0.204771	0.226319	0.152938	-3918.275860	-3918.049541	-3918.122922	

Structure	*ZPE*	*TCH*	*TCG*	*E*	*H*	*G*	Imaginary frequency (cm^{-1})
**Py-3-246-R2-Nu4**	0.183747	0.199758	0.140948	-681.721640	-681.521882	-681.580692	
**Py-3-246-R3**	0.219787	0.238535	0.170686	-931.934092	-931.695557	-931.763406	
**Py-3-246-R3-Nu1**	0.359954	0.388087	0.302487	-1316.516733	-1316.128646	-1316.214246	
**Py-3-246-R3-Nu3**	0.365424	0.393334	0.307002	-1334.519515	-1334.126181	-1334.212513	
**Py-3-246-R3-Nu2**	0.339755	0.368982	0.278668	-4325.185188	-4324.816206	-4324.906520	
**Py-3-246-R3-Nu4**	0.318415	0.342031	0.265959	-1088.620664	-1088.278633	-1088.354705	
**Py-3-246-R4**	0.065816	0.073180	0.035264	-546.046142	-545.972962	-546.010878	
**Py-3-246-R4-Nu1**	0.205791	0.223277	0.162439	-930.654601	-930.431324	-930.492162	
**Py-3-246-R4-Nu3**	0.211250	0.228133	0.167646	-948.656844	-948.428711	-948.489198	
**Py-3-246-R4-Nu2**	0.185091	0.203485	0.138096	-3939.312276	-3939.108791	-3939.174180	
**Py-3-246-R4-Nu4**	0.163715	0.176620	0.125342	-702.754659	-702.578039	-702.629317	
**Py-3-246-R5**	0.098680	0.111333	0.058936	-861.799455	-861.688122	-861.740519	
**Py-3-246-R5-Nu1**	0.239482	0.261965	0.188860	-1246.436319	-1246.174354	-1246.247459	
**Py-3-246-R5-Nu3**	0.244384	0.266825	0.191649	-1264.437257	-1264.170432	-1264.245608	
**Py-3-246-R5-Nu2**	0.218031	0.241949	0.161625	-4255.088684	-4254.846735	-4254.927059	
**Py-3-246-R5-Nu4**	0.197007	0.215510	0.149361	-1018.545901	-1018.330391	-1018.396540	
**Py-3-345-R1**	0.106163	0.121352	0.063850	-1259.520875	-1259.399523	-1259.457025	
**Py-3-345-R1-Nu1**	0.246990	0.271334	0.195588	-1644.103863	-1643.832529	-1643.908275	
**Py-3-345-R1-Nu3**	0.252133	0.276149	0.200993	-1662.114245	-1661.838096	-1661.913252	
**Py-3-345-R1-Nu2**	0.225056	0.251263	0.168018	-4652.778147	-4652.526884	-4652.610129	
**Py-3-345-R1-Nu4**	0.204196	0.224642	0.156521	-1416.228723	-1416.004081	-1416.072202	
**Py-3-345-R2**	0.085693	0.096215	0.050750	-525.002830	-524.906615	-524.952080	
**Py-3-345-R2-Nu1**	0.225897	0.246263	0.178621	-909.613653	-909.367390	-909.435032	
**Py-3-345-R2-Nu3**	0.231378	0.251278	0.183867	-927.621515	-927.370237	-927.437648	
**Py-3-345-R2-Nu2**	0.205202	0.226721	0.153713	-3918.277240	-3918.050519	-3918.123527	
**Py-3-345-R2-Nu4**	0.184425	0.200256	0.142172	-681.723916	-681.523660	-681.581744	
**Py-3-345-R3**	0.220059	0.238650	0.173167	-931.928590	-931.689940	-931.755423	
**Py-3-345-R3-Nu1**	0.360253	0.388083	0.304087	-1316.512106	-1316.124023	-1316.208019	
**Py-3-345-R3-Nu3**	0.365949	0.393367	0.309824	-1334.522972	-1334.129605	-1334.213148	
**Py-3-345-R3-Nu2**	0.339841	0.368823	0.280574	-4325.187294	-4324.818471	-4324.906720	
**Py-3-345-R3-Nu4**	0.318748	0.342172	0.267444	-1088.625452	-1088.283280	-1088.358008	
**Py-3-345-R4**	0.065724	0.073280	0.035088	-546.011344	-545.938064	-545.976256	
**Py-3-345-R4-Nu1**	0.205728	0.223124	0.162530	-930.608513	-930.385389	-930.445983	
**Py-3-345-R4-Nu3**	0.210941	0.228138	0.167283	-948.612764	-948.384626	-948.445481	
**Py-3-345-R4-Nu2**	0.184450	0.203206	0.136684	-3939.274256	-3939.071050	-3939.137572	
**Py-3-345-R4-Nu4**	0.163291	0.176512	0.124558	-702.716302	-702.539790	-702.591744	
**Py-3-345-R5**	0.098803	0.111493	0.059778	-861.775080	-861.663587	-861.715302	
**Py-3-345-R5-Nu1**	0.240367	0.262161	0.191156	-1246.405258	-1246.143097	-1246.214102	
**Py-3-345-R5-Nu3**	0.245891	0.267352	0.196663	-1264.420050	-1264.152698	-1264.223387	
**Py-3-345-R5-Nu2**	0.219697	0.242684	0.167467	-4255.076753	-4254.834069	-4254.909286	
**Py-3-345-R5-Nu4**	0.198351	0.216186	0.152527	-1018.535119	-1018.318933	-1018.382592	
**Py-3-426-R1**	0.105612	0.121365	0.059362	-1259.538824	-1259.417459	-1259.479462	
**Py-3-426-R1-Nu1**	0.246630	0.271806	0.190463	-1644.142309	-1643.870503	-1643.951846	
**Py-3-426-R1-Nu3**	0.251971	0.276774	0.196995	-1662.153903	-1661.877129	-1661.956908	
**Py-3-426-R1-Nu2**	0.225530	0.252080	0.166122	-4652.812341	-4652.560261	-4652.646219	
**Py-3-426-R1-Nu4**	0.204557	0.225492	0.153810	-1416.254182	-1416.028690	-1416.100372	

Structure	*ZPE*	*TCH*	*TCG*	*E*	*H*	*G*	Imaginary frequency (cm^{-1})
**Py-3-426-R2**	0.085565	0.095994	0.050614	-525.004118	-524.908124	-524.953504	
**Py-3-426-R2-Nu1**	0.225714	0.246237	0.177973	-909.620624	-909.374387	-909.442651	
**Py-3-426-R2-Nu3**	0.231474	0.251355	0.184052	-927.624841	-927.373486	-927.440789	
**Py-3-426-R2-Nu2**	0.205234	0.226732	0.153132	-3918.283048	-3918.056316	-3918.129916	
**Py-3-426-R2-Nu4**	0.183821	0.199937	0.140047	-681.725495	-681.525558	-681.585448	
**Py-3-426-R3**	0.219862	0.238578	0.170692	-931.934085	-931.695507	-931.763393	
**Py-3-426-R3-Nu1**	0.360565	0.388608	0.303133	-1316.522193	-1316.133585	-1316.219060	
**Py-3-426-R3-Nu3**	0.365501	0.393417	0.307472	-1334.528693	-1334.135276	-1334.221221	
**Py-3-426-R3-Nu2**	0.339374	0.368951	0.275974	-4325.187305	-4324.818354	-4324.911331	
**Py-3-426-R3-Nu4**	0.318538	0.342347	0.264432	-1088.623999	-1088.281652	-1088.359567	
**Py-3-426-R4**	0.065812	0.073176	0.035261	-546.046145	-545.972969	-546.010884	
**Py-3-426-R4-Nu1**	0.206562	0.223645	0.163475	-930.654854	-930.431209	-930.491379	
**Py-3-426-R4-Nu3**	0.211911	0.228590	0.168738	-948.664352	-948.435762	-948.495614	
**Py-3-426-R4-Nu2**	0.185798	0.204072	0.138823	-3939.321456	-3939.117384	-3939.182633	
**Py-3-426-R4-Nu4**	0.164576	0.177316	0.126555	-702.761568	-702.584252	-702.635013	
**Py-3-426-R5**	0.098678	0.111333	0.058913	-861.799459	-861.688126	-861.740546	
**Py-3-426-R5-Nu1**	0.239792	0.262272	0.188240	-1246.439183	-1246.176911	-1246.250943	
**Py-3-426-R5-Nu3**	0.244864	0.267141	0.191773	-1264.443476	-1264.176335	-1264.251703	
**Py-3-426-R5-Nu2**	0.218757	0.242552	0.161612	-4255.096083	-4254.853531	-4254.934471	
**Py-3-426-R5-Nu4**	0.197538	0.215821	0.150127	-1018.548451	-1018.332630	-1018.398324	
**Py-3-435-R1**	0.106160	0.121354	0.063820	-1259.520867	-1259.399513	-1259.457047	
**Py-3-435-R1-Nu1**	0.248897	0.272594	0.199762	-1644.120370	-1643.847776	-1643.920608	
**Py-3-435-R1-Nu3**	0.253149	0.277060	0.201921	-1662.128057	-1661.850997	-1661.926136	
**Py-3-435-R1-Nu2**	0.226212	0.252181	0.169750	-4652.794322	-4652.542142	-4652.624572	
**Py-3-435-R1-Nu4**	0.205484	0.225746	0.158572	-1416.242457	-1416.016711	-1416.083885	
**Py-3-435-R2**	0.085690	0.096212	0.050748	-525.002819	-524.906607	-524.952071	
**Py-3-435-R2-Nu1**	0.226921	0.247087	0.180548	-909.634650	-909.387563	-909.454102	
**Py-3-435-R2-Nu3**	0.232432	0.252042	0.186111	-927.642511	-927.390469	-927.456400	
**Py-3-435-R2-Nu2**	0.205813	0.227194	0.154556	-3918.297978	-3918.070784	-3918.143422	
**Py-3-435-R2-Nu4**	0.184994	0.200882	0.142121	-681.746153	-681.545271	-681.604032	
**Py-3-435-R3**	0.219955	0.238603	0.172332	-931.930550	-931.691947	-931.758218	
**Py-3-435-R3-Nu1**	0.362391	0.389521	0.308517	-1316.539468	-1316.149947	-1316.230951	
**Py-3-435-R3-Nu3**	0.367276	0.394306	0.312700	-1334.549614	-1334.155308	-1334.236914	
**Py-3-435-R3-Nu2**	0.340511	0.369483	0.280733	-4325.211425	-4324.841942	-4324.930692	
**Py-3-435-R3-Nu4**	0.319514	0.342868	0.268395	-1088.651956	-1088.309088	-1088.383561	
**Py-3-435-R4**	0.065720	0.073275	0.035087	-546.011352	-545.938077	-545.976265	
**Py-3-435-R4-Nu1**	0.206504	0.223708	0.163837	-930.614266	-930.390558	-930.450429	
**Py-3-435-R4-Nu3**	0.211569	0.228610	0.167633	-948.620609	-948.391999	-948.452976	
**Py-3-435-R4-Nu2**	0.185506	0.203938	0.138636	-3939.284303	-3939.080365	-3939.145667	
**Py-3-435-R4-Nu4**	0.164501	0.177344	0.126951	-702.726210	-702.548866	-702.599259	
**Py-3-435-R5**	0.098885	0.111525	0.060167	-861.775085	-861.663560	-861.714918	
**Py-3-435-R5-Nu1**	0.242178	0.263293	0.195098	-1246.436581	-1246.173288	-1246.241483	
**Py-3-435-R5-Nu3**	0.246667	0.267789	0.198458	-1264.433812	-1264.166023	-1264.235354	
**Py-3-435-R5-Nu2**	0.219681	0.242888	0.165716	-4255.093827	-4254.850939	-4254.928111	
**Py-3-435-R5-Nu4**	0.199402	0.216781	0.155491	-1018.557453	-1018.340672	-1018.401962	
**Py-4-2356-R1**	0.111272	0.130116	0.064531	-1596.610303	-1596.480187	-1596.545772	

Structure	*ZPE*	*TCH*	*TCG*	*E*	*H*	*G*	Imaginary frequency (cm^{-1})
**Py-4-2356-R1-Nu1**	0.252752	0.280722	0.196660	-1981.226490	-1980.945768	-1981.029830	
**Py-4-2356-R1-Nu3**	0.257735	0.285549	0.200708	-1999.231711	-1998.946162	-1999.031003	
**Py-4-2356-R1-Nu2**	0.231079	0.260668	0.170068	-4989.893359	-4989.632691	-4989.723291	
**Py-4-2356-R1-Nu4**	0.210018	0.234243	0.156318	-1753.346853	-1753.112610	-1753.190535	
**Py-4-2356-R2**	0.084038	0.096395	0.046320	-617.239642	-617.143247	-617.193322	
**Py-4-2356-R2-Nu1**	0.225558	0.247504	0.176417	-1001.887252	-1001.639748	-1001.710835	
**Py-4-2356-R2-Nu3**	0.230615	0.252337	0.180682	-1019.884835	-1019.632498	-1019.704153	
**Py-4-2356-R2-Nu2**	0.204241	0.227551	0.149647	-4010.545415	-4010.317864	-4010.395768	
**Py-4-2356-R2-Nu4**	0.183436	0.201187	0.137310	-773.994682	-773.793495	-773.857372	
**Py-4-2356-R3**	0.263137	0.286375	0.209419	-1159.810363	-1159.523988	-1159.600944	
**Py-4-2356-R3-Nu1**	0.404356	0.436754	0.342294	-1544.427035	-1543.990281	-1544.084741	
**Py-4-2356-R3-Nu3**	0.409950	0.441942	0.347800	-1562.434312	-1561.992370	-1562.086512	
**Py-4-2356-R3-Nu2**	0.383407	0.417101	0.317277	-4553.094718	-4552.677617	-4552.777441	
**Py-4-2356-R3-Nu4**	0.362647	0.390651	0.305366	-1316.538391	-1316.147740	-1316.233025	
**Py-4-2356-R4**	0.057797	0.066066	0.026031	-645.278800	-645.212734	-645.252769	
**Py-4-2356-R4-Nu1**	0.197978	0.216250	0.153454	-1029.884687	-1029.668437	-1029.731233	
**Py-4-2356-R4-Nu3**	0.203224	0.221194	0.158292	-1047.883786	-1047.662592	-1047.725494	
**Py-4-2356-R4-Nu2**	0.176503	0.196228	0.126089	-4038.543348	-4038.347120	-4038.417259	
**Py-4-2356-R4-Nu4**	0.155817	0.169752	0.116216	-801.988459	-801.818707	-801.872243	
**Py-4-2356-R5**	0.101633	0.116966	0.057785	-1066.273754	-1066.156788	-1066.215969	
**Py-4-2356-R5-Nu1**	0.244030	0.268524	0.191449	-1450.960329	-1450.691805	-1450.768880	
**Py-4-2356-R5-Nu3**	0.249210	0.273365	0.196105	-1468.956227	-1468.682862	-1468.760122	
**Py-4-2356-R5-Nu2**	0.222380	0.248395	0.163912	-4459.612413	-4459.364018	-4459.448501	
**Py-4-2356-R5-Nu4**	0.201705	0.222183	0.152071	-1223.074883	-1222.852700	-1222.922812	
**Py-4-3256-R1**	0.111267	0.130113	0.064524	-1596.610303	-1596.480190	-1596.545779	
**Py-4-3256-R1-Nu1**	0.252413	0.280380	0.195692	-1981.216017	-1980.935637	-1981.020325	
**Py-4-3256-R1-Nu3**	0.257319	0.285231	0.200049	-1999.226120	-1998.940889	-1999.026071	
**Py-4-3256-R1-Nu2**	0.230637	0.260383	0.169035	-4989.887820	-4989.627437	-4989.718785	
**Py-4-3256-R1-Nu4**	0.209787	0.233875	0.156853	-1753.336347	-1753.102472	-1753.179494	
**Py-4-3256-R2**	0.084037	0.096394	0.046320	-617.239641	-617.143247	-617.193321	
**Py-4-3256-R2-Nu1**	0.224736	0.246835	0.174956	-1001.872856	-1001.626021	-1001.697900	
**Py-4-3256-R2-Nu3**	0.230378	0.252002	0.180612	-1019.880056	-1019.628054	-1019.699444	
**Py-4-3256-R2-Nu2**	0.203791	0.227101	0.149670	-4010.538820	-4010.311719	-4010.389150	
**Py-4-3256-R2-Nu4**	0.183131	0.200785	0.137944	-773.985264	-773.784479	-773.847320	
**Py-4-3256-R3**	0.262898	0.286295	0.208474	-1159.809506	-1159.523211	-1159.601032	
**Py-4-3256-R3-Nu1**	0.404425	0.436563	0.343332	-1544.417828	-1543.981265	-1544.074496	
**Py-4-3256-R3-Nu3**	0.409683	0.441568	0.347584	-1562.424205	-1561.982637	-1562.076621	
**Py-4-3256-R3-Nu2**	0.383264	0.416854	0.317299	-4553.089162	-4552.672308	-4552.771863	
**Py-4-3256-R3-Nu4**	0.362215	0.390123	0.304885	-1316.528137	-1316.138014	-1316.223252	
**Py-4-3256-R4**	0.057799	0.066070	0.026029	-645.278784	-645.212714	-645.252755	
**Py-4-3256-R4-Nu1**	0.198469	0.216963	0.153278	-1029.913337	-1029.696374	-1029.760059	
**Py-4-3256-R4-Nu3**	0.203032	0.220871	0.158689	-1047.883497	-1047.662626	-1047.724808	
**Py-4-3256-R4-Nu2**	0.176479	0.196037	0.127633	-4038.536115	-4038.340078	-4038.408482	
**Py-4-3256-R4-Nu4**	0.155179	0.169283	0.115335	-801.985257	-801.815974	-801.869922	
**Py-4-3256-R5**	0.101618	0.116947	0.058460	-1066.274045	-1066.157098	-1066.215585	
**Py-4-3256-R5-Nu1**	0.243636	0.268061	0.190777	-1450.945679	-1450.677618	-1450.754902	

Structure	*ZPE*	*TCH*	*TCG*	*E*	*H*	*G*	Imaginary frequency (cm^{-1})
**Py-4-3256-R5-Nu3**	0.248959	0.273093	0.195332	-1468.945478	-1468.672385	-1468.750146	
**Py-4-3256-R5-Nu2**	0.222403	0.248263	0.164493	-4459.598740	-4459.350477	-4459.434247	
**Py-4-3256-R5-Nu4**	0.201603	0.221986	0.151792	-1223.061623	-1222.839637	-1222.909831	
**Py-5-23456-R1**	0.116428	0.138317	0.066613	-1933.668784	-1933.530467	-1933.602171	
**Py-5-23456-R1-Nu1**	0.258187	0.289095	0.200416	-2318.293111	-2318.004016	-2318.092695	
**Py-5-23456-R1-Nu3**	0.263276	0.293893	0.205297	-2336.305171	-2336.011278	-2336.099874	
**Py-5-23456-R1-Nu2**	0.236610	0.269239	0.173226	-5326.965106	-5326.695867	-5326.791880	
**Py-5-23456-R1-Nu4**	0.215836	0.242854	0.161006	-2090.416542	-2090.173688	-2090.255536	
**Py-5-23456-R2**	0.082444	0.096718	0.042135	-709.468917	-709.372199	-709.426782	
**Py-5-23456-R2-Nu1**	0.223741	0.247773	0.171799	-1094.127585	-1093.879812	-1093.955786	
**Py-5-23456-R2-Nu3**	0.229259	0.252823	0.176530	-1112.131955	-1111.879132	-1111.955425	
**Py-5-23456-R2-Nu2**	0.203074	0.228013	0.148151	-4102.784289	-4102.556276	-4102.636138	
**Py-5-23456-R2-Nu4**	0.182284	0.201864	0.134379	-866.239749	-866.037885	-866.105370	
**Py-5-23456-R3**	0.306190	0.334081	0.246348	-1387.691893	-1387.357812	-1387.445545	
**Py-5-23456-R3-Nu1**	0.448027	0.484745	0.381303	-1772.309677	-1771.824932	-1771.928374	
**Py-5-23456-R3-Nu3**	0.453242	0.489695	0.385936	-1790.320343	-1789.830648	-1789.934407	
**Py-5-23456-R3-Nu2**	0.427691	0.465429	0.358357	-4780.978391	-4780.512962	-4780.620034	
**Py-5-23456-R3-Nu4**	0.405798	0.438426	0.342311	-1544.421243	-1543.982817	-1544.078932	
**Py-5-23456-R4**	0.049958	0.059187	0.016955	-744.516825	-744.457638	-744.499870	
**Py-5-23456-R4-Nu1**	0.189683	0.209216	0.143082	-1129.118484	-1128.909268	-1128.975402	
**Py-5-23456-R4-Nu3**	0.195710	0.214593	0.149855	-1147.130531	-1146.915938	-1146.980676	
**Py-5-23456-R4-Nu2**	0.169055	0.189536	0.119140	-4137.783241	-4137.593705	-4137.664101	
**Py-5-23456-R4-Nu4**	0.148391	0.163245	0.107811	-901.233643	-901.070398	-901.125832	
**Py-5-23456-R5**	0.104072	0.122077	0.057568	-1270.751213	-1270.629136	-1270.693645	
**Py-5-23456-R5-Nu1**	0.246850	0.273895	0.191026	-1655.442486	-1655.168591	-1655.251460	
**Py-5-23456-R5-Nu3**	0.252475	0.278902	0.198068	-1673.451342	-1673.172440	-1673.253274	
**Py-5-23456-R5-Nu2**	0.225501	0.254035	0.164583	-4664.100397	-4663.846362	-4663.935814	
**Py-5-23456-R5-Nu4**	0.204749	0.227720	0.152331	-1427.564492	-1427.336772	-1427.412161	
**Py-5-32456-R1**	0.116428	0.138317	0.066613	-1933.668784	-1933.530467	-1933.602171	
**Py-5-32456-R1-Nu1**	0.257530	0.288540	0.198776	-2318.275818	-2317.987278	-2318.077042	
**Py-5-32456-R1-Nu3**	0.262997	0.293520	0.204930	-2336.288314	-2335.994794	-2336.083384	
**Py-5-32456-R1-Nu2**	0.236254	0.268917	0.173010	-5326.948494	-5326.679577	-5326.775484	
**Py-5-32456-R1-Nu4**	0.215604	0.242778	0.158968	-2090.407372	-2090.164594	-2090.248404	
**Py-5-32456-R2**	0.082444	0.096718	0.042135	-709.468917	-709.372199	-709.426782	
**Py-5-32456-R2-Nu1**	0.223876	0.247697	0.172692	-1094.128631	-1093.880934	-1093.955939	
**Py-5-32456-R2-Nu3**	0.229010	0.252535	0.177020	-1112.132024	-1111.879489	-1111.955004	
**Py-5-32456-R2-Nu2**	0.202590	0.227573	0.147563	-4102.776939	-4102.549366	-4102.629376	
**Py-5-32456-R2-Nu4**	0.181931	0.201510	0.133371	-866.239223	-866.037713	-866.105852	
**Py-5-32456-R3**	0.306190	0.334081	0.246348	-1387.691893	-1387.357812	-1387.445545	
**Py-5-32456-R3-Nu1**	0.448506	0.484630	0.383229	-1772.308798	-1771.824168	-1771.925569	
**Py-5-32456-R3-Nu3**	0.453643	0.489523	0.388119	-1790.320544	-1789.831021	-1789.932425	
**Py-5-32456-R3-Nu2**	0.427134	0.464935	0.356929	-4780.979371	-4780.514436	-4780.622442	
**Py-5-32456-R3-Nu4**	0.405902	0.438212	0.343275	-1544.425642	-1543.987430	-1544.082367	
**Py-5-32456-R4**	0.049958	0.059187	0.016955	-744.516825	-744.457638	-744.499870	
**Py-5-32456-R4-Nu1**	0.189875	0.209089	0.144320	-1129.110136	-1128.901047	-1128.965816	
**Py-5-32456-R4-Nu3**	0.195205	0.214106	0.149793	-1147.123718	-1146.909612	-1146.973925	

Structure	*ZPE*	*TCH*	*TCG*	*E*	*H*	*G*	Imaginary frequency (cm^{-1})
**Py-5-32456-R4-Nu2**	0.168730	0.189151	0.119184	-4137.774326	-4137.585175	-4137.655142	
**Py-5-32456-R4-Nu4**	0.147723	0.162757	0.106942	-901.230746	-901.067989	-901.123804	
**Py-5-32456-R5**	0.104072	0.122077	0.057568	-1270.751213	-1270.629136	-1270.693645	
**Py-5-32456-R5-Nu1**	0.247407	0.273781	0.192515	-1655.437588	-1655.163807	-1655.245073	
**Py-5-32456-R5-Nu3**	0.252359	0.278538	0.198178	-1673.448000	-1673.169462	-1673.249822	
**Py-5-32456-R5-Nu2**	0.225603	0.253729	0.166233	-4664.099931	-4663.846202	-4663.933698	
**Py-5-32456-R5-Nu4**	0.204607	0.227377	0.152541	-1427.566131	-1427.338754	-1427.413590	
**Py-5-42356-R1**	0.116428	0.138317	0.066613	-1933.668784	-1933.530467	-1933.602171	
**Py-5-42356-R1-Nu1**	0.258124	0.289027	0.200124	-2318.283261	-2317.994234	-2318.083137	
**Py-5-42356-R1-Nu3**	0.263658	0.294092	0.205929	-2336.294611	-2336.000519	-2336.088682	
**Py-5-42356-R1-Nu2**	0.236796	0.269516	0.173174	-5326.957341	-5326.687825	-5326.784167	
**Py-5-42356-R1-Nu4**	0.215975	0.243081	0.159895	-2090.411114	-2090.168033	-2090.251219	
**Py-5-42356-R2**	0.082444	0.096718	0.042135	-709.468917	-709.372199	-709.426782	
**Py-5-42356-R2-Nu1**	0.223756	0.247797	0.171793	-1094.131345	-1093.883548	-1093.959552	
**Py-5-42356-R2-Nu3**	0.229294	0.252752	0.177671	-1112.136979	-1111.884227	-1111.959308	
**Py-5-42356-R2-Nu2**	0.202608	0.227705	0.146994	-4102.776008	-4102.548303	-4102.629014	
**Py-5-42356-R2-Nu4**	0.182167	0.201741	0.134735	-866.243179	-866.041438	-866.108444	
**Py-5-42356-R3**	0.306190	0.334081	0.246348	-1387.691893	-1387.357812	-1387.445545	
**Py-5-42356-R3-Nu1**	0.448751	0.485177	0.382225	-1772.307145	-1771.821968	-1771.924920	
**Py-5-42356-R3-Nu3**	0.453729	0.489945	0.386638	-1790.317465	-1789.827520	-1789.930827	
**Py-5-42356-R3-Nu2**	0.427159	0.465336	0.354536	-4780.974958	-4780.509622	-4780.620422	
**Py-5-42356-R3-Nu4**	0.406121	0.438532	0.343584	-1544.421038	-1543.982506	-1544.077454	
**Py-5-42356-R4**	0.049958	0.059187	0.016955	-744.516825	-744.457638	-744.499870	
**Py-5-42356-R4-Nu1**	0.191002	0.209953	0.146079	-1129.135890	-1128.925937	-1128.989811	
**Py-5-42356-R4-Nu3**	0.196506	0.215063	0.151216	-1147.145746	-1146.930683	-1146.994530	
**Py-5-42356-R4-Nu2**	0.169828	0.190045	0.120355	-4137.797971	-4137.607926	-4137.677616	
**Py-5-42356-R4-Nu4**	0.149132	0.163697	0.109070	-901.248309	-901.084612	-901.139239	
**Py-5-42356-R5**	0.104072	0.122077	0.057568	-1270.751213	-1270.629136	-1270.693645	
**Py-5-42356-R5-Nu1**	0.247939	0.274441	0.193100	-1655.440027	-1655.165586	-1655.246927	
**Py-5-42356-R5-Nu3**	0.252688	0.279079	0.197213	-1673.446549	-1673.167470	-1673.249336	
**Py-5-42356-R5-Nu2**	0.225842	0.254222	0.164539	-4664.099071	-4663.844849	-4663.934532	
**Py-5-42356-R5-Nu4**	0.205093	0.227912	0.153089	-1427.563575	-1427.335663	-1427.410486	
**TS-Ph-4-1234-R1-Nu4**	0.217509	0.241680	0.165359	-1737.216775	-1736.975095	-1737.051416	327.6 *i*
**TS-Ph-4-1234-R3-Nu4**	0.370324	0.398265	0.314540	-1300.424877	-1300.026612	-1300.110337	278.0 *i*
**TS-Ph-4-1234-R5-Nu2**	0.231598	0.257946	0.173204	-4443.499552	-4443.241606	-4443.326348	57.6 *i*
**TS-Ph-4-1235-R1-Nu4**	0.217077	0.241578	0.164136	-1737.231573	-1736.989995	-1737.067437	165.1 *i*
**TS-Ph-4-1235-R4-Nu4**	0.162011	0.176876	0.121310	-785.868802	-785.691926	-785.747492	222.7 *i*
**TS-Ph-4-1245-R1-Nu4**	0.217933	0.242115	0.166613	-1737.242202	-1737.000087	-1737.075589	283.9 *i*
**TS-Ph-4-1245-R3-Nu4**	0.370110	0.398555	0.312190	-1300.433163	-1300.034608	-1300.120973	81.7 *i*
**TS-Ph-4-2134-R1-Nu4**	0.218989	0.242580	0.168686	-1737.212529	-1736.969949	-1737.043843	354.8 *i*
**TS-Ph-4-2134-R3-Nu4**	0.370125	0.398214	0.313615	-1300.423448	-1300.025234	-1300.109833	284.7 *i*
**TS-Ph-4-2134-R4-Nu4**	0.162349	0.177306	0.121405	-785.864647	-785.687341	-785.743242	203.3 *i*
**TS-Ph-4-2134-R5-Nu2**	0.231372	0.257687	0.173328	-4443.496500	-4443.238813	-4443.323172	122.5 *i*
**TS-Ph-4-2135-R1-Nu4**	0.218107	0.242177	0.166250	-1737.233552	-1736.991375	-1737.067302	408.8 *i*
**TS-Ph-4-2135-R3-Nu4**	0.370383	0.398395	0.313454	-1300.436099	-1300.037704	-1300.122645	315.1 *i*
**TS-Ph-4-2135-R5-Nu2**	0.231293	0.257732	0.172469	-4443.513503	-4443.255771	-4443.341034	69.3 *i*
**TS-Ph-4-5123-R1-Nu4**	0.217483	0.241974	0.164286	-1737.235571	-1736.993597	-1737.071285	260.9 *i*
**TS-Ph-4-5123-R5-Nu2**	0.231570	0.257908	0.172247	-4443.513185	-4443.255277	-4443.340938	29.4 *i*
**TS-Ph-5-12345-R3-Nu4**	0.413684	0.446418	0.351003	-1528.315302	-1527.868884	-1527.964299	132.3 *i*
**TS-Ph-5-12345-R4-Nu4**	0.154317	0.170075	0.112839	-885.105772	-884.935697	-884.992933	259.6 *i*
**TS-Ph-5-21345-R3-Nu4**	0.414074	0.446395	0.351989	-1528.313786	-1527.867391	-1527.961797	366.1 *i*

Structure	*ZPE*	*TCH*	*TCG*	*E*	*H*	*G*	Imaginary frequency (cm^{-1})
**TS-Ph-5-21345-R4-Nu4**	0.153813	0.169969	0.109889	-885.107925	-884.937956	-884.998036	208.9 *i*
**TS-Ph-5-31245-R3-Nu4**	0.413292	0.446201	0.350263	-1528.311072	-1527.864871	-1527.960809	209.6 *i*
**TS-Ph-6-123456-R3-Nu4**	0.457398	0.494545	0.389335	-1756.193903	-1755.699358	-1755.804568	282.7 *i*
**TS-Ph-6-123456-R4-Nu4**	0.146235	0.163212	0.102452	-984.347279	-984.184067	-984.244827	142.5 *i*
**TS-Py-3-246-R1-Nu4**	0.199576	0.220948	0.149659	-1416.202472	-1415.981524	-1416.052813	185.0 *i*
**TS-Py-3-246-R4-Nu4**	0.158653	0.172322	0.119391	-702.692805	-702.520483	-702.573414	194.0 *i*
**TS-Py-3-345-R1-Nu4**	0.201836	0.222152	0.155042	-1416.185005	-1415.962853	-1416.029963	368.6 *i*
**TS-Py-3-345-R4-Nu4**	0.158887	0.172685	0.119464	-702.657217	-702.484532	-702.537753	239.8 *i*
**TS-Py-3-426-R4-Nu4**	0.158772	0.172567	0.118360	-702.695086	-702.522519	-702.576726	129.8 *i*
**TS-Py-3-435-R1-Nu4**	0.202072	0.222299	0.155502	-1416.187724	-1415.965425	-1416.032222	359.4 *i*
**TS-Py-3-435-R3-Nu4**	0.315788	0.338963	0.265661	-1088.586970	-1088.248007	-1088.321309	327.0 *i*
**TS-Py-3-435-R5-Nu3**	0.243414	0.265096	0.193785	-1264.386020	-1264.120924	-1264.192235	115.8 *i*
**TS-Py-3-435-R5-Nu2**	0.217118	0.240533	0.162955	-4255.041301	-4254.800768	-4254.878346	59.6 *i*
**TS-Py-4-2356-R3-Nu4**	0.358650	0.386803	0.300918	-1316.472181	-1316.085378	-1316.171263	482.9 *i*
**TS-Py-4-2356-R4-Nu4**	0.151504	0.165762	0.111752	-801.931697	-801.765935	-801.819945	239.1 *i*
**TS-Py-4-3256-R3-Nu4**	0.358195	0.386591	0.299780	-1316.474086	-1316.087495	-1316.174306	222.8 *i*
**TS-Py-4-3256-R4-Nu4**	0.150998	0.165535	0.110381	-801.929722	-801.764187	-801.819341	241.0 *i*
**TS-Py-4-3256-R5-Nu1**	0.241589	0.266166	0.188712	-1450.905446	-1450.639280	-1450.716734	85.1 *i*
**TS-Py-5-23456-R2-Nu2**	0.199954	0.225464	0.142429	-4102.732361	-4102.506897	-4102.589932	21.0 *i*
**TS-Py-5-23456-R3-Nu4**	0.403680	0.435527	0.342166	-1544.356041	-1543.920514	-1544.013875	664.5 *i*
**TS-Py-5-23456-R4-Nu4**	0.143146	0.158675	0.101351	-901.171398	-901.012723	-901.070047	267.6 *i*
**TS-Py-5-32456-R3-Nu4**	0.402089	0.434656	0.339588	-1544.355912	-1543.921256	-1544.016324	303.1 *i*
**TS-Py-5-32456-R4-Nu4**	0.142922	0.158652	0.100311	-901.168275	-901.009623	-901.067964	238.3 *i*
**TS-Py-5-42356-R3-Nu4**	0.401809	0.434701	0.337616	-1544.355543	-1543.920842	-1544.017927	298.0 *i*
**TS-Py-5-42356-R4-Nu4**	0.146420	0.160475	0.107114	-901.168782	-901.008307	-901.061668	443.1 *i*

Cartesian Coordinates of Computed Species

Nu1

C	-0.03332300	0.45648900	-0.00009300
C	1.34381600	0.06099300	0.00000600
O	2.29056800	0.87011600	0.00004700
C	-1.11390600	-0.47283400	-0.00007800
O	-1.03966400	-1.71714400	0.00002900
C	-2.52040100	0.13973800	0.00000400
H	-2.68016800	0.77657300	0.88360700
H	-2.67987300	0.77848100	-0.88223800
H	-3.25902400	-0.67134200	-0.00093900
C	1.70045600	-1.42188400	0.00000400
H	1.27254500	-1.93344900	0.87247900
H	1.27260800	-1.93332700	-0.87259300
H	2.79569100	-1.50455300	-0.00003300
C	-0.31184300	1.94188400	-0.00000300
H	0.64811300	2.47343200	0.00024800
H	-0.88274700	2.28215700	-0.88288900
H	-0.88316400	2.28193500	0.88270300

Nu3

C	-1.41369000	0.33783700	0.00000000
C	0.00000000	0.13793400	0.00000000
C	0.90777700	1.23836300	0.00000000

C	0.60490800	-1.15690300	0.00000000
C	2.28760500	1.05236900	0.00000000
H	0.51247400	2.25570800	0.00000000
C	1.98039100	-1.32532700	0.00000000
H	-0.04519400	-2.03534400	0.00000000
C	2.85214800	-0.22547300	0.00000000
H	2.93805400	1.93164100	0.00000000
H	2.38876000	-2.33980600	0.00000000
H	3.93399600	-0.36451200	0.00000000
C	-2.27148300	-0.76706800	0.00000000
N	-2.98654000	-1.69736500	0.00000000
C	-2.00188900	1.72640100	0.00000000
H	-3.10020000	1.68677100	0.00000000
H	-1.69835600	2.31915100	0.88406100
H	-1.69835600	2.31915100	-0.88406100

Nu2

C	0.99066700	-0.28339500	-0.81010000
Br	2.93768400	-0.13532400	-0.46600600
H	0.77226900	0.53754500	-1.51203500
S	0.19521100	0.19716700	0.65950000
O	0.42593400	-0.80180700	1.72028000
O	0.35119400	1.60701600	1.08541900
C	-1.52238600	0.03874200	0.14766900
C	-2.12990100	-1.21516600	0.18963300
C	-2.21948900	1.17091500	-0.26424700
C	-3.46142500	-1.33616800	-0.20178500
H	-1.55285000	-2.07513000	0.53262800
C	-3.55229200	1.04100000	-0.65875500
H	-1.71517600	2.13816300	-0.25863600
C	-4.17004800	-0.20947600	-0.62846600
H	-3.95067200	-2.31099400	-0.17321000
H	-4.11101300	1.91969500	-0.98467600
H	-5.21266200	-0.30798600	-0.93516600

Nu4

C	0.01829300	0.74394300	1.26164300
C	0.01829300	0.13950700	0.00000000
H	-0.00488500	0.14284000	2.17657800
H	-0.03952900	1.83374300	1.37596900
C	-0.02158000	-1.38882900	0.00000000
C	0.01829300	0.74394300	-1.26164300
H	-1.06099400	-1.76606800	0.00000000
H	0.47501300	-1.80923800	-0.89135400
H	0.47501300	-1.80923800	0.89135400
H	-0.03952900	1.83374300	-1.37596900
H	-0.00488500	0.14284000	-2.17657800

Ph-4-1234-R1

C	1.39587100	-1.02117800	0.03067300

C	0.69206300	-2.22023500	0.04379100
C	0.70270900	0.20081500	-0.03407600
C	-0.69205700	-2.22025000	-0.04409200
C	-0.70277200	0.20079400	0.03400800
C	-1.39586000	-1.02119600	-0.03072200
C	-2.91231700	-1.13536200	-0.14131100
C	-1.47666300	1.49893900	0.28221600
C	1.47659300	1.49901100	-0.28222700
C	2.91235300	-1.13532300	0.14135000
F	3.42468100	-0.16589500	0.89548500
F	3.23824400	-2.29186900	0.72057000
F	3.51656700	-1.11472700	-1.04171800
F	2.56077100	1.25183100	-1.01513300
F	1.86908800	2.09834300	0.83279100
F	0.74565800	2.35869100	-0.98868200
F	-2.56082100	1.25161600	1.01500300
F	-0.74571800	2.35857200	0.98879900
F	-1.86905700	2.09845900	-0.83277800
F	-3.51640200	-1.11421500	1.04180500
F	-3.23822100	-2.29210000	-0.72006700
F	-3.42473600	-0.16616700	-0.89577200
H	1.23076500	-3.16446800	0.10354800
H	-1.23079200	-3.16445700	-0.10393000

Ph-4-1234-R1-Nu1

C	-0.96879300	-0.31391600	-0.61268000
C	-0.36007100	-1.52740300	-1.28806200
C	0.17539600	0.68031300	-0.30297900
C	0.93004400	-1.88318700	-1.14043300
C	1.50107700	0.24998400	-0.17946700
C	1.89961300	-1.06729500	-0.47755000
C	3.17964700	-1.72216500	-0.10135400
C	2.58253900	1.21952300	0.28529700
C	-0.19392600	2.11834200	-0.06930600
C	-1.97973400	0.30059800	-1.65986300
F	-1.44984000	1.37230500	-2.27026500
F	-2.28143900	-0.52886900	-2.66315700
F	-3.13829100	0.69040200	-1.13069300
F	-1.52439900	2.25081800	0.11493400
F	0.11679200	2.97622100	-1.05559500
F	0.35798800	2.65358700	1.04186400
F	3.80693900	0.81766800	-0.06349300
F	2.46186900	2.43963000	-0.24472400
F	2.59020200	1.35778600	1.61738000
F	4.22796100	-1.52017300	-0.92949000
F	3.03423000	-3.06389400	-0.07299200
F	3.61440200	-1.38079200	1.12863200
C	-1.73496100	-0.82510600	0.72175000
C	-2.88067900	-1.80504900	0.40164600
C	-2.37483400	0.27382700	1.59761300
C	-0.71828100	-1.59390700	1.59354700
O	-3.25405900	-2.03130000	-0.72183700

C	-3.49022800	-2.55606500	1.56960400
O	-3.56292600	0.49032200	1.53943200
C	-1.52351300	0.96079300	2.64082600
H	-0.46216300	-2.55329800	1.12688200
H	0.20881000	-1.01822700	1.70401000
H	-1.12468800	-1.79301800	2.59433300
H	-2.87503300	-3.44734400	1.76627300
H	-3.53218100	-1.95943800	2.48826400
H	-4.49606300	-2.88555800	1.28518600
H	-1.63173000	0.39360300	3.57864000
H	-0.46037700	0.99163900	2.38468300
H	-1.91060600	1.97337500	2.80305700
H	-1.04189800	-2.17542000	-1.83226800
H	1.25430000	-2.83642900	-1.56296500

Ph-4-1234-R1-Nu3

C	-1.40680900	0.21099800	0.53779700
C	-0.69981700	0.87549700	1.69654600
C	-0.56052300	-0.98216600	0.08294000
C	0.63383800	0.80344400	1.87375700
C	0.82643600	-1.00253400	0.29878600
C	1.47803600	-0.05367600	1.10663800
C	2.94462200	0.19201800	1.21343000
C	1.69930600	-2.05812600	-0.37327200
C	-1.23246200	-2.07894900	-0.68927500
C	-2.81438300	-0.21830300	1.09669800
F	-2.79236800	-1.47635600	1.54215400
F	-3.19458900	0.53259500	2.14014600
F	-3.82841800	-0.11515200	0.22971600
F	-2.50536800	-1.78220000	-0.99911300
F	-1.30510800	-3.26369000	-0.05722600
F	-0.64315100	-2.32998800	-1.88330800
F	2.85251100	-2.24479100	0.27278700
F	1.14349900	-3.27002900	-0.42690000
F	2.01018600	-1.71064500	-1.62947900
F	3.61833400	-0.62237700	2.05586900
F	3.18457600	1.43150200	1.68788600
F	3.59697800	0.12282300	0.03827000
C	-1.61305800	1.32380600	-0.65206800
C	-0.25514800	1.96859100	-0.96435000
C	-2.52281800	2.35779600	-0.12631600
C	-2.22459100	0.80005700	-1.96956800
C	0.64793500	1.30933800	-1.80831600
C	0.12468800	3.19734600	-0.41408400
N	-3.25138100	3.15180500	0.28849300
H	-3.25164100	0.45182300	-1.83559900
H	-2.20807500	1.61147600	-2.70985900
H	-1.62761400	-0.03008000	-2.36251100
C	1.90335200	1.85254800	-2.07204900
H	0.39333700	0.34069600	-2.23648300
C	1.37638700	3.74822500	-0.68657100
H	-0.56135200	3.73076400	0.24561200

C	2.27400000	3.07444000	-1.51283400
H	2.59928800	1.30527100	-2.70973000
H	1.65052900	4.70690900	-0.24329800
H	3.25797200	3.49872600	-1.71900800
H	-1.28587700	1.53213000	2.33580500
H	1.08541900	1.42232800	2.65104300

Ph-4-1234-R1-Nu2

C	0.12576200	0.71379200	0.77910000
C	1.28168100	0.83011500	-0.22577100
C	0.45082500	-0.31203600	1.83699300
C	2.39503300	-0.01584300	-0.16899300
C	1.49146600	-1.16087400	1.75170100
C	2.49139400	-1.08817400	0.73333200
C	3.62030700	0.26955600	-1.03999800
C	1.17624700	1.89958800	-1.27215500
C	3.50019100	-2.18018400	0.70784000
C	-0.07149900	2.06855300	1.51824500
F	1.10864600	2.57647600	1.87084400
F	-0.69384600	2.99447800	0.78452700
F	-0.77258800	1.92135300	2.64653100
F	4.65040200	-1.94347600	1.37600900
F	3.86698000	-2.54592600	-0.53471300
F	3.00448800	-3.29462700	1.28447400
F	3.54925400	-0.27855800	-2.25419800
F	3.84059900	1.57956900	-1.19482200
F	4.73999300	-0.19421000	-0.47575900
F	1.71160700	3.08810800	-0.92702200
F	1.73742500	1.55868700	-2.44218200
F	-0.10481400	2.19043900	-1.59063200
C	-1.23905500	0.31924600	0.05552100
Br	-0.99078000	-1.35962300	-0.86981100
S	-2.75692500	0.22169400	1.09520800
H	-1.52811300	1.05573600	-0.69759600
O	-2.57732400	-0.73423700	2.18460300
O	-3.13570600	1.60405500	1.37970400
C	-4.00316800	-0.43270300	-0.00050500
C	-4.36345500	-1.77377700	0.10675200
C	-4.61931400	0.43427100	-0.90076200
C	-5.36373800	-2.26299300	-0.73051000
H	-3.86244200	-2.41090800	0.83634100
C	-5.61248600	-0.07083900	-1.73796300
H	-4.33348500	1.48686900	-0.93248400
C	-5.98067900	-1.41476200	-1.65242400
H	-5.66258800	-3.30959200	-0.66329200
H	-6.10630500	0.58900700	-2.45199300
H	-6.76122800	-1.80415700	-2.30755400
H	-0.26407400	-0.40518300	2.65138000
H	1.59019800	-1.92905400	2.52087900

Ph-4-1234-R1-Nu4

C	-1.43539500	-0.72701500	-0.35414900
C	-2.44181400	-1.20044700	0.75917500
C	-0.62757300	-1.94064500	-0.75972700
C	-1.79524800	-1.58053600	2.06893900
H	-3.21586200	-0.44156100	0.92774000
H	-2.94341800	-2.08896900	0.34803100
C	-0.49275800	0.44059200	-0.01700000
C	0.71479300	-2.00667800	-0.72864500
C	-1.35878000	-0.47370700	2.99177400
C	-1.62907900	-2.86683800	2.39751900
C	0.89714700	0.32344600	-0.10695000
C	1.55152500	-0.89278100	-0.39107400
H	-2.18801200	0.22403200	3.18322800
H	-1.00416100	-0.87738200	3.94959500
H	-0.54774100	0.11791300	2.54069500
H	-1.94809500	-3.66797100	1.72624100
H	-1.16436900	-3.15537300	3.34392700
C	3.00790300	-1.15988800	-0.27813000
C	1.78092700	1.55716500	0.06953000
C	-1.13659500	1.72416000	0.41366800
C	-2.30035900	-0.35559000	-1.57618600
F	-1.55426400	0.02688900	-2.61340200
F	-3.03868300	-1.39790500	-1.99461900
F	-3.16551000	0.63932800	-1.33657700
F	-2.35300700	1.53554700	0.96072200
F	-1.34496700	2.62024000	-0.57470200
F	-0.44426700	2.38075600	1.36694000
F	2.92788300	1.44339800	-0.60883200
F	1.22093900	2.67309900	-0.40848800
F	2.11018900	1.78499700	1.34459100
F	3.76112700	-0.83910300	-1.35628800
F	3.24103000	-2.47706500	-0.09524000
F	3.59727300	-0.54307100	0.76655800
H	-1.20696300	-2.82574600	-1.03044800
H	1.19077200	-2.95636600	-0.97744100

Ph-4-1234-R2

C	0.00000000	1.39597600	-0.82066200
C	0.00000000	0.69519300	-2.02980800
C	0.00000000	0.70150400	0.40337100
C	0.00000000	-0.69519300	-2.02980800
C	0.00000000	-0.70150400	0.40337100
C	0.00000000	-1.39597600	-0.82066200
C	0.00000000	-2.83673400	-0.82310700
N	0.00000000	-3.99012800	-0.83119900
C	0.00000000	-1.42252700	1.64876100
N	0.00000000	-2.00302300	2.64535200
C	0.00000000	1.42252700	1.64876100
N	0.00000000	2.00302300	2.64535200
C	0.00000000	2.83673400	-0.82310700
N	0.00000000	3.99012800	-0.83119900
H	0.00000000	1.24458300	-2.97040500

H	0.00000000	-1.24458300	-2.97040500

Ph-4-1234-R2-Nu1

C	0.61081800	0.20176100	0.99198100
C	-0.19647400	-0.57746700	2.02689500
C	-0.34526600	1.15607400	0.28559500
C	-1.47078600	-0.93954700	1.77152900
C	-1.63655200	0.71646100	0.01202300
C	-2.18556000	-0.42739500	0.63174100
C	-3.47134800	-0.92009500	0.29027000
N	-4.51652700	-1.34716100	0.01811400
C	-2.46435900	1.48575300	-0.88466000
N	-3.13606500	2.08867300	-1.60535100
C	0.05103000	2.46364500	-0.10574400
N	0.32224100	3.55111900	-0.40554000
C	1.63555200	0.95882300	1.74403300
N	2.40909900	1.49404100	2.41200100
C	1.38458100	-0.79136800	-0.00033000
C	2.33692200	-0.00325100	-0.91837000
C	0.37535500	-1.46683100	-0.94988700
C	2.20113400	-1.81116200	0.80389000
O	2.45487300	1.19239700	-0.83748300
C	3.18092600	-0.80670000	-1.88305000
O	0.07826100	-0.89762400	-1.97506900
C	-0.14274100	-2.84483400	-0.62842800
H	2.96861800	-1.29989800	1.40390200
H	1.55506600	-2.37853100	1.48558300
H	2.70609500	-2.52787900	0.14225500
H	4.11147700	-1.08968800	-1.36716900
H	2.68532600	-1.72349000	-2.22494300
H	3.44174700	-0.17341900	-2.73868600
H	0.65124000	-3.57513500	-0.85347100
H	-0.40080600	-2.95053000	0.43163000
H	-1.01037500	-3.05444400	-1.26402100
H	0.29801600	-0.86720000	2.95420500
H	-2.00209800	-1.58787100	2.47163300

Ph-4-1234-R2-Nu3

C	1.30670000	-0.25619400	0.84283400
C	0.61200600	-1.32299700	0.01271300
C	0.56021500	-0.08470100	2.15274000
C	-0.76629300	-1.50089400	0.13291900
C	-0.76439700	-0.32749700	2.24876400
C	-1.51206300	-0.94163400	1.18407000
C	-1.44486400	-2.31760500	-0.84470600
N	-1.99608700	-2.95785300	-1.63210800
C	1.34764800	-2.05754000	-0.95404200
N	1.95180600	-2.66643500	-1.73657700
C	-2.92113000	-1.09337800	1.26478000
N	-4.07415300	-1.20410900	1.34406300
C	2.69061900	-0.68220700	1.14909700

N	3.76887200	-1.00185000	1.40623800
C	1.42120800	1.16510200	0.05738600
C	0.03392200	1.59503300	-0.41330700
C	2.29220100	0.95953200	-1.11472500
C	2.10454500	2.20645900	0.95746700
C	-0.77631600	2.40034800	0.39461000
C	-0.47980800	1.13083000	-1.63151300
N	3.00351000	0.81075900	-2.01117900
H	3.13504900	1.90099900	1.18301600
H	2.12611000	3.18257000	0.45583000
H	1.56011900	2.31031500	1.90357100
C	-2.07991500	2.70918600	0.00728000
H	-0.40255200	2.78093600	1.34454800
C	-1.78300900	1.43629200	-2.01751500
H	0.14161100	0.51173900	-2.28206100
C	-2.59082100	2.22108900	-1.19437600
H	-2.70014800	3.33207300	0.65347300
H	-2.16778400	1.05599000	-2.96481700
H	-3.61334500	2.45724300	-1.49255700
H	1.12191600	0.27693700	3.01414800
H	-1.28128900	-0.10772800	3.18494100

Ph-4-1234-R2-Nu2

C	0.98104800	-0.43315700	-0.23829500
C	2.02497400	0.68057400	-0.31393800
C	1.67614000	-1.68073800	-0.77227900
C	3.30141800	0.38421400	0.16452900
C	2.93376900	-1.94590900	-0.35207500
C	3.72357100	-0.95227600	0.32369000
C	4.24277900	1.45512400	0.37653100
N	5.00082000	2.30941600	0.54968700
C	1.74003300	1.94314800	-0.89094000
N	1.52008400	2.95998900	-1.40633800
C	4.99786800	-1.26906400	0.86872600
N	6.03293000	-1.54246500	1.31590900
C	0.61341900	-0.71686300	1.18484400
N	0.37863500	-1.01243700	2.27569700
C	-0.29235500	-0.06333000	-1.02411000
Br	-1.43297700	-1.57093600	-1.42952700
S	-1.30778700	1.30216200	-0.23537000
H	-0.02198200	0.38516300	-1.98827100
O	-0.50239100	1.88492900	0.83313700
O	-1.75489100	2.10286900	-1.36972600
C	-2.75231500	0.58821500	0.52255400
C	-2.70117800	0.22356000	1.86592400
C	-3.92274400	0.50259400	-0.23100000
C	-3.86008300	-0.27400900	2.46106500
H	-1.77378500	0.32968300	2.42901500
C	-5.07142200	0.00725000	0.37902600
H	-3.92339500	0.82460600	-1.27284900
C	-5.03674900	-0.38410200	1.71979000
H	-3.84284300	-0.56827900	3.51080900

H	-5.99753000	-0.06939800	-0.19145900
H	-5.94003300	-0.77154700	2.19319400
H	1.12014300	-2.37592100	-1.39776400
H	3.40016200	-2.90144200	-0.59956600

Ph-4-1234-R2-Nu4

C	1.04254900	-0.66048200	-0.67834000
C	2.25534000	-0.65625900	0.31074400
C	0.16905100	0.58265000	-0.50790600
C	1.90178400	-0.28282700	1.73035600
H	2.70122600	-1.66616600	0.28340800
H	3.01359400	0.04669700	-0.06482800
C	0.19791700	-1.92100900	-0.55815100
C	-1.19142000	0.50359000	-0.23616700
C	0.88994800	-1.12732900	2.45641600
C	2.48714900	0.76941600	2.31166000
C	-1.12713900	-1.92329900	-0.32158200
C	-1.89200700	-0.71337900	-0.13213200
H	1.11170600	-2.19963000	2.34290800
H	0.86819500	-0.87762600	3.52556500
H	-0.11826500	-0.96783000	2.04251500
H	3.19693300	1.39682000	1.76732100
H	2.26325100	1.03960300	3.34665200
C	-1.91972500	1.73722600	-0.04622100
N	-2.50713800	2.71940300	0.10734800
C	0.82545200	1.83846300	-0.59659400
N	1.40556900	2.84060100	-0.68786700
C	-3.28067700	-0.75534500	0.14150200
N	-4.41971300	-0.80778100	0.36705100
C	1.63279300	-0.65855000	-2.04727600
N	2.08305200	-0.67040200	-3.11072400
H	0.73546900	-2.86334200	-0.68348700
H	-1.65058400	-2.87847700	-0.24857500

Ph-4-1234-R3

C	1.16415300	-1.48121600	0.14539000
C	0.27165400	-2.55054100	0.15890500
C	0.68746800	-0.16465700	0.07293700
C	2.62999600	-1.80156300	0.17749900
C	-1.09763500	-2.31682800	0.08212500
C	1.64197900	0.99046800	0.21261300
C	-0.68735400	0.07065600	-0.01788200
O	3.32995100	-0.99669900	-0.61372200
O	3.10098900	-2.71220300	0.80484500
C	-1.57999500	-1.01038100	-0.00126000
O	1.46137100	1.90288300	-0.73479400
O	2.44633600	1.08100800	1.09937000
C	-1.18935200	1.48309900	-0.19187800
C	4.74468700	-1.16176900	-0.58883000
C	-3.05590800	-0.74783900	0.04168300
C	2.25158400	3.08394400	-0.61975400

O	-0.89798700	2.23350200	0.86500800
O	-1.73667900	1.87882700	-1.18176100
H	5.14939300	-0.43834900	-1.30162200
H	5.12720000	-0.96179300	0.42063800
H	5.01379800	-2.18416400	-0.88205700
O	-3.76526800	-1.78597100	-0.38259800
O	-3.53984000	0.27676000	0.44742400
H	1.96566000	3.72584100	-1.45729100
H	3.31808300	2.83153100	-0.67309700
H	2.04763800	3.58085200	0.33811200
C	-1.26581900	3.60656800	0.76430200
C	-5.18246400	-1.64381600	-0.32099100
H	-2.35288800	3.69719100	0.64470500
H	-0.77041300	4.06735600	-0.10081800
H	-0.94001300	4.08076900	1.69398500
H	-5.50456100	-0.79715500	-0.94012500
H	-5.50183700	-1.47300800	0.71498900
H	-5.59969500	-2.57933200	-0.70260200
H	0.66340600	-3.56592500	0.22503400
H	-1.80473100	-3.14540400	0.10082200

Ph-4-1234-R3-Nu1

C	-1.01800200	-0.59893500	0.45452300
C	-0.35700600	-1.85230000	1.01287800
C	0.02343700	0.52059500	0.37746300
C	-2.13203000	-0.22421700	1.44243800
C	0.96950200	-2.06183000	0.94331700
C	-0.51419600	1.86804500	0.36362100
C	1.37985400	0.23194100	0.26100200
O	-1.62432800	0.11203900	2.63246700
O	-3.31770700	-0.27926000	1.24244100
C	1.89114800	-1.06727600	0.44377800
O	0.37656600	2.84745400	0.11353100
O	-1.69037400	2.14235700	0.55782500
C	2.38872100	1.32771600	0.01515500
C	-2.56096000	0.56387300	3.59399600
C	3.28355400	-1.37219400	0.19908200
C	-0.14620100	4.15892700	0.02844700
O	2.52087000	1.58326400	-1.28729700
O	3.01499800	1.88782400	0.87399900
H	-1.99056400	0.79524800	4.49892700
H	-3.07207400	1.46525900	3.22730300
H	-3.31222200	-0.20908800	3.80480000
O	3.60630700	-2.65336200	0.52386200
O	4.13366300	-0.62531000	-0.25863900
H	0.70420500	4.81841100	-0.17376700
H	-0.63518500	4.44942300	0.96793700
H	-0.88222500	4.22887000	-0.78476400
C	3.52750900	2.52326700	-1.61434000
C	4.95269500	-3.02177500	0.30819300
H	4.50276500	2.16751500	-1.25494600
H	3.31247900	3.49802900	-1.15443200

H	3.53151100	2.61184900	-2.70527400
H	5.63347800	-2.40917500	0.91605800
H	5.23323600	-2.90026200	-0.74758000
H	5.03811300	-4.07429100	0.59956900
C	-1.65459500	-0.89007600	-0.98370800
C	-2.27049700	0.38778500	-1.60633200
C	-2.68006100	-2.03058700	-0.97965700
C	-0.53821500	-1.35197400	-1.94128300
O	-1.58061200	1.09365600	-2.30742700
C	-3.71599100	0.75907900	-1.36301900
O	-2.87699400	-2.76169000	-0.04017800
C	-3.38227200	-2.31037400	-2.30099100
H	0.23454500	-0.57650300	-1.99074100
H	-0.09202400	-2.28939400	-1.58601500
H	-0.93189900	-1.49849200	-2.95637500
H	-3.71823900	1.46290400	-0.52040000
H	-4.09645900	1.26507700	-2.25994400
H	-4.34797400	-0.09013000	-1.08367700
H	-3.36792800	-1.46389400	-2.99927300
H	-2.87200500	-3.15934500	-2.78062100
H	-4.41648100	-2.61413100	-2.09550800
H	-1.01480500	-2.60718900	1.43994700
H	1.38226600	-3.00459500	1.30672200

Ph-4-1234-R3-Nu3

C	-0.55691700	-1.26618700	0.24877600
C	0.45006300	-2.40062300	0.29685200
C	0.11978500	0.04848500	0.60552900
C	-1.64193300	-1.62809200	1.26954000
C	1.77040600	-2.18546300	0.14597700
C	-0.75464100	1.08465700	1.14449400
C	1.47874700	0.22116600	0.36897200
O	-1.15568800	-1.61903500	2.51136900
O	-2.76177400	-1.98937800	1.01288300
C	2.33007100	-0.85839800	0.05237800
O	-0.21697700	2.32132000	1.16593700
O	-1.89002100	0.89887800	1.54537100
C	2.11997900	1.58014100	0.50676100
C	-2.10528100	-1.83265400	3.54109200
C	3.71772700	-0.64056600	-0.29595800
C	-1.04252400	3.34868200	1.68099900
O	2.01746000	2.26825000	-0.63374800
O	2.66516200	1.99481500	1.49280900
H	-1.55312400	-1.79840300	4.48529900
H	-2.86541000	-1.03911400	3.51580300
H	-2.60001800	-2.80578300	3.42330000
O	4.40839500	-1.79879300	-0.46265800
O	4.27396000	0.43371700	-0.45413800
H	-0.45991500	4.27376700	1.61839000
H	-1.31707300	3.14639300	2.72532100
H	-1.96369100	3.43545200	1.08754600
C	2.67375000	3.52332700	-0.64035700

C	5.77104100	-1.65776200	-0.80942100
H	3.74678100	3.38768700	-0.44780700
H	2.25334200	4.18348300	0.13107200
H	2.51634700	3.95372100	-1.63435800
H	6.32293000	-1.11257800	-0.03066200
H	5.88418100	-1.11144600	-1.75637800
H	6.17256500	-2.67158600	-0.91208000
C	-1.21289700	-1.17614600	-1.22314400
C	-2.20572800	-0.01820600	-1.36024800
C	-1.87667200	-2.45891700	-1.53022000
C	-0.09684300	-1.03902800	-2.28242000
C	-1.73097900	1.27391300	-1.62787600
C	-3.58512700	-0.20456900	-1.21586700
N	-2.31996700	-3.47895500	-1.84094600
H	0.52220500	-1.94284900	-2.30707500
H	-0.54741800	-0.88195800	-3.27181100
H	0.55113200	-0.18774100	-2.04350000
C	-2.61043600	2.35314500	-1.71079800
H	-0.66218700	1.45570100	-1.74049000
C	-4.46486400	0.87194700	-1.31223700
H	-3.96828600	-1.20236300	-1.00366400
C	-3.98148700	2.15825800	-1.55070900
H	-2.21679100	3.35256900	-1.90573000
H	-5.53660500	0.70287800	-1.19437700
H	-4.67035200	3.00186800	-1.61987400
H	0.05921700	-3.41432200	0.41452500
H	2.45636400	-3.03352700	0.11791600

Ph-4-1234-R3-Nu2

C	0.01101500	-1.05252900	0.81878400
C	1.40929200	-0.72506500	0.32039000
C	-0.21315000	-0.23815700	2.08844000
C	-0.19746200	-2.53503200	1.10477400
C	2.21899000	-1.79228600	-0.24073400
C	1.78605200	0.61773300	0.31489300
C	0.23461100	1.03219700	2.13025600
O	0.59743700	-2.95721600	2.08495500
O	-1.02321300	-3.22692300	0.56996800
O	3.48817500	-1.41763000	-0.51746100
O	1.85429000	-2.93755200	-0.43654500
C	1.10511900	1.56335700	1.10833600
C	2.92395900	1.06673800	-0.56654400
C	0.56859300	-4.35259000	2.34033000
C	4.29194700	-2.37554800	-1.17871800
C	1.35161400	2.99699000	1.11513300
O	4.00756400	1.39459500	0.13747600
O	2.85953600	1.13972300	-1.76472700
H	1.25500900	-4.52952900	3.17391000
H	-0.44574400	-4.67980200	2.60353400
H	0.90385200	-4.89797500	1.44748500
H	5.26627700	-1.90305100	-1.34134900
H	3.84732200	-2.66334300	-2.14158700

H	4.40851400	-3.27848600	-0.56432300
O	2.06830200	3.43552000	0.05463900
O	0.95031100	3.77731500	1.95844300
C	5.09321300	1.89466400	-0.62225200
C	2.37840100	4.81504300	0.03982700
H	5.42853500	1.14861000	-1.35590000
H	4.79486500	2.80591900	-1.15957700
H	5.89610200	2.11723500	0.08734200
H	2.99469600	5.08890900	0.90775300
H	1.46295600	5.42144900	0.05976100
H	2.93134300	4.99912800	-0.88733800
C	-1.02348600	-0.57199100	-0.23830100
Br	-0.69532200	-1.16069000	-2.05271800
S	-2.79762100	-0.90105900	0.19679300
H	-0.94526200	0.52145200	-0.27947200
O	-3.41490100	-1.82242900	-0.74915400
O	-2.87968100	-1.17680900	1.63105400
C	-3.52267000	0.70796100	-0.08079100
C	-4.18877500	0.94778800	-1.27921400
C	-3.38836000	1.68456400	0.90532500
C	-4.73608100	2.21260000	-1.49576000
H	-4.27661700	0.14948500	-2.01708700
C	-3.93769300	2.94368700	0.67438200
H	-2.85887200	1.45270600	1.83180400
C	-4.60901600	3.20472700	-0.52286300
H	-5.26448400	2.42229800	-2.42649900
H	-3.84341200	3.72303800	1.43136700
H	-5.03764000	4.19256700	-0.69797000
H	-0.80580700	-0.67269800	2.89099700
H	-0.01263700	1.68536600	2.97025500

Ph-4-1234-R3-Nu4

C	-1.34564000	-1.14262000	0.04972700
C	-1.63806500	-1.34777400	1.57294800
C	-0.43495600	0.05188600	-0.17576400
C	-2.11514500	-0.14457700	2.35388900
H	-2.35746200	-2.17671300	1.66644100
H	-0.67963000	-1.68014400	1.99747400
C	-0.66086200	-2.39838400	-0.47084800
C	-2.65142000	-1.03794700	-0.73014100
C	-1.10467700	1.33192900	-0.29111500
C	0.94338600	-0.10889400	-0.08628000
C	-3.59018200	0.15074700	2.38287800
C	-1.23301600	0.63591500	2.99094000
C	0.67950400	-2.50203900	-0.46679200
O	-2.45087200	-0.69757000	-2.00761100
O	-3.74082900	-1.33992700	-0.30883500
O	-0.28682100	2.38738100	-0.50793200
O	-2.31390900	1.49366400	-0.23167900
C	1.54238500	-1.38909600	-0.13323900
C	1.82388900	1.10390700	0.08360500
H	-3.92888100	0.44502300	1.38050100

H	-4.16452000	-0.74399700	2.66937700
H	-3.81555900	0.96092900	3.09046300
H	-0.15892800	0.43762800	2.94193600
H	-1.55922700	1.51511900	3.55398600
C	-3.61829000	-0.52371300	-2.78949700
C	-0.90187000	3.65986600	-0.51100000
C	2.95894500	-1.67111800	-0.02764000
O	2.40006600	1.47099900	-1.06224500
O	2.00401600	1.67079800	1.12880700
H	-3.28234100	-0.26159700	-3.79772300
H	-4.21617700	-1.44472200	-2.81511100
H	-4.23358200	0.28690400	-2.37379600
H	-0.10378800	4.39059100	-0.68031200
H	-1.39592600	3.85861100	0.45076600
H	-1.65419400	3.73118500	-1.30824600
O	3.71828600	-0.60696400	0.33959200
O	3.48250900	-2.75594300	-0.22360100
C	3.27779300	2.57732000	-0.96832300
C	5.10950900	-0.83518500	0.42205300
H	2.74356900	3.46468200	-0.60074500
H	4.10342000	2.35358700	-0.27790800
H	3.66430700	2.75765000	-1.97636000
H	5.52260500	-1.12148000	-0.55578700
H	5.33556900	-1.63656300	1.13878400
H	5.55981900	0.10544600	0.75747000
H	-1.29151600	-3.24273600	-0.76219100
H	1.16516300	-3.44024000	-0.74338100

Ph-4-1234-R4

C	0.00000000	1.38355300	-0.52618600
C	0.00000000	0.69525100	0.68651800
C	0.00000000	0.69758700	-1.73060000
C	0.00000000	-0.69525100	0.68651800
C	0.00000000	-0.69758700	-1.73060000
C	0.00000000	-1.38355300	-0.52618600
F	0.00000000	2.71102200	-0.49186700
H	0.00000000	-1.26636900	-2.65963500
F	0.00000000	1.35396200	1.83422600
H	0.00000000	1.26636900	-2.65963500
F	0.00000000	-1.35396200	1.83422600
F	0.00000000	-2.71102200	-0.49186700

Ph-4-1234-R4-Nu1

C	0.11002100	-0.37754400	-0.37965700
C	0.75693900	-1.65202800	-0.01106900
C	1.07580900	0.70970500	-0.21821100
C	2.12121400	-1.79759100	0.19133900
C	2.42867700	0.54936000	-0.02919600
C	2.98899400	-0.71328600	0.18259700
F	0.62415200	1.97924600	-0.44532600
F	3.21656500	1.63708600	-0.02324000

F	4.32684100	-0.83700300	0.39156000
F	-0.31760500	-0.37532000	-1.79912500
C	-1.27674700	-0.12713200	0.34089000
C	-2.32074700	-1.20061300	-0.02789000
C	-1.90427100	1.20140900	-0.11909600
C	-1.07704600	-0.17796400	1.86141900
O	-2.09528500	-2.15676400	-0.72406100
C	-3.68320100	-1.05502400	0.63165600
O	-2.53273900	1.24487800	-1.15190400
C	-1.83188500	2.40819400	0.78938200
H	-0.78320900	-1.19411300	2.15968700
H	-0.26147900	0.49545700	2.15802700
H	-1.98586800	0.10609000	2.41064800
H	-3.65626500	-1.60022000	1.58787900
H	-3.96020700	-0.01442400	0.84070100
H	-4.43923400	-1.52618400	-0.00749300
H	-2.62661800	2.31297600	1.54675300
H	-0.87256700	2.48860200	1.30949400
H	-2.02327700	3.30930200	0.19527700
H	0.12159900	-2.53478300	0.02410100
H	2.53520600	-2.78861900	0.39482500

Ph-4-1234-R4-Nu3

C	0.50555300	1.01381400	-0.02471400
C	0.75717800	0.74048200	-1.43468900
C	1.33291800	0.16536600	0.80496700
C	1.85321600	0.02797300	-1.89811200
C	2.42398900	-0.53934400	0.34094900
C	2.71704700	-0.61256600	-1.02103300
F	1.11008200	0.12432500	2.14971000
F	3.19043900	-1.19711500	1.22278000
F	3.79463200	-1.31937300	-1.44118600
F	0.87527500	2.50269200	0.29088800
C	-1.02726900	1.11873300	0.34886000
C	-1.74014800	-0.21528400	0.11424800
C	-1.59280000	2.15262800	-0.54099400
C	-1.28227000	1.62742600	1.78667100
C	-1.43986400	-1.29948100	0.95251800
C	-2.67957100	-0.40102000	-0.90365200
N	-2.06990000	2.96172500	-1.21219400
H	-0.69924800	2.53989800	1.94946400
H	-2.35219200	1.84134400	1.91717000
H	-0.98141000	0.87549600	2.52137200
C	-2.06680500	-2.53018000	0.77646100
H	-0.70348700	-1.17962000	1.74813900
C	-3.30854200	-1.63564600	-1.08091900
H	-2.92905500	0.42583400	-1.57018100
C	-3.00641400	-2.70449200	-0.24173000
H	-1.81768000	-3.36018600	1.43968600
H	-4.03968900	-1.75604800	-1.88203000
H	-3.49771400	-3.66908600	-0.37844100
H	0.07051800	1.17990500	-2.16111400

H	2.02810600	-0.07483100	-2.97142200

Ph-4-1234-R4-Nu2

C	1.46694200	-0.05678800	-0.43899200
C	1.94900900	-1.44162100	-0.51692400
C	2.42657600	0.74761000	0.31448100
C	3.26968100	-1.79817400	-0.28286600
C	3.73407700	0.38363200	0.54026700
C	4.19251400	-0.89899300	0.22928900
F	2.03070300	1.97334000	0.74801900
F	4.55876400	1.26172200	1.13001900
F	5.48786900	-1.22806900	0.46051900
F	1.29784000	0.48559900	-1.81789600
C	0.02791000	0.13529500	0.14130300
Br	-0.81522900	1.81135400	-0.39358000
S	-1.10431300	-1.24866300	-0.28352500
H	0.05501500	0.13743900	1.23692600
O	-1.09057700	-1.44086600	-1.73057500
O	-0.76325700	-2.35546200	0.61089700
C	-2.73973700	-0.70934100	0.18212200
C	-3.61414200	-0.26757400	-0.80750800
C	-3.11396500	-0.79472800	1.52130400
C	-4.90095900	0.11613300	-0.43625800
H	-3.27938200	-0.22947400	-1.84471200
C	-4.40122900	-0.39850600	1.88024800
H	-2.41145400	-1.17616700	2.26383600
C	-5.28933100	0.05596500	0.90378500
H	-5.60275400	0.46416900	-1.19498300
H	-4.71404400	-0.45343000	2.92349500
H	-6.29701800	0.36136400	1.18926500
H	3.59358400	-2.82400600	-0.47319800
H	1.26809400	-2.19886200	-0.89894700

Ph-4-1234-R4-Nu4

C	-0.43738100	-0.27658800	0.87351300
C	-1.96030400	-0.08458400	0.80302200
C	0.33024400	0.79960400	0.27494000
C	-2.53868800	-0.04327100	-0.59146900
H	-2.41181100	-0.90876700	1.37804900
H	-2.18955000	0.85117600	1.32901200
C	0.09916100	-1.57112100	0.46900000
C	1.60430500	0.64211600	-0.21745500
C	-2.71786800	-1.35910500	-1.29798100
C	-2.87103700	1.11753700	-1.16655000
C	1.38682600	-1.73064100	-0.03149000
C	2.16815900	-0.63250700	-0.36063800
H	-3.33919300	-2.03964900	-0.69364200
H	-3.18944400	-1.22989100	-2.28151400
H	-1.74285400	-1.85201700	-1.43016300
H	-2.71996100	2.06539400	-0.64484300
H	-3.28501400	1.15295100	-2.17786000

F	3.42621100	-0.75470700	-0.85988600
F	2.29616100	1.72089300	-0.62249600
F	-0.18345800	2.05791400	0.33196900
F	-0.29262100	-0.13785500	2.43257000
H	1.79032500	-2.72871900	-0.22022600
H	-0.50963200	-2.45532600	0.67243100

Ph-4-1234-R5

C	-1.37364600	-0.95391500	0.00151500
C	-0.69650200	-2.16266600	0.00328800
C	-0.69289900	0.26144700	0.00343400
C	0.69661200	-2.16264100	-0.00350300
C	0.69294200	0.26149100	-0.00350300
C	1.37373600	-0.95384800	-0.00161400
N	1.41299900	1.55403700	-0.04265200
O	1.15635900	2.33099800	0.83680200
O	2.16830300	1.71025100	-0.96063800
N	2.84833000	-0.98523000	0.09247800
O	3.36712900	-0.10334100	0.72716100
O	3.40251700	-1.90751300	-0.44231500
N	-2.84825700	-0.98535700	-0.09235200
O	-3.36718100	-0.10346400	-0.72696900
O	-3.40233100	-1.90767100	0.44243600
N	-1.41308300	1.55399200	0.04262700
O	-2.16804200	1.71024300	0.96087900
O	-1.15694300	2.33075500	-0.83712300
H	-1.26546200	-3.09170800	0.00232300
H	1.26560300	-3.09166200	-0.00258300

Ph-4-1234-R5-Nu1

C	-0.70467500	0.33344200	0.63089100
C	-0.12938200	1.66389600	1.08759200
C	0.44034600	-0.64139200	0.48781900
C	1.14531000	2.02223900	0.87327700
C	1.72411200	-0.20914500	0.15297400
C	2.08507100	1.12103400	0.25622700
N	2.70117900	-1.19226600	-0.38067900
O	2.47804800	-1.60141400	-1.49186900
O	3.63145300	-1.48409300	0.31489300
N	3.30859200	1.64060800	-0.26165200
O	3.97639000	0.94310200	-1.00561800
O	3.62512400	2.77260900	0.05747600
N	-1.57315100	-0.14194700	1.84109500
O	-0.97686400	-0.29709600	2.87712900
O	-2.75186100	-0.29581100	1.69025400
N	0.17612100	-2.01827500	0.64614600
O	-0.99531000	-2.36018400	0.77253700
O	1.09920500	-2.81624200	0.65487300
C	-1.61472600	0.46916900	-0.64994900
C	-2.78976700	1.45076600	-0.43873200
C	-2.15946300	-0.89113300	-1.16562300

C	-0.75525400	1.10625800	-1.76845500
O	-2.83096800	2.20885400	0.49883700
C	-3.85839200	1.51405100	-1.50779500
O	-3.30959600	-1.20054400	-0.98265600
C	-1.26469900	-1.74510600	-2.03854100
H	-0.48255300	2.13569600	-1.50224800
H	0.16819700	0.53424400	-1.93074700
H	-1.30828200	1.12828400	-2.71667200
H	-4.19336500	2.55383500	-1.60164400
H	-3.53459600	1.12026200	-2.47798500
H	-4.69848600	0.89539800	-1.16450900
H	-1.38404000	-1.39592300	-3.07664100
H	-0.19620600	-1.68250900	-1.79889500
H	-1.61362800	-2.78188800	-1.97884400
H	-0.82367000	2.34749800	1.56854000
H	1.50160100	3.01035000	1.16030700

Ph-4-1234-R5-Nu3

C	-1.40365000	-0.00692000	0.49752000
C	-0.84163600	0.56438400	1.78307300
C	-0.54285800	-1.16084400	0.05956400
C	0.46549600	0.48934500	2.07328500
C	0.82336800	-1.14815100	0.32364000
C	1.36834300	-0.25993200	1.23700900
N	1.71572100	-2.09172900	-0.40029800
O	1.84512700	-1.91931000	-1.58327600
O	2.23989200	-2.94718900	0.25820500
N	2.76225500	0.02700500	1.29937200
O	3.50103100	-0.44687200	0.45302600
O	3.15431500	0.75257800	2.19699100
N	-2.78887200	-0.56414200	0.93252400
O	-2.76841800	-1.51567700	1.66644200
O	-3.77870500	0.02605200	0.58842300
N	-1.13562800	-2.24036800	-0.64116300
O	-2.28995400	-2.11146500	-1.02967000
O	-0.48948600	-3.25926900	-0.81686800
C	-1.57134300	1.13913900	-0.60123500
C	-0.15283400	1.66686800	-0.90201400
C	-2.35568400	2.24389500	-0.01091100
C	-2.27669500	0.69064300	-1.89494600
C	0.65192800	1.00886900	-1.83912000
C	0.39950200	2.72724400	-0.17438400
N	-2.93899400	3.13612600	0.43008900
H	-3.30896700	0.39122900	-1.69234000
H	-2.26469300	1.52629400	-2.60741600
H	-1.75795800	-0.16272000	-2.34155700
C	1.98964400	1.36365800	-2.00297100
H	0.26431300	0.16812700	-2.41251300
C	1.73552200	3.08833400	-0.34244000
H	-0.20999600	3.26221300	0.55508500
C	2.53957600	2.39810200	-1.24820300
H	2.60628000	0.80875200	-2.71090900

H	2.15001400	3.90639800	0.24832700
H	3.59118800	2.66423400	-1.36416300
H	-1.53432100	1.11029900	2.42615500
H	0.88028100	0.99834200	2.94227700

Ph-4-1234-R5-Nu2

C	-0.66587400	-1.21964900	0.41252900
C	-1.64912600	-0.62108900	-0.55620700
C	-1.26404200	-1.18172500	1.80402200
C	-2.49555800	0.40054600	-0.14912200
C	-2.15582400	-0.24414800	2.16143500
C	-2.69849600	0.67561200	1.19974300
N	-3.45530700	1.79101000	1.65958300
O	-3.72863400	1.84562200	2.84613000
O	-3.78533100	2.64875300	0.85821600
N	-3.22600100	1.19818600	-1.16834200
O	-4.39172300	0.95124400	-1.31113100
O	-2.58822000	2.03301900	-1.74849000
N	-1.58747000	-1.03973000	-1.91350000
O	-2.40650400	-0.61595900	-2.70550300
O	-0.71117500	-1.83394800	-2.21417600
N	-0.47733400	-2.72467400	0.09980300
O	-1.48797600	-3.37767400	0.07384500
O	0.63652600	-3.14382200	-0.03659200
C	0.69329200	-0.46265900	0.43530000
Br	1.92638700	-1.08131300	1.79550700
S	1.59816500	-0.15572300	-1.14924300
H	0.42525400	0.55982700	0.73650900
O	2.28374900	-1.34878900	-1.61436900
O	0.65549100	0.56722400	-1.99984900
C	2.83178300	1.02492000	-0.63496700
C	4.13759700	0.59067100	-0.41897000
C	2.46027300	2.36281900	-0.51139800
C	5.09741900	1.53014100	-0.04842500
H	4.38458000	-0.46414300	-0.54477100
C	3.42968800	3.28991500	-0.13344800
H	1.43458700	2.66942600	-0.72391200
C	4.74170500	2.87255200	0.09801400
H	6.12650700	1.21379700	0.12474500
H	3.16166300	4.34175800	-0.02976100
H	5.49743400	3.60320400	0.38976400
H	-0.88217400	-1.90453600	2.52455300
H	-2.50589300	-0.16815300	3.18955400

Ph-4-1234-R5-Nu4

C	1.25889200	-0.83091700	-0.13897100
C	2.39486400	-0.61415000	0.89759300
C	0.36346600	0.35305300	-0.34603900
C	1.93502300	0.22093700	2.07005100
H	2.71521600	-1.60789600	1.24731700
H	3.25311800	-0.13911200	0.41517800

C	0.46311700	-2.06807400	0.16383700
C	-1.01513100	0.28901600	-0.22110500
C	0.85706800	-0.34992700	2.95209800
C	2.46960500	1.42640300	2.29012000
C	-0.87738000	-2.09329200	0.24565600
C	-1.67103000	-0.90837000	0.07247900
H	1.09777900	-1.38187700	3.25124600
H	0.72109700	0.26030600	3.85446400
H	-0.10699100	-0.38966200	2.41925300
H	3.22648900	1.83871800	1.61858300
H	2.15405100	2.03753900	3.13928700
N	-3.08331500	-1.01302600	0.19531600
O	-3.55575900	-2.10314300	0.47699100
O	-3.77268200	-0.02158300	0.01551500
N	-1.81688400	1.53058900	-0.36882300
O	-2.17216200	1.81630000	-1.47862500
O	-2.04817700	2.13535800	0.64208300
N	1.02543300	1.56733700	-0.69149900
O	0.40988300	2.61744300	-0.70249100
O	2.21162000	1.49194800	-0.97794200
N	1.96152000	-1.16222700	-1.51096700
O	1.37950900	-0.86681300	-2.52022700
O	3.00008400	-1.77320000	-1.45775900
H	1.04662100	-2.97740000	0.31865800
H	-1.40416300	-3.01787000	0.47314400

Ph-4-1235-R1

C	0.11573900	1.21022800	-0.00316600
C	-1.27797100	1.20433700	-0.01493200
C	0.82950700	-0.00011000	0.05392600
C	-1.97008100	0.00225200	0.00999800
C	0.11375800	-1.20944900	-0.00297800
C	-1.27987300	-1.20115600	-0.01524000
C	-3.47894000	0.00194700	0.06391400
C	0.77231500	-2.58079500	-0.10714400
C	2.35383600	-0.00166600	0.16563400
C	0.77630400	2.58045100	-0.10718400
F	1.80889900	2.56013400	-0.94723300
F	-0.09187800	3.47474200	-0.58100800
F	1.19614200	3.03793700	1.06735500
F	2.78753200	1.06088800	0.83676700
F	2.93511300	-0.00174300	-1.02775100
F	2.78541200	-1.06580600	0.83577800
F	-0.09741100	-3.47399300	-0.58074300
F	1.80456700	-2.56222000	-0.94737900
F	1.19180300	-3.03887000	1.06725700
F	-3.91243500	-0.03388800	1.32401300
F	-3.98534200	1.09610500	-0.50016300
F	-3.98648600	-1.05775700	-0.56170000
H	-1.82358700	2.14586900	-0.06179500
H	-1.82720800	-2.14183900	-0.06192800

Ph-4-1235-R1-Nu1

C	-0.59056500	0.32154900	0.40916800
C	0.30275800	1.54209000	0.51513300
C	0.30317900	-0.92911000	0.28774900
C	1.59790100	1.55175200	0.12589700
C	1.64063900	-0.81835900	-0.13297800
C	2.30250600	0.38994800	-0.28914600
C	2.33927500	2.85463900	0.08887900
C	2.45442700	-2.03540300	-0.52634400
C	-0.26700300	-2.26122400	0.63619400
C	-1.43282600	0.29191400	1.74576400
F	-0.93905800	-0.60985800	2.60677400
F	-1.39984400	1.44406400	2.42339300
F	-2.71795700	-0.00818900	1.56689500
F	-1.61312100	-2.21191700	0.73750500
F	0.16251700	-2.79296000	1.80014600
F	-0.02285700	-3.23262200	-0.27385100
F	3.74830200	-1.74269100	-0.71246500
F	2.43299900	-3.01474700	0.38366900
F	2.03227400	-2.56010300	-1.68793700
F	2.54668800	3.25537800	-1.17799300
F	1.69837000	3.85269600	0.70345600
F	3.55139500	2.76527100	0.65255900
C	-1.54839900	0.53492300	-0.88724900
C	-2.45426000	1.77398000	-0.76404700
C	-2.49871200	-0.63611500	-1.21308000
C	-0.63031300	0.76495600	-2.10682200
O	-2.54841100	2.41859600	0.25054800
C	-3.18424500	2.21910000	-2.01621800
O	-3.66760500	-0.58152600	-0.90668300
C	-1.98189700	-1.77729200	-2.05774200
H	-0.13249600	1.74110300	-2.03467600
H	0.14852300	-0.00669500	-2.14652100
H	-1.19857300	0.73520900	-3.04633500
H	-2.50581900	2.85897600	-2.60105400
H	-3.49662200	1.38145400	-2.65060400
H	-4.05418500	2.81648500	-1.72026100
H	-2.17561400	-1.51987000	-3.11111000
H	-0.90736700	-1.94920300	-1.94456200
H	-2.54770200	-2.68378100	-1.81356200
H	-0.16708400	2.46211000	0.85231200
H	3.33290500	0.44466400	-0.62485100

Ph-4-1235-R1-Nu3

C	-0.52586600	-1.05766300	-0.21706800
C	0.76635500	-1.04792400	-0.99953700
C	-1.17117900	0.32057800	-0.31397900
C	1.45696900	0.08717300	-1.25433500
C	-0.36794200	1.45386100	-0.55958500
C	0.95238600	1.38150800	-0.97086000
C	2.81595000	0.00092700	-1.88353000
C	-0.87028700	2.86349600	-0.30830700

C	-2.63832600	0.48315400	-0.12200900
C	-1.39243900	-2.19666000	-0.86663300
F	-2.16936100	-1.71511400	-1.83809100
F	-0.63295300	-3.14360800	-1.43833600
F	-2.17748900	-2.86825300	-0.01783700
F	-3.24705900	-0.65090300	0.26506700
F	-3.30911600	0.87986700	-1.22463200
F	-2.96827600	1.39352800	0.82859000
F	-0.02678000	3.79171300	-0.77826700
F	-2.05141300	3.12762200	-0.87618100
F	-0.99909900	3.12120000	1.00283200
F	3.74707700	0.58799400	-1.11709900
F	3.22479200	-1.25400300	-2.09392800
F	2.86185900	0.62617200	-3.06912400
C	-0.18439000	-1.46884100	1.34754900
C	0.91812800	-0.53087200	1.85146200
C	0.32369400	-2.85031700	1.35065200
C	-1.39260500	-1.40372200	2.30242700
C	0.57764600	0.72419700	2.37103700
C	2.27329100	-0.87011600	1.76362200
N	0.70760700	-3.93954300	1.33596700
H	-2.13224300	-2.17669400	2.07926000
H	-1.03835100	-1.52516700	3.33513700
H	-1.88236300	-0.42693700	2.22347700
C	1.56572100	1.62185600	2.77064200
H	-0.46573100	1.03056800	2.42718400
C	3.26292800	0.02366300	2.16955000
H	2.56294300	-1.84185100	1.35934400
C	2.91279500	1.27689500	2.67045500
H	1.27491000	2.60205400	3.15216300
H	4.31290300	-0.26012700	2.08270000
H	3.68643900	1.98036200	2.98260500
H	1.20207200	-2.00705700	-1.26867700
H	1.55956100	2.26857400	-1.12120700

Ph-4-1235-R1-Nu2

C	-0.43920800	-0.11433300	0.76768400
C	-1.50905700	-0.92713700	0.03072600
C	-0.72198700	1.36909400	0.68114100
C	-2.62557300	-0.28596900	-0.54279800
C	-1.80555700	1.87992300	0.06119900
C	-2.80243900	1.08594800	-0.57027400
C	-3.74581400	-1.06576200	-1.21201800
C	-1.45034600	-2.41124200	0.12735900
C	-1.99216400	3.36907300	0.01496400
C	-0.43691900	-0.46590700	2.28269800
F	-1.68481100	-0.47461200	2.74862500
F	0.09421600	-1.65960100	2.55291000
F	0.23109900	0.44187200	3.00340600
F	-3.16000200	3.73554000	0.56227300
F	-1.03528200	4.04609600	0.65366000
F	-2.01276000	3.82220800	-1.24692200

F	-3.39074400	-1.57316100	-2.39753300
F	-4.19693400	-2.07484600	-0.45685300
F	-4.81522400	-0.29560100	-1.45689700
F	-2.13210300	-2.93175300	1.17777800
F	-1.92788600	-3.04842000	-0.95346700
F	-0.19378300	-2.89074600	0.27820200
C	1.01142300	-0.40109100	0.17034400
Br	1.03331300	0.13551000	-1.68652900
S	2.46007600	0.34004500	1.03743800
H	1.24878000	-1.46705900	0.19559400
O	2.30166800	1.78848100	1.14752900
O	2.66883400	-0.47437900	2.23237400
C	3.84494500	0.02871600	-0.04258800
C	4.33289800	1.06571700	-0.83424900
C	4.43168400	-1.23503500	-0.02582200
C	5.43648100	0.81872300	-1.64774000
H	3.84866600	2.04242800	-0.80459300
C	5.52996500	-1.47003700	-0.85105100
H	4.04095900	-2.01336700	0.63148200
C	6.02799900	-0.44624400	-1.65896800
H	5.83596400	1.61615300	-2.27523300
H	6.00324100	-2.45242400	-0.85609600
H	6.88996300	-0.63459900	-2.30057200
H	0.01198200	2.02783800	1.14000900
H	-3.67081000	1.54525000	-1.03061300

Ph-4-1235-R1-Nu4

C	-0.00062800	-1.17848300	-0.27472200
C	-0.24583300	-2.46337000	0.59385800
C	1.46516300	-0.82741700	-0.11380100
C	-0.04293600	-2.26245900	2.07619500
H	-1.24359100	-2.87269900	0.39920000
H	0.48352300	-3.20803400	0.24305600
C	-0.86938100	0.05596900	0.00811600
C	1.92377200	0.44232900	-0.09961700
C	-1.12394300	-1.55690100	2.84901500
C	1.06637700	-2.70848100	2.67732900
C	-0.29602100	1.34088600	-0.02280800
C	1.06767900	1.58072900	-0.11670900
H	-2.09494100	-2.05290500	2.70034400
H	-0.89270100	-1.53685100	3.92250200
H	-1.24208900	-0.52214300	2.49203100
H	1.84771100	-3.22622200	2.11510500
H	1.22601100	-2.56826000	3.74956400
C	3.39663800	0.69888900	0.01770500
C	-1.12818000	2.59591500	0.16565400
C	-2.34606500	-0.10701900	0.01289500
C	-0.19925400	-1.62079300	-1.73854000
F	0.02007700	-0.61950400	-2.59285500
F	0.63862300	-2.61242100	-2.08706100
F	-1.44006800	-2.07077600	-1.97555200
F	-2.74824900	-1.35358000	0.33128200

F	-2.94674900	0.14293200	-1.17914400
F	-2.98329600	0.69638200	0.89194200
F	-0.44253900	3.70758400	-0.13877700
F	-2.22466800	2.63359300	-0.60059400
F	-1.52563500	2.75314900	1.43606000
F	3.70744800	1.32122400	1.16506900
F	4.13667700	-0.41380900	-0.02592500
F	3.83856300	1.49566900	-0.96724100
H	2.16360500	-1.66146900	-0.04025500
H	1.47648900	2.58583500	-0.10381000

Ph-4-1235-R2

C	-0.22438600	1.21494900	-0.00007600
C	1.17137500	1.21601900	0.00002100
C	-0.93304000	0.00005000	-0.00015100
C	1.86113500	-0.00004900	-0.00006700
C	-0.22437600	-1.21488800	-0.00012300
C	1.17132300	-1.21614000	-0.00004000
C	3.30357600	-0.00016400	0.00004800
N	4.45699700	-0.00004100	0.00008600
C	-0.94165800	-2.46469300	-0.00004500
N	-1.51268200	-3.46670500	0.00011200
C	-2.37180500	-0.00003100	0.00001600
N	-3.52524400	-0.00003400	0.00009700
C	-0.94157400	2.46480900	-0.00000400
N	-1.51242000	3.46692300	0.00004800
H	1.71492300	-2.16029900	0.00002900
H	1.71509500	2.16011900	0.00009000

Ph-4-1235-R2-Nu1

C	0.19035500	-0.17780900	0.90616700
C	-1.13160800	-0.85084000	1.23883200
C	-0.14162200	1.24371400	0.47942400
C	-2.23350900	-0.59025300	0.47386400
C	-1.27450300	1.43925800	-0.32931000
C	-2.27820800	0.48421900	-0.46554300
C	-3.42589500	-1.37551200	0.68679500
N	-4.37810100	-2.01306400	0.83765700
C	-1.43869400	2.70221900	-1.00837000
N	-1.58634400	3.70570400	-1.56196300
C	0.66249100	2.34874200	0.85204900
N	1.27807900	3.28391300	1.16214500
C	0.97683300	-0.17681100	2.15988400
N	1.52155300	-0.24064800	3.17480400
C	1.03088500	-1.02144100	-0.17268700
C	2.43746800	-0.41756400	-0.34136000
C	0.37225500	-0.90546600	-1.55824500
C	1.16562300	-2.47824500	0.28858800
O	2.80136200	0.54147700	0.28940600
C	3.37928500	-1.13292700	-1.28519100
O	0.68196800	0.02197500	-2.27121500

C	-0.55612700	-1.99535000	-2.03201800
H	1.73547400	-2.53212200	1.22817700
H	0.17853900	-2.92634200	0.46203700
H	1.68553300	-3.08874800	-0.46194200
H	3.90288200	-1.91598800	-0.71523900
H	2.86149700	-1.60893500	-2.12683500
H	4.12463900	-0.41812500	-1.65204800
H	0.05711900	-2.85613800	-2.34499200
H	-1.23138300	-2.34200200	-1.24108700
H	-1.12231900	-1.63087400	-2.89626500
H	-3.14023400	0.64637900	-1.10685900
H	-1.17486600	-1.56165300	2.06374700

Ph-4-1235-R2-Nu3

C	0.99402800	0.93902500	0.16686400
C	1.04270200	-0.26093900	1.09415600
C	-0.09337800	1.87202600	0.65123300
C	-0.15213400	-0.71710700	1.68727900
C	-1.20355700	1.38255800	1.27992600
C	-1.31787000	0.03153400	1.72703500
C	-0.15801600	-2.01915800	2.31118900
N	-0.17997800	-3.06501000	2.80187700
C	2.25713600	-0.96163700	1.28908500
N	3.25557700	-1.53183800	1.46214100
C	-2.28190800	2.29809800	1.57275000
N	-3.15496300	3.02111400	1.79676800
C	2.28143900	1.66848200	0.21510200
N	3.27573500	2.25279600	0.24336400
C	0.76161400	0.53656800	-1.39946200
C	-0.50219500	-0.30846200	-1.52020800
C	1.92747900	-0.26051500	-1.81790800
C	0.72501900	1.80460700	-2.26616500
C	-1.74283800	0.29671000	-1.75058900
C	-0.45306400	-1.69624900	-1.33478700
N	2.85928000	-0.85622300	-2.14786900
H	1.69858700	2.31189700	-2.24656200
H	0.47949700	1.54765200	-3.30476100
H	-0.03418800	2.50193800	-1.89045300
C	-2.91102000	-0.46428500	-1.76225400
H	-1.81131100	1.37327100	-1.90530700
C	-1.61987100	-2.45711500	-1.34712200
H	0.50809100	-2.18631000	-1.16659300
C	-2.85471200	-1.84146200	-1.55228600
H	-3.87057200	0.02548600	-1.93472600
H	-1.56188000	-3.53547600	-1.19218700
H	-3.76985800	-2.43556500	-1.55865000
H	0.00822400	2.94001700	0.45929100
H	-2.22926500	-0.34485500	2.18295200

Ph-4-1235-R2-Nu2

C	-1.10533300	0.19693700	-0.02271600

C	-2.06359000	-0.97057900	-0.21357300
C	-1.95999600	1.41246500	-0.31524100
C	-3.28557600	-0.88336900	0.48397800
C	-3.17505500	1.48796900	0.31523800
C	-3.78481700	0.34374700	0.91407400
C	-4.08846200	-2.06756800	0.65667300
N	-4.74008000	-3.01157600	0.79851400
C	-1.76520900	-2.06049400	-1.06192400
N	-1.53347000	-2.93605000	-1.79068600
C	-3.92226600	2.71932800	0.25134800
N	-4.52369700	3.70562600	0.21083900
C	-0.64150100	0.29311800	1.40135000
N	-0.34392100	0.43234300	2.50788900
C	0.12023600	0.08950700	-0.94556900
Br	1.08183100	1.75396000	-1.15580600
S	1.31003000	-1.28012400	-0.47987500
H	-0.19183800	-0.21677300	-1.95186000
O	0.64510700	-2.12750200	0.50420400
O	1.74467900	-1.81307500	-1.76645600
C	2.73506800	-0.56332600	0.31272500
C	2.75607800	-0.45431600	1.70123500
C	3.82947400	-0.21743200	-0.47980100
C	3.90583300	0.04983500	2.30849900
H	1.89045000	-0.75789800	2.28992900
C	4.97040800	0.28104800	0.14210500
H	3.77889800	-0.34376400	-1.56173400
C	5.00463800	0.41820500	1.53203300
H	3.94332900	0.14672200	3.39389100
H	5.83694400	0.55910500	-0.45854700
H	5.90136900	0.80946600	2.01467600
H	-1.57982800	2.21890500	-0.93926500
H	-4.71358100	0.41938100	1.47366100

Ph-4-1235-R2-Nu4

C	-0.19368200	-0.81530700	0.76119200
C	-1.01388000	-1.89567700	-0.02064900
C	-0.65804500	0.60590300	0.44152500
C	-1.21418100	-1.58136800	-1.48346000
H	-0.48347300	-2.85830600	0.09099800
H	-1.99664100	-2.00647500	0.46113900
C	1.30023000	-0.94429600	0.51425100
C	0.23191900	1.59357300	-0.01365900
C	0.00852200	-1.37149000	-2.33552400
C	-2.44837700	-1.50101300	-1.99106000
C	2.08385600	0.08363600	0.08967900
C	1.59160500	1.39792200	-0.20295200
H	0.75235500	-2.16586300	-2.16818800
H	-0.25613500	-1.34942700	-3.40113400
H	0.49861200	-0.41784600	-2.08111900
H	-3.33167300	-1.61889600	-1.35893100
H	-2.60987100	-1.30184000	-3.05347500
C	-0.30456500	2.90187500	-0.31598100

N	-0.71878500	3.95206700	-0.56125800
C	-2.03842900	0.87197500	0.58658300
N	-3.18026600	1.04775200	0.72790200
C	3.49098900	-0.17993700	-0.11040500
N	4.61695800	-0.38115800	-0.27455700
C	-0.42149000	-1.10543600	2.21005500
N	-0.58185200	-1.31986400	3.33376100
H	1.74005300	-1.92549500	0.70230200
H	2.24754000	2.19041300	-0.55010500

Ph-4-1235-R3

C	-0.39325700	-1.31012400	0.10063600
C	-0.73666600	0.04978000	0.13439400
C	0.94655500	-1.69272600	0.04472500
C	-1.40179900	-2.42159200	0.10996700
C	-2.17850900	0.48049000	0.29713100
C	0.28449300	1.01294100	0.09277100
C	1.95418500	-0.73315900	-0.00510400
O	-2.60776900	-2.02049000	-0.27204000
O	-1.13388000	-3.55766400	0.40033100
O	-2.69479100	0.88799700	-0.85122500
O	-2.75992600	0.44214900	1.34497400
C	1.62207600	0.62030000	0.01241300
C	-0.06365100	2.47036300	0.15948000
C	3.37362800	-1.20239100	-0.07879000
C	-3.63891100	-3.00385000	-0.25696000
C	-4.01696700	1.40784000	-0.77532900
O	0.98197000	3.25066700	-0.08657100
O	-1.16204100	2.89300300	0.41588200
O	4.24043800	-0.19549300	-0.10977300
O	3.69326100	-2.36242900	-0.10724000
H	-4.54771400	-2.49696000	-0.59220400
H	-3.38420200	-3.82831700	-0.93454100
H	-3.76989400	-3.39856000	0.75850100
H	-4.29690600	1.68900900	-1.79431900
H	-4.03197400	2.28555700	-0.11551500
H	-4.70499300	0.64757900	-0.38292300
C	0.74705000	4.65475200	-0.02136600
C	5.61705600	-0.55469200	-0.18090500
H	-0.00911200	4.94697700	-0.76095600
H	0.39598900	4.93459900	0.97996600
H	1.70447000	5.13434300	-0.24107200
H	5.81198900	-1.13369400	-1.09266600
H	5.89792400	-1.15734100	0.69225600
H	6.17777100	0.38367400	-0.19552400
H	2.40412500	1.37654800	-0.02604000
H	1.20272800	-2.75292500	0.03195500

Ph-4-1235-R3-Nu1

C	0.64838600	-0.59217100	0.23375100
C	0.03025100	0.79674200	0.28205700

C	-0.48490500	-1.58850700	0.44815800
C	1.61631600	-0.80022700	1.41136400
C	0.83196400	1.86005300	0.78504900
C	-1.29800900	1.00070500	-0.17926300
C	-1.77852100	-1.32205000	0.13869900
O	1.04661600	-0.47500600	2.57700600
O	2.72130700	-1.26945000	1.34463200
O	0.15385900	3.02618700	0.99827400
O	2.03345400	1.79610300	1.03944800
C	-2.20625500	-0.02103900	-0.30944500
C	-1.70715500	2.33467800	-0.72208800
C	-2.76062500	-2.42665300	0.34213700
C	1.89499800	-0.53575200	3.70921400
C	0.94717200	4.15887600	1.27530600
O	-2.87551400	2.76213600	-0.23223700
O	-1.10740600	2.93948600	-1.57872100
O	-4.00119900	-2.08419200	-0.03358700
O	-2.50833000	-3.52140300	0.78524000
H	1.28425500	-0.25159700	4.57193100
H	2.29894400	-1.54794800	3.84433600
H	2.73074700	0.16778900	3.58727300
H	0.26034100	5.00819700	1.36534200
H	1.66775800	4.34730400	0.46558600
H	1.50848600	4.03220400	2.21156700
C	-3.36061900	3.98369000	-0.76766500
C	-4.99492700	-3.08430500	0.12246400
H	-2.64785500	4.79710900	-0.57561200
H	-3.51222400	3.89990800	-1.85225600
H	-4.31157100	4.18633300	-0.26561900
H	-5.08011000	-3.38378700	1.17562300
H	-4.74733500	-3.97247600	-0.47453800
H	-5.93479600	-2.64399100	-0.22402600
C	1.46388100	-0.88026400	-1.11285100
C	2.08689600	-2.28779900	-1.13195400
C	2.54714600	0.20171200	-1.33749400
C	0.48518000	-0.87405900	-2.30569300
O	1.60938600	-3.20376100	-0.50366500
C	3.24547300	-2.55486300	-2.07051000
O	3.70405300	0.00714300	-1.04672800
C	2.15384800	1.48013100	-2.04586200
H	-0.25034100	-1.68083400	-2.18843000
H	-0.05528900	0.08002600	-2.35149700
H	1.01671300	-1.02231100	-3.25772900
H	3.23172500	-3.61550200	-2.34740400
H	3.22469700	-1.91674100	-2.96346800
H	4.17186800	-2.32291800	-1.52886200
H	2.13209500	1.27126900	-3.12821000
H	1.16034000	1.84937800	-1.76261800
H	2.92629300	2.23267700	-1.85011600
H	-3.21084900	0.14353800	-0.69059300
H	-0.23668700	-2.56896300	0.84976000

Ph-4-1235-R3-Nu3

C	0.21451200	-0.90679400	0.20541200
C	1.70557600	-1.18773800	0.18124600
C	0.00009200	0.56300700	0.49080500
C	-0.35363200	-1.78227500	1.33289600
C	2.62626700	-0.22975900	-0.09840300
C	-1.15371800	0.93691600	1.25332800
C	0.96895800	1.50164100	0.05132600
O	0.13477300	-1.41978500	2.52032400
O	-1.06227400	-2.74488000	1.19056400
C	2.25706600	1.14768800	-0.27943600
C	4.05493200	-0.66053400	-0.16426300
O	-1.21201300	2.26764600	1.55611400
O	-2.02742200	0.18199100	1.66381800
C	0.61662400	2.93943100	-0.17145900
C	-0.41969700	-2.08812500	3.63976300
O	4.87777600	0.34345200	-0.49032700
O	4.45777500	-1.77963900	0.04227000
C	-2.38626300	2.69760900	2.20982300
O	-0.44403300	3.06532600	-0.98603400
O	1.23807500	3.89099100	0.22807300
H	0.08782800	-1.68845100	4.52319400
H	-1.49802400	-1.88316300	3.69549100
H	-0.26321600	-3.17257200	3.56740700
C	6.25611700	0.01606900	-0.57488100
H	-2.28516200	3.77899800	2.35559200
H	-2.50490500	2.19892700	3.18195800
H	-3.27803800	2.48125500	1.60318300
C	-0.89782600	4.38977700	-1.20345500
H	6.62303200	-0.35977700	0.38957200
H	6.42374200	-0.75526400	-1.33850700
H	6.77699900	0.93918700	-0.84598600
H	-0.11582600	5.00180700	-1.67285300
H	-1.18344400	4.85430200	-0.24907700
H	-1.76805600	4.31638200	-1.86347900
C	-0.46503200	-1.35847900	-1.19067000
C	-1.97065400	-1.09016700	-1.21989900
C	-0.18643100	-2.79036500	-1.41491900
C	0.22306700	-0.62078000	-2.35908500
C	-2.43663000	0.20853300	-1.47057700
C	-2.90992100	-2.10578500	-1.00771800
N	0.10250900	-3.88292400	-1.65358400
H	1.28290900	-0.89740600	-2.41800200
H	-0.27030000	-0.88521200	-3.30449100
H	0.15662700	0.46293500	-2.21045000
C	-3.80385400	0.48458900	-1.47424600
H	-1.73186600	1.02413900	-1.63051800
C	-4.27630300	-1.83075900	-1.02132600
H	-2.56372300	-3.12018400	-0.81018400
C	-4.73037000	-0.53139100	-1.24626300
H	-4.14433700	1.50562600	-1.65798000
H	-4.98964900	-2.63901300	-0.85073700
H	-5.80004400	-0.31458000	-1.25166100

H	2.98011500	1.88431600	-0.62072500
H	2.04925800	-2.20644400	0.37692700

Ph-4-1235-R3-Nu2

C	0.83049700	-0.29450600	0.90679200
C	2.03584500	-0.94848400	0.26006800
C	0.85074900	1.14686900	0.43373700
C	0.90037900	-0.43250100	2.42228800
C	2.30450100	-0.69790000	-1.05123700
C	0.74434900	2.22959400	1.37854300
C	1.05640300	1.34763700	-0.95439900
O	2.10884300	-0.13672400	2.89281700
O	-0.00714800	-0.82290000	3.10833200
C	1.66921400	0.38836300	-1.74027300
C	3.37088100	-1.50898100	-1.70145800
O	0.96251200	3.45061100	0.81345800
O	0.53882500	2.14182900	2.57763000
C	0.64065000	2.60323600	-1.65729800
C	2.20039300	-0.02957100	4.30466200
O	3.51718800	-1.21995500	-3.00111600
O	4.04297000	-2.34838800	-1.15145400
C	0.78606300	4.56306200	1.66447900
O	-0.62085000	2.96373900	-1.37799500
O	1.31411000	3.20356600	-2.45702200
H	3.23907100	0.23083700	4.52994400
H	1.52140800	0.76283900	4.64746400
H	1.93024400	-0.97771800	4.78777700
C	4.51253500	-1.95624700	-3.69382000
H	0.98277700	5.45417200	1.05813500
H	1.48358600	4.52581200	2.51285000
H	-0.23794200	4.59753400	2.06316600
C	-1.05084900	4.19321600	-1.93616100
H	5.50206500	-1.77860000	-3.25179500
H	4.29767400	-3.03245100	-3.65123900
H	4.49180200	-1.60532400	-4.72994400
H	-1.00851500	4.16337600	-3.03336900
H	-0.41218300	5.01428500	-1.58105200
H	-2.08150000	4.34293900	-1.59882700
C	-0.44843300	-1.01121700	0.38305900
Br	-0.36675300	-2.94925800	0.49143200
S	-2.07785000	-0.46348600	1.06700000
H	-0.51359800	-0.78873500	-0.68969100
O	-2.64861400	-1.45920900	1.96611400
O	-1.96376800	0.92348100	1.50227500
C	-3.06744900	-0.48343400	-0.42361000
C	-3.96199600	-1.52918700	-0.62947100
C	-2.90893700	0.55503700	-1.34144400
C	-4.72501900	-1.53451800	-1.79767300
H	-4.05355200	-2.31440700	0.12168500
C	-3.67518400	0.53344800	-2.50514100
H	-2.19342700	1.35833100	-1.14424000
C	-4.58047600	-0.50713200	-2.73055200

H	-5.43471500	-2.34285700	-1.97819200
H	-3.56709400	1.33384700	-3.23858200
H	-5.17861200	-0.51641800	-3.64298100
H	2.64164700	-1.65873900	0.82267000
H	1.81555400	0.54197700	-2.80673800

Ph-4-1235-R3-Nu4

C	0.30340900	-1.09917800	0.22134100
C	0.13299400	-1.43655700	1.74438500
C	-0.55748300	0.08386000	-0.17618000
C	-1.28581900	-1.54227100	2.25148400
H	0.69591200	-2.35956600	1.95409000
H	0.62686200	-0.60486000	2.26838200
C	1.75931200	-0.74004400	-0.01938700
C	0.02421400	-2.33629000	-0.62515400
C	-1.90655800	-0.20645100	-0.54719100
C	-0.07042300	1.36826700	0.14104500
C	-1.97551000	-2.87766900	2.18066600
C	-1.91148500	-0.45366600	2.71811600
C	2.18826500	0.52993800	0.20402400
O	0.06726800	-2.07070700	-1.93495500
O	-0.10893900	-3.45837700	-0.20213100
O	-2.69262200	0.89425800	-0.73237000
O	-2.38381100	-1.32715200	-0.70288600
C	1.26090300	1.60521900	0.43036000
C	-0.97958900	2.55976700	0.20770800
H	-2.13427800	-3.15230100	1.12893400
H	-1.35611200	-3.66840800	2.63207500
H	-2.94634800	-2.84832100	2.69508900
H	-1.42029700	0.52351300	2.72303500
H	-2.94470000	-0.50444300	3.07419200
C	3.63650200	0.87340700	0.15972100
C	-0.27556700	-3.14476300	-2.79052500
C	-4.05397600	0.64223900	-1.00090500
O	-1.03570400	3.22899700	-0.94774500
O	-1.54546700	2.93429300	1.20407700
O	4.42746400	-0.16557900	-0.14559700
O	4.08734900	1.97190800	0.38375800
H	-0.19586500	-2.76558400	-3.81442600
H	0.40244100	-3.99718400	-2.64726300
H	-1.30534400	-3.47131700	-2.58716300
H	-4.54148700	1.61930300	-1.09346400
H	-4.51855100	0.06867800	-0.18594000
H	-4.17715100	0.07395200	-1.93365600
C	-1.92251000	4.33471100	-0.97172400
C	5.81820700	0.10623600	-0.19275600
H	-2.94481400	4.00695400	-0.73631800
H	-1.62121200	5.09583400	-0.23916700
H	-1.87986100	4.74686600	-1.98467700
H	6.17677800	0.46478000	0.78174600
H	6.03926300	0.87143300	-0.94921300
H	6.30921700	-0.83636100	-0.45329300

H	2.46895100	-1.52656100	-0.28185900
H	1.63297900	2.60192500	0.66151300

Ph-4-1235-R4

C	0.00000000	1.19801100	-0.38315700
C	0.00000000	1.21940700	1.00469400
C	0.00000000	0.00000000	-1.09297600
C	0.00000000	0.00000000	1.67104700
C	0.00000000	-1.19801100	-0.38315700
C	0.00000000	-1.21940700	1.00469400
F	0.00000000	0.00000000	-2.41881300
F	0.00000000	-2.33139800	-1.06983100
F	0.00000000	0.00000000	3.00076900
H	0.00000000	-2.16405400	1.54624900
H	0.00000000	2.16405400	1.54624900
F	0.00000000	2.33139800	-1.06983100

Ph-4-1235-R4-Nu1

C	0.22903500	-0.21173400	-0.43563800
C	1.01250400	-1.42882000	-0.13265800
C	1.07478200	0.97556500	-0.22956100
C	2.35694300	-1.38982000	0.14510300
C	2.41592300	0.94456300	0.03735500
C	3.13878500	-0.23749000	0.25683000
F	0.49102800	2.18283100	-0.49402300
F	3.06509900	2.12293800	0.10203000
F	2.97777400	-2.57380900	0.35952200
F	-0.20275500	-0.18550900	-1.82703000
C	-1.15848500	-0.15229700	0.32551300
C	-2.09394800	-1.30130700	-0.09754400
C	-1.91282900	1.13771400	-0.03364100
C	-0.91939500	-0.28302900	1.83572400
O	-1.77370500	-2.19395600	-0.83999000
C	-3.45907100	-1.32920300	0.57008200
O	-2.59195800	1.17816600	-1.03442100
C	-1.88003300	2.30169300	0.92989700
H	-0.55113400	-1.29242100	2.06619600
H	-0.14543400	0.42786700	2.15627400
H	-1.83353900	-0.09717500	2.41720100
H	-3.37455200	-1.93736800	1.48402400
H	-3.82968200	-0.33636700	0.85198000
H	-4.17274500	-1.82202500	-0.10093700
H	-2.62348500	2.11212000	1.72072700
H	-0.90088300	2.42525100	1.40393900
H	-2.16449900	3.21410700	0.39313500
H	0.51556600	-2.39533900	-0.17596900
H	4.20013300	-0.24538100	0.48544700

Ph-4-1235-R4-Nu3

C	-0.75509300	0.88861000	-0.21607900

C	-0.99991200	0.75224100	1.22430200
C	-1.40610300	-0.20012600	-0.94013800
C	-1.92475300	-0.12680500	1.73362600
C	-2.31166500	-1.06030600	-0.37475700
C	-2.63545300	-1.06766700	0.98753900
F	-1.15631400	-0.33126900	-2.27678700
F	-2.89878000	-1.95902500	-1.18542200
F	-2.13470600	-0.10721200	3.06778500
F	-1.31772500	2.18931700	-0.70829300
C	0.77411300	1.14237700	-0.56342500
C	1.63379600	-0.05497500	-0.15624300
C	1.17060800	2.33443100	0.21178600
C	1.01334500	1.51371600	-2.04500300
C	1.50585000	-1.25502100	-0.87156700
C	2.54467200	-0.00407500	0.90258500
N	1.50084000	3.27462000	0.79459100
H	0.33959400	2.33047400	-2.32665100
H	2.05554200	1.83430100	-2.18091700
H	0.81994800	0.65517300	-2.69389100
C	2.27226000	-2.36816500	-0.53587900
H	0.79599800	-1.31949100	-1.69687800
C	3.31405700	-1.12012800	1.23895700
H	2.66039000	0.91690000	1.47601900
C	3.18152400	-2.30614100	0.52176000
H	2.15595300	-3.29225500	-1.10451300
H	4.01932200	-1.05611000	2.06920000
H	3.78159800	-3.17891100	0.78392000
H	-0.46706300	1.40525000	1.91708500
H	-3.36291100	-1.75176800	1.41415900

Ph-4-1235-R4-Nu2

C	1.58047900	0.26441800	-0.38379500
C	2.26936600	-1.02244500	-0.21251800
C	2.36584200	1.34178400	0.24765000
C	3.53365700	-1.15036000	0.29794900
C	3.62537400	1.13738700	0.75989300
C	4.27793900	-0.09134000	0.83749700
F	4.26853800	2.21446400	1.25928000
F	4.07969300	-2.37848000	0.28255100
F	1.69459700	-2.10355800	-0.79653700
F	1.39924000	0.49229700	-1.80834500
C	0.10053800	0.30763700	0.13408700
Br	-0.79392500	1.93556000	-0.45440000
S	-0.95874500	-1.14197300	-0.26849900
H	0.10196200	0.32964500	1.22945000
O	-0.97707700	-1.36482800	-1.70903300
O	-0.55783500	-2.19656400	0.66162000
C	-2.60692400	-0.65365400	0.22162300
C	-3.52032200	-0.26869900	-0.75660400
C	-2.94786000	-0.71431300	1.57118500
C	-4.81112400	0.08011400	-0.36464100
H	-3.21213200	-0.24679400	-1.80249600

C	-4.23939900	-0.35310600	1.95132500
H	-2.21502900	-1.05110700	2.30603200
C	-5.16617100	0.04307100	0.98539000
H	-5.54239900	0.38238400	-1.11519400
H	-4.52533900	-0.39045000	3.00309300
H	-6.17717100	0.32073900	1.28708800
H	1.95644900	2.35142900	0.25384300
H	5.27326200	-0.22318500	1.25070100

Ph-4-1235-R4-Nu4

C	-0.27892800	0.06660500	0.89796300
C	-1.80556100	-0.11870100	0.84103000
C	0.19858900	1.37371900	0.43298100
C	-2.43594600	0.09856700	-0.51085800
H	-2.02209100	-1.14141100	1.19054100
H	-2.23947000	0.57832200	1.57100000
C	0.49230400	-1.00953200	0.27408600
C	1.42545200	1.51370600	-0.17239000
C	-1.92729600	-0.73992000	-1.65265600
C	-3.41514500	0.99488800	-0.67847000
C	1.70183100	-0.81534800	-0.33209100
C	2.25115800	0.45794000	-0.56498900
H	-1.83483400	-1.79442100	-1.35263300
H	-2.58383200	-0.66395500	-2.52990500
H	-0.91528100	-0.41126500	-1.93556800
H	-3.76675600	1.61079500	0.15326900
H	-3.89286100	1.14015100	-1.65100200
F	-0.01133700	-2.27452800	0.36169500
F	2.37587300	-1.89871500	-0.76962300
F	1.84931300	2.76803100	-0.45793000
F	-0.08059400	-0.05130100	2.39381200
H	3.22555100	0.59752400	-1.02448500
H	-0.40846900	2.26133300	0.61957000

Ph-4-1235-R5

C	0.00000000	1.19657800	-0.14952700
C	0.01137200	1.21588000	1.23773500
C	0.00000000	0.00000000	-0.86364300
C	0.00000000	0.00000000	1.90133100
C	0.00000000	-1.19657800	-0.14952700
C	-0.01137200	-1.21588000	1.23773500
N	0.05651600	-2.50072000	-0.84613500
O	0.50757600	-2.50219700	-1.96247300
O	-0.33372200	-3.45717100	-0.23247100
N	0.00000000	0.00000000	3.38224100
O	-0.00229900	-1.07325900	3.92689900
O	0.00229900	1.07325900	3.92689900
N	-0.05651600	2.50072000	-0.84613500
O	-0.50757600	2.50219700	-1.96247300
O	0.33372200	3.45717100	-0.23247100
N	0.00000000	0.00000000	-2.34800800

O	1.02766800	0.32298800	-2.87177500
O	-1.02766800	-0.32298800	-2.87177500
H	0.02006500	2.16106700	1.77937300
H	-0.02006500	-2.16106700	1.77937300

Ph-4-1235-R5-Nu1

C	-0.37373200	0.30569500	0.41416100
C	0.51570700	1.53429300	0.36822500
C	0.51971600	-0.90275800	0.42896700
C	1.79893700	1.48246400	-0.03065700
C	1.82044600	-0.85755000	-0.13247700
C	2.49773300	0.28643800	-0.40361900
N	2.42565400	-2.09496900	-0.66936100
O	1.73632300	-2.77209700	-1.39473300
O	3.58392700	-2.30864800	-0.41588100
N	2.55530800	2.75361500	-0.08110200
O	3.70317800	2.69058600	-0.45411600
O	1.99488200	3.77253000	0.24212900
N	-1.06155500	0.41104900	1.82674500
O	-0.30900200	0.42858700	2.76734600
O	-2.25489400	0.49575800	1.89019800
N	0.08295000	-2.06233500	1.04341200
O	-1.09463700	-2.11239900	1.41897900
O	0.86217200	-2.99907100	1.20858000
C	-1.48856500	0.31423200	-0.69836800
C	-2.38811900	1.56652500	-0.62883900
C	-2.35891600	-0.97496900	-0.69201600
C	-0.76244800	0.41353300	-2.06148400
O	-2.06706500	2.54470400	0.00253500
C	-3.64593800	1.58765900	-1.46648200
O	-3.48421900	-0.95335000	-0.26115800
C	-1.82249300	-2.20698300	-1.38556900
H	-0.25007800	1.38090100	-2.15400600
H	-0.01664900	-0.38700300	-2.16180500
H	-1.47529200	0.32239400	-2.89183600
H	-3.82109100	2.61589900	-1.80398000
H	-3.61476400	0.89667100	-2.31696000
H	-4.47517700	1.27325800	-0.81860700
H	-2.05137000	-2.11000000	-2.45924200
H	-0.73770800	-2.33949500	-1.29424600
H	-2.35487500	-3.08050200	-0.99406500
H	0.08069500	2.48632400	0.65928800
H	3.46051700	0.30576900	-0.90448400

Ph-4-1235-R5-Nu3

C	0.92149300	-0.71068400	0.26694100
C	-0.17803100	-1.11810600	1.21782700
C	1.08441000	0.77842700	0.30874700
C	-1.12374400	-0.24682600	1.61133100
C	-0.01388300	1.59820200	0.66534700
C	-1.14109500	1.14521300	1.27585500

N	-0.06470500	3.00377200	0.20399300
O	0.23278600	3.22457000	-0.94381300
O	-0.46564500	3.82923100	0.98765700
N	-2.23521400	-0.76481000	2.43883300
O	-3.06990600	0.03163000	2.79567300
O	-2.25312800	-1.94319500	2.70108400
N	2.19583200	-1.37033600	0.90686500
O	2.51655700	-0.94663800	1.98417200
O	2.72081000	-2.28836400	0.33593200
N	2.34240900	1.34502100	0.13251300
O	3.26465700	0.62756400	-0.26560400
O	2.50987500	2.53099500	0.40412400
C	0.65325100	-1.31765700	-1.18130300
C	-0.68323800	-0.71285600	-1.66037800
C	0.47758600	-2.78019600	-1.07177200
C	1.77867200	-1.03423800	-2.19287100
C	-0.69460400	0.53672000	-2.28966400
C	-1.90490100	-1.35122400	-1.41434800
N	0.29967300	-3.91821400	-1.00551100
H	2.69546600	-1.55814000	-1.90874700
H	1.45016900	-1.36425200	-3.18753900
H	1.99941600	0.03702300	-2.22401600
C	-1.89880400	1.14214500	-2.64598100
H	0.23491000	1.07278700	-2.47259700
C	-3.10921900	-0.74984500	-1.77701800
H	-1.92274900	-2.32653000	-0.92457300
C	-3.11063300	0.50252100	-2.39025700
H	-1.88242800	2.12539100	-3.11833600
H	-4.04963600	-1.26425800	-1.57435800
H	-4.05284900	0.97706000	-2.66848900
H	-0.22633000	-2.15811700	1.54382300
H	-2.00354600	1.77507200	1.46945900

Ph-4-1235-R5-Nu2

C	-0.91243000	0.34146800	0.74599600
C	-1.65370500	-0.91358700	0.40922200
C	-1.68543900	1.52092700	0.20240000
C	-2.49594200	-0.96517200	-0.71991000
C	-2.56306400	1.37865300	-0.81048500
C	-2.95096700	0.13277200	-1.38884100
N	-3.20473000	2.60562800	-1.33837800
O	-2.94835500	3.65939000	-0.80906200
O	-3.94722600	2.47247700	-2.28121800
N	-2.83101500	-2.26655500	-1.34088100
O	-3.99672500	-2.48769800	-1.56003100
O	-1.91267500	-2.97999800	-1.65100900
N	-1.50903400	-2.01952100	1.24947800
O	-2.22292000	-3.00006700	1.08323900
O	-0.68135600	-1.94348000	2.15443800
N	-0.92853600	0.55386300	2.29663600
O	-2.01947600	0.56128300	2.80406600
O	0.11176900	0.74499700	2.85740800

C	0.54687100	0.39192300	0.20293100
Br	1.43879500	2.09402600	0.47640200
S	1.70770900	-1.00816200	0.55760700
H	0.43258900	0.30475300	-0.88623000
O	2.20568800	-0.96848800	1.92125500
O	1.06587400	-2.19702200	0.00617300
C	3.07135200	-0.61901100	-0.52728700
C	4.22057600	-0.04278800	0.00899900
C	2.96697600	-0.95523600	-1.87590300
C	5.29151900	0.22233400	-0.84182900
H	4.26321600	0.18520900	1.07463000
C	4.04344500	-0.67804000	-2.71662800
H	2.06460100	-1.44063200	-2.25175700
C	5.19931500	-0.08990400	-2.19973800
H	6.20164200	0.67183500	-0.44336600
H	3.98333100	-0.93218600	-3.77532400
H	6.04073600	0.12079600	-2.86134300
H	-1.49790400	2.50626200	0.62619000
H	-3.56396600	0.07254800	-2.28221600

Ph-4-1235-R5-Nu4

C	-0.08950200	-1.11428700	-0.22335600
C	-0.48905100	-2.09303000	0.90532000
C	1.39213100	-0.87399600	-0.22167500
C	-0.46535700	-1.41701400	2.25769400
H	-1.48715400	-2.49053100	0.68791100
H	0.22339300	-2.92801600	0.88918700
C	-0.83143300	0.17768600	-0.32082900
C	1.92509900	0.36298400	-0.18304000
C	-1.70475100	-0.66980600	2.67073100
C	0.62061700	-1.48676900	3.03501500
C	-0.17808500	1.41871400	-0.18461600
C	1.17477200	1.57795900	-0.11850700
H	-2.59727500	-1.30103600	2.54256300
H	-1.64310200	-0.35139000	3.71992800
H	-1.85085800	0.22885900	2.05096600
H	1.51006200	-2.03752100	2.71760800
H	0.65190200	-0.99718400	4.01145900
N	-0.95985500	2.64339500	0.08751800
O	-1.69805200	2.62594900	1.04127000
O	-0.75027800	3.60269800	-0.61221500
N	3.40218100	0.47556600	-0.16594100
O	3.86799700	1.58876100	-0.11103600
O	4.05656300	-0.53841300	-0.20701700
N	-0.33297500	-1.90542000	-1.59321400
O	-0.45778800	-1.24838300	-2.59132300
O	-0.29338400	-3.10871400	-1.55170600
N	-2.17593600	0.11291000	-0.67500700
O	-2.71558000	-0.99714500	-0.69785700
O	-2.77986200	1.13800400	-0.98039100
H	2.04637700	-1.74567000	-0.25235800
H	1.64918500	2.54063700	0.04102400

Ph-4-1245-R1

C	1.21368800	-0.69994200	0.00265100
C	1.21372600	0.69992500	-0.00253900
C	0.00003300	-1.38220800	0.00004100
C	0.00005100	1.38223600	0.00005400
C	-1.21363700	-0.69993000	-0.00256800
C	-1.21362100	0.69995900	0.00256900
C	2.48491000	1.53125600	0.02913600
C	-2.48486500	1.53120100	-0.02915200
C	-2.48485600	-1.53118200	0.02913500
C	2.48485300	-1.53127400	-0.02915500
F	3.01440500	-1.56266000	-1.24895900
F	3.40431900	-1.05521000	0.80620700
F	2.23690200	-2.79025200	0.32661800
F	-3.40421600	-1.05497100	-0.80622300
F	-2.23702200	-2.79018900	-0.32660000
F	-3.01454100	-1.56250600	1.24891100
F	-3.01458800	1.56244000	-1.24890900
F	-2.23706200	2.79023000	0.32654200
F	-3.40417100	1.05500300	0.80627400
F	3.40441500	1.05495200	-0.80599900
F	2.23697600	2.79013700	-0.32696900
F	3.01438100	1.56299400	1.24898800
H	0.00004100	-2.47082300	0.00001700
H	0.00007900	2.47085100	0.00001400

Ph-4-1245-R1-Nu1

C	0.80104800	-0.19152900	0.41042200
C	-0.44851000	-1.01516300	0.65546700
C	0.35252800	1.25432700	0.19341900
C	-1.70319600	-0.63611300	0.30633000
C	-0.93894400	1.57954000	-0.15237000
C	-2.00265100	0.67135900	-0.20455900
C	-2.77670500	-1.68820500	0.41862200
C	-3.35024400	1.15417000	-0.56558100
C	1.27825000	2.40760900	0.35199400
C	1.68885600	-0.30380700	1.70396300
F	1.56770200	0.79665200	2.46290700
F	1.33752100	-1.30734800	2.51096500
F	2.98844400	-0.44370000	1.44733000
F	2.57260400	2.03196400	0.37007200
F	1.09101300	3.13739400	1.47086600
F	1.16187400	3.31618200	-0.64496000
F	-4.16442500	1.43694000	0.48032100
F	-4.04964200	0.27945500	-1.31765500
F	-3.29254600	2.29842800	-1.27386200
F	-3.07071200	-2.21500300	-0.78047600
F	-2.42332000	-2.71464800	1.19978800
F	-3.91987100	-1.20778300	0.92053000
C	1.55457500	-0.81367000	-0.88136500

C	2.04867900	-2.25231500	-0.64480400
C	2.78675700	-0.01878800	-1.36481100
C	0.53299100	-0.88004000	-2.03700700
O	1.97276400	-2.80108500	0.42606800
C	2.56154000	-3.01017400	-1.85316900
O	3.90302500	-0.38637300	-1.08125700
C	2.57960600	1.13317500	-2.32005700
H	-0.21598000	-1.65949800	-1.84422300
H	0.00234400	0.07544300	-2.13189700
H	1.02597600	-1.10336400	-2.99268600
H	1.70039400	-3.48016100	-2.35248500
H	3.07138000	-2.36763400	-2.58008000
H	3.23504300	-3.80333300	-1.50887700
H	2.59795500	0.72259700	-3.34190600
H	1.62171300	1.64333100	-2.18074400
H	3.41355500	1.83622200	-2.21339800
H	-0.28770700	-2.01083400	1.05971400
H	-1.14624400	2.62782600	-0.38331400

Ph-4-1245-R1-Nu3

C	-1.30186000	-0.32054700	-0.40321500
C	-0.07705200	-0.91079700	-1.06912400
C	-1.05225600	1.16466500	-0.22494100
C	1.16000800	-0.35631900	-1.01711100
C	0.22964700	1.67104800	-0.17315900
C	1.38598000	0.94794200	-0.47285800
C	2.29853900	-1.18838600	-1.55395600
C	2.70385800	1.60735300	-0.35898100
C	-2.11573000	2.16088400	0.05738600
C	-2.49451200	-0.62025300	-1.36728700
F	-2.59636300	0.32210800	-2.30532400
F	-2.33524300	-1.78241500	-2.01745700
F	-3.68899900	-0.72069000	-0.77512000
F	-3.35933500	1.69002100	-0.10335400
F	-2.02327500	3.26327500	-0.71123700
F	-2.07955600	2.65117500	1.33111700
F	3.18118000	2.14587500	-1.50638200
F	3.67701200	0.77964800	0.06679400
F	2.66854300	2.63335300	0.51272400
F	3.00373800	-1.76861900	-0.57429500
F	1.88178900	-2.18026700	-2.34975900
F	3.16092800	-0.46156400	-2.27463100
C	-1.54114900	-1.08327500	1.02818700
C	-0.24422500	-0.97573200	1.84008700
C	-1.81410700	-2.50347600	0.74520800
C	-2.72381000	-0.54575500	1.86282900
C	-0.00501500	0.16319500	2.62011500
C	0.74167000	-1.96632400	1.78620300
N	-2.05074700	-3.61048000	0.51790200
H	-3.68815500	-0.78635900	1.40874000
H	-2.67914900	-0.99161400	2.86570400
H	-2.65515700	0.54075500	1.97520100

C	1.19569000	0.31266900	3.31137600
H	-0.74445500	0.96200000	2.66378300
C	1.94287300	-1.81898700	2.47810800
H	0.57892700	-2.86052900	1.18186500
C	2.17653900	-0.67608300	3.24038400
H	1.36678900	1.21366600	3.90263700
H	2.70239900	-2.59897700	2.40828200
H	3.11878800	-0.55616800	3.77747000
H	-0.19156400	-1.89086500	-1.52578000
H	0.34251100	2.72343200	0.10362200

Ph-4-1245-R1-Nu2

C	0.25543900	0.89762900	0.59166100
C	1.11633000	1.56466200	-0.47037600
C	0.97097100	-0.31541300	1.13663500
C	2.24734100	0.95851700	-0.96952400
C	2.09207600	-0.84955000	0.59470300
C	2.76734500	-0.25989800	-0.52603300
C	0.74378400	2.89609500	-1.01389100
C	4.04201700	-0.74574100	-1.09479400
C	2.57043200	-2.15157400	1.19085300
C	0.00845800	1.86106100	1.77748200
F	1.14308600	2.44552500	2.15338000
F	-0.86204200	2.82837600	1.47364100
F	-0.46204600	1.22068800	2.85227200
F	3.89350900	-2.16135600	1.39276300
F	2.00631200	-2.41934800	2.37304900
F	2.29019900	-3.19107800	0.39562200
F	5.13724700	-0.48954400	-0.33923600
F	4.30408000	-0.17647200	-2.28619300
F	4.07076600	-2.07763300	-1.30014300
F	0.95518700	3.92399800	-0.16175800
F	1.42564500	3.20997800	-2.12442300
F	-0.56791400	2.99871100	-1.34497700
C	-1.15654000	0.49581200	-0.02924700
Br	-0.87219800	-0.77052200	-1.46237700
S	-2.42769200	-0.17893500	1.12157500
H	-1.64753700	1.37197200	-0.46587700
O	-1.87998600	-1.32834000	1.83724500
O	-2.96317500	0.96524100	1.85533100
C	-3.73910500	-0.76463000	0.06495200
C	-3.85380400	-2.13068400	-0.18061600
C	-4.64236800	0.16365300	-0.44864000
C	-4.90213100	-2.57594900	-0.98295500
H	-3.12894600	-2.82015300	0.25377500
C	-5.68107000	-0.29528900	-1.25662400
H	-4.53774400	1.22269200	-0.20783100
C	-5.80771900	-1.65995700	-1.52242000
H	-5.01222300	-3.64121100	-1.18854600
H	-6.39806300	0.41347900	-1.67216200
H	-6.62432000	-2.01437200	-2.15302900
H	0.48938400	-0.81271000	1.97518300

H	2.77973600	1.47724000	-1.76711100

Ph-4-1245-R1-Nu4

C	-1.18201100	-0.74800200	0.00208100
C	-1.73880200	-1.16100900	1.41279100
C	0.19703600	-1.34466400	-0.18392800
C	-0.98204700	-0.55226800	2.56866500
H	-2.80111700	-0.88509300	1.47310800
H	-1.67320700	-2.25855500	1.47099400
C	-1.10251000	0.75432800	-0.23799300
C	1.34710900	-0.63099500	-0.20952200
C	-1.45541700	0.78390700	3.07535600
C	0.06290600	-1.18357300	3.11524500
C	0.09968900	1.41997100	-0.25578800
C	1.35910200	0.80698900	-0.18620800
H	-2.51915100	0.73529500	3.35725300
H	-0.87270300	1.10778200	3.94818000
H	-1.37081400	1.55030900	2.29122000
H	0.39579700	-2.15494600	2.74129000
H	0.62418000	-0.74073900	3.94202400
C	2.63325000	-1.41660900	-0.20228400
C	2.56973000	1.63962100	-0.30056800
C	-2.34612100	1.55116100	-0.36154000
C	-2.09914200	-1.43749200	-1.02249300
F	-1.77599100	-1.13303500	-2.27899200
F	-2.03104700	-2.77669900	-0.92037800
F	-3.39154400	-1.12375400	-0.85986200
F	-3.29167500	1.22020300	0.54955200
F	-2.96893300	1.42681800	-1.55792600
F	-2.13975800	2.87091800	-0.20878600
F	3.11851400	1.69973100	-1.54210100
F	3.58341800	1.23691600	0.49439500
F	2.32652300	2.92464500	0.02463100
F	3.22627300	-1.38973100	0.99922000
F	2.45620100	-2.71027400	-0.49990700
F	3.52417900	-0.94759300	-1.08481300
H	0.24443300	-2.43322700	-0.20706500
H	0.07050700	2.50767900	-0.33518000

Ph-4-1245-R2

C	0.00000000	1.20655400	0.70466700
C	0.00000000	1.20655400	-0.70466700
C	0.00000000	0.00000000	1.40554900
C	0.00000000	0.00000000	-1.40554900
C	0.00000000	-1.20655400	0.70466700
C	0.00000000	-1.20655400	-0.70466700
C	0.00000000	2.45199300	-1.42885100
N	0.00000000	3.44869600	-2.00935500
C	0.00000000	-2.45199300	-1.42885100
N	0.00000000	-3.44869600	-2.00935500
C	0.00000000	-2.45199300	1.42885100

N	0.00000000	-3.44869600	2.00935500
C	0.00000000	2.45199300	1.42885100
N	0.00000000	3.44869600	2.00935500
H	0.00000000	0.00000000	-2.49460500
H	0.00000000	0.00000000	2.49460500

Ph-4-1245-R2-Nu1

C	0.48797800	0.45045500	0.75669300
C	-0.81379300	0.14812800	1.48372800
C	0.17769200	1.47368700	-0.33458500
C	-1.98841400	0.12662900	0.80100300
C	-1.02284900	1.40328100	-1.00831900
C	-2.09487900	0.61043900	-0.55447200
C	-3.17773400	-0.32023900	1.48577300
N	-4.12579700	-0.69665200	2.02839100
C	-3.30009300	0.49082800	-1.29140500
N	-4.28400800	0.38099600	-1.89919900
C	1.10007200	2.49892700	-0.68954500
N	1.79684900	3.37704100	-0.98875000
C	1.40455300	1.02179900	1.76873500
N	2.05336700	1.44549200	2.62327700
C	1.16053800	-0.90066800	0.21412800
C	2.56419300	-0.61260800	-0.34993700
C	0.34386100	-1.44752100	-0.97280700
C	1.28943800	-1.92460600	1.35026600
O	3.04815800	0.48959100	-0.32034400
C	3.35058500	-1.79546100	-0.87060200
O	0.61298800	-1.05990900	-2.08633800
C	-0.69589600	-2.51282800	-0.73521800
H	1.94520800	-1.53645300	2.14384400
H	0.31029600	-2.14671900	1.79329200
H	1.71522500	-2.86957500	0.98684200
H	3.92228500	-2.21917800	-0.03050500
H	2.71401400	-2.58528500	-1.28714600
H	4.06242100	-1.44284000	-1.62565500
H	-0.17564600	-3.47511500	-0.60038300
H	-1.28611800	-2.32883200	0.16983400
H	-1.34415500	-2.57989300	-1.61615200
H	-0.78231700	-0.08940300	2.54663400
H	-1.16477400	2.02625400	-1.89381200

Ph-4-1245-R2-Nu3

C	-1.28209100	-0.65324400	0.26245500
C	-0.23699700	-1.19532900	1.21193700
C	-0.92572400	-1.06014500	-1.16072200
C	1.03796600	-1.41576700	0.79953800
C	0.39147200	-1.27621500	-1.52431900
C	1.43034600	-1.36353500	-0.58956500
C	2.02477700	-1.77085600	1.79219400
N	2.81910700	-2.03993200	2.58646900
C	2.78049700	-1.53120600	-0.99103800

N	3.88614700	-1.66048900	-1.32219000
C	-1.94963300	-1.14353500	-2.14251800
N	-2.79299100	-1.20437300	-2.93909800
C	-2.59539000	-1.22290900	0.64035600
N	-3.61632400	-1.66792100	0.94122800
C	-1.41244300	0.96298900	0.41971400
C	-0.08252100	1.60969000	0.04093900
C	-1.70431000	1.21937600	1.84234400
C	-2.59555900	1.49849900	-0.40286600
C	0.17712500	1.95811100	-1.28981500
C	0.92571400	1.79739100	0.99437900
N	-1.95938100	1.38773300	2.95526200
H	-3.54286000	1.09153600	-0.02569100
H	-2.62816800	2.59399700	-0.33822300
H	-2.49824800	1.21280200	-1.45660700
C	1.42579100	2.45379000	-1.66151800
H	-0.58906300	1.82610200	-2.05353900
C	2.17415700	2.29270100	0.62321200
H	0.73880300	1.54308400	2.03933600
C	2.43102200	2.61496000	-0.70887800
H	1.61325500	2.71133300	-2.70493000
H	2.94897000	2.42345700	1.37990400
H	3.40979500	2.99681900	-1.00270500
H	-0.51284500	-1.32995500	2.25803300
H	0.61924200	-1.42289400	-2.58225500

Ph-4-1245-R2-Nu2

C	1.03091900	0.13551300	-0.11119400
C	1.81351300	1.44936500	-0.20650100
C	2.00042500	-0.94831700	-0.54989600
C	3.09580300	1.47568700	0.30792000
C	3.27284000	-0.89874900	-0.06758400
C	3.79924600	0.28624500	0.56753000
C	1.27142000	2.59912600	-0.83834900
N	0.85637500	3.52579000	-1.40096600
C	5.09751300	0.30254200	1.14441800
N	6.15411600	0.31531800	1.62430300
C	4.15467300	-2.01256600	-0.31663000
N	4.86075500	-2.90653300	-0.50942600
C	0.66949200	-0.16432500	1.31099600
N	0.45704800	-0.45998800	2.40633600
C	-0.25480200	0.17634500	-0.96163600
Br	-1.00914000	-1.56350500	-1.33792100
S	-1.58426900	1.30950200	-0.27462700
H	-0.04640700	0.63791200	-1.93491000
O	-0.98277300	2.09318100	0.79969500
O	-2.14063400	1.95253100	-1.45981200
C	-2.86848700	0.31224600	0.45082600
C	-2.79969700	-0.00144400	1.80635700
C	-3.95152300	-0.05547300	-0.34726300
C	-3.84233600	-0.73717700	2.36882300
H	-1.94825200	0.32371900	2.40443000

C	-4.98557600	-0.78606200	0.23070500
H	-3.97475800	0.23259500	-1.39882100
C	-4.92670900	-1.12944100	1.58364000
H	-3.80816800	-0.99743600	3.42710500
H	-5.84142400	-1.08547200	-0.37498300
H	-5.73941700	-1.70313100	2.03144400
H	1.65321400	-1.78194200	-1.15611000
H	3.60232200	2.43551600	0.42933800

Ph-4-1245-R2-Nu4

C	-0.85010400	-0.52511300	-0.70927000
C	-1.91825800	0.55645800	-1.08177700
C	-0.72179800	-0.70666500	0.80409300
C	-1.81781200	1.83049200	-0.27821500
H	-1.80504300	0.77601400	-2.15805600
H	-2.91819900	0.12049900	-0.94030100
C	0.50810700	-0.22135800	-1.31705700
C	0.47104700	-0.57367500	1.47862100
C	-0.52729400	2.60263500	-0.33279800
C	-2.85808100	2.24679600	0.45123500
C	1.64987100	-0.12846800	-0.59249900
C	1.69308800	-0.28662600	0.84531200
H	-0.17411500	2.71741100	-1.36914100
H	-0.64774300	3.59791600	0.11519500
H	0.26552100	2.07092600	0.21741100
H	-3.77921900	1.66232000	0.51194900
H	-2.81285300	3.18042100	1.01753700
C	-1.92122600	-0.98596100	1.51576900
N	-2.91909300	-1.22070800	2.06186700
C	2.89914800	-0.16275900	1.57405700
N	3.88977700	-0.06071800	2.17441800
C	2.87309100	0.17725800	-1.29761700
N	3.85098000	0.42356800	-1.86118700
C	-1.32598700	-1.79764900	-1.32311100
N	-1.68581700	-2.78550800	-1.80083500
H	0.46137300	-0.69449300	2.56384900
H	0.53659400	-0.07965100	-2.39874400

Ph-4-1245-R3

C	-1.20696100	0.90423000	-0.07499900
C	-1.20611500	-0.49680300	-0.10803000
C	0.00005800	1.59607900	0.00002100
C	-2.47690800	1.70798800	-0.11190200
C	-2.48330100	-1.24092100	-0.35897600
C	-0.00002200	-1.18903900	-0.00003100
C	1.20706500	0.90414400	0.07501300
O	-3.30884300	1.34877500	0.85693100
O	-2.67683800	2.60335700	-0.88738400
O	-2.43105000	-2.49361100	0.07648500
O	-3.43297400	-0.76549800	-0.92609200
C	1.20613300	-0.49688300	0.10798600

C	2.47695800	1.70798900	0.11187600
C	-4.59496400	1.95909600	0.83015300
C	-3.58098700	-3.29562200	-0.18133000
C	2.48327100	-1.24106800	0.35897300
O	3.30899500	1.34876000	-0.85682000
O	2.67671500	2.60350100	0.88725800
H	-5.13476600	1.57626100	1.70045500
H	-4.50413600	3.05120500	0.88272000
H	-5.11496900	1.68136500	-0.09651500
H	-3.36271600	-4.28384600	0.23178300
H	-3.76378100	-3.36034700	-1.26147900
H	-4.46238100	-2.86035700	0.30623900
O	2.43096700	-2.49375300	-0.07651700
O	3.43298100	-0.76570600	0.92607100
C	4.59502300	1.95927000	-0.83003300
C	3.58085400	-3.29582800	0.18131300
H	5.11459900	1.68266500	0.09720800
H	4.50406700	3.05131100	-0.88387600
H	5.13532300	1.57554600	-1.69963500
H	3.76354700	-3.36068700	1.26147300
H	4.46230800	-2.86053400	-0.30611900
H	3.36259200	-4.28400000	-0.23193000
H	0.00010100	2.68725100	0.00005000
H	-0.00003900	-2.27822400	-0.00003600

Ph-4-1245-R3-Nu1

C	-0.90831800	-0.30969500	0.23504300
C	0.41121200	-0.90217900	0.69636600
C	-0.71564600	1.19726100	0.07082400
C	-1.97875200	-0.57944100	1.30220000
C	1.60549400	-0.35450000	0.37387200
C	-1.84778200	2.09582300	0.26900400
C	0.52044100	1.70466600	-0.26784800
O	-1.55808400	-0.19658200	2.50912200
O	-3.05966000	-1.07250600	1.11733300
C	1.70427300	0.94750000	-0.24532900
C	2.82153000	-1.09357100	0.83584700
O	-1.66394200	3.32197600	-0.27499800
O	-2.86457700	1.84196900	0.88630600
C	-2.54833200	-0.19673000	3.52430200
C	2.91553500	1.50164800	-0.79766100
O	3.59446500	-0.35660100	1.63735100
O	3.06820600	-2.24544400	0.57130500
C	-2.69974600	4.25640900	-0.04621000
H	-2.05992400	0.16150800	4.43585300
H	-3.36511400	0.47820800	3.23369900
H	-2.95167800	-1.20628000	3.67879500
O	3.90150600	0.57174800	-0.94896000
O	3.09831500	2.65386000	-1.15341300
C	4.81930600	-0.95187100	2.03090200
H	-2.38873000	5.18939900	-0.52802300
H	-2.85317800	4.42083900	1.02961600

H	-3.64640100	3.90573900	-0.48003200
C	5.14497000	1.05729700	-1.40900800
H	4.64221300	-1.87359500	2.60121900
H	5.42389400	-1.19547100	1.14579200
H	5.33748500	-0.21537800	2.65263900
H	5.56346400	1.79776300	-0.71163800
H	5.04367700	1.53236100	-2.39467500
H	5.81259800	0.19135200	-1.47835900
C	-1.34503300	-1.05143000	-1.12506000
C	-2.82976700	-0.78793100	-1.46048100
C	-1.03402300	-2.55166000	-0.92402300
C	-0.56609900	-0.47370200	-2.31560100
O	-3.18734800	0.35116400	-1.65330900
C	-3.76318500	-1.94962000	-1.69150900
O	-1.64768300	-3.21431900	-0.11867100
C	0.10896400	-3.17574700	-1.69114700
H	-0.82108100	0.58521500	-2.42707500
H	0.51721300	-0.56185600	-2.16529100
H	-0.84078600	-0.99441600	-3.24536400
H	-4.73394800	-1.56312000	-2.02178900
H	-3.34393600	-2.61607100	-2.46186700
H	-3.85846100	-2.52802600	-0.76639800
H	0.02959700	-3.00442500	-2.77200200
H	1.04956800	-2.71890100	-1.34365500
H	0.12585300	-4.24991900	-1.47514700
H	0.59743500	2.75943600	-0.53910100
H	0.38040100	-1.83164500	1.26938600

Ph-4-1245-R3-Nu3

C	-0.42507000	-0.74459800	0.13334700
C	0.98235000	-1.30329600	0.05269400
C	-0.37099800	0.68854000	0.63789900
C	-1.19590500	-1.63688900	1.11539400
C	2.07615000	-0.51338000	-0.04440300
C	-1.56247200	1.22065900	1.28086400
C	0.76639200	1.45336300	0.47712000
O	-0.70467100	-1.52519700	2.34957500
O	-2.07084800	-2.41366200	0.83184700
C	1.99361500	0.92757200	0.04671000
C	3.39802600	-1.21571000	-0.09443700
O	-1.54082400	2.56180600	1.45636600
O	-2.51789000	0.56030900	1.64933700
C	-1.42090800	-2.21712600	3.35831000
C	3.07588200	1.83012000	-0.27649800
O	4.21124300	-0.82204600	0.89075200
O	3.69462700	-2.08204800	-0.87843800
C	-2.69398500	3.11814400	2.05803900
H	-0.89612900	-2.02540000	4.29918400
H	-2.44940400	-1.83364400	3.41010300
H	-1.45004100	-3.29475700	3.14975800
O	4.08738200	1.22168300	-0.95321200
O	3.12261300	3.02492800	-0.04065800

C	5.52033900	-1.36459500	0.86278200
H	-2.52135700	4.19770700	2.12095700
H	-2.85528900	2.70315300	3.06262400
H	-3.58664900	2.91074600	1.45017900
C	5.21375300	2.02499100	-1.23873000
H	5.49352700	-2.45887900	0.95190700
H	6.02150900	-1.09990400	-0.07918200
H	6.05579000	-0.92861400	1.71178800
H	5.68549700	2.38658600	-0.31340700
H	4.93257100	2.89685600	-1.84526100
H	5.91559100	1.39295700	-1.79357000
C	-1.12171500	-0.82198400	-1.31988900
C	-2.50909200	-0.17522200	-1.33802500
C	-1.21149000	-2.23537800	-1.73862400
C	-0.20033100	-0.15227800	-2.36282300
C	-2.62525500	1.21066500	-1.51145000
C	-3.67850300	-0.92523100	-1.16972600
N	-1.20995600	-3.31916000	-2.13690200
H	0.72002000	-0.73388500	-2.49060000
H	-0.72135600	-0.08282000	-3.32719200
H	0.07897600	0.85397400	-2.02935700
C	-3.87319000	1.83264100	-1.47852600
H	-1.73444600	1.82154300	-1.65231800
C	-4.92697300	-0.30616200	-1.14976900
H	-3.60458600	-2.00374800	-1.03383800
C	-5.03022100	1.07729200	-1.29423300
H	-3.93778200	2.91508500	-1.60397400
H	-5.82529300	-0.91122200	-1.01595200
H	-6.00758600	1.56240000	-1.27269700
H	0.72153000	2.51740800	0.71917200
H	1.09914800	-2.39043300	0.05927200

Ph-4-1245-R3-Nu2

C	1.07127200	-0.02193100	0.67775100
C	1.72738700	1.12103200	-0.08476300
C	-0.06651500	0.57228300	1.49310300
C	2.03921800	-0.77194700	1.58443400
C	3.15191300	1.07432000	-0.35428900
C	0.89586600	2.06929000	-0.65330300
C	-0.83543500	1.54559900	0.95148300
O	2.53000500	0.02027100	2.53309400
O	2.28927600	-1.94480100	1.49545300
O	3.58479400	2.09307600	-1.13288900
O	3.93034200	0.23225700	0.05823300
C	-0.44936900	2.20454700	-0.27687300
C	-1.93424500	2.11678000	1.79345700
C	3.55259000	-0.55059000	3.33468100
C	4.96424900	2.08550500	-1.44491800
C	-1.40604400	3.00778200	-1.01203800
O	-2.71934300	1.16041400	2.31538400
O	-2.08584200	3.28325700	2.04436900
H	3.85091600	0.21912900	4.05275800

H	3.18448300	-1.44196700	3.85913500
H	4.40249900	-0.83126400	2.69787600
H	5.14471800	2.96260100	-2.07513400
H	5.24055000	1.16956500	-1.98552100
H	5.57380300	2.14525300	-0.53273000
O	-0.83954100	3.75151800	-1.99433400
O	-2.60902900	3.03017700	-0.82743600
C	-3.74072300	1.60986300	3.19447100
C	-1.73175100	4.52050400	-2.77578100
H	-4.41982400	2.29721300	2.67302200
H	-3.30475100	2.13164500	4.05676100
H	-4.27995000	0.71642100	3.52336500
H	-2.26806100	5.25291300	-2.15628600
H	-2.47439500	3.88058000	-3.27288400
H	-1.12297700	5.03861800	-3.52432100
C	0.43737000	-1.01011800	-0.33996100
Br	1.61668500	-1.64338700	-1.73749300
S	-0.42937300	-2.46229200	0.43632700
H	-0.35346200	-0.45998100	-0.86506200
O	0.18607800	-3.71878900	0.02666300
O	-0.62155700	-2.18739000	1.86032400
C	-2.02796100	-2.35903400	-0.35250500
C	-2.30460500	-3.20533100	-1.42232400
C	-2.94171800	-1.41057000	0.10734300
C	-3.54389000	-3.09669100	-2.05391700
H	-1.55831600	-3.93341600	-1.74237700
C	-4.17191300	-1.31017600	-0.53909800
H	-2.68922600	-0.75731800	0.94691700
C	-4.47107400	-2.15115800	-1.61418100
H	-3.78424900	-3.75168100	-2.89224100
H	-4.89866400	-0.57025900	-0.20123200
H	-5.43674700	-2.06674900	-2.11485800
H	1.30442100	2.74499000	-1.40603200
H	-0.27396600	0.16926000	2.48234400

Ph-4-1245-R3-Nu4

C	-0.94728900	-0.86435500	0.14883600
C	-1.28555800	-1.14395800	1.65227700
C	-0.85064300	0.62174100	-0.14343400
C	-2.45295200	-0.39973000	2.25864100
H	-1.42909100	-2.23057100	1.76343800
H	-0.37504900	-0.86491700	2.20149500
C	0.39385300	-1.52318400	-0.13442700
C	-1.96359800	-1.56223900	-0.75313300
C	-2.09907000	1.32541800	-0.35434500
C	0.36017600	1.27667800	-0.12638700
C	-3.83719600	-0.95787400	2.06978900
C	-2.24117300	0.72733600	2.95027000
C	1.55525600	-0.83410800	-0.12261000
O	-1.82270600	-1.20849200	-2.03289900
O	-2.74268800	-2.41294500	-0.39953300
O	-1.97914100	2.67226900	-0.44041800

O	-3.19240700	0.79271500	-0.46648500
C	1.59946800	0.61319900	-0.05247600
H	-4.12484500	-0.88801500	1.01268300
H	-3.86903600	-2.02293700	2.34609400
H	-4.56872900	-0.40770900	2.67806700
H	-1.23294900	1.12840500	3.08225100
H	-3.06974600	1.28351500	3.39735700
C	2.82782800	-1.62352000	-0.15715100
C	-2.75955400	-1.76728400	-2.93613700
C	-3.18159500	3.38498000	-0.64719600
C	2.85360800	1.30724100	-0.20506100
O	3.48130900	-1.53711000	1.00657600
O	3.20634500	-2.31062300	-1.07172500
H	-2.50132400	-1.38538400	-3.92873400
H	-2.70904200	-2.86442800	-2.92596900
H	-3.77801100	-1.45651000	-2.66396200
H	-2.91100800	4.44517500	-0.69502200
H	-3.88703400	3.21436500	0.17856600
H	-3.66791600	3.07896700	-1.58415100
O	2.74143700	2.65918600	-0.09564100
O	3.94410000	0.79814800	-0.42330500
C	4.79324300	-2.07121900	1.01169800
C	3.93070500	3.39271800	-0.29852700
H	5.40788300	-1.54094500	0.27105400
H	4.78571800	-3.14335900	0.77362000
H	5.19198300	-1.91044100	2.01838900
H	4.34800900	3.20633500	-1.29837500
H	4.69380100	3.12487200	0.44585800
H	3.66574200	4.45071000	-0.19608800
H	0.40777200	-2.61462700	-0.21198400
H	0.35982100	2.36125900	-0.24091000

Ph-4-1245-R4

C	1.19562500	0.69669900	0.00035900
C	1.19500200	-0.69667100	0.00039700
C	0.00023200	1.40260900	0.00005300
C	-0.00052000	-1.40222800	0.00039800
C	-1.19479300	0.69693900	0.00035400
C	-1.19536300	-0.69695800	0.00036300
F	2.35597300	-1.34088000	-0.00005800
F	-2.35675900	-1.33987900	-0.00074900
H	0.00085900	-2.49197300	0.00015400
H	0.00004100	2.49218400	-0.00028400
F	-2.35592600	1.34034700	0.00012200
F	2.35648900	1.34012800	-0.00058200

Ph-4-1245-R4-Nu1

C	0.15536700	-0.04217600	-0.43090500
C	1.10774100	-1.12389100	-0.13488900
C	0.83249400	1.24772400	-0.23654200
C	2.45794500	-0.90926600	0.06257000

C	2.17221100	1.44576700	-0.03259600
C	3.03276900	0.35358100	0.12413100
F	0.03914400	2.35185500	-0.40943700
F	4.36523500	0.52084000	0.33625900
F	3.25871200	-1.98062100	0.24889500
F	-0.30057700	-0.08568000	-1.83570000
C	-1.22658700	-0.17740100	0.32961900
C	-1.96933500	-1.47144600	-0.05885100
C	-2.18482400	0.96203100	-0.06265700
C	-0.97218800	-0.22526800	1.84213900
O	-1.52246600	-2.31450500	-0.79349900
C	-3.30362600	-1.70509800	0.63107000
O	-2.83173200	0.88225800	-1.08179300
C	-2.39730100	2.11337300	0.89465600
H	-0.43294200	-1.14848200	2.09642100
H	-0.33423400	0.61692200	2.14367600
H	-1.90482200	-0.18683300	2.42261200
H	-3.10833200	-2.24224400	1.57215300
H	-3.83936200	-0.77879400	0.87246100
H	-3.92482100	-2.34693700	-0.00478300
H	-3.12531200	1.79368300	1.65754800
H	-1.47687500	2.41463500	1.40387000
H	-2.82257100	2.95786000	0.34008900
H	2.55689400	2.46638900	0.01034800
H	0.75534000	-2.15335400	-0.13867700

Ph-4-1245-R4-Nu3

C	0.57970700	0.97710600	0.12926700
C	1.10436900	0.36190900	-1.08008900
C	1.14259300	0.32231300	1.30026300
C	2.20585600	-0.47254300	-1.08105500
C	2.22859200	-0.51765200	1.29393200
C	2.80188500	-0.92515700	0.08774200
F	0.60601200	0.64393400	2.51705200
F	3.87556100	-1.75223800	0.05666700
F	2.68832100	-0.91359400	-2.25878900
F	0.99043600	2.47221000	0.18540400
C	-0.98745800	1.20155800	0.09469800
C	-1.72630300	-0.13646700	0.02088400
C	-1.25513100	1.99096200	-1.12419300
C	-1.51990600	2.05991700	1.26494300
C	-1.71727300	-0.97750400	1.14362400
C	-2.40505400	-0.56199200	-1.12431600
N	-1.49724100	2.61866700	-2.06234700
H	-0.92992500	2.98030200	1.32822300
H	-2.57578200	2.30771200	1.08807700
H	-1.43710900	1.51839700	2.21111100
C	-2.37225000	-2.20600200	1.11802400
H	-1.18546900	-0.66897900	2.04451400
C	-3.06252700	-1.79412600	-1.15044600
H	-2.42431000	0.07223800	-2.01187700
C	-3.04961100	-2.62086000	-0.03032100

H	-2.35125500	-2.84517100	2.00227900
H	-3.58620400	-2.10450300	-2.05613400
H	-3.56256800	-3.58368700	-0.04949000
H	2.62660200	-0.87675900	2.24442400
H	0.65879300	0.60821100	-2.04543400

Ph-4-1245-R4-Nu2

C	1.49694200	0.32175400	-0.29778000
C	2.12451200	-0.99558700	-0.40340800
C	2.33439900	1.18587100	0.54326500
C	3.46430400	-1.19669100	-0.12309000
C	3.65575400	0.96954300	0.83300900
C	4.26590700	-0.23796700	0.47721300
F	1.75129100	2.33239600	0.99274100
F	5.57131500	-0.48023800	0.75419200
F	4.00454200	-2.40005300	-0.39767800
F	1.35154500	0.92350800	-1.65171500
C	0.01825400	0.32500300	0.21087300
Br	-0.99180700	1.90768100	-0.31665100
S	-0.92541400	-1.16534400	-0.30794900
H	-0.00495400	0.29387800	1.30618600
O	-0.84144900	-1.29406600	-1.75964000
O	-0.48513800	-2.26265900	0.55356700
C	-2.62610400	-0.83955300	0.12070800
C	-3.52396500	-0.49031600	-0.88446100
C	-3.01927400	-0.99564800	1.44819400
C	-4.85733100	-0.27605300	-0.54105800
H	-3.17172700	-0.39213600	-1.91189700
C	-4.35363400	-0.76774700	1.77955500
H	-2.29333800	-1.30131400	2.20322400
C	-5.26752700	-0.40871500	0.78704700
H	-5.57848200	-0.00369200	-1.31245800
H	-4.68221900	-0.88015700	2.81330900
H	-6.31194300	-0.23576600	1.05060300
H	4.21190000	1.73181400	1.38137000
H	1.58167500	-1.82605200	-0.85002500

Ph-4-1245-R4-Nu4

C	-0.41521600	-0.17372700	0.82686800
C	-1.93974500	0.01461900	0.79632800
C	0.37023800	1.03067300	0.60989100
C	-2.49820600	0.58180400	-0.48299000
H	-2.39098500	-0.96961700	0.99748400
H	-2.19164000	0.67335200	1.63812400
C	0.11619400	-1.26893900	0.02544800
C	1.65961500	0.97685400	0.10901300
C	-2.32948700	-0.24201600	-1.73041000
C	-3.12503000	1.76380600	-0.49884000
C	1.38561200	-1.31849100	-0.48935100
C	2.20628700	-0.18334000	-0.42197600
H	-2.67233600	-1.27417500	-1.56352000

H	-2.87957700	0.19142200	-2.57635500
H	-1.26307400	-0.30830300	-1.99689700
H	-3.23610300	2.35646000	0.41291500
H	-3.54383100	2.17393800	-1.42163900
F	-0.69772800	-2.35404800	-0.13134000
F	3.47612600	-0.20335800	-0.90598900
F	2.39685700	2.10777200	0.06449500
F	-0.29898000	-0.62856600	2.31871600
H	-0.02976400	1.99995300	0.91400800
H	1.73926300	-2.22668900	-0.98094400

Ph-4-1245-R5

C	1.19088000	-0.69546900	0.01783700
C	-0.00000800	-1.40567900	0.02487600
C	1.19091100	0.69551200	-0.01787600
C	-1.19086600	-0.69551400	0.01784700
C	-0.00000800	1.40570100	-0.02488500
C	-1.19086500	0.69553500	-0.01780400
N	-2.44111100	1.47111300	-0.15183800
O	-2.49628300	2.50798900	0.45410900
O	-3.27531500	1.02119100	-0.89181300
N	-2.44114400	-1.47112900	0.15184600
O	-3.27543800	-1.02112900	0.89166700
O	-2.49619100	-2.50807300	-0.45398400
N	2.44111100	-1.47116700	0.15212600
O	3.27531500	-1.02119000	0.89199600
O	2.49617200	-2.50817900	-0.45362300
N	2.44118200	1.47116600	-0.15210000
O	3.27554000	1.02098000	-0.89170300
O	2.49612700	2.50836100	0.45334000
H	0.00001700	-2.49534100	0.06036100
H	0.00005400	2.49535700	-0.06046800

Ph-4-1245-R5-Nu1

C	0.53888200	-0.10915700	0.46673900
C	-0.64740500	-1.04507000	0.57517200
C	0.00294100	1.30419000	0.41384500
C	-1.88734900	-0.68812300	0.20731800
C	-1.27674400	1.62393400	-0.00139200
C	-2.23583500	0.64716000	-0.19164200
N	-3.43730100	0.94058900	-0.88619800
O	-3.76313700	2.10662900	-1.03267000
O	-4.08229400	0.00264600	-1.32810300
N	-2.94940000	-1.67554100	0.47963600
O	-3.91928600	-1.28204000	1.07713100
O	-2.75601500	-2.81599000	0.13378100
N	1.29990400	-0.28400300	1.82657200
O	0.64045300	-0.06911700	2.81260100
O	2.45161700	-0.61247800	1.81932100
N	0.88111200	2.37590700	0.70676600
O	2.01632000	2.08812900	1.07352300

O	0.50233600	3.52861600	0.58543600
C	1.49337800	-0.52598400	-0.71882900
C	2.03427000	-1.96387700	-0.53983300
C	2.67758000	0.45035600	-0.95338000
C	0.63481500	-0.57815200	-2.00604600
O	1.56557100	-2.72283600	0.27280600
C	3.10957500	-2.43955000	-1.48942900
O	3.79521500	0.15519300	-0.61471600
C	2.42754700	1.70507700	-1.76260000
H	-0.06843300	-1.42058000	-1.96551100
H	0.05388200	0.34643000	-2.12526000
H	1.26802900	-0.70012900	-2.89433300
H	2.95220300	-3.50686500	-1.68407200
H	3.14646100	-1.87354700	-2.42741600
H	4.07500700	-2.30323700	-0.98388000
H	2.57177200	1.45197300	-2.82466000
H	1.41606800	2.11483400	-1.66009000
H	3.17450300	2.45456900	-1.47864900
H	-0.46619500	-2.04350900	0.96509700
H	-1.51968000	2.66656000	-0.20107700

Ph-4-1245-R5-Nu3

C	1.29281200	0.46049400	-0.15342600
C	0.20874800	1.51206700	-0.17360400
C	0.98494100	-0.56703300	-1.20736000
C	-1.03133000	1.21295500	-0.58880300
C	-0.31208500	-0.86519800	-1.57130800
C	-1.36536200	-0.04460700	-1.19565200
N	-2.69658400	-0.52930200	-1.22524900
O	-2.93099300	-1.56915000	-1.81857800
O	-3.55148500	0.10903000	-0.63043400
N	-2.01280600	2.31337800	-0.57197700
O	-2.62653700	2.51538300	-1.58847800
O	-2.10013400	2.96364000	0.44177700
N	2.57151600	1.24876100	-0.55769000
O	2.59571600	1.66054100	-1.68611900
O	3.41059600	1.45730500	0.27813900
N	2.03382700	-1.38214500	-1.70752400
O	3.17812000	-1.12666100	-1.34793700
O	1.77300800	-2.28778200	-2.48039600
C	1.43650700	-0.14676700	1.32035000
C	0.08982300	-0.82931400	1.64052000
C	1.61921600	0.97505700	2.26552800
C	2.61202100	-1.12338500	1.51584600
C	-0.12897600	-2.16056200	1.26710400
C	-0.97203700	-0.10857700	2.20000100
N	1.73544300	1.83369200	3.02691500
H	3.56839200	-0.61862600	1.35431900
H	2.56747000	-1.51719300	2.54016100
H	2.54881500	-1.96150300	0.81626300
C	-1.39011700	-2.73932800	1.40433300
H	0.67274500	-2.75163400	0.82534400

C	-2.23266000	-0.68603600	2.33786800
H	-0.82882200	0.93136100	2.49760000
C	-2.44953600	-2.00071400	1.92869100
H	-1.54402700	-3.77162400	1.08724900
H	-3.05133200	-0.09610100	2.75172800
H	-3.44039400	-2.44812800	2.01821500
H	-0.51137400	-1.77990900	-2.12831100
H	0.44062800	2.50990700	0.20180200

Ph-4-1245-R5-Nu2

C	-0.75308800	0.83347200	-0.56897100
C	-1.35744500	1.36652900	0.70168800
C	-1.74007600	-0.12462200	-1.19738700
C	-2.26398100	0.64776400	1.44654500
C	-2.64994300	-0.77697600	-0.45321000
C	-2.87082300	-0.49107800	0.93341600
N	-3.60357800	-1.62346700	-1.19949100
O	-3.14866200	-2.39259200	-2.01051400
O	-4.77476700	-1.44493700	-0.98332200
N	-3.59783300	-1.36844500	1.77772000
O	-3.84167900	-2.48923100	1.35890100
O	-3.92612800	-0.97978700	2.88549200
N	-0.84687200	2.58474600	1.23417400
O	-1.26622200	2.99579800	2.29745200
O	-0.00790500	3.17323000	0.56830100
N	-0.59466100	1.98551700	-1.59911800
O	-1.60537100	2.58785300	-1.85335300
O	0.48142000	2.17294500	-2.08908600
C	0.59322400	0.07886500	-0.35791800
Br	1.27844100	-0.75716800	-1.96704500
S	1.96399700	0.89150800	0.58458300
H	0.33646300	-0.75439700	0.31062800
O	2.61418100	1.93319400	-0.18987300
O	1.40023500	1.15654200	1.90671400
C	3.12370300	-0.44922300	0.77713700
C	4.24328000	-0.49544900	-0.05093800
C	2.90305100	-1.38610500	1.78520800
C	5.16035600	-1.52849500	0.12807200
H	4.38316600	0.26957500	-0.81543400
C	3.82617300	-2.41744700	1.94846100
H	2.03266000	-1.29700700	2.43726500
C	4.94837000	-2.48719200	1.12103100
H	6.04421700	-1.58429300	-0.50808300
H	3.67343000	-3.16208300	2.73018400
H	5.66947200	-3.29459600	1.25572100
H	-1.69040600	-0.29083800	-2.27312200
H	-2.49541600	0.97755900	2.45859400

Ph-4-1245-R5-Nu4

C	-1.04739200	-0.74156600	-0.02032800
C	-1.80577100	-0.85625900	1.32551100

C	0.32371400	-1.34776200	0.09254800
C	-1.30830300	0.16553900	2.32657400
H	-2.87911800	-0.73149600	1.14171000
H	-1.64602800	-1.87329600	1.70669400
C	-0.94880400	0.63145100	-0.60879300
C	1.45036200	-0.63562500	-0.07357900
C	-2.05489900	1.47130300	2.39263900
C	-0.25199900	-0.09215400	3.10439700
C	0.22637400	1.33604100	-0.72993200
C	1.45480000	0.75260400	-0.44461400
H	-3.12916300	1.29411900	2.55623100
H	-1.67477600	2.10412000	3.20516600
H	-1.96421400	2.03987600	1.45387200
H	0.28035300	-1.04511700	3.04433800
H	0.11753800	0.64741200	3.81877200
N	2.60694300	1.56294300	-0.30925100
O	2.59320900	2.69081800	-0.77725300
O	3.56702000	1.10123100	0.29086000
N	2.70658200	-1.40880800	-0.04683700
O	3.43274000	-1.30023100	-1.00178600
O	2.89287900	-2.13992100	0.89588200
N	-1.78987900	-1.67161800	-1.04767000
O	-1.62611200	-1.44109600	-2.21597000
O	-2.41860800	-2.59756200	-0.60165200
N	-2.16952000	1.28271800	-0.92591000
O	-3.20168700	0.63191200	-0.81011800
O	-2.16229700	2.44678800	-1.29313600
H	0.38300300	-2.41308200	0.32328000
H	0.18786600	2.38388000	-1.02355500

Ph-4-2134-R1

C	-0.69335600	0.21031900	-0.06266100
C	0.70880500	0.21842800	0.03694400
C	-1.38438600	-1.01850500	-0.00025400
C	1.40866600	-1.00142000	-0.05300800
C	-0.67380600	-2.21139800	0.00907200
C	0.71138000	-2.20136600	-0.09329000
C	2.92342400	-1.10556300	-0.20138500
C	1.46369900	1.49837700	0.41712400
C	-2.89229500	-1.12383200	0.22009400
C	-1.49432900	1.46269500	-0.42995800
F	-2.44345000	1.73120700	0.46003900
F	-0.75407100	2.55108200	-0.58115900
F	-2.07919100	1.25473100	-1.61021900
F	-3.62173400	-0.46547800	-0.67163600
F	-3.20672400	-0.65485600	1.42698800
F	-3.28452200	-2.39628600	0.17903700
F	3.41312400	-0.10241200	-0.92701600
F	3.56132200	-1.13957500	0.96288400
F	3.23440500	-2.23407400	-0.84185000
F	0.74666300	2.21720200	1.27936200
F	2.59054100	1.19206700	1.05777400

F	1.78620900	2.26137300	-0.61671800
H	-1.20402000	-3.15987200	0.06564600
H	1.25407100	-3.14137300	-0.17908700

Ph-4-2134-R1-Nu1

C	-0.44134100	0.12870100	0.20351200
C	-0.84091600	1.56197900	-0.20259100
C	1.10898500	0.02107200	0.14203500
C	0.03771700	2.46326200	-0.73711800
C	1.91188100	1.02662300	-0.39860500
C	1.40856300	2.23204200	-0.89237100
C	-2.25405800	2.05852500	-0.14311900
C	3.42702800	0.90936300	-0.50768400
C	1.75704300	-1.14523200	0.82745900
C	-0.85357700	0.00653500	1.71525600
F	-0.05022700	0.81223100	2.43023900
F	-2.09106500	0.44060800	1.96263000
F	-0.74585700	-1.19882000	2.26250900
F	0.96694100	-2.23174000	0.89629000
F	2.12284000	-0.89430200	2.10257800
F	2.87103100	-1.58764900	0.21773900
F	3.99257500	2.06942200	-0.86682300
F	4.01367900	0.57188200	0.64559500
F	3.80852500	0.02586800	-1.44037800
F	-2.55974000	2.75858100	0.96587700
F	-2.52817000	2.90445900	-1.16087100
F	-3.18565300	1.09172100	-0.24053300
C	-1.17852400	-0.92862100	-0.82065300
C	-2.60421500	-1.37340600	-0.41179700
C	-0.48534900	-2.30080400	-1.07894900
C	-1.16687700	-0.26140600	-2.21366700
O	-2.87602000	-1.66469800	0.72289200
C	-3.62273800	-1.55263900	-1.51763700
O	-1.03725300	-3.33394000	-0.78228000
C	0.78304500	-2.35137600	-1.90744800
H	-1.87230700	0.57477000	-2.26808600
H	-0.16506100	0.13510500	-2.42729000
H	-1.43263700	-0.98052500	-3.00077300
H	-3.81921900	-0.60451600	-2.03362300
H	-3.24690000	-2.27261000	-2.25885200
H	-4.54873000	-1.93438900	-1.07352200
H	0.48527000	-2.48291500	-2.95875600
H	1.40312200	-1.45328800	-1.83338800
H	1.35227700	-3.23610500	-1.59961500
H	-0.35813900	3.42792500	-1.06054100
H	2.05459700	2.98759000	-1.32384700

Ph-4-2134-R1-Nu3

C	-0.33477400	-0.73490100	0.02350400
C	-1.02149900	-0.05952900	1.22256700
C	1.18513000	-0.47664000	0.13597600

C	-0.41092500	0.93925400	1.93682700
C	1.69214800	0.59662600	0.87977100
C	0.91315300	1.34654700	1.75432500
C	-2.45830300	-0.27523600	1.61930800
C	3.12455000	1.09516000	0.74597400
C	2.18037600	-1.38939900	-0.51738000
C	-0.62462800	-2.28060800	0.06149500
F	0.31021300	-2.93012100	0.75878800
F	-1.78376900	-2.57295600	0.65457100
F	-0.70476300	-2.85598200	-1.14362000
F	1.64245300	-2.30345400	-1.33508900
F	2.92009300	-2.09629500	0.36097300
F	3.08117300	-0.73364200	-1.28700300
F	3.41679000	2.02706500	1.66222800
F	4.05949200	0.15255600	0.88773100
F	3.32650500	1.68363300	-0.44390500
F	-2.63914800	-1.20926500	2.57490200
F	-3.00235300	0.84349400	2.14142800
F	-3.27189700	-0.61155400	0.60670600
C	-0.91142600	-0.12008900	-1.40950000
C	-1.11238400	1.38867500	-1.22896000
C	-2.18996900	-0.72784700	-1.82271700
C	0.03341600	-0.37594000	-2.60432300
C	-0.01583100	2.25421900	-1.32826200
C	-2.36792400	1.93308300	-0.93556600
N	-3.15483000	-1.20295800	-2.24246200
H	0.06869700	-1.43896400	-2.85987600
H	-0.33216600	0.19244900	-3.47004000
H	1.04977500	-0.03626300	-2.37557700
C	-0.16979800	3.62411600	-1.12094500
H	0.97701800	1.86063500	-1.54290300
C	-2.52260800	3.30317900	-0.72845500
H	-3.23705800	1.27854800	-0.85951100
C	-1.42252600	4.15490500	-0.81613800
H	0.70105700	4.27785400	-1.19336000
H	-3.51084200	3.70387500	-0.49708300
H	-1.54159600	5.22727200	-0.65311400
H	-0.99837800	1.45250900	2.69849500
H	1.31713500	2.18803100	2.30582500

Ph-4-2134-R1-Nu2

C	-0.76291300	0.58433600	0.37300300
C	-1.69482200	-0.62398100	0.19795800
C	-1.26653100	1.75513900	-0.46603200
C	-2.76564900	-0.60762600	-0.70444300
C	-2.32151100	1.65776700	-1.32492600
C	-3.09066800	0.49579300	-1.48761900
C	-3.67614500	-1.80845400	-0.92240000
C	-1.42528800	-1.85679300	1.01256600
C	-0.61483900	3.09794000	-0.35579900
C	-0.79302700	1.06339800	1.86145300
F	-1.96978300	1.64908100	2.10112300

F	-0.67689800	0.10102500	2.76796700
F	0.15535700	1.96978200	2.11704500
F	0.70706600	3.03447200	-0.16840900
F	-1.09400100	3.84541800	0.66271200
F	-0.80802300	3.84314700	-1.45694400
F	-3.11941000	-2.73889800	-1.70531900
F	-4.04736500	-2.40426900	0.21507200
F	-4.81603400	-1.45724000	-1.53259000
F	-2.20507400	-1.98958200	2.10248200
F	-1.59320100	-2.99295300	0.31130300
F	-0.15849000	-1.93299000	1.47498300
C	0.72386700	0.11606800	-0.09674200
Br	1.10801200	0.56950100	-1.94328700
S	2.26405700	0.38993700	0.89432300
H	0.71277600	-0.97323300	-0.11900400
O	2.84229900	1.71037200	0.68848000
O	1.97089200	-0.08620400	2.24331800
C	3.36933100	-0.80893000	0.16133900
C	4.37797800	-0.37455500	-0.69424100
C	3.21211500	-2.15095600	0.50513300
C	5.24874000	-1.32079400	-1.23228900
H	4.46900300	0.68738000	-0.92556500
C	4.08471300	-3.08701000	-0.04632600
H	2.42247100	-2.44922800	1.19760100
C	5.09866700	-2.67126900	-0.91193600
H	6.04673700	-1.00256900	-1.90408500
H	3.97764200	-4.14239700	0.20649200
H	5.78122800	-3.40767000	-1.33854500
H	-2.58201400	2.54219000	-1.90856400
H	-3.93012000	0.47729400	-2.17388500

Ph-4-2134-R1-Nu4

C	0.69421400	-0.41596100	-0.01688600
C	1.15356200	-1.17767300	1.28446600
C	1.43820200	0.92660700	-0.14264200
C	0.78838100	-0.47252900	2.56922400
H	0.73254600	-2.18922200	1.28217200
H	2.24032300	-1.28632900	1.23676200
C	-0.83691800	-0.17581300	-0.09711600
C	0.79041900	2.12462300	-0.26221500
C	-0.60220200	-0.67127500	3.11121800
C	1.68443600	0.29443900	3.20161400
C	-1.37534500	1.10157300	-0.25813500
C	-0.60328000	2.26388600	-0.32117500
H	-0.84128100	-1.74342100	3.18554000
H	-0.70972000	-0.21361000	4.10405800
H	-1.35281700	-0.22813100	2.43965400
H	2.69069100	0.43229300	2.79910100
H	1.43280300	0.81022000	4.13215300
C	2.92819300	1.00131400	-0.26327400
C	-2.87197300	1.36823700	-0.33986600
C	-1.75648700	-1.35743800	-0.02803400

C	1.12291400	-1.29996700	-1.21231100
F	0.92932100	-0.68087900	-2.37768300
F	2.42245000	-1.63115800	-1.15372700
F	0.45763400	-2.45968200	-1.27850800
F	-1.23605800	-2.43066800	0.59497200
F	-2.16416800	-1.81633900	-1.23339300
F	-2.89386400	-1.10606400	0.65293100
F	-3.13273800	2.58670300	-0.83547600
F	-3.52521900	0.51695000	-1.13775500
F	-3.46466600	1.33883800	0.86177000
F	3.37637500	0.77617100	-1.52119000
F	3.40323800	2.21937100	0.05543200
F	3.62131100	0.14096900	0.51022600
H	-1.05735500	3.24155900	-0.43687000
H	1.39759300	3.02858600	-0.33016400

Ph-4-2134-R2

C	0.70146400	0.40344300	-0.00015100
C	1.39592000	-0.82066400	0.00012700
C	-0.70146400	0.40336300	-0.00039900
C	0.69525700	-2.02981400	-0.00001500
C	-1.39586000	-0.82079100	-0.00025000
C	-0.69510100	-2.02988500	0.00001800
C	2.83676700	-0.82294700	0.00005600
N	3.99015900	-0.83117800	0.00024600
C	-2.83669000	-0.82307700	-0.00013500
N	-3.99008200	-0.83125000	0.00019100
C	-1.42263500	1.64870400	0.00009400
N	-2.00320600	2.64525200	0.00035200
C	1.42248300	1.64883800	-0.00015000
N	2.00296800	2.64543200	-0.00013000
H	1.24477500	-2.97032900	0.00010100
H	-1.24449600	-2.97047300	0.00011300

Ph-4-2134-R2-Nu1

C	-0.32555000	0.39596800	0.56554700
C	-0.51075800	1.84109600	0.08367500
C	1.17813500	0.16090800	0.66358700
C	0.35735300	2.36369100	-0.85705800
C	2.00086200	0.72242200	-0.31036600
C	1.56563600	1.73586700	-1.18412000
C	-1.62006300	2.60719900	0.53328000
N	-2.53492900	3.22773700	0.88954300
C	3.38373300	0.31464300	-0.37696300
N	4.49289100	0.00043400	-0.45460700
C	1.75164700	-0.59351700	1.72247000
N	2.26855600	-1.16745700	2.58833800
C	-0.93997600	0.28134300	1.90640200
N	-1.45338100	0.26199000	2.93888300
C	-1.08640100	-0.64350900	-0.39033500
C	-0.98271200	-2.07248000	0.17649900

C	-0.39027500	-0.70378200	-1.76315800
C	-2.56977800	-0.26837500	-0.50939000
O	-0.41781300	-2.30973400	1.21308900
C	-1.69330100	-3.16653800	-0.59016200
O	0.53402600	-1.47042200	-1.90991700
C	-0.93893100	0.10813000	-2.90856800
H	-3.05735100	-0.30648200	0.47598200
H	-2.69142200	0.74623500	-0.90837200
H	-3.09916300	-0.95823500	-1.18042000
H	-2.72500000	-3.23202000	-0.21160400
H	-1.73469800	-2.97776600	-1.66977500
H	-1.19367600	-4.12120200	-0.38987800
H	-1.84546600	-0.39463300	-3.28296500
H	-1.22547400	1.11995800	-2.59970200
H	-0.19400400	0.14093600	-3.71126400
H	0.11286100	3.32741000	-1.31008100
H	2.23400200	2.13973100	-1.94044400

Ph-4-2134-R2-Nu3

C	1.16557100	0.14152100	-0.14592100
C	0.34946700	1.40008300	-0.40657700
C	1.15502500	-0.70117500	-1.41861500
C	-0.72289400	1.35315600	-1.30096500
C	0.07933900	-0.66520100	-2.28622700
C	-0.93760000	0.29338600	-2.19111500
C	-1.62845000	2.47731500	-1.35170400
N	-2.37312300	3.35920700	-1.40027500
C	0.63851900	2.59266600	0.30718500
N	0.87473200	3.57681600	0.87655700
C	2.24952900	-1.56891800	-1.68905700
N	3.14182800	-2.28072500	-1.90190300
C	2.55930600	0.52223000	0.17731600
N	3.64717500	0.80926200	0.43109300
C	0.61067500	-0.70014200	1.12942600
C	-0.85559900	-1.06476900	0.90661600
C	0.71920200	0.17615900	2.31100800
C	1.49802700	-1.92775400	1.39455100
C	-1.20186600	-2.27301100	0.29142100
C	-1.87592200	-0.17448900	1.26498800
N	0.83727200	0.84737300	3.24211400
H	2.52057100	-1.61637500	1.64618200
H	1.08810400	-2.50944400	2.23055000
H	1.54610500	-2.57374300	0.51056500
C	-2.53601300	-2.57243600	0.01841500
H	-0.42990600	-2.98661400	0.00460300
C	-3.20880200	-0.47150900	0.98904600
H	-1.62582500	0.76700200	1.75835700
C	-3.54289000	-1.67032600	0.35895100
H	-2.78701900	-3.51740200	-0.46568000
H	-3.98824700	0.23831800	1.26974100
H	-4.58633300	-1.90466700	0.14264800
H	0.06542700	-1.38017300	-3.11190500

H	-1.78578800	0.29885400	-2.87060300

Ph-4-2134-R2-Nu2

C	-1.25176800	0.38327900	0.20064900
C	-2.28111700	-0.66264900	-0.20885200
C	-2.07749800	1.67105700	0.27158200
C	-3.42799700	-0.71601500	0.58679300
C	-3.22347200	1.60902600	1.05364700
C	-3.80004800	0.37737400	1.39573600
C	-4.32678200	-1.83496300	0.46491000
N	-5.05608100	-2.72753200	0.37820100
C	-2.13083900	-1.46594400	-1.36568000
N	-2.02432500	-2.09712600	-2.33402900
C	-1.79207100	2.85403500	-0.46070100
N	-1.62902800	3.83490100	-1.05979700
C	-0.74542900	0.08984400	1.58106700
N	-0.35261500	-0.09599900	2.64981700
C	-0.03424500	0.43272600	-0.73733600
Br	1.16250600	1.89883500	-0.34099500
S	0.94547300	-1.17859500	-0.78128400
H	-0.35199200	0.54367500	-1.78242000
O	0.22667200	-2.16738700	0.01576100
O	1.20151600	-1.39147400	-2.20113800
C	2.51439800	-0.91273700	0.01751400
C	2.62081200	-1.13430000	1.38870200
C	3.60947500	-0.56758800	-0.77224200
C	3.86889900	-0.97419900	1.98877900
H	1.74358200	-1.42115600	1.96933300
C	4.84996700	-0.41730000	-0.15820500
H	3.48165100	-0.42383800	-1.84560300
C	4.97604600	-0.61525300	1.21868400
H	3.97623800	-1.13738500	3.06152400
H	5.72107300	-0.14757700	-0.75604400
H	5.94990700	-0.49443700	1.69527300
H	-3.74271400	2.53601800	1.30674800
H	-4.67415500	0.31581100	2.03992500

Ph-4-2134-R2-Nu4

C	-0.45110700	-0.15264600	0.66138200
C	-1.23512700	1.19658400	0.66485000
C	1.01073200	0.03989800	0.25735300
C	-1.18546200	1.97541200	-0.63017300
H	-2.28238900	0.97034800	0.92704800
H	-0.82508200	1.81721900	1.47474500
C	-1.07695400	-1.22433600	-0.23684200
C	1.69015600	-0.91686100	-0.49516800
C	-1.90769600	1.41595700	-1.82729000
C	-0.54037100	3.14557900	-0.68681900
C	-0.32268600	-2.11949000	-0.96608100
C	1.07352000	-2.03439700	-1.07548200
H	-2.92603100	1.09540800	-1.55970500

H	-1.96718400	2.16203600	-2.63074800
H	-1.38970700	0.52648400	-2.21724400
H	-0.00276200	3.54137100	0.17762800
H	-0.52187900	3.73331700	-1.60817800
C	3.10296300	-0.72374100	-0.73335700
N	4.23238400	-0.58709000	-0.93400600
C	1.69140400	1.15989100	0.79887100
N	2.22284700	2.07741800	1.27464400
C	-2.49379000	-1.33870000	-0.21955000
N	-3.65342700	-1.40355300	-0.19479200
C	-0.49731800	-0.65311300	2.07356300
N	-0.52030300	-1.06069900	3.15431200
H	-0.85185700	-2.90397400	-1.51178200
H	1.64680500	-2.74496700	-1.66441200

Ph-4-2134-R3

C	0.77145100	0.13333300	0.09078000
C	1.66688300	-0.93461900	-0.05448800
C	-0.60447400	-0.10351200	0.03788800
C	1.25562200	1.53413300	0.37804800
C	3.13855500	-0.65560400	-0.11940200
C	1.18548700	-2.23261700	-0.23108000
C	-1.54274800	1.06933300	0.12011200
C	-1.08107900	-1.41292700	-0.11856800
O	0.95240600	2.35946600	-0.61618300
O	1.78950500	1.85689400	1.40104400
O	3.86899800	-1.73334300	0.13741500
O	3.60207300	0.41881000	-0.40146600
C	-0.18477800	-2.47013600	-0.26349500
O	-2.41310000	1.06317400	-0.88147500
O	-1.48134900	1.92389900	0.96315200
C	-2.54394000	-1.74705500	-0.08937100
C	1.22170100	3.73778800	-0.37626500
C	5.28221100	-1.56820700	0.04831700
C	-3.40182500	2.08866900	-0.85225800
O	-3.21256600	-0.98342500	0.76963100
O	-3.03878800	-2.63800100	-0.72641300
H	0.93647200	4.26968400	-1.28797000
H	0.62551000	4.08701200	0.47739100
H	2.28723100	3.88534200	-0.16057400
H	5.71844200	-2.54377000	0.27823300
H	5.62069800	-0.81475800	0.77062200
H	5.56384500	-1.24773000	-0.96282000
H	-4.02703300	1.93685500	-1.73611500
H	-2.92615500	3.07701100	-0.88079600
H	-4.00118200	2.00452800	0.06434000
C	-4.62021800	-1.19520200	0.83649200
H	-4.83505200	-2.23186900	1.12430200
H	-5.07862400	-0.99033000	-0.14015000
H	-4.99779900	-0.50068400	1.59175300
H	-0.57489700	-3.47998300	-0.39319500
H	1.89402800	-3.05136500	-0.35092700

Ph-4-2134-R3-Nu1

C	-0.51217900	0.02891400	0.06575000
C	-1.28247600	-1.29587000	-0.00350800
C	0.96842400	-0.30175200	0.26541800
C	-1.02650000	0.79986100	1.30112600
C	-2.69737600	-1.29428900	0.33385200
C	-0.67114000	-2.47473700	-0.38384100
C	1.77562500	0.70052400	0.93153200
C	1.50482000	-1.50876600	-0.17569800
O	-0.80188200	0.08491700	2.41286100
O	-1.57690300	1.86446400	1.32870200
O	-3.31614000	-2.48392300	0.13724700
O	-3.32856900	-0.33233400	0.73387100
C	0.71082700	-2.59408800	-0.56493400
O	3.04418700	0.31334100	1.20284000
O	1.38008600	1.80286600	1.27370000
C	2.98503500	-1.75343000	-0.26122800
C	-1.24449200	0.68398700	3.61615300
C	-4.70221900	-2.50941100	0.42006300
C	3.89139300	1.30139400	1.75642200
O	3.56471900	-0.97792000	-1.18164600
O	3.57862600	-2.61291600	0.33800200
H	-1.00550700	-0.01870700	4.42082600
H	-0.72904900	1.64051600	3.78119200
H	-2.32720000	0.86758400	3.57814200
H	-5.04041300	-3.53128600	0.21919800
H	-5.24611400	-1.79986000	-0.21958000
H	-4.89583000	-2.24719700	1.46915800
H	4.85921800	0.81883400	1.92896400
H	3.48408600	1.67994500	2.70317600
H	4.00885100	2.14735500	1.06426200
C	4.97123300	-1.10896900	-1.29900700
H	5.24712900	-2.13514900	-1.57644500
H	5.45552100	-0.85974200	-0.34423000
H	5.28471700	-0.40666900	-2.07728600
C	-0.70324900	0.92634900	-1.28966900
C	0.25512200	2.15511500	-1.27164900
C	-2.16541900	1.34474400	-1.54142600
C	-0.25325000	0.06185300	-2.48239900
O	1.34691000	2.02474100	-1.78404700
C	-0.12103700	3.50598600	-0.71227400
O	-2.57439100	2.46667900	-1.35484600
C	-3.09621300	0.34500600	-2.21123600
H	0.81085200	-0.17126100	-2.38180100
H	-0.82016000	-0.87276200	-2.53804500
H	-0.38772900	0.61702400	-3.42313300
H	0.81455900	4.03261800	-0.48911800
H	-0.67388400	4.05194200	-1.49046200
H	-0.76633200	3.42682500	0.16238300
H	-2.99261200	0.48204200	-3.29945700
H	-2.87281900	-0.70335100	-1.99074900

H	-4.12473600	0.59224100	-1.92446400
H	-1.30419200	-3.35602100	-0.49975500
H	1.16692500	-3.52876200	-0.88692600

Ph-4-2134-R3-Nu3

C	-0.79545400	-0.05762800	0.00231100
C	-1.87829500	-1.09704900	-0.31526700
C	0.49881900	-0.79951800	0.31972300
C	-1.24049500	0.77069700	1.22240700
C	-3.28452100	-0.77847700	-0.06666500
C	-1.54170900	-2.32297300	-0.84858100
C	1.41485900	-0.21455700	1.28110400
C	0.77027100	-2.01784100	-0.30910500
O	-1.39736000	-0.00956800	2.29432000
O	-1.44768300	1.95362700	1.23675600
O	-4.14318600	-1.68508100	-0.59231900
O	-3.70439900	0.17890100	0.55219400
C	-0.21510100	-2.77212600	-0.94998100
O	2.47485200	-1.01152100	1.57949900
O	1.28721100	0.86271800	1.83354200
C	2.15414000	-2.59255000	-0.37958600
C	-1.80407600	0.66101700	3.47165100
C	-5.51664000	-1.43550900	-0.36392100
C	3.45064800	-0.44733900	2.43240400
O	2.99456900	-1.76420500	-1.01703200
O	2.47528200	-3.69615000	-0.02306000
H	-1.88436800	-0.10108300	4.25349000
H	-1.06028400	1.41925400	3.75367300
H	-2.77508300	1.15182400	3.31684500
H	-6.06462200	-2.24615300	-0.85552400
H	-5.81553100	-0.46667800	-0.78753600
H	-5.74252600	-1.42678300	0.71149900
H	4.22830200	-1.20796100	2.56110600
H	3.01681800	-0.18465200	3.40677900
H	3.88042100	0.46256600	1.98885000
C	4.34542000	-2.19007900	-1.08402100
H	4.42879200	-3.14801100	-1.61434900
H	4.75438200	-2.31023600	-0.07079800
H	4.89120100	-1.40884000	-1.62224900
C	-0.57325700	0.94898300	-1.23907300
C	0.58562200	1.92872400	-1.01059400
C	-1.81630100	1.70233900	-1.50381600
C	-0.32249800	0.15496900	-2.54125800
C	1.90469200	1.50669900	-1.22932900
C	0.37081200	3.24968000	-0.60128100
N	-2.77407900	2.25799500	-1.83134300
H	-1.20260200	-0.44469600	-2.80138900
H	-0.11183500	0.85693000	-3.35980400
H	0.53226900	-0.51936500	-2.42172200
C	2.97655200	2.37349100	-1.01419600
H	2.11224700	0.48358500	-1.54085100
C	1.44104800	4.11921200	-0.39828500

H	-0.64700300	3.59434000	-0.42350400
C	2.75123900	3.68373500	-0.59586600
H	3.99545200	2.01723200	-1.17933700
H	1.24689400	5.14461500	-0.07871200
H	3.58922200	4.36345100	-0.43141900
H	-2.35450800	-2.98310600	-1.15497100
H	0.03197400	-3.72535900	-1.41451100

Ph-4-2134-R3-Nu2

C	-0.40062800	0.78840300	-0.00805800
C	-1.75520900	0.14420500	0.22441600
C	0.16822900	1.16233400	1.36581000
C	-0.47788000	1.99694800	-0.93432600
C	-2.83910000	0.35833800	-0.71959900
C	-1.84826100	-0.77367800	1.27003300
C	0.94312900	2.39082800	1.56735400
C	0.00858600	0.25363500	2.38849900
O	-1.38798600	2.87195800	-0.53034000
O	0.22184200	2.14153800	-1.90287600
O	-4.03939800	-0.04569400	-0.23168900
O	-2.75457000	0.85228900	-1.82693000
C	-0.90155500	-0.81856300	2.30196300
C	-2.93431700	-1.81026800	1.29981700
O	1.75254200	2.31784100	2.65076500
O	0.90069100	3.39594400	0.89299200
C	-1.44192500	4.07524700	-1.27141300
C	-5.10412400	-0.08155100	-1.16117900
O	-3.66786200	-1.74302100	2.41448700
O	-3.09133900	-2.66407100	0.46552600
C	2.54455200	3.46422000	2.89635800
H	-2.23757300	4.68081100	-0.82619300
H	-0.47794400	4.59676200	-1.19759500
H	-1.66628600	3.86756900	-2.32666900
H	-5.98455600	-0.43440500	-0.61356700
H	-4.87691500	-0.76829900	-1.98933600
H	-5.29415400	0.91656800	-1.57786900
C	-4.69295200	-2.71809700	2.53594200
H	3.14537400	3.24195100	3.78451600
H	1.91693700	4.34776000	3.07801500
H	3.19856500	3.67246000	2.03842300
H	-5.40679800	-2.62792000	1.70581100
H	-4.26854000	-3.73090500	2.52528700
H	-5.19348000	-2.52464600	3.48945300
C	0.55444200	-0.29029800	-0.61270600
Br	-0.14267700	-1.25410300	-2.14259100
S	2.29705800	0.22050000	-0.99055900
H	0.66839800	-1.05678300	0.16374500
O	2.56317200	0.19275700	-2.42325400
O	2.63730200	1.41521800	-0.22177300
C	3.18899100	-1.13795900	-0.24682500
C	3.59784500	-2.20344900	-1.04307900
C	3.43276200	-1.09447700	1.12606200

C	4.27129300	-3.26587100	-0.43949500
H	3.39271500	-2.18660100	-2.11439200
C	4.10470400	-2.16359000	1.71492500
H	3.09689600	-0.23259500	1.70859200
C	4.52099400	-3.24458800	0.93325200
H	4.60330700	-4.11098700	-1.04373300
H	4.30802800	-2.15278000	2.78641500
H	5.04724400	-4.07844700	1.40017400
H	0.55230400	0.42325100	3.31977800
H	-0.99536900	-1.54583900	3.10699100

Ph-4-2134-R3-Nu4

C	0.78593300	0.23023700	-0.31856700
C	0.79454700	0.79784900	-1.77942300
C	-0.64422800	-0.11913600	0.09490700
C	-0.24209900	1.84562900	-2.11980400
H	1.81063600	1.17162200	-1.97818000
H	0.61666700	-0.07717300	-2.42130500
C	1.62314600	-1.05172600	-0.29586600
C	1.40378300	1.25962900	0.63301100
C	-1.45987600	0.96077600	0.61451600
C	-1.16614600	-1.33570000	-0.31732200
C	0.09140900	3.29688900	-1.90248200
C	-1.44145800	1.47019800	-2.58494200
C	3.05447800	-0.97681300	-0.07354900
C	1.03021100	-2.23932400	-0.67816400
O	1.40941800	0.81564700	1.89576200
O	1.86192400	2.32639900	0.31811500
O	-2.78358900	0.67570600	0.69733200
O	-1.05041300	2.05978400	0.94865100
C	-0.35762700	-2.37347700	-0.81847200
C	-2.62816100	-1.66674600	-0.24678100
H	0.15235300	3.50274900	-0.82540600
H	1.07604400	3.54292100	-2.32908200
H	-0.66931900	3.94775600	-2.35635000
H	-1.69409300	0.41307800	-2.70898800
H	-2.21201400	2.20741300	-2.82881500
O	3.68751100	-2.17621500	-0.17307000
O	3.69305500	0.02990000	0.18309400
C	1.97712300	1.69658800	2.84367200
C	-3.61946300	1.73360000	1.12115100
O	-2.99675900	-2.10600900	0.95840600
O	-3.37448500	-1.64007100	-1.19253800
C	5.08823100	-2.14765400	0.00598100
H	1.91793900	1.19397700	3.81475000
H	3.02474700	1.91054400	2.58989100
H	1.41797100	2.64246400	2.87268400
H	-4.64228800	1.34189000	1.11969100
H	-3.54338500	2.58936900	0.43530100
H	-3.34727900	2.07262300	2.13017300
C	-4.37744800	-2.39200700	1.11103700
H	5.43953500	-3.17715000	-0.12295900

H	5.35259700	-1.78489000	1.00935200
H	5.56954600	-1.49179500	-0.73322000
H	-4.97597400	-1.49321900	0.90644600
H	-4.69174600	-3.18797200	0.42252900
H	-4.51616600	-2.71181500	2.14832800
H	1.67098000	-3.10939900	-0.83303300
H	-0.80416700	-3.31699600	-1.13124700

Ph-4-2134-R4

C	-0.69524900	0.68690600	0.00017600
C	-1.38321500	-0.52629500	-0.00005400
C	0.69508500	0.68663100	-0.00010700
C	-0.69770000	-1.73078500	0.00004500
C	1.38326200	-0.52633200	0.00005300
C	0.69773800	-1.73087800	0.00043700
F	1.35429300	1.83403400	-0.00040200
F	2.71106000	-0.49162000	-0.00012600
F	-2.71103800	-0.49190700	-0.00056700
F	-1.35422800	1.83447600	0.00063600
H	-1.26645600	-2.66003200	0.00019600
H	1.26615200	-2.66029300	0.00063800

Ph-4-2134-R4-Nu1

C	0.33908500	-0.31775000	-0.36874400
C	0.85477700	-1.62362500	0.07741600
C	1.42565400	0.64581800	-0.17981600
C	2.16309300	-1.93652200	0.33650200
C	2.74612800	0.32660400	0.04475800
C	3.18519700	-0.96885800	0.31100400
F	1.12555100	1.95087600	-0.46282500
F	3.63418300	1.34188800	0.02869000
F	-0.08138300	-2.60554600	0.24723900
F	-0.01225600	-0.30719500	-1.79122600
C	-1.03486700	0.06353200	0.31472200
C	-2.22957700	-0.78263100	-0.18672500
C	-1.43044800	1.50487600	-0.06536100
C	-0.89838200	-0.11617400	1.83385200
O	-2.19246100	-1.56891400	-1.09361800
C	-3.52497100	-0.57383700	0.58346700
O	-2.01393700	1.71685100	-1.10313800
C	-1.17330700	2.61602000	0.92637500
H	-0.81935100	-1.18634700	2.06981700
H	0.01801200	0.37200900	2.19020900
H	-1.75353800	0.30164300	2.38169500
H	-3.49367500	-1.18074500	1.50100100
H	-3.67948300	0.47272200	0.87918400
H	-4.36173400	-0.91899300	-0.03444100
H	-1.97381900	2.59645700	1.68290100
H	-0.21416800	2.49913900	1.44165700
H	-1.21099900	3.57627600	0.39955700
H	2.37720900	-2.97404500	0.60395500

H	4.23358000	-1.18654400	0.49691900

Ph-4-2134-R4-Nu3

C	-0.83350400	0.78284000	-0.06290500
C	-0.79231300	0.65788600	1.39223600
C	-1.68300600	-0.27787600	-0.56771300
C	-1.64254200	-0.08330600	2.16892800
C	-2.55508300	-1.01740700	0.20760200
C	-2.59260700	-0.94728800	1.59481100
F	-1.70161200	-0.51546800	-1.91236100
F	-3.37081500	-1.87158700	-0.44084500
F	0.17424000	1.39595700	2.01209700
F	-1.42071900	2.14267600	-0.45256800
C	0.58814900	0.96005800	-0.72991900
C	1.49617900	-0.20502800	-0.34036500
C	1.16276600	2.24060400	-0.26624900
C	0.51378800	1.12421900	-2.26785600
C	1.22078000	-1.47819500	-0.86000000
C	2.57418900	-0.05533300	0.53525900
N	1.66107500	3.24821400	-0.00488200
H	-0.23764000	1.88521700	-2.50866700
H	1.49395000	1.44639100	-2.64532000
H	0.23880300	0.18505500	-2.75345100
C	2.01777700	-2.56907200	-0.52267400
H	0.37025700	-1.61494500	-1.53032300
C	3.37324700	-1.14953700	0.87369300
H	2.79263200	0.92487200	0.96126000
C	3.09975300	-2.40871600	0.34545800
H	1.78954000	-3.55213700	-0.93770500
H	4.21279400	-1.01168000	1.55707200
H	3.72397700	-3.26386500	0.60950100
H	-1.52878700	-0.00390100	3.25185700
H	-3.29831900	-1.53981200	2.17121600

Ph-4-2134-R4-Nu2

C	1.69545600	0.43148600	-0.18375500
C	2.39258900	-0.84585300	-0.26253000
C	2.38784600	1.25009900	0.81846800
C	3.63235800	-1.09507800	0.28848300
C	3.60760000	0.99060100	1.38389700
C	4.29228700	-0.21208100	1.13778400
F	1.73749100	2.40186200	1.15498200
F	4.20113400	-2.27816900	-0.01091000
F	1.88908100	-1.75862600	-1.13156200
F	1.71770400	1.06297500	-1.51739500
C	0.15457500	0.39299400	0.13598500
Br	-0.81839100	1.94606500	-0.52836700
S	-0.75978500	-1.11142000	-0.39048600
H	0.02256500	0.39721700	1.22436900
O	-0.78729300	-1.19753000	-1.84544500
O	-0.25728000	-2.21064400	0.43100600

C	-2.43248200	-0.80896500	0.16285200
C	-3.39729800	-0.42901100	-0.76668800
C	-2.73852300	-1.01590000	1.50646900
C	-4.70567600	-0.23386500	-0.32909400
H	-3.11394400	-0.29202800	-1.81082200
C	-4.04895100	-0.80753200	1.93334500
H	-1.96357700	-1.34785400	2.19930000
C	-5.02745000	-0.41707800	1.01731700
H	-5.47672200	0.06287200	-1.04102300
H	-4.30846800	-0.96038900	2.98148000
H	-6.05275800	-0.25957300	1.35523700
H	4.00740500	1.74192400	2.06831600
H	5.25964200	-0.45758500	1.56805600

Ph-4-2134-R4-Nu4

C	0.09066000	0.24454700	0.76010600
C	1.52660500	-0.28728400	0.89346900
C	-0.88081000	-0.71742900	0.27846400
C	2.15822000	-0.73550200	-0.40166300
H	2.12259800	0.51752800	1.34918200
H	1.48693000	-1.12269400	1.60375000
C	-0.08708100	1.48387900	0.01712600
C	-2.01035100	-0.37892400	-0.43502600
C	2.59970900	0.33926500	-1.35798900
C	2.31445900	-2.03433000	-0.67950300
C	-1.19711900	1.83010400	-0.70933300
C	-2.22866500	0.90058900	-0.94645700
H	3.26258700	1.05865900	-0.85282400
H	3.12628900	-0.08685700	-2.22247900
H	1.73175700	0.91168100	-1.71863100
H	1.97483600	-2.80385100	0.01783000
H	2.77264800	-2.36406600	-1.61574900
F	0.92626300	2.39311600	0.15859700
F	-2.90605900	-1.35712200	-0.68922800
F	-0.71478800	-2.01250900	0.67030300
F	-0.17450900	0.53745600	2.25578900
H	-3.13153700	1.13382900	-1.50589000
H	-1.23803800	2.83780600	-1.12950000

Ph-4-2134-R5

C	-0.69282700	0.26152000	-0.00336400
C	0.69309000	0.26134700	0.00353100
C	-1.37378800	-0.95370800	-0.00164100
C	1.37360200	-0.95407700	0.00164400
C	-0.69678600	-2.16262800	-0.00349600
C	0.69631500	-2.16280800	0.00333300
N	2.84818700	-0.98546600	-0.09238400
O	3.40241300	-1.90780600	0.44222800
O	3.36699200	-0.10339900	-0.72686600
N	1.41325600	1.55393200	0.04264600
O	2.16792100	1.71047400	0.96108000

O	1.15718200	2.33045200	-0.83733300
N	-1.41298800	1.55397300	-0.04283600
O	-1.15563000	2.33156000	0.83595300
O	-2.16898700	1.70960500	-0.96030400
N	-2.84833000	-0.98504200	0.09266300
O	-3.36702700	-0.10335000	0.72771800
O	-3.40261200	-1.90705700	-0.44251200
H	1.26527200	-3.09185100	0.00226100
H	-1.26580400	-3.09162500	-0.00263900

Ph-4-2134-R5-Nu1

C	-0.29018100	0.17972600	0.27774500
C	-0.57443000	1.65779400	0.05505800
C	1.21054700	0.05659100	0.39236800
C	0.32514000	2.55324400	-0.44615100
C	2.06495900	0.97157800	-0.22870400
C	1.66965900	2.20174700	-0.69382800
N	3.44523100	0.57113300	-0.57795400
O	3.56775600	-0.44503300	-1.21919500
O	4.33791900	1.32034900	-0.27801400
N	-1.89748100	2.15951000	0.35839400
O	-2.18237100	3.29804400	0.05714600
O	-2.66927800	1.40432100	0.92317300
N	-0.86036800	-0.17568200	1.70087100
O	-0.42160600	0.48422800	2.60675800
O	-1.66719100	-1.05070000	1.80251300
N	1.75704100	-0.98812300	1.15366000
O	1.00229100	-1.87140000	1.55310000
O	2.95430500	-0.98088200	1.40694500
C	-0.92133100	-0.74758600	-0.86422400
C	-2.42512000	-0.38935200	-1.10651700
C	-0.67687500	-2.26617800	-0.63507400
C	-0.19959100	-0.35809800	-2.17570800
O	-2.64825300	0.55728700	-1.82826100
C	-3.57381300	-1.18381500	-0.54510200
O	-1.54297500	-3.00187000	-0.23538900
C	0.64425700	-2.85593300	-1.09389200
H	-0.38694600	0.69437500	-2.40857100
H	0.88008600	-0.53061600	-2.11676200
H	-0.60434200	-0.95736300	-3.00353800
H	-4.48033300	-0.58230500	-0.67902400
H	-3.66143000	-2.12117200	-1.11218400
H	-3.41176400	-1.45436200	0.49984300
H	0.54277000	-3.10295300	-2.16264800
H	1.50780900	-2.18904000	-0.99479500
H	0.80983900	-3.78306000	-0.53491300
H	-0.03207700	3.56178700	-0.65016000
H	2.36132600	2.86377700	-1.20808300

Ph-4-2134-R5-Nu3

C	-0.60860400	-0.70514500	0.00396100

C	-0.97383400	-0.03924800	1.32144300
C	0.85795700	-1.06298400	0.09663400
C	-0.07608900	0.59328200	2.13127200
C	1.72496200	-0.32105700	0.90655000
C	1.31293500	0.54034100	1.89324000
N	3.18109100	-0.32595000	0.63745100
O	3.53267900	-0.10240100	-0.49464700
O	3.91647000	-0.47352500	1.58041900
N	-2.36795600	0.05224000	1.70106500
O	-2.69408100	0.86488300	2.54351800
O	-3.15339800	-0.70255700	1.16224800
N	-1.40722100	-2.05403500	-0.08257300
O	-1.20158700	-2.83150100	0.80963600
O	-2.16046200	-2.21967200	-0.99903100
N	1.35250100	-2.21059200	-0.55389500
O	0.63910500	-2.78195000	-1.37135700
O	2.46940800	-2.61661600	-0.26074300
C	-0.93840900	0.21981500	-1.25432400
C	-0.26056200	1.58227100	-0.99358400
C	-2.38895400	0.46789200	-1.39430500
C	-0.45771400	-0.37403900	-2.59404800
C	1.07085800	1.78948700	-1.36866800
C	-0.93644000	2.60934500	-0.32372100
N	-3.49364600	0.74102500	-1.58303400
H	-1.01960200	-1.27902100	-2.83929100
H	-0.59821000	0.38073200	-3.37920900
H	0.60405600	-0.63540500	-2.54252200
C	1.71611300	2.98839000	-1.06585400
H	1.63577400	1.00342000	-1.86699200
C	-0.29523700	3.81024100	-0.02699900
H	-1.97479400	2.46965500	-0.01609900
C	1.03655500	4.00297600	-0.39428400
H	2.75965300	3.12115000	-1.35539700
H	-0.84108400	4.59614700	0.49685300
H	1.54113100	4.94122800	-0.15875900
H	-0.47137100	1.15095100	2.97917500
H	2.02476000	1.12963900	2.46493100

Ph-4-2134-R5-Nu2

C	-1.15689200	0.59453900	0.18000800
C	-1.86401100	-0.74046200	0.23308500
C	-1.78081500	1.35659300	-0.98153100
C	-2.27987800	-1.34525700	-0.94365200
C	-2.24343200	0.71459300	-2.10339500
C	-2.40874300	-0.67626400	-2.14981400
N	-2.56834000	-2.79617500	-0.99702300
O	-3.68739600	-3.11917300	-1.30519500
O	-1.64370700	-3.54206100	-0.80884900
N	-1.96906400	-1.42552200	1.46932900
O	-2.66242300	-2.42625100	1.52362800
O	-1.37511200	-0.96715500	2.43086000
N	-1.44003500	1.35337400	1.50308700

O	-2.60129300	1.50119200	1.77543300
O	-0.50306000	1.73235300	2.14007100
N	-1.79512600	2.79716900	-0.99219700
O	-1.45787200	3.38772600	0.01649100
O	-2.16072400	3.36776600	-2.00258900
C	0.37182100	0.41138300	-0.06748000
Br	1.37584100	2.04335000	-0.33190300
S	1.34027200	-0.74727100	1.01176000
H	0.42028500	-0.10896100	-1.03514100
O	1.64449400	-0.15303700	2.30160200
O	0.67506800	-2.04315900	0.90612000
C	2.86636000	-0.88944200	0.09752000
C	3.98341600	-0.17385100	0.52193900
C	2.90794900	-1.76185000	-0.98872600
C	5.17662200	-0.32631700	-0.18101400
H	3.90774200	0.48477400	1.38795500
C	4.10653700	-1.89879600	-1.68664800
H	2.01994900	-2.33275800	-1.26630400
C	5.23448300	-1.18180500	-1.28319000
H	6.06448600	0.22321300	0.13336700
H	4.16236100	-2.57514800	-2.54012200
H	6.17147400	-1.29576300	-1.83014200
H	-2.51802100	1.33400100	-2.95602500
H	-2.72342200	-1.19616300	-3.05072000

Ph-4-2134-R5-Nu4

C	-0.69405600	0.41780200	0.05360200
C	-1.10141600	0.49778700	1.54324400
C	-1.31835200	-0.75431800	-0.66795200
C	-0.51710600	-0.64845000	2.33971000
H	-0.76025100	1.45960700	1.94005400
H	-2.19661800	0.48338200	1.59098200
C	0.79339700	0.37458900	-0.20476300
C	-0.63283700	-1.80387200	-1.21648400
C	0.82773700	-0.41984000	2.97563100
C	-1.17768500	-1.80493400	2.45855100
C	1.42767700	-0.75007700	-0.72248300
C	0.77031400	-1.85271800	-1.23117100
H	0.81114000	0.49024900	3.59500800
H	1.11938900	-1.27015100	3.60639700
H	1.61090800	-0.28057800	2.21364700
H	-2.15490600	-1.95173400	1.98955200
H	-0.76103300	-2.64176200	3.02445800
N	2.89918800	-0.89094200	-0.65366200
O	3.39019600	-0.96344400	0.44481200
O	3.48939100	-1.01404200	-1.69565300
N	-2.75349000	-0.81514100	-0.71472700
O	-3.29993900	-1.85579500	-1.03213000
O	-3.37125000	0.20090600	-0.43953700
N	-1.22534900	1.74280600	-0.64787800
O	-0.96838500	1.84564100	-1.82163200
O	-1.83440300	2.54347300	0.00167000

N	1.54155400	1.53469000	0.08666700
O	0.97259600	2.45903100	0.65629200
O	2.71326600	1.59697400	-0.25953600
H	-1.21917000	-2.61786400	-1.64070700
H	1.31939400	-2.70881300	-1.61378700

Ph-4-2135-R1

C	-0.83137700	-0.00013900	-0.05814500
C	-0.11474500	-1.20982100	-0.01914200
C	-0.11592600	1.21014400	-0.01923500
C	1.27879600	-1.20194500	-0.03802500
C	1.27770400	1.20367100	-0.03821900
C	1.96868500	0.00123500	-0.06984900
C	-0.77070800	-2.58202100	0.08980600
C	3.47822900	0.00122800	-0.05464400
C	-0.77320500	2.58171900	0.08976300
C	-2.35798500	-0.00099900	-0.13576300
F	-2.80698800	1.06195300	-0.79600700
F	-2.80568100	-1.06424700	-0.79647100
F	-2.91097300	-0.00156900	1.07104300
F	-1.78562200	2.56768000	0.95429800
F	-1.22034800	3.03155800	-1.07749300
F	0.10634500	3.47873600	0.53696700
F	3.96814400	-1.07201100	-0.67095500
F	3.94564200	-0.00799100	1.19388600
F	3.96847300	1.08301900	-0.65539600
F	-1.21762300	-3.03206500	-1.07747400
F	0.10974100	-3.47830700	0.53670200
F	-1.78300400	-2.56904900	0.95446800
H	1.82413000	2.14589300	-0.02086700
H	1.82611400	-2.14366600	-0.02051800

Ph-4-2135-R1-Nu1

C	0.45481800	0.02302500	-0.27925500
C	-0.39397700	1.31240600	-0.29445500
C	-0.52799800	-1.17102600	-0.32211400
C	-1.73070100	1.33257200	0.00641100
C	-1.85829100	-1.05090600	-0.02091300
C	-2.49318200	0.17772100	0.22781600
C	0.22905100	2.65951000	-0.48115800
C	-3.94147000	0.23449900	0.48585900
C	-0.10334800	-2.54280300	-0.76084900
C	1.26461400	-0.01678000	-1.60674800
F	0.41278200	-0.14601000	-2.63547600
F	1.95269800	1.09290000	-1.86630200
F	2.10948000	-1.04378700	-1.67874900
F	1.10681300	-2.92844700	-0.31824400
F	-0.05052000	-2.69366900	-2.09896200
F	-0.95582400	-3.49187500	-0.33238800
F	-4.71602700	0.05475700	-0.61340000
F	-4.32700200	1.41504800	0.99957600

F	-4.35359500	-0.72079000	1.34413100
F	0.34242900	3.06194100	-1.76141500
F	-0.48141100	3.63390300	0.11987900
F	1.46809600	2.75414400	0.04622000
C	1.38261400	-0.00583000	1.07824600
C	2.74847300	0.71835800	0.97956600
C	1.80588900	-1.42261100	1.58723200
C	0.51638600	0.58588900	2.20756800
O	3.49225400	0.54423400	0.05113100
C	3.19258700	1.54399400	2.16908600
O	2.95201700	-1.79327700	1.51924000
C	0.79713600	-2.25781400	2.35111000
H	0.38747600	1.66613400	2.07927300
H	-0.48249900	0.13015300	2.20059900
H	0.97204700	0.41137900	3.19142400
H	2.49762700	2.36797800	2.37124900
H	3.23579300	0.91050100	3.06752000
H	4.19138200	1.93953900	1.95500000
H	0.82210400	-1.93944000	3.40419200
H	-0.22917500	-2.14611200	1.98830600
H	1.11561000	-3.30464100	2.29746200
H	-2.22699500	2.30146200	0.07919400
H	-2.46441300	-1.95885400	-0.00543200

Ph-4-2135-R1-Nu3

C	-0.97111700	0.15434900	-0.13405600
C	-0.03819200	-0.58923600	-1.10825900
C	-0.34442100	1.53308300	0.12099500
C	1.28037700	-0.23949500	-1.27143100
C	0.98220600	1.80560500	-0.09626300
C	1.85568400	0.91843000	-0.73447300
C	-0.41971200	-1.85444200	-1.82171500
C	3.29640800	1.20296200	-0.84917900
C	-1.08925600	2.73993100	0.60349300
C	-2.38853700	0.35035400	-0.76838000
F	-2.43335500	1.46472600	-1.50336500
F	-2.73873700	-0.64512900	-1.58228900
F	-3.37569600	0.43075800	0.13180600
F	-2.36359500	2.53263500	0.94830000
F	-1.11390600	3.73462500	-0.30701300
F	-0.50141400	3.29545000	1.69155100
F	3.55650200	2.52014400	-0.96362300
F	3.86135600	0.59812800	-1.90854800
F	4.03104300	0.80146200	0.21780600
F	-0.94760700	-1.66587300	-3.04911900
F	0.64636000	-2.65143400	-2.03441500
F	-1.29966700	-2.61893600	-1.15420300
C	-1.13015200	-0.67687100	1.28556700
C	0.21794000	-1.33078000	1.61068300
C	-2.16621000	-1.72429900	1.21059600
C	-1.57280500	0.22066300	2.46244500
C	1.24848900	-0.56012800	2.16240900

C	0.46765900	-2.67990200	1.33437000
N	-3.00292200	-2.51874200	1.24109800
H	-2.59209600	0.59075300	2.31642700
H	-1.53109700	-0.36567800	3.39007000
H	-0.90023500	1.07910300	2.56921000
C	2.50343400	-1.11696800	2.40247900
H	1.08456600	0.49336100	2.38658800
C	1.72103900	-3.23885200	1.57896000
H	-0.32171600	-3.30123000	0.91057300
C	2.74714000	-2.45743200	2.10806300
H	3.29729500	-0.49235200	2.81403500
H	1.89405800	-4.29175700	1.35071400
H	3.73041900	-2.89210100	2.29462700
H	1.91177300	-0.89106200	-1.87649600
H	1.35608600	2.79130500	0.19675200

Ph-4-2135-R1-Nu2

C	-0.61806000	0.12961200	0.76712500
C	-1.27007800	-1.25317900	0.67643700
C	-1.55109400	1.15418400	0.11866400
C	-2.34951000	-1.51275600	-0.13361400
C	-2.62955400	0.80435800	-0.64432200
C	-3.04164200	-0.52414600	-0.83957300
C	-0.72250500	-2.43142300	1.42234300
C	-4.24535900	-0.83707200	-1.63131200
C	-1.24639300	2.61383200	0.22311900
C	-0.41591200	0.53326000	2.25194300
F	-1.59961300	0.75702000	2.82322200
F	0.18781200	-0.39687400	2.98354300
F	0.30201300	1.65407400	2.37065800
F	0.05535200	2.88351700	0.06740700
F	-1.59903400	3.16252800	1.40513800
F	-1.89567400	3.33623800	-0.70459100
F	-5.41123800	-0.69523200	-0.95451300
F	-4.25123000	-2.10507800	-2.07734400
F	-4.37796100	-0.03920400	-2.70772300
F	-1.17731800	-2.54097400	2.68616000
F	-1.05095300	-3.59468000	0.83549800
F	0.62518500	-2.44609200	1.52250000
C	0.81172200	0.00176900	0.00699000
Br	0.65956300	0.43803100	-1.87317000
S	2.41663900	0.69693400	0.62726100
H	1.06810000	-1.05992900	0.01194500
O	2.55763100	2.12233200	0.37055200
O	2.59368100	0.16612700	1.97610000
C	3.59432700	-0.16053900	-0.40932200
C	4.22403100	0.53113300	-1.44042300
C	3.88699300	-1.49240400	-0.11992000
C	5.16699700	-0.14171000	-2.21549300
H	3.97170500	1.57672800	-1.62113300
C	4.82522100	-2.15561700	-0.90832800
H	3.39254000	-1.99270200	0.71505500

C	5.46161100	-1.48076500	-1.95218900
H	5.67326300	0.38112100	-3.02770100
H	5.06650700	-3.19877800	-0.70151800
H	6.19827300	-2.00317500	-2.56420000
H	-3.19096500	1.60433900	-1.12981300
H	-2.69168300	-2.54570100	-0.21338900

Ph-4-2135-R1-Nu4

C	0.93087200	0.02807100	0.03397900
C	1.62989800	-0.01800700	1.44365400
C	0.04193200	1.27748900	-0.12169400
C	0.66725300	-0.12474500	2.60238300
H	2.32289200	-0.86969600	1.45736400
H	2.22682300	0.89511100	1.54859000
C	0.06979500	-1.22117600	-0.23425100
C	-1.32282800	1.21325000	-0.18843300
C	0.31031000	-1.50724500	3.07938000
C	0.17043600	0.97423700	3.18117300
C	-1.29719800	-1.18008800	-0.29682100
C	-2.04437000	0.00768000	-0.23249100
H	1.21902700	-2.06430700	3.35791700
H	-0.36018800	-1.46924300	3.94856200
H	-0.18068600	-2.08370000	2.28155800
H	0.44986500	1.96987200	2.82867500
H	-0.53372200	0.90768100	4.01460500
C	0.63084100	2.65038500	-0.15150300
C	-3.50610600	-0.00178200	-0.38477800
C	0.68779100	-2.57538400	-0.35837300
C	2.07910800	0.09005500	-0.99370200
F	1.63031100	0.17731200	-2.24511700
F	2.88948900	1.13818300	-0.78831100
F	2.87609400	-0.98603800	-0.93490800
F	1.69154100	-2.80955500	0.51233800
F	1.20566200	-2.83617100	-1.57913800
F	-0.20173000	-3.56365000	-0.14424800
F	-3.94611100	0.05471800	-1.67026200
F	-4.09891500	1.04611000	0.21998600
F	-4.07321700	-1.11677500	0.11607700
F	1.18954700	2.98043600	-1.33738700
F	-0.28580900	3.60736600	0.07528700
F	1.59952100	2.86490100	0.76570300
H	-1.83586800	-2.12338200	-0.40277200
H	-1.88199700	2.14970400	-0.21582200

Ph-4-2135-R2

C	0.00020700	0.93308300	0.00000000
C	0.00024300	0.22440700	1.21482500
C	0.00024300	0.22440700	-1.21482500
C	0.00028300	-1.17129700	1.21604400
C	0.00028300	-1.17129700	-1.21604400
C	0.00004100	-1.86115200	0.00000000

C	-0.00019500	0.94155000	2.46472100
N	-0.00053200	1.51251500	3.46676700
C	-0.00011100	-3.30357300	0.00000000
N	-0.00013300	-4.45699500	0.00000000
C	-0.00019500	0.94155000	-2.46472100
N	-0.00053200	1.51251500	-3.46676700
C	0.00026000	2.37179200	0.00000000
N	0.00026000	3.52522500	0.00000000
H	0.00010500	-1.71482600	2.16024900
H	0.00010500	-1.71482600	-2.16024900

Ph-4-2135-R2-Nu1

C	-0.33543200	0.54923500	0.48874700
C	0.80536800	1.51561500	0.13556400
C	0.25096700	-0.52418700	1.40957800
C	2.08448800	1.04032500	-0.02449100
C	1.53821500	-0.95553500	1.22301100
C	2.44614100	-0.26672400	0.37518700
C	0.52290700	2.88839500	-0.11230200
N	0.28580400	4.00439700	-0.32527000
C	3.76019000	-0.76168600	0.14846200
N	4.82844800	-1.17001600	-0.05018700
C	-0.50570800	-1.08609100	2.48140700
N	-1.05816600	-1.54252400	3.39252700
C	-1.37731100	1.31668700	1.20595000
N	-2.15160500	1.97684800	1.74882000
C	-1.00636700	-0.04593300	-0.83771100
C	-2.21920100	-0.92620700	-0.48079600
C	-0.01137000	-0.98847700	-1.54538500
C	-1.48402900	1.08602000	-1.75809300
O	-2.59032800	-1.06710100	0.65594200
C	-2.98190700	-1.54795700	-1.63001400
O	0.01788300	-2.15066200	-1.21310200
C	0.82507900	-0.46865200	-2.68626100
H	-2.22650900	1.71368800	-1.24342500
H	-0.65092500	1.72953000	-2.06546900
H	-1.94806700	0.68258900	-2.66825500
H	-3.78049000	-0.84974800	-1.92452100
H	-2.35535000	-1.74577300	-2.50787600
H	-3.45065900	-2.47518800	-1.28103200
H	0.18185400	-0.39296400	-3.57810400
H	1.22875800	0.53043500	-2.48568500
H	1.63185700	-1.18186800	-2.88928200
H	2.85072500	1.71144800	-0.41923400
H	1.89481900	-1.80993100	1.80180700

Ph-4-2135-R2-Nu3

C	1.18537200	0.39758700	0.14023700
C	0.65143700	0.09267800	1.53798800
C	0.52110900	1.67861900	-0.35923700
C	-0.60831100	0.48824700	1.91488500

C	-0.73661100	2.03656500	0.06634900
C	-1.38238200	1.38470400	1.14017600
C	1.44768900	-0.66287100	2.44816200
N	2.09033300	-1.26211600	3.20514000
C	-2.70045700	1.74715700	1.53369000
N	-3.77914900	2.03660600	1.84938000
C	1.19454500	2.48561100	-1.32003000
N	1.74709300	3.13556200	-2.10679200
C	2.65013500	0.59620100	0.20743300
N	3.79212300	0.75057200	0.25419100
C	0.92306300	-0.83061500	-0.88664400
C	-0.57483500	-1.12291900	-0.96057700
C	1.62713400	-2.01245500	-0.35193300
C	1.54007200	-0.53302400	-2.26440400
C	-1.38252900	-0.45868600	-1.89085100
C	-1.17611800	-2.01142400	-0.06072200
N	2.20808700	-2.92988700	0.03800600
H	2.62799300	-0.40922900	-2.18058700
H	1.32925900	-1.36389200	-2.95022800
H	1.12393900	0.38592100	-2.69183700
C	-2.76237700	-0.65765300	-1.90130300
H	-0.94342100	0.23622900	-2.60610400
C	-2.55486600	-2.21195700	-0.07178100
H	-0.56059400	-2.54802600	0.66377500
C	-3.35414000	-1.52914500	-0.98795600
H	-3.37692600	-0.12487900	-2.62836500
H	-3.00492800	-2.90461400	0.64074500
H	-4.43449000	-1.68096700	-0.99493800
H	-1.22150600	2.89251900	-0.40799200
H	-0.99631800	0.14204400	2.87518700

Ph-4-2135-R2-Nu2

C	-1.23462000	0.05939300	-0.09062900
C	-2.04775100	-1.13274100	-0.60952700
C	-2.22625700	1.23168700	-0.11210700
C	-3.26987300	-1.35085900	-0.02206800
C	-3.44894700	0.99809800	0.48228700
C	-3.89326200	-0.32277400	0.73506100
C	-1.61600400	-1.93159900	-1.70360600
N	-1.30021600	-2.55784300	-2.62718200
C	-5.12854800	-0.55828800	1.40638500
N	-6.12817800	-0.75128400	1.96159100
C	-2.00056100	2.48417300	-0.74627100
N	-1.89801700	3.51210000	-1.27447300
C	-0.87078400	-0.17493900	1.34271300
N	-0.59833600	-0.31402800	2.45509200
C	0.07621600	0.28009900	-0.86778000
Br	1.00722100	1.89423400	-0.35300900
S	1.26832700	-1.18069900	-0.75134000
H	-0.11521200	0.34272500	-1.94700600
O	0.59936200	-2.25153100	-0.01897100
O	1.72273600	-1.36985900	-2.12391400

C	2.67703200	-0.69354400	0.22246200
C	2.64289900	-0.88418700	1.60202300
C	3.80464300	-0.21109800	-0.43922300
C	3.77577500	-0.55087700	2.34289400
H	1.74646500	-1.27941800	2.08089200
C	4.92949600	0.11174300	0.31504500
H	3.79159200	-0.09646800	-1.52358800
C	4.91134000	-0.05372600	1.70182900
H	3.77171000	-0.68704800	3.42471800
H	5.82344900	0.49101300	-0.18092100
H	5.79490300	0.20216900	2.28831500
H	-4.12905700	1.83512200	0.65413300
H	-3.81812300	-2.26996600	-0.23882100

Ph-4-2135-R2-Nu4

C	-0.64555900	0.33327500	-0.56793900
C	-1.89411500	0.36067700	0.37021300
C	-0.05199700	-1.07498000	-0.66724100
C	-1.68982300	-0.32670400	1.69889100
H	-2.16697300	1.41871100	0.52780300
H	-2.73366500	-0.11574800	-0.15685700
C	0.44904400	1.31974400	-0.14299100
C	1.26707700	-1.35920500	-0.43723100
C	-0.59742600	0.18350400	2.60019900
C	-2.47333800	-1.35122400	2.05131400
C	1.75836300	0.96431300	0.05029900
C	2.21858300	-0.36721300	-0.08602600
H	-0.61895400	1.28226800	2.66815000
H	-0.69351200	-0.23960100	3.60892800
H	0.39266200	-0.09103700	2.20297700
H	-3.24393000	-1.73449800	1.37824300
H	-2.35920900	-1.84113200	3.02153200
C	-0.95976700	-2.12057100	-1.00850100
N	-1.72218800	-2.94578600	-1.29806700
C	3.57956300	-0.70201000	0.13971800
N	4.69326100	-0.97620500	0.32418900
C	0.04559200	2.67056200	0.06164100
N	-0.31432700	3.76114000	0.23003200
C	-1.13012500	0.74484800	-1.91660400
N	-1.49813800	1.06470400	-2.96327600
H	2.46491900	1.74212800	0.34706400
H	1.59199200	-2.39816500	-0.52244800

Ph-4-2135-R3

C	-0.82670600	0.15449600	0.10519600
C	0.17868900	1.13275100	0.07559500
C	-0.46607700	-1.20136100	0.06411400
C	-2.28000700	0.55368000	0.25865300
C	-0.19652300	2.58475900	0.09443200
C	1.52282500	0.75848100	0.01251900
C	-1.53340300	-2.25487600	0.06006700

C	0.87919700	-1.56611400	0.00329700
O	-2.85587900	0.74365200	-0.91680500
O	-2.81428900	0.67393300	1.32477100
O	0.86609600	3.38136800	0.09690000
O	-1.33032700	2.99033100	0.10273900
C	1.87266800	-0.58978000	-0.02012300
O	-1.03099300	-3.48248000	0.11614600
O	-2.71341300	-2.02085000	0.00558300
C	-4.22392600	1.12914300	-0.86810900
C	0.60094700	4.78140700	0.10844400
C	3.29896800	-1.03868300	-0.08638200
C	-1.97615800	-4.54878400	0.10354500
H	-4.55032200	1.24114900	-1.90580800
H	-4.81491500	0.35576700	-0.36004800
H	-4.32691200	2.07932100	-0.32757700
H	1.57576200	5.27610700	0.10865800
H	0.03109200	5.05187200	1.00638300
H	0.02452200	5.06570100	-0.78103200
O	4.15218400	-0.02005400	-0.10089600
O	3.63496400	-2.19409700	-0.12270400
H	-1.39408200	-5.47245700	0.15776700
H	-2.56847000	-4.51984600	-0.81985000
H	-2.65099100	-4.46694400	0.96494900
C	5.53424400	-0.35942100	-0.16334400
H	5.74498900	-0.92723300	-1.07861900
H	5.81612800	-0.96600300	0.70676200
H	6.08182300	0.58677900	-0.16464000
H	2.29507300	1.52527700	-0.01139600
H	1.16112200	-2.61809400	-0.02884300

Ph-4-2135-R3-Nu1

C	-0.51272700	-0.07421700	0.08607200
C	0.66809200	-1.01157900	0.35105400
C	-0.01704000	1.36180400	0.30099300
C	-1.64209300	-0.36945400	1.08682700
C	0.37877100	-2.38468600	0.74337800
C	1.97274100	-0.60656100	0.18676000
C	-0.96750600	2.40608400	0.71046200
C	1.29568200	1.69718000	0.12243800
O	-1.18482300	-0.35835900	2.34053000
O	-2.79389200	-0.56784500	0.81062600
O	1.47644500	-3.15551800	0.91448000
O	-0.73556300	-2.85274000	0.89108500
C	2.32858800	0.73672900	-0.03920400
O	-0.52575200	3.65637700	0.46618500
O	-2.03746400	2.21999200	1.25156500
C	-2.16347700	-0.56586200	3.34136700
C	1.23465600	-4.50392400	1.27002400
C	3.69574400	1.18126100	-0.22155400
C	-1.39570400	4.70154300	0.85630000
H	-1.64247600	-0.51740100	4.30291400
H	-2.93348200	0.21563600	3.28414500

H	-2.63921400	-1.54841500	3.21534200
H	2.21659000	-4.97332200	1.38880700
H	0.66226700	-5.02074400	0.48692000
H	0.66998500	-4.56668900	2.21016500
O	4.58870700	0.15943400	-0.26689200
O	4.06732400	2.33571000	-0.33259200
H	-0.89445500	5.63779400	0.58995200
H	-1.59084900	4.67141700	1.93725500
H	-2.35570400	4.62276100	0.32745200
C	5.94228200	0.53196100	-0.42911200
H	6.27914200	1.17052700	0.39972900
H	6.09061100	1.08240400	-1.36882400
H	6.52196100	-0.39715600	-0.44443200
C	-1.10631900	-0.27025300	-1.41581800
C	-2.31100600	0.68153000	-1.65390000
C	-1.44169100	-1.75505200	-1.68375300
C	-0.03775700	0.18452700	-2.42872700
O	-2.08906900	1.87404200	-1.66006200
C	-3.70066000	0.19833100	-1.99158700
O	-2.55445000	-2.20806900	-1.56867800
C	-0.34262900	-2.65710800	-2.21939100
H	0.13579700	1.25872600	-2.30808300
H	0.90974900	-0.34487800	-2.27539700
H	-0.38210900	0.00139600	-3.45836100
H	-4.31841300	1.08590300	-2.17189700
H	-3.67583100	-0.43464600	-2.88921100
H	-4.09460600	-0.41301900	-1.17590700
H	-0.24677100	-2.47840400	-3.30176700
H	0.63869000	-2.47001000	-1.76960400
H	-0.65270500	-3.69601600	-2.05938500
H	1.58084000	2.74844100	0.18484100
H	2.76159600	-1.35200900	0.28960200

Ph-4-2135-R3-Nu3

C	0.04627500	0.52105400	0.12232800
C	-1.43318300	0.90597500	0.04658900
C	0.15115700	-0.92197100	0.61796500
C	0.77249600	1.45824900	1.10293500
C	-1.79952400	2.32047600	0.12900200
C	-2.41236300	-0.04379900	-0.10935500
C	1.39260300	-1.35121300	1.27101900
C	-0.87371800	-1.81346300	0.44180400
O	0.24037500	1.36229400	2.32234000
O	1.68692200	2.19089100	0.84040500
O	-3.09451300	2.56507600	-0.17471200
O	-1.05632000	3.22640800	0.44842600
C	-2.16021200	-1.43022300	-0.00788700
O	1.51916100	-2.69283300	1.35846700
O	2.25287100	-0.61483700	1.70875300
C	0.86269500	2.15802300	3.31352900
C	-3.50169700	3.91717700	-0.08110500
C	-3.19043000	-2.44070600	-0.15835900

C	2.71340200	-3.15597300	1.96067400
H	0.32872600	1.96624500	4.24963300
H	1.92072000	1.87812800	3.41213100
H	0.79707300	3.22217500	3.04775300
H	-4.55872200	3.94276100	-0.36527300
H	-2.91216400	4.55059000	-0.75798900
H	-3.37724900	4.29562500	0.94305700
O	-4.39840100	-1.92467300	-0.49570200
O	-3.04499300	-3.64085500	-0.01305100
H	2.66451200	-4.24971100	1.94470200
H	2.79879500	-2.79715800	2.99568800
H	3.58928000	-2.80551500	1.39558500
C	-5.44931700	-2.85870500	-0.64138700
H	-5.62781900	-3.40212700	0.29725700
H	-5.21888000	-3.59334600	-1.42545800
H	-6.34047900	-2.28478600	-0.91651300
C	0.74180800	0.64195900	-1.32793000
C	2.15872600	0.05163300	-1.34936600
C	0.79069200	2.05479900	-1.75947600
C	-0.13472400	-0.06047800	-2.38918700
C	2.33391400	-1.32824800	-1.52358100
C	3.29763100	0.84951600	-1.19291900
N	0.78022000	3.11745300	-2.21059000
H	-1.07341800	0.48610800	-2.53411400
H	0.40704300	-0.10418800	-3.34361500
H	-0.38083700	-1.07912200	-2.06776800
C	3.60676300	-1.89819600	-1.49345300
H	1.46999500	-1.97742900	-1.65982400
C	4.57123700	0.28364900	-1.17934500
H	3.18019400	1.92394600	-1.05901800
C	4.73210000	-1.09507800	-1.31632000
H	3.71488100	-2.97764400	-1.61498500
H	5.44372200	0.92693400	-1.05324600
H	5.72890400	-1.53903600	-1.29616200
H	-0.71618800	-2.85624900	0.72203300
H	-3.43842100	0.29312600	-0.25620300

Ph-4-2135-R3-Nu2

C	0.89341300	-0.44144600	0.41403800
C	1.92774200	0.60136700	0.00092000
C	0.12231200	0.10647100	1.62185900
C	1.51164000	-1.80397900	0.70869100
C	3.17074900	0.17667800	-0.64120000
C	1.57915500	1.92858300	0.06596900
C	-0.33853600	-0.77427400	2.70668000
C	-0.18833800	1.43919100	1.63513300
O	2.50475400	-1.71647600	1.58168300
O	1.12189200	-2.83170100	0.21879100
O	3.97639800	1.21084300	-0.97124500
O	3.49523400	-0.96737900	-0.88570100
C	0.42627100	2.36964100	0.75673100
O	-1.40604200	-0.26461300	3.36031800

O	0.15324600	-1.82832000	3.03922900
C	3.08202400	-2.94940100	1.96760600
C	5.17175100	0.86714900	-1.64616900
C	0.02148700	3.76481900	0.81665300
C	-1.91893000	-1.06692500	4.40767300
H	3.87381300	-2.71205400	2.68497000
H	2.32071100	-3.58753400	2.43622900
H	3.50271800	-3.46595100	1.09360300
H	5.69878400	1.80670800	-1.84145900
H	4.95400600	0.35226300	-2.59224000
H	5.79428800	0.20596600	-1.02802500
O	0.76513600	4.57018200	0.02188000
O	-0.88558200	4.21571700	1.49023400
H	-2.75961100	-0.51395000	4.83910900
H	-1.15405100	-1.25151500	5.17421000
H	-2.26224200	-2.03383600	4.01481000
C	0.42482600	5.94281200	0.04410100
H	0.53019600	6.35875500	1.05580900
H	-0.61227000	6.09621100	-0.28528900
H	1.11433700	6.44570100	-0.64194200
C	-0.14023300	-0.56358100	-0.74910900
Br	0.60512000	-0.87866800	-2.51184300
S	-1.56147400	-1.74403900	-0.50973500
H	-0.62432700	0.41759200	-0.82813800
O	-1.52090200	-2.81871100	-1.49306500
O	-1.70047100	-2.07599800	0.90539400
C	-2.92940700	-0.67194200	-0.92058900
C	-3.46821100	-0.71191500	-2.20287200
C	-3.40704200	0.19118600	0.06607700
C	-4.52271800	0.14936500	-2.50799700
H	-3.06686600	-1.41244000	-2.93655400
C	-4.45730900	1.04812000	-0.25549500
H	-2.95455400	0.18197600	1.06156900
C	-5.01158900	1.02547500	-1.53818800
H	-4.96415700	0.13420700	-3.50521900
H	-4.84689700	1.73395200	0.49761500
H	-5.83491500	1.69817900	-1.78282900
H	2.23995400	2.66794300	-0.38697800
H	-0.86816900	1.82309800	2.39780500

Ph-4-2135-R3-Nu4

C	0.73929700	0.18457300	0.27869000
C	0.96461600	-0.04447600	1.81110100
C	-0.10063900	-0.94537400	-0.31417500
C	1.36219800	-1.43422500	2.26073600
H	1.70412400	0.70004400	2.14480800
H	-0.00051800	0.19703800	2.27923800
C	-0.02925600	1.49994800	0.09956500
C	2.09542200	0.28455200	-0.42833100
C	0.58201400	-2.17273100	-0.70615500
C	-1.46705500	-0.87548500	-0.32032600
C	2.81682800	-1.81940700	2.26137800

C	0.41766500	-2.30116200	2.64936700
C	0.71242900	2.75492800	0.12191400
C	-1.40015400	1.50491900	0.07894800
O	1.93915400	0.49796800	-1.74090600
O	3.17665500	0.20881000	0.09148000
O	-0.24840100	-3.19331400	-1.01829400
O	1.78938800	-2.31633600	-0.76767300
C	-2.16829400	0.32142000	-0.02353800
H	3.18348700	-1.88749700	1.22902500
H	3.42413400	-1.05348500	2.76736100
H	2.96467600	-2.78451000	2.76616100
H	-0.64008500	-2.02413400	2.64239400
H	0.67397900	-3.31203400	2.97857200
O	-0.06931500	3.85611700	0.02735500
O	1.92166900	2.86221600	0.21152600
C	3.13729700	0.59494700	-2.48690700
C	0.37902900	-4.40639400	-1.38522300
C	-3.61456300	0.41186300	-0.00921500
C	0.60662500	5.09741700	0.06550500
H	2.84564000	0.76840600	-3.52779000
H	3.75241200	1.42829300	-2.12020100
H	3.71560000	-0.33603400	-2.40426600
H	-0.42408400	-5.12208800	-1.58960700
H	1.01804200	-4.77843800	-0.57194700
H	1.00198100	-4.27406500	-2.28079700
O	-4.22678200	-0.78481900	-0.20346000
O	-4.27159700	1.42365400	0.16067300
H	-0.16067500	5.87159300	-0.03873400
H	1.33444500	5.17503800	-0.75393100
H	1.14291100	5.22599500	1.01631900
C	-5.63952800	-0.76077900	-0.20290200
H	-6.02512600	-0.11665100	-1.00591700
H	-6.02822700	-0.38557200	0.75434500
H	-5.96935100	-1.79309300	-0.36146900
H	-1.92893200	2.45931900	0.10055800
H	-2.03419200	-1.75969800	-0.61232400

Ph-4-2135-R4

C	-1.09334000	0.00004100	0.00000800
C	-0.38307200	1.19787600	-0.00001400
C	-0.38303400	-1.19783900	-0.00051600
C	1.00481700	1.21911900	0.00011100
C	1.00477700	-1.21933700	0.00005100
C	1.67135100	-0.00007400	0.00053900
H	1.54509400	-2.16466500	0.00013700
F	-1.06983500	2.33138300	0.00027400
F	3.00092100	-0.00001500	-0.00041700
F	-1.07008100	-2.33122800	0.00046900
H	1.54541400	2.16429600	0.00016400
F	-2.41872700	0.00004500	-0.00047900

Ph-4-2135-R4-Nu1

C	0.32178600	-0.07410000	-0.38302900
C	1.14458700	-1.20865100	0.03359100
C	1.14947600	1.11581300	-0.20831200
C	2.51532200	-1.21395300	0.20457500
C	2.51876100	1.16290600	-0.05631000
C	3.22508100	-0.02078500	0.13606500
F	0.49331500	2.30852300	-0.37233900
F	4.57830100	-0.00546500	0.28257200
F	0.49025600	-2.38407500	0.27475900
F	-0.06498000	-0.12793100	-1.82923700
C	-1.09461700	-0.04639900	0.31309600
C	-2.03262300	-1.18454100	-0.15899000
C	-1.86894600	1.23276200	-0.07292600
C	-0.89898200	-0.15875600	1.83264900
O	-1.80507700	-1.94569800	-1.05934700
C	-3.32575700	-1.31233800	0.63285800
O	-2.48128800	1.27842500	-1.11411300
C	-1.95298800	2.36990000	0.92096200
H	-0.53062600	-1.16324900	2.08204800
H	-0.14471200	0.56442700	2.16843800
H	-1.82867400	0.02242000	2.38849200
H	-3.12181200	-1.87009700	1.55935200
H	-3.74891400	-0.33857700	0.91503600
H	-4.04952700	-1.87906400	0.03614900
H	-2.74531600	2.13345300	1.64901600
H	-1.01777300	2.52026900	1.46875900
H	-2.23412900	3.28366100	0.38491800
H	3.01207100	-2.15668300	0.44038000
H	3.02171600	2.13101900	-0.07208200

Ph-4-2135-R4-Nu3

C	-0.71035700	0.19820800	-0.06903400
C	-0.91856300	-0.68223300	0.99057400
C	-1.76608200	0.28986400	-0.98163900
C	-2.07045900	-1.43922400	1.17986300
C	-2.93757600	-0.44757600	-0.85482400
C	-3.07235600	-1.29441100	0.23628900
F	-1.65731300	1.02789300	-2.07797800
F	-4.19788900	-2.00049300	0.36971400
F	0.05171600	-0.80474300	1.90332200
F	-1.54201300	2.64898500	0.10283900
C	0.60035300	0.98398500	-0.11756800
C	1.79109100	0.01083600	-0.18224900
C	0.67641200	1.79502900	1.12052300
C	0.72507500	1.97276600	-1.29849400
C	1.74641100	-1.03136300	-1.11657500
C	2.94555900	0.17160900	0.58732400
N	0.90111700	2.37448200	2.09363500
H	-0.13465400	2.64989400	-1.19624800
H	1.68810100	2.49487800	-1.20520400
H	0.71239800	1.42569000	-2.24962400

C	2.82814400	-1.89429700	-1.27669500
H	0.84956500	-1.16869400	-1.72535100
C	4.03050500	-0.69295000	0.42847700
H	2.99561800	0.97365000	1.32494700
C	3.97752700	-1.72859900	-0.50220300
H	2.77152200	-2.70150000	-2.00896500
H	4.92198300	-0.55407100	1.04250600
H	4.82501100	-2.40505300	-0.62316600
H	-2.17151700	-2.10347300	2.03592900
H	-3.73046900	-0.34732300	-1.59395100

Ph-4-2135-R4-Nu2

C	1.64112800	0.30417300	-0.29928500
C	2.27924600	-0.96941900	-0.58296900
C	2.41129500	0.95446100	0.74881400
C	3.53384500	-1.36287800	-0.16431700
C	3.66811700	0.60049200	1.19601400
C	4.24545000	-0.57523600	0.73306400
F	1.82390200	2.05646200	1.29574500
F	5.47784800	-0.95430900	1.15717200
F	1.64848800	-1.74981500	-1.49703600
F	1.62705600	1.10447600	-1.59149200
C	0.11719900	0.31730300	0.07831400
Br	-0.81142900	1.96629800	-0.40082300
S	-0.89490100	-1.09231500	-0.51854500
H	0.02714100	0.23980700	1.16804400
O	-1.02350600	-1.04190500	-1.96950100
O	-0.38998600	-2.28226500	0.16475800
C	-2.51320400	-0.77839800	0.17424500
C	-3.51576200	-0.26560400	-0.64526700
C	-2.74450900	-1.11327600	1.50677200
C	-4.78411700	-0.06649700	-0.10381600
H	-3.29153000	-0.03238200	-1.68671500
C	-4.01536000	-0.90120600	2.03885500
H	-1.94362600	-1.54588300	2.10843600
C	-5.02989700	-0.37860600	1.23490200
H	-5.58361300	0.33328000	-0.72865300
H	-4.21630700	-1.15423400	3.08043300
H	-6.02427200	-0.21856600	1.65423800
H	4.16169700	1.22523100	1.94159000
H	3.94062300	-2.30044800	-0.54535100

Ph-4-2135-R4-Nu4

C	0.13300800	0.15132100	0.29319000
C	1.56652300	0.16125300	0.74762600
C	-0.39878400	-0.97577200	-0.34350900
C	2.54708800	0.35945400	-0.38416900
H	1.68867700	0.98393100	1.47025100
H	1.64589200	-0.81367000	1.27736100
C	-0.76233000	1.18461300	0.53213400
C	-1.73456800	-1.08123000	-0.70884600

C	2.47882200	1.68854700	-1.08665300
C	3.42048800	-0.59097000	-0.72985400
C	-2.11590100	1.14251400	0.19881900
C	-2.57281400	-0.01121100	-0.41542000
H	2.65308900	2.50945100	-0.37225300
H	3.21472700	1.76630800	-1.89825700
H	1.47323400	1.84983800	-1.50817900
H	3.43834700	-1.54911000	-0.20531900
H	4.13315800	-0.44033600	-1.54552900
F	-0.30611700	2.29659600	1.12628800
F	-3.86400200	-0.09501100	-0.75535500
F	0.42005400	-1.95221200	-0.70430000
F	0.55820600	-2.08077000	1.99488600
H	-2.11055800	-1.97276500	-1.20801100
H	-2.77903000	1.97781700	0.41635300

Ph-4-2135-R5

C	-0.86360500	0.00003700	0.00003400
C	-0.14944500	1.19659300	-0.00516100
C	-0.14953400	-1.19655400	0.00514900
C	1.23781200	1.21590300	0.00614200
C	1.23774000	-1.21594500	-0.00613900
C	1.90137300	-0.00004800	0.00002500
N	3.38231300	-0.00008800	0.00004400
O	3.92696400	-1.07335500	0.00155600
O	3.92703000	1.07314400	-0.00148700
N	-0.84608700	2.50046400	-0.06711900
O	-0.23246600	3.45857000	0.31908700
O	-1.96247200	2.49997200	-0.51810100
N	-2.34803900	0.00003900	0.00005800
O	-2.87181600	0.32715400	1.02639200
O	-2.87178300	-0.32710700	-1.02632300
N	-0.84623400	-2.50038500	0.06705700
O	-1.96254100	-2.49990900	0.51821500
O	-0.23273300	-3.45847500	-0.31940700
H	1.77948000	2.16109900	0.01082200
H	1.77934400	-2.16117900	-0.01084900

Ph-4-2135-R5-Nu1

C	0.26965400	0.01412000	0.33898100
C	-0.60942800	-1.22424400	0.36895500
C	-0.66681800	1.20674300	0.41530600
C	-1.93676400	-1.24061200	0.05654200
C	-1.99905700	1.17228000	0.10303300
C	-2.63356200	-0.04305800	-0.16265600
N	-4.02142600	-0.07095200	-0.50520900
O	-4.60685700	0.98978900	-0.63106200
O	-4.55428200	-1.15445900	-0.66403200
N	-0.01195700	-2.48531300	0.73253900
O	-0.65918100	-3.50381100	0.62279800
O	1.13058100	-2.46162900	1.15998000

N	1.09249900	0.01897700	1.67728400
O	0.42602200	-0.00148300	2.67977600
O	2.28573000	0.05100100	1.63229900
N	-0.12406200	2.47447100	0.80703400
O	1.04634600	2.50286300	1.15545200
O	-0.82833600	3.46494000	0.77485800
C	1.21726900	0.03906500	-0.95524400
C	1.96176200	-1.33249800	-1.13072900
C	2.18998800	1.25312300	-1.01716300
C	0.27110000	0.13426900	-2.17321700
O	1.35774300	-2.20776700	-1.70805200
C	3.37682100	-1.57865800	-0.68040500
O	3.35333600	1.15847900	-0.72220000
C	1.68975400	2.54808500	-1.63302200
H	-0.38812600	-0.73826700	-2.20556400
H	-0.33639500	1.04399900	-2.15237900
H	0.86568000	0.13247500	-3.09718400
H	3.53456100	-2.66298800	-0.69957200
H	4.06107700	-1.09188300	-1.38933400
H	3.57335400	-1.15380700	0.30535700
H	1.81721100	2.46879400	-2.72392700
H	0.63494000	2.77707400	-1.44851700
H	2.32314600	3.36246200	-1.26491000
H	-2.45852900	-2.19490300	0.01645200
H	-2.56543200	2.10168700	0.09722500

Ph-4-2135-R5-Nu3

C	-1.06636200	0.19976600	-0.18585300
C	-0.37495400	-0.68416900	-1.21065800
C	-0.37286400	1.54828700	-0.23638900
C	0.93056700	-0.51805300	-1.57231700
C	0.93428100	1.69826800	-0.61143700
C	1.63167200	0.63312200	-1.18917900
N	3.03044600	0.74652900	-1.44897600
O	3.60042000	1.76718700	-1.10460100
O	3.59632400	-0.18789700	-1.98823700
N	-1.04401300	-1.86241500	-1.70231500
O	-0.39394600	-2.70451100	-2.29098200
O	-2.24296500	-1.95555400	-1.51729900
N	-2.54502100	0.40036700	-0.64350700
O	-2.68127800	0.90798300	-1.72362500
O	-3.42857100	0.04146400	0.08165500
N	-1.05854800	2.72090900	0.23152800
O	-2.23398700	2.61324800	0.53989500
O	-0.45188300	3.77263500	0.29615600
C	-1.00120800	-0.43675300	1.28306700
C	0.48717100	-0.73074100	1.57269400
C	-1.72781200	-1.72298400	1.33589700
C	-1.60520400	0.45215000	2.39007000
C	1.30856500	0.25255200	2.13520900
C	1.06756500	-1.94632100	1.19002600
N	-2.24890500	-2.74167500	1.47903400

H	-2.67630500	0.59888500	2.22850400
H	-1.43709200	-0.04133400	3.35653600
H	-1.12329500	1.43430500	2.42076700
C	2.68160300	0.04462900	2.26656000
H	0.89301700	1.21020800	2.44661000
C	2.43811300	-2.15739500	1.32320200
H	0.44815000	-2.73308200	0.75473600
C	3.25310000	-1.15605300	1.85055900
H	3.30557600	0.83315100	2.68929600
H	2.86955100	-3.10704000	1.00426700
H	4.32846800	-1.31438600	1.94292100
H	1.41772100	-1.28452700	-2.17228300
H	1.41819700	2.66354900	-0.47411900

Ph-4-2135-R5-Nu2

C	1.04405300	-0.61448100	0.40997700
C	1.63678800	0.53576700	1.20220200
C	1.94265700	-0.80632000	-0.80477400
C	2.32641200	1.55770200	0.62109400
C	2.62587900	0.23135700	-1.37586000
C	2.73147800	1.46350900	-0.71901600
N	3.43505500	2.54506500	-1.33494400
O	3.84504100	2.39416300	-2.47193500
O	3.58353300	3.57285900	-0.69879700
N	1.32968000	0.64559200	2.60461500
O	1.79317700	1.57027000	3.23542900
O	0.61466300	-0.21142900	3.08940400
N	1.08055400	-1.89061800	1.28752800
O	2.16340900	-2.18201000	1.72106200
O	0.05632800	-2.47854200	1.46673300
N	2.02636600	-2.08819100	-1.46341500
O	1.53902700	-3.05343300	-0.90802500
O	2.59924800	-2.15430300	-2.53271900
C	-0.40277400	-0.27716000	-0.07005300
Br	-1.22694100	-1.59749300	-1.21768300
S	-1.65140300	0.34562300	1.15597000
H	-0.27146000	0.60940000	-0.70659500
O	-2.15258000	-0.71964700	2.00581100
O	-1.06492300	1.55983400	1.72168700
C	-2.97960300	0.87009300	0.08798700
C	-4.10661800	0.06088600	-0.03574900
C	-2.87172200	2.10190600	-0.55510200
C	-5.15116600	0.49653300	-0.84803400
H	-4.15274400	-0.88858600	0.49884600
C	-3.92123300	2.52094400	-1.37056600
H	-1.98804100	2.72452100	-0.40584200
C	-5.05461700	1.71902100	-1.51602200
H	-6.04359600	-0.12005100	-0.95951000
H	-3.85753100	3.48008000	-1.88526100
H	-5.87488900	2.05326800	-2.15278800
H	3.12930300	0.07240100	-2.32763100
H	2.59013700	2.42768700	1.21999500

Ph-4-2135-R5-Nu4

C	0.88333500	0.11897900	-0.16458800
C	1.76321600	0.05974500	1.10701100
C	-0.08077400	1.28247200	-0.16034500
C	0.94312600	-0.26929000	2.33721600
H	2.54563700	-0.69102200	0.94982100
H	2.25772400	1.03206100	1.21573600
C	0.07894000	-1.13612600	-0.40835300
C	-1.44123200	1.18687500	-0.17633600
C	0.89161700	-1.71574700	2.75131100
C	0.30320500	0.68899100	3.01490000
C	-1.28389900	-1.21565500	-0.42203500
C	-2.06746000	-0.06215500	-0.29111400
H	1.90827800	-2.09846700	2.93262600
H	0.29889800	-1.84860600	3.66573100
H	0.44887100	-2.34834000	1.96571400
H	0.35752100	1.73794700	2.71017400
H	-0.29829900	0.45434200	3.89632800
N	-3.49014300	-0.15380700	-0.31350100
O	-3.99920500	-1.25617600	-0.42789700
O	-4.13913000	0.87380200	-0.21299400
N	0.47917800	2.60394000	-0.06888900
O	-0.24017800	3.54018900	0.22295400
O	1.66970000	2.72633700	-0.30557000
N	1.84794500	0.30800600	-1.40463000
O	1.30176900	0.36504900	-2.47814100
O	3.02898000	0.38297500	-1.21989600
N	0.80940700	-2.36150500	-0.57400300
O	2.01210700	-2.27679100	-0.75840800
O	0.21712100	-3.42410600	-0.53041100
H	-2.03671400	2.09506100	-0.10613300
H	-1.75638200	-2.18817900	-0.54593100

Ph-4-5123-R1

C	1.97019700	-0.00339500	0.01108800
C	1.27752300	-1.20510800	-0.01431000
C	1.28061800	1.20039100	-0.01377200
C	-0.11614400	-1.21009100	-0.00275800
C	-0.11300400	1.20947200	-0.00243500
C	-0.82952200	0.00051400	0.05383400
C	-0.77772400	-2.57980400	-0.10693200
C	-2.35388400	0.00237500	0.16518300
C	-0.77084100	2.58109600	-0.10692500
C	3.47908400	-0.00346100	0.06363200
F	3.91352000	0.02803000	1.32361600
F	3.98507300	-1.09554700	-0.50477200
F	3.98597800	1.05858200	-0.55855300
F	-1.19207400	3.03884200	1.06687200
F	0.09968100	3.47418000	-0.57901400
F	-1.80194200	2.56284500	-0.94883000

F	-2.78798500	-1.05961600	0.83687100
F	-2.93450900	0.00192200	-1.02833700
F	-2.78552100	1.06699200	0.83486900
F	0.08981400	-3.47492100	-0.58038300
F	-1.19852700	-3.03665300	1.06746600
F	-1.81005700	-2.55862700	-0.94740500
H	1.82262000	-2.14694000	-0.06111200
H	1.82852800	2.14074300	-0.06010800

Ph-4-5123-R1-Nu1

C	1.21485300	0.02701100	0.54678900
C	0.35558400	-1.20732500	0.66736900
C	0.28937800	1.21378900	0.68022300
C	-0.96167100	-1.22232400	0.31460700
C	-1.02672800	1.16927400	0.32879000
C	-1.70009900	-0.04344200	-0.01585600
C	-1.61459000	-2.58495100	0.31760200
C	-3.04718700	-0.07236600	-0.62242500
C	-1.74605600	2.49744700	0.35173800
C	2.21005500	0.06382800	1.75701700
F	1.78581800	0.90741800	2.71357200
F	2.35959700	-1.10643900	2.37538800
F	3.42986200	0.49247900	1.41681300
F	-3.01594100	2.39788300	0.75530300
F	-1.16151300	3.38133100	1.17320700
F	-1.75878400	3.08370000	-0.85695700
F	-4.09146300	-0.09604700	0.23999500
F	-3.23112500	-1.14725900	-1.41509700
F	-3.27485600	0.99976500	-1.40942500
F	-1.62184400	-3.14840800	-0.90033200
F	-0.97926000	-3.45423300	1.11520300
F	-2.88297600	-2.55076600	0.74205900
C	2.00593100	0.01790000	-0.86253700
C	2.91189000	-1.22140400	-0.98007400
C	2.91666300	1.23499900	-1.11276900
C	0.96897100	-0.01480300	-2.00340400
O	3.19423300	-1.89661600	-0.02143300
C	3.40980300	-1.59833700	-2.35810900
O	4.12020800	1.10971200	-1.14672800
C	2.28433500	2.56678900	-1.44769400
H	0.40363800	-0.95624300	-1.97636900
H	0.25522200	0.81123400	-1.89887100
H	1.45814400	0.07326200	-2.98344400
H	2.60532000	-2.12475500	-2.89386400
H	3.69597900	-0.71961100	-2.94844800
H	4.26284100	-2.27724000	-2.24753500
H	2.12843600	2.59213700	-2.53785100
H	1.30956000	2.71826300	-0.97294900
H	2.98629600	3.36688500	-1.18464600
H	0.69818400	2.14206000	1.07243900
H	0.83642400	-2.12209200	1.00164600

Ph-4-5123-R1-Nu3

C	1.72418400	-0.22169800	-0.41855100
C	1.05633800	1.05023500	-0.85207400
C	0.80059100	-1.36250900	-0.72983100
C	-0.30029900	1.18104900	-0.81367300
C	-0.55612300	-1.20219400	-0.70873400
C	-1.18227000	0.07359800	-0.60351400
C	-0.82606900	2.57724700	-1.05321400
C	-2.63068400	0.23740200	-0.33992700
C	-1.36139200	-2.47344200	-0.85543500
C	2.99808000	-0.41677700	-1.25483900
F	2.75775100	-0.23499300	-2.55433400
F	3.97577400	0.43514700	-0.92174800
F	3.51303600	-1.65019600	-1.13883300
F	-2.47484600	-2.30200400	-1.57640300
F	-0.66598400	-3.44126300	-1.47197100
F	-1.73065700	-2.99013400	0.32453200
F	-3.42546100	0.28286900	-1.43758500
F	-2.90774200	1.37188800	0.33061300
F	-3.13479700	-0.76312400	0.40715100
F	-1.06780400	3.23587300	0.08927800
F	0.04564800	3.33611700	-1.73275200
F	-1.96000500	2.58987600	-1.76250000
C	2.12074000	-0.16403200	1.16550100
C	0.82469100	-0.09209800	1.97769800
C	2.88216700	1.07164800	1.42421400
C	2.99217900	-1.36003100	1.59031100
C	0.17221700	-1.26909900	2.36130300
C	0.22694000	1.13602900	2.28437800
N	3.47374400	2.03674300	1.65084600
H	4.01103900	-1.26466100	1.19652500
H	3.03867300	-1.41942800	2.68570400
H	2.56315900	-2.29352200	1.20348200
C	-1.06684800	-1.21893100	2.99628200
H	0.61050800	-2.23974300	2.13073100
C	-1.00967000	1.18738500	2.92256900
H	0.71649300	2.06550500	1.99129600
C	-1.66604000	0.00875900	3.27187500
H	-1.57324700	-2.14855800	3.26037100
H	-1.46951000	2.15557000	3.12643300
H	-2.64371800	0.04626900	3.75527400
H	1.23103700	-2.33804800	-0.94845500
H	1.68274600	1.89366100	-1.14234600

Ph-4-5123-R1-Nu2

C	-0.00999800	1.15059900	0.65039400
C	0.99712200	1.65212900	-0.34841300
C	0.59737800	0.04364700	1.45876500
C	2.08351400	0.92267700	-0.73040900
C	1.69341200	-0.65059500	1.04275400
C	2.44099800	-0.32529500	-0.13430500
C	2.92757600	1.55501600	-1.81790100

C	3.53794300	-1.17524100	-0.65032900
C	2.10662700	-1.79143900	1.94973000
C	-0.34498500	2.32601500	1.58983400
F	0.76824700	2.88309400	2.06476800
F	-1.02339100	3.29061700	0.95625100
F	-1.06202400	1.94934900	2.65060700
F	3.43509500	-1.87373900	2.09049900
F	1.61187900	-1.66074800	3.18780600
F	1.67829300	-2.98086500	1.51058000
F	4.76884100	-0.91444600	-0.14784200
F	3.68218300	-1.07756100	-1.98610300
F	3.33589200	-2.48442500	-0.40762200
F	2.66387100	1.05453600	-3.03083600
F	2.71930600	2.87571000	-1.91751800
F	4.23836700	1.40445500	-1.60002100
C	-1.32923300	0.74409900	-0.13739900
Br	-0.95608700	-0.77660700	-1.27078400
S	-2.81671000	0.38627800	0.88791200
H	-1.63805000	1.56969600	-0.79282400
O	-2.45112400	-0.58625300	1.91376800
O	-3.37499300	1.68561300	1.25730700
C	-3.99543300	-0.38401600	-0.20562400
C	-4.14289200	-1.76877100	-0.17620900
C	-4.76902100	0.43034400	-1.03012600
C	-5.08894000	-2.35453500	-1.01440700
H	-3.52206100	-2.36438600	0.49408400
C	-5.70601000	-0.17011800	-1.86906200
H	-4.64503700	1.51392200	-1.00234400
C	-5.86268500	-1.55735400	-1.86060300
H	-5.22230600	-3.43682400	-1.00777400
H	-6.32118700	0.44742500	-2.52432100
H	-6.59958400	-2.02216800	-2.51713500
H	0.10390500	-0.21806400	2.39197600
H	0.82238300	2.63952500	-0.77464700

Ph-4-5123-R1-Nu4

C	-1.87244300	0.02794800	0.04563400
C	-2.20523800	-0.05114400	1.57761700
C	-1.11504500	-1.17830400	-0.44243500
C	-1.01552300	-0.22373000	2.49295600
H	-2.75350200	0.86680500	1.84726400
H	-2.88739400	-0.90451500	1.71459600
C	-1.08381400	1.25308300	-0.34548100
C	0.24589200	-1.18455700	-0.42575100
C	-0.35686000	1.02408600	3.01920800
C	-0.59245900	-1.44415200	2.84109300
C	0.27660100	1.22224400	-0.35482000
C	1.01904100	0.00448900	-0.22327800
H	-1.07880400	1.62336000	3.59764500
H	0.49315700	0.77943400	3.67065700
H	0.00447400	1.65781800	2.19765500
H	-1.08592400	-2.34330300	2.46471300

H	0.27843600	-1.57854200	3.48779100
C	0.90312500	-2.51916600	-0.67484000
C	2.45678900	-0.02112300	0.10439400
C	0.96591400	2.54719900	-0.57077000
C	-3.21642700	0.08133400	-0.67397300
F	-3.08379200	0.16668300	-1.99987300
F	-3.96508500	-1.00643100	-0.43480300
F	-3.94967900	1.13904400	-0.29317800
F	2.10534200	2.43486900	-1.26128000
F	0.19830200	3.41168500	-1.25080000
F	1.26974700	3.16673700	0.58255800
F	3.31914100	-0.01540900	-0.94316900
F	2.79857300	-1.11331500	0.81927900
F	2.82837400	1.03857500	0.85440100
F	1.17902000	-3.17744900	0.46068200
F	0.12091600	-3.34299600	-1.38959300
F	2.05285600	-2.41655900	-1.35275300
H	-1.62001200	2.17086300	-0.58859000
H	-1.67118200	-2.07009200	-0.73229300

Ph-4-5123-R2

C	-0.00015100	-1.86111900	0.00000000
C	0.00010100	-1.17116300	1.21613000
C	0.00010100	-1.17116300	-1.21613000
C	0.00023300	0.22452600	1.21490500
C	0.00023300	0.22452600	-1.21490500
C	0.00062000	0.93326500	0.00000000
C	0.00002300	0.94175900	2.46472700
N	0.00010100	1.51183300	3.46728200
C	0.00043000	2.37199000	0.00000000
N	-0.00087400	3.52543200	0.00000000
C	0.00002300	0.94175900	-2.46472700
N	0.00010100	1.51183300	-3.46728200
C	-0.00031200	-3.30359800	0.00000000
N	-0.00037900	-4.45701700	0.00000000
H	-0.00022300	-1.71463000	2.16037900
H	-0.00022300	-1.71463000	-2.16037900

Ph-4-5123-R2-Nu1

C	0.69447600	-0.36504400	0.93853100
C	-0.40052800	-1.41745600	0.93011800
C	0.05443300	0.99124500	1.14147500
C	-1.63724600	-1.11904600	0.43668100
C	-1.18532800	1.24409100	0.63863000
C	-2.04503300	0.21595900	0.11717300
C	-2.58934300	-2.19498400	0.28817200
N	-3.34026900	-3.06298400	0.15589800
C	-3.28931300	0.49931200	-0.48584200
N	-4.31122500	0.72869200	-0.99200200
C	-1.67810500	2.60006300	0.68783200
N	-2.07000500	3.68644000	0.71999200

C	1.58050200	-0.67611000	2.08508000
N	2.22743400	-0.98538600	2.98911500
C	1.60777300	-0.42735300	-0.37910600
C	2.76653900	0.58082000	-0.25975500
C	0.77259900	0.04162200	-1.58455000
C	2.17244800	-1.83918900	-0.57169700
O	2.90244000	1.28782900	0.70729400
C	3.77110800	0.59867700	-1.38896900
O	0.73994400	1.22310600	-1.84209200
C	0.07590000	-0.98043400	-2.44466200
H	2.84211700	-2.10258700	0.26040200
H	1.36227700	-2.57945900	-0.60596100
H	2.74175800	-1.91912800	-1.50764000
H	4.51839200	-0.18639600	-1.19550800
H	3.31198500	0.39906500	-2.36526400
H	4.28157900	1.56829800	-1.39766500
H	0.82869800	-1.47044200	-3.08289600
H	-0.40529700	-1.76259500	-1.84521700
H	-0.65604500	-0.47101300	-3.08122800
H	0.64266600	1.77822000	1.60746000
H	-0.17404800	-2.42181800	1.28621400

Ph-4-5123-R2-Nu3

C	-1.34570200	-1.00204100	0.02386300
C	-0.68477700	-0.94378600	1.37877300
C	-0.38378000	-1.58444200	-0.98225300
C	0.66884500	-0.81056300	1.48817900
C	0.96524900	-1.43943200	-0.82079200
C	1.56522200	-0.94516600	0.37915100
C	1.22224800	-0.56873500	2.80033700
N	1.65950500	-0.36103800	3.84915000
C	2.95678200	-0.73436800	0.49936200
N	4.10175500	-0.55674800	0.59700100
C	1.82429900	-1.85923000	-1.90382400
N	2.50497500	-2.18173900	-2.77964900
C	-2.54184100	-1.87160900	0.10755900
N	-3.47021800	-2.55389200	0.17156200
C	-1.88219300	0.46875000	-0.45006600
C	-0.69719400	1.42444600	-0.54167100
C	-2.81305100	0.92462500	0.59582100
C	-2.65991500	0.34479400	-1.76862100
C	0.02679900	1.53916600	-1.73452100
C	-0.26638400	2.14613200	0.57855300
N	-3.55339300	1.23815000	1.42409600
H	-3.58241300	-0.23130900	-1.62028000
H	-2.91961000	1.34010300	-2.15179400
H	-2.04932500	-0.17038700	-2.52107500
C	1.17090500	2.33327800	-1.79602500
H	-0.28652100	0.99324900	-2.62413100
C	0.87577800	2.94176200	0.51721500
H	-0.82363300	2.07760400	1.51462000
C	1.60354000	3.03113000	-0.66903800

H	1.72853900	2.40206000	-2.73115200
H	1.19940800	3.48982300	1.40318600
H	2.50234900	3.64760500	-0.71664400
H	-0.77986800	-2.08168300	-1.86697700
H	-1.31627300	-0.92846300	2.26711400

Ph-4-5123-R2-Nu2

C	0.51909200	-0.43053800	-0.18666700
C	1.09736900	0.73375100	-0.97237000
C	1.63910100	-1.44716700	-0.04621500
C	2.35574500	1.16332000	-0.65344700
C	2.89227700	-0.99858600	0.25823300
C	3.25577000	0.38346100	0.14181900
C	2.82982900	2.39331300	-1.24162300
N	3.20106200	3.38316700	-1.70760800
C	4.52568000	0.86568600	0.53681200
N	5.56574400	1.26481400	0.86675700
C	3.90954300	-1.96361600	0.59937100
N	4.72100300	-2.73710700	0.87848900
C	0.14068000	0.02641800	1.19222700
N	-0.03271100	0.38538900	2.27591600
C	-0.69240400	-1.09092600	-0.88104500
Br	-1.42889900	-2.52222200	0.19481100
S	-2.06735200	0.01598100	-1.43283800
H	-0.36315300	-1.52890800	-1.83423400
O	-1.51196100	0.82538500	-2.51620400
O	-3.21472500	-0.84029200	-1.69957700
C	-2.42509200	1.06037800	-0.05106400
C	-1.83779500	2.32353100	0.00986400
C	-3.26623700	0.57470500	0.95082700
C	-2.07722900	3.11062000	1.13365200
H	-1.20809000	2.67684900	-0.80727500
C	-3.48843600	1.37225300	2.06960400
H	-3.73048400	-0.40703700	0.84993100
C	-2.88918800	2.63042100	2.16185700
H	-1.62603000	4.10038600	1.20671800
H	-4.13368400	1.01232500	2.87138800
H	-3.06469500	3.24782000	3.04388900
H	1.40769700	-2.51037100	-0.09083400
H	0.50311500	1.23427700	-1.73221500

Ph-4-5123-R2-Nu4

C	-1.29019900	-0.53824600	0.67438600
C	-2.52167100	-0.46378700	-0.29372400
C	-0.68034300	0.83283300	0.89658000
C	-2.29891600	0.39108300	-1.51739800
H	-2.76391600	-1.49862000	-0.59392000
H	-3.38548200	-0.07336400	0.26444400
C	-0.23544200	-1.52130800	0.20021300
C	0.62051700	1.11779100	0.61817600
C	-1.16061900	0.03172500	-2.43324400

C	-3.10504700	1.42813500	-1.76861400
C	1.05838200	-1.17109400	-0.03465600
C	1.57413900	0.15818700	0.13045200
H	-1.16706800	-1.04350500	-2.66964000
H	-1.21558200	0.60372100	-3.36895500
H	-0.19190600	0.24523300	-1.95365500
H	-3.91909100	1.69369800	-1.08974700
H	-2.97374700	2.04159500	-2.66342200
C	1.06327100	2.47877300	0.82464300
N	1.41465500	3.56679100	0.98924100
C	2.91490800	0.49068000	-0.14093600
N	4.02385900	0.76448400	-0.36808300
C	1.95487100	-2.20338100	-0.50344600
N	2.66746000	-3.03135300	-0.87924300
C	-1.82663900	-1.04298600	1.97062100
N	-2.23286600	-1.43800700	2.97703500
H	-0.55522600	-2.55294000	0.04608500
H	-1.34301700	1.62033800	1.25783300

Ph-4-5123-R3

C	2.17285100	-0.31548000	0.00191300
C	1.59799700	0.95178800	-0.05418000
C	1.35544300	-1.43866000	0.10512700
C	3.65457700	-0.52129600	-0.05024200
C	0.21172400	1.10121300	0.01735900
C	-0.03118600	-1.30202200	0.13704100
O	4.32246700	0.62329900	-0.14372300
O	4.18010600	-1.60351200	-0.01209100
C	-0.61818200	-0.02704700	0.10837600
C	-0.29844500	2.51305400	-0.00643500
C	-0.82500500	-2.57488400	0.17878700
C	5.74199200	0.51963000	-0.20136900
C	-2.11821600	0.11537500	0.22999500
O	-1.52125000	2.62997200	0.49720400
O	0.35019300	3.44303000	-0.40831600
O	-2.04249400	-2.43156700	-0.33216800
O	-0.38859500	-3.61695200	0.59058500
H	6.12059300	1.54266200	-0.27317800
H	6.12409300	0.03038900	0.70367500
H	6.04463400	-0.06527700	-1.07935500
O	-2.66346300	0.46975500	-0.92431100
O	-2.72133500	-0.07667800	1.24816100
C	-2.08316100	3.93994600	0.50048100
C	-2.88309600	-3.58186700	-0.29766000
C	-4.07340200	0.66212300	-0.90273700
H	-1.43894600	4.62542700	1.06481800
H	-2.18819100	4.30859300	-0.52796600
H	-3.06169600	3.85065900	0.97974500
H	-3.03370300	-3.90876300	0.73898500
H	-2.42823300	-4.39907200	-0.87130600
H	-3.83275000	-3.27916500	-0.74684800
H	-4.33615600	1.44924600	-0.18342100

H	-4.57889400	-0.26873100	-0.61394500
H	-4.36028100	0.95600200	-1.91604000
H	2.22070400	1.84173800	-0.14259400
H	1.80045000	-2.43371300	0.14595600

Ph-4-5123-R3-Nu1

C	-1.32319100	-0.01722600	0.35065400
C	-0.42347100	-1.21640300	0.22077300
C	-0.51775200	1.17602300	0.79461100
C	-2.44333400	-0.22639200	1.37334900
C	0.92226600	-1.11258800	0.06747200
C	0.82838100	1.24000900	0.61595000
O	-2.42304300	-1.41480400	1.96798300
O	-3.25015500	0.62775300	1.65266800
C	1.62348100	0.13664200	0.16349300
C	1.65730300	-2.41051900	-0.06611300
C	1.47673700	2.56815100	0.86826700
C	-3.48713900	-1.67379300	2.87146900
C	3.01771500	0.30566000	-0.12082400
O	2.56989000	-2.58210900	0.89530000
O	1.42468500	-3.25283300	-0.89869300
O	1.76112900	3.19038900	-0.28150500
O	1.66804400	3.06565900	1.94804500
H	-3.31095100	-2.67428500	3.27749800
H	-3.50102900	-0.92951800	3.67800800
H	-4.44694400	-1.64165400	2.33780100
O	3.54170400	-0.71487800	-0.86392300
O	3.72789000	1.24714500	0.20848200
C	3.37978100	-3.73857600	0.77614200
C	2.52874300	4.37558300	-0.16173000
C	4.94290300	-0.70108900	-1.02884500
H	2.77013700	-4.65134800	0.81374000
H	3.92840800	-3.72044300	-0.17634800
H	4.08030900	-3.71616400	1.61665700
H	3.49523800	4.14416800	0.30719300
H	2.00427500	5.12527800	0.44579900
H	2.68185900	4.75200400	-1.17800700
H	5.45771900	-0.76466600	-0.05829700
H	5.27424600	0.21704300	-1.53334900
H	5.19514700	-1.57429600	-1.64106100
C	-2.06203800	0.26257700	-1.08813300
C	-3.05402200	1.43945100	-0.96602300
C	-2.74582000	-1.06250100	-1.42190800
C	-0.99710000	0.67652300	-2.10426500
O	-2.60952000	2.56396800	-0.91297100
C	-4.54528400	1.20551200	-1.00858900
O	-3.62552000	-1.48946800	-0.70243900
C	-2.23745000	-1.87687200	-2.58238200
H	-0.52862300	1.60747300	-1.76650700
H	-0.21996700	-0.09240800	-2.19904900
H	-1.44678400	0.85534000	-3.09216600
H	-5.04589000	2.17779400	-0.93909700

H	-4.81674800	0.71281100	-1.95471600
H	-4.85733100	0.54522500	-0.19285900
H	-2.21105900	-1.29114700	-3.51046400
H	-1.20232500	-2.18092600	-2.35608300
H	-2.86633800	-2.76586600	-2.70309400
H	-1.07012900	2.04142900	1.16231900
H	-0.88830900	-2.20344500	0.23829200

Ph-4-5123-R3-Nu3

C	-0.84129600	-0.75561700	0.29108400
C	0.38686700	-1.61749500	0.15688600
C	-0.46043400	0.61370700	0.78219900
C	-1.84910700	-1.45467900	1.20665800
C	1.63209600	-1.08762600	0.03153500
C	0.79168600	1.10947500	0.59360500
O	-2.48533600	-0.62255700	2.02808100
O	-2.08485000	-2.63667200	1.17036400
C	1.89126600	0.32214500	0.12396700
C	2.75449100	-2.07702300	-0.05502000
C	0.97730100	2.57340900	0.85768900
C	-3.51347100	-1.19419700	2.82450200
C	3.15450300	0.93469700	-0.16489500
O	3.64531700	-1.91497700	0.93037300
O	2.83615700	-2.96686300	-0.86396300
O	0.96672800	3.26809700	-0.28831500
O	1.04762200	3.09679800	1.93898600
H	-3.91213900	-0.38318200	3.44095100
H	-4.30278400	-1.60765300	2.18125100
H	-3.11116700	-1.99624500	3.45672500
O	3.99350500	0.12594200	-0.87544800
O	3.50585100	2.06858500	0.13606100
C	4.78939100	-2.74820300	0.86583100
C	1.29596800	4.64244100	-0.17315400
C	5.31885000	0.58730000	-1.02340100
H	4.50868800	-3.80783500	0.93543100
H	5.32397800	-2.58785400	-0.08143500
H	5.42589700	-2.46929900	1.71132900
H	2.30453700	4.74661800	0.24981900
H	0.57956600	5.16651500	0.47364600
H	1.26516900	5.05620400	-1.18598400
H	5.81767400	0.67770200	-0.04642800
H	5.34392900	1.56911900	-1.51468200
H	5.84156000	-0.15314500	-1.63918900
C	-1.61446200	-0.63838500	-1.15332800
C	-2.95486400	0.06295100	-0.96778800
C	-1.83070900	-1.99825200	-1.67889200
C	-0.69850700	0.07488700	-2.16058300
C	-3.02384400	1.46232800	-0.95987000
C	-4.12526500	-0.66628600	-0.71689500
N	-1.97548600	-3.04770900	-2.13795900
H	0.21175000	-0.51837800	-2.31625000
H	-1.21963800	0.19997400	-3.11980600

H	-0.39866900	1.05719600	-1.77713800
C	-4.23016100	2.11203800	-0.69738200
H	-2.12815600	2.05453500	-1.14703600
C	-5.33092900	-0.01647600	-0.45768800
H	-4.09046700	-1.75817500	-0.71914300
C	-5.38762300	1.37721900	-0.44414800
H	-4.26269500	3.20275000	-0.69325400
H	-6.23029200	-0.60475200	-0.26774300
H	-6.33043400	1.88788600	-0.24247900
H	0.24383700	-2.69914600	0.16795900
H	-1.24217700	1.24414000	1.20458000

Ph-4-5123-R3-Nu2

C	-0.21100700	-1.37544800	0.71577700
C	1.06607800	-1.83410500	0.04221800
C	0.15612500	-0.26833200	1.68947300
C	-0.89968800	-2.53739100	1.41646900
C	2.00349200	-0.91382500	-0.30002400
C	1.09856100	0.63899200	1.32246000
O	-0.11892800	-3.09146400	2.33890900
O	-2.01228600	-2.92646100	1.17188200
C	1.94670100	0.43927500	0.18300000
C	3.20304700	-1.42869200	-1.03358500
C	1.32365800	1.83328300	2.20482600
C	-0.67581300	-4.19042300	3.04962100
C	2.74656800	1.51476500	-0.33093100
O	4.35069900	-1.05581000	-0.45727200
O	3.16645100	-2.15410800	-1.99642400
O	0.54699500	2.86881600	1.85962200
O	2.06884700	1.86465300	3.14818900
H	0.08989500	-4.51758800	3.75867400
H	-1.58421900	-3.88017200	3.58212300
H	-0.93051800	-5.00416500	2.35800000
O	3.29920700	1.23792900	-1.54469400
O	2.92795200	2.60453200	0.19486200
C	5.53334200	-1.43126800	-1.14090600
C	0.79514300	4.07940200	2.55758000
C	4.21218100	2.19438300	-2.03935200
H	5.60787700	-2.52376600	-1.22610100
H	5.54321200	-0.99887500	-2.15163800
H	6.36963800	-1.03919800	-0.55387900
H	1.83283800	4.39869500	2.39031300
H	0.62857400	3.95172800	3.63526100
H	0.09950300	4.82076400	2.15228400
H	5.06276600	2.32144900	-1.35343400
H	3.72867100	3.17202700	-2.17202700
H	4.56543000	1.81849900	-3.00587900
C	-1.16071300	-0.79026500	-0.36826000
Br	-1.41621900	-1.92194100	-1.92113200
S	-2.81898900	-0.24227400	0.26117300
H	-0.67896400	0.11925600	-0.74953200
O	-3.89691700	-1.04966300	-0.29703000

O	-2.72222400	-0.08270400	1.71202000
C	-2.94247000	1.38569400	-0.46136400
C	-3.83208800	1.59177200	-1.51115400
C	-2.13140800	2.40210000	0.04282500
C	-3.91420400	2.86629400	-2.07332100
H	-4.44413500	0.76354700	-1.86972500
C	-2.21891500	3.66632700	-0.53509700
H	-1.43293600	2.20743000	0.86092500
C	-3.10967400	3.89717500	-1.58699800
H	-4.60701400	3.05247900	-2.89484000
H	-1.58677100	4.47358300	-0.16304900
H	-3.17378500	4.89039500	-2.03386900
H	-0.42460800	-0.15762900	2.60350400
H	1.19416100	-2.88815500	-0.20632600

Ph-4-5123-R3-Nu4

C	1.68654700	0.50887700	0.04834900
C	2.05163600	0.74418800	1.55176000
C	1.07176300	-0.85574400	-0.15451700
C	2.90165400	-0.33290500	2.17636200
H	2.56487700	1.71880500	1.61203300
H	1.09795500	0.82328300	2.09224700
C	0.69128100	1.57588000	-0.38565000
C	2.94588000	0.73817700	-0.78268400
C	-0.26965100	-1.03888600	-0.09254700
C	4.27294000	-0.55572200	1.59355700
C	2.44272900	-1.07456200	3.18987200
C	-0.64339100	1.35341800	-0.29541100
O	3.19235600	-0.21790400	-1.68096600
O	3.65303600	1.70965300	-0.66787800
C	-1.20306900	0.05990900	-0.01278600
C	-0.74848800	-2.43892000	-0.32011200
H	4.19829600	-0.99674400	0.58530700
H	4.81557300	0.39489500	1.48278000
H	4.86493700	-1.24146700	2.21378400
H	1.43574300	-0.92480700	3.58690600
H	3.05635800	-1.85527400	3.64716200
C	-1.56972600	2.51080400	-0.52248800
C	4.34661700	-0.03309200	-2.48853400
C	-2.59458700	-0.09026200	0.26637100
O	-1.64021800	-2.51508000	-1.31748000
O	-0.34531600	-3.41880700	0.25719000
O	-1.96473500	3.05785300	0.63188300
O	-1.89613800	2.94364100	-1.59815300
H	4.39268600	-0.89338700	-3.16270300
H	4.26950300	0.89952400	-3.06240100
H	5.24819400	0.01081300	-1.86187700
O	-2.90642900	-1.31423100	0.79769000
O	-3.48067600	0.74390200	0.10502300
C	-2.20928100	-3.79252400	-1.53678500
C	-3.02040300	3.99713300	0.52948800
C	-4.28099800	-1.60428800	0.90915200

H	-1.43950900	-4.52562700	-1.81423900
H	-2.71805600	-4.14559300	-0.62826800
H	-2.93075700	-3.67644100	-2.35183300
H	-3.90115600	3.50882000	0.08940700
H	-2.73083700	4.85223100	-0.09598800
H	-3.24125000	4.33162900	1.54817400
H	-4.77222900	-1.59219800	-0.07590700
H	-4.79180000	-0.87603600	1.55419100
H	-4.35628300	-2.60612500	1.34749300
H	1.72937900	-1.70989900	-0.32342600
H	1.08461500	2.56250300	-0.64170200

Ph-4-5123-R4

C	-1.67116700	0.00000900	0.00004800
C	-1.00476000	1.21934300	-0.00002400
C	-1.00472200	-1.21939600	-0.00001300
C	0.38314100	1.19790800	0.00003600
C	0.38312400	-1.19788300	0.00005100
C	1.09314600	0.00004300	0.00006700
F	2.41889700	-0.00002500	-0.00002700
F	1.06989200	-2.33134700	-0.00003600
H	-1.54576900	2.16422000	-0.00005800
H	-1.54569600	-2.16427900	0.00002900
F	1.06973900	2.33145000	-0.00001900
F	-3.00087300	-0.00009000	-0.00002500

Ph-4-5123-R4-Nu1

C	-0.01074900	-0.05641700	-0.39245800
C	0.78062300	-1.25820500	-0.04619300
C	0.78654000	1.17781000	-0.21351300
C	2.14419400	-1.20934300	0.08694300
C	2.15532000	1.14527300	-0.09422000
C	2.89347100	-0.02860300	0.05378000
F	2.83782400	2.30744600	-0.07679100
F	4.23946700	-0.02238300	0.21785300
F	2.82351000	-2.35371100	0.30356600
F	-0.42179200	-0.11623400	-1.80364000
C	-1.41822500	-0.02096000	0.32495800
C	-2.30132500	-1.23169400	-0.03730900
C	-2.19978700	1.20688600	-0.15916000
C	-1.22713200	-0.01425600	1.84763000
O	-1.94749800	-2.14400500	-0.73952100
C	-3.66483200	-1.28394800	0.63302100
O	-2.92389500	1.12794400	-1.12601600
C	-2.12167700	2.48925200	0.63818000
H	-0.79968200	-0.97498900	2.16874100
H	-0.51384300	0.77114400	2.13002400
H	-2.17193000	0.15120900	2.38518300
H	-3.55203800	-1.81871400	1.58893900
H	-4.08586200	-0.29343400	0.84330100
H	-4.34963300	-1.85941700	-0.00100500

H	-2.81970000	2.39866600	1.48603000
H	-1.12570200	2.67292200	1.05574000
H	-2.44642700	3.32545900	0.00795900
H	0.32938600	2.15614800	-0.35872900
H	0.29696300	-2.23133400	-0.00073300

Ph-4-5123-R4-Nu3

C	-0.40870100	1.08790200	-0.19415200
C	-0.76268700	0.58086500	1.13522800
C	-1.17378400	0.45009000	-1.26757300
C	-1.90227200	-0.15534400	1.33698400
C	-2.30655800	-0.28040600	-0.99230100
C	-2.72549700	-0.60180200	0.29823800
F	-3.04611400	-0.75037500	-2.01406400
F	-3.83752100	-1.33814700	0.53070900
F	-2.24022400	-0.52244200	2.58646700
F	-0.69740600	2.57703100	-0.24047600
C	1.15462000	1.15477000	-0.42993100
C	1.81485300	-0.21125600	-0.21988100
C	1.69169600	2.11664900	0.55295200
C	1.48382200	1.72219700	-1.82423000
C	1.57406800	-1.24160100	-1.13987500
C	2.65041100	-0.47915400	0.86919600
N	2.14244000	2.86166000	1.31103500
H	0.95661000	2.67368500	-1.96049300
H	2.56590700	1.87947100	-1.92813200
H	1.16457200	1.02222600	-2.60560300
C	2.15278900	-2.49757600	-0.97477400
H	0.91160800	-1.06561300	-1.98729900
C	3.23123900	-1.73790700	1.03665700
H	2.85524200	0.30247700	1.60199300
C	2.98599100	-2.75281700	0.11575400
H	1.94838000	-3.28373400	-1.70348500
H	3.87924000	-1.92018500	1.89555200
H	3.43900000	-3.73698400	0.24502600
H	-0.93664600	0.63957800	-2.31402100
H	-0.14953600	0.83518700	2.00173900

Ph-4-5123-R4-Nu2

C	-1.38222900	0.15749700	0.36647800
C	-1.99529700	-1.17990000	0.25636700
C	-2.22393400	1.19585800	-0.25699000
C	-3.33416000	-1.32286900	-0.01377300
C	-3.55506100	0.97523800	-0.51390900
C	-4.17091000	-0.27084100	-0.39128400
F	-4.31390100	1.99620400	-0.95196800
F	-5.48314900	-0.45692200	-0.66654200
F	-3.88070800	-2.55110700	0.03356400
F	-1.16345300	0.48439000	1.78331500
C	0.07576300	0.21403000	-0.17851400
Br	0.92113300	1.93088600	0.18845900

S	1.17376700	-1.11964700	0.46026400
H	0.08564000	0.08304000	-1.26616800
O	1.19038900	-1.09378600	1.91721000
O	0.78823400	-2.34372800	-0.24544500
C	2.80943200	-0.69890500	-0.11724500
C	3.72023100	-0.14488700	0.77867900
C	3.14491300	-0.97963600	-1.44019000
C	5.00424400	0.15115700	0.32611500
H	3.41542100	0.04517100	1.80845000
C	4.42984900	-0.66965500	-1.88240200
H	2.41411100	-1.44348700	-2.10459000
C	5.35415900	-0.10553700	-1.00117300
H	5.73398500	0.58400100	1.01127100
H	4.71262800	-0.87760300	-2.91489500
H	6.35987900	0.13161200	-1.35115500
H	-1.83373300	2.20332500	-0.39658600
H	-1.42793300	-2.07499100	0.50099300

Ph-4-5123-R4-Nu4

C	-0.60474000	-0.08972500	0.91141300
C	-2.13602600	-0.22127600	0.82424500
C	-0.08222500	1.17158600	0.36584100
C	-2.73934300	-0.05910800	-0.54755500
H	-2.40162400	-1.21119900	1.23378800
H	-2.55910400	0.53521300	1.49959200
C	0.15482200	-1.25555200	0.43509700
C	1.21010600	1.25232200	-0.08940000
C	-2.37637700	-1.07787800	-1.59276100
C	-3.57524100	0.95109300	-0.81501900
C	1.44226400	-1.10972700	-0.01955800
C	2.03213700	0.13398700	-0.26624600
H	-2.55095400	-2.09849000	-1.21606800
H	-2.95799500	-0.93351500	-2.51334800
H	-1.30367300	-1.01440600	-1.82790900
H	-3.83110900	1.69028200	-0.05143800
H	-4.02573300	1.07059600	-1.80394400
F	2.17620100	-2.20422200	-0.30947600
F	3.30570800	0.24501700	-0.72163900
F	1.71819000	2.44884100	-0.45046800
F	-0.46493400	-0.04851500	2.42568700
H	-0.25190000	-2.26258400	0.54537800
H	-0.68664200	2.07969100	0.40068200

Ph-4-5123-R5

C	-0.86360500	0.00003700	0.00003400
C	-0.14944500	1.19659300	-0.00516100
C	-0.14953400	-1.19655400	0.00514900
C	1.23781200	1.21590300	0.00614200
C	1.23774000	-1.21594500	-0.00613900
C	1.90137300	-0.00004800	0.00002500
N	3.38231300	-0.00008800	0.00004400

O	3.92696400	-1.07335500	0.00155600
O	3.92703000	1.07314400	-0.00148700
N	-0.84608700	2.50046400	-0.06711900
O	-0.23246600	3.45857000	0.31908700
O	-1.96247200	2.49997200	-0.51810100
N	-2.34803900	0.00003900	0.00005800
O	-2.87181600	0.32715400	1.02639200
O	-2.87178300	-0.32710700	-1.02632300
N	-0.84623400	-2.50038500	0.06705700
O	-1.96254100	-2.49990900	0.51821500
O	-0.23273300	-3.45847500	-0.31940700
H	1.77948000	2.16109900	0.01082200
H	1.77934400	-2.16117900	-0.01084900

Ph-4-5123-R5-Nu1

C	0.84573400	-0.08875000	0.55376300
C	-0.01627100	-1.32150600	0.57881700
C	0.03127000	1.16213200	0.71012100
C	-1.32773600	-1.23243900	0.29416000
C	-1.27406300	1.16704400	0.38873800
C	-2.02531900	-0.00343400	0.05080900
N	-1.96933500	2.46711100	0.53111900
O	-1.41533500	3.44357300	0.08296400
O	-3.01518700	2.47326400	1.12639700
N	-2.99610100	0.05890200	-0.94482400
O	-3.36135700	1.16323900	-1.34676400
O	-3.45090500	-0.98956800	-1.39848600
N	-2.09418900	-2.49790200	0.36739400
O	-3.12955600	-2.47822900	0.98235300
O	-1.60771300	-3.47526800	-0.14865200
N	1.77202600	-0.21451300	1.79542700
O	1.61201100	0.54826900	2.70673200
O	2.56469000	-1.12538100	1.78110100
C	1.76333400	0.00708900	-0.73383200
C	2.70552200	-1.22996900	-0.84867000
C	2.55058800	1.32610700	-0.59664800
C	0.85675000	-0.03486500	-1.97121500
O	2.20962000	-2.30409200	-1.07746400
C	4.19450700	-1.03059900	-0.77216000
O	3.20733800	1.51310500	0.40196300
C	2.43098100	2.37175900	-1.66902000
H	0.32540300	-0.99356000	-1.99536100
H	0.12463300	0.78380000	-1.96634800
H	1.45856200	0.03815300	-2.88697000
H	4.68938300	-1.99716400	-0.91378500
H	4.51440800	-0.32730100	-1.55723500
H	4.46337100	-0.58872400	0.19477400
H	2.64389800	1.95778100	-2.66333500
H	1.39259300	2.73976600	-1.68367100
H	3.11140900	3.19886700	-1.44097800
H	0.44683400	-2.27935800	0.81075000
H	0.52207300	2.06583900	1.06802500

Ph-4-5123-R5-Nu3

C	1.51096300	-0.17375400	-0.64799600
C	0.93867000	1.19527300	-0.85546900
C	0.56436400	-1.24471400	-1.09763400
C	-0.39715000	1.35795100	-0.86227800
C	-0.75931900	-0.99895200	-1.09992900
C	-1.34168200	0.28362000	-0.86005700
N	-1.62784500	-2.13079800	-1.49336900
O	-1.41655300	-3.19916100	-0.96987500
O	-2.45640700	-1.92326000	-2.34193100
N	-2.54348600	0.40106000	-0.16279100
O	-3.22326000	-0.60496100	0.02347500
O	-2.88121500	1.50940800	0.24737400
N	-0.88006800	2.75053400	-0.99310900
O	-1.69619700	2.97355300	-1.84978300
O	-0.38925400	3.57816100	-0.26282200
N	2.81799500	-0.28905100	-1.46303300
O	3.07395400	-1.34982400	-1.97369100
O	3.53383100	0.67973600	-1.48680400
C	1.96818300	-0.39833000	0.88261000
C	0.68858000	-0.42573300	1.72565300
C	2.79650600	0.74780000	1.30413700
C	2.81512400	-1.67408300	1.02695600
C	0.02495500	-1.63662100	1.95320200
C	0.10308300	0.76385900	2.17397700
N	3.45406200	1.62604100	1.65992500
H	3.80118100	-1.54463600	0.56245800
H	2.95656200	-1.90140400	2.09114300
H	2.31748400	-2.52617200	0.54737300
C	-1.21601000	-1.65210500	2.58605700
H	0.45115800	-2.57657600	1.60369200
C	-1.13875800	0.74664400	2.80741800
H	0.60075900	1.71905800	1.99684400
C	-1.80563400	-0.46080100	3.00647000
H	-1.73128600	-2.60194000	2.73400700
H	-1.59268000	1.68518900	3.12669500
H	-2.78767000	-0.47288800	3.48109900
H	1.61635400	2.04386800	-0.93608700
H	0.95308200	-2.21854800	-1.38682500

Ph-4-5123-R5-Nu2

C	0.43033300	-1.03418700	0.87634500
C	0.64837800	0.29780900	1.55416400
C	1.69939400	-1.47306200	0.18414900
C	1.46523000	1.19086300	0.96392300
C	2.49696800	-0.53172400	-0.35361500
C	2.32524600	0.87078200	-0.13034500
N	3.70593900	-1.02278700	-1.05450700
O	3.57157700	-1.96699000	-1.79558600
O	4.74937700	-0.48027700	-0.79869600

N	2.61100600	1.80342900	-1.12769700
O	3.19217400	1.42367900	-2.13983600
O	2.25985500	2.96805300	-0.96130900
N	1.57536500	2.50990100	1.62835000
O	2.67883200	2.95204900	1.80589100
O	0.54758100	3.03602800	1.99247100
N	0.11984900	-2.07705000	1.95992500
O	0.92584800	-2.16896100	2.84761900
O	-0.87801800	-2.73850000	1.84152800
C	-0.74109900	-0.95031800	-0.12596700
Br	-0.92566600	-2.46895500	-1.29686400
S	-2.37625100	-0.52931200	0.68056200
H	-0.51391900	-0.09942600	-0.78100300
O	-3.35464200	-1.56047200	0.37600100
O	-2.08660000	-0.17815300	2.06991800
C	-2.82655600	0.95659400	-0.18625800
C	-3.76473500	0.87248000	-1.21158200
C	-2.21095400	2.15542800	0.17520200
C	-4.09727000	2.03817500	-1.90115600
H	-4.22313500	-0.08738700	-1.45206400
C	-2.54979400	3.30780600	-0.53008800
H	-1.47508900	2.19063500	0.98279100
C	-3.48987000	3.24785200	-1.56195600
H	-4.83245000	1.99981700	-2.70556500
H	-2.07691200	4.25526200	-0.26973300
H	-3.75095400	4.15537500	-2.10803500
H	1.93176700	-2.53389000	0.09782900
H	0.11441000	0.52492700	2.47363200

Ph-4-5123-R5-Nu4

C	1.68016500	0.10847700	-0.12788200
C	2.02743900	-0.00063000	1.39672700
C	0.89444200	1.35498800	-0.42026800
C	0.80748900	-0.25750500	2.25593900
H	2.75638200	-0.81871600	1.51183800
H	2.51623000	0.94330800	1.67814100
C	0.98498400	-1.11907400	-0.63973400
C	-0.44325800	1.27561300	-0.48586900
C	0.50530400	-1.69189900	2.60082500
C	0.04679800	0.75587300	2.68385800
C	-0.35643400	-1.12097300	-0.69181100
C	-1.17489900	0.03902900	-0.46939100
H	1.32748900	-2.12484400	3.19311100
H	-0.42389100	-1.76811600	3.18039300
H	0.39614500	-2.30939800	1.69649000
H	0.27845900	1.79305000	2.42934500
H	-0.84997700	0.57283700	3.28062800
N	-0.99835300	-2.39155900	-1.09712700
O	-0.52490900	-3.41829300	-0.66467200
O	-1.92706400	-2.32894700	-1.86085500
N	-2.27162400	-0.07036900	0.37210900
O	-2.70492800	-1.19141600	0.64720200

O	-2.77041200	0.95725600	0.83479700
N	-1.17378300	2.54744800	-0.68443800
O	-2.10318300	2.54255100	-1.45082800
O	-0.76797600	3.52195300	-0.09393000
N	3.06128900	0.23403300	-0.79576300
O	3.56573500	-0.76190500	-1.24829400
O	3.58968300	1.31725400	-0.75517300
H	1.56943900	-2.00012400	-0.89775400
H	1.41928000	2.30410800	-0.51600000

Ph-5-12345-R1

C	1.21010700	1.16492700	-0.02414200
C	1.21987000	-0.23476400	0.03902500
C	-0.00003600	1.84219900	-0.10054100
C	-0.00004000	-0.93282800	-0.03299700
C	-1.21016500	1.16493900	-0.02416100
C	-1.21993800	-0.23476500	0.03883100
C	0.00002900	-2.43212900	-0.37704700
C	2.54084600	-0.97674800	0.28161400
C	-2.54093900	-0.97681200	0.28114900
C	-2.46571300	2.03225500	-0.08049800
C	2.46573000	2.03215600	-0.08094300
F	2.95554200	2.28847500	1.12561300
F	3.41815700	1.46684700	-0.81576600
F	2.18091100	3.20592700	-0.64373500
F	-2.95549500	2.28791400	1.12618400
F	-2.18073300	3.20632000	-0.64259000
F	-3.41818700	1.46741100	-0.81566100
F	-2.33140400	-2.11731300	0.93138500
F	-3.20171500	-1.24159900	-0.83470700
F	-3.32928400	-0.24262000	1.06234500
F	1.06334600	-2.72070900	-1.12510700
F	-0.00008200	-3.23597500	0.67254900
F	-1.06306900	-2.72076000	-1.12535500
F	3.32901300	-0.24246000	1.06291700
F	2.33126500	-2.11721000	0.93186300
F	3.20190400	-1.24156100	-0.83407200
H	-0.00002400	2.92522800	-0.20449900

Ph-5-12345-R1-Nu1

C	-1.06343900	-0.38306700	-0.43828700
C	-0.02630600	-1.42123400	-0.80384600
C	-0.34956500	0.99004500	-0.43354900
C	1.28395600	-1.28657000	-0.51225000
C	1.01042400	1.07656600	-0.15481700
C	1.84698300	-0.05701900	-0.03234600
C	2.14281000	-2.50084400	-0.78457500
C	3.09788700	-0.07003900	0.77539300
C	1.68799000	2.44217000	-0.03359900
C	-1.18606800	2.22863000	-0.62960200
C	-2.14747300	-0.45043400	-1.58855700

F	-1.96149100	0.53509400	-2.47873000
F	-2.08956400	-1.56435500	-2.32260400
F	-3.39845200	-0.35079900	-1.14762000
F	-2.49399200	1.95183000	-0.45360400
F	-1.09403000	2.81804000	-1.82940800
F	-0.91953900	3.20294200	0.26321800
F	3.00549100	2.36344700	-0.23574800
F	1.25048100	3.31195700	-0.94826300
F	1.51240900	3.00017400	1.16817500
F	4.25449300	0.13484500	0.11880500
F	3.25319500	-1.25953200	1.39308400
F	3.07321900	0.84476500	1.76590600
F	2.16764700	-3.35082300	0.25201000
F	1.68881800	-3.20927900	-1.82634300
F	3.40964400	-2.18231300	-1.06388500
C	-1.69150200	-0.76824700	0.99997300
C	-2.41120200	-2.13093500	0.97121700
C	-2.71971100	0.24098600	1.56129800
C	-0.54833700	-0.89799400	2.03160400
O	-2.56573300	-2.76041300	-0.04486200
C	-2.85015500	-2.71301200	2.29971100
O	-3.90377000	0.01287200	1.48480700
C	-2.21710500	1.43332100	2.34161400
H	0.08215000	-1.76805200	1.80589900
H	0.08985800	-0.00688100	2.02419000
H	-0.95258000	-1.01977700	3.04550700
H	-2.00664400	-3.28269000	2.71890800
H	-3.14843900	-1.95148400	3.02897300
H	-3.67763200	-3.40813400	2.11720900
H	-2.16270000	1.13382000	3.40012700
H	-1.22125400	1.76834000	2.03634600
H	-2.94411300	2.24837100	2.24951000
H	-0.38269800	-2.34107700	-1.25744900

Ph-5-12345-R1-Nu3

C	-1.41206700	-0.64019900	-0.26509800
C	-0.31663500	-1.29864900	-1.06533400
C	-1.12775300	0.86584500	-0.26257300
C	0.94291500	-0.81375400	-1.12317400
C	0.18866500	1.32649700	-0.33743400
C	1.28621200	0.48380300	-0.62301900
C	1.94556900	-1.69880600	-1.83417100
C	2.70957600	0.81139700	-0.30506100
C	0.49535600	2.81443400	-0.15378200
C	-2.27859500	1.81661700	-0.06971400
C	-2.74310400	-1.01272300	-1.02168200
F	-3.04615000	-0.08869900	-1.93475200
F	-2.63887800	-2.17033700	-1.68903500
F	-3.81274100	-1.17941700	-0.23942700
F	-3.40794100	1.17964200	0.27922100
F	-2.61185200	2.53875200	-1.15025800
F	-2.06451600	2.70102000	0.92819500

F	1.64028800	3.16563500	-0.74365200
F	-0.42336100	3.61204000	-0.70261700
F	0.59464700	3.15254300	1.13547100
F	3.41568700	1.40912600	-1.28521400
F	3.40275400	-0.30371300	-0.00987800
F	2.83354100	1.60843300	0.77095500
F	2.55711200	-2.55740500	-1.00964700
F	1.35850100	-2.45332100	-2.77372900
F	2.89713400	-0.99826500	-2.45743100
C	-1.44633200	-1.26926900	1.24108600
C	-0.04151000	-1.19413500	1.85869300
C	-1.82652100	-2.68882400	1.11373900
C	-2.45285200	-0.60542600	2.20528900
C	0.36416000	-0.03692900	2.53564200
C	0.86190000	-2.25682800	1.75373500
N	-2.14212400	-3.79410300	1.00775900
H	-3.48777900	-0.77434900	1.89901200
H	-2.29788300	-1.01847500	3.21094700
H	-2.28015800	0.47527500	2.25093100
C	1.64018400	0.05333100	3.08677500
H	-0.30225100	0.82178900	2.60826500
C	2.13958100	-2.16573500	2.30266400
H	0.57149100	-3.16562500	1.22414800
C	2.53340900	-1.01031700	2.97355200
H	1.94096800	0.97214900	3.59221600
H	2.83117900	-3.00262500	2.19470300
H	3.53570900	-0.93478600	3.39816000
H	-0.53737900	-2.24092000	-1.55732400

Ph-5-12345-R1-Nu2

C	-0.05229700	0.68138000	0.88568900
C	1.05201400	1.33017900	0.04033300
C	0.40773400	-0.66548700	1.37568900
C	2.16098300	0.60285700	-0.38055900
C	1.44048100	-1.34774800	0.83980900
C	2.33318500	-0.77576700	-0.12895500
C	3.35202400	1.34066800	-1.00880100
C	0.85999700	2.77293400	-0.33784900
C	3.24269300	-1.63875300	-0.93612700
C	1.65202500	-2.74018100	1.40110000
C	-0.30878600	1.52931500	2.16883600
F	0.85062300	1.89046600	2.71505700
F	-1.00735300	2.64133000	1.94631800
F	-0.96615200	0.83042200	3.09846000
F	2.94631500	-3.06421100	1.47788500
F	1.16039900	-2.85730400	2.64072500
F	1.04590000	-3.68470500	0.67520800
F	4.46773200	-1.87101300	-0.42705200
F	3.44947700	-1.14568800	-2.17144900
F	2.70565700	-2.85983300	-1.12236900
F	3.24838500	1.53433100	-2.32231700
F	3.54278500	2.53408900	-0.43377300

F	4.49491500	0.67944500	-0.80429400
F	1.32119000	3.65877000	0.56286500
F	1.41610100	3.09642200	-1.51124500
F	-0.44609100	3.08270700	-0.49027900
C	-1.40642600	0.54025900	0.06790200
Br	-1.08438300	-0.55007100	-1.49611700
S	-2.86290400	-0.11634600	0.98690900
H	-1.76323800	1.51053100	-0.28754100
O	-2.53109700	-1.40006100	1.59824400
O	-3.34433400	0.99385400	1.80547100
C	-4.09715400	-0.40544100	-0.26508800
C	-4.31846300	-1.70610500	-0.71162400
C	-4.83990800	0.67905500	-0.72769400
C	-5.30917300	-1.92035600	-1.66729700
H	-3.71994400	-2.52509700	-0.31102600
C	-5.82200900	0.45049600	-1.68961600
H	-4.65848800	1.67859800	-0.32954300
C	-6.05290500	-0.84457500	-2.15699400
H	-5.50105800	-2.93013900	-2.03147300
H	-6.41426400	1.28422800	-2.06799700
H	-6.82518600	-1.01838000	-2.90762300
H	-0.18753900	-1.11949400	2.16282700

Ph-5-12345-R1-Nu4

C	-1.16857600	-1.18227300	0.05275800
C	-1.52798600	-1.67034900	1.49970900
C	0.17898800	-1.75056700	-0.32016400
C	-0.72045900	-0.98017000	2.57612900
H	-2.60229100	-1.53563900	1.68520300
H	-1.31844300	-2.75035800	1.52362000
C	-1.14403100	0.33830100	-0.15271300
C	1.31924700	-1.03330700	-0.32005800
C	-1.27103000	0.30040600	3.14754200
C	0.44135100	-1.49291000	2.99649300
C	0.05630500	1.02624100	-0.25680900
C	1.32645900	0.40026900	-0.20349300
H	-2.31083900	0.16320000	3.48268800
H	-0.66750700	0.64291900	3.99875500
H	-1.28230800	1.10033000	2.39235300
H	0.84039700	-2.41686200	2.57145500
H	1.03775300	-0.98911400	3.76158600
C	2.58924700	-1.82477100	-0.54061700
C	2.56463800	1.12352000	0.18600700
C	0.04421600	2.51730900	-0.61033800
C	-2.46758500	1.04062100	-0.06735100
C	-2.15662700	-1.86673400	-0.91869400
F	-1.90950700	-1.53268200	-2.18632300
F	-2.05216000	-3.20457800	-0.84024800
F	-3.44307500	-1.59186300	-0.68850900
F	-3.30414400	0.41653600	0.78665500
F	-3.13775000	1.11960800	-1.23296500
F	-2.39505300	2.29394300	0.40800900

F	1.17114800	2.88240200	-1.23053900
F	-0.93236700	2.80397700	-1.48151900
F	-0.09467400	3.33088800	0.43754200
F	3.37003800	1.54184300	-0.81307800
F	3.35221300	0.34740000	0.95990400
F	2.31014000	2.22313800	0.92203800
F	3.14770900	-2.23320900	0.60657500
F	2.36717600	-2.93902700	-1.25220800
F	3.51727600	-1.13242400	-1.20751100
H	0.21556200	-2.82156300	-0.51645100

Ph-5-12345-R2

C	1.20769100	-1.11850400	0.00007500
C	1.21550500	0.28806300	0.00024800
C	-0.00012800	-1.81760300	-0.00005200
C	0.00002400	0.99103400	0.00012200
C	-1.20774400	-1.11840200	0.00006200
C	-1.21551700	0.28822500	-0.00005200
C	0.00015200	2.42993000	-0.00001600
N	0.00034700	3.58290600	-0.00013700
C	2.46130900	1.00742600	0.00013100
N	3.46151000	1.58124400	0.00011200
C	-2.46131100	1.00755500	-0.00019100
N	-3.46150400	1.58138500	-0.00007600
C	-2.45306200	-1.84255500	0.00003500
N	-3.44845700	-2.42456800	0.00013800
C	2.45289700	-1.84283800	-0.00008000
N	3.44829500	-2.42484400	-0.00027300
H	-0.00023600	-2.90684600	-0.00004700

Ph-5-12345-R2-Nu1

C	0.72141600	0.30124600	-0.78055000
C	-0.37893000	1.34034700	-0.54965000
C	0.06771300	-0.97205900	-1.28302700
C	-1.63335700	0.92560500	-0.11339100
C	-1.17857200	-1.31938400	-0.88191500
C	-2.03734000	-0.42059800	-0.14054000
C	-2.57032900	1.92076400	0.35124800
N	-3.31350800	2.71507800	0.73789600
C	-0.08950000	2.72228200	-0.69739900
N	0.16667100	3.84848700	-0.81292700
C	-3.29015500	-0.83835900	0.37669100
N	-4.31087800	-1.18514900	0.80546200
C	-1.69587700	-2.61038300	-1.26552800
N	-2.11110000	-3.64562900	-1.56452400
C	1.62880800	0.81536600	-1.83274100
N	2.30443300	1.26563600	-2.65153200
C	1.60809800	0.05472500	0.52677800
C	2.71573700	-0.97417800	0.22170000
C	0.72256800	-0.59076600	1.61248500
C	2.25527700	1.36093900	1.00341100

O	2.84892800	-1.46041600	-0.87330900
C	3.67529800	-1.29545200	1.34345700
O	0.60635200	-1.79397700	1.62156600
C	0.08579900	0.28099200	2.66252800
H	2.93543100	1.75821200	0.23568700
H	1.49868400	2.12659000	1.21559400
H	2.83298700	1.19881500	1.92336900
H	4.49344600	-0.55937400	1.31453700
H	3.20469500	-1.24584600	2.33310700
H	4.10098600	-2.29073400	1.17274400
H	0.86248100	0.56699300	3.38979200
H	-0.32249600	1.20901500	2.24399900
H	-0.69308500	-0.29097600	3.17905800
H	0.65539900	-1.63302400	-1.91592900

Ph-5-12345-R2-Nu3

C	-0.10764800	-0.65462700	-0.54926400
C	1.28202300	-1.25221700	-0.35773700
C	0.03129000	0.77874900	-1.01784000
C	2.36751500	-0.43693600	-0.05000700
C	1.12862800	1.52171100	-0.72518200
C	2.32186900	0.96487600	-0.13438800
C	3.61637300	-1.06420400	0.31309400
N	4.61512400	-1.56300000	0.60607100
C	1.44457800	-2.66054300	-0.45398700
N	1.55086500	-3.81270700	-0.53996700
C	3.42852800	1.77835400	0.21832800
N	4.32904200	2.45050700	0.50627300
C	1.12491400	2.91860300	-1.09214300
N	1.11685200	4.03711300	-1.37814700
C	-0.82968200	-1.41518400	-1.59503500
N	-1.38057100	-2.00529600	-2.41857100
C	-1.00998200	-0.74487600	0.78593000
C	-2.36237900	-0.07130700	0.54641600
C	-0.27880600	-0.01074100	1.83631200
C	-1.14075000	-2.20475700	1.25371300
C	-3.44429400	-0.81313600	0.05698600
C	-2.52887800	1.30324900	0.76063800
N	0.28333100	0.54040500	2.67925500
H	-0.17293600	-2.58977100	1.59715800
H	-1.85762900	-2.26490600	2.08271500
H	-1.49166200	-2.84532100	0.43352300
C	-4.66244300	-0.19211800	-0.21635800
H	-3.34451400	-1.88379800	-0.12085000
C	-3.74718400	1.92298700	0.48691200
H	-1.69998700	1.89618500	1.15196200
C	-4.81810300	1.17690400	-0.00341200
H	-5.49456000	-0.78659900	-0.59587400
H	-3.85763200	2.99380600	0.66281300
H	-5.77287200	1.66025800	-0.21509700
H	-0.80411400	1.22227600	-1.55861000

Ph-5-12345-R2-Nu2

C	0.79013300	-0.14884600	-0.18849100
C	1.69846100	1.06258200	-0.39140700
C	1.64462100	-1.33881100	-0.58644600
C	2.97247300	0.98982900	0.16360300
C	2.90796100	-1.40473100	-0.08346100
C	3.54003200	-0.25482500	0.51422000
C	3.77220200	2.18428400	0.26848300
N	4.41494100	3.13951700	0.35602900
C	1.29069700	2.19500800	-1.14345900
N	0.97622900	3.10140300	-1.79513300
C	4.80170000	-0.34939900	1.16158500
N	5.82835000	-0.43293200	1.69317100
C	3.67699600	-2.61100200	-0.26260100
N	4.29041300	-3.58005600	-0.39853000
C	0.44297100	-0.31453500	1.25715300
N	0.22561600	-0.50949600	2.37332400
C	-0.50674700	-0.04128700	-1.01415800
Br	-1.44001400	-1.72085900	-1.20734200
S	-1.68214900	1.28512700	-0.39940400
H	-0.27982100	0.30814600	-2.02942700
O	-0.95214700	2.08267400	0.58110800
O	-2.20411500	1.87982900	-1.62369600
C	-3.03812600	0.50656700	0.44803900
C	-2.96203000	0.33031200	1.82780900
C	-4.17692200	0.17684800	-0.28688000
C	-4.05919900	-0.22578800	2.48471700
H	-2.06438800	0.62500900	2.37165400
C	-5.26394100	-0.37441500	0.38480000
H	-4.20236700	0.35620100	-1.36225900
C	-5.20127800	-0.57876000	1.76551900
H	-4.02181900	-0.37519600	3.56408100
H	-6.16432300	-0.64108700	-0.16935100
H	-6.05647100	-1.01097300	2.28698000
H	1.22531200	-2.14130400	-1.18973000

Ph-5-12345-R2-Nu4

C	0.96185600	-0.84492200	-0.65871600
C	2.07089700	-1.34578200	0.32581800
C	0.72322400	0.65825600	-0.52042300
C	1.92741900	-0.82289200	1.73479100
H	2.04022200	-2.44924600	0.32095400
H	3.04994100	-1.04350700	-0.07346800
C	-0.34052800	-1.60478300	-0.48793500
C	-0.52492000	1.19308400	-0.24766800
C	0.66486700	-1.14860500	2.48672600
C	2.90671600	-0.09460300	2.28049800
C	-1.53291300	-1.01030800	-0.24944100
C	-1.69539900	0.41991800	-0.10269400
H	0.40268700	-2.21301000	2.38606500
H	0.77152300	-0.90743800	3.55245700
H	-0.18582600	-0.57105600	2.09050400

H	3.80478000	0.16479600	1.71478700
H	2.83205100	0.27138800	3.30744900
C	-0.63224600	2.62622500	-0.09485900
N	-0.72137000	3.77016500	0.02961600
C	1.86684400	1.49369200	-0.64307600
N	2.82813900	2.13279300	-0.76042000
C	-2.95521100	1.00293500	0.17104000
N	-3.99281500	1.47327800	0.39655600
C	-2.69529000	-1.85480300	-0.10193000
N	-3.62322100	-2.53170600	0.01682400
C	1.46173100	-1.14066200	-2.03110600
N	1.84150900	-1.37617500	-3.09543900
H	-0.28335700	-2.69090800	-0.57485300

Ph-5-12345-R3

C	-1.25603400	-1.38851800	-0.14987900
C	-0.03525900	-2.05405500	-0.17557800
C	-1.30153500	0.00588000	-0.00353500
C	-2.50163300	-2.22608500	-0.26328200
C	1.15303200	-1.34041600	-0.04760900
C	-2.61846300	0.69532800	-0.24354900
C	-0.10788400	0.72123800	0.16780100
O	-3.32524200	-2.01160100	0.75419500
O	-2.67996700	-3.03854700	-1.12916500
C	1.12039400	0.05081000	0.09954900
C	2.43214100	-2.12766900	-0.02461300
O	-2.66255200	1.92006900	0.25359100
O	-3.51188700	0.19328800	-0.87406900
C	-0.07883000	2.20807400	0.45618400
C	-4.58701900	-2.66766700	0.68033000
C	2.38585300	0.86576400	0.07297300
O	3.30322600	-1.63050800	0.84683200
O	2.61401100	-3.12199400	-0.67346200
C	-3.84354100	2.67433100	-0.01370900
O	0.18925700	2.90200500	-0.64260800
O	-0.22370600	2.67430000	1.54819600
H	-5.12456000	-2.40305800	1.59485300
H	-5.13457600	-2.31358000	-0.20347100
H	-4.45229000	-3.75442800	0.61301800
O	3.18686600	0.46791200	-0.90734200
O	2.61526200	1.79231900	0.80284100
C	4.56834800	-2.28411100	0.91190900
H	-3.69951300	3.64274200	0.47233900
H	-4.71925800	2.16006500	0.40064200
H	-3.97444500	2.79765800	-1.09616200
C	0.36577700	4.30429800	-0.45450200
C	4.45056800	1.12280100	-0.98638600
H	4.43775400	-3.33620300	1.19427800
H	5.06862000	-2.23276500	-0.06429500
H	5.14817500	-1.75154100	1.67039800
H	1.20266100	4.48226600	0.23349500
H	-0.54662000	4.74970600	-0.03763300

H	0.58126400	4.72183400	-1.44162800
H	5.01462700	0.96005100	-0.05821300
H	4.31110800	2.20021400	-1.13905200
H	4.97236400	0.67759300	-1.83751600
H	-0.00296400	-3.13852100	-0.28974500

Ph-5-12345-R3-Nu1

C	-1.08395300	-0.68807600	0.22511100
C	0.12123100	-1.54203200	0.55424200
C	-0.70352400	0.78089400	0.42064900
C	-2.22608500	-1.06447100	1.18325000
C	1.38356300	-1.09730000	0.37533900
C	-1.77479700	1.72215600	0.77084100
C	0.59940500	1.19257100	0.21564500
O	-1.80160800	-1.08360600	2.44629200
O	-3.35676200	-1.32761200	0.87032900
C	1.67614700	0.28333800	0.07008000
C	2.47436900	-2.07611500	0.67749100
O	-1.57688500	2.96665200	0.29926600
O	-2.75646600	1.45576000	1.43361900
C	0.95092900	2.66032100	0.20393900
C	-2.81760300	-1.21370900	3.42749100
C	2.97636400	0.71789400	-0.38336300
O	3.35890500	-1.60399800	1.55884300
O	2.53201800	-3.19593800	0.23003100
C	-2.53394500	3.93640000	0.68273900
O	0.96120500	3.14298100	-1.03850600
O	1.20763800	3.31353900	1.17949000
H	-2.31461100	-1.20208700	4.39930300
H	-3.51231800	-0.36698800	3.34295700
H	-3.37231600	-2.15180600	3.29437200
O	3.73458000	-0.31626300	-0.84386400
O	3.40862200	1.85762600	-0.41921100
C	4.47036100	-2.44204600	1.82885700
H	-2.20764000	4.88438400	0.24267800
H	-2.58691700	4.02471800	1.77642700
H	-3.52842800	3.66487400	0.30353500
C	1.41203900	4.47971900	-1.16279000
C	5.07335300	-0.00482700	-1.17054000
H	4.14387900	-3.39908000	2.25713800
H	5.02878300	-2.64235700	0.90346800
H	5.10013200	-1.90216700	2.54246500
H	2.44207500	4.56340000	-0.79011100
H	0.76778800	5.16205000	-0.59119200
H	1.37177800	4.72516400	-2.22867800
H	5.61702300	0.36793300	-0.28985100
H	5.12009000	0.76219700	-1.95544800
H	5.53160700	-0.93397100	-1.52652800
C	-1.54912700	-1.01608800	-1.28012900
C	-2.96505200	-0.46803900	-1.55881600
C	-1.45404300	-2.55003400	-1.44174000
C	-0.64363000	-0.28798600	-2.28529000

O	-3.15589700	0.72186900	-1.45070700
C	-4.03479500	-1.38490200	-2.09345700
O	-2.20745200	-3.28695100	-0.84740600
C	-0.35068100	-3.13331200	-2.29380600
H	-0.72916500	0.79292600	-2.12919100
H	0.40694800	-0.57800000	-2.15902600
H	-0.95348500	-0.51568500	-3.31649600
H	-4.91360900	-0.78640600	-2.35824300
H	-3.66393900	-1.92363800	-2.97905200
H	-4.27861200	-2.12963400	-1.32790700
H	-0.32651200	-2.69750800	-3.30049200
H	0.61446500	-2.91779500	-1.80748800
H	-0.49347700	-4.21829700	-2.35021800
H	-0.04987600	-2.56084700	0.90773600

Ph-5-12345-R3-Nu3

C	-0.52835900	-1.16319500	0.14731700
C	0.84838000	-1.79364600	0.12949300
C	-0.43067900	0.26344900	0.66867200
C	-1.37530400	-2.03559600	1.08395100
C	1.96856200	-1.05031000	0.02123700
C	-1.63708800	0.80010500	1.29170700
C	0.73004200	0.99521700	0.45771600
O	-0.89743400	-1.99825000	2.32779600
O	-2.28526300	-2.74987000	0.75322500
C	1.93201100	0.39658800	0.02629400
C	3.26740200	-1.79478700	0.05600500
O	-1.64175800	2.13880700	1.43784000
O	-2.58964100	0.13297600	1.65313200
C	0.76282200	2.48576000	0.69060000
C	-1.67443800	-2.67379000	3.30262600
C	3.02196400	1.19704700	-0.49504900
O	4.12753400	-1.26428600	0.93004200
O	3.50727600	-2.79654200	-0.57030400
C	-2.79884700	2.69753100	2.03181500
O	0.37089400	3.13645400	-0.40914300
O	1.11046300	3.02592200	1.70334900
H	-1.15393700	-2.54725700	4.25674600
H	-2.67592300	-2.22409000	3.35141900
H	-1.76993800	-3.73974400	3.05749600
O	3.91439400	0.45609300	-1.20149700
O	3.16409100	2.40333200	-0.39607100
C	5.41507400	-1.85623700	0.96384500
H	-2.62609100	3.77736700	2.08702200
H	-2.95977300	2.28808800	3.03814900
H	-3.68626800	2.48255600	1.41964500
C	0.49768500	4.54716700	-0.35287600
C	5.05593100	1.14270300	-1.67474300
H	5.35071900	-2.91708500	1.23974900
H	5.89536000	-1.77733800	-0.02187700
H	5.99170300	-1.30334400	1.71159000
H	1.54705200	4.81848700	-0.17424200

H	-0.12284800	4.96027600	0.45425600
H	0.16354300	4.92984400	-1.32224100
H	5.63187200	1.56909300	-0.84094700
H	4.77053600	1.95923000	-2.35184000
H	5.66203300	0.40482800	-2.21103400
C	-1.16848000	-1.18041100	-1.33089500
C	-2.52539100	-0.47095500	-1.38727800
C	-1.29965000	-2.58281300	-1.77583200
C	-0.19428800	-0.53161200	-2.33998200
C	-2.57181100	0.92676100	-1.49527100
C	-3.73275700	-1.17512900	-1.32473000
N	-1.32449300	-3.66033500	-2.18966300
H	0.73066600	-1.11558400	-2.41406900
H	-0.67197500	-0.48608500	-3.32804200
H	0.06588400	0.48423200	-2.02196900
C	-3.79310500	1.60001800	-1.50583300
H	-1.65132000	1.50882300	-1.53780700
C	-4.95347400	-0.50250900	-1.34949900
H	-3.71303300	-2.26127800	-1.23590000
C	-4.98990600	0.88913400	-1.43059600
H	-3.80420000	2.68935100	-1.57637600
H	-5.88254900	-1.07278200	-1.29872900
H	-5.94587000	1.41544600	-1.44332400
H	0.92356700	-2.88072600	0.21085300

Ph-5-12345-R3-Nu2

C	-0.30264500	-1.45313800	0.29190400
C	1.01604800	-1.94818800	-0.26307700
C	0.01638900	-0.47889200	1.42827500
C	-1.17987100	-2.62205800	0.72805500
C	2.02002900	-1.06024300	-0.43286200
C	-0.79887400	-0.51775600	2.64395800
C	1.02777700	0.43939600	1.19981100
O	-0.48826000	-3.53909100	1.39465300
O	-2.34530700	-2.72646900	0.45076100
C	1.94324300	0.26815200	0.13318500
C	3.29017600	-1.58152000	-1.02828400
O	-0.74455900	0.61974800	3.35895900
O	-1.46183900	-1.46228700	3.01777700
C	1.29621800	1.58064300	2.15191300
C	-1.26128900	-4.56367700	2.00158200
C	2.81587600	1.34695200	-0.28921000
O	4.36959600	-1.20498700	-0.33542000
O	3.35372400	-2.31440100	-1.98359200
C	-1.49194200	0.63636600	4.56158800
O	0.59374500	2.67169600	1.84058200
O	2.06632600	1.52451300	3.07123400
H	-0.55403500	-5.22640800	2.50906500
H	-1.95333300	-4.11208500	2.72487800
H	-1.83305300	-5.11903600	1.24678700
O	3.40431900	1.09552200	-1.48444700
O	3.01023700	2.40028400	0.29015400

C	5.61871100	-1.59583500	-0.87990800
H	-1.31781600	1.61547900	5.01947400
H	-1.16184600	-0.16268900	5.23901900
H	-2.56145900	0.50001500	4.35103300
C	0.87281900	3.81670900	2.62858300
C	4.35598500	2.04644700	-1.91939400
H	5.69229500	-2.68963200	-0.94330000
H	5.74303500	-1.17581100	-1.88813100
H	6.38710000	-1.20195000	-0.20779200
H	1.93131600	4.09239400	2.52753600
H	0.65235800	3.61952400	3.68641600
H	0.23139600	4.61908600	2.25069100
H	5.17661900	2.14005100	-1.19361000
H	3.89373700	3.03446500	-2.04946400
H	4.74170000	1.68395100	-2.87804800
C	-1.02067500	-0.66772600	-0.84356400
Br	-1.16901200	-1.65327800	-2.50736900
S	-2.67703700	0.07167300	-0.45157600
H	-0.38590800	0.19621100	-1.07805600
O	-3.74991200	-0.59695100	-1.17638500
O	-2.78319600	0.24698300	0.99468400
C	-2.47022300	1.69146400	-1.17460800
C	-3.07468500	1.97514400	-2.39535900
C	-1.68101400	2.61961800	-0.49565000
C	-2.88082600	3.23857900	-2.95522100
H	-3.68441600	1.21527900	-2.88571900
C	-1.48951000	3.87296600	-1.07264000
H	-1.21715000	2.36281300	0.46032600
C	-2.08969700	4.18062900	-2.29666800
H	-3.34742200	3.48590900	-3.90949800
H	-0.86830900	4.61137600	-0.56398200
H	-1.93685500	5.16475700	-2.74216600
H	1.12895300	-2.99638800	-0.53847400

Ph-5-12345-R3-Nu4

C	-1.01458900	-1.20757300	0.29727300
C	-1.18674000	-1.24382800	1.85035700
C	-0.88859100	0.22096900	-0.20557300
C	-2.25049700	-0.34240500	2.43431400
H	-1.35841700	-2.29057900	2.14580600
H	-0.20963900	-0.93314800	2.24842500
C	0.25677200	-1.95764300	-0.05745100
C	-2.14387000	-1.96850600	-0.38803500
C	-2.13264400	0.88873200	-0.53435600
C	0.34285100	0.85449000	-0.10347400
C	-3.65599700	-0.86798000	2.54005000
C	-1.93326600	0.89748200	2.82686900
C	1.44302500	-1.31837700	-0.06097900
O	-2.08670300	-1.85015400	-1.71800500
O	-2.93522200	-2.69114000	0.16367100
O	-1.99089800	2.14930100	-1.00017000
O	-3.24097900	0.38617800	-0.43502200

C	1.53293400	0.12048200	0.10561000
C	0.41679000	2.36094600	-0.15788700
H	-4.07110300	-1.01934300	1.53456300
H	-3.67428900	-1.84485500	3.04819900
H	-4.29760800	-0.16726000	3.09238900
H	-0.91829600	1.28749300	2.71266100
H	-2.68993300	1.56987500	3.24125900
C	2.65221800	-2.08383700	-0.50092400
C	-3.14795000	-2.45526700	-2.43547900
C	-3.18821200	2.86349400	-1.23632500
C	2.81126800	0.66585900	0.51456600
O	0.82004400	2.79616100	-1.35038200
O	0.15929400	3.08822400	0.76494100
O	2.76355400	-3.26464300	0.12307100
O	3.41160800	-1.73625000	-1.36823000
H	-2.94759200	-2.27803200	-3.49667800
H	-3.19690700	-3.53316100	-2.23142000
H	-4.10316900	-1.99387400	-2.14759500
H	-2.89283800	3.86302900	-1.57225800
H	-3.78871700	2.93525100	-0.31881000
H	-3.79259900	2.36944700	-2.00930500
O	2.89314900	2.01681300	0.44838000
O	3.75865600	0.00949200	0.91198800
C	0.95699000	4.20043800	-1.46922100
C	3.84015800	-4.08542900	-0.30228200
C	4.09078900	2.59194900	0.93085100
H	-0.00175300	4.70038400	-1.27264900
H	1.70252200	4.57388400	-0.75323500
H	1.28456800	4.39710600	-2.49478000
H	4.79817000	-3.56969800	-0.15358600
H	3.73955000	-4.33815800	-1.36658900
H	3.79835100	-4.99252300	0.30847800
H	4.95349300	2.26208100	0.33571400
H	4.26716100	2.31369300	1.97924600
H	3.96982000	3.67728500	0.84653600
H	0.20056800	-3.02472700	-0.28571700

Ph-5-12345-R4

C	-1.19368800	0.96787600	0.00008600
C	-1.20797700	-0.42458200	-0.00005000
C	0.00006800	1.67610900	0.00001000
C	0.00007300	-1.11696900	0.00011700
C	1.19353600	0.96796300	0.00008400
C	1.20782600	-0.42465400	-0.00000900
F	2.35437300	1.60695700	-0.00010300
F	2.35107900	-1.09041800	-0.00002000
F	-0.00031700	-2.43782800	0.00007200
F	-2.35086000	-1.09063400	-0.00016100
H	-0.00028300	2.76520500	0.00002900
F	-2.35413500	1.60751600	0.00005200

Ph-5-12345-R4-Nu1

C	-0.02366100	-0.19305900	-0.42360200
C	0.78656800	-1.38890100	-0.11962400
C	0.79520800	1.00995400	-0.22382700
C	2.14915500	-1.34314200	0.07118900
C	2.15569400	1.01561500	-0.04502800
C	2.88886300	-0.16487500	0.12905400
F	0.18366300	2.21124900	-0.43965900
F	2.80253900	2.18898600	-0.00936200
F	4.22885000	-0.14359100	0.32182700
F	2.81679300	-2.49767400	0.26410600
F	-0.46489500	-0.18154900	-1.82349800
C	-1.41145900	-0.16386300	0.33460400
C	-2.31344500	-1.34364800	-0.08037800
C	-2.20596500	1.10030300	-0.03858200
C	-1.17498900	-0.27212100	1.84697700
O	-1.96737000	-2.23135400	-0.81676700
C	-3.67728300	-1.40524400	0.58723100
O	-2.87387300	1.11464100	-1.04696800
C	-2.22999100	2.26738000	0.92171400
H	-0.76993900	-1.26465700	2.08868600
H	-0.43131100	0.47058500	2.16656000
H	-2.09886400	-0.11646500	2.42182400
H	-3.57522600	-2.00031400	1.50797400
H	-4.07792400	-0.42109300	0.85851400
H	-4.37549900	-1.92648200	-0.07843800
H	-2.98315400	2.05613500	1.69770300
H	-1.26587800	2.42635000	1.41474100
H	-2.53430500	3.16768800	0.37549700
H	0.30732900	-2.36522300	-0.12227500

Ph-5-12345-R4-Nu3

C	-0.36060800	1.07281800	-0.15394400
C	-0.76436200	0.73109700	1.20996800
C	-1.09194800	0.25561200	-1.11204000
C	-1.89703300	0.00066100	1.49341300
C	-2.21503500	-0.47338900	-0.79983300
C	-2.66390600	-0.62573100	0.51591700
F	-0.72209500	0.28324800	-2.42408500
F	-2.88233100	-1.08779800	-1.78436700
F	-3.78715700	-1.32076300	0.79889200
F	-2.26045500	-0.17463100	2.77607100
F	-0.67225300	2.53425000	-0.43912900
C	1.21277000	1.13606000	-0.33517800
C	1.84695400	-0.23321000	-0.08373900
C	1.69469000	2.10579500	0.66852700
C	1.65155100	1.70585000	-1.70333800
C	1.62757200	-1.26306500	-1.01052300
C	2.63319500	-0.50352700	1.03954500
N	2.09835000	2.86984200	1.43387900
H	1.13364300	2.65471800	-1.87927000
H	2.73739400	1.87359600	-1.70017300

H	1.40380800	1.01028400	-2.50975200
C	2.18342800	-2.52492500	-0.81581400
H	1.01043200	-1.07768600	-1.89037100
C	3.19158700	-1.76874100	1.23462500
H	2.81612100	0.27995100	1.77645200
C	2.96953600	-2.78422500	0.30855600
H	1.99868600	-3.31248700	-1.54820200
H	3.80253100	-1.95595100	2.11919200
H	3.40450500	-3.77334000	0.46004300
H	-0.18728600	1.11680100	2.05164700

Ph-5-12345-R4-Nu2

C	1.33131400	0.27481700	-0.45181900
C	2.03733400	-1.00322000	-0.30739200
C	2.12935300	1.35592400	0.15047300
C	3.34288800	-1.11981000	0.10209000
C	3.43308700	1.19171800	0.56574400
C	4.08506500	-0.03506700	0.58560300
F	4.10425200	2.26171500	1.02561400
F	5.36411000	-0.18385100	0.99503900
F	3.92429200	-2.32370300	0.05728000
F	1.45005700	-2.10026700	-0.84280900
F	1.09193500	0.50259300	-1.87837600
C	-0.13091900	0.30789200	0.11230400
Br	-1.05082100	1.93651100	-0.43449000
S	-1.19444100	-1.14640100	-0.26852300
H	-0.09807500	0.32204200	1.20726400
O	-1.23889200	-1.37135300	-1.70782100
O	-0.76964700	-2.19796900	0.65438800
C	-2.83393700	-0.66449000	0.25439400
C	-3.76586600	-0.27496900	-0.70445200
C	-3.14939500	-0.73398300	1.60969400
C	-5.04930700	0.07012600	-0.28615900
H	-3.47750300	-0.24640200	-1.75584000
C	-4.43409400	-0.37702000	2.01607700
H	-2.40244100	-1.07440600	2.32837300
C	-5.37897700	0.02433300	1.07009800
H	-5.79482100	0.37638700	-1.02089300
H	-4.70042800	-0.42161200	3.07269800
H	-6.38441600	0.29906100	1.39235900
H	1.70470200	2.35816100	0.19636100

Ph-5-12345-R4-Nu4

C	-0.55977700	-0.13351000	0.91938600
C	-2.09122600	-0.23465300	0.80839500
C	-0.00129600	1.04362300	0.25846000
C	-2.63116800	-0.34195000	-0.59795600
H	-2.39630200	-1.11022700	1.40343900
H	-2.50244800	0.65788300	1.29749700
C	0.20206900	-1.32981600	0.55640300
C	1.27611500	1.09748600	-0.23425000

C	-2.56690300	-1.69171500	-1.25857000
C	-3.14310800	0.72489300	-1.22079700
C	1.48965100	-1.24880500	0.06590400
C	2.07591900	-0.05350500	-0.33553400
H	-3.08451000	-2.44707500	-0.64552400
H	-3.02381900	-1.67459100	-2.25720800
H	-1.52050200	-2.01903500	-1.34980600
H	-3.17041700	1.70121400	-0.73172400
H	-3.53202900	0.65491600	-2.24013100
F	2.20495500	-2.37984700	-0.10313600
F	3.35137700	0.02157700	-0.78473500
F	1.76759400	2.26171500	-0.68666100
F	-0.74578000	2.18140500	0.25650500
F	-0.45198800	0.08877400	2.42717800
H	-0.20704600	-2.31800100	0.77245000

Ph-5-12345-R5

C	1.19161500	-1.16117200	0.00484900
C	1.20706700	0.22878500	-0.01075800
C	0.00015400	-1.86831000	-0.00007600
C	-0.00006400	0.91818100	-0.00009300
C	-1.19138200	-1.16135500	-0.00459000
C	-1.20706400	0.22867800	0.01067400
N	-2.45035400	-1.93677400	-0.08312900
O	-2.41738500	-3.05644700	0.34821500
O	-3.38836700	-1.38577400	-0.59648600
N	-2.48107500	0.99127500	0.03515800
O	-3.14746600	0.86237100	1.02077800
O	-2.70198100	1.66686000	-0.92995600
N	-0.00009000	2.39961400	-0.00069000
O	0.78896700	2.92940400	-0.73397200
O	-0.78915800	2.92997800	0.73218300
N	2.48087000	0.99171900	-0.03458000
O	2.70010700	1.66861800	0.93005500
O	3.14865000	0.86200400	-1.01911800
N	2.45067800	-1.93640300	0.08317100
O	2.41827100	-3.05535600	-0.35008200
O	3.38806900	-1.38618500	0.59846900
H	0.00019200	-2.95864600	-0.00024200

Ph-5-12345-R5-Nu1

C	-0.78872200	-0.29936400	0.51650900
C	0.00444200	0.98977900	0.58605200
C	0.17106700	-1.45460300	0.71907400
C	1.31813000	1.03456300	0.14240100
C	1.46414800	-1.34382900	0.40159100
C	2.06788100	-0.11801500	-0.05817800
N	2.33596800	-2.48523400	0.74548000
O	1.88893600	-3.59523400	0.59300700
O	3.42160700	-2.22357800	1.19826300
N	3.14404900	-0.18530400	-0.98923300

O	3.45863400	-1.29042700	-1.39524500
O	3.68402700	0.84413800	-1.34668800
N	1.91785100	2.34330600	-0.23640500
O	2.74391400	2.79788900	0.50151300
O	1.52393900	2.81838000	-1.26813400
N	-0.65998500	2.17377100	1.00326900
O	-0.03191700	3.20982100	1.09743600
O	-1.84879800	2.08475300	1.27806100
N	-1.69790300	-0.37785500	1.77980000
O	-1.12177600	-0.30660000	2.83376600
O	-2.87878200	-0.52696400	1.63929200
C	-1.64013500	-0.44559500	-0.79682200
C	-2.56671300	0.78947300	-1.03422000
C	-2.41080900	-1.77299900	-0.84306400
C	-0.66411400	-0.48725400	-1.99537400
O	-2.10656200	1.73888900	-1.61798400
C	-4.00370300	0.79247300	-0.57276300
O	-2.25284300	-2.64150200	-0.02111000
C	-3.29349400	-2.00411900	-2.05130900
H	-0.08743800	0.44467300	-2.03565200
H	0.01382600	-1.34843500	-1.92960600
H	-1.23081600	-0.55742300	-2.93234900
H	-4.03540800	1.35418600	0.37098400
H	-4.60208800	1.32812200	-1.32052200
H	-4.41075800	-0.20548500	-0.38193300
H	-3.57580000	-1.08266500	-2.57547600
H	-2.73933400	-2.64577500	-2.75296700
H	-4.18993800	-2.55128100	-1.73446000
H	-0.21300100	-2.37627200	1.15079200

Ph-5-12345-R5-Nu3

C	-1.43943000	-0.31188300	-0.34346400
C	-0.54069900	-1.17110000	-1.20297000
C	-0.91485200	1.10344000	-0.36413600
C	0.75044500	-0.86035100	-1.35847100
C	0.44482300	1.34269800	-0.45983400
C	1.34120400	0.34948200	-0.84467200
N	0.97892900	2.68471500	-0.09149900
O	0.88858700	3.00362400	1.06370200
O	1.45739200	3.32946600	-0.98118200
N	2.70112200	0.34217000	-0.42242300
O	3.15820400	1.34112200	0.10112300
O	3.33945900	-0.68039600	-0.59503900
N	1.53990600	-1.73675700	-2.24707400
O	2.23516700	-1.19587600	-3.06756600
O	1.39462100	-2.92758700	-2.12017100
N	-2.78805800	-0.34290500	-1.12061800
O	-2.78332500	0.20322300	-2.19103700
O	-3.70651800	-0.95703200	-0.65126800
N	-1.83919600	2.18379800	-0.25391200
O	-2.99487700	1.91644000	0.03637300
O	-1.44785500	3.31404700	-0.46819800

C	-1.59472700	-0.93897700	1.11156900
C	-0.18672000	-0.94479700	1.74331600
C	-2.03876600	-2.34141400	0.96578800
C	-2.60413000	-0.20632000	2.01469500
C	0.28208900	0.19099100	2.41265700
C	0.68041000	-2.03122600	1.57406200
N	-2.35641300	-3.44668000	0.87956200
H	-3.61555100	-0.27279800	1.60410000
H	-2.57540600	-0.66757000	3.01065200
H	-2.34313100	0.85193700	2.10865200
C	1.60023800	0.25619800	2.86186500
H	-0.35579500	1.06312600	2.54724400
C	1.99417700	-1.97074700	2.03504400
H	0.34087400	-2.92657400	1.05062600
C	2.46226600	-0.82213700	2.67166800
H	1.95373200	1.16597700	3.34838400
H	2.65840500	-2.82103600	1.87626900
H	3.49746100	-0.76476400	3.01071100
H	-0.95943100	-2.05806600	-1.68147900

Ph-5-12345-R5-Nu2

C	0.50918100	0.41801100	-1.14621000
C	1.28196400	-0.86625700	-0.96496700
C	1.38519400	1.56324700	-0.68342600
C	2.11126800	-1.01432300	0.13009100
C	2.25466500	1.37576700	0.31699300
C	2.53762200	0.08307200	0.87973000
N	3.11969900	2.52424600	0.66270800
O	2.60510700	3.61443000	0.69397400
O	4.28799900	2.29241600	0.83784000
N	2.99348100	-0.02238300	2.22376600
O	3.01779700	0.99861200	2.88711500
O	3.31653200	-1.11449600	2.65058800
N	2.54336200	-2.37565700	0.54989400
O	3.67017900	-2.68402600	0.28280700
O	1.72611700	-3.03493900	1.13053900
N	0.97347700	-1.96479600	-1.82129400
O	1.61463300	-2.98900900	-1.71832800
O	0.07859500	-1.80148200	-2.63100700
N	0.28884100	0.68794100	-2.65266400
O	1.29185600	0.71908900	-3.31733000
O	-0.82548600	0.88976900	-3.03694800
C	-0.81839100	0.44598900	-0.34454300
Br	-1.73033800	2.14945500	-0.41259800
S	-2.01007900	-0.95722200	-0.54889000
H	-0.51061200	0.32288700	0.70388800
O	-2.73243500	-0.86563700	-1.80454700
O	-1.24399900	-2.14811600	-0.18998200
C	-3.15651300	-0.64912500	0.77760100
C	-4.38187100	-0.05282100	0.48721700
C	-2.80768500	-1.05392300	2.06527700
C	-5.27924500	0.16001800	1.53119600

H	-4.61789700	0.23126200	-0.53897700
C	-3.71385000	-0.82869700	3.09966100
H	-1.85165800	-1.54856900	2.24558200
C	-4.94261900	-0.22258000	2.83131400
H	-6.24498100	0.62413300	1.32897700
H	-3.46329300	-1.13591400	4.11535400
H	-5.64923000	-0.05165700	3.64461700
H	1.29528900	2.53385100	-1.17053100

Ph-5-12345-R5-Nu4

C	-1.11740000	-1.07793000	-0.01529000
C	-1.65407200	-1.35021800	1.40894200
C	0.20652800	-1.75684800	-0.22783100
C	-0.92327600	-0.50801500	2.43516400
H	-2.73069700	-1.14257300	1.42411500
H	-1.51206300	-2.41922800	1.61495000
C	-1.02279200	0.36861800	-0.41633100
C	1.34586000	-1.06227800	-0.31373900
C	-1.55170200	0.81036100	2.79904500
C	0.22930800	-0.92535100	2.96941800
C	0.18412400	1.03944700	-0.43029400
C	1.40895500	0.37744200	-0.31689300
H	-2.59901000	0.66821700	3.10666700
H	-1.00559700	1.29539300	3.61860700
H	-1.55242100	1.50606800	1.94540800
H	0.67156000	-1.88627100	2.69298200
H	0.76794600	-0.31370400	3.69706100
N	0.19188100	2.52523000	-0.50958500
O	-0.07279000	3.11716600	0.50266600
O	0.47209700	3.00162200	-1.57189300
N	2.57019500	1.02163600	0.18830500
O	2.65192500	2.23491400	0.12032400
O	3.43401400	0.31582600	0.68111300
N	2.56808500	-1.83994000	-0.60197400
O	3.30281300	-1.40531400	-1.45148600
O	2.71912300	-2.87829700	-0.00621700
N	-2.05790100	-1.79762300	-1.03373800
O	-2.02241500	-1.42058600	-2.17479100
O	-2.69988200	-2.72752500	-0.62222600
N	-2.25755700	1.05036500	-0.61096400
O	-3.28023100	0.43580400	-0.34634200
O	-2.25402400	2.19254300	-1.02873000
H	0.21912700	-2.84617300	-0.29656900

Ph-5-21345-R1

C	-1.21395400	-0.26097100	0.06884300
C	0.00975600	-0.94150500	-0.04713300
C	-1.22573100	1.14188900	-0.01243900
C	1.22168300	-0.22651400	0.03111900
C	-0.02614100	1.83356500	-0.09903400
C	1.19384300	1.17124200	-0.00840100

C	2.54324100	-0.96097500	0.29413800
C	0.03110000	-2.41061600	-0.50692900
C	2.43904200	2.05351100	-0.02781900
C	-2.49910600	1.96901100	-0.19188100
C	-2.50999400	-0.97985500	0.46220700
F	-3.48819200	-0.75289300	-0.40538100
F	-2.37264200	-2.28929400	0.60087700
F	-2.89070400	-0.52193400	1.65337200
F	-3.42089700	1.74118200	0.73267700
F	-3.03967200	1.71199500	-1.38074400
F	-2.21933900	3.26985900	-0.15655200
F	3.41845200	1.50518800	-0.73958000
F	2.89282100	2.30516000	1.19393200
F	2.15503900	3.22799300	-0.58932900
F	2.32109300	-2.11830100	0.91191500
F	3.24824700	-1.19414600	-0.80072300
F	3.29399900	-0.23530000	1.11984500
F	-0.97705300	-2.62139000	-1.35191500
F	1.14079400	-2.64272600	-1.20703600
F	-0.03347400	-3.30187300	0.46664700
H	-0.03867900	2.91564300	-0.20805000

Ph-5-21345-R1-Nu1

C	0.85101800	-0.02569400	-0.29671400
C	0.50738800	1.47092300	-0.40666000
C	-0.48940200	-0.82048500	-0.29656100
C	-0.73142200	1.95450000	-0.11829000
C	-1.72695000	-0.22790800	-0.12095200
C	-1.87236100	1.16578200	0.10521200
C	1.55094900	2.52788500	-0.62490400
C	-3.08145800	1.86144200	0.62414200
C	-3.02450800	-1.03041500	-0.26634800
C	-0.39892600	-2.31250800	-0.53490600
C	1.56405100	-0.38610200	-1.65376000
F	0.64955800	-0.28430200	-2.63332400
F	2.53066700	0.46777600	-1.98487600
F	2.07046300	-1.60888500	-1.75039100
F	0.77330700	-2.82869700	-0.13089200
F	-0.52385700	-2.67268700	-1.82058700
F	-1.31644100	-3.01147500	0.15246200
F	-4.04795200	-0.23692600	-0.58930500
F	-2.96859300	-1.92794600	-1.25319000
F	-3.37011200	-1.66558500	0.85549000
F	-3.97581700	2.27276800	-0.29766700
F	-2.73349600	2.98928100	1.27714100
F	-3.77413600	1.12210500	1.50954400
F	1.74039600	2.87546500	-1.90900700
F	1.23253700	3.68074600	-0.00248000
F	2.76361700	2.19681000	-0.14122700
C	1.72753400	-0.24989100	1.06940900
C	3.26010200	-0.10195200	0.89366600
C	1.62590000	-1.62415300	1.80045700

C	1.18209800	0.73247800	2.13166000
O	3.83199300	-0.57622700	-0.05151200
C	4.03404300	0.55467200	2.01534700
O	2.60964500	-2.30665400	1.95627400
C	0.34313700	-1.99695000	2.51688900
H	1.45922700	1.76797500	1.90841000
H	0.08582600	0.68029000	2.16867400
H	1.57344000	0.48588800	3.12817600
H	3.73051500	1.60097300	2.14646400
H	3.84846000	0.02401200	2.95998300
H	5.10055800	0.50375700	1.77020300
H	0.44674100	-1.67570300	3.56419600
H	-0.55836900	-1.53651000	2.10287900
H	0.25278000	-3.08924200	2.50234900
H	-0.85070200	3.03583200	-0.05078400

Ph-5-21345-R1-Nu3

C	-1.12539000	0.51370400	-0.09459400
C	-0.93835000	-0.63304900	-1.10218200
C	0.19844800	1.32966200	-0.07466600
C	0.30768800	-1.14269300	-1.32794300
C	1.42404600	0.74253300	-0.37012000
C	1.51129600	-0.56738400	-0.89627600
C	-2.04495300	-1.46195700	-1.70742800
C	2.72042900	-1.44238300	-0.95399600
C	2.75127800	1.48204800	-0.16888800
C	0.18086100	2.78561100	0.34170900
C	-2.32087000	1.42773300	-0.55483200
F	-1.89682900	2.39664200	-1.36872200
F	-3.24430300	0.75533600	-1.23925700
F	-2.99866500	1.99806100	0.44606800
F	-0.97732000	3.18074300	0.88026000
F	0.41027700	3.64416800	-0.66241200
F	1.09959600	3.05498600	1.29325200
F	3.72517700	0.95118900	-0.91012900
F	2.72466000	2.76645200	-0.52240700
F	3.15573000	1.41854200	1.10440500
F	3.51563400	-1.28059700	-2.03060700
F	2.36535300	-2.74089800	-0.99349800
F	3.51671800	-1.31325200	0.12249700
F	-2.54403200	-0.97218300	-2.85741700
F	-1.61879300	-2.69889700	-2.02834900
F	-3.09197700	-1.66401300	-0.89148200
C	-1.45115900	-0.07597000	1.40978800
C	-0.58591400	-1.31911900	1.66075700
C	-2.86994600	-0.46091400	1.54761700
C	-1.21621800	0.95093400	2.54068400
C	0.72001100	-1.18327600	2.14605800
C	-1.06739500	-2.60456100	1.38730700
N	-3.97519300	-0.72811900	1.74449700
H	-1.94326500	1.76616500	2.49637400
H	-1.31070000	0.43440600	3.50492400

H	-0.20838300	1.37667000	2.47899700
C	1.52744700	-2.30361500	2.33730100
H	1.13229400	-0.19548000	2.35179500
C	-0.26072000	-3.72524100	1.57793300
H	-2.08372300	-2.73295700	1.01158700
C	1.04252300	-3.57818300	2.05079600
H	2.54812300	-2.17242100	2.69879700
H	-0.65578100	-4.71717400	1.35333600
H	1.67746000	-4.45353100	2.19622700
H	0.38062700	-2.07864900	-1.87749000

Ph-5-21345-R1-Nu2

C	-0.20314000	0.42597000	0.80088000
C	-1.12389700	-0.80443800	0.69202100
C	-0.91959400	1.64859600	0.24090200
C	-2.31909600	-0.78702900	-0.01889100
C	-2.05305200	1.55213400	-0.49845500
C	-2.77525400	0.36014100	-0.70479400
C	-3.25308600	-2.00247700	-0.02212600
C	-0.61187100	-2.10664200	1.27445100
C	-3.92171200	0.43191800	-1.65706300
C	-0.33668100	3.02023900	0.40829500
C	0.06411700	0.72706300	2.31375300
F	-1.05702300	1.20608900	2.85767800
F	0.40370500	-0.32128800	3.05111300
F	1.01095800	1.65424000	2.47301100
F	0.99476700	3.04215300	0.29475700
F	-0.61578800	3.58001600	1.60255500
F	-0.81109700	3.88104100	-0.50527000
F	-5.12999700	0.64928900	-1.10182800
F	-4.05165200	-0.67016700	-2.41481600
F	-3.75544100	1.45421500	-2.51809200
F	-3.00731200	-2.83935400	-1.02951200
F	-3.20530200	-2.70227000	1.11195900
F	-4.52810200	-1.62001400	-0.13274400
F	-1.09930200	-2.42035700	2.48161800
F	-0.87185100	-3.15367000	0.47265900
F	0.72843500	-2.12360800	1.41885900
C	1.14849000	0.10931300	-0.03542000
Br	1.11991900	0.82075000	-1.83955200
S	2.85009100	0.36208300	0.65390500
H	1.17409800	-0.96596000	-0.20919700
O	3.30082600	1.73984900	0.52756800
O	2.86807600	-0.30702300	1.95085900
C	3.82748100	-0.63955900	-0.45566800
C	4.59772600	-0.01820800	-1.43491400
C	3.82005300	-2.02255100	-0.27953200
C	5.37527100	-0.81397700	-2.27441700
H	4.57915900	1.06832800	-1.52739600
C	4.59659600	-2.80614900	-1.13087200
H	3.22060700	-2.47018500	0.51559800
C	5.37017800	-2.20196700	-2.12416100

H	5.98687500	-0.34888600	-3.04830800
H	4.60382300	-3.89023300	-1.01327000
H	5.97859300	-2.81986800	-2.78609100
H	-2.43314200	2.46352200	-0.95837000

Ph-5-21345-R1-Nu4

C	1.22288100	-0.44293300	0.03409400
C	1.91688700	-0.77255900	1.40802900
C	1.24757600	1.07374300	-0.20413900
C	1.17726300	-0.23316400	2.60949300
H	2.05419000	-1.85543900	1.50412800
H	2.91502900	-0.32628900	1.37739700
C	-0.23630700	-0.95663800	-0.09164700
C	0.11671500	1.82634100	-0.19035900
C	0.05197800	-1.05735600	3.17748700
C	1.53192400	0.93912400	3.14787400
C	-1.32205800	-0.10390800	-0.21418200
C	-1.19344200	1.30623000	-0.15547700
H	0.38608000	-2.08736200	3.37654200
H	-0.32335000	-0.62017800	4.11247100
H	-0.78805200	-1.12930100	2.47077900
H	2.35183000	1.52566200	2.72596800
H	1.00770700	1.34732400	4.01588200
C	2.52794600	1.82520900	-0.41334600
C	-2.29906800	2.29445500	-0.04732600
C	-2.72509600	-0.64347100	-0.50821800
C	-0.47667000	-2.44121900	0.06702300
C	2.07738400	-1.11180500	-1.06991200
F	1.68824800	-0.73583500	-2.28787100
F	3.37490400	-0.79918800	-0.94923400
F	2.02684500	-2.44778400	-1.04564500
F	0.46254500	-3.05431200	0.80695800
F	-0.53414200	-3.13331200	-1.08337500
F	-1.62418900	-2.70810100	0.72098500
F	-3.42558700	0.23260200	-1.23618000
F	-2.71300200	-1.75813200	-1.24304400
F	-3.43758000	-0.88608100	0.59308700
F	-2.83501600	2.71541100	-1.21422000
F	-1.86835200	3.42571900	0.54764500
F	-3.33443500	1.86084000	0.69437600
F	3.04740500	1.66204600	-1.64791100
F	2.36643400	3.15099700	-0.27426600
F	3.52436300	1.50004600	0.43653200
H	0.22309900	2.90903800	-0.24006800

Ph-5-21345-R2

C	-1.21548000	-0.28815200	0.00034700
C	-0.00007700	-0.99099500	0.00060200
C	-1.20765900	1.11843700	0.00002700
C	1.21543400	-0.28814400	0.00059100
C	0.00002500	1.81762500	0.00005400

C	1.20769500	1.11848800	0.00002400
C	2.46124300	-1.00747200	0.00021800
N	3.46138800	-1.58138300	-0.00004400
C	-0.00013000	-2.42990900	-0.00012500
N	0.00002900	-3.58288500	-0.00068300
C	2.45293400	1.84273700	-0.00026300
N	3.44845900	2.42452800	-0.00027500
C	-2.45286400	1.84281200	-0.00008200
N	-3.44831400	2.42472500	-0.00026000
C	-2.46127800	-1.00746200	0.00027000
N	-3.46142700	-1.58136200	-0.00012800
H	-0.00001100	2.90686300	-0.00024900

Ph-5-21345-R2-Nu1

C	0.54243600	0.47505000	-0.55079800
C	-0.83722400	1.13175100	-0.42990200
C	0.35515200	-0.79656100	-1.38312100
C	-1.95546800	0.32987300	-0.28096400
C	-0.77790500	-1.54787400	-1.23723500
C	-1.91492100	-1.06359100	-0.53576800
C	-3.21115100	0.93822500	0.08574300
N	-4.21438500	1.41697600	0.39768200
C	-0.95012800	2.54834000	-0.37939700
N	-1.02639200	3.70423400	-0.32557000
C	-3.04756600	-1.89145000	-0.30085200
N	-3.96344400	-2.57313200	-0.09871900
C	1.33803200	-1.21704600	-2.33124800
N	2.08276300	-1.57528700	-3.14251800
C	1.43185600	1.41727900	-1.26476900
N	2.07392400	2.19966800	-1.81664000
C	1.17707500	0.21483300	0.89367200
C	2.59981100	-0.36052200	0.75437800
C	0.36786000	-0.87125800	1.63471700
C	1.25520300	1.52395100	1.69149100
O	3.11255300	-0.52561900	-0.32241700
C	3.35710500	-0.63820600	2.03291600
O	0.65895300	-2.02999300	1.45166700
C	-0.68733200	-0.46640100	2.63167100
H	1.89375300	2.25460600	1.17354700
H	0.26325100	1.97305200	1.82428000
H	1.67682500	1.34876600	2.69027000
H	3.91253100	0.27292700	2.30351300
H	2.70312300	-0.90544900	2.87176200
H	4.08258800	-1.43832700	1.84643700
H	-0.17798200	-0.18153100	3.56675100
H	-1.27396400	0.40013200	2.30507500
H	-1.33745700	-1.32557700	2.83207900
H	-0.83899000	-2.51927500	-1.73055700

Ph-5-21345-R2-Nu3

C	0.09789700	0.57387900	0.45682900

C	1.56358600	0.97036700	0.29016800
C	0.04399100	-0.81247300	1.10001000
C	2.54209900	0.00463000	0.12206300
C	1.05494100	-1.71961400	0.92470900
C	2.29814200	-1.37277000	0.34042300
C	3.88009100	0.42866600	-0.21506100
N	4.95090700	0.76149600	-0.48863700
C	1.90177100	2.35248300	0.25753000
N	2.15179900	3.48435200	0.23462100
C	3.30978100	-2.34969900	0.12924700
N	4.12737300	-3.15331600	-0.04354600
C	-1.09586000	-1.16781500	1.88454500
N	-2.00586500	-1.44993500	2.54450800
C	-0.55998000	1.55081000	1.35286100
N	-1.05641400	2.31614200	2.05795700
C	-0.69336000	0.61594700	-0.94662200
C	-2.12035500	0.09680900	-0.76411500
C	0.03400700	-0.27257200	-1.87367700
C	-0.65226200	2.02967900	-1.55735300
C	-3.12849100	0.96105700	-0.31892700
C	-2.43359400	-1.24972200	-0.98475800
N	0.59327500	-0.93583600	-2.63356500
H	0.37044000	2.30078500	-1.84692700
H	-1.28953200	2.06135200	-2.45032300
H	-1.01406100	2.77545700	-0.83727300
C	-4.41613200	0.48247900	-0.08434200
H	-2.91546500	2.01509300	-0.14170200
C	-3.72233700	-1.72613800	-0.75275400
H	-1.66222700	-1.93661100	-1.33748100
C	-4.71673700	-0.86238000	-0.29752400
H	-5.18821400	1.16835700	0.26670600
H	-3.94670300	-2.77922700	-0.92733000
H	-5.72530500	-1.23476400	-0.11258700
H	0.92036300	-2.73249000	1.30803800

Ph-5-21345-R2-Nu2

C	-0.95498000	0.35663000	-0.08264600
C	-1.91247400	-0.73766800	-0.55019500
C	-1.80259200	1.63242100	-0.17799300
C	-3.13841400	-0.78432000	0.09068800
C	-3.02468000	1.59227300	0.45530800
C	-3.61348000	0.35337000	0.80740900
C	-4.00717800	-1.91664100	-0.10014700
N	-4.70723200	-2.82366000	-0.24507500
C	-1.61023900	-1.60176400	-1.63732500
N	-1.38317500	-2.27618500	-2.55183500
C	-4.82533800	0.30109500	1.55637000
N	-5.80543600	0.26379600	2.17284100
C	-1.43742900	2.79191100	-0.91915100
N	-1.21123300	3.74595400	-1.53739800
C	-0.62033200	0.14754300	1.36006900
N	-0.36087300	0.02821700	2.47733200

C	0.36929300	0.38452600	-0.86786100
Br	1.48697700	1.88447700	-0.38725800
S	1.35454300	-1.21880300	-0.70508900
H	0.18361100	0.44402400	-1.94837500
O	0.54582600	-2.15282200	0.07275700
O	1.75867200	-1.52195000	-2.07209400
C	2.82402800	-0.88545600	0.23875900
C	2.78263800	-1.02437000	1.62435900
C	3.99319200	-0.56412900	-0.44854500
C	3.95594800	-0.80519900	2.34434100
H	1.85148900	-1.29457000	2.12372900
C	5.15705900	-0.35350900	0.28571500
H	3.98157700	-0.48465300	-1.53606600
C	5.13502900	-0.46920900	1.67750200
H	3.94859000	-0.90407900	3.43013400
H	6.08412600	-0.10086900	-0.22968200
H	6.04958000	-0.30150800	2.24805700
H	-3.59650300	2.51211600	0.59134000

Ph-5-21345-R2-Nu4

C	0.99210000	-0.02310900	-0.59543500
C	2.10792800	0.60607900	0.29874700
C	-0.29167100	0.80728900	-0.53736900
C	1.65458400	0.96635400	1.69335900
H	2.94176600	-0.11566500	0.34105300
H	2.48474400	1.50793000	-0.20498600
C	0.67961300	-1.47907100	-0.23344200
C	-1.52588900	0.26552200	-0.25133100
C	1.14327200	-0.13773000	2.58012300
C	1.72101500	2.23450400	2.11143500
C	-0.57914600	-1.94755900	0.01796000
C	-1.72637900	-1.11586900	0.01867600
H	1.79810600	-1.02161000	2.53334300
H	1.07282000	0.19974600	3.62246100
H	0.14207400	-0.46547600	2.25816600
H	2.06537100	3.03340900	1.45044100
H	1.42420300	2.50978900	3.12643700
C	-2.66944700	1.14760900	-0.21152600
N	-3.58868400	1.84465800	-0.17685400
C	-0.14825900	2.20223700	-0.79064200
N	0.01420800	3.32802300	-1.01423600
C	-3.01533500	-1.63888200	0.30149400
N	-4.06547000	-2.07489500	0.53334000
C	1.79204400	-2.36920100	-0.16312200
N	2.71597700	-3.06742800	-0.10343300
C	1.51079200	-0.00216400	-1.99281100
N	1.90986400	0.00697600	-3.07584900
H	-0.70168000	-3.00550700	0.25560100

Ph-5-21345-R3

C	-1.21354900	-0.38186300	0.10286800

C	-1.46499400	0.99231700	0.03320800
C	0.10514200	-0.85010700	0.04020100
C	-2.34726200	-1.36144200	0.31102300
C	-2.87959900	1.48483300	-0.03695300
C	-0.40199300	1.89064300	-0.03525200
C	0.34119900	-2.33413200	0.10739500
C	1.17212200	0.05340800	-0.05097800
O	-2.54015200	-2.10779100	-0.76568000
O	-2.95667300	-1.45195100	1.33817800
O	-2.98085300	2.77155700	0.26810600
O	-3.80983700	0.79335200	-0.36039000
C	0.91241900	1.42896700	-0.05803000
O	1.35314800	-2.71371600	-0.65962000
O	-0.32286400	-3.08527600	0.77102000
C	2.59351200	-0.44834200	-0.17330200
C	-3.50700700	-3.14499900	-0.62736800
C	-4.28749100	3.33622600	0.18168300
C	2.05321100	2.40129800	-0.00604100
C	1.70040400	-4.09652300	-0.61200400
O	2.99365800	-1.02429300	0.95193300
O	3.24708600	-0.35240000	-1.17219800
H	-3.55424100	-3.65203800	-1.59482300
H	-3.19005400	-3.84204500	0.15974100
H	-4.48422000	-2.71991700	-0.36609000
H	-4.18299700	4.38908900	0.45607800
H	-4.96669200	2.82286600	0.87379300
H	-4.67496600	3.23979400	-0.84044100
O	1.69963500	3.61483100	-0.40717700
O	3.15466400	2.11489300	0.38527100
H	2.52666900	-4.22593900	-1.31597200
H	0.84096800	-4.71106300	-0.90657600
H	2.01021100	-4.37038000	0.40494500
C	4.31110300	-1.56645000	0.92918600
C	2.71142400	4.61762200	-0.34069300
H	5.04065200	-0.77333900	0.72125900
H	4.38734800	-2.33873400	0.15211000
H	4.48300600	-1.99785400	1.91888800
H	3.56149100	4.33849600	-0.97570600
H	3.05867600	4.73696100	0.69341300
H	2.24952500	5.54061800	-0.70046900
H	-0.59971900	2.96109700	-0.06536700

Ph-5-21345-R3-Nu1

C	0.88608900	-0.20488900	0.10125600
C	0.82332600	1.29802800	0.36000800
C	-0.51263900	-0.78541300	0.37537400
C	1.89692300	-0.85893000	1.05879000
C	2.05790600	2.00063200	0.69444900
C	-0.34520700	2.00257300	0.22329900
C	-0.60532900	-2.19992400	0.80329600
C	-1.64533100	-0.02894800	0.22211900
O	1.68493700	-0.48806300	2.31872900

O	2.75882400	-1.63002800	0.73024900
O	1.91228400	3.33721900	0.82900000
O	3.14984400	1.48018600	0.81956500
C	-1.60072300	1.39183100	0.04052600
O	-1.72214500	-2.81194300	0.38465100
O	0.20951600	-2.77822200	1.48727300
C	-3.01613400	-0.64482200	0.37077500
C	2.51028800	-1.11938900	3.27991900
C	3.09622100	4.06036600	1.11095200
C	-2.80781600	2.17982600	-0.12433200
C	-1.88023700	-4.15766200	0.79346500
O	-3.52976100	-0.96457100	-0.81546300
O	-3.58496900	-0.80860000	1.41690900
H	2.20445600	-0.73228500	4.25692800
H	2.36781600	-2.20781600	3.23619000
H	3.56723700	-0.88565300	3.09101900
H	2.80412000	5.11228200	1.19210200
H	3.83358800	3.93369200	0.30590700
H	3.54899700	3.72020100	2.05214600
O	-2.55889700	3.50901400	-0.23589200
O	-3.95153500	1.76239600	-0.17044000
H	-2.82816200	-4.50119000	0.36692800
H	-1.90631700	-4.23517500	1.88901700
H	-1.04899900	-4.76795000	0.41532600
C	-4.86779900	-1.42819000	-0.78758300
C	-3.69631600	4.33812300	-0.36889800
H	-5.52000200	-0.65807800	-0.35372600
H	-4.94855000	-2.34554500	-0.18785000
H	-5.15108800	-1.62821600	-1.82563500
H	-4.36028500	4.24051500	0.50138400
H	-4.26949400	4.07924300	-1.26996900
H	-3.32481100	5.36558500	-0.44315200
C	1.37594800	-0.53397600	-1.40864500
C	1.54566300	-2.06179300	-1.61388000
C	2.66413500	0.25762200	-1.73824300
C	0.26356100	-0.13245800	-2.39640500
O	0.58385000	-2.77263800	-1.41614300
C	2.80323400	-2.67367700	-2.18054800
O	3.76774400	-0.21211000	-1.60558300
C	2.52339800	1.64684100	-2.33363600
H	-0.62155500	-0.75230700	-2.21380100
H	-0.01728200	0.92080200	-2.27385100
H	0.59255000	-0.29085700	-3.43509900
H	2.58810700	-3.72619700	-2.39806000
H	3.12178100	-2.15068900	-3.09237900
H	3.61130600	-2.57322900	-1.44959500
H	2.25760700	1.54648200	-3.39748200
H	1.73799800	2.24411000	-1.85645000
H	3.49470500	2.14886700	-2.25676200
H	-0.30622900	3.08764800	0.31165800

Ph-5-21345-R3-Nu3

C	0.34029700	0.93318000	0.11318000
C	-0.98767200	1.68821900	0.07009500
C	0.08631300	-0.49245500	0.61539700
C	1.29721600	1.67260800	1.06849700
C	-0.99388600	3.15049700	0.18391500
C	-2.16677100	1.01644700	-0.09877100
C	1.22276900	-1.17629500	1.25632600
C	-1.12490300	-1.11180200	0.39585000
O	0.76790300	1.74594200	2.28966000
O	2.35889700	2.15177800	0.77728300
O	-2.20212900	3.70395400	-0.05714100
O	-0.04117000	3.84288200	0.47627000
C	-2.27053300	-0.39316700	-0.05422700
O	1.10191800	-2.51380800	1.30920700
O	2.20697200	-0.62455700	1.70118900
C	-1.32423000	-2.57968600	0.69266400
C	1.55571900	2.42653600	3.24791700
C	-2.26776100	5.11321600	0.06254300
C	-3.52612300	-1.06828400	-0.33948200
C	2.18135600	-3.21255900	1.90317500
O	-1.02792000	-3.31499500	-0.38104700
O	-1.71632800	-3.03203000	1.73229000
H	1.00505100	2.38380200	4.19292600
H	2.53362300	1.93693600	3.35245100
H	1.70652900	3.47065100	2.94049400
H	-3.30350000	5.39379300	-0.15411000
H	-1.58707700	5.59814100	-0.65061800
H	-1.99324000	5.43368300	1.07687100
O	-4.52393300	-0.21109600	-0.66094700
O	-3.72406000	-2.26930800	-0.32083700
H	1.91629900	-4.27426600	1.87061000
H	2.33197600	-2.89040700	2.94205000
H	3.10665900	-3.03104000	1.33834200
C	-1.26900700	-4.70495800	-0.24676700
C	-5.77509700	-0.80329700	-0.95220500
H	-2.33029300	-4.88212500	-0.02626700
H	-0.65942100	-5.12560700	0.56509700
H	-0.99598500	-5.16307000	-1.20243800
H	-6.14950600	-1.37540600	-0.09211400
H	-5.69786900	-1.48302300	-1.81205000
H	-6.46059200	0.01834800	-1.18402100
C	1.01195400	0.85920700	-1.35113600
C	2.23577800	-0.06914900	-1.39424600
C	1.40584500	2.21018600	-1.80398100
C	-0.02558800	0.38920500	-2.39664000
C	2.05418200	-1.45334100	-1.52930700
C	3.54290500	0.42285500	-1.30491700
N	1.66146800	3.23276200	-2.27501400
H	-0.82150500	1.13395800	-2.51113600
H	0.47388500	0.24690500	-3.36445100
H	-0.48244300	-0.55825600	-2.08953000
C	3.14729700	-2.32003700	-1.53152200
H	1.05191300	-1.87576600	-1.59803100

C	4.63605700	-0.44167100	-1.32451600
H	3.70126000	1.49520300	-1.19864000
C	4.44379900	-1.81925100	-1.42565500
H	2.97807500	-3.39493700	-1.61975300
H	5.64524500	-0.03285400	-1.25192900
H	5.29947600	-2.49662100	-1.43214100
H	-3.07953800	1.59529700	-0.23105600

Ph-5-21345-R3-Nu2

C	1.14834800	0.04462200	0.53761200
C	1.78764000	1.19130300	-0.23344900
C	-0.06847000	0.59661200	1.29841500
C	2.14360100	-0.63871900	1.47338800
C	3.22584600	1.17958800	-0.51320100
C	0.96202200	2.11085200	-0.82083700
C	-0.39235000	0.05817800	2.63638800
C	-0.86320300	1.52051600	0.66116900
O	2.76659300	0.23753100	2.24708900
O	2.32488000	-1.82740000	1.50042600
O	3.63725400	2.26922600	-1.19545000
O	4.00768600	0.30693300	-0.19932900
C	-0.41735800	2.19996500	-0.51640900
O	-1.69912100	0.14999900	2.93086700
O	0.39676800	-0.41242600	3.42214600
C	-2.17100500	1.98880800	1.25609800
C	3.64731100	-0.31783100	3.20540300
C	5.00950900	2.29482600	-1.54335000
C	-1.28675600	3.13039200	-1.22330500
C	-2.08412900	-0.35054100	4.19838900
O	-3.20081800	1.27505200	0.79967000
O	-2.27856900	2.90663900	2.02280900
H	4.07202700	0.52277400	3.76283000
H	3.09065300	-0.98225800	3.88025000
H	4.44460400	-0.88667300	2.70784300
H	5.17184100	3.22626800	-2.09508300
H	5.27111800	1.43191900	-2.17126700
H	5.64017800	2.27338400	-0.64421100
O	-0.66791400	3.74741200	-2.25512000
O	-2.45295900	3.36543600	-0.96783000
H	-3.16558300	-0.19894900	4.27477800
H	-1.56707900	0.18599400	5.00513700
H	-1.84193400	-1.41925500	4.27156300
C	-4.48075000	1.74598300	1.18615500
C	-1.45318500	4.67429400	-2.98117700
H	-4.62531400	2.76974800	0.81532600
H	-4.58319300	1.74011100	2.27977800
H	-5.21198300	1.06895200	0.73340900
H	-1.80359100	5.48747500	-2.33065300
H	-2.33029600	4.18306400	-3.42464100
H	-0.80848400	5.07516300	-3.77000800
C	0.59031900	-0.97795400	-0.50238300
Br	1.82004200	-1.53213000	-1.89448700

S	-0.24537200	-2.50634500	0.14557500
H	-0.20914500	-0.45291700	-1.03933500
O	0.53645800	-3.70637700	-0.11943700
O	-0.72587600	-2.24773300	1.50111400
C	-1.67745200	-2.54375200	-0.92092900
C	-1.71839500	-3.44768600	-1.97751800
C	-2.70987000	-1.64055800	-0.66585500
C	-2.83818900	-3.44663900	-2.81028700
H	-0.88570300	-4.13476800	-2.13289200
C	-3.81623700	-1.64615800	-1.51217300
H	-2.64325100	-0.93949400	0.17130000
C	-3.87989200	-2.54794700	-2.57815200
H	-2.89499900	-4.14840000	-3.64325200
H	-4.63194800	-0.94317500	-1.33832100
H	-4.75061800	-2.54785700	-3.23552600
H	1.38799700	2.83170300	-1.51876900

Ph-5-21345-R3-Nu4

C	1.25094700	-0.08857700	0.36498600
C	1.33420900	-0.17809200	1.92261400
C	-0.06172100	-0.68184300	-0.14386400
C	0.95576800	-1.49770900	2.55949600
H	2.35477300	0.11914200	2.21094700
H	0.63734800	0.58795500	2.29176200
C	1.27030300	1.38969500	-0.03389200
C	2.45132700	-0.80693700	-0.25878600
C	-0.05852700	-2.09492400	-0.52240500
C	-1.21595600	0.06468100	-0.07036000
C	2.00643600	-2.56355000	2.71574300
C	-0.30495900	-1.70002100	2.96284300
C	2.54418700	2.07384900	-0.23801000
C	0.10018500	2.09503200	-0.00285500
O	2.39505800	-0.77016600	-1.59555100
O	3.35021300	-1.33309600	0.34062900
O	-1.17606800	-2.49611900	-1.15979500
O	0.85858200	-2.86869400	-0.32817200
C	-1.16656400	1.47681900	0.13975600
C	-2.56427400	-0.59362200	-0.22658500
H	2.26712600	-2.96965000	1.72946600
H	2.92990200	-2.14715600	3.14560700
H	1.64485300	-3.37753200	3.35980900
H	-1.07636800	-0.93903300	2.81759900
H	-0.60879600	-2.64497800	3.42215200
O	2.41504400	3.39286400	-0.50419700
O	3.64334800	1.55690000	-0.18417500
C	3.44152900	-1.44961600	-2.26309400
C	-1.27730200	-3.88178100	-1.43098500
C	-2.31491600	2.35389400	0.31031200
O	-3.11928700	-0.31106200	-1.40545400
O	-3.09477500	-1.26854500	0.61495900
C	3.62304100	4.10769600	-0.68120100
H	3.26385500	-1.32288500	-3.33586500

H	4.41575000	-1.02533700	-1.98426100
H	3.42785200	-2.51623900	-1.99776800
H	-2.23929000	-4.03004800	-1.93224000
H	-1.24168700	-4.46481300	-0.50029700
H	-0.45645600	-4.21143800	-2.08175900
O	-3.47630400	1.69851500	0.54034200
O	-2.28509400	3.57035500	0.28542200
C	-4.42496900	-0.82507300	-1.59886500
H	3.34173700	5.14764200	-0.87620200
H	4.19589100	3.70685800	-1.52876500
H	4.25091100	4.04938700	0.21884500
C	-4.62930600	2.50527300	0.68197600
H	-4.42354900	-1.92166200	-1.52967000
H	-5.10885500	-0.42558900	-0.83676800
H	-4.74135800	-0.50816000	-2.59734700
H	-4.82720900	3.07333000	-0.23799600
H	-4.50968600	3.21733900	1.50969400
H	-5.46111000	1.82392000	0.88966900
H	0.12199100	3.18016900	-0.10962600

Ph-5-21345-R4

C	1.20815600	0.42474800	-0.00023100
C	1.19352100	-0.96790700	0.00043700
C	0.00005500	1.11679000	0.00058300
C	-0.00011200	-1.67577900	-0.00023800
C	-1.20798400	0.42442100	-0.00036600
C	-1.19394600	-0.96804600	0.00073000
F	2.35420500	-1.60734200	0.00035700
F	-2.35432600	-1.60763400	-0.00022300
H	0.00079700	-2.76496200	-0.00010100
F	-2.35052400	1.09117100	-0.00055000
F	-0.00017500	2.43768200	0.00089500
F	2.35093700	1.09052300	-0.00107700

Ph-5-21345-R4-Nu1

C	0.11324400	-0.21849800	-0.37938100
C	0.77228700	-1.46401800	0.03727000
C	1.08519800	0.85172800	-0.17247500
C	2.12500100	-1.65492100	0.20626300
C	2.44924100	0.68529800	-0.03548400
C	3.00560600	-0.57688600	0.15034800
F	0.63236500	2.13118100	-0.32585100
F	3.23758200	1.77175700	-0.04285500
F	4.34737600	-0.72951500	0.27517800
F	-0.03965900	-2.53725200	0.26694000
F	-0.25204800	-0.21697700	-1.81421200
C	-1.28701400	-0.00110700	0.31459200
C	-2.36350600	-1.00684600	-0.16361800
C	-1.88486400	1.36728600	-0.08625700
C	-1.11263000	-0.12870300	1.83471800
O	-2.23693500	-1.77063800	-1.08110100

C	-3.65679900	-0.98305800	0.63595500
O	-2.46694600	1.48578300	-1.13858900
C	-1.83752000	2.50970700	0.90301000
H	-0.87715300	-1.17071200	2.09138000
H	-0.27502200	0.49525400	2.17227200
H	-2.01458600	0.17236900	2.38380500
H	-3.51177900	-1.54972500	1.56799800
H	-3.96379200	0.03601800	0.90933800
H	-4.44385900	-1.46909900	0.04835800
H	-2.65235500	2.36989800	1.63103100
H	-0.89183300	2.55174000	1.45199200
H	-2.00953000	3.44739700	0.36237200
H	2.49093800	-2.65694100	0.43654100

Ph-5-21345-R4-Nu3

C	-0.47726000	-0.94099800	0.10740300
C	-0.67351900	-0.59544500	-1.28757300
C	-1.38747600	-0.15000100	0.89568200
C	-1.75855700	0.06179800	-1.82081500
C	-2.50049400	0.50681000	0.39324400
C	-2.71497600	0.61553900	-0.97343300
F	-1.23382200	-0.10710900	2.24739300
F	-3.34915300	1.08389300	1.25511100
F	-3.81100700	1.24803100	-1.44693700
F	0.29601500	-1.01308400	-2.14552600
F	-0.80722000	-2.47876000	0.32424000
C	1.03056000	-1.01242200	0.55403200
C	1.72264400	0.32073900	0.26501400
C	1.68651100	-2.10513200	-0.19490500
C	1.20153900	-1.42001400	2.03763400
C	1.34429500	1.45002500	1.00555400
C	2.69435300	0.46315600	-0.72811000
N	2.27173300	-2.96541100	-0.69396600
H	0.58655600	-2.30590200	2.23240000
H	2.25765700	-1.65438200	2.22831400
H	0.89339800	-0.61283100	2.70660500
C	1.93177200	2.68843400	0.76047600
H	0.57784000	1.35836100	1.77714700
C	3.28449100	1.70518300	-0.97304200
H	2.99222300	-0.40197000	-1.32188900
C	2.90604600	2.82118300	-0.23124800
H	1.62400500	3.55633000	1.34590300
H	4.04271800	1.79600500	-1.75256000
H	3.36556200	3.79191600	-0.42396400
H	-1.83498000	0.18047500	-2.90221100

Ph-5-21345-R4-Nu2

C	1.45049200	0.12623300	-0.50288400
C	2.02431700	-1.21185300	-0.60927300
C	2.28579100	0.89236600	0.41060400
C	3.27613700	-1.59049100	-0.18403600

C	3.54837400	0.53348100	0.84428200
C	4.06966400	-0.72377800	0.56343600
F	1.79264000	2.08666700	0.83089500
F	4.23692300	1.39511700	1.60554300
F	5.31160900	-1.06464200	0.97824900
F	1.32245100	-2.08682100	-1.37329800
F	1.41247200	0.71100400	-1.87756700
C	-0.05096100	0.26099500	-0.05377200
Br	-0.93606500	1.85663800	-0.73934400
S	-1.14509300	-1.17758200	-0.37573600
H	-0.08176100	0.35351400	1.03798000
O	-1.35241400	-1.33993600	-1.80911800
O	-0.64232400	-2.26985600	0.45518900
C	-2.70688500	-0.68972900	0.34336300
C	-3.72972200	-0.24258500	-0.48966700
C	-2.87762100	-0.82507200	1.71971200
C	-4.95447700	0.09777800	0.08074600
H	-3.55530600	-0.16763200	-1.56353700
C	-4.10523400	-0.47379300	2.27876800
H	-2.06521500	-1.21350400	2.33590700
C	-5.13810300	-0.01280900	1.46065800
H	-5.76855900	0.45033000	-0.55356300
H	-4.25845300	-0.57005300	3.35417600
H	-6.09844300	0.25720300	1.90229700
H	3.63390000	-2.58889100	-0.43904300

Ph-5-21345-R4-Nu4

C	0.41429100	0.15587700	0.80686900
C	1.92510300	-0.09356100	0.74079200
C	-0.41718000	-0.93112600	0.35653000
C	2.46358900	-0.33747200	-0.64787700
H	2.41113600	0.78499700	1.19011400
H	2.13087600	-0.95826500	1.38317700
C	-0.08487500	1.38987400	0.24187500
C	-1.69932200	-0.77612400	-0.13929900
C	2.49443800	0.83484000	-1.59107800
C	2.89270600	-1.54964800	-1.01522800
C	-1.34916600	1.58418600	-0.26985200
C	-2.19131400	0.48795700	-0.45392700
H	3.00326100	1.69376700	-1.12768800
H	3.00333900	0.57876300	-2.53000300
H	1.47068300	1.16544900	-1.82469000
H	2.84896200	-2.39509700	-0.32444100
H	3.28956700	-1.73308600	-2.01724200
F	0.75487700	2.46181100	0.32339100
F	-3.45185700	0.62874600	-0.93071900
F	-2.44673700	-1.86499800	-0.38255900
F	0.03905700	-2.19564300	0.55350200
F	0.30702700	0.26909100	2.39709700
H	-1.66874500	2.58359200	-0.56846400

Ph-5-21345-R5

C	-1.20711900	-0.22846900	0.01076500
C	-0.00013000	-0.91806600	-0.00005000
C	-1.19135500	1.16152300	-0.00458700
C	1.20704100	-0.22883900	-0.01072500
C	0.00026000	1.86838800	0.00002300
C	1.19168500	1.16119600	0.00458800
N	2.45075500	1.93641400	0.08314800
O	2.41833100	3.05559800	-0.34950800
O	3.38828200	1.38592900	0.59793400
N	2.48069300	-0.99187600	-0.03478900
O	3.14847100	-0.86213400	-1.01933300
O	2.70010300	-1.66894800	0.92971700
N	-0.00039300	-2.39950000	-0.00008800
O	0.78791600	-2.92971500	-0.73385900
O	-0.78892000	-2.92943700	0.73363600
N	-2.48087000	-0.99136400	0.03479800
O	-2.70059900	-1.66783600	-0.93008400
O	-3.14832600	-0.86222500	1.01961000
N	-2.45026600	1.93699400	-0.08306100
O	-3.38813100	1.38639400	-0.59711000
O	-2.41739200	3.05640000	0.34897800
H	0.00040500	2.95872200	0.00002000

Ph-5-21345-R5-Nu1

C	0.63329200	0.01531400	0.36249100
C	-0.64990900	-0.81841400	0.44319500
C	0.20668200	1.45906300	0.53779800
C	-1.86911100	-0.31015100	0.07474400
C	-1.03030300	1.93485100	0.22891000
C	-2.07579400	1.07568700	-0.12211400
N	-3.32216600	1.65111900	-0.52616100
O	-3.38349400	2.86206800	-0.62265500
O	-4.25792100	0.91101100	-0.76187600
N	-3.03866100	-1.21124900	-0.10724900
O	-3.74184100	-1.39132300	0.84690200
O	-3.18428300	-1.65148500	-1.21340300
N	-0.57849100	-2.20342900	0.89158200
O	-1.45501300	-2.96570800	0.55874500
O	0.34667500	-2.51547300	1.60548600
N	1.52350700	-0.33416400	1.60172500
O	1.08883100	-0.00486300	2.67049600
O	2.56239800	-0.89446200	1.39920500
N	1.17949400	2.42888900	0.97465400
O	2.31680800	2.03578200	1.16752100
O	0.84144800	3.58492600	1.11381900
C	1.46737400	-0.25035700	-0.97265900
C	1.88797600	-1.74790400	-1.08197200
C	2.66272000	0.73755900	-1.12391100
C	0.50614300	-0.04381000	-2.16581700
O	0.99670100	-2.56501500	-1.07528800
C	3.30582600	-2.18809900	-1.32368700

O	3.78838600	0.42527000	-0.83606500
C	2.40240800	2.08253000	-1.77304100
H	-0.28459100	-0.80249100	-2.14093800
H	0.04825500	0.95309700	-2.15561600
H	1.05749800	-0.15834300	-3.11006600
H	3.28568300	-3.27051900	-1.49161100
H	3.72838800	-1.67263700	-2.19645600
H	3.93170200	-1.92713000	-0.46458000
H	2.39783100	1.94104700	-2.86482600
H	1.44101500	2.53692300	-1.50636600
H	3.22823800	2.75168500	-1.50797400
H	-1.21318700	3.00676800	0.26986400

Ph-5-21345-R5-Nu3

C	-1.20277300	0.37920100	-0.09655300
C	-0.92574200	-0.59022700	-1.23406900
C	-0.05981700	1.38157200	-0.10839600
C	0.33360600	-0.91741900	-1.61966500
C	1.21173400	1.00773100	-0.47959100
C	1.44899900	-0.20159300	-1.14435000
N	2.36474900	1.90100400	-0.19066200
O	2.65680400	2.03509100	0.96766700
O	2.91289900	2.39429500	-1.13519200
N	2.75037300	-0.78219100	-1.27089700
O	3.67419700	-0.25859300	-0.67764800
O	2.86428400	-1.78294900	-1.95054200
N	-2.01086300	-1.35664100	-1.81642400
O	-1.73728600	-2.30195300	-2.52423100
O	-3.14621100	-1.00321200	-1.57302300
N	-2.50152200	1.16818700	-0.45233600
O	-2.44821100	1.80444400	-1.47034200
O	-3.44256200	1.08662800	0.28164300
N	-0.31176700	2.73987300	0.29451900
O	-1.35626000	2.98148000	0.86961900
O	0.51911800	3.58349200	0.03029200
C	-1.34449800	-0.36697100	1.31011200
C	-0.06588800	-1.21171400	1.49904400
C	-2.49643200	-1.29264100	1.29094300
C	-1.54657200	0.58492800	2.50607100
C	1.07583200	-0.64226600	2.07415400
C	0.01464800	-2.51756200	1.00062500
N	-3.35860900	-2.05518500	1.36023000
H	-2.49979100	1.11380300	2.42751800
H	-1.52490100	-0.01232500	3.42698400
H	-0.74479400	1.32827300	2.55476600
C	2.28316700	-1.33960600	2.09755500
H	1.05760300	0.37506200	2.46219100
C	1.21921200	-3.21769800	1.02863600
H	-0.86426600	-2.98741200	0.55469400
C	2.36201300	-2.62525900	1.56552200
H	3.16863300	-0.86190000	2.51858400
H	1.26410000	-4.22729300	0.61831500

H	3.31034300	-3.16442700	1.56936900
H	0.48744000	-1.76174700	-2.28959000

Ph-5-21345-R5-Nu2

C	0.66451800	-0.99011100	0.26116600
C	1.05514500	-0.60232600	1.67507500
C	1.80917200	-0.51871700	-0.62963400
C	1.72311200	0.54613900	1.94092400
C	2.43950300	0.67421400	-0.35432600
C	2.30474400	1.29349100	0.89694600
N	3.33438300	1.30617700	-1.36089100
O	2.79580500	1.79144300	-2.31717100
O	4.50781700	1.29582800	-1.11528900
N	2.79554200	2.61223300	1.17119900
O	3.10530000	3.31526400	0.22901900
O	2.85753200	2.96503700	2.33109700
N	0.51604500	-1.34541300	2.79355200
O	0.84046300	-1.01968100	3.91149300
O	-0.24071200	-2.26035000	2.54071000
N	0.57864800	-2.53258400	0.18437000
O	1.57787500	-3.11412700	0.51566300
O	-0.44757400	-3.01804000	-0.18142200
N	2.19276000	-1.28637600	-1.78891100
O	1.49009600	-2.21609000	-2.12317200
O	3.21427500	-0.97302100	-2.36470800
C	-0.65984400	-0.29440600	-0.16906800
Br	-1.18466500	-0.59106500	-2.00114400
S	-2.13962500	-0.41598300	0.94478500
H	-0.43360400	0.78014000	-0.10525200
O	-2.77521800	-1.71725600	0.84941500
O	-1.69339700	0.12082500	2.22967300
C	-3.23396800	0.79311500	0.22946800
C	-4.28367000	0.36021200	-0.57795800
C	-3.03563800	2.13735700	0.54035500
C	-5.15209400	1.31366600	-1.10434100
H	-4.40914600	-0.70395300	-0.78097500
C	-3.90885700	3.07979200	0.00086700
H	-2.22249000	2.43409000	1.20485400
C	-4.96078800	2.66715400	-0.81896400
H	-5.98144500	0.99888200	-1.73836600
H	-3.77286400	4.13719800	0.22929000
H	-5.64341300	3.40889100	-1.23586900
H	1.81248400	0.89338300	2.96905800

Ph-5-21345-R5-Nu4

C	1.15671000	-0.33802200	-0.19551300
C	1.94351300	-0.88316300	1.02045400
C	1.08096000	1.16585300	-0.22142600
C	1.26639400	-0.53652600	2.33025200
H	2.04861400	-1.96892200	0.90670000
H	2.95047600	-0.45304900	0.97930500

C	-0.25277900	-0.88382400	-0.28138400
C	-0.05797100	1.89925700	-0.17817400
C	0.25789000	-1.51630500	2.87004800
C	1.56850500	0.59778500	2.96995100
C	-1.38723300	-0.11161400	-0.22126200
C	-1.32701400	1.29346900	-0.16955600
H	0.70170200	-2.52111900	2.94987000
H	-0.09761800	-1.21078100	3.86246100
H	-0.62189200	-1.60728000	2.21326900
H	2.31004100	1.29752500	2.57402100
H	1.08288800	0.86396300	3.91166400
N	-2.72178800	-0.76460100	-0.17791100
O	-3.21535900	-0.87118400	0.91089800
O	-3.18973900	-1.11152900	-1.22531900
N	-2.48399300	2.13341000	-0.19315900
O	-3.57589400	1.61677600	-0.34150300
O	-2.32020000	3.33182100	-0.06589800
N	2.32751400	1.89661100	-0.25476000
O	2.32721900	3.07972000	0.01252000
O	3.32758900	1.27672400	-0.56751200
N	1.91823200	-0.78705500	-1.50887300
O	1.40725200	-0.43345300	-2.54110600
O	2.92524700	-1.42498600	-1.40594100
N	-0.35673900	-2.32132200	-0.33227500
O	0.61962600	-2.92859300	-0.72715900
O	-1.38816800	-2.86107100	0.01222000
H	0.00877600	2.98510100	-0.15838200

Ph-5-31245-R1

C	-0.00983300	-0.94151900	-0.04721200
C	-1.22166000	-0.22641400	0.03110300
C	1.21396100	-0.26109300	0.06865200
C	-1.19371800	1.17134000	-0.00840700
C	1.22588300	1.14178600	-0.01257100
C	0.02631900	1.83355800	-0.09907300
C	-2.43889800	2.05362400	-0.02791400
C	-2.54326200	-0.96076500	0.29422000
C	2.49933000	1.96883000	-0.19178500
C	2.50992900	-0.98000200	0.46216600
C	-0.03140400	-2.41062800	-0.50692900
F	0.03300700	-3.30187600	0.46668900
F	-1.14107600	-2.64261900	-1.20708400
F	0.97681200	-2.62146500	-1.35181700
F	2.37258200	-2.28947700	0.60044100
F	2.89057600	-0.52221100	1.65339900
F	3.48815600	-0.75288300	-0.40536400
F	2.21958500	3.26968400	-0.15664000
F	3.42087100	1.74107300	0.73301700
F	3.04013900	1.71169900	-1.38053100
F	-3.41842400	1.50508800	-0.73935200
F	-2.89250500	2.30565800	1.19382700
F	-2.15500400	3.22793300	-0.58982800

F	-2.32115500	-2.11810900	0.91195200
F	-3.29380900	-0.23509400	1.12009400
F	-3.24851200	-1.19383800	-0.80051500
H	0.03893100	2.91563500	-0.20809800

Ph-5-31245-R1-Nu1

C	-0.09177100	-0.57084400	0.28014500
C	-1.26922100	0.43942100	0.15010000
C	1.25920000	0.18774900	0.18727200
C	-1.06568000	1.77141900	-0.15458300
C	1.33010300	1.53359900	-0.12640000
C	0.20255200	2.33117400	-0.34422600
C	-2.19469200	2.77991500	-0.34795100
C	-2.68583600	-0.05721800	0.28998700
C	2.64568000	2.29417700	-0.26742400
C	2.51235700	-0.53461000	0.60801700
C	-0.18147600	-1.08712200	1.77024300
F	0.09164900	-0.03971000	2.56786400
F	-1.39501900	-1.49627700	2.13724300
F	0.67534600	-2.04048200	2.11439600
F	2.43780700	-1.86732700	0.46667400
F	2.83692200	-0.32493300	1.89719500
F	3.59702500	-0.18536700	-0.10258600
F	2.44930500	3.61435100	-0.36466500
F	3.46063600	2.13015500	0.77778300
F	3.31360200	1.94909000	-1.37516500
F	-2.73285500	2.70302000	-1.56971400
F	-3.18021400	2.65867800	0.54311300
F	-1.75292000	4.03814400	-0.22431400
F	-3.26039400	0.21351000	1.47136600
F	-3.50487500	0.47142600	-0.64271600
F	-2.81240100	-1.38381100	0.11603100
C	-0.22741300	-1.69739900	-0.90824400
C	-1.02949200	-2.96573200	-0.52941700
C	1.07359700	-2.32840000	-1.49773400
C	-0.81980400	-0.97742700	-2.14213800
O	-0.89834500	-3.49093900	0.54420600
C	-1.90155500	-3.58643300	-1.59813600
O	1.23894300	-3.52437500	-1.45639400
C	2.01449300	-1.48355500	-2.33350700
H	-1.87637600	-0.72374800	-2.00991600
H	-0.27417300	-0.04158200	-2.32408400
H	-0.73670700	-1.60453600	-3.04071500
H	-2.69649800	-2.89626200	-1.90792500
H	-1.29420900	-3.82822900	-2.48171900
H	-2.34158200	-4.50440000	-1.19316300
H	1.72137400	-1.61491000	-3.38608300
H	1.99954300	-0.41585600	-2.09841800
H	3.02694900	-1.88532500	-2.21029000
H	0.30812400	3.38086300	-0.57877300

Ph-5-31245-R1-Nu3

C	0.26706400	0.96220600	-0.20418700
C	-0.91283900	0.16883000	-0.81435300
C	1.53100100	0.07524200	-0.20896300
C	-0.85794600	-1.20474500	-0.97688300
C	1.44449400	-1.30768800	-0.31124900
C	0.26995000	-1.96823500	-0.65983400
C	-2.01299600	-2.05278900	-1.50064100
C	-2.23555800	0.85786800	-1.06736700
C	2.59865200	-2.24596500	0.02439600
C	2.91265600	0.67638500	-0.14499500
C	0.54960700	2.23319200	-1.10486200
F	1.50522500	1.97737900	-2.00241800
F	-0.50299900	2.61893700	-1.82551200
F	0.92659100	3.30965800	-0.41037400
F	2.93337200	1.99450600	0.08318100
F	3.61690500	0.49914600	-1.27640200
F	3.67320500	0.14307900	0.83583200
F	2.31932100	-3.51459700	-0.29506000
F	3.74541900	-1.96467700	-0.59367000
F	2.84056300	-2.24669200	1.34441200
F	-2.87928300	-2.38348900	-0.53891000
F	-2.69530600	-1.47565400	-2.49127300
F	-1.56863900	-3.21403000	-2.00211700
F	-2.48589600	1.14303700	-2.35516200
F	-3.27979200	0.11727300	-0.65560200
F	-2.36010400	2.01237000	-0.39738400
C	-0.08032300	1.43077300	1.36041600
C	-0.93227400	0.32495500	2.00055600
C	-0.79806500	2.71429500	1.47500500
C	1.17789900	1.65609600	2.22962900
C	-0.32274700	-0.87355500	2.39788900
C	-2.30912000	0.46892200	2.19962500
N	-1.30134200	3.73497600	1.66800300
H	1.72455200	2.54787300	1.90889000
H	0.85806400	1.78901500	3.27163800
H	1.84824400	0.79034100	2.18704900
C	-1.07816800	-1.90849200	2.94416000
H	0.74947200	-1.01563800	2.26678900
C	-3.06451800	-0.56469700	2.75375100
H	-2.80518900	1.39511900	1.90847200
C	-2.45409600	-1.76132300	3.12110800
H	-0.58338200	-2.83694600	3.23396500
H	-4.13828300	-0.42960500	2.89261200
H	-3.04441000	-2.57305500	3.54902600
H	0.24705200	-3.04579700	-0.74953600

Ph-5-31245-R1-Nu2

C	0.41700800	0.00884200	0.67294900
C	0.85116800	1.43404700	0.29396800
C	1.44382100	-1.02781700	0.18840400
C	2.02685300	1.67533800	-0.40495300

C	2.60327000	-0.66098200	-0.45690200
C	2.91202500	0.66756200	-0.78214500
C	2.45182600	3.06610700	-0.86437300
C	-0.00643800	2.59828000	0.72345900
C	3.65260000	-1.66876100	-0.91023000
C	1.18132500	-2.47017600	0.53162900
C	0.39432000	-0.09487000	2.24078500
F	1.66061900	-0.11249800	2.66962400
F	-0.18257700	0.93170800	2.85324800
F	-0.18294000	-1.20741200	2.69281400
F	-0.11487000	-2.72259800	0.72604400
F	1.80602800	-2.86805500	1.65642900
F	1.57148600	-3.31629200	-0.43139700
F	4.81440800	-1.06782500	-1.19525700
F	3.93647600	-2.58205800	0.02135300
F	3.29123000	-2.31359300	-2.02234200
F	1.76946500	3.46543900	-1.94150700
F	2.31232900	4.00274900	0.07611100
F	3.74293300	3.09481400	-1.21538200
F	0.47560700	3.27222100	1.77974600
F	-0.15874200	3.50913200	-0.25363000
F	-1.26315200	2.25144500	1.06581400
C	-1.02882500	-0.22214700	-0.02287000
Br	-0.91166600	-1.14880100	-1.72274900
S	-2.53792900	-0.82435100	0.87604300
H	-1.38900600	0.75939400	-0.32691000
O	-2.64943200	-2.27532400	0.90777700
O	-2.60627200	-0.05187200	2.11260400
C	-3.83245300	-0.21160600	-0.19298400
C	-4.48151500	-1.09695000	-1.04963100
C	-4.18565000	1.13430600	-0.10605300
C	-5.50904100	-0.61096600	-1.85625800
H	-4.17917000	-2.14449100	-1.07303300
C	-5.21090700	1.60746100	-0.92284100
H	-3.66697400	1.79177900	0.59379600
C	-5.86828800	0.73665900	-1.79431300
H	-6.03125200	-1.28689000	-2.53428200
H	-5.50220400	2.65713100	-0.87298700
H	-6.67206700	1.11192800	-2.42932500
H	3.83019000	0.90579100	-1.30163000

Ph-5-31245-R1-Nu4

C	0.00759000	-1.03462800	-0.12412700
C	0.02485600	-2.16705400	0.96937000
C	1.28164100	-0.15385300	-0.03563400
C	0.06544000	-1.63370900	2.38270800
H	-0.85359400	-2.80315300	0.84148500
H	0.89053100	-2.80697400	0.79582200
C	-1.27482100	-0.16595700	-0.05430900
C	1.20664400	1.22642000	-0.08361600
C	-1.24065900	-1.20749800	2.99886400
C	1.21877500	-1.54864900	3.05562200

C	-1.20954600	1.21618900	-0.09885600
C	-0.00552900	1.92774300	-0.12470000
H	-1.98480300	-2.01603800	2.92745600
H	-1.11228500	-0.93549400	4.05520500
H	-1.66588000	-0.34326300	2.46550500
H	2.16335500	-1.86298200	2.60747300
H	1.24743700	-1.15886800	4.07652800
C	2.42353100	2.14830200	-0.03292400
C	2.64616300	-0.79333700	-0.08294000
C	-2.43519900	2.12270000	-0.04194500
C	-2.62791100	-0.82552300	-0.11310100
C	0.01722200	-1.72671600	-1.50676600
F	-0.00213400	-0.83106000	-2.49508400
F	1.10184200	-2.48937300	-1.69429800
F	-1.03939400	-2.52956200	-1.68673800
F	-2.66177300	-2.10617300	0.28643800
F	-3.15817900	-0.83267600	-1.35376700
F	-3.53751800	-0.21230500	0.66761500
F	-2.13575200	3.38373600	-0.38092000
F	-3.41459000	1.75769300	-0.87316700
F	-2.94879700	2.19297400	1.19218700
F	2.93349200	2.24618300	1.19862500
F	3.41033300	1.78320600	-0.85525500
F	2.10842100	3.39996700	-0.39472800
F	3.19245500	-0.76988200	-1.31696300
F	3.53035500	-0.17569000	0.72092100
F	2.70310300	-2.08232500	0.28928000
H	-0.01071300	3.00779200	-0.15266100

Ph-5-31245-R2

C	0.00012500	0.99107600	0.00000000
C	0.00026100	0.28818900	-1.21547700
C	0.00026100	0.28818900	1.21547700
C	0.00001000	-1.11842700	-1.20769700
C	0.00001000	-1.11842700	1.20769700
C	0.00014000	-1.81758900	0.00000000
C	-0.00011500	-1.84272100	-2.45292400
N	-0.00002800	-2.42463600	-3.44837500
C	-0.00014100	1.00747100	-2.46133300
N	-0.00013000	1.58117200	-3.46159700
C	-0.00011500	-1.84272100	2.45292400
N	-0.00002800	-2.42463600	3.44837500
C	-0.00014100	1.00747100	2.46133300
N	-0.00013000	1.58117200	3.46159700
C	0.00006200	2.42995600	0.00000000
N	0.00000800	3.58292900	0.00000000
H	0.00000200	-2.90682800	0.00000000

Ph-5-31245-R2-Nu1

C	-0.12008200	-0.32726400	-0.68998100
C	1.40842100	-0.41041500	-0.70258400

C	-0.46235800	1.16015000	-0.70615900
C	2.13156600	0.49715000	0.06946000
C	0.30559900	2.02981300	0.05630800
C	1.55871200	1.66595500	0.58827000
C	3.53778400	0.26236400	0.29460500
N	4.65958600	0.07506400	0.49566600
C	2.06466400	-1.44716200	-1.41578000
N	2.58971600	-2.30801300	-1.99049900
C	-0.14427800	3.38769500	0.24593000
N	-0.48982600	4.47660900	0.41473300
C	-1.53537600	1.66978100	-1.49157000
N	-2.37320500	2.13125000	-2.14578000
C	-0.61841100	-0.96833000	-1.92671600
N	-0.94141200	-1.49871900	-2.89784400
C	-0.74811700	-1.13391800	0.54209100
C	-2.28726100	-1.11160400	0.46035600
C	-0.40348300	-0.42909900	1.86988000
C	-0.27070500	-2.59200800	0.51801600
O	-2.86671300	-0.55353100	-0.43554100
C	-3.04037200	-1.88622500	1.51738000
O	-1.12883200	0.45795100	2.25624100
C	0.75718900	-0.92486500	2.69388000
H	-0.61353400	-3.09556800	-0.39781700
H	0.82439900	-2.65353900	0.55016000
H	-0.66219000	-3.14773700	1.38054200
H	-3.13807200	-2.92709100	1.17231700
H	-2.52991400	-1.89333700	2.48824400
H	-4.04536700	-1.46128900	1.61845700
H	0.43750400	-1.83887800	3.22013200
H	1.62713700	-1.19000000	2.08162400
H	1.01966300	-0.16317700	3.43643300
H	2.12977000	2.34982000	1.21006300

Ph-5-31245-R2-Nu3

C	0.90660000	0.80544000	0.08032600
C	1.18807900	-0.36951700	1.00917800
C	-0.29531900	1.55862400	0.63565500
C	0.13424200	-1.00505800	1.65795000
C	-1.30453600	0.85982600	1.29622500
C	-1.15810500	-0.46236900	1.73416200
C	0.38557500	-2.25591500	2.33405400
N	0.56535800	-3.26392900	2.86782000
C	2.51864600	-0.85049600	1.15696900
N	3.60284700	-1.23959100	1.29105800
C	-2.54185000	1.54285600	1.59193500
N	-3.54170200	2.07336000	1.82062800
C	-0.40627000	2.95780200	0.41718100
N	-0.48663300	4.09917100	0.22365000
C	2.08270400	1.70341700	0.06538000
N	2.99957600	2.40191300	0.04194300
C	0.66057800	0.33692700	-1.45474400
C	-0.52160700	-0.62836900	-1.50974500

C	1.88606500	-0.35651800	-1.89390900
C	0.47418900	1.56187600	-2.36602600
C	-1.82326200	-0.14205500	-1.67867200
C	-0.33322500	-2.00316500	-1.32168900
N	2.86012500	-0.86684000	-2.24302400
H	1.38656200	2.17216100	-2.38464300
H	0.24642900	1.23108600	-3.38756400
H	-0.35089700	2.19092300	-2.01193400
C	-2.91461200	-1.00723100	-1.61999800
H	-2.00030100	0.92199300	-1.83574300
C	-1.42384900	-2.86841900	-1.26533300
H	0.67641900	-2.40238900	-1.20431700
C	-2.71937200	-2.37100900	-1.40420300
H	-3.92306800	-0.60964200	-1.74255600
H	-1.25783500	-3.93531200	-1.10970100
H	-3.57467800	-3.04658200	-1.35571500
H	-1.96764300	-0.99070600	2.22947000

Ph-5-31245-R2-Nu2

C	1.03346500	-0.03252800	0.04479000
C	1.85472700	1.21359500	-0.25803700
C	2.06794000	-1.13905600	-0.13020500
C	3.00851100	1.37312300	0.50755500
C	3.22525200	-0.99174600	0.64550500
C	3.59846300	0.26519600	1.15131300
C	3.69535700	2.63853600	0.51923200
N	4.25299300	3.65018900	0.53741800
C	1.51074700	2.11589100	-1.29862500
N	1.25098400	2.83123800	-2.17320700
C	4.12339300	-2.10542400	0.80966000
N	4.84826400	-2.99411400	0.95003000
C	1.95616500	-2.19943200	-1.06660500
N	1.92108800	-3.06447100	-1.83865100
C	0.58719000	-0.02513500	1.47373900
N	0.24490900	-0.05936600	2.57442800
C	-0.21845100	-0.17201900	-0.83749200
Br	-1.11573400	-1.86385200	-0.58720900
S	-1.43871900	1.24696600	-0.60499900
H	0.04234700	-0.09201500	-1.90113000
O	-0.82731700	2.20697000	0.30932000
O	-1.80179600	1.62374500	-1.96522000
C	-2.89402500	0.61038200	0.19378300
C	-2.95545100	0.60737500	1.58564400
C	-3.95826700	0.19560500	-0.60490300
C	-4.12171500	0.14504200	2.19321500
H	-2.10713500	0.95546700	2.17595000
C	-5.11741200	-0.25843000	0.01836700
H	-3.87062900	0.23254600	-1.69123100
C	-5.19480700	-0.28741300	1.41260400
H	-4.19295800	0.12885500	3.28107800
H	-5.96316100	-0.58839500	-0.58556300
H	-6.10475000	-0.64595600	1.89582700

H	4.46520300	0.38142800	1.79719500

Ph-5-31245-R2-Nu4

C	-0.06284500	-0.50639300	0.76532800
C	-0.33910000	-2.00419300	0.42784900
C	-1.16519300	0.40758800	0.22974800
C	-0.56731000	-2.30659800	-1.03685400
H	0.51379100	-2.59139400	0.80677400
H	-1.22380500	-2.31361100	1.00251300
C	1.28743800	-0.00763000	0.24827500
C	-0.89301500	1.69979200	-0.19849400
C	0.59584400	-2.18400600	-1.98529600
C	-1.77043100	-2.71332400	-1.45602800
C	1.46376500	1.30331100	-0.17750900
C	0.40719000	2.20981300	-0.35357700
H	1.48125700	-2.70727400	-1.59362400
H	0.34102300	-2.60210300	-2.96789700
H	0.88610800	-1.13128600	-2.12427100
H	-2.61598300	-2.79298200	-0.76946200
H	-1.94326000	-2.96759800	-2.50474700
C	-1.99985700	2.55821900	-0.55533700
N	-2.87293500	3.25698300	-0.84278800
C	-2.50635600	-0.04874200	0.34522300
N	-3.59322500	-0.43419200	0.47546900
C	2.79719400	1.74594200	-0.51605100
N	3.85707900	2.11345700	-0.78967000
C	2.39241200	-0.89358400	0.34429700
N	3.27271100	-1.64538900	0.43069100
C	-0.04808500	-0.41416400	2.26073800
N	-0.03077700	-0.31859900	3.41148200
H	0.58099200	3.22424300	-0.69945100

Ph-5-31245-R3

C	0.01380800	0.77162700	0.04615500
C	1.26350300	0.14554000	0.00285600
C	-1.16342900	0.01584900	0.04443200
C	-0.05041300	2.27097300	0.15122600
C	2.50612300	1.00217100	0.01599900
C	1.33212400	-1.25047400	-0.07665200
C	-2.49007300	0.72984100	0.12398900
C	-1.08480000	-1.38187100	0.01081700
O	-0.92631400	2.78291800	-0.70184700
O	0.61409400	2.91158800	0.91944000
O	3.23511300	0.77289400	1.09707000
O	2.77239000	1.79627600	-0.84260400
C	0.15951600	-2.00209600	-0.06375000
C	2.62228500	-2.00843400	-0.19569900
O	-3.23374700	0.48538700	-0.94346000
O	-2.80516800	1.44249900	1.03710500
C	-2.28749200	-2.27725700	0.08059000
C	-1.17588800	4.18211000	-0.58176400

C	4.46018200	1.49629200	1.17951100
O	3.58044800	-1.28837700	-0.76680200
O	2.75237100	-3.15262600	0.14843900
C	-4.53949500	1.05577000	-0.93152900
O	-3.29008200	-1.72268200	0.75221000
O	-2.31571200	-3.38707400	-0.37947600
H	-1.88443500	4.43462800	-1.37490300
H	-1.60808900	4.39644400	0.40458600
H	-0.24251400	4.74515900	-0.70213100
H	4.93169200	1.19504800	2.11861000
H	4.26292300	2.57565500	1.17490200
H	5.10265600	1.24344300	0.32548300
C	4.85242400	-1.91916200	-0.89935100
H	-5.01139100	0.76197800	-1.87278200
H	-4.47617700	2.14876400	-0.85579300
H	-5.10878300	0.66829300	-0.07599100
C	-4.48594300	-2.49273800	0.85357200
H	5.25655900	-2.16331700	0.09160900
H	4.75742000	-2.84023500	-1.48751100
H	5.49692000	-1.20012300	-1.41184900
H	-4.27593300	-3.45328500	1.33998100
H	-4.90108100	-2.67714600	-0.14578500
H	-5.17946200	-1.90007100	1.45600600
H	0.21795100	-3.09049900	-0.10744700

Ph-5-31245-R3-Nu1

C	-0.04147500	-0.69841600	0.07944800
C	1.23953800	0.10927800	0.24081600
C	-1.21903000	0.26041600	0.32916300
C	-0.09742500	-1.81407600	1.13954400
C	2.41921200	-0.56582200	0.73092000
C	1.27475400	1.45828600	-0.12542000
C	-2.49359200	-0.27840500	0.84280300
C	-1.10705100	1.59720000	0.03606600
O	0.14794000	-1.34616700	2.36144500
O	-0.36378400	-2.96549000	0.91676600
O	3.42376600	0.27584500	1.08337200
O	2.55373700	-1.77257300	0.84995300
C	0.12168100	2.20770600	-0.31764800
C	2.57093000	2.14177200	-0.44854000
O	-3.56355600	0.39024600	0.37509500
O	-2.61914700	-1.20370600	1.61359000
C	-2.25146100	2.55574200	0.20549300
C	0.03663300	-2.29633200	3.40411100
C	4.66317800	-0.33122400	1.38907100
O	2.78039400	3.23010500	0.29167500
O	3.30797100	1.80770400	-1.34382200
C	-4.82138100	-0.04025600	0.85923300
O	-2.71200700	2.96761800	-0.97894400
O	-2.65710300	2.98270500	1.25429900
H	0.25420000	-1.76447300	4.33568000
H	-0.98124500	-2.70888200	3.42698300

H	0.75568000	-3.11406500	3.25568800
H	5.36200400	0.48246800	1.61087100
H	5.03155500	-0.92279400	0.53863900
H	4.57042500	-0.99491500	2.25935200
C	3.96696400	3.95589300	0.00287900
H	-5.57224800	0.60070800	0.38580600
H	-4.87588200	0.05579100	1.95232100
H	-4.99325200	-1.09075200	0.58800000
C	-3.78219300	3.90053800	-0.93435900
H	4.84972100	3.32003000	0.15471300
H	3.96180800	4.30906100	-1.03685800
H	3.98620300	4.80413600	0.69351400
H	-3.48175900	4.81056000	-0.39832400
H	-4.65011300	3.46076400	-0.42421800
H	-4.03208000	4.13515000	-1.97334100
C	-0.13914300	-1.41845700	-1.37227200
C	-1.39898000	-2.31225600	-1.45998900
C	1.16868700	-2.18926900	-1.66708700
C	-0.34908300	-0.34454600	-2.45798700
O	-2.47934800	-1.79079200	-1.27871400
C	-1.35112600	-3.75737600	-1.89357300
O	1.28494000	-3.37107500	-1.44793800
C	2.30697600	-1.45151600	-2.34362800
H	-1.29634000	0.17590000	-2.27400800
H	0.46384300	0.39103500	-2.45342000
H	-0.39503700	-0.81040500	-3.45506900
H	-2.38510300	-4.08741000	-2.04816100
H	-0.76833500	-3.87360000	-2.81725500
H	-0.85272900	-4.34952300	-1.11996000
H	2.06292900	-1.34470500	-3.41266800
H	2.48002900	-0.44395400	-1.94607700
H	3.20924900	-2.06673200	-2.24900200
H	0.16636700	3.25797000	-0.60168600

Ph-5-31245-R3-Nu3

C	0.14043100	-0.62632300	0.10831800
C	1.63365900	-0.29081400	0.05183600
C	-0.60648100	0.64134100	0.52073300
C	-0.08729600	-1.72496000	1.16513600
C	2.61053200	-1.31090900	0.41993600
C	2.03704500	0.97685100	-0.34710800
C	-1.80760400	0.51482900	1.34025800
C	-0.13852100	1.88127800	0.10148100
O	0.31284000	-1.31219900	2.36993900
O	-0.54352900	-2.81676800	0.96410200
O	3.87954200	-0.84153400	0.47206600
O	2.37011000	-2.46752600	0.70159000
C	1.15414800	2.06262400	-0.40784100
C	3.42531700	1.24913800	-0.85286000
O	-2.30048300	1.70403400	1.76111400
O	-2.34017900	-0.52459700	1.67455200
C	-0.98901200	3.11882300	0.11810300

C	0.14552700	-2.25326800	3.41392100
C	4.88556600	-1.80079900	0.73724300
O	4.09603500	2.10334100	-0.08107600
O	3.84983500	0.83016700	-1.89933500
C	-3.52907700	1.64992800	2.46106100
O	-2.06765200	2.97788000	-0.66375100
O	-0.70610800	4.15432400	0.66050900
H	0.51412700	-1.77557100	4.32719900
H	-0.91605800	-2.51616300	3.52142600
H	0.72119700	-3.16423900	3.19966000
H	5.84083500	-1.26714200	0.69782300
H	4.86816400	-2.60357100	-0.01229200
H	4.74414500	-2.24849700	1.73034000
C	5.40190500	2.44218600	-0.52615300
H	-3.78300000	2.68240600	2.72290300
H	-3.43360900	1.04173800	3.37030600
H	-4.31625000	1.21200400	1.83057700
C	-2.96775800	4.07445600	-0.67663100
H	6.03183300	1.54372400	-0.57903100
H	5.36427700	2.90507100	-1.52115300
H	5.80573900	3.14641000	0.20715900
H	-2.47263100	4.98275700	-1.04476000
H	-3.34667600	4.26370700	0.33757500
H	-3.78954300	3.79301600	-1.34228800
C	-0.39710200	-1.17140900	-1.30967400
C	-1.91994800	-1.36366800	-1.32881300
C	0.26998500	-2.44903200	-1.63769100
C	0.01374800	-0.22380300	-2.46090300
C	-2.75809300	-0.25361700	-1.50572900
C	-2.51065700	-2.62442600	-1.18999800
N	0.80413000	-3.40193400	-2.01094900
H	1.10583600	-0.16467600	-2.54306700
H	-0.39734400	-0.60688300	-3.40504200
H	-0.37588400	0.78510000	-2.28927300
C	-4.14553500	-0.39966300	-1.50856200
H	-2.33748900	0.74507200	-1.61455600
C	-3.89658400	-2.77269300	-1.20845900
H	-1.87506400	-3.49759000	-1.04741900
C	-4.72283200	-1.65934000	-1.35895100
H	-4.77660800	0.48248400	-1.63435600
H	-4.33179300	-3.76749100	-1.09825100
H	-5.80809600	-1.77464900	-1.36665300
H	1.48351300	3.03917800	-0.75976800

Ph-5-31245-R3-Nu2

C	0.62580300	-0.38874400	0.73163900
C	1.87575200	0.25778700	0.16407100
C	-0.30475000	0.73596900	1.21085000
C	0.94451100	-1.36578400	1.86209800
C	3.17618000	-0.37210300	0.34128500
C	1.69058500	1.36162600	-0.66249200
C	-1.16209800	0.55598800	2.39565300

C	-0.40352000	1.85298000	0.40926400
O	1.68803100	-0.80101100	2.80213600
O	0.54966800	-2.50144800	1.89819000
O	4.19901700	0.45172900	0.01012700
O	3.38912900	-1.49630900	0.74873000
C	0.49873600	2.09637200	-0.65209100
C	2.73106400	1.77573400	-1.66497800
O	-2.33429600	1.21610100	2.26961300
O	-0.91061400	-0.09069700	3.38484900
C	-1.33781600	2.98838800	0.71996400
C	1.95710300	-1.61126300	3.93037200
C	5.48275800	-0.14118300	-0.03126900
O	3.13574700	3.03443000	-1.48369000
O	3.10577200	1.09181800	-2.58177200
C	-3.22583400	1.10723100	3.36399300
O	-2.33290200	3.06797300	-0.17014300
O	-1.18112600	3.78981400	1.60198400
H	2.58951200	-1.01714000	4.59724100
H	1.01539000	-1.87467800	4.43063800
H	2.47815900	-2.52919300	3.62648200
H	6.17610000	0.64055900	-0.35881600
H	5.50166200	-0.98305100	-0.73754300
H	5.77394300	-0.51142300	0.96093300
C	4.09228300	3.51384700	-2.41812000
H	-4.10220600	1.71273400	3.11083700
H	-2.76084300	1.48060900	4.28626700
H	-3.51697500	0.05862100	3.51200100
C	-3.24069500	4.14564500	0.01985000
H	4.99935500	2.89514300	-2.38738600
H	3.68425100	3.48883600	-3.43727400
H	4.32309400	4.54222100	-2.12500600
H	-2.71239900	5.10711800	-0.01923000
H	-3.74050600	4.05573400	0.99372800
H	-3.97280100	4.08186000	-0.79078400
C	-0.10468000	-1.12719600	-0.43597800
Br	1.00313400	-2.33287300	-1.46854900
S	-1.70553400	-1.98251500	-0.03793200
H	-0.39511000	-0.34560200	-1.14906500
O	-1.62236300	-3.41089000	-0.31774400
O	-2.18729700	-1.53877800	1.26761200
C	-2.77083400	-1.23994200	-1.26345100
C	-3.06831900	-1.94271400	-2.42689100
C	-3.24553700	0.04991600	-1.02431000
C	-3.87443800	-1.32917400	-3.38640700
H	-2.67402500	-2.95023800	-2.56547400
C	-4.03947700	0.65238000	-1.99749400
H	-2.98754900	0.57216000	-0.09868400
C	-4.35463700	-0.03647600	-3.17165500
H	-4.12577400	-1.86171000	-4.30445200
H	-4.41262000	1.66434500	-1.83625300
H	-4.97955100	0.44070400	-3.92804600
H	0.37477600	2.96565000	-1.29651500

Ph-5-31245-R3-Nu4

C	0.07013500	-0.84959300	0.30303200
C	-0.01331500	-1.05156500	1.85347300
C	-1.08192200	0.03804000	-0.16924000
C	-1.37606100	-1.37804000	2.42478900
H	0.72296700	-1.82423000	2.12131800
H	0.31971100	-0.09539100	2.28287100
C	1.37668200	-0.13578800	-0.04325800
C	0.04580000	-2.21223900	-0.39680700
C	-2.35276300	-0.61692900	-0.44058900
C	-0.95704800	1.40346500	0.00296500
C	-1.77303200	-2.82273500	2.56324900
C	-2.21278000	-0.39087900	2.77310000
C	2.54510500	-0.89814600	-0.44539600
C	1.43728600	1.23676900	0.16148100
O	0.11556300	-2.07892300	-1.72588600
O	0.01013000	-3.28340500	0.14847100
O	-3.39952500	0.23030100	-0.56607700
O	-2.51936300	-1.81967400	-0.52903400
C	0.27838400	2.01077700	0.30274500
C	-2.10717800	2.35988800	-0.11298700
H	-1.88242600	-3.27355200	1.56767600
H	-0.99278700	-3.39559500	3.08835200
H	-2.71868000	-2.92097400	3.11460000
H	-1.94016500	0.66052700	2.64247300
H	-3.20600800	-0.60814300	3.17685600
O	3.50373200	-0.13652700	-1.03247700
O	2.70028300	-2.09996700	-0.32726900
C	2.73436000	1.98188400	0.27052300
C	0.15072900	-3.29018000	-2.45496400
C	-4.67077400	-0.37261000	-0.71338500
O	-2.36957100	2.69632200	-1.37565700
O	-2.67130400	2.84959800	0.83228900
C	4.74670100	-0.77169500	-1.25457500
O	3.54964400	1.44539600	1.18642700
O	2.99890300	2.99831300	-0.31985700
H	0.20854900	-3.01691700	-3.51355300
H	1.02982700	-3.88315500	-2.16598700
H	-0.75586900	-3.88098200	-2.26390900
H	-5.39290700	0.44457400	-0.81157800
H	-4.91278100	-0.98511600	0.16680000
H	-4.70068800	-1.01450200	-1.60387900
C	-3.47754600	3.56273400	-1.56198500
H	5.39375900	-0.03088200	-1.73645700
H	4.62860500	-1.64894000	-1.90434500
H	5.19360600	-1.10042900	-0.30438900
C	4.82530600	2.05308100	1.30201900
H	-4.39389000	3.09354700	-1.17715700
H	-3.32175400	4.51495000	-1.03771000
H	-3.56193500	3.73176000	-2.63966400
H	4.73132900	3.10983500	1.58557900
H	5.36335500	1.99296400	0.34507000
H	5.36501900	1.49828200	2.07565200

H	0.34681800	3.08346000	0.47880600

Ph-5-31245-R4

C	-0.00005200	1.11729200	-0.00019600
C	-1.20802400	0.42445100	0.00060800
C	1.20784300	0.42465100	0.00063700
C	-1.19365100	-0.96793000	-0.00002600
C	1.19371500	-0.96785500	0.00001500
C	0.00014500	-1.67592700	0.00083300
F	2.35092900	1.09045900	-0.00038300
F	2.35434300	-1.60727000	-0.00035300
F	-2.35407100	-1.60774000	-0.00034600
F	-2.35101800	1.09050600	-0.00036600
F	-0.00016300	2.43815300	0.00018600
H	-0.00002700	-2.76505400	0.00013600

Ph-5-31245-R4-Nu1

C	0.21207700	-0.06315700	-0.43074600
C	1.02173000	-1.23702500	-0.06704700
C	1.04311400	1.12747800	-0.20003700
C	2.37671600	-1.21543300	0.15605200
C	2.40123000	1.11853900	-0.00712300
C	3.14710400	-0.04894400	0.18427600
F	0.43160600	2.33170700	-0.40528700
F	3.03262500	2.30649500	0.01656200
F	2.97725400	-2.39267800	0.41466200
F	0.37538200	-2.42405200	0.09962000
F	-0.16980000	-0.04893200	-1.83639600
C	-1.18446100	-0.05865200	0.30770800
C	-2.15262500	-1.14466100	-0.22101600
C	-1.92798900	1.26523800	0.02873900
C	-0.95401900	-0.28142900	1.80970900
O	-1.94625700	-1.85025600	-1.17054500
C	-3.43506200	-1.30033300	0.58059300
O	-2.58231500	1.38924900	-0.97994200
C	-1.90753400	2.34806700	1.08246000
H	-0.61547900	-1.31218900	1.98144600
H	-0.16826200	0.39323700	2.17365200
H	-1.86438200	-0.10818700	2.39881900
H	-3.22633300	-1.92136300	1.46485000
H	-3.83440400	-0.33939400	0.93235800
H	-4.17837500	-1.81430100	-0.03942100
H	-2.64815800	2.09226400	1.85659100
H	-0.92865900	2.44079900	1.56458700
H	-2.19809100	3.29701300	0.61768300
H	4.22542900	-0.05081000	0.30947500

Ph-5-31245-R4-Nu3

C	-0.70888400	0.82548500	-0.31786000
C	-0.84229700	0.62999900	1.12375800

C	-1.48744800	-0.20967200	-0.98720500
C	-1.78857000	-0.16322700	1.72370400
C	-2.43493800	-0.99170100	-0.36697100
C	-2.64949400	-1.00190700	1.01100900
F	-1.33625800	-0.36516800	-2.33361500
F	-3.16368200	-1.80913500	-1.14677400
F	-1.84810000	-0.15506600	3.06645900
F	0.02481500	1.30978400	1.92038400
F	-1.23259900	2.18211200	-0.71482000
C	0.79613800	1.00229800	-0.77729400
C	1.62269200	-0.19929200	-0.32207000
C	1.31694000	2.24769800	-0.17469200
C	0.93370300	1.24196300	-2.30027100
C	1.41475100	-1.43831600	-0.94482700
C	2.55227300	-0.11669800	0.71742900
N	1.77776700	3.23443000	0.20696400
H	0.23679600	2.03243300	-2.60200400
H	1.96153200	1.55738400	-2.52522300
H	0.70969100	0.33492100	-2.86639400
C	2.12915300	-2.56344200	-0.54097200
H	0.67901200	-1.52403400	-1.74632700
C	3.26913100	-1.24483200	1.12152500
H	2.71566100	0.83616600	1.22256900
C	3.06220900	-2.47059500	0.49354200
H	1.95365400	-3.51959400	-1.03658200
H	3.99262200	-1.15974000	1.93394800
H	3.62240400	-3.35219900	0.80904300
H	-3.42546600	-1.59565500	1.48470100

Ph-5-31245-R4-Nu2

C	-1.52537300	-0.22375200	-0.40472600
C	-2.13556400	1.10467700	-0.34489900
C	-2.29566200	-1.11897600	0.46305600
C	-3.37875600	1.36031600	0.18173300
C	-3.52829400	-0.82974400	0.99492400
C	-4.13514700	0.42306400	0.88917500
F	-1.74626500	-2.33823300	0.70810400
F	-4.13996500	-1.79629900	1.70012900
F	-3.87235600	2.59839700	0.01408900
F	-1.53507100	2.07455400	-1.07709900
F	-1.53240500	-0.66591400	-1.80061400
C	-0.00333700	-0.32867100	-0.01699500
Br	0.90993400	-1.81794000	-0.87555100
S	1.01637200	1.18067500	-0.25888700
H	0.07089400	-0.50888900	1.06165600
O	1.14240500	1.47596800	-1.68041200
O	0.51216000	2.17325200	0.68763500
C	2.62887300	0.69690400	0.34018800
C	3.62877000	0.38437400	-0.57744000
C	2.85654800	0.69343000	1.71502800
C	4.89086500	0.04009800	-0.09796700
H	3.40744200	0.41402000	-1.64479500

C	4.12089700	0.33806400	2.18191800
H	2.05880500	0.97760500	2.40311300
C	5.13259000	0.01169800	1.27702300
H	5.68822300	-0.20750000	-0.79955400
H	4.31882400	0.32531500	3.25424600
H	6.12163600	-0.26227700	1.64708800
H	-5.11590600	0.64364700	1.29937100

Ph-5-31245-R4-Nu4

C	-0.23284500	0.01761100	0.85699800
C	-1.76927400	-0.05927600	0.83649500
C	0.31978400	1.21986500	0.24431200
C	-2.38152800	-0.08282900	-0.54256300
H	-2.05051900	-0.96312100	1.39743000
H	-2.13160600	0.81281600	1.39544200
C	0.46839200	-1.14294500	0.32518300
C	1.53947900	1.26668800	-0.38330200
C	-2.30947000	-1.37725600	-1.30659400
C	-2.95989000	1.00866100	-1.05461900
C	1.68673300	-1.08120400	-0.30548200
C	2.29392600	0.12349400	-0.67600100
H	-2.73383100	-2.20150900	-0.71246200
H	-2.85097500	-1.30870000	-2.25954800
H	-1.26290500	-1.64664600	-1.51342600
H	-2.98942300	1.94510400	-0.49264500
H	-3.40369100	1.00090700	-2.05370900
F	-0.09504800	-2.36133600	0.56284100
F	2.29345000	-2.24189800	-0.62419100
F	2.00351800	2.46921100	-0.77772200
F	-0.38441000	2.37269900	0.41336400
F	-0.01582700	0.07630300	2.36009000
H	3.27595500	0.16948800	-1.13708700

Ph-5-31245-R5

C	-1.20711900	-0.22846900	0.01076500
C	-0.00013000	-0.91806600	-0.00005000
C	-1.19135500	1.16152300	-0.00458700
C	1.20704100	-0.22883900	-0.01072500
C	0.00026000	1.86838800	0.00002300
C	1.19168500	1.16119600	0.00458800
N	2.45075500	1.93641400	0.08314800
O	2.41833100	3.05559800	-0.34950800
O	3.38828200	1.38592900	0.59793400
N	2.48069300	-0.99187600	-0.03478900
O	3.14847100	-0.86213400	-1.01933300
O	2.70010300	-1.66894800	0.92971700
N	-0.00039300	-2.39950000	-0.00008800
O	0.78791600	-2.92971500	-0.73385900
O	-0.78892000	-2.92943700	0.73363600
N	-2.48087000	-0.99136400	0.03479800
O	-2.70059900	-1.66783600	-0.93008400

O	-3.14832600	-0.86222500	1.01961000
N	-2.45026600	1.93699400	-0.08306100
O	-3.38813100	1.38639400	-0.59711000
O	-2.41739200	3.05640000	0.34897800
H	0.00040500	2.95872200	0.00002000

Ph-5-31245-R5-Nu1

C	0.29470500	-0.29727800	0.30895900
C	-1.18961000	-0.68140800	0.23849100
C	0.32765100	1.20759300	0.44176600
C	-2.13726600	0.19446200	-0.20437400
C	-0.66665300	2.01404800	-0.10755000
C	-1.89430900	1.54916300	-0.50566300
N	-0.39665200	3.43360400	-0.43361400
O	0.55899900	3.65627700	-1.13581700
O	-1.18996800	4.24973900	-0.04709000
N	-3.56246600	-0.18513100	-0.33270600
O	-4.18296300	-0.35162700	0.68249100
O	-3.99961000	-0.21393700	-1.45377800
N	-1.62962700	-2.01859300	0.62206800
O	-2.59258400	-2.47765700	0.05389700
O	-1.01849200	-2.58655300	1.49592700
N	0.84765900	-0.88117900	1.66107800
O	0.40569900	-0.40217000	2.66735700
O	1.65198800	-1.76644300	1.58904300
N	1.40098400	1.81934900	1.12565000
O	2.37771400	1.13221600	1.39658400
O	1.31346900	2.99853200	1.42605200
C	1.18344700	-0.85828800	-0.88781800
C	1.12413700	-2.40946900	-0.96572200
C	2.63789000	-0.29886400	-0.83217700
C	0.52000100	-0.38695900	-2.20169200
O	0.02717900	-2.91004300	-1.06528500
C	2.35222100	-3.27384300	-1.05485700
O	3.54710700	-0.94705000	-0.38264900
C	2.91475000	1.04012300	-1.48169000
H	-0.47850500	-0.83167900	-2.29085800
H	0.43628900	0.70624600	-2.24721400
H	1.11837600	-0.72049100	-3.06180400
H	2.01765900	-4.29500200	-1.26820200
H	3.02669600	-2.91613300	-1.84418500
H	2.90981000	-3.22456800	-0.11375300
H	2.99596100	0.88332900	-2.56884000
H	2.13119100	1.79104700	-1.32289900
H	3.87738200	1.40358400	-1.10606200
H	-2.64426400	2.18135900	-0.97339900

Ph-5-31245-R5-Nu3

C	-0.13698900	-1.07352300	0.02024500
C	0.96071800	-0.48219000	-0.84844500
C	-1.40589800	-0.39418800	-0.45672900

C	0.91191700	0.81986500	-1.28777600
C	-1.38700500	0.92874100	-0.87333600
C	-0.23712700	1.60868400	-1.23196300
N	-2.62096300	1.74881400	-0.83428900
O	-3.25605800	1.73186800	0.19019100
O	-2.85713100	2.42854500	-1.79889900
N	2.13256000	1.51591300	-1.75980300
O	2.02907500	2.15578600	-2.77458000
O	3.11287200	1.44963600	-1.06341000
N	2.08648800	-1.30146000	-1.21529400
O	2.73087500	-0.98777000	-2.19347700
O	2.31476000	-2.28033900	-0.53277600
N	-0.23440900	-2.58331300	-0.38018000
O	-0.39043200	-2.79635300	-1.55346500
O	-0.13698700	-3.41108400	0.47753200
N	-2.63582900	-1.11713900	-0.51731200
O	-2.70406200	-2.19536800	0.04847300
O	-3.55418500	-0.63644200	-1.15474200
C	0.07871200	-0.92396800	1.59185800
C	0.38386800	0.56339600	1.86262700
C	1.23048500	-1.70809300	2.08444200
C	-1.15037600	-1.38523300	2.40193000
C	-0.64804300	1.46214700	2.15412700
C	1.69010700	1.05123900	1.74624800
N	2.10270600	-2.26894800	2.58893200
H	-1.29491100	-2.46470900	2.30746700
H	-0.99129900	-1.11985000	3.45524000
H	-2.05887400	-0.88440300	2.05054000
C	-0.38164000	2.82358100	2.29595500
H	-1.67823600	1.11958000	2.23721700
C	1.95713700	2.41151100	1.88610600
H	2.50961800	0.36664300	1.51834900
C	0.91974700	3.30381200	2.15626100
H	-1.20217300	3.51015200	2.50964500
H	2.98061300	2.77171800	1.77458500
H	1.12634800	4.36983100	2.26236300
H	-0.22844600	2.67146900	-1.45786300

Ph-5-31245-R5-Nu2

C	-0.87455200	-0.12482000	-0.73628900
C	-1.36379100	1.28797600	-0.47895500
C	-1.83973700	-1.02865500	0.01024100
C	-1.86554900	1.62251500	0.75458200
C	-2.30059000	-0.63210000	1.25429600
C	-2.25669600	0.67625100	1.70689100
N	-2.79750300	-1.62469800	2.23715500
O	-2.06375800	-2.54327700	2.49427000
O	-3.85873900	-1.39584400	2.75683600
N	-1.99251000	3.03257100	1.18913700
O	-3.10728400	3.42995500	1.40595400
O	-0.97044600	3.64008600	1.36244900
N	-1.14363700	2.30256700	-1.47177100

O	-1.68452800	3.37702100	-1.31942000
O	-0.43729200	2.02069400	-2.41740000
N	-1.00284300	-0.41398600	-2.25188400
O	-2.09981500	-0.24886100	-2.71545800
O	-0.02387100	-0.76943700	-2.83390000
N	-2.25018400	-2.28956200	-0.52303100
O	-1.67010400	-2.73177700	-1.49531900
O	-3.18865500	-2.85035700	0.01346400
C	0.57570900	-0.33304000	-0.21498600
Br	1.22602000	-2.14434800	-0.33684000
S	1.89083000	0.86813000	-0.72615400
H	0.50440200	-0.12592800	0.86337000
O	2.35238700	0.61494400	-2.07923100
O	1.38419900	2.17947900	-0.32609100
C	3.21075100	0.45780600	0.39717600
C	4.26344400	-0.33439000	-0.05572000
C	3.17257700	0.98402300	1.68744000
C	5.30336000	-0.62264900	0.82517500
H	4.25792600	-0.70900000	-1.07995500
C	4.21725900	0.68195200	2.55884000
H	2.34726600	1.63011800	1.99162800
C	5.27583700	-0.11970400	2.12762200
H	6.13862000	-1.24011500	0.49345400
H	4.20937100	1.08191200	3.57316900
H	6.09267500	-0.35004100	2.81298800
H	-2.57237200	0.95491800	2.70883800

Ph-5-31245-R5-Nu4

C	0.00911400	-1.01516800	0.03574400
C	-0.05092600	-1.61106400	1.45600000
C	1.26329100	-0.21315400	-0.24403600
C	-0.21734200	-0.52566300	2.49994000
H	-0.88535300	-2.32026800	1.49711300
H	0.88010000	-2.16837400	1.61409000
C	-1.16776700	-0.14337800	-0.34954400
C	1.26238500	1.16436100	-0.28710600
C	-1.62412700	-0.23368100	2.94601600
C	0.84219400	0.13031800	2.98393000
C	-1.08327800	1.23035200	-0.39880400
C	0.11081100	1.94141500	-0.32996700
H	-2.10286100	-1.14853700	3.32864400
H	-1.64308600	0.52980800	3.73489400
H	-2.24136300	0.12871600	2.10871700
H	1.85693900	-0.09617500	2.64637600
H	0.72489600	0.92268000	3.72735200
N	-2.30086900	2.07407400	-0.42585900
O	-3.05169600	1.98080800	0.51126400
O	-2.40624200	2.84503400	-1.34278000
N	2.52025200	1.93793600	-0.17002100
O	2.76409100	2.71765000	-1.05362800
O	3.15893000	1.78623500	0.83883000
N	2.48901500	-0.92381400	-0.42160600

O	3.45254500	-0.32896600	-0.86748000
O	2.50243900	-2.11236200	-0.14699600
N	0.01431300	-2.21801800	-0.99124900
O	0.05994500	-1.90796200	-2.15572200
O	-0.02722600	-3.33611600	-0.56811800
N	-2.41507700	-0.78857500	-0.61160200
O	-2.52519600	-1.95851400	-0.28521500
O	-3.29622300	-0.15981700	-1.16646900
H	0.14021800	3.02698600	-0.29735800

Ph-6-123456-R1

C	0.00000000	1.40367000	0.01791700
C	-1.21561400	0.70183500	-0.01791700
C	1.21561400	0.70183500	-0.01791700
C	-1.21561400	-0.70183500	0.01791700
C	1.21561400	-0.70183500	0.01791700
C	0.00000000	-1.40367000	-0.01791700
C	0.00000000	2.92018500	0.30558000
C	-2.52895400	1.46009300	-0.30558000
C	2.52895400	1.46009200	-0.30558000
C	-2.52895400	-1.46009200	0.30558000
C	2.52895400	-1.46009300	0.30558000
C	0.00000000	-2.92018500	-0.30558000
F	3.18840300	1.84082500	0.77335200
F	2.26934500	2.53739700	-1.04106300
F	3.33212300	0.69661200	-1.04106300
F	3.18840300	-1.84082500	-0.77335200
F	3.33212300	-0.69661200	1.04106300
F	2.26934500	-2.53739700	1.04106300
F	1.06277800	-3.23400900	-1.04106300
F	0.00000000	-3.68165000	0.77335200
F	-1.06277800	-3.23400900	-1.04106300
F	-2.26934500	-2.53739700	1.04106300
F	-3.33212300	-0.69661200	1.04106300
F	-3.18840300	-1.84082500	-0.77335200
F	-3.33212300	0.69661200	-1.04106300
F	-3.18840300	1.84082500	0.77335200
F	-2.26934500	2.53739700	-1.04106300
F	-1.06277800	3.23400900	1.04106300
F	1.06277800	3.23400900	1.04106300
F	0.00000000	3.68165000	-0.77335200

Ph-6-123456-R1-Nu1

C	1.05693500	0.00440000	-0.10686000
C	2.47877200	0.02807200	-0.74985200
C	1.36039800	-0.23102900	1.47783900
F	3.39273500	0.63223300	0.01660900
F	2.52266200	0.62261300	-1.93658300
F	2.93025800	-1.22457800	-0.90753600
C	0.20405400	-1.11112700	-0.74242800
C	0.23172400	1.27723400	-0.39916700

C	0.13019300	-0.38685300	2.40373800
C	2.23419500	-1.50650200	1.65931000
C	2.00594400	1.03953800	2.09924800
C	0.55566400	-1.92122400	-1.96293600
C	-1.11626900	-1.18638000	-0.27315400
C	0.87535600	2.56774600	-0.89940800
C	-1.12211800	1.22245600	-0.19101000
H	-0.48701400	0.51789400	2.37382500
H	0.49624200	-0.51022200	3.43659400
H	-0.48560400	-1.25544800	2.16498700
C	1.57402000	-2.86295000	1.60148100
O	3.42404200	-1.42764700	1.85114900
C	3.09255900	0.93680500	3.14320200
O	1.53072800	2.11950700	1.84093100
F	-0.54194700	-2.20661500	-2.68683900
F	1.15682600	-3.10757500	-1.71896400
F	1.36083100	-1.29057800	-2.82778400
C	-1.79448500	-2.53908000	-0.08519200
C	-1.76538100	-0.02368000	0.15365600
F	2.17400100	2.67103100	-0.62282100
F	0.31286700	3.64454300	-0.34415600
F	0.76996400	2.70928600	-2.22882200
C	-2.04528300	2.40804100	-0.47970200
H	1.13670300	-3.02400600	0.60688500
H	0.76510900	-2.95743700	2.33646300
H	2.34365200	-3.61906200	1.79288700
H	4.05101300	0.75129500	2.64371000
H	2.92684300	0.09958000	3.83244800
H	3.12032500	1.88876100	3.68518300
F	-1.30107700	-3.52264500	-0.83293800
F	-3.10803700	-2.49418400	-0.30625000
F	-1.62195000	-2.96363100	1.18247600
C	-2.99247700	-0.01273500	1.02368500
F	-1.72196800	3.03108600	-1.61710600
F	-2.07004400	3.32215200	0.48927000
F	-3.30727200	2.00209000	-0.65446700
F	-4.16480800	-0.14791200	0.39071500
F	-3.07074700	1.13304900	1.72540500
F	-2.96654300	-0.99288100	1.94153100

Ph-6-123456-R1-Nu3

C	-1.37619500	0.03255300	-0.13125500
C	-0.43577300	-0.99925600	-0.80140800
C	-0.70440900	1.42840500	-0.21601400
C	0.91881400	-0.76634900	-0.90460800
C	0.66961600	1.55888400	-0.28099600
C	1.53070600	0.45200100	-0.49921800
C	1.84115100	-1.76190600	-1.63775200
C	-0.95169100	-2.40218000	-1.09709100
C	2.96060200	0.45263700	-0.02942300
C	1.34804400	2.93960800	-0.20009300
C	-1.55809700	2.68760600	-0.14260300

C	-2.73640500	0.09969100	-0.95066000
F	-2.70292500	1.08911000	-1.84660200
F	-2.99437200	-0.98941800	-1.66852800
F	-3.80731300	0.28098900	-0.17648400
F	-2.82190400	2.45844400	0.23004700
F	-1.64802100	3.36078600	-1.29528600
F	-1.09042400	3.55771700	0.77363600
F	2.57308900	2.91871600	-0.72452600
F	0.70846300	3.88569800	-0.88638200
F	1.46721300	3.36496500	1.05987400
F	3.90645700	0.83974300	-0.89963900
F	3.33621800	-0.77360800	0.37135000
F	3.11275400	1.24543700	1.05026200
F	2.33402000	-2.74013100	-0.88356600
F	1.21929100	-2.32709700	-2.67654100
F	2.89001400	-1.12176400	-2.16357200
F	-1.29705200	-2.63909500	-2.36737700
F	-0.05017000	-3.34523100	-0.78347100
F	-2.01822800	-2.71048500	-0.34486800
C	-1.66662700	-0.35935000	1.45011400
C	-0.39207200	-0.98115200	2.04447700
C	-2.77793500	-1.31359100	1.64131600
C	-2.09329500	0.84307200	2.32477300
C	0.63711500	-0.15450100	2.51345800
C	-0.22830800	-2.36755400	2.13396300
N	-3.66567100	-2.00233400	1.90370200
H	-3.09931400	1.18314500	2.06418700
H	-2.08541400	0.52120300	3.37420900
H	-1.39423000	1.67984300	2.22017000
C	1.80179200	-0.70333900	3.04599500
H	0.54536600	0.93004700	2.45139100
C	0.94121400	-2.91653600	2.65891100
H	-1.02105200	-3.03197800	1.78940400
C	1.96154200	-2.08615300	3.11650700
H	2.59600500	-0.03989400	3.39000700
H	1.04848200	-4.00098500	2.71140700
H	2.87711900	-2.51357900	3.52797400

Ph-6-123456-R1-Nu2

C	-0.06400600	-0.17785100	0.88912400
C	0.52634200	1.23762500	0.76900200
C	0.97324800	-1.22932800	0.46191000
C	1.70786400	1.47944400	0.09429300
C	2.15639700	-0.89444800	-0.13885100
C	2.51212700	0.45045300	-0.45557700
C	2.29642100	2.90252300	0.04945800
C	-0.32120900	2.39796000	1.26705700
C	3.46572600	0.72506100	-1.58427500
C	3.24436800	-1.96405000	-0.34197400
C	0.58043000	-2.67877200	0.67227800
C	-0.32683800	-0.45452500	2.42361200
F	0.85946800	-0.64062500	3.01060300

F	-0.90367200	0.54255100	3.07825700
F	-1.05103400	-1.54606100	2.65312200
F	-0.74379200	-2.83302400	0.59269400
F	0.94716100	-3.17881800	1.86069200
F	1.08245000	-3.49318100	-0.26128500
F	4.45654300	-1.40497200	-0.36191200
F	3.27197700	-2.82256500	0.68200600
F	3.12586900	-2.67195100	-1.46111800
F	4.75037300	0.95146100	-1.27261100
F	3.07342300	1.79308000	-2.30230200
F	3.48303800	-0.30315900	-2.45257500
F	1.82962600	3.64763800	-0.94884200
F	2.07599800	3.56406800	1.18762900
F	3.62429100	2.86677200	-0.07651400
F	0.01306300	2.86714100	2.47288000
F	-0.28445100	3.44361900	0.42701200
F	-1.62941900	2.08536700	1.34792500
C	-1.37825200	-0.21282900	-0.04373400
Br	-1.04895600	-0.97829900	-1.79336400
S	-3.04240700	-0.78878200	0.54919100
H	-1.62172000	0.82092900	-0.28588100
O	-3.24178100	-2.21908500	0.37168100
O	-3.24295600	-0.16793400	1.85404800
C	-4.11593300	0.05312700	-0.60296700
C	-4.67045900	-0.66118600	-1.66201100
C	-4.40365400	1.39912900	-0.37969400
C	-5.53043500	0.00344400	-2.53465700
H	-4.42861500	-1.71716400	-1.78770500
C	-5.26074300	2.05149500	-1.26366100
H	-3.96669900	1.91785100	0.47535400
C	-5.82021100	1.35461100	-2.33668000
H	-5.97693000	-0.53599200	-3.37059500
H	-5.49846700	3.10446500	-1.10940300
H	-6.49302900	1.86966800	-3.02379100

Ph-6-123456-R1-Nu4

C	-0.05856900	-1.46213100	0.11781800
C	-0.13194300	-2.16737100	1.51800000
C	1.26217200	-0.66589800	-0.04948700
C	-0.18238900	-1.17645700	2.66062200
H	-1.00801700	-2.82139900	1.54796900
H	0.74230000	-2.81047800	1.63258500
C	-1.28279400	-0.54557200	-0.13633900
C	1.27687500	0.70084500	-0.17965300
C	-1.53988200	-0.73660200	3.14104600
C	0.94661600	-0.71710100	3.21122700
C	-1.15605200	0.81961500	-0.24396600
C	0.09349600	1.49777300	-0.15775700
H	-2.16062200	-1.60889700	3.39926900
H	-1.45754400	-0.08849200	4.02373900
H	-2.08036000	-0.18269500	2.35850700
H	1.92620800	-1.04760200	2.85655600

H	0.92461500	0.00929000	4.02745400
C	2.57158300	1.45203100	-0.55236500
C	2.57576500	-1.43005600	-0.02539400
C	0.15443700	2.91216200	0.33437400
C	-2.36143800	1.69536300	-0.63716800
C	-2.66236300	-1.18214500	-0.16832900
C	-0.06417500	-2.54977500	-0.99696800
F	0.06119400	-1.99005000	-2.20091500
F	0.92758100	-3.43535000	-0.86869400
F	-1.18323500	-3.27709500	-1.01985600
F	-2.76072000	-2.29185900	0.58068600
F	-3.07831700	-1.52769000	-1.39628400
F	-3.61192600	-0.37892500	0.33793800
F	-1.96052900	2.82890600	-1.21733700
F	-3.12630100	1.09393600	-1.55438700
F	-3.14380700	2.04751500	0.38185600
F	0.21578000	3.90210300	-0.57349300
F	1.22685400	3.09979100	1.12922300
F	-0.91623100	3.20436100	1.09982800
F	3.36261600	1.75114000	0.47513600
F	3.29618900	0.76369400	-1.44158300
F	2.29072000	2.60694400	-1.16207600
F	2.98619700	-1.83943600	-1.23601900
F	3.57881000	-0.70728800	0.49288300
F	2.55350100	-2.53045500	0.74419800

Ph-6-123456-R2

C	-1.40426700	0.00043800	0.00000000
C	-0.70222700	0.00051300	1.21614800
C	-0.70222700	0.00051300	-1.21614800
C	0.70221100	0.00045500	1.21618000
C	0.70221100	0.00045500	-1.21618000
C	1.40429200	0.00014700	0.00000000
C	1.42149300	-0.00010600	2.46163500
N	1.99762600	-0.00041300	3.46020100
C	-1.42148900	-0.00019000	2.46163600
N	-1.99765500	-0.00054100	3.46018200
C	2.84248200	-0.00010700	0.00000000
N	3.99533100	0.00011700	0.00000000
C	1.42149300	-0.00010600	-2.46163500
N	1.99762600	-0.00041300	-3.46020100
C	-1.42148900	-0.00019000	-2.46163600
N	-1.99765500	-0.00054100	-3.46018200
C	-2.84245400	0.00042200	0.00000000
N	-3.99529800	-0.00013300	0.00000000

Ph-6-123456-R2-Nu1

C	-0.69322100	-0.37404800	-0.65193400
C	0.53265100	-1.27960800	-0.81141100
C	-0.22036900	1.05032400	-0.94043500
C	1.75581900	-0.83993500	-0.34597300

C	1.01352700	1.45271300	-0.47939900
C	1.98233000	0.51917400	-0.00842000
C	2.85241400	-1.77188800	-0.24909400
N	3.72687600	-2.51891900	-0.15249000
C	0.38833300	-2.59015300	-1.34857900
N	0.25471900	-3.66014700	-1.77254900
C	3.20421800	0.94323600	0.58210700
N	4.19571800	1.28254600	1.07616200
C	1.37813300	2.84620600	-0.54408900
N	1.67945400	3.95957200	-0.58332800
C	-1.02275600	1.96400100	-1.68815000
N	-1.61966100	2.72307000	-2.32596500
C	-1.69602500	-0.78089100	-1.66016700
N	-2.44240500	-1.15738300	-2.45335200
C	-1.36097500	-0.55721500	0.78789700
C	-2.64690900	0.29006100	0.87765900
C	-0.42822200	0.00788700	1.88386500
C	-1.71428500	-2.03084400	1.02757200
O	-3.02239500	0.96154200	-0.04841500
C	-3.44816600	0.18689900	2.15343300
O	-0.49272100	1.18782700	2.13652800
C	0.45819400	-0.92697200	2.66475700
H	-2.43943400	-2.38224100	0.27900200
H	-0.82607900	-2.67253300	0.96984200
H	-2.15733100	-2.16771500	2.02289800
H	-4.14309000	-0.66094500	2.05238300
H	-2.82307500	0.01474500	3.03829100
H	-4.03761300	1.10244000	2.27594700
H	-0.16291700	-1.43485100	3.42024500
H	0.91014100	-1.70746200	2.04131100
H	1.22927500	-0.34124100	3.17798800

Ph-6-123456-R2-Nu3

C	-1.33591500	0.34737500	-0.35536700
C	-0.82804200	-0.89491800	-1.07976100
C	-0.42225400	1.51097200	-0.72285700
C	0.52350200	-1.06219200	-1.30119900
C	0.92229100	1.28991900	-0.95184300
C	1.46040100	-0.00885600	-1.13066000
C	1.00459200	-2.34038000	-1.76599100
N	1.39886400	-3.36466200	-2.12239400
C	-1.75074100	-1.91415200	-1.45834300
N	-2.50235500	-2.73578100	-1.77625000
C	2.85969800	-0.22540600	-1.26363500
N	4.00000900	-0.40297800	-1.36521600
C	1.80909600	2.42320400	-1.05664200
N	2.52499800	3.32577400	-1.12410600
C	-0.95262100	2.83262300	-0.75235100
N	-1.39931700	3.90161900	-0.76298600
C	-2.71347600	0.64252500	-0.80478800
N	-3.79193600	0.87045900	-1.14125400
C	-1.39370000	0.13266600	1.25075600

C	0.00298700	-0.21283600	1.76606700
C	-2.30675500	-0.99999300	1.49663000
C	-1.99843100	1.36975700	1.93581800
C	0.88905900	0.80410700	2.14099600
C	0.44968000	-1.53996800	1.78562500
N	-3.04308800	-1.86749600	1.68541100
H	-3.03475800	1.52557000	1.60895900
H	-1.98630700	1.22734800	3.02396100
H	-1.42363200	2.27243100	1.69811400
C	2.20887700	0.50382200	2.47460800
H	0.56654200	1.84540000	2.15087400
C	1.76839400	-1.84021900	2.12086700
H	-0.23365300	-2.34890800	1.51849900
C	2.65595900	-0.81683700	2.45226600
H	2.89115500	1.30987100	2.74774700
H	2.10256200	-2.87850700	2.11684600
H	3.69225900	-1.04903300	2.70138900

Ph-6-123456-R2-Nu2

C	-0.74578800	0.08584200	-0.15376900
C	-1.55816100	-1.13691200	-0.57382600
C	-1.73969400	1.22838500	-0.36357200
C	-2.77154900	-1.30551900	0.06604800
C	-2.95701100	1.08246500	0.28782700
C	-3.38802100	-0.20006600	0.72295300
C	-3.49092700	-2.54617400	-0.05559500
N	-4.07044000	-3.54054900	-0.14603000
C	-1.13647700	-2.00856500	-1.61667000
N	-0.81437700	-2.69081400	-2.49494600
C	-4.56966900	-0.36316500	1.50294700
N	-5.52624600	-0.49538400	2.14104400
C	-3.85477500	2.20213300	0.40240100
N	-4.57614100	3.09790900	0.50277700
C	-1.51519800	2.34015700	-1.22236000
N	-1.39113300	3.24325500	-1.93706100
C	-0.43029000	0.01107500	1.30460600
N	-0.18715900	-0.00509600	2.43133100
C	0.58281700	0.23197100	-0.91781500
Br	1.49075100	1.88602400	-0.51568300
S	1.74452900	-1.22922500	-0.63633600
H	0.41634700	0.20036500	-2.00276800
O	1.01680600	-2.20674800	0.16825900
O	2.22409400	-1.55356200	-1.97285000
C	3.12821300	-0.67268100	0.32838600
C	3.06098300	-0.75912200	1.71733400
C	4.26798900	-0.23193800	-0.34256900
C	4.17347100	-0.36125900	2.45702400
H	2.15812800	-1.12901900	2.20436000
C	5.37102800	0.15756700	0.41183600
H	4.28156100	-0.19991800	-1.43256400
C	5.31983600	0.09708900	1.80647900
H	4.14488700	-0.41660400	3.54549800

H	6.27400200	0.50655500	-0.08979400
H	6.18692900	0.40492400	2.39246700

Ph-6-123456-R2-Nu4

C	0.93088300	-0.63365100	-0.60494500
C	2.05133500	-1.15614200	0.35061200
C	0.79865200	0.88678900	-0.50687100
C	1.95474200	-0.62228600	1.75980600
H	1.99959700	-2.25821800	0.34983800
H	3.02296400	-0.87671600	-0.08133400
C	-0.42654700	-1.29173400	-0.34524400
C	-0.39419100	1.52627100	-0.27067100
C	0.71964600	-0.94687100	2.55798800
C	2.95156100	0.11101100	2.26547500
C	-1.58447800	-0.57864800	-0.13771200
C	-1.62947400	0.84054000	-0.09339700
H	0.43917700	-2.00582800	2.44837600
H	0.87486300	-0.72882300	3.62253300
H	-0.13940800	-0.34890400	2.21349700
H	3.82989000	0.36545400	1.66761100
H	2.91245700	0.48314500	3.29208600
C	-0.39755900	2.96813600	-0.18448000
N	-0.40705600	4.11970600	-0.11439200
C	2.00648200	1.62989600	-0.66255500
N	3.00920100	2.19188200	-0.80531200
C	-2.84118600	1.54038100	0.14202500
N	-3.83110000	2.11233500	0.33514800
C	-2.81126800	-1.30667700	0.08656400
N	-3.79376000	-1.88362500	0.26869200
C	-0.44337100	-2.71676100	-0.32311100
N	-0.41516700	-3.87497700	-0.30389500
C	1.35055600	-0.98905100	-1.98956300
N	1.67299200	-1.26493900	-3.06254400

Ph-6-123456-R3

C	1.03041100	0.94355200	0.16273300
C	1.33234500	-0.42058600	0.16273300
C	2.13531000	1.96471500	0.24292200
C	-0.30193400	1.36413800	0.16273300
C	2.76914800	-0.86687500	0.24292200
C	0.30193400	-1.36413800	0.16273300
O	3.05162900	1.76185900	-0.69035600
O	2.16411200	2.84311000	1.06081900
C	-0.63383800	2.83159000	0.24292200
C	-1.33234500	0.42058600	0.16273300
O	3.05162900	-1.76185900	-0.69035600
O	3.54426200	-0.45262100	1.06081900
C	-1.03041100	-0.94355200	0.16273300
C	0.63383800	-2.83159000	0.24292200
C	4.22231400	2.57268600	-0.60489300
O	0.00000000	3.52371800	-0.69035600

O	-1.38015000	3.29573100	1.06081900
C	-2.76914800	0.86687500	0.24292200
C	4.33916900	-2.37028800	-0.60489300
C	-2.13531000	-1.96471500	0.24292200
O	0.00000000	-3.52371800	-0.69035600
O	1.38015000	-3.29573100	1.06081900
H	3.95243600	3.63499300	-0.64642900
H	4.74519200	2.36407000	0.33772500
H	4.84620900	2.29887800	-1.45957400
C	-0.11685500	4.94297500	-0.60489300
O	-3.05162900	1.76185900	-0.69035600
O	-3.54426200	0.45262100	1.06081900
H	4.41994100	-2.92742100	0.33772500
H	5.12421400	-1.60541300	-0.64642900
H	4.41399100	-3.04750100	-1.45957400
O	-3.05162900	-1.76185900	-0.69035600
O	-2.16411200	-2.84311000	1.06081900
C	0.11685500	-4.94297500	-0.60489300
H	0.32525100	5.29149200	0.33772500
H	0.43221800	5.34637900	-1.45957400
H	-1.17177800	5.24040600	-0.64642900
C	-4.33916900	2.37028800	-0.60489300
C	-4.22231400	-2.57268600	-0.60489300
H	-0.32525100	-5.29149200	0.33772500
H	1.17177800	-5.24040600	-0.64642900
H	-0.43221800	-5.34637900	-1.45957400
H	-4.41994100	2.92742100	0.33772500
H	-4.41399100	3.04750100	-1.45957400
H	-5.12421400	1.60541300	-0.64642900
H	-4.74519200	-2.36407000	0.33772500
H	-4.84620900	-2.29887800	-1.45957400
H	-3.95243600	-3.63499300	-0.64642900

Ph-6-123456-R3-Nu1

C	0.95824900	-0.64122300	-0.04605200
C	-0.45564700	-1.15037900	-0.30454600
C	0.97877400	0.83868700	-0.47128000
C	1.95494800	-1.41877800	-0.92731600
C	-0.60941100	-2.55476700	-0.68646400
C	-1.54632600	-0.33178200	-0.10377000
C	2.23439000	1.40619500	-1.02400500
C	-0.13281100	1.61831500	-0.32119100
O	1.52378400	-1.50154700	-2.18587200
O	2.99717400	-1.88266900	-0.55238500
O	-1.76977400	-2.82688900	-1.30991800
O	0.21793000	-3.42119200	-0.48528200
C	-1.41478300	1.07299800	0.02244200
C	-2.91634400	-0.95453400	-0.01010700
O	2.44233100	2.67605800	-0.65039700
O	3.00005200	0.83013600	-1.76147600
C	-0.07339400	3.10519200	-0.57449800
C	2.44428700	-2.05968300	-3.10550100

C	-2.05225200	-4.19764200	-1.53098800
C	-2.52201400	1.95196500	0.36223900
O	-3.71718500	-0.57866400	-1.00282500
O	-3.25148500	-1.70146100	0.87385500
C	3.60272300	3.29459100	-1.17548900
O	0.06047700	3.77352100	0.57046200
O	-0.14986200	3.62647700	-1.65413300
H	1.95040500	-2.05471600	-4.08226700
H	3.35340000	-1.44365000	-3.13185900
H	2.70744500	-3.08618900	-2.81578900
H	-3.02613400	-4.23630200	-2.02923800
H	-2.09168400	-4.74304200	-0.57763000
H	-1.28220400	-4.65442200	-2.16635000
O	-3.51354900	1.31409800	1.02809900
O	-2.60206200	3.14305700	0.12750300
C	-5.04402600	-1.07538900	-0.93895600
H	3.61533600	4.31489100	-0.77897100
H	3.57411600	3.31393600	-2.27344700
H	4.50080600	2.74985100	-0.85383700
C	-0.03891700	5.18279400	0.46277500
C	-4.68592000	2.06796100	1.27224700
H	-5.04641800	-2.17336000	-0.97508300
H	-5.52764900	-0.75231100	-0.00659700
H	-5.57195400	-0.66503700	-1.80490600
H	-1.01690900	5.45349100	0.04205400
H	0.75466600	5.57999400	-0.18491000
H	0.06461100	5.58136900	1.47680400
H	-5.15073800	2.38508800	0.32760100
H	-4.45816200	2.96263000	1.86657800
H	-5.36614300	1.41163700	1.82521200
C	1.40203300	-0.83755200	1.47986300
C	2.85339900	-0.38023500	1.72963200
C	1.20349800	-2.31143700	1.91896800
C	0.57820500	0.07862300	2.41141900
O	3.32210900	0.55615700	1.12563600
C	3.64391200	-0.99897000	2.86087100
O	2.11948900	-3.09719800	1.91786100
C	-0.14421700	-2.73052600	2.46842900
H	0.74721900	1.13044700	2.14716500
H	-0.49499800	-0.13076100	2.32760000
H	0.87895500	-0.06991300	3.45984100
H	4.40548600	-0.27826500	3.17986000
H	3.00833100	-1.29666600	3.70488800
H	4.12102700	-1.91054400	2.48049900
H	-0.19816600	-2.40111800	3.51878900
H	-0.99875700	-2.28807200	1.94228900
H	-0.19746400	-3.82457700	2.44036200

Ph-6-123456-R3-Nu3

C	-0.44969600	-0.97660200	0.07321100
C	1.06206600	-1.18676700	0.10046800
C	-0.74919700	0.39861600	0.67981400

C	-1.11694800	-2.07599200	0.92591800
C	1.57062100	-2.55051900	0.30996000
C	1.91217800	-0.11968600	-0.08396100
C	-2.06936400	0.57862500	1.31602400
C	0.14756300	1.42863000	0.52961000
O	-0.62713800	-2.06397500	2.16646200
O	-1.94041500	-2.86249800	0.54852400
O	2.86511400	-2.58994000	0.67809300
O	0.91768500	-3.56640500	0.21316900
C	1.46175000	1.22566500	0.00847800
C	3.36584600	-0.37784000	-0.40093600
O	-2.45369200	1.85973700	1.42107500
O	-2.78469400	-0.31970500	1.70346200
C	-0.19613900	2.83847700	0.95377800
C	-1.20499100	-3.00672000	3.05145600
C	3.44942500	-3.87779700	0.76372500
C	2.28874600	2.37484600	-0.34659400
O	4.17285100	0.03042000	0.57590700
O	3.74890400	-0.87356600	-1.42702100
C	-3.71593500	2.08482600	2.02455700
O	-0.68865400	3.52737000	-0.07727400
O	-0.02978400	3.28947700	2.05263000
H	-0.69910700	-2.88187900	4.01385200
H	-2.28115400	-2.81098500	3.15625200
H	-1.05962100	-4.02680200	2.67074600
H	4.49527000	-3.72389100	1.04785300
H	3.39005000	-4.39639400	-0.20295400
H	2.93627200	-4.48565700	1.52046900
O	3.32611000	2.05036500	-1.14724300
O	2.09904500	3.52992500	-0.01852800
C	5.55945900	-0.11568000	0.31884100
H	-3.86370500	3.16942900	2.03267500
H	-3.73340600	1.69070200	3.04925800
H	-4.50794200	1.59365800	1.44224200
C	-0.89024000	4.91007100	0.16257500
C	4.23023200	3.09557800	-1.45453600
H	5.81272300	-1.17270300	0.15878800
H	5.84303500	0.45396900	-0.57717900
H	6.08227900	0.27174000	1.19842600
H	0.06379200	5.37895200	0.43954600
H	-1.61629900	5.06151400	0.97320500
H	-1.26948300	5.33664700	-0.77112900
H	4.70183000	3.48444200	-0.54101700
H	3.71286100	3.92183900	-1.95956900
H	4.98743800	2.66327000	-2.11676100
C	-1.04427900	-1.04710900	-1.42203700
C	-2.52185500	-0.62425700	-1.48257500
C	-0.90923200	-2.41493000	-1.96666900
C	-0.21942400	-0.16928200	-2.39206300
C	-2.85767200	0.73737100	-1.50342000
C	-3.55936400	-1.56336000	-1.52502800
N	-0.78061500	-3.42339100	-2.51299500
H	0.81114600	-0.53651100	-2.46714200

H	-0.68471100	-0.20133500	-3.38659900
H	-0.19182200	0.87000300	-2.04797300
C	-4.19211900	1.14434100	-1.52190200
H	-2.08033400	1.50049200	-1.47094400
C	-4.89183400	-1.15676500	-1.55751000
H	-3.31842200	-2.62549600	-1.51236600
C	-5.21632300	0.19961500	-1.54481900
H	-4.42644800	2.21055100	-1.52045000
H	-5.68197900	-1.90882500	-1.58763800
H	-6.26025900	0.51724200	-1.56285500

Ph-6-123456-R3-Nu2

C	0.38455800	-1.09462900	0.54223900
C	1.76102100	-0.54520800	0.20732800
C	-0.26598600	-0.12022000	1.53540000
C	0.46739300	-2.51113400	1.10764300
C	2.88413800	-1.47566800	0.00346900
C	1.85623900	0.79741300	-0.06309500
C	-1.08776700	-0.65004300	2.64493200
C	-0.13004600	1.22138200	1.27234000
O	1.35395100	-2.59138200	2.09025200
O	-0.19257200	-3.43009600	0.70371100
O	4.08029200	-0.87864900	0.14618100
O	2.78782500	-2.65369300	-0.25632500
C	0.81401400	1.69822300	0.30840100
C	3.09023500	1.33095100	-0.74832200
O	-1.95430400	0.25322500	3.12721200
O	-1.01908600	-1.76345800	3.11090600
C	-0.87273700	2.26821200	2.07151400
C	1.43586500	-3.85050200	2.73261200
C	5.20926500	-1.65277200	-0.22091000
C	0.76915500	3.09033000	-0.12849600
O	3.77582600	2.15628700	0.04055800
O	3.41419800	1.05182900	-1.87126800
C	-2.77310700	-0.17996400	4.19916200
O	-2.04668800	2.56946900	1.51587700
O	-0.45058000	2.78110100	3.07073900
H	2.19570900	-3.75239800	3.51391100
H	0.46219100	-4.10285800	3.17373800
H	1.72608900	-4.62758600	2.01268500
H	6.08557600	-1.01676400	-0.06113500
H	5.14450000	-1.95246600	-1.27606200
H	5.27689900	-2.55618900	0.39926800
O	1.55411300	3.32941100	-1.19965100
O	0.09370400	3.97578200	0.35799100
C	4.90991600	2.76518500	-0.55464400
H	-3.40110900	0.67409200	4.47161200
H	-2.16153800	-0.49148900	5.05640700
H	-3.39644200	-1.02688000	3.88311900
C	-2.75180500	3.63306400	2.13361400
C	1.61946300	4.67323900	-1.64093700
H	5.63273100	2.00360800	-0.87743000

H	4.60779400	3.35752000	-1.42969500
H	5.35306300	3.41162000	0.20869400
H	-2.14801800	4.54994900	2.09720600
H	-2.97580300	3.39053800	3.18127600
H	-3.67931800	3.76282000	1.56722900
H	2.02356500	5.32368300	-0.85267600
H	0.62266600	5.03936000	-1.92019900
H	2.28121600	4.67886500	-2.51315400
C	-0.47335200	-1.06963900	-0.75987100
Br	0.35305500	-1.85263800	-2.32376600
S	-2.21089500	-1.69946800	-0.65393200
H	-0.59797100	-0.01133000	-1.01917800
O	-2.37253000	-2.97060900	-1.34617300
O	-2.66786500	-1.56459100	0.72771400
C	-3.05141100	-0.44940300	-1.61366800
C	-3.47574900	-0.74979700	-2.90390800
C	-3.24306500	0.80569100	-1.03491800
C	-4.11694100	0.24614000	-3.64176400
H	-3.30444400	-1.74741700	-3.30995000
C	-3.87642200	1.79151400	-1.78794300
H	-2.89630100	1.00991600	-0.01792600
C	-4.31319600	1.51053900	-3.08554000
H	-4.46160300	0.03329800	-4.65438700
H	-4.02993300	2.78267800	-1.35924400
H	-4.81046800	2.28681000	-3.66899100

Ph-6-123456-R3-Nu4

C	-1.30352900	-0.30846900	0.46793600
C	-1.31631900	-0.39535500	2.02240400
C	-0.17653100	-1.13311600	-0.15806400
C	-1.07369000	-1.75847400	2.64508000
H	-2.28178600	0.01370600	2.35280300
H	-0.52850500	0.28669700	2.37530100
C	-2.65797300	-0.77413600	-0.08158500
C	-1.04600500	1.14658300	0.05536600
C	1.10574000	-0.64442600	-0.07473100
C	-0.48232700	-2.44269300	-0.74461200
C	-2.05690600	-2.64250700	2.84372400
C	0.34060800	-2.04351200	3.07916400
O	-2.70899900	-0.62997000	-1.41182500
O	-3.58479800	-1.18266100	0.56398200
C	-2.12978200	2.09915300	-0.22570800
C	0.24193000	1.61299500	0.09741300
C	1.35278800	0.72637400	0.24135700
C	2.28289000	-1.54967300	-0.34537800
O	0.50542500	-2.91758400	-1.52975000
O	-1.51503100	-3.06256700	-0.60016200
H	-3.07568400	-2.42877500	2.51869700
H	-1.85149700	-3.60988300	3.31067100
H	0.69049100	-1.26694300	3.77905400
H	1.03098200	-2.03650000	2.22173800
H	0.41704800	-3.02032600	3.57689400

C	-3.89297700	-1.09975700	-2.02995300
O	-3.32327300	1.68309600	0.23236100
O	-2.00827000	3.16641900	-0.78827300
C	0.51000600	3.09689700	0.01334000
C	2.66607100	1.26130300	0.56752100
O	2.93400100	-1.20095800	-1.45311300
O	2.61433300	-2.46519900	0.36186100
C	0.33890900	-4.23785100	-2.01320400
H	-4.01821300	-2.17363400	-1.83321300
H	-4.77090600	-0.56170600	-1.64590700
H	-3.77762700	-0.91986600	-3.10343400
C	-4.43511100	2.51069800	-0.05931300
O	1.00830400	3.43961500	-1.17244700
O	0.30718600	3.87243800	0.90874200
O	3.58289600	0.30177700	0.82547500
O	2.96284300	2.43829000	0.65341200
C	4.10653700	-1.94781000	-1.72979600
H	0.24609800	-4.94893200	-1.18060300
H	-0.56057400	-4.31072500	-2.63877800
H	1.23067400	-4.46500900	-2.60619400
H	-4.31207300	3.50093000	0.39904900
H	-4.54866000	2.63813300	-1.14472000
H	-5.31182300	2.00499200	0.35757900
C	1.38044900	4.80096000	-1.29876600
C	4.89155800	0.74958100	1.12240000
H	4.82876500	-1.83824200	-0.90861400
H	3.86793300	-3.01344000	-1.84934200
H	4.52160900	-1.54431800	-2.65825500
H	0.51097300	5.45475100	-1.14569700
H	2.15133500	5.04917500	-0.55622800
H	1.77386700	4.92453100	-2.31250400
H	5.31700600	1.30771600	0.27676100
H	4.88872100	1.40339100	2.00474900
H	5.48689800	-0.14787400	1.32053000

Ph-6-123456-R4

C	0.00000000	1.39212500	0.00000000
C	1.20561600	0.69606200	0.00000000
C	-1.20561600	0.69606200	0.00000000
C	1.20561600	-0.69606200	0.00000000
C	-1.20561600	-0.69606200	0.00000000
C	0.00000000	-1.39212500	0.00000000
F	-2.34932400	-1.35638300	0.00000000
F	0.00000000	-2.71276600	0.00000000
F	2.34932400	-1.35638300	0.00000000
F	2.34932400	1.35638300	0.00000000
F	0.00000000	2.71276600	0.00000000
F	-2.34932400	1.35638300	0.00000000

Ph-6-123456-R4-Nu1

C	-0.02161800	-0.05224000	-0.43246400

C	0.79950000	-1.21366400	-0.05488200
C	0.78916500	1.14447800	-0.17583400
C	2.16652000	-1.19554300	0.10897000
C	2.15529400	1.16574500	-0.03235400
C	2.89774600	-0.00774500	0.12769700
F	0.14497300	2.34651400	-0.21727100
F	2.79161500	2.34514900	-0.00795100
F	4.25041800	0.00122600	0.10149200
F	2.80691700	-2.35296300	0.33471400
F	0.17107700	-2.39061900	0.21394600
F	-0.38935900	-0.04838200	-1.84675600
C	-1.41775600	-0.05757800	0.29807500
C	-2.34268500	-1.19680100	-0.19752000
C	-2.22019500	1.22071600	-0.05151500
C	-1.19481600	-0.20472000	1.81004200
O	-2.10569600	-1.91704900	-1.12838700
C	-3.62287700	-1.37354900	0.60179700
O	-2.79656300	1.29644500	-1.11021700
C	-2.36982900	2.30443300	0.99187100
H	-0.80341400	-1.20699400	2.03022300
H	-0.45444700	0.52803200	2.15685400
H	-2.12113700	-0.05813200	2.38074800
H	-3.39684800	-1.94965100	1.51174100
H	-4.06293400	-0.41614200	0.91322600
H	-4.34084400	-1.93996800	-0.00195500
H	-3.15551400	1.99734300	1.70032700
H	-1.44801400	2.47407700	1.55658000
H	-2.69024400	3.22675300	0.49421600

Ph-6-123456-R4-Nu3

C	0.33005400	-1.04132800	-0.19975100
C	0.66750800	-0.53443200	1.12441600
C	1.15907400	-0.34707600	-1.17128700
C	1.80534100	0.17377000	1.43833100
C	2.30575600	0.35736300	-0.86552500
C	2.66703800	0.64612400	0.44792700
F	0.85491900	-0.45697200	-2.49405100
F	3.06835200	0.81404200	-1.86627200
F	3.83324100	1.25296100	0.74863200
F	2.05731000	0.47039300	2.71886000
F	-0.19348000	-0.80862600	2.13725800
F	0.59782600	-2.53917400	-0.28251200
C	-1.22825400	-1.06768900	-0.46881200
C	-1.81850700	0.32675300	-0.25928100
C	-1.83722500	-2.03443100	0.46946200
C	-1.58717300	-1.62740200	-1.86681400
C	-1.50578400	1.33760000	-1.17936200
C	-2.63228900	0.63950600	0.83220100
N	-2.38083300	-2.81165400	1.12656700
H	-1.04407000	-2.56568100	-2.02805100
H	-2.66785200	-1.81882500	-1.91088000
H	-1.32181800	-0.92052000	-2.65640600

C	-2.00432400	2.62714700	-1.01285400
H	-0.85903100	1.11445800	-2.02958900
C	-3.13258500	1.93258800	0.99861200
H	-2.87537600	-0.13090000	1.56505300
C	-2.82205300	2.92995700	0.07768200
H	-1.74947100	3.40113200	-1.73853900
H	-3.76753700	2.15701200	1.85718400
H	-3.21200400	3.94059900	0.20836200

Ph-6-123456-R4-Nu2

C	1.28649000	0.27843200	-0.48043700
C	1.92767000	-1.03298500	-0.47270200
C	2.06753700	1.16806500	0.37855800
C	3.21593700	-1.26872400	-0.04255800
C	3.34964900	0.92830700	0.82074700
C	3.96324700	-0.30987600	0.64084900
F	1.49334200	2.35302400	0.70970400
F	3.98748800	1.88150200	1.50949300
F	5.23213800	-0.54304700	1.03308400
F	3.75707700	-2.47097900	-0.26612800
F	1.31987400	-2.01726800	-1.17550400
F	1.23034000	0.75231600	-1.87801000
C	-0.22076000	0.34958200	-0.03733800
Br	-1.18653000	1.84388600	-0.82655500
S	-1.22066000	-1.17177800	-0.28232500
H	-0.26179300	0.50445700	1.04693400
O	-1.37799200	-1.43769300	-1.70636000
O	-0.67005600	-2.17247600	0.62911300
C	-2.82551400	-0.73553900	0.36963300
C	-3.85290400	-0.41394000	-0.51407700
C	-3.02087400	-0.78397700	1.74859700
C	-5.11030100	-0.11381900	0.00557300
H	-3.65664200	-0.40330000	-1.58673500
C	-4.28127700	-0.47264700	2.25589300
H	-2.20157200	-1.07493600	2.40784700
C	-5.32040300	-0.13801200	1.38590600
H	-5.92904600	0.13962500	-0.66873900
H	-4.45447300	-0.50169800	3.33216300
H	-6.30631400	0.10052000	1.78751800

Ph-6-123456-R4-Nu4

C	-0.55409500	0.01549900	0.88879900
C	-2.08356100	-0.00530900	0.72311500
C	0.09108200	1.19881900	0.34034800
C	-2.56277800	0.00000300	-0.70869400
H	-2.44825900	-0.90015700	1.24846300
H	-2.46452100	0.87658100	1.25328600
C	0.15012500	-1.16227100	0.41012800
C	1.37256700	1.22279100	-0.15947200
C	-2.49871200	-1.30132800	-1.46170200
C	-3.02294600	1.12086600	-1.27438100

C	1.43301400	-1.15365300	-0.09017800
C	2.08510700	0.04297500	-0.39618300
H	-3.03973000	-2.08991200	-0.91561600
H	-2.93249100	-1.20276900	-2.46565100
H	-1.45830100	-1.64519900	-1.55849900
H	-3.05076700	2.06061600	-0.71820200
H	-3.36936200	1.13252800	-2.31113400
F	-0.48015700	-2.36172300	0.54309300
F	2.04918800	-2.31655500	-0.35201300
F	3.38388300	0.06628600	-0.77293200
F	1.93475600	2.39589900	-0.48828800
F	-0.59470800	2.37034600	0.41250100
F	-0.48133800	0.05663200	2.41727000

Ph-6-123456-R5

C	0.00000000	1.38823200	0.00000000
C	1.20196500	0.69407300	-0.00000600
C	-1.20196500	0.69407300	0.00000600
N	2.48842900	1.43629800	-0.00170100
C	1.20196500	-0.69407300	0.00000600
N	0.00000000	2.87350500	0.00000000
N	-2.48842900	1.43629800	0.00170000
C	-1.20196500	-0.69407300	-0.00000600
O	3.27774800	1.11465600	0.84050600
O	2.60272000	2.27971800	-0.84516900
C	0.00000000	-1.38823200	0.00000000
N	2.48842900	-1.43629800	0.00170000
O	0.67652300	3.39529900	0.84012700
O	-0.67652500	3.39529900	-0.84012500
O	-3.27774800	1.11465600	-0.84050600
O	-2.60272000	2.27971800	0.84516800
N	-2.48842900	-1.43629800	-0.00170100
N	0.00000000	-2.87350500	0.00000000
O	3.27774800	-1.11465600	-0.84050600
O	2.60272000	-2.27971800	0.84516800
O	-3.27774800	-1.11465600	0.84050600
O	-2.60272000	-2.27971800	-0.84516900
O	0.67652500	-3.39529900	-0.84012500
O	-0.67652300	-3.39529900	0.84012700

Ph-6-123456-R5-Nu1

C	0.83108200	0.05121200	-0.35859000
C	-0.08848700	1.26478200	-0.55108000
C	-0.04983900	-1.15898800	-0.60850000
C	-1.39691200	1.20996900	-0.16215100
C	-1.35549500	-1.18463900	-0.22063600
C	-2.04372700	-0.01405800	0.14041500
N	-2.09745600	-2.47494100	-0.20497400
O	-1.67636100	-3.32390000	0.53530500
O	-3.05487200	-2.55163400	-0.92057200
N	-3.24720700	-0.08709500	0.91200000

O	-3.48243400	-1.13826700	1.47704200
O	-3.96224000	0.89012000	0.96838500
N	-2.20761700	2.45037500	-0.01360500
O	-2.88582100	2.76883900	-0.94750800
O	-2.12711100	2.99356200	1.05302800
N	0.44060700	2.51566000	-1.08993400
O	-0.18303700	3.52947600	-0.88674900
O	1.46139000	2.46827800	-1.73213700
N	1.88439500	0.04719900	-1.51223200
O	1.45602900	-0.16318100	-2.61397900
O	3.02648000	0.24666000	-1.21755600
N	0.53026700	-2.33695900	-1.22017100
O	1.72905200	-2.48824600	-1.10156900
O	-0.20178900	-3.09721000	-1.80800900
C	1.58643500	0.02792000	1.03407600
C	2.48128500	1.29074100	1.18563000
C	2.35635000	-1.31350700	1.24041000
C	0.55165500	0.18146100	2.17320000
O	1.96665400	2.36227900	0.96726300
C	3.88275100	1.21347300	1.72203400
O	3.53586400	-1.39892500	1.02172500
C	1.60589800	-2.48508600	1.83469500
H	0.04500300	1.15109700	2.09454600
H	-0.19552900	-0.62234500	2.17044800
H	1.07275300	0.15163100	3.14095500
H	4.20253500	2.23324500	1.96167000
H	3.93342600	0.57104600	2.61104200
H	4.53468800	0.76281900	0.96486500
H	1.53042900	-2.33171400	2.92247400
H	0.58371100	-2.60674800	1.45517700
H	2.19165900	-3.39121000	1.64686600

Ph-6-123456-R5-Nu3

C	-1.35975500	0.18186500	-0.03376200
C	-0.72335400	-0.95704300	-0.81981000
C	-0.56902400	1.41761400	-0.43482300
C	0.61804300	-1.00129100	-1.04768000
C	0.78317500	1.34807100	-0.63335500
C	1.44715100	0.11902800	-0.80801100
N	1.58645800	2.60204000	-0.68116500
O	1.64261400	3.23167400	0.34067300
O	2.10915700	2.86410800	-1.72586500
N	2.84351800	-0.03139200	-0.52704700
O	3.46679500	0.95541400	-0.19405900
O	3.32832900	-1.13972000	-0.62487500
N	1.23951000	-2.24264000	-1.58748200
O	1.71255500	-2.16162500	-2.68527300
O	1.21457800	-3.20861400	-0.87490200
N	-1.56794100	-2.03860400	-1.29724100
O	-1.17293200	-2.70489400	-2.22466300
O	-2.62848900	-2.19782200	-0.73502100
N	-2.79760700	0.34802700	-0.61744000

O	-2.84527600	0.51450800	-1.80810500
O	-3.72924500	0.28905900	0.12721100
N	-1.25317500	2.68495400	-0.59312000
O	-2.36913100	2.78646600	-0.12743000
O	-0.68362900	3.56812300	-1.19237600
C	-1.40049600	-0.02638100	1.54436500
C	0.02916900	-0.38015800	2.00156300
C	-2.28089600	-1.15177500	1.92332300
C	-1.92101300	1.21752600	2.29393400
C	0.92537200	0.62527900	2.38113400
C	0.47793000	-1.70530800	1.95899200
N	-2.93302000	-2.00822800	2.33583200
H	-2.97213300	1.40442300	2.05922600
H	-1.80531400	1.04070500	3.37092900
H	-1.34346300	2.10840100	2.02567900
C	2.25317400	0.31412500	2.67426900
H	0.61607700	1.66902900	2.41286900
C	1.80485700	-2.01524500	2.24793700
H	-0.20630500	-2.50512900	1.66882300
C	2.69874800	-1.00460900	2.60111100
H	2.94319200	1.11442100	2.94406900
H	2.14083700	-3.05057200	2.18208600
H	3.74179900	-1.24398900	2.81230700

Ph-6-123456-R5-Nu2

C	0.50781200	-0.63283600	0.89416500
C	1.10854700	0.71333900	1.26760200
C	1.54175200	-1.29213400	-0.01051900
C	1.74446900	1.45122400	0.31883400
C	2.16188700	-0.53776900	-0.96474300
C	2.13675200	0.87150500	-0.91468400
N	2.93814300	-1.19531000	-2.05390800
O	2.30470000	-1.84178000	-2.84109600
O	4.12185000	-1.00979700	-2.04703700
N	2.38965200	1.66933100	-2.07569000
O	2.59066200	1.09440900	-3.12568100
O	2.37185400	2.87631500	-1.95126800
N	2.11148100	2.87042800	0.58436900
O	3.28016400	3.08212000	0.74913200
O	1.21582900	3.66552100	0.60003000
N	0.84327500	1.26297500	2.58344100
O	1.52786200	2.18322200	2.96161000
O	-0.05114100	0.75624000	3.22142500
N	0.38738900	-1.47669000	2.18236400
O	1.41460400	-1.61216200	2.79419500
O	-0.68351200	-1.91681700	2.46276000
N	1.82249800	-2.71199300	0.09397000
O	1.02921500	-3.40612500	0.68726500
O	2.84354200	-3.11766500	-0.41359300
C	-0.84160300	-0.47068900	0.14730700
Br	-1.61289600	-2.11336000	-0.49605400
S	-2.13897800	0.60475600	0.92663600

H	-0.59354400	0.10747800	-0.75527900
O	-2.80554800	-0.07920000	2.01899100
O	-1.48148000	1.89475800	1.12170900
C	-3.29585400	0.79650800	-0.41017400
C	-4.45743000	0.02704500	-0.41174900
C	-3.02349200	1.74501200	-1.39524600
C	-5.36776000	0.20472300	-1.45093300
H	-4.63503700	-0.69021300	0.39037100
C	-3.94139800	1.90601100	-2.43112500
H	-2.11886800	2.35324600	-1.33948400
C	-5.10629400	1.13675300	-2.45735400
H	-6.28443200	-0.38514900	-1.47438400
H	-3.75082000	2.64069800	-3.21380700
H	-5.82247200	1.26979300	-3.26934300

Ph-6-123456-R5-Nu4

C	-0.29691500	-1.33109900	0.19025800
C	-0.41137000	-1.63557600	1.69490900
C	1.07996300	-0.86856800	-0.24329100
C	-0.28586100	-0.37980900	2.53604400
H	-1.38042800	-2.12083600	1.86464600
H	0.38021000	-2.35598000	1.93672500
C	-1.27019300	-0.29358600	-0.33465000
C	1.39662500	0.45071600	-0.34235600
C	-1.57300000	0.27613100	2.95723400
C	0.91364000	0.09810500	2.88356100
C	-0.93314900	1.01824100	-0.45332400
C	0.40657800	1.46072000	-0.33505800
H	-2.17839400	-0.42372000	3.55432500
H	-1.38369200	1.17611100	3.55642400
H	-2.18414600	0.56785200	2.08886900
H	1.83866100	-0.39891700	2.57932200
H	1.00646000	1.00965200	3.47905200
N	-1.99347700	2.02445800	-0.73567500
O	-2.80278100	2.21146000	0.13258100
O	-1.94436600	2.55783200	-1.80685700
N	0.70639000	2.78401200	0.10155500
O	-0.20843100	3.58024800	0.18807200
O	1.86092000	3.04764500	0.37667100
N	2.82173500	0.85816600	-0.48435000
O	3.12503400	1.36957400	-1.52498600
O	3.53753900	0.64170300	0.45497500
N	2.09996900	-1.87845900	-0.43295800
O	3.06637200	-1.60476600	-1.10593000
O	1.91055800	-2.95635300	0.09269500
N	-0.59248200	-2.64349100	-0.61071700
O	-0.53528000	-2.53956200	-1.81127800
O	-0.85117400	-3.63836600	-0.00216700
N	-2.62754800	-0.72662400	-0.59403500
O	-2.99692800	-1.73119100	-0.02087000
O	-3.30630000	-0.08225500	-1.35866500

Py-3-246-R1

C	0.71042500	-1.10565700	-0.00129200
C	-0.67666500	-1.25401000	-0.00181300
C	-1.43864600	-0.09255700	-0.00135800
C	0.58181700	1.15993100	-0.00045900
C	-0.80963300	1.15072900	-0.00098900
C	-2.94912000	-0.14919900	0.00001700
C	1.36200000	2.46020300	0.00006600
C	1.63001700	-2.31188000	-0.00006400
F	2.40552300	-2.31624500	1.07970300
F	0.93480500	-3.44774300	-0.01120500
F	2.42317700	-2.30560200	-1.06665100
F	0.54464700	3.51179400	-0.00003900
F	2.14100100	2.54629400	1.07366200
F	2.14181300	2.54671500	-1.07289000
F	-3.44615200	0.47441900	-1.06621500
F	-3.39478600	-1.40098800	-0.01089000
F	-3.44325500	0.45418000	1.07922500
N	1.32765100	0.06398300	-0.00044400
H	-1.13847300	-2.24025600	-0.00262900
H	-1.37722100	2.08161400	-0.00122700

Py-3-246-R1-Nu1

C	0.56439700	-0.06969600	0.40385900
C	-0.37264000	-1.25236100	0.37367800
C	-1.66771800	-1.07845200	0.01932200
C	-1.36186800	1.25705500	0.03913700
C	-2.22673000	0.20993400	-0.23742900
C	-2.56922600	-2.26906600	-0.11423800
C	-1.87110400	2.67832800	-0.06134900
C	1.35643300	-0.14763700	1.72043500
F	0.52440100	-0.25866500	2.76087200
F	2.15549500	-1.22951600	1.75955900
F	2.11736600	0.91708300	1.95586200
F	-3.16281000	2.74551700	-0.40338700
F	-1.73796300	3.34733300	1.08737900
F	-1.19447300	3.37458600	-0.99030700
F	-2.93366400	-2.45923500	-1.39217500
F	-2.01017100	-3.40898400	0.30032300
F	-3.70693000	-2.11954800	0.57916500
N	-0.09908600	1.19809200	0.40482400
H	0.01226800	-2.24005300	0.61978900
H	-3.26623300	0.35698500	-0.51447200
C	1.63119200	-0.13970700	-0.88023200
C	2.28170100	-1.53545700	-0.95751800
C	2.63464200	1.01379300	-0.77750900
C	0.75033300	0.00961700	-2.12871700
O	1.58902900	-2.46229500	-1.32430300
C	3.74186400	-1.79127400	-0.66004200
O	3.70934700	0.88409800	-0.23200100
C	2.23958300	2.34589100	-1.36575000
H	0.04004000	-0.82333300	-2.16894900

H	0.19330700	0.95297700	-2.10079600
H	1.36450200	-0.01273500	-3.04173800
H	3.91256100	-2.86811200	-0.77491700
H	4.36763100	-1.23297000	-1.36998600
H	4.02126100	-1.44708800	0.33923900
H	2.15371300	2.27449100	-2.45951500
H	1.25608200	2.63101600	-0.96663500
H	3.00504900	3.08478600	-1.10285100

Py-3-246-R1-Nu3

C	-0.11463900	-0.45785200	0.42366200
C	0.07223700	1.03385800	0.55962900
C	1.24651400	1.59339300	0.16839000
C	2.19117800	-0.55320200	-0.07507700
C	2.36359700	0.81904900	-0.24788500
C	1.35842200	3.08852300	0.11936300
C	3.35983200	-1.47655200	-0.34974600
C	-0.80744100	-0.96027800	1.70782700
F	0.03441900	-0.91857900	2.74049700
F	-1.86223700	-0.20515400	2.05006800
F	-1.25220900	-2.21747400	1.61533200
F	4.49977200	-0.81423800	-0.58322300
F	3.59638000	-2.31290400	0.66280400
F	3.13168600	-2.23401700	-1.43279100
F	1.17902000	3.53833000	-1.13523300
F	0.45212900	3.71196300	0.87833100
F	2.56172900	3.52687800	0.50651800
N	1.11420800	-1.19673800	0.30212200
H	-0.74272600	1.64279600	0.94752100
H	3.31170800	1.26183400	-0.53623100
C	-1.04585700	-0.81241400	-0.86235800
C	-2.46066900	-0.25557800	-0.73995500
C	-1.09346900	-2.27646000	-1.03864400
C	-0.32680600	-0.27967100	-2.11784800
C	-2.71139900	1.08964400	-1.04201500
C	-3.52480600	-1.04862200	-0.29150000
N	-1.14030800	-3.40853400	-1.25904000
H	0.62479400	-0.80954300	-2.24936400
H	-0.95478300	-0.43965300	-3.00475700
H	-0.11383400	0.79097100	-2.01235000
C	-3.98857600	1.62858400	-0.88470800
H	-1.90441500	1.72925000	-1.39950600
C	-4.80201500	-0.51236000	-0.14184400
H	-3.34878800	-2.09839700	-0.04973000
C	-5.03920700	0.83080500	-0.43459000
H	-4.16147700	2.67937100	-1.12277200
H	-5.61634700	-1.14984400	0.20622700
H	-6.03899200	1.25168400	-0.31755600

Py-3-246-R1-Nu2

C	0.88976500	-0.52127100	0.94685100

C	1.18053900	0.91244300	1.29997500
C	1.62248200	1.68550000	0.27478900
C	2.27929300	-0.26419300	-0.88031200
C	2.03576300	1.10936200	-0.96485600
C	1.69669100	3.17232700	0.43528200
C	3.16044900	-0.92928400	-1.91442500
C	0.84231600	-1.38633400	2.20483300
F	2.02070900	-1.38125800	2.82660300
F	-0.06051800	-0.92626700	3.08181600
F	0.53005000	-2.65445700	1.95055700
F	3.47082700	-0.10843400	-2.92576000
F	4.31542800	-1.34835200	-1.38514800
F	2.57489200	-2.00211500	-2.45641600
F	0.70408200	3.77950800	-0.24037300
F	1.59159900	3.56713700	1.70640900
F	2.83727500	3.68638100	-0.04209200
N	1.90748600	-1.06894100	0.08883200
H	0.94139200	1.30740900	2.28472300
H	2.40846800	1.71375300	-1.78720300
C	-0.48657100	-0.53957600	0.17034800
Br	-0.87174900	-2.17891500	-0.78891700
S	-1.98395300	-0.04250600	1.09588800
H	-0.37266500	0.22466500	-0.61094200
O	-2.57876500	-1.21059500	1.73632800
O	-1.65727600	1.13567800	1.89556700
C	-3.06729300	0.49551500	-0.21417200
C	-4.11015500	-0.33091700	-0.62184300
C	-2.84164800	1.74939500	-0.78260600
C	-4.95311900	0.11492800	-1.63944500
H	-4.24937900	-1.29990100	-0.14091300
C	-3.68733600	2.17802800	-1.80292800
H	-2.01843600	2.37405300	-0.42821700
C	-4.73945200	1.36251000	-2.22721000
H	-5.77874100	-0.51422000	-1.97385400
H	-3.52904600	3.15332900	-2.26428200
H	-5.40064200	1.70509100	-3.02445100

Py-3-246-R1-Nu4

C	0.45380800	1.21264000	-0.01938100
C	0.65376700	1.75293500	1.43753200
C	-1.00795400	0.94560100	-0.30801600
C	0.26216100	0.77117700	2.51275800
H	1.71767700	2.01875500	1.54503800
H	0.05551500	2.67214200	1.53408200
C	-1.45688700	-0.33157000	-0.28854200
C	1.34679000	-0.14864700	3.00519400
C	-0.98107700	0.74102000	3.00614400
C	0.77351600	-1.11130800	-0.27345700
C	-0.57555300	-1.44940900	-0.17981800
H	2.11770500	0.43168700	3.53902200
H	0.95258000	-0.91248200	3.68922700
H	1.84916100	-0.63704800	2.15883600

H	-1.74810000	1.43108100	2.64598200
H	-1.27217400	0.01302300	3.76787600
C	-2.92480300	-0.60987600	-0.38885700
C	1.78682700	-2.22390700	-0.45045400
C	0.92806600	2.33651400	-0.94932000
F	0.82274000	1.99810300	-2.23581800
F	0.20093900	3.45861400	-0.78893800
F	2.20302700	2.68058000	-0.73936500
F	1.23997500	-3.44435600	-0.37447400
F	2.40475500	-2.15431700	-1.63589700
F	2.75178000	-2.18465500	0.48128700
F	-3.40073300	-1.13370400	0.75323200
F	-3.65887100	0.47950200	-0.64264300
F	-3.21151400	-1.49966300	-1.35110600
N	1.31729400	0.08014300	-0.29266600
H	-1.68620100	1.78613700	-0.45866800
H	-0.92806000	-2.47626400	-0.19193700

Py-3-246-R2

C	0.00000000	1.33036800	0.00000000
C	-1.23001000	0.66398900	0.00000000
C	-1.20510600	-0.73210700	0.00000000
C	1.18069800	-0.61368200	0.00000000
C	0.02227800	-1.39778800	0.00000000
C	-2.43893000	-1.48109500	0.00000000
N	-3.42478600	-2.07915100	0.00000000
C	2.48175900	-1.25930000	0.00000000
N	3.50783600	-1.78470600	0.00000000
C	0.02660700	2.78266000	0.00000000
N	0.03328400	3.93541600	0.00000000
N	1.17839200	0.71550400	0.00000000
H	-2.16798400	1.21776700	0.00000000
H	0.08111900	-2.48547000	0.00000000

Py-3-246-R2-Nu1

C	-0.00872200	-0.11378200	0.15629300
C	-0.87695400	-1.26169100	-0.31034300
C	-2.21911700	-1.12011700	-0.11017500
C	-1.92610700	1.21742200	0.07233600
C	-2.78505800	0.13586600	0.28203800
C	-3.09923400	-2.22252300	-0.41570100
N	-3.81024400	-3.10228500	-0.65397800
C	-2.52476000	2.54416000	-0.03221900
N	-3.00551300	3.59181200	-0.11019000
C	0.12054500	-0.21127700	1.65957100
N	0.09822700	-0.25876100	2.81313800
N	-0.62658000	1.16338200	-0.16067300
H	-0.43678300	-2.16914800	-0.71658300
H	-3.85137900	0.26555200	0.44462100
C	1.42052100	-0.11996900	-0.48548100
C	2.24846200	-1.32323700	-0.01035200

C	2.13250300	1.16430000	-0.00860800
C	1.29370800	-0.18836200	-2.01298500
O	1.73670400	-2.26650400	0.54229300
C	3.73533500	-1.29194100	-0.28107700
O	2.63637200	1.16915000	1.09136900
C	2.17356800	2.36337700	-0.91391800
H	0.86195100	-1.14899700	-2.32424300
H	0.62513800	0.61012400	-2.35836200
H	2.26973200	-0.07567500	-2.50408700
H	4.10192200	-2.31542100	-0.42069300
H	3.99846900	-0.66494700	-1.14243800
H	4.21830700	-0.86114600	0.60786000
H	2.73765100	2.13528800	-1.82972900
H	1.14278600	2.61449600	-1.19635200
H	2.64823200	3.19632100	-0.38364300

Py-3-246-R2-Nu3

C	0.47466400	-0.29964300	0.05662900
C	0.63082400	1.17342400	-0.22493400
C	1.83578400	1.71536900	0.12522500
C	2.79101000	-0.44603800	0.07325800
C	2.94388900	0.88584100	0.48046700
C	2.03107200	3.14143900	0.01485600
N	2.19249900	4.28309600	-0.06494900
C	4.00158500	-1.24519900	-0.08628000
N	4.96575700	-1.87025600	-0.20375500
C	0.22971800	-0.48499100	1.53752500
N	0.09656900	-0.61819700	2.67700400
N	1.67040900	-1.03228500	-0.29994100
H	-0.18666300	1.76714700	-0.63063100
H	3.91052500	1.29837300	0.75538600
C	-0.74610900	-0.92831700	-0.72703300
C	-2.07192500	-0.25812700	-0.34703900
C	-0.81153500	-2.35897600	-0.35941600
C	-0.44997100	-0.85817800	-2.23339300
C	-2.60854500	0.76916900	-1.13291000
C	-2.76346400	-0.64595700	0.80814800
N	-0.88976700	-3.47738300	-0.08759600
H	0.45150100	-1.44137600	-2.44953600
H	-1.30046800	-1.25125500	-2.80589500
H	-0.25734100	0.17898700	-2.53497500
C	-3.79317400	1.40565800	-0.76253100
H	-2.10790000	1.08331400	-2.04874200
C	-3.94845300	-0.01186600	1.17737800
H	-2.37412900	-1.45678600	1.42607300
C	-4.46658400	1.01933100	0.39483900
H	-4.19165600	2.20506300	-1.38888200
H	-4.46992300	-0.33095700	2.08084000
H	-5.39491500	1.51502100	0.68229800

Py-3-246-R2-Nu2

C	1.33722400	-0.05785100	0.10664000
C	1.93877500	1.29816500	-0.18721200
C	3.29953500	1.35485200	-0.05894600
C	3.40062700	-0.97716200	-0.39450700
C	4.08176500	0.16489100	0.04674400
C	3.96953800	2.62790300	-0.17389700
N	4.51412400	3.64382000	-0.25836700
C	4.19819900	-2.10737300	-0.85553200
N	4.83851000	-3.00082400	-1.21062600
C	1.38667400	-0.30865700	1.59907900
N	1.53809100	-0.52497100	2.72262800
N	2.09659800	-1.09470200	-0.56343000
H	1.32993400	2.15810500	-0.45484800
H	5.16302800	0.18957800	0.15014200
C	-0.12116100	-0.20162300	-0.39137500
Br	-0.87860300	-1.89673200	0.13906900
S	-1.18974700	1.19605300	0.13552800
H	-0.10866100	-0.17132100	-1.48717400
O	-0.98844300	1.40097400	1.56555300
O	-0.91453900	2.28013100	-0.80833700
C	-2.86683200	0.67463300	-0.13377200
C	-3.60785500	0.20527000	0.94855700
C	-3.40219500	0.78819300	-1.41510200
C	-4.92974000	-0.17582200	0.73066800
H	-3.14785700	0.14506700	1.93555000
C	-4.72400100	0.39652300	-1.61831000
H	-2.79644400	1.18616300	-2.23038300
C	-5.48175900	-0.08421000	-0.54883400
H	-5.53079700	-0.54525600	1.56204900
H	-5.16539500	0.47448700	-2.61226800
H	-6.51692500	-0.38653000	-0.71329700

Py-3-246-R2-Nu4

C	-0.52793500	-0.11272100	0.74559300
C	-2.06429100	-0.28653100	0.80889700
C	-0.11752200	1.19269700	0.11358400
C	-2.77338800	-0.11472100	-0.51307400
H	-2.25184900	-1.29646700	1.20709500
H	-2.46129300	0.44334300	1.52938600
C	1.21560100	1.31532100	-0.16289500
C	-2.57610600	-1.18149300	-1.55420800
C	-3.56153000	0.94562000	-0.72564900
C	1.38396100	-1.04500800	-0.16426800
C	2.07751500	0.17431900	-0.10202200
H	-2.82189300	-2.17161900	-1.13922200
H	-3.20477000	-0.99750700	-2.43571500
H	-1.52060300	-1.22931000	-1.85420200
H	-3.70313800	1.71137700	0.04154100
H	-4.08940600	1.07790500	-1.67341600
C	1.73561000	2.56679000	-0.65821900
N	2.16042000	3.56961900	-1.04646800
C	2.11357400	-2.20863700	-0.65689600

N	2.70033600	-3.12728500	-1.04014800
C	-0.05845200	-0.12363900	2.18577900
N	0.28144600	-0.13679400	3.28990100
N	0.09789600	-1.23149000	0.05995700
H	-0.85038800	1.97001500	-0.09982100
H	3.14042600	0.24192700	-0.31806900

Py-3-246-R3

C	0.74773700	-1.11686600	0.00076500
C	1.23801600	0.19370900	0.00046000
C	1.68503800	-2.30050600	0.00411800
C	-1.42230000	-0.41181700	-0.00211200
C	0.31288500	1.23485700	-0.00037300
O	2.95892700	-1.91483200	-0.00579100
O	1.32891300	-3.44541100	0.01478100
C	-1.04677000	0.93418800	-0.00127000
C	-2.87583800	-0.81773400	-0.00479700
C	0.72510700	2.67837700	-0.00019600
C	3.92864600	-2.95772000	-0.00334100
O	-3.68011400	0.24223300	0.00714800
O	-3.25987900	-1.95348300	-0.01630000
O	2.04285800	2.82599800	0.00022000
O	-0.05843000	3.59065700	-0.00048100
H	4.90601100	-2.46782800	-0.01079800
H	3.81645800	-3.57877400	0.89464500
H	3.80863500	-3.59006100	-0.89240700
C	-5.07726400	-0.03375400	0.00633000
C	2.52525100	4.16760200	0.00030500
H	-5.35204300	-0.59967100	-0.89307500
H	-5.34897500	-0.61980700	0.89369000
H	-5.58157600	0.93613400	0.01814600
H	2.17175300	4.69659600	0.89443300
H	2.17146900	4.69682100	-0.89357300
H	3.61617400	4.09988900	0.00013200
N	-0.54675900	-1.41064300	-0.00097200
H	2.30860100	0.38816100	0.00123600
H	-1.79043300	1.72973000	-0.00159200

Py-3-246-R3-Nu1

C	-0.58513300	0.04476300	0.28471500
C	0.24602200	1.29212800	0.28305600
C	-1.61170000	0.17397700	1.41956900
C	1.44684700	-1.15227600	0.11187500
C	1.59418200	1.21770900	0.08579100
O	-1.66888600	-0.87546300	2.22750200
O	-2.27808600	1.16904800	1.58900700
C	2.25716600	-0.03209000	-0.06725100
C	2.06378100	-2.52533000	0.00246600
C	2.42848200	2.45628300	0.06071000
C	-2.68382300	-0.83332100	3.21718600
O	3.39330900	-2.51693600	0.17024400

O	1.45693800	-3.53957600	-0.23779800
O	1.73982400	3.57261100	0.32836400
O	3.61302500	2.47621100	-0.17421700
H	-2.58371900	-1.75195500	3.80335300
H	-2.56540700	0.04699800	3.86253500
H	-3.67103700	-0.79478500	2.73626600
C	4.04716500	-3.76534000	0.01376500
C	2.48226800	4.78036600	0.31706600
H	3.67853600	-4.49119300	0.75121200
H	3.87351300	-4.17142400	-0.99223700
H	5.11430600	-3.57815100	0.16779900
H	2.93186600	4.94997100	-0.67062400
H	3.28490400	4.74880900	1.06636600
H	1.77439700	5.58062300	0.55318800
N	0.15106000	-1.16712000	0.35683100
H	-0.24865400	2.24606700	0.44835000
H	3.32721800	-0.09638900	-0.23473700
C	-1.55809600	-0.04515200	-1.12886300
C	-2.36432900	1.23955900	-1.27879200
C	-2.38511500	-1.29582600	-0.91385500
C	-0.57034800	-0.13806700	-2.28756100
O	-1.79184500	2.23589100	-1.67742700
C	-3.85149900	1.29316900	-1.00837600
O	-3.31700900	-1.29740900	-0.13036200
C	-1.96111300	-2.56747500	-1.60031800
H	0.06190900	0.75767900	-2.28934700
H	0.06821800	-1.02331800	-2.18370300
H	-1.09423900	-0.19109100	-3.25459500
H	-4.19862500	2.30267000	-1.25848400
H	-4.37850400	0.54945500	-1.62263600
H	-4.06261900	1.05693200	0.03934200
H	-1.85786300	-2.43683600	-2.68569900
H	-0.97140500	-2.83028300	-1.18883100
H	-2.68691500	-3.35740200	-1.37493700

Py-3-246-R3-Nu3

C	-0.10373100	-0.58372000	0.34049000
C	-0.07767100	0.91912400	0.46517400
C	-0.80456400	-1.20752300	1.56131800
C	2.22850900	-0.43711400	-0.06182600
C	1.02433800	1.60838900	0.05226300
O	-0.50449100	-0.55737100	2.68685600
O	-1.49309500	-2.19613000	1.55467600
C	2.21801100	0.93306000	-0.33309000
C	3.54713600	-1.16911900	-0.10757200
C	1.04719000	3.10028300	0.08722200
C	-1.02465200	-1.11514300	3.88436400
O	4.45076100	-0.54977900	-0.88458700
O	3.80115300	-2.19342500	0.47165400
O	-0.13267300	3.64452400	0.41999800
O	2.01166900	3.77963300	-0.16999300
H	-0.69700100	-0.46112000	4.69800400

H	-2.12137300	-1.15850400	3.84667100
H	-0.63747900	-2.13197300	4.03343500
C	5.72731100	-1.16146900	-0.95385600
C	-0.16536800	5.06040600	0.49085700
H	6.18925000	-1.21185000	0.04169400
H	5.64755600	-2.18233500	-1.35181100
H	6.33375200	-0.54105800	-1.62102600
H	0.09545400	5.50326600	-0.47975200
H	0.54459100	5.42763300	1.24435600
H	-1.18772400	5.33300800	0.76989600
N	1.21425400	-1.18146100	0.33537900
H	-0.92861100	1.43861400	0.90191700
H	3.10787600	1.47701100	-0.63178600
C	-0.92093400	-1.05249900	-0.97702400
C	-2.36936500	-0.56999100	-0.94215600
C	-0.86715800	-2.52287200	-1.08156300
C	-0.18958200	-0.53065900	-2.22899900
C	-2.66609600	0.76457900	-1.25800700
C	-3.42790100	-1.41578300	-0.58448200
N	-0.80798800	-3.66014000	-1.27331700
H	0.80600100	-0.98598500	-2.29508700
H	-0.76232800	-0.78301500	-3.13227200
H	-0.06452800	0.55678400	-2.17151100
C	-3.97754900	1.23835900	-1.20700200
H	-1.86589100	1.44906500	-1.53813000
C	-4.73971100	-0.94573500	-0.54463700
H	-3.21529900	-2.45322400	-0.32557400
C	-5.02132300	0.38520600	-0.85300900
H	-4.18154800	2.28184400	-1.45317500
H	-5.54657900	-1.62703700	-0.26926300
H	-6.04791500	0.75379300	-0.82064600

Py-3-246-R3-Nu2

C	0.84369700	-0.52766700	0.85999600
C	0.11837700	0.66212500	1.43069900
C	0.99455300	-1.67085000	1.86225900
C	2.21826200	0.97015200	-0.25905800
C	0.21875800	1.81609800	0.70312700
O	1.42893300	-1.23576900	3.04189000
O	0.78312700	-2.83171900	1.62538000
C	1.19043500	1.92565300	-0.33327600
C	3.55452300	1.27351900	-0.88445200
C	-0.59874300	3.01627000	1.02960700
C	1.64276200	-2.23322700	4.03056800
O	3.48432900	2.26564000	-1.78578600
O	4.58860600	0.70965600	-0.63487500
O	-1.51181900	2.78834500	1.98879700
O	-0.49633600	4.09248600	0.49229700
H	1.97675000	-1.70906400	4.93087500
H	0.71247400	-2.78160900	4.22917200
H	2.40901400	-2.94585600	3.69767900
C	4.70501000	2.61478700	-2.41594000

C	-2.30761800	3.90070100	2.36593800
H	5.43986200	2.95975600	-1.67564100
H	5.12691100	1.75215500	-2.94935500
H	4.47202300	3.41912300	-3.12043700
H	-2.87595000	4.28057200	1.50609300
H	-1.67834100	4.71290600	2.75419900
H	-2.98869200	3.54411600	3.14456600
N	2.17853300	-0.14690600	0.44118000
H	-0.47705900	0.57705800	2.33583300
H	1.29134500	2.83780100	-0.91271300
C	0.01913400	-1.02565300	-0.36976400
Br	0.93459100	-2.26703100	-1.53579200
S	-1.64704600	-1.67131500	0.10817900
H	-0.17345500	-0.14518200	-0.99544400
O	-1.86258500	-2.99626300	-0.46354700
O	-1.83800400	-1.47637000	1.54715600
C	-2.73783500	-0.53613100	-0.73510600
C	-3.19216300	-0.87054900	-2.00832700
C	-3.08382700	0.66124000	-0.11074900
C	-4.01672300	0.03124200	-2.68054900
H	-2.90378100	-1.82396100	-2.45305200
C	-3.90759100	1.55263800	-0.79598000
H	-2.70214000	0.89019000	0.88627700
C	-4.37032200	1.23848600	-2.07603200
H	-4.38499400	-0.21119700	-3.67809200
H	-4.19200200	2.49599900	-0.32781400
H	-5.01379900	1.94159400	-2.60680700

Py-3-246-R3-Nu4

C	-0.64348900	-0.85809400	0.14860700
C	-1.02433300	-0.76640100	1.65872200
C	-2.35029500	-0.09870500	1.92635400
H	-1.02844800	-1.79581900	2.05273000
H	-0.22437000	-0.20648600	2.16347000
C	0.81147600	-1.26864100	0.01345700
C	-1.46058900	-1.98598300	-0.48509300
C	0.03782400	1.31934100	-0.27923600
C	-3.54171400	-0.62539300	1.17299900
C	-2.45409100	0.92181600	2.78458400
C	1.73405100	-0.27633300	0.19617700
O	-1.88996400	-1.72881400	-1.71965600
O	-1.65837200	-3.05156700	0.05130600
C	1.32342700	1.08783600	0.24683300
C	-0.30584700	2.69841900	-0.77717900
H	-3.39842800	-0.43715700	0.09620000
H	-3.64408000	-1.71338000	1.30424300
H	-4.47116000	-0.13428100	1.49069300
H	-1.57417100	1.31671500	3.29947400
H	-3.41666300	1.39737400	2.99123600
C	3.19167400	-0.57141100	0.25102700
C	-2.62376600	-2.76956700	-2.34456000
O	0.48672700	3.64972600	-0.25268300

O	-1.18719400	2.96737400	-1.55342300
O	3.48469500	-1.86393500	0.03283600
O	4.05530200	0.24308900	0.47812600
H	-2.89079400	-2.40527200	-3.34119100
H	-2.01749900	-3.68232100	-2.42065400
H	-3.53103400	-3.00047400	-1.76875400
C	0.23128300	4.97343100	-0.68634900
C	4.85932600	-2.20438000	0.07690000
H	0.35442100	5.05812300	-1.77490600
H	-0.79273100	5.27721700	-0.42894700
H	0.95536600	5.61453700	-0.17371900
H	5.28373100	-1.98392300	1.06594100
H	5.42499900	-1.64122600	-0.67790300
H	4.91978400	-3.27766300	-0.12877200
N	-0.87558300	0.40646800	-0.52116800
H	1.09501600	-2.30096000	-0.19076400
H	2.04596300	1.88712500	0.37829100

Py-3-246-R4

C	0.00000000	1.11922700	-0.69261000
C	0.00000000	1.21359400	0.69333900
C	0.00000000	0.00000000	1.37081600
C	0.00000000	-1.11922700	-0.69261000
C	0.00000000	-1.21359400	0.69333900
F	0.00000000	-2.23526200	-1.40104900
F	0.00000000	0.00000000	2.69144600
N	0.00000000	0.00000000	-1.38001000
H	0.00000000	2.17029600	1.21114700
H	0.00000000	-2.17029600	1.21114700
F	0.00000000	2.23526200	-1.40104900

Py-3-246-R4-Nu1

C	0.23987100	0.03744400	-0.21342600
C	0.96022700	-1.24473600	-0.04020400
C	2.32201900	-1.21696100	-0.05868700
C	2.27901900	1.10540900	0.07164900
C	3.07430100	-0.02827600	-0.07145200
F	2.92855800	2.27637900	0.24901500
F	3.00083900	-2.37839200	-0.01698500
F	-0.12347500	0.17153700	-1.65267700
N	0.98661400	1.18488200	0.11166300
H	0.43959200	-2.19880600	0.03489300
H	4.15982700	-0.00689400	-0.05224100
C	-1.16779800	0.05078200	0.45623500
C	-1.89145800	-1.28828600	0.20101400
C	-2.09060100	1.15539300	-0.08321500
C	-1.03051400	0.26930600	1.96445400
O	-2.22008500	-1.99138200	1.12835600
C	-2.14945900	-1.72004300	-1.22379800
O	-3.27406300	0.91491800	-0.20566700
C	-1.55099200	2.52725100	-0.38039700

H	-0.42936900	-0.54261000	2.39574000
H	-0.52237300	1.22197400	2.15677300
H	-2.01736900	0.26135200	2.44610800
H	-2.81552400	-2.59074100	-1.21141200
H	-2.58619900	-0.90321700	-1.80927000
H	-1.19415100	-1.97946200	-1.69874700
H	-0.97880100	2.91469600	0.47078800
H	-0.83803300	2.44764800	-1.21018700
H	-2.39169800	3.18418800	-0.63282200

Py-3-246-R4-Nu3

C	0.72200400	0.25156700	-0.27886700
C	0.81050000	-1.21708200	-0.12212700
C	2.04742400	-1.78523600	-0.13822700
C	3.03403100	0.32566200	-0.08106400
C	3.24686200	-1.04804800	-0.16088800
F	4.13616700	1.09698400	0.02824700
F	2.14988400	-3.12712400	-0.10160600
F	0.30004900	0.54049800	-1.64415100
N	1.91041300	0.97028100	-0.05471000
H	-0.08629000	-1.83402300	-0.09718300
H	4.23171300	-1.50460200	-0.14285900
C	-0.45210300	0.89502500	0.56023600
C	-1.78408500	0.20154000	0.26536500
C	-0.56137500	2.30952500	0.14981300
C	-0.07119300	0.86883800	2.04472500
C	-2.33825600	-0.71734400	1.16269900
C	-2.45836000	0.44868300	-0.93880800
N	-0.67587800	3.42332500	-0.13098700
H	0.83770400	1.46458000	2.18737600
H	-0.88101900	1.26887800	2.67010000
H	0.15046600	-0.16349600	2.34693800
C	-3.53088400	-1.37953800	0.86199700
H	-1.84184200	-0.93031500	2.10907700
C	-3.65033200	-0.20618400	-1.23661900
H	-2.03200100	1.15746800	-1.64906200
C	-4.19191300	-1.12668100	-0.33724100
H	-3.94354300	-2.09434200	1.57590200
H	-4.15998100	0.00367200	-2.17845700
H	-5.12579100	-1.64064900	-0.57033200

Py-3-246-R4-Nu2

C	1.61250500	0.05916800	0.31485600
C	2.02222000	1.44998000	0.00109200
C	3.30892000	1.66373700	-0.38434300
C	3.66240700	-0.62967700	-0.52704100
C	4.23436600	0.63176400	-0.63483400
F	4.44274600	-1.67621700	-0.86817900
F	3.72800000	2.92762000	-0.57920200
F	1.52986500	-0.09433100	1.76210500
N	2.45304000	-0.94564300	-0.17745800

H	1.33931800	2.27872800	0.16762400
H	5.25151100	0.80560500	-0.97243300
C	0.12956000	-0.19547200	-0.10524300
Br	-0.34226800	-2.06216300	-0.28306200
S	-1.07450700	0.56262200	1.06112700
H	-0.07043500	0.26881300	-1.07782100
O	-1.28663800	-0.29981700	2.21596900
O	-0.67712000	1.95713700	1.25511900
C	-2.58590800	0.57771600	0.10924100
C	-3.52859000	-0.42773800	0.31132000
C	-2.79320200	1.61621300	-0.79736900
C	-4.70755500	-0.39647700	-0.43189500
H	-3.33127300	-1.21249200	1.04243500
C	-3.97442900	1.63367500	-1.53634700
H	-2.04360800	2.40139600	-0.90901100
C	-4.92633200	0.62852300	-1.35400300
H	-5.45865900	-1.17416900	-0.28913200
H	-4.15540800	2.43761300	-2.25061700
H	-5.85044100	0.64710000	-1.93353100

Py-3-246-R4-Nu4

C	-0.22134400	-0.05086500	0.90000500
C	-1.74474100	-0.23730200	0.93482800
C	0.20173500	1.23922400	0.30012200
C	-2.44104200	-0.12485000	-0.39750300
H	-1.91744700	-1.23766800	1.36520500
H	-2.15800800	0.50812600	1.62940900
C	1.41700200	1.29209200	-0.30559000
C	-1.98732600	-1.04127100	-1.50124900
C	-3.43294700	0.75445400	-0.58239400
C	1.59031500	-1.03242500	-0.16648200
C	2.22403700	0.15580900	-0.52710400
H	-1.92996400	-2.08144500	-1.14681400
H	-2.66316700	-0.98595200	-2.36576800
H	-0.96959600	-0.77334600	-1.82183100
H	-3.75076100	1.42672200	0.21883600
H	-3.95601400	0.82821100	-1.53960200
F	2.23963600	-2.17525400	-0.49579000
F	1.87709900	2.47263200	-0.77056500
F	0.09402200	-0.00162700	2.33478400
N	0.43571700	-1.19990900	0.39374400
H	-0.41896300	2.13023200	0.39606000
H	3.18295000	0.19353400	-1.03535200

Py-3-246-R5

C	0.00000000	1.11626800	-0.66804900
C	0.00000000	1.21409500	0.71945200
C	0.00000000	0.00000000	1.38863400
C	0.00000000	-1.11626800	-0.66804900
C	0.00000000	-1.21409500	0.71945200
N	0.00000000	-2.37924600	-1.47225500

O	0.00000000	-2.27208300	-2.66521400
O	0.00000000	-3.40689000	-0.84097800
N	0.00000000	0.00000000	2.87301300
O	0.00000000	-1.07384800	3.41519300
O	0.00000000	1.07384800	3.41519300
N	0.00000000	2.37924600	-1.47225500
O	0.00000000	3.40689000	-0.84097800
O	0.00000000	2.27208300	-2.66521400
N	0.00000000	0.00000000	-1.35300100
H	0.00000000	-2.16997600	1.23940700
H	0.00000000	2.16997600	1.23940700

Py-3-246-R5-Nu1

C	0.29380000	-0.04163000	0.08703000
C	-0.51658300	-1.26031600	-0.16524000
C	-1.86942700	-1.13707500	-0.13011400
C	-1.67546200	1.16504000	-0.10745900
C	-2.54613300	0.09487700	0.00135300
N	-2.30678700	2.52181200	-0.27365300
O	-1.62553900	3.41936600	-0.69816600
O	-3.47286200	2.63144200	0.03235500
N	-2.68590700	-2.36014400	-0.28796700
O	-3.87856800	-2.25835600	-0.12089400
O	-2.12495200	-3.39298700	-0.57077200
N	0.57348900	0.01655600	1.72380000
O	-0.12396300	-0.65393000	2.43623500
O	1.37130000	0.83822700	2.11563500
N	-0.37565100	1.16123900	-0.20009000
H	-3.62388900	0.18863200	0.03917600
H	-0.05443000	-2.22937800	-0.33541100
C	1.70778500	-0.08331900	-0.56247800
C	2.45676000	-1.36245500	-0.13540400
C	2.64878200	1.08792700	-0.17470200
C	1.51798600	-0.02795100	-2.08964900
O	2.18197900	-1.91416100	0.90285200
C	3.51969100	-1.90419700	-1.05645500
O	3.77198600	0.83050100	0.19748100
C	2.20934100	2.51271800	-0.37995300
H	1.02467300	-0.94284900	-2.44875700
H	0.87485500	0.82312700	-2.34485900
H	2.47948200	0.08050900	-2.61014900
H	3.04242800	-2.35020700	-1.94196700
H	4.18564400	-1.10110300	-1.39485300
H	4.08855900	-2.67569400	-0.52589000
H	1.70795200	2.65095200	-1.34497000
H	1.47113600	2.76105700	0.39144200
H	3.09327700	3.15617300	-0.29776500

Py-3-246-R5-Nu3

C	0.16270000	-0.46701400	0.14849000
C	1.42286400	-1.22320200	0.25803900

C	2.55219200	-0.54497600	-0.09072600
C	1.38640500	1.45703000	-0.00328300
C	2.57745600	0.84103400	-0.35250300
N	1.40314400	2.95626000	0.07903400
O	0.50750700	3.50328400	0.66722400
O	2.32370300	3.52804700	-0.46071300
N	3.83410900	-1.28058500	-0.12263600
O	4.80629100	-0.68844700	-0.52765800
O	3.84200800	-2.43028300	0.25006100
N	-0.31256200	-0.64332600	-1.49556200
O	-0.69808700	0.33306800	-2.07793700
O	-0.20235500	-1.74526300	-1.98568700
N	0.26700600	0.90022900	0.36521300
H	3.47602200	1.37935000	-0.62791100
H	1.46620000	-2.27087800	0.53762500
C	-1.07739400	-1.07264600	0.90794700
C	-2.37799400	-0.48202800	0.34724700
C	-0.93116200	-0.64333600	2.31475900
C	-1.08095200	-2.61234700	0.90743500
C	-3.16705000	-1.21857600	-0.54136300
C	-2.75736900	0.82608200	0.66934600
N	-0.82856300	-0.36110000	3.42874700
H	-0.26466100	-3.00074700	1.52779300
H	-2.02838000	-2.98069400	1.32077400
H	-0.95447100	-2.98891400	-0.11399900
C	-4.31229900	-0.65460000	-1.10425100
H	-2.88648000	-2.23451700	-0.81968700
C	-3.90535400	1.38497100	0.11225800
H	-2.13436100	1.41325000	1.34412100
C	-4.68599400	0.64743900	-0.77834600
H	-4.91374700	-1.24067200	-1.80075400
H	-4.18785800	2.40602100	0.37291600
H	-5.58304000	1.08722000	-1.21704200

Py-3-246-R5-Nu2

C	0.87347200	-0.14848900	0.32425400
C	1.10279900	1.31269500	0.17909800
C	2.35115300	1.73066600	-0.15380000
C	3.00381700	-0.45105900	-0.53545200
C	3.41977800	0.86524200	-0.46906800
N	4.00746100	-1.43874400	-1.07280200
O	3.60897500	-2.51583100	-1.43023600
O	5.16430700	-1.08406100	-1.11201200
N	2.59384300	3.18943100	-0.23852600
O	3.73067100	3.55226800	-0.42619500
O	1.65173400	3.93578400	-0.11440100
N	0.97126100	-0.51215200	1.93382700
O	1.74328800	0.14454200	2.58176600
O	0.37657900	-1.48653600	2.32672200
N	1.83101800	-0.96419200	-0.28651000
H	4.41445800	1.20320600	-0.72950900
H	0.30427600	2.02511700	0.36737200

C	-0.57177500	-0.54977900	-0.09890300
Br	-0.82380000	-2.38724300	-0.59771000
S	-1.90358600	-0.04588200	1.09326700
H	-0.79635400	0.03156900	-1.00088500
O	-2.46136200	-1.18875100	1.79802800
O	-1.35395000	1.08730200	1.84039900
C	-3.16366900	0.56549000	-0.00733300
C	-4.11231800	-0.33134500	-0.49630600
C	-3.17122400	1.91721200	-0.34570700
C	-5.08869200	0.14522300	-1.36926900
H	-4.08337100	-1.37703700	-0.18732300
C	-4.15672400	2.37921700	-1.21624100
H	-2.42566700	2.59228500	0.07750400
C	-5.10797600	1.49437800	-1.72767800
H	-5.84055400	-0.53808700	-1.76528600
H	-4.18472400	3.43399000	-1.49126000
H	-5.87655700	1.86174000	-2.40908800

Py-3-246-R5-Nu4

C	0.86397800	-0.01958200	0.49538900
C	2.39661100	-0.05004400	0.46990400
C	0.21412300	-1.24607000	-0.00510500
C	2.96138000	-0.12060500	-0.92901800
H	2.74226400	0.86458700	0.97628600
H	2.72174000	-0.92169600	1.05450800
C	-1.12569900	-1.15973100	-0.22969400
C	2.95879200	1.15620800	-1.72260600
C	3.43619700	-1.27181800	-1.41717600
C	-0.97982300	1.15755700	-0.15288400
C	-1.83230900	0.06451900	-0.19754700
H	3.58528200	1.91108500	-1.22077500
H	3.34335100	0.99892700	-2.73888400
H	1.94414400	1.57658000	-1.76865500
H	3.44519000	-2.18341400	-0.81403700
H	3.83239500	-1.33535200	-2.43362700
N	-1.58618000	2.48570900	-0.49110700
O	-0.85932300	3.35610300	-0.89693700
O	-2.78153900	2.60552000	-0.33733600
N	-1.86375800	-2.37858400	-0.61343600
O	-3.06594900	-2.29651500	-0.71490500
O	-1.23415800	-3.39416600	-0.79876300
N	0.59769600	-0.03858900	2.18361400
O	0.68575900	-1.11015200	2.73621300
O	0.37338400	1.00653600	2.73756200
N	0.31096800	1.17656900	0.03622400
H	-2.89787700	0.14129200	-0.37661000
H	0.76753700	-2.16895600	-0.15953600

Py-3-345-R1

C	0.49364400	0.60482400	1.19798500
C	-0.01415700	0.08364500	0.00000000

C	1.40944800	1.66025800	1.13504900
C	0.49364400	0.60482400	-1.19798500
C	1.40944800	1.66025800	-1.13504900
C	0.14856400	0.07596500	-2.58072100
C	-1.06199100	-1.02890400	0.00000000
C	0.14856400	0.07596500	2.58072100
F	-1.03345600	0.50237200	3.01253300
F	0.14856400	-1.25585600	2.60319900
F	1.05554900	0.48208600	3.46942400
F	0.14856400	-1.25585600	-2.60319900
F	-1.03345600	0.50237200	-3.01253300
F	1.05554900	0.48208600	-3.46942400
F	-1.85144400	-0.94479900	1.06473200
F	-1.85144400	-0.94479900	-1.06473200
F	-0.49012800	-2.22611700	0.00000000
N	1.84140100	2.19149900	0.00000000
H	1.80626400	2.08255300	2.06037900
H	1.80626400	2.08255300	-2.06037900

Py-3-345-R1-Nu1

C	-0.57241400	0.07901600	-0.60643800
C	0.86254600	-0.32564500	-0.34962400
C	-0.50065500	1.13819300	-1.70483300
C	1.83362900	0.69353100	-0.41911100
C	1.57162000	1.88098500	-1.09136600
C	3.20326700	0.61758100	0.22112700
C	1.14208800	-1.68824600	0.17406400
C	-1.41152500	-1.04309700	-1.23970900
F	-0.81797500	-1.55789900	-2.31619900
F	-1.67935500	-2.05514200	-0.40543500
F	-2.60047000	-0.59012500	-1.66712500
F	3.14678400	0.24473000	1.50740200
F	4.05117600	-0.20778800	-0.40392000
F	3.81236300	1.81429800	0.22560200
F	0.80361200	-2.68264600	-0.67818000
F	2.43186400	-1.90766400	0.45425700
F	0.46181800	-1.96727800	1.30885900
N	0.44676500	1.99747400	-1.84720500
H	-1.35418300	1.19588800	-2.38852900
H	2.30023000	2.68600700	-1.14494600
C	-1.37210800	0.67392000	0.75581200
C	-2.24546900	-0.35944700	1.52534400
C	-2.24192500	1.84894000	0.26786700
C	-0.31334000	1.14866100	1.75774000
O	-1.78485300	-0.88550000	2.51239300
C	-3.68552100	-0.65254000	1.15756000
O	-3.29882400	1.66720300	-0.29097600
C	-1.73144200	3.25698700	0.47492600
H	0.29927600	0.30074400	2.07497600
H	0.34208500	1.91019100	1.32453000
H	-0.80526000	1.55809700	2.65165300
H	-3.96587100	-1.58688100	1.65815200

H	-4.30655500	0.16453600	1.55459200
H	-3.87369600	-0.69773800	0.08466600
H	-1.63258000	3.47961700	1.54636000
H	-0.73995000	3.37472900	0.01628900
H	-2.44230200	3.95144500	0.01383400

Py-3-345-R1-Nu3

C	0.25351600	0.55626200	-0.39186000
C	-1.23578100	0.33115100	-0.27651700
C	0.64121100	-0.12117600	-1.70746200
C	-1.65286000	-0.98243400	-0.58593700
C	-0.87449700	-1.79133300	-1.40085900
C	-2.90608500	-1.62529900	-0.04193900
C	-2.17351500	1.36571600	0.21756600
C	0.65544600	2.02515400	-0.59121800
F	0.19925400	2.51185100	-1.74562700
F	0.23882500	2.85439600	0.36990800
F	1.99276100	2.14951200	-0.63546600
F	-3.14794800	-1.29044000	1.23051900
F	-4.01324000	-1.34191100	-0.74429300
F	-2.80999700	-2.96450200	-0.05636400
F	-2.12338400	2.52516800	-0.47662200
F	-3.45330100	0.97586700	0.14487600
F	-1.98264400	1.74061900	1.51022700
N	0.16295800	-1.24891800	-2.09749300
H	1.38521100	0.38016700	-2.33762700
H	-1.14258600	-2.82569100	-1.60758500
C	1.11556100	-0.03543600	0.87720800
C	2.53104300	-0.48102100	0.48158700
C	0.42991700	-1.23239600	1.41172700
C	1.12695200	0.93256500	2.08117100
C	3.65953700	0.29306800	0.77328800
C	2.71776800	-1.70047500	-0.18336800
N	-0.02930000	-2.13705800	1.96308500
H	0.09853600	1.20960900	2.34163300
H	1.59643200	0.43690100	2.94154000
H	1.68415200	1.84790700	1.85986200
C	4.93475400	-0.13325900	0.40191900
H	3.55621300	1.24609300	1.28981800
C	3.99058100	-2.12945300	-0.55452500
H	1.85151900	-2.32462100	-0.41168200
C	5.10651200	-1.34500200	-0.26514500
H	5.79905300	0.48849200	0.64087000
H	4.10791400	-3.08348400	-1.07075900
H	6.10468000	-1.67837900	-0.55345900

Py-3-345-R1-Nu2

C	-0.63864000	0.73240000	-0.45580600
C	-1.80496600	-0.00432400	0.18620200
C	-0.72341700	0.60952300	-1.98047800
C	-2.82542300	-0.50940100	-0.64313800

C	-2.75914700	-0.46492300	-2.02325100
C	-4.06661700	-1.17338900	-0.08540900
C	-1.97673600	0.06988900	1.65851600
C	-0.70138300	2.24672700	-0.13697400
F	-1.93462400	2.70231600	-0.35881600
F	-0.39515800	2.53674600	1.12894000
F	0.11198200	2.95963500	-0.92517000
F	-3.82066200	-2.38012000	0.43828100
F	-4.66275000	-0.43912300	0.86329600
F	-4.99845000	-1.37098000	-1.02816100
F	-2.71326900	1.12723200	2.08477300
F	-2.57236600	-1.00897500	2.19237900
F	-0.80716900	0.19052300	2.32964600
N	-1.67740200	0.08313000	-2.65346100
H	0.13670100	1.01672200	-2.52183300
H	-3.54542300	-0.87511200	-2.65125800
C	0.75792100	0.14316500	0.02339200
Br	0.89641800	-1.68775300	-0.58882800
S	2.29637100	1.04805100	-0.43583000
H	0.82132300	0.11631700	1.11402600
O	2.39327900	1.18067200	-1.88734500
O	2.33900800	2.22961100	0.42254700
C	3.61910900	-0.01960000	0.10381000
C	4.29865900	-0.78911600	-0.83745500
C	3.96789900	-0.01394200	1.45286800
C	5.35134300	-1.59251600	-0.40482500
H	3.99919400	-0.74984900	-1.88535500
C	5.01755600	-0.82893000	1.87277900
H	3.43380800	0.62835700	2.15475900
C	5.70426300	-1.61543400	0.94617200
H	5.89810700	-2.20277900	-1.12442100
H	5.30560600	-0.84230400	2.92441500
H	6.52731900	-2.24907200	1.27959400

Py-3-345-R1-Nu4

C	1.22801600	-0.25929100	0.43934800
C	2.13611500	1.01074300	0.35926500
C	-0.18333000	-0.09168800	-0.11610900
C	1.41508600	2.29736600	0.68518300
H	2.94812800	0.86985100	1.09556100
H	2.60717300	1.09391900	-0.62844100
C	1.14134200	-0.68798500	1.90878300
C	-1.27748700	-0.45312100	0.67992100
C	0.68724900	2.37424500	2.00119100
C	1.44668800	3.33508500	-0.15729900
C	-1.14510100	-0.89554000	1.99113100
H	1.33785700	2.05307700	2.82959600
H	0.33819300	3.39732700	2.19706100
H	-0.18372300	1.69928100	2.00490000
H	1.95055800	3.26472100	-1.12386400
H	0.95630300	4.27991000	0.09143400
C	-2.70510700	-0.29292600	0.20890700

C	-0.34830400	0.37107900	-1.51625200
C	1.98029800	-1.39250300	-0.27529300
F	1.34362600	-2.56019400	-0.14973700
F	2.14348400	-1.18319700	-1.58796600
F	3.21238800	-1.56662000	0.23564200
F	-3.11477500	0.98258300	0.24822800
F	-2.90487800	-0.73301300	-1.03936800
F	-3.56930800	-0.97552300	0.97492000
F	-0.58242700	-0.62475200	-2.41207800
F	-1.36999700	1.23494400	-1.69361200
F	0.73951500	1.00204100	-1.99621900
N	0.08730400	-0.97663500	2.57734300
H	2.10990500	-0.73169900	2.42689700
H	-2.00695600	-1.16914600	2.59450700

Py-3-345-R2

C	0.00000000	1.20220000	-0.38688400
C	0.00000000	0.00000000	0.33522200
C	0.00000000	1.14301700	-1.78887500
C	0.00000000	-1.20220000	-0.38688400
C	0.00000000	-1.14301700	-1.78887500
C	0.00000000	-2.47224600	0.28680400
N	0.00000000	-3.49513600	0.82014100
C	0.00000000	0.00000000	1.77474100
N	0.00000000	0.00000000	2.92785300
C	0.00000000	2.47224600	0.28680400
N	0.00000000	3.49513600	0.82014100
N	0.00000000	0.00000000	-2.46224800
H	0.00000000	-2.07025900	-2.36675800
H	0.00000000	2.07025900	-2.36675800

Py-3-345-R2-Nu1

C	-0.01874200	-0.10532600	0.68530700
C	1.38810500	0.33382100	0.31395500
C	0.14379400	-1.52178200	1.26988300
C	2.28103100	-0.66050400	-0.14341900
C	2.03078500	-2.01289900	0.04411300
C	3.52608300	-0.27439700	-0.75650200
N	4.52525700	0.01478900	-1.26134900
C	1.81090200	1.67934100	0.41872300
N	2.20227100	2.77112000	0.50458300
C	-0.53375700	0.73055500	1.79158600
N	-0.89175600	1.31822200	2.71771700
N	1.01636500	-2.38173400	0.87789400
H	2.70743500	-2.78285800	-0.32421100
H	-0.55882600	-1.80852300	2.06284500
C	-1.08086700	-0.10212200	-0.50524900
C	-1.50424100	1.33227800	-0.86433800
C	-2.33407500	-0.84331400	-0.00491400
C	-0.47633100	-0.74069400	-1.76147700
O	-0.89533900	2.28975000	-0.45915400

C	-2.71539000	1.49701000	-1.75594300
O	-3.11526900	-0.25257100	0.70563900
C	-2.54874100	-2.28128100	-0.39571400
H	0.37895500	-0.14612400	-2.10809500
H	-0.12048200	-1.75698100	-1.55020000
H	-1.21354900	-0.79337800	-2.57423500
H	-2.60213800	2.40584200	-2.35800900
H	-2.89737200	0.62746300	-2.39982500
H	-3.58750900	1.61711000	-1.09697900
H	-2.73679000	-2.34565800	-1.47785500
H	-1.65026900	-2.87814900	-0.18541500
H	-3.41088200	-2.67705400	0.15212900

Py-3-345-R2-Nu3

C	-0.36564000	0.37367600	-0.52032000
C	-1.85563000	0.49743500	-0.29819700
C	-0.17888300	-0.74127500	-1.55434400
C	-2.61527900	-0.69430900	-0.35688500
C	-2.10695800	-1.84433100	-0.93866600
C	-3.97242900	-0.69977500	0.12322000
N	-5.06173300	-0.71685000	0.51017200
C	-2.43506800	1.74204400	0.02962600
N	-2.89215100	2.77587300	0.30747400
C	0.17143600	1.62113400	-1.09902600
N	0.58990900	2.58748400	-1.57078300
N	-0.93918600	-1.77567000	-1.63856400
H	-2.67285600	-2.77457600	-0.96456800
H	0.66541200	-0.64499400	-2.24601900
C	0.48670500	0.10933900	0.82648300
C	1.96283000	-0.06877200	0.47566200
C	-0.00389800	-1.13620800	1.44774500
C	0.22398600	1.24384000	1.82939500
C	2.84493600	1.01751900	0.51363900
C	2.44457500	-1.31207300	0.04361500
N	-0.35150600	-2.09181600	1.99296200
H	-0.82669600	1.23104500	2.14522100
H	0.86378400	1.12670100	2.71398300
H	0.42690400	2.22025000	1.36897500
C	4.17621800	0.86348200	0.12762400
H	2.49845100	1.99786800	0.84031200
C	3.77502600	-1.46515100	-0.34306300
H	1.77174600	-2.17145700	0.01534400
C	4.64597300	-0.37688300	-0.30205300
H	4.84896300	1.72143700	0.16587800
H	4.13112300	-2.44189400	-0.67363300
H	5.68844200	-0.49568900	-0.60105600

Py-3-345-R2-Nu2

C	-1.38092100	0.54810300	-0.00539300
C	-2.50013100	-0.41232200	0.33363000
C	-1.55031600	0.83472500	-1.50523400

C	-3.75814700	-0.09218500	-0.23055800
C	-3.84316300	0.74347400	-1.33378600
C	-4.95576900	-0.69779200	0.28711200
N	-5.92344700	-1.17700200	0.70060100
C	-2.31566000	-1.42064900	1.29841200
N	-2.12213700	-2.24446000	2.09857400
C	-1.59297800	1.82593900	0.74638600
N	-1.89390100	2.80788900	1.27393000
N	-2.70352500	1.02412900	-2.03435800
H	-4.80099400	1.02388200	-1.77018300
H	-0.64522100	0.90997400	-2.11373000
C	0.00394600	-0.03963700	0.35256400
Br	0.45438100	-1.53088400	-0.78874000
S	1.31401700	1.25960200	0.34164500
H	0.00795700	-0.41715200	1.38316600
O	1.12214200	2.07523900	-0.85453500
O	1.25330100	1.86781400	1.66682800
C	2.86709600	0.40984700	0.20326400
C	3.47772600	0.31908300	-1.04577200
C	3.44605500	-0.10904500	1.35977200
C	4.70708800	-0.32924400	-1.13622400
H	2.99350500	0.75297400	-1.92132400
C	4.67314000	-0.76034800	1.25135100
H	2.94763600	0.00649800	2.32323900
C	5.29796300	-0.86999700	0.00777500
H	5.20592600	-0.41188100	-2.10226900
H	5.14567000	-1.17642800	2.14151600
H	6.25986000	-1.37858800	-0.07022300

Py-3-345-R2-Nu4

C	-0.37953700	-0.74852800	0.47026700
C	-1.84763300	-0.33001600	0.74165600
C	0.54523500	0.39764700	0.12436200
C	-2.52878000	0.46736700	-0.34966400
H	-2.42040400	-1.25549100	0.92703700
H	-1.86694000	0.25284000	1.67374500
C	-0.25268100	-1.74582700	-0.68372900
C	1.77862000	0.03422800	-0.46288900
C	-2.79970300	-0.20561700	-1.66990600
C	-2.92559800	1.72294300	-0.11924000
C	1.95104000	-1.21008500	-1.05481000
H	-3.27340300	-1.18907900	-1.52293400
H	-3.46108000	0.41166500	-2.29215000
H	-1.86687100	-0.37833500	-2.22692600
H	-2.71356700	2.22175000	0.82863900
H	-3.45453200	2.29526000	-0.88560800
C	2.85652500	0.98669200	-0.52920100
N	3.73039600	1.74209700	-0.58301600
C	0.31128800	1.69171900	0.63353400
N	0.12446700	2.75280900	1.07604300
C	0.10145000	-1.43526100	1.71391300
N	0.49645500	-1.96924400	2.65967500

N	0.85546600	-1.99696100	-1.28356200
H	2.89845900	-1.50198100	-1.50677400
H	-1.15049800	-2.30910600	-0.96476700

Py-3-345-R3

C	-1.23739500	1.12541500	0.03523700
C	-1.20649600	2.51835300	-0.08863300
C	-0.02718700	0.42179100	0.05992400
C	-2.58900500	0.48469500	0.11170700
C	-0.00552600	-1.07542600	0.27815800
C	1.15242100	1.16623700	-0.06413900
O	-2.55851800	-0.80390700	-0.20563600
O	-3.59232100	1.08370500	0.39592300
C	1.05911800	2.55602400	-0.20402800
O	0.21410500	-1.72212800	-0.85446900
O	-0.17213700	-1.58458700	1.34980700
C	2.53400500	0.58618600	-0.05503400
C	-3.79894800	-1.50328700	-0.13471600
C	0.27862900	-3.14071000	-0.74869700
O	2.56306300	-0.66669200	0.38133300
O	3.51179400	1.20315300	-0.38685400
H	-3.58349800	-2.53595900	-0.42133100
H	-4.19802600	-1.46155700	0.88650600
H	-4.52430700	-1.05435300	-0.82464300
H	0.45788400	-3.51687400	-1.75942200
H	-0.66636800	-3.53448300	-0.35232500
H	1.09781800	-3.43179100	-0.07781100
C	3.84232000	-1.29525200	0.42168800
H	4.51964000	-0.72914200	1.07300100
H	4.27034800	-1.34594800	-0.58742400
H	3.67527700	-2.29907300	0.82098200
N	-0.08806900	3.22507200	-0.20909800
H	-2.15518000	3.06215700	-0.09700900
H	1.98339600	3.13100800	-0.30648700

Py-3-345-R3-Nu1

C	0.78788700	0.03063100	-0.51399100
C	0.98154500	-0.54408600	-1.92715500
C	-0.70880700	0.19623700	-0.37391900
C	1.48119400	1.39780000	-0.46640800
C	-1.26893700	1.38991400	0.18964900
C	-1.51968900	-0.82512800	-0.93344800
O	1.16162300	2.13695600	-1.53173100
O	2.24144100	1.78401300	0.38186700
C	-1.03031400	-1.64985700	-1.92266900
O	-2.63322300	1.44176800	0.09785800
O	-0.66256500	2.33641800	0.67262600
C	-2.90441100	-1.10982500	-0.45994400
C	1.59199400	3.48676400	-1.48498700
C	-3.24983400	2.54691700	0.71948200
O	-2.97375600	-1.18707000	0.87792300

O	-3.85549400	-1.34296600	-1.16592800
H	1.23491800	3.95938700	-2.40534800
H	1.15343900	3.97733500	-0.60533500
H	2.68682800	3.54747500	-1.42454800
H	-4.32886400	2.42947300	0.56762900
H	-2.91181200	3.49362500	0.27502600
H	-3.02504200	2.57545700	1.79581900
C	-4.26890300	-1.38189500	1.41474300
H	-4.70555000	-2.32491000	1.05839500
H	-4.93246300	-0.55587800	1.12099400
H	-4.15500400	-1.40490700	2.50307900
N	0.18564800	-1.37674900	-2.49896100
H	1.87517300	-0.22669700	-2.47915200
H	-1.62267600	-2.46614900	-2.33809200
C	1.49430800	-0.92835900	0.56325800
C	1.69395400	-0.19824800	1.91198200
C	2.82489600	-1.38024400	-0.06881400
C	0.59356900	-2.12250600	0.91496800
O	0.72101300	0.18702000	2.51504600
C	3.07360800	-0.13338000	2.52126000
O	3.69182500	-0.58056100	-0.34168300
C	3.00590500	-2.83672400	-0.42820800
H	-0.35767500	-1.74246200	1.30289400
H	0.38665900	-2.75148700	0.04192800
H	1.06547400	-2.74264400	1.69308800
H	3.00193500	0.32332100	3.51493200
H	3.48705700	-1.15112600	2.60819300
H	3.73699200	0.44312000	1.86796200
H	2.84768000	-3.49360900	0.43734400
H	2.25627600	-3.10734700	-1.18764200
H	4.01242600	-2.97741200	-0.83704600

Py-3-345-R3-Nu3

C	-0.40064200	0.27745300	0.40328200
C	-0.73123500	-0.25191800	1.80542300
C	1.10439300	0.23218400	0.29751100
C	-0.90491800	1.72520200	0.35503600
C	1.80032900	1.29437500	-0.35900700
C	1.76076900	-0.86320000	0.91456800
O	-0.36451900	2.44493400	1.34189700
O	-1.72255000	2.18470000	-0.39957200
C	1.13945400	-1.58263800	1.90958700
O	3.15309800	1.25409500	-0.18683900
O	1.29290300	2.20757800	-1.00365900
C	3.10378200	-1.34099300	0.47923000
C	-0.64349700	3.83425300	1.30792800
C	3.90056300	2.18076400	-0.94337000
O	3.20046700	-1.42720200	-0.85538900
O	3.99367700	-1.69636300	1.21346800
H	-0.12462900	4.27584200	2.16425300
H	-0.26500000	4.26165100	0.36904800
H	-1.72352100	4.02018700	1.37791300

H	4.95348800	2.01482000	-0.68938500
H	3.61561300	3.21373500	-0.70044900
H	3.74941800	2.02765600	-2.02255300
C	4.46856300	-1.80867600	-1.35550500
H	4.75144700	-2.80618300	-0.99276600
H	5.23726900	-1.08932400	-1.03844000
H	4.38427200	-1.81409400	-2.44683700
N	-0.05690900	-1.15177500	2.42720200
H	-1.60992100	0.17106400	2.30941800
H	1.60275100	-2.46186600	2.35855400
C	-1.13933200	-0.57044800	-0.73856600
C	-2.66068000	-0.62767100	-0.54508300
C	-0.66454100	-1.97224900	-0.66571300
C	-0.71028300	-0.07479500	-2.13254100
C	-3.53132800	0.12508800	-1.34140800
C	-3.21099500	-1.45451200	0.44420600
N	-0.34972900	-3.08244000	-0.70079300
H	0.37628000	-0.19262400	-2.23431000
H	-1.20838600	-0.66572200	-2.91387600
H	-0.95476900	0.98485800	-2.25251100
C	-4.90989200	0.06300700	-1.14558300
H	-3.12825700	0.78154700	-2.11042900
C	-4.59000600	-1.51009200	0.64842400
H	-2.55147100	-2.07390900	1.05501200
C	-5.44610400	-0.75035900	-0.14662700
H	-5.56972000	0.65788700	-1.77945200
H	-4.99371500	-2.15987900	1.42658000
H	-6.52571000	-0.79674600	0.00536600

Py-3-345-R3-Nu2

C	-0.81134200	-1.14987500	0.54061800
C	-1.12273300	-1.53161900	1.99396700
C	-1.57752300	0.13588900	0.35346900
C	-1.23515700	-2.26125400	-0.40443500
C	-2.47239500	0.31305300	-0.76197400
C	-1.40357900	1.08603300	1.38847100
O	-2.43750300	-2.74141000	-0.09702400
O	-0.54542500	-2.70263800	-1.28559700
C	-1.00935500	0.65904900	2.64764500
O	-3.21481400	1.45046900	-0.66764900
O	-2.63491800	-0.44970800	-1.69931100
C	-1.63964900	2.54759500	1.21853300
C	-2.99067700	-3.64726800	-1.03884600
C	-4.04880600	1.73407200	-1.77095800
O	-1.09158000	3.03570300	0.09412100
O	-2.18967600	3.26155000	2.02096300
H	-3.97344100	-3.93460600	-0.65291100
H	-3.09140600	-3.14304500	-2.00939200
H	-2.35075000	-4.53209200	-1.15266700
H	-4.55529800	2.67880900	-1.54468900
H	-4.79135900	0.93762100	-1.92025100
H	-3.46196800	1.83437700	-2.69521700

C	-1.35615100	4.39982600	-0.18150000
H	-0.97032700	5.04509200	0.61923700
H	-2.43864800	4.56541100	-0.27608900
H	-0.85528900	4.62756000	-1.12798100
N	-1.07638100	-0.67329700	2.95106300
H	-1.38351000	-2.57246600	2.21355000
H	-0.82469100	1.36473600	3.45868600
C	0.71292400	-0.90791300	0.41988400
Br	1.79553700	-2.35244900	1.13795100
S	1.36027500	-0.46147900	-1.25356100
H	0.94855400	-0.03723700	1.04502500
O	2.12628600	-1.55166300	-1.84511100
O	0.28632600	0.16767600	-2.01285400
C	2.53286000	0.81538700	-0.81366100
C	3.89223600	0.51845700	-0.81688200
C	2.04462500	2.07829200	-0.47893700
C	4.79533400	1.52510800	-0.47318100
H	4.22344200	-0.48368900	-1.09121300
C	2.95871100	3.07164800	-0.13361000
H	0.96802000	2.27037800	-0.48583400
C	4.32867600	2.79521500	-0.13295500
H	5.86582400	1.31604300	-0.47250000
H	2.60078800	4.06704500	0.13337700
H	5.03897400	3.57850300	0.13590800

Py-3-345-R3-Nu4

C	1.05446900	-0.16546700	-0.22072600
C	1.43367400	-1.14255400	0.92978800
C	1.31098500	-0.78553100	-1.59713600
C	2.85359300	-1.66976900	0.95216900
H	1.20301900	-0.62582500	1.87355600
H	0.73262800	-1.98685500	0.83820400
C	-0.43416900	0.10657900	-0.23088900
C	1.85600300	1.12048700	-0.10428800
C	3.73100600	-1.21075700	2.08604100
C	3.29726400	-2.53646000	0.03170500
C	-0.97105300	1.35056500	0.22430000
C	-1.22906400	-0.97337200	-0.69163700
O	1.68249900	1.91309900	-1.16707400
O	2.60706100	1.40492200	0.79460300
C	-0.67226000	-1.92506000	-1.52467000
H	3.79045200	-0.11427700	2.09487300
H	3.29140200	-1.51780800	3.04941600
H	4.74152500	-1.63640000	2.01582400
H	2.64736100	-2.90323700	-0.76555500
H	4.32547700	-2.90774800	0.06000600
O	-2.30059100	1.50190200	-0.05512000
O	-0.36281600	2.25253000	0.79160800
C	-2.64755400	-1.17858400	-0.28931500
C	2.24168400	3.21028300	-1.06142500
C	-2.92871400	2.61883600	0.53257500
O	-2.84459500	-0.97860700	1.02258600

O	-3.53262500	-1.56186000	-1.01714000
H	1.99118900	3.73424100	-1.98931800
H	1.80127000	3.72909500	-0.19878900
H	3.33166700	3.16118500	-0.93488700
H	-3.98252800	2.57981600	0.23392900
H	-2.47771300	3.55887500	0.18461600
H	-2.85114600	2.58869500	1.62992700
C	-4.18594200	-1.07878400	1.46236800
H	-4.58540700	-2.08689800	1.28518700
H	-4.81956000	-0.35448300	0.93059400
H	-4.18005400	-0.85868900	2.53469800
N	0.54738700	-1.68468900	-2.11009800
H	2.19741900	-0.47029400	-2.16166600
H	-1.23970100	-2.79493000	-1.85878000

Py-3-345-R4

C	0.00000000	1.18937500	0.01004500
C	0.00000000	0.00000000	0.73078200
C	0.00000000	1.14643700	-1.38097400
C	0.00000000	-1.18937500	0.01004500
C	0.00000000	-1.14643700	-1.38097400
F	0.00000000	-2.34159200	0.66048100
F	0.00000000	0.00000000	2.04838200
N	0.00000000	0.00000000	-2.05434700
H	0.00000000	-2.08657700	-1.93861100
H	0.00000000	2.08657700	-1.93861100
F	0.00000000	2.34159200	0.66048100

Py-3-345-R4-Nu1

C	0.30942900	0.34060200	-0.36420100
C	1.39001700	-0.55876500	0.00963500
C	0.80677200	1.72938500	-0.24496600
C	2.69792600	-0.15284800	0.06492000
C	3.04700000	1.19478500	-0.07989300
F	3.65023000	-1.06820700	0.31490900
F	1.08511600	-1.86018400	0.27333000
F	-0.08410300	0.12608000	-1.78454200
N	2.06776500	2.09888300	-0.19442400
H	4.09111400	1.51051900	-0.04083400
H	0.06242200	2.53111500	-0.30876400
C	-1.03187900	0.02797800	0.40208300
C	-1.55966700	-1.41371900	0.16038600
C	-2.13730300	0.97913000	-0.10666400
C	-0.79485600	0.19374900	1.90410600
O	-2.01227200	-2.02562900	1.10179400
C	-1.55760100	-2.02680000	-1.21525000
O	-2.77305600	0.74077800	-1.10682600
C	-2.45949300	2.20533900	0.72048700
H	-0.08143100	-0.57044900	2.24098000
H	-0.36866000	1.17981100	2.12566700
H	-1.72500500	0.05457300	2.46862600

H	-2.09605900	-2.98068300	-1.16316900
H	-2.01645700	-1.33534600	-1.92875100
H	-0.52166300	-2.18610500	-1.53692400
H	-2.98847600	1.88916200	1.63194500
H	-1.55761700	2.74044200	1.04052900
H	-3.11316800	2.86136600	0.13478200

Py-3-345-R4-Nu3

C	0.71214800	-0.22883700	0.36392500
C	2.07662700	0.24560900	0.25155500
C	0.55111700	-1.39626200	-0.51591900
C	3.08325900	-0.57009600	-0.20100600
C	2.80418400	-1.81264000	-0.77839100
F	4.35065500	-0.12781900	-0.13942800
F	2.39313500	1.49288700	0.68682800
F	0.46310200	-0.66524300	1.81782900
N	1.52839000	-2.16115700	-0.96182800
H	3.61104200	-2.45148800	-1.14329700
H	-0.47107100	-1.71118900	-0.74688000
C	-0.40256500	0.87599900	0.22714600
C	-1.79721600	0.26123700	0.03922600
C	-0.05391400	1.68139300	-0.96008500
C	-0.44590600	1.83707100	1.43764000
C	-2.33849900	-0.52536400	1.06748400
C	-2.56066400	0.47351000	-1.11194400
N	0.21035200	2.34624600	-1.86582800
H	0.52639600	2.31696500	1.58972400
H	-1.21090000	2.60717400	1.26672500
H	-0.70126200	1.26378000	2.33395700
C	-3.60927000	-1.07945600	0.94177700
H	-1.73357700	-0.72571800	1.95072800
C	-3.83658000	-0.08250900	-1.23636000
H	-2.16178800	1.07732200	-1.92821700
C	-4.36708500	-0.85942700	-0.21069700
H	-4.00973400	-1.69338300	1.75039200
H	-4.41334900	0.09617000	-2.14533600
H	-5.36279100	-1.29505200	-0.30738100

Py-3-345-R4-Nu2

C	-1.66269400	0.58697100	-0.03312400
C	-2.69052100	-0.03765900	0.76621500
C	-2.00627000	0.47474400	-1.45711100
C	-3.92297500	-0.34940500	0.24701100
C	-4.18797900	-0.20796400	-1.11688600
F	-4.86694400	-0.83289000	1.07088300
F	-2.43265300	-0.26252100	2.07931700
F	-1.52579700	2.04605200	0.36132400
N	-3.19610200	0.17205900	-1.93049700
H	-5.17059100	-0.45601200	-1.52278900
H	-1.21663500	0.72185600	-2.17183700
C	-0.23865400	0.14657100	0.40113100

Br	0.16111700	-1.63925600	-0.21832400
S	1.06921900	1.34815900	-0.09356000
H	-0.17846700	0.13329800	1.49561000
O	0.88063000	1.70889800	-1.49683300
O	1.12958700	2.37959700	0.93676600
C	2.60350100	0.43991100	0.02182900
C	3.18854500	-0.05656800	-1.14004700
C	3.19491300	0.28664000	1.27408900
C	4.39747500	-0.74229900	-1.03827900
H	2.69579800	0.09785700	-2.10063800
C	4.39958700	-0.40837200	1.36306800
H	2.72123100	0.71530800	2.15858200
C	4.99662600	-0.92161800	0.21000100
H	4.87310200	-1.13834800	-1.93623800
H	4.87782300	-0.54227000	2.33399800
H	5.94134500	-1.46212800	0.28452800

Py-3-345-R4-Nu4

C	-0.08361700	-0.46621300	0.76370800
C	-1.57737600	-0.14856700	0.92515100
C	0.71765300	0.63270100	0.25744900
C	-2.29313100	0.24151100	-0.34590200
H	-2.04792600	-1.04096800	1.36890300
H	-1.65253500	0.66373500	1.66059900
C	0.26049100	-1.68460000	0.01128400
C	1.87914600	0.41007000	-0.43464200
C	-2.65325800	-0.86626900	-1.29817300
C	-2.58925900	1.51920200	-0.60662600
C	2.23091000	-0.87633400	-0.86838600
H	-3.24542600	-1.64021000	-0.78383800
H	-3.22977100	-0.49078000	-2.15422800
H	-1.74324900	-1.35979400	-1.67121500
H	-2.30878300	2.31432700	0.08791700
H	-3.10469600	1.80590200	-1.52715900
F	2.66257700	1.45816800	-0.75590700
F	0.36182400	1.90100500	0.59087700
F	0.27023400	-0.69985700	2.22968900
N	1.37287000	-1.88328200	-0.66588600
H	3.15353400	-1.04045800	-1.42919900
H	-0.43231300	-2.53163000	0.10432500

Py-3-345-R5

C	0.00427500	-1.18250100	0.61737500
C	0.00000000	0.00000000	-0.11089800
C	0.00000000	-1.14045500	2.01200000
C	-0.00427500	1.18250100	0.61737500
C	0.00000000	1.14045500	2.01200000
N	-0.06227900	2.49584200	-0.04498500
O	-0.48753100	2.51895700	-1.17225600
O	0.30307100	3.44380600	0.59831800
N	0.00000000	0.00000000	-1.59432600

O	1.03067900	0.31387300	-2.11742400
O	-1.03067900	-0.31387300	-2.11742400
N	0.06227900	-2.49584200	-0.04498500
O	0.48753100	-2.51895700	-1.17225600
O	-0.30307100	-3.44380600	0.59831800
N	0.00000000	0.00000000	2.68958500
H	-0.00710000	2.08017300	2.56883100
H	0.00710000	-2.08017300	2.56883100

Py-3-345-R5-Nu1

C	0.33526200	-0.07768700	0.57122100
C	-1.05212700	-0.44658700	0.20218700
C	0.21689200	1.01389400	1.65478400
C	-2.07473000	0.51299100	0.34236500
C	-1.92718200	1.63623900	1.10237900
N	-3.28147500	0.45683500	-0.49542100
O	-4.34368800	0.66847800	0.03929000
O	-3.12622300	0.27710700	-1.67943900
N	-1.33557100	-1.71925900	-0.28679100
O	-2.50845900	-2.07263200	-0.38921900
O	-0.39429900	-2.45303900	-0.58219500
N	0.97610700	-1.21631200	1.43007200
O	0.24083400	-1.91361000	2.07079300
O	2.18076500	-1.22629300	1.50884200
N	-0.78674100	1.78072000	1.85474700
H	-2.68786600	2.41410900	1.14506900
H	1.09531600	1.11395300	2.30224800
C	1.32048100	0.33379600	-0.64874000
C	2.53642500	-0.62920400	-0.88034300
C	1.77761400	1.77455800	-0.33430600
C	0.52420300	0.26827000	-1.96146900
O	2.32485500	-1.69869200	-1.39293700
C	3.95405600	-0.18135700	-0.61648800
O	2.64003600	1.98473400	0.48573400
C	1.05243000	2.91757200	-0.99974000
H	0.25166000	-0.77555500	-2.14701200
H	-0.38922400	0.87223600	-1.92814900
H	1.15233300	0.61435600	-2.79558600
H	4.61675100	-1.01156300	-0.88493400
H	4.19245800	0.69719100	-1.23372000
H	4.08262900	0.11035100	0.42937100
H	1.16199300	2.87706000	-2.09178900
H	-0.02443100	2.84945600	-0.77881200
H	1.45162100	3.86118700	-0.61299800

Py-3-345-R5-Nu3

C	0.03465000	0.33534900	0.42604900
C	1.49968000	0.40127300	0.14981700
C	-0.14727800	-0.62906600	1.61075100
C	2.28166300	-0.74235900	0.44099800
C	1.83544600	-1.74019400	1.25028600

N	3.52126900	-1.00814900	-0.30101300
O	4.44729400	-1.48877100	0.31107700
O	3.51515600	-0.79317700	-1.48831200
N	2.09842900	1.58404400	-0.25549800
O	3.32513200	1.66435200	-0.26157300
O	1.37674300	2.53270700	-0.58259400
N	-0.42970100	1.69122900	1.02305700
O	0.16287700	2.05278900	2.00620900
O	-1.36446600	2.26412700	0.53134100
N	0.65089900	-1.57210900	1.93362900
H	2.38506300	-2.67082200	1.38108500
H	-1.03699900	-0.46008700	2.22943500
C	-0.87134500	-0.05937600	-0.81596300
C	-2.35206000	-0.27712200	-0.46060100
C	-0.33846700	-1.37405800	-1.24837100
C	-0.65367600	0.88299600	-2.01571100
C	-3.32642000	0.66592500	-0.80551200
C	-2.75486400	-1.44541800	0.19960300
N	0.06069000	-2.39039100	-1.62046100
H	0.40563800	0.87597800	-2.30111300
H	-1.25534900	0.52993600	-2.86396400
H	-0.93246500	1.90814100	-1.75867300
C	-4.66504500	0.45396500	-0.48183800
H	-3.03937600	1.58137400	-1.32004800
C	-4.09313600	-1.65350700	0.53339500
H	-2.01879100	-2.21375600	0.44514100
C	-5.05358000	-0.70275500	0.19429200
H	-5.40923400	1.20143800	-0.76064500
H	-4.38273100	-2.56935200	1.05044900
H	-6.10214400	-0.86534800	0.44798900

Py-3-345-R5-Nu2

C	-1.25841000	-0.88595400	0.24332200
C	-1.87776800	0.42329800	-0.13215600
C	-1.85788900	-1.19972500	1.63069200
C	-1.89244800	1.38366200	0.90496900
C	-1.82736400	1.01381800	2.22152400
N	-1.84240000	2.82395900	0.62226900
O	-2.49780400	3.55238400	1.32951500
O	-1.10502200	3.20601400	-0.25653500
N	-2.50021300	0.67452300	-1.35699400
O	-3.09527900	1.74143000	-1.49087500
O	-2.46098000	-0.17321700	-2.23842800
N	-1.67145500	-2.02495900	-0.67519800
O	-2.80115700	-2.42410800	-0.56924200
O	-0.83314700	-2.46657600	-1.41496700
N	-2.00609700	-0.30997300	2.53539000
H	-1.73148800	1.74121200	3.02603300
H	-2.16738100	-2.23281100	1.81730900
C	0.27685000	-0.83117600	0.35825200
Br	1.03683200	-2.37857200	1.23364600
S	1.22817600	-0.46154400	-1.19277200

H	0.49060400	0.01876500	1.02025700
O	1.95142500	-1.63932200	-1.64651000
O	0.33012700	0.25073800	-2.09052100
C	2.42417200	0.70017600	-0.56297500
C	3.72193000	0.26560900	-0.30941800
C	2.01458900	2.01539900	-0.34163300
C	4.64383100	1.18643300	0.18862400
H	3.99499800	-0.77116500	-0.50924700
C	2.94721900	2.92048300	0.16013500
H	0.98538100	2.31784500	-0.55483000
C	4.25577100	2.50621400	0.42253900
H	5.66803900	0.87176200	0.39175700
H	2.65174600	3.95441000	0.34186300
H	4.98112300	3.22168500	0.81256700

Py-3-345-R5-Nu4

C	-0.80989200	0.67898000	-0.08112700
C	-2.20799500	0.06790100	-0.33299700
C	0.36064200	-0.20567100	-0.10877100
C	-2.53280100	-1.11366400	0.55477000
H	-2.93200300	0.87867300	-0.14993400
H	-2.28290400	-0.22505100	-1.38427600
C	-0.79928500	1.46222000	1.21662200
C	1.49518800	0.18314600	0.63529100
C	-2.71600800	-0.85791400	2.02851700
C	-2.68325100	-2.33571300	0.03416700
C	1.44318700	1.15979800	1.59050400
H	-3.40811500	-0.01857600	2.19993700
H	-3.11421300	-1.74839400	2.53234300
H	-1.76320700	-0.59194300	2.51148900
H	-2.52231700	-2.51937600	-1.02948400
H	-2.95003700	-3.18522900	0.66799700
N	2.83814100	-0.30922000	0.29785300
O	3.15069700	-0.32259200	-0.86754300
O	3.57160400	-0.60579800	1.21333900
N	0.39890400	-1.37796200	-0.88023800
O	1.22986400	-2.23677800	-0.60106000
O	-0.38974100	-1.50983600	-1.81045500
N	-0.69990400	1.78211700	-1.25160800
O	0.07701800	1.58219200	-2.14482200
O	-1.42789600	2.74361400	-1.15842400
N	0.23545300	1.70697100	1.93749500
H	2.34009600	1.50105900	2.10591400
H	-1.76652900	1.85459600	1.55136800

Py-3-426-R1

C	0.03080000	1.44125900	0.00000000
C	0.02631700	0.74464600	1.20422000
C	0.02631700	0.74464600	-1.20422000
C	0.00719200	-0.64666500	1.13467500
C	0.00719200	-0.64666500	-1.13467500

N	-0.00418900	-1.32909800	0.00000000
C	-0.00095900	-1.49784500	-2.38976400
C	0.00087300	2.95261200	0.00000000
C	-0.00095900	-1.49784500	2.38976400
F	0.04574200	-0.74127500	-3.48489100
F	-1.10155200	-2.24119400	-2.45176800
F	1.04332800	-2.31958200	-2.41363800
F	0.60395400	3.44721800	-1.07734600
F	-1.25363600	3.40042700	0.00000000
F	0.60395400	3.44721800	1.07734600
F	-1.10155200	-2.24119400	2.45176800
F	1.04332800	-2.31958200	2.41363800
F	0.04574200	-0.74127500	3.48489100
H	0.04245600	1.26098400	-2.16355100
H	0.04245600	1.26098400	2.16355100

Py-3-426-R1-Nu1

C	0.66674600	-0.04020200	0.43569500
C	-0.21962500	-1.25399900	0.38994300
C	-0.22131800	1.17005500	0.47832500
C	-1.52775400	-1.14377500	0.02898000
C	-1.53162200	1.08971400	0.10139100
N	-2.23584300	-0.01517200	-0.19393200
C	-2.34501200	2.35691600	0.00112500
C	1.48969900	-0.08248100	1.73465900
C	-2.33987900	-2.39840500	-0.17292300
F	-1.62802800	3.46083000	0.25440200
F	-3.37717900	2.36546500	0.85180800
F	-2.85976400	2.50958100	-1.22584400
F	2.05921700	1.08687400	2.03918200
F	0.72003400	-0.40188800	2.77869300
F	2.47001500	-1.00016400	1.69777100
F	-3.48511400	-2.37716400	0.51727700
F	-2.67610700	-2.55620500	-1.46231200
F	-1.69087300	-3.51100900	0.19144600
H	0.21153100	2.13075200	0.75388400
H	0.21992800	-2.23279300	0.57053000
C	1.67734100	0.00466500	-0.87622700
C	2.59900700	-1.23705400	-0.89905100
C	2.44073500	1.33246700	-0.86338400
C	0.77531900	-0.11072400	-2.11267000
O	2.09263100	-2.31838200	-1.10375700
C	4.09995600	-1.13364100	-0.75710000
O	3.44758700	1.47834700	-0.20630400
C	1.89781200	2.48203900	-1.68124700
H	0.29274400	-1.09326400	-2.11092500
H	-0.00455300	0.66146400	-2.10442800
H	1.36552400	-0.01319500	-3.03585800
H	4.51379600	-2.14231900	-0.86821600
H	4.50696000	-0.47155000	-1.53451000
H	4.37706000	-0.69485100	0.20634900
H	1.95835800	2.24742600	-2.75353400

H	0.83840600	2.65700000	-1.44691200
H	2.49115900	3.37748800	-1.46525700

Py-3-426-R1-Nu3

C	-0.22599200	-0.46930300	0.41370700
C	0.13685900	0.98310400	0.53342300
C	1.04963200	-1.26162200	0.30966400
C	1.37314700	1.41620400	0.14856100
C	2.21161400	-0.64914400	-0.05089500
N	2.43240200	0.67203400	-0.21505500
C	3.43670200	-1.48995500	-0.31313400
C	-0.98547200	-0.89061600	1.68154400
C	1.64465600	2.89977400	0.09513000
F	3.24072200	-2.79250900	-0.07556000
F	4.47753800	-1.11425600	0.43789500
F	3.83096600	-1.38694500	-1.59020600
F	-1.54167800	-2.10649800	1.56919100
F	-0.17157500	-0.94024200	2.73779500
F	-1.97059200	-0.04441200	2.00499400
F	2.71068800	3.24887600	0.82156200
F	1.88984700	3.29934200	-1.16137900
F	0.61754700	3.63761400	0.53626700
H	1.01312800	-2.34060900	0.45828600
H	-0.61922600	1.69094700	0.86766400
C	-1.17190400	-0.74593700	-0.88873000
C	-2.56833000	-0.14642200	-0.74493000
C	-1.26603400	-2.20213200	-1.10075700
C	-0.44527300	-0.19535800	-2.13070300
C	-2.76171200	1.22764700	-0.94275700
C	-3.66987000	-0.92904600	-0.37669700
N	-1.30336800	-3.33608900	-1.31443800
H	0.52119400	-0.70115400	-2.25391500
H	-1.05835600	-0.35909500	-3.02705900
H	-0.25371600	0.87743200	-2.01225200
C	-4.01900700	1.80275800	-0.76018000
H	-1.92518100	1.86177300	-1.23542200
C	-4.92794400	-0.35532300	-0.20253500
H	-3.54179900	-2.00112700	-0.21761400
C	-5.10774400	1.01481800	-0.38946500
H	-4.14636000	2.87534300	-0.91516700
H	-5.77210000	-0.98566900	0.08153700
H	-6.09196500	1.46512600	-0.25212600

Py-3-426-R1-Nu2

C	0.67708100	-0.45875000	0.89639500
C	1.19719000	0.94717100	1.04585800
C	1.67304600	-1.23260900	0.07224600
C	2.08431800	1.43673300	0.13317000
C	2.52346000	-0.56703600	-0.76018800
N	2.72497100	0.76218500	-0.84110500
C	3.40342600	-1.35610200	-1.69932600

C	0.52818200	-1.11209200	2.27345700
C	2.45970600	2.89764500	0.18457100
F	3.22947800	-2.67901800	-1.59235500
F	4.70286200	-1.12027500	-1.48488100
F	3.16465400	-1.03674400	-2.97769700
F	-0.01171400	-2.33259100	2.20636500
F	1.71318100	-1.24271500	2.86936000
F	-0.23916400	-0.38633700	3.09393300
F	3.77716500	3.07182100	0.33860400
F	2.12277800	3.52786300	-0.94889100
F	1.86408300	3.55774000	1.18433900
H	1.64077700	-2.32031200	0.09241300
H	0.76075400	1.58848500	1.80718100
C	-0.71272300	-0.41910600	0.13740900
Br	-1.30756400	-2.12120700	-0.59593500
S	-2.14018700	0.34133900	1.01428100
H	-0.54939800	0.22835700	-0.73285400
O	-2.77285200	-0.65667800	1.86921900
O	-1.68458500	1.60882100	1.58033500
C	-3.26516300	0.72722200	-0.31420300
C	-4.32789500	-0.13413000	-0.57656700
C	-3.06229800	1.90205400	-1.03695000
C	-5.20720300	0.18963900	-1.60840900
H	-4.45403400	-1.03506700	0.02493400
C	-3.94598700	2.20977300	-2.06919400
H	-2.23188600	2.56354900	-0.78364100
C	-5.01308300	1.35465700	-2.35263600
H	-6.04717200	-0.46916100	-1.83123100
H	-3.80540100	3.12278300	-2.64862000
H	-5.70343800	1.60163600	-3.16042900

Py-3-426-R1-Nu4

C	0.95101200	-0.48866800	0.01333200
C	1.80949900	-0.62929000	-1.29608800
C	-0.25715100	-1.39218500	-0.08209000
C	3.08520400	0.17816700	-1.38484700
H	1.12405100	-0.32580400	-2.09863500
H	2.02824200	-1.69985800	-1.44195500
C	0.46491800	0.92137100	0.24373100
C	-1.51036800	-0.87662400	-0.21062900
C	4.37188100	-0.54487300	-1.08503800
C	3.06574000	1.46674900	-1.74602100
C	-0.84951900	1.24301700	0.08471000
N	-1.88725700	0.41962000	-0.17439400
H	5.23223700	0.13755000	-1.10305000
H	4.54015000	-1.33582800	-1.83391500
H	4.33469600	-1.03964000	-0.10457000
H	2.12821900	1.96885600	-1.99693900
H	3.98619800	2.05441700	-1.79680200
C	-1.27283600	2.68448600	0.22465600
C	1.79909400	-0.92609300	1.20773900
C	-2.67482600	-1.81584200	-0.40398400

F	-0.25035800	3.51695300	0.46318600
F	-2.14491900	2.85565200	1.22737200
F	-1.87931200	3.13251200	-0.88334800
F	2.80506600	-0.07453200	1.46475900
F	1.08773100	-1.01669200	2.33332100
F	2.36212000	-2.13282900	1.01267900
F	-3.57994000	-1.69965200	0.57689000
F	-3.32526600	-1.56870100	-1.54912600
F	-2.31530500	-3.10624100	-0.43977600
H	1.19899100	1.69348500	0.46848500
H	-0.09623400	-2.47004600	-0.11828100

Py-3-426-R2

C	0.00000000	0.00000000	1.41009900
C	0.00000000	1.20618200	0.70638200
C	0.00000000	-1.20618200	0.70638200
C	0.00000000	1.13717300	-0.69081800
C	0.00000000	-1.13717300	-0.69081800
N	0.00000000	0.00000000	-1.37865100
C	0.00000000	0.00000000	2.85338300
N	0.00000000	0.00000000	4.00646600
C	0.00000000	-2.36468200	-1.46726100
N	0.00000000	-3.34662400	-2.07115700
C	0.00000000	2.36468200	-1.46726100
N	0.00000000	3.34662400	-2.07115700
H	0.00000000	2.16651500	1.22048000
H	0.00000000	-2.16651500	1.22048000

Py-3-426-R2-Nu1

C	0.08448100	-0.14375100	0.64543500
C	-0.83028900	-1.33051300	0.42792900
C	-0.79847800	1.08786400	0.66690200
C	-2.08710300	-1.15964000	-0.08558600
C	-2.05876700	1.06348200	0.12997900
N	-2.74798100	-0.00247700	-0.32993000
C	0.73688600	-0.26084700	1.97694800
N	1.16798400	-0.32774100	3.04563200
C	-2.77213600	2.33432900	0.07244100
N	-3.32847800	3.34536700	0.01938200
C	-2.84062100	-2.37032900	-0.38905500
N	-3.43424800	-3.33025300	-0.63568800
H	-0.43866600	-2.32734100	0.62104000
H	-0.39958600	2.01398200	1.08346400
C	1.23057900	-0.04007400	-0.46919800
C	2.26975000	-1.15952100	-0.29549700
C	1.92567400	1.31347500	-0.27629500
C	0.59456400	-0.20315000	-1.85469500
O	2.03748600	-2.13654000	0.37384900
C	3.58373100	-1.00329700	-1.02481000
O	2.72805500	1.43694200	0.62181500
C	1.56900400	2.45921800	-1.18492900

H	0.23779800	-1.23299100	-1.98590500
H	-0.27273500	0.46523200	-1.95553000
H	1.30805800	0.02363000	-2.65831400
H	4.02887400	-1.99047200	-1.19230800
H	3.47245900	-0.46190800	-1.97361400
H	4.25110800	-0.41390300	-0.37937000
H	1.92969500	2.25047300	-2.20311700
H	0.47769500	2.57591300	-1.24462000
H	2.03636900	3.37641600	-0.81023900

Py-3-426-R2-Nu3

C	0.38115000	-0.60689500	-0.60675000
C	1.77381900	-1.16123900	-0.43497400
C	0.51053600	0.87901500	-0.82602000
C	2.81242800	-0.33476500	-0.09013500
C	1.64957300	1.53997400	-0.44643000
N	2.81553200	1.01231400	-0.01528900
C	-0.25337900	-1.23527300	-1.79322600
N	-0.71707500	-1.72732700	-2.72917000
C	1.63405700	2.99363500	-0.56072100
N	1.61475700	4.14511100	-0.64928200
C	4.09511300	-0.97143600	0.18573900
N	5.10804100	-1.48165700	0.40421300
H	1.93947700	-2.23232700	-0.54692200
H	-0.33531700	1.42895200	-1.23698700
C	-0.58641500	-0.96919000	0.64771100
C	-1.96235000	-0.34127200	0.44895600
C	0.05994900	-0.37680000	1.83157400
C	-0.63475500	-2.49076900	0.85549200
C	-2.97941100	-1.04815900	-0.20517100
C	-2.21707100	0.97484200	0.85839500
N	0.56947600	0.06554700	2.76761200
H	0.35681900	-2.86572500	1.13896300
H	-1.34875600	-2.74688300	1.64917900
H	-0.94061300	-2.99555800	-0.07077000
C	-4.21747700	-0.45298700	-0.44511500
H	-2.81142900	-2.07178600	-0.53958300
C	-3.45491100	1.56942400	0.61785800
H	-1.43945400	1.53958500	1.37577900
C	-4.46022300	0.85731100	-0.03510700
H	-4.99669200	-1.02143700	-0.95493800
H	-3.63265200	2.59416300	0.94719600
H	-5.43029600	1.32061100	-0.22096800

Py-3-426-R2-Nu2

C	1.43382400	-0.26691000	0.82901800
C	1.89918500	1.16584900	0.94295600
C	2.47516700	-0.98962100	0.00496000
C	2.66968400	1.70037400	-0.05654900
C	3.20132100	-0.28974100	-0.92377000
N	3.27652000	1.04964700	-1.07413500

C	1.34018900	-0.89710000	2.16820400
N	1.32162000	-1.38250600	3.21426300
C	4.04488000	-1.06340500	-1.82598700
N	4.70807700	-1.67902800	-2.54379700
C	2.92327000	3.13430900	-0.00227700
N	3.11901800	4.27195700	0.03777600
H	1.51668200	1.78488000	1.75059000
H	2.57123400	-2.06988100	0.09827100
C	0.02159600	-0.34154900	0.12771900
Br	-0.44587000	-2.13199100	-0.44121800
S	-1.35325300	0.31406300	1.15454300
H	0.06536900	0.28457100	-0.77296000
O	-1.77986100	-0.72230100	2.08607500
O	-0.91332000	1.61060900	1.66492600
C	-2.65995500	0.59121300	-0.01906200
C	-3.65749300	-0.37197300	-0.15614300
C	-2.65833900	1.77618800	-0.75401500
C	-4.68116700	-0.14073500	-1.07314300
H	-3.62474300	-1.27635200	0.45260400
C	-3.68630000	1.99053600	-1.66950500
H	-1.87131000	2.51637200	-0.59967400
C	-4.69169500	1.03399900	-1.82719300
H	-5.47408600	-0.87890300	-1.19718800
H	-3.70655700	2.90960200	-2.25584100
H	-5.49529400	1.20882600	-2.54390200

Py-3-426-R2-Nu4

C	0.44615400	-0.62763600	0.13498000
C	1.55732900	-0.99698400	-0.89743800
C	-0.86052500	-1.29982300	-0.23723300
C	2.81800300	-0.17208100	-0.82030300
H	1.10194900	-0.87575000	-1.88987900
H	1.80015400	-2.06633100	-0.76696600
C	0.18400100	0.86163000	0.20379500
C	-2.04677000	-0.61897700	-0.21082800
C	3.61782600	-0.23007600	0.45388900
C	3.19785400	0.58686700	-1.85399100
C	-1.09032500	1.36149000	0.19365500
N	-2.25702500	0.69444500	0.03822800
H	4.58861500	0.26830300	0.33406800
H	3.78501300	-1.27120400	0.76983000
H	3.08074900	0.26204100	1.28012300
H	2.60268300	0.63683100	-2.76887500
H	4.11628800	1.17807200	-1.81517700
C	0.86552300	-1.14023600	1.47863400
N	1.13773000	-1.54531500	2.52660100
C	-1.22695100	2.80757500	0.33063400
N	-1.33237200	3.95270900	0.44172300
C	-3.24852300	-1.38194900	-0.52897400
N	-4.19893300	-1.98902500	-0.77993500
H	-0.83787200	-2.35912600	-0.49826200
H	1.03504400	1.53867800	0.28789800

Py-3-426-R3

C	1.17229700	0.50146800	0.00000000
C	1.08745800	-0.88857600	0.00000000
C	0.00000000	1.25355200	0.00000000
C	2.53349900	1.13480900	0.00000000
C	-0.18379700	-1.46941800	0.00000000
C	-1.21799000	0.56526700	0.00000000
O	2.47191100	2.45942400	0.00000000
O	3.55763400	0.50451600	0.00000000
C	-0.36046700	-2.96877600	0.00000000
C	-2.53397900	1.30523500	0.00000000
C	3.72042100	3.14769500	0.00000000
O	0.81194700	-3.59819100	0.00000000
O	-1.42286400	-3.52503700	0.00000000
O	-2.35387300	2.62401500	0.00000000
O	-3.60857400	0.77324800	0.00000000
H	3.48117200	4.21423000	0.00000000
H	4.29870400	2.88205400	0.89399000
H	4.29870400	2.88205400	-0.89399000
C	0.75751200	-5.02121800	0.00000000
C	-3.53744700	3.41602400	0.00000000
H	0.23128500	-5.38099800	0.89350500
H	0.23128500	-5.38099800	-0.89350500
H	1.79432300	-5.36794800	0.00000000
H	-4.13785700	3.20170500	0.89338200
H	-4.13785700	3.20170500	-0.89338200
H	-3.20891900	4.45875800	0.00000000
N	-1.30580300	-0.75905100	0.00000000
H	1.98947500	-1.49871900	0.00000000
H	0.02581000	2.34135800	0.00000000

Py-3-426-R3-Nu1

C	0.44689800	-0.35561700	0.29298400
C	0.29803300	1.12855000	0.35947000
C	-0.91966100	-0.95560200	0.36107300
C	1.29532700	-0.93727600	1.42498900
C	-0.90022600	1.73904500	0.08097100
C	-2.03902900	-0.20120900	0.10444200
O	1.83474100	-0.02060300	2.22421800
O	1.43673000	-2.12289000	1.60856300
C	-0.95575200	3.24221300	0.00969800
C	-3.38365900	-0.87794600	0.06750000
C	2.70265200	-0.51245100	3.23314600
O	0.26165800	3.81604000	0.03417700
O	-1.96136000	3.89944800	-0.07458000
O	-3.32984700	-2.18363100	0.37778400
O	-4.42298400	-0.33623900	-0.21070700
H	3.03740200	0.35790600	3.80553900
H	2.17760000	-1.22120800	3.88670200
H	3.56161800	-1.01913400	2.77187000

C	0.27754600	5.23247200	-0.00121800
C	-4.56386800	-2.87787900	0.34520900
H	-0.18167100	5.60364800	-0.92755400
H	-0.27485400	5.64970900	0.85175300
H	1.32897200	5.53265400	0.04590800
H	-5.00443500	-2.84099400	-0.66072400
H	-5.27874600	-2.43690100	1.05353000
H	-4.34499400	-3.91350900	0.62328400
N	-2.08870300	1.13034400	-0.10087800
H	1.17432800	1.73503000	0.57964800
H	-1.00796200	-2.02136500	0.55686700
C	1.25122000	-0.80457900	-1.11912700
C	1.40358100	-2.33032000	-1.16123900
C	2.56772600	-0.05566700	-1.04580400
C	0.36107300	-0.39078900	-2.28716100
O	0.42936000	-3.00052900	-1.43109900
C	2.74652700	-2.99435700	-0.95801700
O	3.39705300	-0.34350500	-0.20431500
C	2.77973400	1.12827100	-1.95483600
H	-0.57671600	-0.95442500	-2.22834500
H	0.12908100	0.68127900	-2.24464800
H	0.83898000	-0.61581900	-3.25246200
H	2.61136000	-4.07481600	-1.08338100
H	3.46690400	-2.61856200	-1.69968600
H	3.14745100	-2.76433900	0.03415500
H	2.65609200	0.85431800	-3.01107400
H	2.01114300	1.88316700	-1.72107000
H	3.77781000	1.54507300	-1.77896800

Py-3-426-R3-Nu3

C	0.01812100	-0.49045200	0.30022900
C	1.44212700	-0.95329700	0.30663600
C	0.01635700	0.99970100	0.41413400
C	-0.76546800	-1.18370000	1.41491800
C	2.46706900	-0.08713400	0.01741000
C	1.13994600	1.72947100	0.10903900
O	-1.61695300	-0.37741800	2.04900100
O	-0.66304700	-2.35497700	1.68520000
C	3.86797500	-0.62727800	-0.10435300
C	1.04690000	3.23280000	0.11166400
C	-2.44717300	-0.98995500	3.02423900
O	3.96246000	-1.92913300	0.21329200
O	4.82918300	0.01008500	-0.45197700
O	-0.21958200	3.68151200	0.18290900
O	1.98119000	3.99010600	0.04957800
H	-3.06716400	-0.19338700	3.44640600
H	-3.07994900	-1.75645100	2.55489700
H	-1.83991300	-1.46076800	3.80822000
C	5.25529400	-2.49878700	0.11052300
C	-0.37369800	5.08920900	0.22532400
H	5.63788300	-2.41364200	-0.91577800
H	5.95843600	-1.99311400	0.78662200

H	5.15371900	-3.55204600	0.39005700
H	0.04826400	5.55391400	-0.67618900
H	0.13413200	5.51135000	1.10348700
H	-1.44948900	5.28258400	0.28264600
N	2.37146300	1.24871400	-0.15321500
H	1.64028100	-2.00641100	0.49542200
H	-0.90191600	1.50635800	0.69732400
C	-0.76332400	-0.98434000	-1.08500000
C	-2.25027200	-0.69477400	-0.96640300
C	-0.53242200	-2.42704700	-1.24774500
C	-0.10274300	-0.28903300	-2.28100900
C	-2.74410900	0.58701100	-1.24660200
C	-3.14360600	-1.66301900	-0.48548600
N	-0.31660600	-3.54983800	-1.41339000
H	0.95282700	-0.58527500	-2.34010700
H	-0.61092300	-0.56207200	-3.21624400
H	-0.14018700	0.79888400	-2.14936200
C	-4.09076200	0.89015100	-1.04398300
H	-2.07294600	1.36422700	-1.61161000
C	-4.48961200	-1.36032400	-0.28731300
H	-2.77672800	-2.66600600	-0.25650000
C	-4.96948200	-0.07982400	-0.56368600
H	-4.45372400	1.89514600	-1.26586200
H	-5.16632000	-2.13199000	0.08391000
H	-6.02299700	0.15889900	-0.41034400

Py-3-426-R3-Nu2

C	0.85904900	-0.34715600	0.84275400
C	2.16750100	0.11458900	0.25197500
C	0.16860000	0.91931300	1.28000300
C	1.09256600	-1.33212400	1.97867500
C	2.14605200	1.24761400	-0.52339600
C	0.25710200	2.00755100	0.44522500
O	1.66295900	-0.75249400	3.03239800
O	0.82683800	-2.50618100	1.93843100
C	3.33239300	1.58288000	-1.38226500
C	-0.63147200	3.19662100	0.67537900
C	1.93269400	-1.60231300	4.13928100
O	4.37309500	0.75667200	-1.18005400
O	3.38323100	2.48250400	-2.18145000
O	-1.57453700	2.97018600	1.61169100
O	-0.55252600	4.25309300	0.10353300
H	2.39206700	-0.97215300	4.90640700
H	1.00218100	-2.04717600	4.51583400
H	2.61731800	-2.40954100	3.84702800
C	5.52678900	1.01489600	-1.96088400
C	-2.42400900	4.06500100	1.91076200
H	5.29938200	0.92404800	-3.03184800
H	5.90717300	2.02790800	-1.77104000
H	6.27308000	0.26951400	-1.66883100
H	-2.97925100	4.38166000	1.01715500
H	-1.84046800	4.92016700	2.27804400

H	-3.11599700	3.71881600	2.68476200
N	1.14285500	2.15279400	-0.56380800
H	3.05731300	-0.50642300	0.33397300
H	-0.47929500	0.91372900	2.15190600
C	-0.01164800	-1.02472300	-0.25580400
Br	0.87110400	-2.40953000	-1.28516300
S	-1.64390400	-1.63687500	0.39048800
H	-0.26070600	-0.24541800	-0.98676500
O	-1.82128500	-3.04984800	0.07243300
O	-1.79411800	-1.19233900	1.77729000
C	-2.80657600	-0.70920800	-0.59678900
C	-3.35178200	-1.30729100	-1.72957600
C	-3.12144800	0.59410800	-0.21456900
C	-4.24285600	-0.56906000	-2.50855300
H	-3.08180000	-2.33316100	-1.98337700
C	-4.01105300	1.31920300	-1.00487800
H	-2.66612300	1.03031800	0.67759800
C	-4.56860200	0.73884500	-2.14697900
H	-4.68435700	-1.01779700	-3.39917500
H	-4.27164900	2.34150400	-0.72741200
H	-5.26407000	1.31278400	-2.76104500

Py-3-426-R3-Nu4

C	0.33850900	-0.95719700	-0.24296500
C	0.82320300	-1.52842600	-1.61781400
C	0.87790700	0.42393700	0.03065100
C	2.31293000	-1.46662800	-1.83693600
H	0.47565000	-2.57385400	-1.67989000
H	0.31268600	-0.94930600	-2.39944700
C	-1.17356600	-0.86799800	-0.21380700
C	0.71664300	-1.98790800	0.82894400
C	0.06921600	1.52672700	0.07417900
C	3.18464800	-2.21876700	-0.86525300
C	2.84094900	-0.74974800	-2.83562200
C	-1.82945900	0.33447600	-0.14614000
O	1.24460400	-1.45362600	1.93430000
O	0.52129200	-3.17621500	0.72483000
C	0.69949300	2.87881600	0.28246300
H	3.13571000	-1.75726800	0.13545000
H	2.84116500	-3.25747300	-0.74918900
H	4.23513300	-2.21710900	-1.18473700
H	2.20525400	-0.18813100	-3.52446200
H	3.92180600	-0.70462900	-2.99384700
C	-3.33480000	0.33781900	-0.22246300
C	1.56942000	-2.36590400	2.97184000
O	2.04480900	2.84285500	0.32534700
O	0.09840000	3.91655400	0.40189700
O	-3.87735200	-0.89232200	-0.15977900
O	-4.02333700	1.32040500	-0.33249700
H	1.98153500	-1.76951300	3.79155100
H	0.67370900	-2.90627100	3.30589000
H	2.31091100	-3.09813300	2.62253700

C	2.68860900	4.08681800	0.52798200
C	-5.28935700	-0.94836500	-0.24764100
H	2.44425600	4.78959700	-0.28055700
H	2.38016700	4.53638000	1.48204400
H	3.76416200	3.88301000	0.53764400
H	-5.63873000	-0.53389600	-1.20351800
H	-5.75428300	-0.37706300	0.56753500
H	-5.56426400	-2.00520100	-0.17227000
N	-1.28230400	1.56191800	-0.02610600
H	1.95388400	0.54552800	0.14836000
H	-1.73603300	-1.79721600	-0.29765400

Py-3-426-R4

C	0.00000000	0.00000000	1.37098300
C	0.00000000	1.21341800	0.69364200
C	0.00000000	-1.21341800	0.69364200
C	0.00000000	1.11920400	-0.69252500
C	0.00000000	-1.11920400	-0.69252500
N	0.00000000	0.00000000	-1.38032700
F	0.00000000	0.00000000	2.69165900
F	0.00000000	-2.23509800	-1.40133000
F	0.00000000	2.23509800	-1.40133000
H	0.00000000	-2.17037100	1.21100000
H	0.00000000	2.17037100	1.21100000

Py-3-426-R4-Nu1

C	0.17016000	-0.10035900	-0.40706100
C	1.02124400	-1.26017300	-0.06961100
C	0.96712400	1.14417800	-0.37195100
C	2.36875900	-1.10123700	0.06441600
C	2.32321700	1.08356800	-0.21602400
N	3.08204800	0.01926200	0.00332300
F	-0.36082200	-0.28125100	-1.74553800
F	3.00688500	2.24575300	-0.26740900
F	3.10656400	-2.20344900	0.31346700
H	0.50165400	2.11027000	-0.56757600
H	0.57988600	-2.24987700	0.04106400
C	-1.17194800	-0.03340100	0.46735900
C	-2.01722600	-1.27153900	0.09819300
C	-1.86616200	1.28959100	0.11408600
C	-0.79476600	-0.12002700	1.94519400
O	-1.84870000	-2.29620500	0.72381800
C	-3.02863300	-1.23371000	-1.02070600
O	-2.41204100	1.46407500	-0.95113800
C	-1.81139500	2.41206100	1.12719700
H	-0.34823200	-1.09903200	2.14497100
H	-0.05791700	0.65253400	2.20077800
H	-1.67809300	-0.00706600	2.59054200
H	-3.39479300	-2.25442700	-1.18051800
H	-3.86412200	-0.57564000	-0.74366000
H	-2.57643000	-0.81309800	-1.92274900

H	-2.33892800	2.12762200	2.04835800
H	-0.76930600	2.62586900	1.40296500
H	-2.27802700	3.30184800	0.68989600

Py-3-426-R4-Nu3

C	0.68294900	0.30658300	-0.34502200
C	0.83302100	-1.15603000	-0.22993000
C	1.98162900	0.97935300	-0.14308400
C	2.08348300	-1.70265400	-0.16198000
C	3.12580300	0.23927200	-0.08257900
N	3.25434700	-1.08305600	-0.09386000
F	0.17982800	0.64297300	-1.67022100
F	4.29515200	0.90235700	0.02733600
F	2.17376400	-3.04830500	-0.13745700
H	2.03646000	2.06801700	-0.10437300
H	-0.03954900	-1.80655000	-0.27541600
C	-0.47440800	0.89121100	0.56935700
C	-1.79852400	0.18110400	0.27841500
C	-0.62258400	2.32027400	0.23012300
C	-0.06244700	0.80478900	2.04413200
C	-2.27335200	-0.83331200	1.11658600
C	-2.54223700	0.50629400	-0.86436200
N	-0.71685500	3.44785600	0.00010200
H	0.81315900	1.44031700	2.22393900
H	-0.88444900	1.12128100	2.70075400
H	0.22181600	-0.22890500	2.28142600
C	-3.45751700	-1.51079500	0.81729100
H	-1.72000900	-1.10986800	2.01366500
C	-3.72611200	-0.16412100	-1.16007600
H	-2.17741500	1.28850300	-1.53064600
C	-4.18907900	-1.17878900	-0.32029400
H	-3.80768600	-2.30074500	1.48377500
H	-4.29082900	0.10729700	-2.05351200
H	-5.11637100	-1.70525600	-0.55168600

Py-3-426-R4-Nu2

C	1.59836200	-0.00322000	0.41199900
C	2.03456000	1.39098700	0.16816900
C	2.57235700	-0.94394100	-0.17265100
C	3.28211200	1.63482400	-0.32881700
C	3.77509000	-0.48648000	-0.63196700
N	4.19743100	0.76501700	-0.73880400
F	1.46787700	-0.25443000	1.83688700
F	4.66040700	-1.40510300	-1.06617900
F	3.65402300	2.92372000	-0.46151200
H	2.38248300	-2.01458600	-0.14948800
H	1.39118000	2.22017600	0.44913000
C	0.13154100	-0.22796000	-0.06099500
Br	-0.38001300	-2.08635000	-0.30891300
S	-1.09930000	0.50469300	1.10038200
H	-0.03483500	0.26312500	-1.02626900

O	-1.36896700	-0.40662100	2.20472200
O	-0.67653400	1.87737500	1.37407100
C	-2.57465900	0.59423900	0.09878500
C	-3.55598100	-0.38263600	0.24926400
C	-2.71496700	1.65537400	-0.79452900
C	-4.70711900	-0.29816900	-0.53254500
H	-3.40953300	-1.18598600	0.97219100
C	-3.86821600	1.72497100	-1.57336800
H	-1.93621500	2.41666200	-0.86656600
C	-4.85904900	0.74949700	-1.44222500
H	-5.48846000	-1.05196600	-0.42959400
H	-3.99717600	2.54633000	-2.27903800
H	-5.76115100	0.80967500	-2.05274800

Py-3-426-R4-Nu4

C	0.21084700	-0.52527200	0.14341900
C	1.46101000	-0.93209500	-0.66041700
C	-0.99104200	-1.30492500	-0.22503700
C	2.68738800	-0.08791500	-0.41878500
H	1.20247800	-0.89740300	-1.72759200
H	1.67906700	-1.98251800	-0.40017900
C	-0.09874200	0.92006500	0.12009400
C	-2.21435400	-0.70305000	-0.20399000
C	3.27423900	-0.09266500	0.96707900
C	3.22302500	0.64386200	-1.40323600
C	-1.40167400	1.32536600	0.11157900
N	-2.49639200	0.58324000	-0.02031100
H	4.20888400	0.48301400	1.00663400
H	3.47404100	-1.12387700	1.29766300
H	2.55473300	0.32185500	1.68602000
H	2.78650200	0.65234000	-2.40522800
H	4.11331900	1.25599100	-1.23594300
F	0.59229300	-0.90571200	1.52001400
F	-1.64999800	2.65164300	0.20449200
F	-3.30182700	-1.47245000	-0.43745700
H	0.69985800	1.65972200	0.18365400
H	-0.91254200	-2.37336500	-0.43052900

Py-3-426-R5

C	0.00000000	0.00000000	1.38857600
C	0.00000000	1.21406500	0.71937700
C	0.00000000	-1.21406500	0.71937700
C	0.00000000	1.11636400	-0.66808900
C	0.00000000	-1.11636400	-0.66808900
N	0.00000000	0.00000000	-1.35311200
N	0.00000000	0.00000000	2.87293400
O	0.00000000	1.07375900	3.41536500
O	0.00000000	-1.07375900	3.41536500
N	0.00000000	2.37930600	-1.47212200
O	0.00000000	2.27253000	-2.66513400
O	0.00000000	3.40712800	-0.84107900

N	0.00000000	-2.37930600	-1.47212200
O	0.00000000	-2.27253000	-2.66513400
O	0.00000000	-3.40712800	-0.84107900
H	0.00000000	-2.17020400	1.23880200
H	0.00000000	2.17020400	1.23880200

Py-3-426-R5-Nu1

C	0.33543400	-0.02568900	0.17953100
C	-0.50874300	-1.22233000	-0.05815000
C	-0.47320600	1.20230200	0.00915200
C	-1.86679900	-1.08231500	-0.08691200
C	-1.83334900	1.10775400	-0.04729600
N	-2.59818700	0.02459500	-0.06239900
N	0.78289200	-0.12108500	1.73873600
O	0.24218600	0.60322900	2.52602600
O	1.55244000	-1.01050200	2.03073600
N	-2.66751300	-2.34391600	-0.24374500
O	-3.84696500	-2.23835000	-0.46135200
O	-2.08325000	-3.39972300	-0.14388700
N	-2.59640900	2.39876100	-0.12799800
O	-3.79395500	2.35169100	-0.01871700
O	-1.96541300	3.41892800	-0.29662200
H	0.00093600	2.17995400	-0.02296900
H	-0.04981200	-2.20191800	-0.15533300
C	1.67686200	-0.00450800	-0.64617600
C	2.55507900	-1.26784600	-0.38817100
C	2.47493000	1.25967400	-0.28156900
C	1.29945600	-0.03869100	-2.13327900
O	2.18906100	-2.33192400	-0.82366900
C	3.89430400	-1.10591200	0.28528100
O	2.47678800	1.67201400	0.85479500
C	3.25442700	1.96426400	-1.36357600
H	0.72641100	-0.95227900	-2.33222500
H	0.67891100	0.82885900	-2.39730700
H	2.19345600	-0.05115300	-2.77091300
H	4.32783100	-2.09702000	0.45786000
H	4.56202400	-0.52958100	-0.37457000
H	3.79057400	-0.55760600	1.22777600
H	3.87968700	1.26345300	-1.93379900
H	2.55329800	2.42288000	-2.07660800
H	3.87371500	2.74280000	-0.90557200

Py-3-426-R5-Nu3

C	0.08744600	-0.38136600	0.08969400
C	0.38850000	1.06028400	-0.00922900
C	1.31806700	-1.18613000	-0.05652800
C	1.69078600	1.46857800	0.03442300
C	2.53048700	-0.55818600	-0.00032000
N	2.79718700	0.73831800	0.06824700
N	-0.52555400	-0.66283600	1.56657700
O	-0.88997000	0.28082400	2.21588100

O	-0.57891000	-1.81564200	1.92328200
N	1.93369400	2.95112900	0.01069500
O	3.06443800	3.33695200	0.15237200
O	0.97755800	3.67688900	-0.14888000
N	3.74959800	-1.43459800	-0.07406700
O	4.82780800	-0.90126800	-0.04200800
O	3.57929600	-2.63006200	-0.15960100
H	1.25934300	-2.26655200	-0.16339300
H	-0.41293600	1.79131000	-0.06963600
C	-1.09328200	-0.84761800	-0.87012400
C	-2.42482700	-0.18418400	-0.50329900
C	-1.25082300	-2.31518400	-0.79388300
C	-0.66126100	-0.54432400	-2.31670500
C	-2.79840800	1.03588000	-1.07861600
C	-3.27963000	-0.77258900	0.43807000
N	-1.37798600	-3.46162800	-0.82048000
H	0.23750800	-1.12345600	-2.56501600
H	-1.46871300	-0.80482400	-3.01345600
H	-0.41241100	0.51879200	-2.42251700
C	-3.99217700	1.65758400	-0.71186500
H	-2.16219800	1.51480400	-1.82258000
C	-4.47363000	-0.15345400	0.80067800
H	-3.00468400	-1.72446900	0.89810700
C	-4.83413500	1.06567000	0.22753800
H	-4.26436700	2.60921500	-1.17044300
H	-5.12500600	-0.62951900	1.53484700
H	-5.76956100	1.55070000	0.50992300

Py-3-426-R5-Nu2

C	0.84665000	-0.34161800	0.52854300
C	1.06709800	1.12609800	0.62190300
C	1.97131600	-0.97083600	-0.21068800
C	2.19910500	1.67858500	0.10272100
C	3.02219800	-0.20816300	-0.63080900
N	3.21273400	1.09790500	-0.52498700
N	0.86167400	-0.96072200	2.00927700
O	1.71535800	-0.55086400	2.74625800
O	0.07233800	-1.83775800	2.26540000
N	2.34302000	3.16692800	0.25354400
O	3.42364000	3.64879700	0.03544200
O	1.36472100	3.79765200	0.58721500
N	4.11880600	-0.93186000	-1.36379300
O	4.94507300	-0.26675700	-1.93144200
O	4.10674500	-2.14191700	-1.34403600
H	1.97047900	-2.04297100	-0.38475600
H	0.33150500	1.74961900	1.11965100
C	-0.55204300	-0.68630900	-0.06468500
Br	-0.78574400	-2.47343700	-0.75862400
S	-1.99764900	-0.26244600	1.03540600
H	-0.67302400	-0.03000800	-0.93302500
O	-2.72359900	-1.45554700	1.44117900
O	-1.48903700	0.66875900	2.04292900

C	-3.03937400	0.64595500	-0.08820000
C	-4.04557500	-0.04061500	-0.76419900
C	-2.81276300	2.00953000	-0.27020900
C	-4.84572400	0.66670600	-1.66049500
H	-4.19617300	-1.10479900	-0.57861200
C	-3.62150700	2.70212600	-1.16869600
H	-2.02103200	2.51706600	0.28408500
C	-4.63216700	2.03117700	-1.86110300
H	-5.64060800	0.15070300	-2.19991900
H	-3.46470000	3.76971800	-1.32544000
H	-5.26183500	2.57962500	-2.56300300

Py-3-426-R5-Nu4

C	0.71107900	-0.57646400	-0.16914400
C	1.90830200	-1.05761000	-1.00674800
C	-0.53383600	-1.32019400	-0.45976900
C	3.15875400	-0.22132600	-0.89187000
H	1.57177100	-1.07065800	-2.05222200
H	2.11843000	-2.09737200	-0.71029400
C	0.45061700	0.87341300	-0.18061900
C	-1.72942700	-0.68978200	-0.25504900
C	3.81088500	-0.13509200	0.46249200
C	3.65741800	0.41300000	-1.95816700
C	-0.83586500	1.30424400	-0.01603700
N	-1.95356400	0.59016900	0.01543700
H	4.79465600	0.34747600	0.40013400
H	3.92823100	-1.13616600	0.90557900
H	3.18876700	0.44843700	1.15921400
H	3.16718000	0.35448400	-2.93292700
H	4.57258000	1.00602500	-1.88728900
N	1.11608400	-1.01177900	1.38765100
O	1.01601900	-0.18267900	2.25596400
O	1.40222800	-2.17169600	1.57732200
N	-2.97406400	-1.51082700	-0.42460900
O	-4.03389200	-0.98021200	-0.21387100
O	-2.84430800	-2.66751900	-0.76049500
N	-1.04928200	2.78332800	0.10891400
O	-2.13679500	3.16916200	0.45284500
O	-0.11269000	3.51125000	-0.13657700
H	1.26587100	1.58656600	-0.27969800
H	-0.49375900	-2.36261300	-0.76628100

Py-3-435-R1

C	-0.05158000	-0.06743700	0.00000000
C	-0.77930400	-0.05012100	1.19788000
C	-0.77930400	-0.05012100	-1.19788000
C	-2.17580900	-0.09770800	1.13477900
C	-2.17580900	-0.09770800	-1.13477900
N	-2.85886900	-0.14293700	0.00000000
C	-0.15673800	0.05702500	-2.58096300
C	1.47680300	-0.07766500	0.00000000

C	-0.15673800	0.05702500	2.58096300
F	0.33576900	-1.09899600	-3.01245400
F	-1.07190800	0.44446800	-3.46960900
F	0.81840400	0.96403300	-2.60366800
F	1.95291500	-0.71266500	-1.06483100
F	1.96444100	1.15626600	0.00000000
F	1.95291500	-0.71266500	1.06483100
F	0.81840400	0.96403300	2.60366800
F	0.33576900	-1.09899600	3.01245400
F	-1.07190800	0.44446800	3.46960900
H	-2.75513200	-0.09434700	-2.06031500
H	-2.75513200	-0.09434700	2.06031500

Py-3-435-R1-Nu1

C	-0.08805400	-0.17593000	-0.04439100
C	-1.40468000	-0.31952500	0.74904800
C	1.01283800	-0.57105500	0.95185300
C	-1.40037500	-0.19144900	2.12430100
C	0.81709400	-0.41630400	2.30371500
N	-0.33692700	-0.14065500	2.93243400
C	2.33723900	-1.13252200	0.53783500
C	-0.08886600	-1.17832800	-1.21969100
C	-2.73005400	-0.64487500	0.13957700
F	2.96122900	-0.46172600	-0.45187900
F	3.21666000	-1.15287200	1.55463800
F	2.27631500	-2.40682100	0.09658400
F	1.01653600	-1.08082600	-1.96560700
F	-0.16573300	-2.43423500	-0.78144700
F	-1.11582500	-1.01699400	-2.06227300
F	-2.81214300	-1.89862100	-0.35233600
F	-3.11455000	0.15459100	-0.88515500
F	-3.73336600	-0.55241600	1.02929500
H	1.67665700	-0.57744400	2.96120800
H	-2.36892800	-0.16692400	2.63175800
C	0.10879100	1.34558300	-0.63338800
C	1.60025700	1.71490600	-0.92971900
C	-0.43217000	2.26691900	0.48975600
C	-0.63689200	1.61256700	-1.95043800
O	1.97736000	1.71488000	-2.07793600
C	2.55030600	2.17931200	0.15196200
O	0.21071500	2.48072900	1.48867600
C	-1.78119000	2.92462800	0.30347800
H	-0.17990700	1.04465700	-2.76515800
H	-1.69594400	1.35892900	-1.88921500
H	-0.53738600	2.67623300	-2.20860400
H	3.56443200	2.12403600	-0.25930400
H	2.31083000	3.22414200	0.39734400
H	2.45566600	1.60648500	1.07825500
H	-1.70952700	3.69830000	-0.47527900
H	-2.54314200	2.20497300	-0.01617100
H	-2.06645400	3.39204200	1.25246100

Py-3-435-R1-Nu3

C	0.56176200	0.15293400	-0.02476100
C	1.88291400	0.00097700	0.72757300
C	-0.31807200	1.00928400	0.89233300
C	1.87357900	-0.02789000	2.10451300
C	-0.15555000	0.91031800	2.25468000
N	0.85477600	0.31185000	2.90602700
C	-1.40076400	1.93350700	0.41873900
C	0.78257300	0.87943800	-1.36276800
C	3.18364700	-0.28918300	0.06165300
F	-2.32747300	1.39674000	-0.39185200
F	-2.09467600	2.45060700	1.44853500
F	-0.93117100	3.00279100	-0.26158400
F	-0.36447000	1.00753600	-2.04591700
F	1.28501400	2.09752500	-1.18490300
F	1.62797800	0.24601600	-2.18490400
F	3.64960000	0.72441400	-0.69772900
F	3.16085100	-1.36497300	-0.75920100
F	4.16259100	-0.54426800	0.94375600
H	-0.89913400	1.39428900	2.89413700
H	2.79269000	-0.32808600	2.61560900
C	-0.13878700	-1.32932400	-0.34969600
C	-1.66852600	-1.29418700	-0.26060700
C	0.32306400	-2.25782200	0.70166400
C	0.29951300	-2.00125800	-1.67346900
C	-2.46447000	-1.24342700	-1.40896700
C	-2.30130100	-1.29925500	0.98923200
N	0.65864600	-3.05768800	1.46336400
H	1.38183900	-2.14691200	-1.69965700
H	-0.19774400	-2.97765100	-1.74903600
H	0.01376500	-1.39945800	-2.54130600
C	-3.85327300	-1.17019300	-1.31037900
H	-2.01001500	-1.23677600	-2.39829700
C	-3.68856000	-1.22448200	1.09042100
H	-1.69761000	-1.36562200	1.89627600
C	-4.47119200	-1.15319300	-0.06160300
H	-4.45459300	-1.12420800	-2.21988000
H	-4.15850400	-1.22606300	2.07523300
H	-5.55819000	-1.09360400	0.01401400

Py-3-435-R1-Nu2

C	-1.21899600	0.23953200	-0.15739600
C	-1.57075000	1.57566600	0.50983700
C	-2.20479100	-0.78277900	0.37503900
C	-2.43191000	1.63846700	1.58090500
C	-3.00700500	-0.52292100	1.45939300
N	-3.14022900	0.63692600	2.11896700
C	-2.28427700	-2.15420100	-0.19919200
C	-1.35854500	0.40909900	-1.69379800
C	-0.98053800	2.86784400	0.04661500
F	-1.10030100	-2.59622400	-0.65130300
F	-2.70497300	-3.06300100	0.69803000

F	-3.14256900	-2.27269800	-1.23595800
F	-1.37582900	-0.73360000	-2.36806000
F	-2.50223100	1.02725400	-1.98768500
F	-0.35919200	1.15256900	-2.19200100
F	-1.47958700	3.32121100	-1.12110400
F	0.36138700	2.82864500	-0.15063300
F	-1.17941000	3.86139500	0.92428700
H	-3.62211700	-1.34998800	1.83050000
H	-2.57579900	2.61742400	2.04791200
C	0.31422600	-0.05077300	0.20363900
Br	0.50981000	-0.32208600	2.10781200
S	1.42468700	-1.29627900	-0.61931900
H	0.86509600	0.86679900	-0.01584900
O	1.45132700	-2.56760400	0.09132300
O	1.16877000	-1.25538300	-2.05507600
C	3.00708700	-0.50567600	-0.35377000
C	3.84853900	-0.98263900	0.64675100
C	3.36496900	0.55615500	-1.18409200
C	5.08685400	-0.36638700	0.82563900
H	3.52852000	-1.82293100	1.26389800
C	4.60222700	1.16594100	-0.99078300
H	2.68209500	0.89076600	-1.96785600
C	5.45899900	0.70410500	0.01148600
H	5.76289800	-0.72431900	1.60293700
H	4.90148300	1.99915100	-1.62748100
H	6.42811100	1.18343600	0.15709500

Py-3-435-R1-Nu4

C	0.14500500	-0.00724100	-0.17950700
C	-0.00538300	1.01893400	-1.36519000
C	1.46967000	-0.74286000	-0.40432600
C	-1.23692600	1.88676100	-1.45843500
H	0.04988400	0.37759400	-2.25672000
H	0.89683300	1.64814600	-1.35960400
C	-0.93028400	-1.09893000	-0.22181400
C	1.47105500	-1.79351600	-1.28967200
C	-1.14694900	3.27527500	-0.88095700
C	-2.33222700	1.45263000	-2.08986500
C	-0.74861600	-2.13353800	-1.10765200
N	0.39046400	-2.44470000	-1.75660600
H	-2.10060400	3.81185600	-0.97590900
H	-0.37052600	3.85216600	-1.40946300
H	-0.86085200	3.25572800	0.17943000
H	-2.36568000	0.45340800	-2.53195300
H	-3.23222500	2.06936400	-2.15901300
C	-2.20072800	-1.07616900	0.55840100
C	0.14192600	0.73873300	1.15343900
C	2.78050800	-0.17248600	-0.00115200
F	-3.00060900	-0.01339500	0.35098800
F	-2.96987300	-2.14880600	0.29084400
F	-2.00408300	-1.10708800	1.89597900
F	-1.04949200	1.29246500	1.42249200

F	0.44487800	-0.04217000	2.18862800
F	1.01784600	1.75706200	1.15513900
F	2.95021800	-0.04938600	1.33105300
F	3.01416700	1.06962000	-0.49421600
F	3.81442000	-0.91914400	-0.42461700
H	-1.58686400	-2.81529500	-1.28368600
H	2.43723900	-2.17804800	-1.63305500

Py-3-435-R2

C	0.00000000	0.00000000	0.33539300
C	0.00000000	1.20213000	-0.38697500
C	0.00000000	-1.20213000	-0.38697500
C	0.00000000	1.14277000	-1.78895000
C	0.00000000	-1.14277000	-1.78895000
N	0.00000000	0.00000000	-2.46218400
C	0.00000000	0.00000000	1.77494400
N	0.00000000	0.00000000	2.92805600
C	0.00000000	-2.47243400	0.28681400
N	0.00000000	-3.49545000	0.81991700
C	0.00000000	2.47243400	0.28681400
N	0.00000000	3.49545000	0.81991700
H	0.00000000	-2.07057900	-2.36632400
H	0.00000000	2.07057900	-2.36632400

Py-3-435-R2-Nu1

C	0.40367300	-0.30234300	0.31573100
C	1.87153100	-0.28778200	-0.11567800
C	-0.03726000	-1.76461000	0.17489800
C	2.36450700	-1.25857300	-0.96567400
C	0.60035000	-2.62505600	-0.69889000
N	1.74181300	-2.38342100	-1.35600700
C	0.29334400	0.07348600	1.74567300
N	0.22360000	0.28173500	2.87807800
C	-1.16199400	-2.23745500	0.90449200
N	-2.09054100	-2.62211000	1.48592700
C	2.79093200	0.70429600	0.33289500
N	3.61099900	1.44935400	0.67714500
H	0.15352400	-3.61666900	-0.84189800
H	3.39096200	-1.12573300	-1.32849500
C	-0.51769300	0.65886800	-0.56272200
C	-0.27571300	2.14049500	-0.22977700
C	-1.98944500	0.37166500	-0.20086200
C	-0.21220800	0.44182600	-2.05016500
O	0.63069100	2.49111400	0.48207500
C	-1.22892700	3.15021300	-0.83069300
O	-2.43618100	0.85306300	0.81422500
C	-2.83067100	-0.47453000	-1.11931600
H	0.77383200	0.85525900	-2.29933700
H	-0.19592600	-0.63031800	-2.28796600
H	-0.96126400	0.92544400	-2.69017200
H	-0.69415400	4.09046400	-1.00807300

H	-1.70517400	2.79842400	-1.75406600
H	-2.01932600	3.33235500	-0.08805500
H	-3.12154600	0.13207300	-1.99135000
H	-2.26985700	-1.34110700	-1.49247400
H	-3.73080500	-0.79550500	-0.58399300

Py-3-435-R2-Nu3

C	0.87389200	-0.10621500	-0.41166600
C	2.37509300	-0.06832900	-0.15567600
C	0.47905000	1.29803400	-0.84906100
C	3.00748400	1.12588000	0.14232800
C	1.25557800	2.38626400	-0.50199200
N	2.47424200	2.35012100	0.05767000
C	0.57874400	-1.07123300	-1.49518900
N	0.36937100	-1.82140800	-2.34648800
C	-0.69694200	1.49468500	-1.62705300
N	-1.63457800	1.65838300	-2.29145600
C	3.12974400	-1.27225200	-0.18076600
N	3.74208600	-2.25856600	-0.20769800
H	0.86253100	3.37999600	-0.74760100
H	4.06182600	1.07218700	0.43886200
C	0.04913800	-0.59293800	0.88841200
C	-1.45528500	-0.45328300	0.65494400
C	0.45818900	0.30120300	1.98758800
C	0.44886100	-2.02395600	1.29417500
C	-2.15518700	-1.45923900	-0.02351500
C	-2.14992800	0.69234200	1.06092400
N	0.78051400	0.97202900	2.86896500
H	1.49394200	-2.05835000	1.62488700
H	-0.19293800	-2.36985700	2.11488600
H	0.33771500	-2.71150400	0.44522700
C	-3.51271100	-1.31237900	-0.30299800
H	-1.64233300	-2.36379300	-0.35022600
C	-3.50800100	0.83684000	0.78449100
H	-1.62259100	1.48607900	1.59263200
C	-4.19344500	-0.16284200	0.09671800
H	-4.03957500	-2.10400900	-0.83762900
H	-4.02983100	1.73932200	1.10588900
H	-5.25584900	-0.04947000	-0.12386500

Py-3-435-R2-Nu2

C	-1.63259800	0.16792000	0.57598300
C	-2.49404400	-1.08535000	0.70472900
C	-2.29945000	1.05730700	-0.46135000
C	-3.44309200	-1.41081100	-0.24387400
C	-3.26352300	0.56008800	-1.31648200
N	-3.82189400	-0.65884800	-1.28282700
C	-1.61174400	0.83224200	1.90695100
N	-1.68574300	1.28887800	2.96351400
C	-1.97321000	2.44169400	-0.53628100
N	-1.76439900	3.58058400	-0.60311700

C	-2.19956600	-1.98463600	1.76495000
N	-1.92893700	-2.70711000	2.63251100
H	-3.64320700	1.24637800	-2.08257000
H	-3.95333300	-2.37393100	-0.12404800
C	-0.15500400	-0.25834500	0.23131400
Br	-0.08459100	-1.39872200	-1.31975300
S	1.00251800	1.14339400	-0.03736600
H	0.26539400	-0.82635500	1.07425200
O	0.84532100	1.64415200	-1.39603600
O	0.82564000	2.02865800	1.11022100
C	2.60311800	0.37792800	0.09505800
C	3.26960600	-0.00073500	-1.06875900
C	3.15441300	0.20035600	1.36347900
C	4.52555700	-0.59294000	-0.95120300
H	2.80627300	0.17439100	-2.04039200
C	4.40941000	-0.39612200	1.46432400
H	2.61132200	0.53547200	2.24865700
C	5.08905500	-0.79220800	0.31045100
H	5.06666600	-0.89720700	-1.84765700
H	4.86075900	-0.54569800	2.44555300
H	6.07208000	-1.25744300	0.39562900

Py-3-435-R2-Nu4

C	-0.31827200	-0.14708300	0.12363700
C	0.39529100	-0.85526300	-1.07356700
C	-1.82090000	-0.07584000	-0.15245100
C	1.90388000	-0.85487000	-1.06926300
H	0.03361900	-0.33491500	-1.97144600
H	0.01847700	-1.89303100	-1.11277200
C	0.15081900	1.29016100	0.33543500
C	-2.43689300	1.10725900	-0.50437300
C	2.60991800	-1.64580100	0.00021700
C	2.57716100	-0.17467700	-2.00349900
C	-0.62306100	2.36642000	-0.04745400
N	-1.88223100	2.32857500	-0.51093300
H	3.67554700	-1.76153600	-0.23757900
H	2.15846900	-2.64279900	0.11736600
H	2.53385900	-1.14287800	0.97623500
H	2.05535000	0.40523500	-2.76858700
H	3.66982000	-0.17529600	-2.02657000
C	-0.10448000	-0.93612800	1.36421200
N	0.03583800	-1.55440200	2.32912300
C	1.42848400	1.52266100	0.91494600
N	2.46032500	1.70566400	1.41609600
C	-2.55733000	-1.29127500	-0.16736400
N	-3.14259800	-2.29463000	-0.17305100
H	-0.17746700	3.36276800	0.06095200
H	-3.49471200	1.05261500	-0.78910400

Py-3-435-R3

C	-0.14249100	0.06852800	0.00000000

C	-0.86075200	-0.01446100	1.19748200
C	-0.86075200	-0.01446100	-1.19748200
C	1.35218000	0.31609700	0.00000000
C	-0.14541900	0.04038400	2.51089400
C	-2.25101300	-0.16769600	1.13747500
C	-0.14541900	0.04038400	-2.51089400
C	-2.25101300	-0.16769600	-1.13747500
O	2.02705600	-0.81971900	0.00000000
O	1.82841500	1.41534300	0.00000000
O	-0.97996000	0.01530400	3.54201400
O	1.05235500	0.09654600	2.61949400
O	-0.97996000	0.01530400	-3.54201400
O	1.05235500	0.09654600	-2.61949400
C	3.44422200	-0.68594800	0.00000000
C	-0.38264900	0.05724500	4.83585800
C	-0.38264900	0.05724500	-4.83585800
H	3.84930600	-1.70154200	0.00000000
H	3.76933700	-0.14062100	-0.89541000
H	3.76933700	-0.14062100	0.89541000
H	-1.20730600	0.02876900	5.55273600
H	0.19955400	0.97981200	4.95388500
H	0.27996800	-0.80620800	4.97551300
H	-1.20730600	0.02876900	-5.55273600
H	0.27996800	-0.80620800	-4.97551300
H	0.19955400	0.97981200	-4.95388500
N	-2.93448100	-0.24095700	0.00000000
H	-2.81930800	-0.23378700	-2.06812500
H	-2.81930800	-0.23378700	2.06812500

Py-3-435-R3-Nu1

C	0.12070300	-0.05847600	-0.03957300
C	-0.98898400	-0.20135700	-1.07088300
C	1.41800200	-0.01068000	-0.83442600
C	0.11276400	-1.27491400	0.90098900
C	-2.22265100	-0.92429100	-0.75168200
C	-0.83366000	0.38866100	-2.29945700
C	2.65358800	-0.48631900	-0.24272400
C	1.40483600	0.56642200	-2.09493700
O	0.39214000	-2.39736900	0.24490900
O	-0.12429500	-1.24561700	2.08262900
O	-3.24537200	-0.62873500	-1.58333600
O	-2.36065700	-1.74862000	0.12826900
O	3.73648700	-0.34613700	-1.04965900
O	2.77809600	-0.97351400	0.86822100
C	0.35402200	-3.57812500	1.02251700
C	-4.45407300	-1.31870600	-1.33474100
C	4.96618700	-0.78861900	-0.51104100
H	0.60534700	-4.40400400	0.34942800
H	1.08306400	-3.51977400	1.84257300
H	-0.65110400	-3.72019800	1.44287300
H	-5.17532300	-0.96009600	-2.07657000
H	-4.81877100	-1.10727200	-0.32004500

H	-4.31837000	-2.40478800	-1.43499100
H	5.72641200	-0.59967700	-1.27641800
H	4.93016000	-1.86130400	-0.27460500
H	5.21523500	-0.24210100	0.40945500
N	0.31948000	0.88948600	-2.80465300
H	2.37113800	0.75597400	-2.57585900
H	-1.69945500	0.41287900	-2.96835400
C	-0.05765400	1.26110900	0.88430500
C	-1.33887600	1.14069000	1.73281800
C	-0.25862600	2.49275300	-0.03758100
C	1.18595500	1.47671100	1.75699700
O	-2.40620900	0.88922300	1.22802100
C	-1.25050500	1.43546700	3.21500200
O	-1.35242000	2.82306000	-0.42588800
C	0.95397300	3.32689600	-0.40234000
H	1.33741400	0.62816500	2.43091900
H	2.08579300	1.57141900	1.14027900
H	1.09544500	2.39703200	2.35100400
H	-2.24469100	1.29694800	3.65440300
H	-0.52778200	0.76300800	3.69205800
H	-0.91722200	2.47047000	3.38105300
H	1.23656200	3.94554800	0.46336700
H	1.81876400	2.70824300	-0.66600800
H	0.67886000	3.98147100	-1.23677400

Py-3-435-R3-Nu3

C	-0.29411500	-0.39690000	0.09077700
C	-1.33113100	0.38215000	-0.70332500
C	0.84829900	-0.69347000	-0.87292000
C	-0.89265900	-1.71506000	0.61635800
C	-2.72651700	0.32995000	-0.30486500
C	-0.93370800	1.14409300	-1.78770600
C	1.71738300	-1.84064800	-0.63898000
C	1.08177700	0.15120100	-1.93654300
O	-1.39813500	-2.43484600	-0.38618600
O	-0.91110900	-2.08553700	1.76055700
O	-3.54334200	1.11390600	-1.04947200
O	-3.18037800	-0.34310500	0.60421000
O	2.87340700	-1.80230400	-1.34392200
O	1.47477700	-2.77619700	0.09779400
C	-1.96022000	-3.67861500	-0.01223500
C	-4.91308900	1.08597100	-0.69795400
C	3.73335500	-2.91324600	-1.17260900
H	-2.32968400	-4.14354600	-0.93177500
H	-1.19744700	-4.31465500	0.45745700
H	-2.78608400	-3.52767600	0.69687600
H	-5.42529200	1.75860700	-1.39387900
H	-5.06311300	1.42872000	0.33543800
H	-5.32172800	0.06994800	-0.78646800
H	4.60585600	-2.73037300	-1.80841700
H	3.23732000	-3.84673600	-1.47249900
H	4.04442000	-3.00848000	-0.12294800

N	0.26893800	1.12826500	-2.37870500
H	1.99415900	-0.01319600	-2.51905200
H	-1.68501600	1.79770500	-2.24380400
C	0.23603000	0.47340900	1.34017500
C	0.93451100	1.74428400	0.83004100
C	1.24298100	-0.30662200	2.08975400
C	-0.88449300	0.83587000	2.33564700
C	0.20687400	2.92426100	0.63410300
C	2.29986800	1.74987100	0.51674600
N	2.06011600	-0.84318200	2.70383400
H	-1.28614400	-0.06817100	2.80113600
H	-0.48546200	1.50976700	3.10663900
H	-1.70488400	1.34453800	1.81903000
C	0.82085500	4.06970800	0.12752300
H	-0.85840300	2.95243800	0.85904200
C	2.91624500	2.89254300	0.01030400
H	2.88835100	0.84237500	0.66046600
C	2.17762200	4.05804300	-0.19047700
H	0.23010900	4.97502200	-0.02181500
H	3.98054200	2.86869900	-0.22960700
H	2.65813600	4.95334100	-0.58845400

Py-3-435-R3-Nu2

C	1.15688700	0.24317000	-0.01211400
C	1.29391100	1.57268200	0.71188600
C	1.74931600	-0.81353900	0.90977700
C	1.91593800	0.28475800	-1.36006400
C	1.05365800	2.77368600	-0.06807500
C	1.45765900	1.62462600	2.07971700
C	2.28765100	-2.03885600	0.33355500
C	1.88433600	-0.57331700	2.26363500
O	3.19723700	0.59372400	-1.16976700
O	1.43656100	0.08977900	-2.44445000
O	1.12517500	3.92084300	0.63560900
O	0.80358600	2.78274900	-1.26036300
O	2.59457400	-2.98635100	1.25006400
O	2.49562000	-2.23521400	-0.84926300
C	4.00780100	0.56721700	-2.33197800
C	0.79294500	5.09809300	-0.07467500
C	3.18349800	-4.16752000	0.74205400
H	5.01896600	0.83819800	-2.01288800
H	4.00293900	-0.44041100	-2.77005300
H	3.63860800	1.28399200	-3.07779400
H	0.89812500	5.92760900	0.63216300
H	-0.24034500	5.04783700	-0.44595100
H	1.46392300	5.24455000	-0.93202500
H	3.37213800	-4.81523800	1.60458200
H	4.12706700	-3.94748000	0.22334600
H	2.50934300	-4.66978500	0.03446600
N	1.70037000	0.59233800	2.90012800
H	2.23736600	-1.40594400	2.88088800
H	1.41219100	2.61497300	2.54501200

C	-0.34060300	0.00513700	-0.42838400
Br	-0.74045300	-1.84904000	-0.83961600
S	-1.55371100	0.60024400	0.81293900
H	-0.55905200	0.58650100	-1.33178300
O	-1.30397200	-0.05796300	2.09148300
O	-1.59804800	2.06102600	0.72497800
C	-3.13846000	0.03090700	0.21273100
C	-3.74439000	-1.05619900	0.83657600
C	-3.75269600	0.72764000	-0.82577100
C	-5.00018100	-1.46617200	0.39305100
H	-3.22880200	-1.56272800	1.65321300
C	-5.00418000	0.30097100	-1.26703600
H	-3.26163800	1.59482800	-1.27022100
C	-5.62387100	-0.79242800	-0.65881200
H	-5.49295500	-2.31465100	0.86920900
H	-5.50081500	0.82936600	-2.08156700
H	-6.60557100	-1.11935100	-1.00472800

Py-3-435-R3-Nu4

C	0.18762300	0.11037600	0.25378000
C	-0.05462600	0.06457000	1.80017800
C	-0.89885500	0.91039800	-0.44917500
C	-1.45460200	-0.24946200	2.27983400
H	0.66956000	-0.64786900	2.22476800
H	0.20525400	1.07170200	2.15625200
C	1.49736200	0.84573200	-0.02197200
C	0.26685500	-1.31952800	-0.28949100
C	-2.15624000	0.24927500	-0.74996800
C	-0.73877400	2.26324600	-0.64543800
C	-1.87189000	-1.68724000	2.43295200
C	-2.30260700	0.74966700	2.55881700
C	2.74963700	0.12948700	0.12705600
C	1.48206600	2.20684200	-0.25379600
O	0.48664400	-1.33044600	-1.61057000
O	0.17058400	-2.32992900	0.35592700
O	-3.14085200	1.08269600	-1.16382400
O	-2.35800700	-0.94979800	-0.66543000
H	-1.95766000	-2.15639400	1.44437700
H	-1.11392000	-2.25655800	2.99190300
H	-2.83561500	-1.76071900	2.95651000
H	-2.00302100	1.79469800	2.44174500
H	-3.32083100	0.55163400	2.90545100
O	3.85480700	0.89667100	-0.05261600
O	2.86074300	-1.05732300	0.38184400
C	0.55732300	-2.61298600	-2.20304000
C	-4.38187900	0.47201300	-1.45458100
C	5.09459300	0.23519800	0.09729300
H	0.75607400	-2.45593900	-3.26826300
H	1.36323100	-3.20627400	-1.74873900
H	-0.39352100	-3.14799600	-2.06969000
H	-5.05474700	1.27358800	-1.77740300
H	-4.79213900	-0.02681400	-0.56488500

H	-4.27804300	-0.27615500	-2.25278500
H	5.87123200	0.98698700	-0.07919600
H	5.19515200	-0.58538700	-0.62671200
H	5.20125700	-0.18374500	1.10796000
N	0.39992300	2.96741200	-0.48772800
H	2.44719200	2.72416900	-0.28133100
H	-1.60617200	2.82868600	-1.00320500

Py-3-435-R4

C	0.00000000	0.00000000	0.73073300
C	0.00000000	1.18928800	0.00938400
C	0.00000000	-1.18928800	0.00938400
C	0.00000000	1.14611700	-1.38132800
C	0.00000000	-1.14611700	-1.38132800
N	0.00000000	0.00000000	-2.05442700
H	0.00000000	-2.08736500	-1.93746000
H	0.00000000	2.08736500	-1.93746000
F	0.00000000	-2.34152500	0.66097500
F	0.00000000	0.00000000	2.04858800
F	0.00000000	2.34152500	0.66097500

Py-3-435-R4-Nu1

C	0.59188400	-0.10421800	-0.40326100
C	1.41962200	-1.23463000	0.04941800
C	1.46050800	1.06957300	-0.22467500
C	2.74709900	-1.16914400	0.37854800
C	2.79049100	1.05247600	0.09090900
N	3.49656400	-0.05108100	0.40131200
H	3.30946500	2.01920100	0.09598000
H	3.22344000	-2.11452400	0.66815800
C	-0.81536600	-0.04090800	0.31160900
C	-1.79181500	-1.13801000	-0.17291500
C	-1.52607000	1.28000800	-0.04143100
C	-0.61065500	-0.19643200	1.82558300
O	-1.56999900	-1.91566100	-1.06119100
C	-3.10053200	-1.21507900	0.59749700
O	-2.18834500	1.35797600	-1.05032200
C	-1.46424600	2.42577500	0.94143700
H	-0.29017300	-1.22363400	2.04852100
H	0.18081400	0.48121800	2.17144900
H	-1.52724100	0.01675800	2.39176900
H	-2.93434400	-1.79678900	1.51691800
H	-3.48161700	-0.22716600	0.88866800
H	-3.84109600	-1.73985300	-0.01677100
H	-2.21115700	2.24342800	1.73034400
H	-0.48151500	2.51481800	1.41650200
H	-1.72377400	3.35362700	0.41910200
F	0.87756900	2.26828400	-0.53948800
F	0.24409600	-0.17664000	-1.80462800
F	0.78527700	-2.43436700	0.19255600

Py-3-435-R4-Nu3

C	0.98726900	0.04592000	-0.48734300
C	2.38171800	-0.36794900	-0.31184400
C	0.93161800	1.40699200	0.05304200
C	3.40878000	0.43343400	0.10958600
C	2.01230800	2.14731200	0.45704700
N	3.28182600	1.71107600	0.50538700
H	1.80497400	3.17550300	0.77832100
H	4.40620900	-0.02233600	0.13580600
C	-0.10788000	-0.97355300	0.05439500
C	-1.52793500	-0.39067400	0.04615700
C	0.29132200	-1.29905300	1.43639500
C	-0.12907200	-2.30067900	-0.74169600
C	-2.13583800	-0.08062000	-1.17867400
C	-2.25710800	-0.19267000	1.22163100
N	0.60901000	-1.59300200	2.50632800
H	0.82338800	-2.83124000	-0.65496600
H	-0.93901400	-2.93800800	-0.36110100
H	-0.31439100	-2.07644500	-1.79745500
C	-3.43319500	0.42201300	-1.21942400
H	-1.57035100	-0.20612700	-2.10066800
C	-3.55911300	0.31037400	1.18005900
H	-1.80818600	-0.42581400	2.18810800
C	-4.15351800	0.62096000	-0.03987100
H	-3.88506000	0.66424600	-2.18297400
H	-4.10704900	0.45896700	2.11210200
H	-5.17039900	1.01518400	-0.07399800
F	-0.29023100	2.00870300	0.06717500
F	0.64417800	0.05711700	-1.92861800
F	2.67582000	-1.65818600	-0.64988400

Py-3-435-R4-Nu2

C	-1.69376400	0.38090000	0.62382200
C	-2.53203000	-0.67685600	1.17791700
C	-2.46621900	0.98672600	-0.45043000
C	-3.84334400	-0.89338700	0.85357200
C	-3.78196400	0.71553400	-0.71777300
N	-4.52391800	-0.21001400	-0.08419100
H	-4.25193000	1.29156600	-1.52411900
H	-4.36471900	-1.69281700	1.39385800
C	-0.24939300	-0.10665200	0.29210300
Br	-0.25165900	-1.64971400	-0.86659300
S	0.91140200	1.13834500	-0.41888500
H	0.21525500	-0.41225500	1.23666000
O	0.81673800	1.13678000	-1.87369900
O	0.77782700	2.38442500	0.32437800
C	2.49368800	0.42968800	0.01474400
C	3.16507800	-0.36029900	-0.91571200
C	3.02772900	0.71095500	1.27153300
C	4.40255600	-0.89837800	-0.56521200
H	2.71780700	-0.53928500	-1.89418300
C	4.26459400	0.16555200	1.60930000

H	2.48214600	1.35587000	1.96231600
C	4.94711100	-0.63796100	0.69342100
H	4.94474400	-1.51994300	-1.27869100
H	4.70024600	0.37416000	2.58705700
H	5.91602200	-1.06125200	0.96215700
F	-1.35121800	1.34707600	1.67344000
F	-1.91556700	-1.46988600	2.09877200
F	-1.80291500	1.91569500	-1.18909700

Py-3-435-R4-Nu4

C	-0.23676100	-0.28395700	0.18950700
C	0.96241100	-0.92773700	-0.53107800
C	-1.51563100	-0.92243500	-0.12611000
C	2.31272800	-0.27587500	-0.36175800
H	0.72290200	-0.99007900	-1.60231700
H	1.01565300	-1.95950300	-0.14589800
C	-0.47314400	1.14738400	0.00567200
C	-2.71343700	-0.27646300	-0.26378200
C	2.80485500	-0.03426300	1.03841900
C	3.05818300	0.03152600	-1.42970200
C	-1.70646600	1.72920000	-0.12833400
N	-2.86928400	1.06238200	-0.25442100
H	3.84042700	0.33244500	1.03439500
H	2.74791300	-0.95691200	1.63512700
H	2.15828000	0.69898300	1.53779200
H	2.69394300	-0.13949500	-2.44589300
H	4.05379100	0.46870000	-1.31829700
H	-1.73527400	2.82584900	-0.16269500
H	-3.60303700	-0.89938900	-0.42122000
F	-1.47719000	-2.28452500	-0.22551900
F	0.10050200	-0.55552800	1.62434400
F	0.62490500	1.95765800	0.08039200

Py-3-435-R5

C	0.00000000	0.00000000	-0.11080200
C	-0.02542400	1.18223000	0.61734700
C	0.02542400	-1.18223000	0.61734700
C	-0.02782800	1.14019100	2.01196700
C	0.02782800	-1.14019100	2.01196700
N	0.00000000	0.00000000	2.68947000
N	0.00000000	0.00000000	-1.59407600
O	-1.03636500	0.29454600	-2.11724000
O	1.03636500	-0.29454600	-2.11724000
N	0.00000000	2.49636000	-0.04514200
O	-0.39609500	3.43372900	0.59550800
O	0.43205000	2.53111100	-1.16953500
N	0.00000000	-2.49636000	-0.04514200
O	-0.43205000	-2.53111100	-1.16953500
O	0.39609500	-3.43372900	0.59550800
H	0.04296100	-2.07984700	2.56877600
H	-0.04296100	2.07984700	2.56877600

Py-3-435-R5-Nu1

C	0.27961400	-0.06429400	-0.30530500
C	-0.52059400	-1.33628200	-0.46681700
C	1.67770800	-0.54384500	-0.00913800
C	-0.13449800	-2.52605100	0.10056000
C	1.93762100	-1.78607600	0.54198200
N	1.05480600	-2.77346400	0.67299700
N	0.30528000	0.69100400	-1.68199400
O	0.81800400	0.11113500	-2.59584500
O	-0.19079400	1.78633800	-1.73555300
N	-1.76757600	-1.34441600	-1.18985800
O	-2.61405400	-2.16371000	-0.88245300
O	-1.90408300	-0.56160000	-2.10730900
N	2.78676800	0.32452300	-0.22432300
O	2.59467800	1.37404000	-0.82240300
O	3.89369600	-0.00495600	0.16996500
H	2.96238800	-1.97590500	0.86906500
H	-0.84520200	-3.35466700	0.04566700
C	-0.29736500	0.91491200	0.81727600
C	-0.50322500	-0.01541300	2.06848900
C	-1.65372000	1.52536900	0.40708200
C	0.71063900	1.98867800	1.23960500
O	0.44636400	-0.21956500	2.78284900
C	-1.84923200	-0.61822200	2.38931500
O	-2.57823400	0.80785300	0.10576700
C	-1.83893500	3.01996200	0.48987400
H	1.62772100	1.51993400	1.61129200
H	0.95307600	2.65908800	0.41024800
H	0.29452100	2.57201700	2.07219700
H	-1.70933500	-1.32754300	3.21248900
H	-2.53695100	0.18109300	2.70473100
H	-2.30491800	-1.10197700	1.51796500
H	-1.79213300	3.34145700	1.54108700
H	-1.05226900	3.54793000	-0.06054100
H	-2.82455200	3.26634500	0.08036700

Py-3-435-R5-Nu3

C	-0.77440800	0.08590400	-0.12016700
C	-1.62769100	1.29842300	0.11021700
C	-1.74704900	-1.05612600	-0.09647900
C	-2.94979400	1.26463500	0.50561700
C	-3.05514200	-0.96753600	0.32196200
N	-3.68368400	0.16248800	0.66611800
N	-0.26026700	0.19575900	-1.63305200
O	-1.16082600	0.29505800	-2.43055900
O	0.90020400	0.18547200	-1.91162200
N	-1.02973700	2.57192300	-0.09939900
O	-1.65395200	3.59091600	0.13147800
O	0.12285400	2.57908500	-0.51582700
N	-1.26366900	-2.32684400	-0.53621200

O	-0.12951900	-2.35229900	-0.99628200
O	-1.96996200	-3.31140800	-0.45614700
H	-3.62570200	-1.89861000	0.36028300
H	-3.43105200	2.22707300	0.69545100
C	0.43559700	-0.09134800	0.96592400
C	1.90287100	-0.02868200	0.49771300
C	0.25701400	-1.40783000	1.62151400
C	0.21500500	0.89413700	2.14271800
C	2.59754700	1.18372300	0.48407700
C	2.58247300	-1.19758300	0.13705400
N	0.12445100	-2.38501200	2.22056700
H	-0.78154700	0.75438700	2.57883500
H	0.97078300	0.69390900	2.91311400
H	0.31965000	1.93476200	1.82176700
C	3.93664400	1.22837400	0.09770800
H	2.08918300	2.11134300	0.74065100
C	3.92027800	-1.15269200	-0.24785200
H	2.05101700	-2.15030900	0.13158300
C	4.60459600	0.06226300	-0.27235500
H	4.45880600	2.18654000	0.08528600
H	4.42893400	-2.07528300	-0.53219600
H	5.65223700	0.09944700	-0.57516700

Py-3-435-R5-Nu2

C	1.49632900	0.24885900	0.12173800
C	1.97460200	1.39453500	-0.70910000
C	2.35965400	-0.90771100	-0.27705300
C	2.94886800	1.31485100	-1.68328600
C	3.32195100	-0.85556400	-1.26035300
N	3.63663600	0.21740500	-1.99501000
N	1.84508400	0.58868600	1.63964000
O	2.90004500	1.14803300	1.79918800
O	1.11021700	0.22464000	2.51004500
N	1.40005900	2.66845800	-0.42727500
O	1.70272500	3.63850000	-1.09502200
O	0.61177600	2.72006400	0.50977200
N	2.15208600	-2.15961200	0.39089300
O	1.29832900	-2.18483300	1.25677300
O	2.81518000	-3.12903100	0.07241800
H	3.86706700	-1.78115400	-1.45844200
H	3.17345600	2.23198600	-2.23341400
C	-0.06949100	0.06860300	-0.06443400
Br	-0.46562900	-1.37184000	-1.30786200
S	-1.35097500	-0.02195400	1.30120600
H	-0.42502000	0.96471700	-0.57989300
O	-1.37340200	-1.33252600	1.92993300
O	-1.29301600	1.20107300	2.08962600
C	-2.83914200	0.13542200	0.31546800
C	-3.65373500	-0.97636600	0.12372400
C	-3.17563400	1.39617400	-0.17513500
C	-4.83448500	-0.82113200	-0.60078200
H	-3.35537400	-1.94011700	0.53802200

C	-4.35259300	1.53518000	-0.90832000
H	-2.52861100	2.25247200	0.02689000
C	-5.17799800	0.42877600	-1.11922200
H	-5.48815900	-1.67915900	-0.76176700
H	-4.63126100	2.51173900	-1.30573600
H	-6.10131600	0.54349600	-1.68885500

Py-3-435-R5-Nu4

C	-0.25610800	-0.07658900	-0.02355200
C	0.29260700	-1.00407800	-1.14710700
C	-1.67379800	0.35625400	-0.28935500
C	1.79347100	-1.10183500	-1.32551600
H	-0.12470900	-0.57160000	-2.06729500
H	-0.14957900	-1.99730800	-1.01199600
C	0.50511300	1.20503500	0.16574400
C	-2.04924400	1.65531700	-0.56032700
C	2.51233700	-2.23916200	-0.65463200
C	2.43113100	-0.21421300	-2.09780400
C	0.00622200	2.45267700	-0.12996600
N	-1.24666400	2.72219600	-0.52007600
H	3.58134500	-2.23380700	-0.90598900
H	2.07922100	-3.20096400	-0.97249000
H	2.39489400	-2.17558100	0.43445300
H	1.89079000	0.59344700	-2.59926000
H	3.51283500	-0.26496700	-2.24468700
N	-0.25507700	-0.86825000	1.34488100
O	-0.70277900	-0.27469900	2.29322400
O	0.16884500	-1.99044100	1.37001200
N	-2.67992700	-0.65283400	-0.33835900
O	-3.81710100	-0.36397900	-0.67053300
O	-2.35641000	-1.78882100	-0.01835300
N	1.83569200	1.13316300	0.68283000
O	2.15886400	0.10357500	1.25372500
O	2.58271400	2.08842400	0.57130800
H	0.69042400	3.29705100	-0.01479000
H	-3.09684500	1.82089500	-0.82285400

Py-4-2356-R1

C	0.00000000	1.14579200	0.66590000
C	0.01474100	1.20537000	-0.73286400
C	0.00000000	0.00000000	-1.42695900
C	0.00000000	-1.14579200	0.66590000
C	-0.01474100	-1.20537000	-0.73286400
C	0.02434000	2.49869800	-1.52865300
C	-0.02434000	-2.49869800	-1.52865300
C	-0.03293500	-2.38707200	1.55740100
C	0.03293500	2.38707200	1.55740100
F	1.27004600	2.86754200	1.64282300
F	-0.74976700	3.34604900	1.06774100
F	-0.38277300	2.10375100	2.77889100
F	0.74976700	-3.34604900	1.06774100

F	0.38277300	-2.10375100	2.77889100
F	-1.27004600	-2.86754200	1.64282300
F	1.17737900	-3.06351500	-1.55726800
F	-0.38567600	-2.26554300	-2.78817900
F	-0.88424500	-3.37439400	-1.01811000
F	0.88424500	3.37439400	-1.01811000
F	0.38567600	2.26554300	-2.78817900
F	-1.17737900	3.06351500	-1.55726800
N	0.00000000	0.00000000	1.32442500
H	0.00000000	0.00000000	-2.51676900

Py-4-2356-R1-Nu1

C	-0.74987900	-0.21292400	0.31849400
C	-0.44025400	1.27988200	0.20642000
C	0.82986600	1.66186200	-0.11534800
C	1.61568100	-0.53220900	0.30829700
C	1.91163900	0.75497200	-0.19301400
C	-1.42082400	2.36233500	0.52973700
C	3.25478600	1.25498800	-0.56112800
C	2.71277000	-1.57283900	0.50420800
C	-1.65260300	-0.50600400	1.53634500
F	-1.10221100	-0.03835000	2.65398300
F	-2.86652900	0.04622600	1.43162200
F	-1.84263900	-1.81550000	1.72112900
F	3.84200100	-1.04435900	0.98224400
F	2.35184900	-2.54189100	1.33833500
F	3.01017200	-2.15638100	-0.66575000
F	4.04385900	1.60312700	0.47909700
F	3.16705900	2.36473500	-1.31682800
F	3.97275300	0.36475100	-1.26978000
F	-1.65444800	2.47706100	1.85434000
F	-0.98558400	3.56700800	0.13383100
F	-2.63703300	2.20935400	-0.02141600
N	0.44020700	-0.97302000	0.62125700
H	1.03158900	2.72224000	-0.27315700
C	-1.41794200	-0.83486300	-1.05429300
C	-2.43029000	0.15958900	-1.68503200
C	-2.03044900	-2.23113300	-0.81067300
C	-0.27928400	-0.98013500	-2.07988800
O	-1.99898500	0.97522800	-2.46823500
C	-3.91527700	0.12942900	-1.40759400
O	-3.22607400	-2.41337900	-0.78236700
C	-1.08356500	-3.39819600	-0.65245300
H	0.18947200	-0.00588600	-2.25030400
H	0.48042300	-1.69011800	-1.74176100
H	-0.68965300	-1.33109700	-3.03788400
H	-4.31512800	1.11291900	-1.68055000
H	-4.36548000	-0.64207200	-2.04719600
H	-4.15665200	-0.12980500	-0.37520600
H	-0.70469000	-3.69228700	-1.64291400
H	-0.22787700	-3.12076800	-0.02419500
H	-1.64244900	-4.23732900	-0.22255500

Py-4-2356-R1-Nu3

C	0.56395300	0.60288600	-0.22821800
C	-0.45005800	1.71970700	0.04570000
C	-1.73886300	1.34760600	0.31215800
C	-1.30402600	-0.83597900	-0.51053100
C	-2.20544500	0.02463400	0.15701000
C	-0.15884600	3.18604400	-0.03835400
C	-3.59238800	-0.31003500	0.54827600
C	-1.73140400	-2.22004600	-0.98560300
C	1.56605100	1.01914500	-1.32955000
F	0.90968700	1.41993500	-2.41775100
F	2.35809900	2.03230700	-0.94422400
F	2.36658100	0.03181800	-1.71798200
F	-3.00113700	-2.25416600	-1.39881300
F	-0.98344700	-2.66161200	-1.99055700
F	-1.61363700	-3.10963800	0.01003800
F	-4.50536100	-0.24385500	-0.44651100
F	-4.04733500	0.53633200	1.49036300
F	-3.70737600	-1.54945100	1.05665600
F	0.09360700	3.61185200	-1.29167100
F	-1.19807200	3.91973900	0.39048500
F	0.89857600	3.60389800	0.68366600
N	-0.06826700	-0.57016500	-0.78797100
H	-2.45582800	2.11437400	0.60830500
C	1.35910000	0.14404200	1.10935300
C	2.45758100	-0.88702200	0.81526100
C	0.36969400	-0.52359800	1.98441700
C	1.88798400	1.32234300	1.94629500
C	3.80471900	-0.51120700	0.79759400
C	2.12780400	-2.21940900	0.53506200
N	-0.33531600	-1.03608200	2.74063800
H	1.05331800	1.93545200	2.30231200
H	2.43227800	0.92809400	2.81473300
H	2.55490500	1.96201000	1.36050500
C	4.79838000	-1.43980600	0.48796400
H	4.09348900	0.51795800	1.01028300
C	3.12025900	-3.14689200	0.22663300
H	1.08251000	-2.52937200	0.55266000
C	4.46036600	-2.76024300	0.19770300
H	5.84325900	-1.12559200	0.47563100
H	2.84258100	-4.17951100	0.00953600
H	5.23775400	-3.48712600	-0.04295100

Py-4-2356-R1-Nu2

C	-0.50703900	1.05502500	-0.48731800
C	-1.00690200	1.42085200	0.90661500
C	-1.61706000	0.39271900	1.56668400
C	-2.09332100	-0.64058600	-0.51467200
C	-1.98235100	-0.80108500	0.89156500
C	-1.00724100	2.78242100	1.53438300

C	-2.59410000	-1.89555200	1.68147900
C	-2.97006600	-1.55844400	-1.35505700
C	-0.18820700	2.26223000	-1.38157400
F	-1.29295000	2.91624300	-1.72260500
F	0.61923600	3.12715000	-0.76034600
F	0.41554100	1.89906400	-2.51039000
F	-4.10137600	-1.89483200	-0.72595700
F	-3.31696400	-1.00022000	-2.50842900
F	-2.32599700	-2.69439500	-1.64365400
F	-3.89552400	-1.71392800	1.98992300
F	-1.97505700	-2.03524800	2.86870700
F	-2.52470500	-3.08525000	1.06574400
F	-1.62495600	3.69385100	0.75834600
F	-1.68419500	2.76315000	2.69629300
F	0.18399100	3.30897800	1.82321700
N	-1.53386000	0.31591000	-1.18816200
H	-1.90731400	0.51559300	2.61182900
C	0.74665800	0.09402900	-0.35250000
Br	1.09945700	-0.99517900	-1.92246700
S	2.33238100	0.82598200	0.18499100
H	0.49071200	-0.62267000	0.43797000
O	2.96410200	1.50474300	-0.94051900
O	2.07406800	1.53997000	1.42886900
C	3.31669400	-0.60518300	0.59206400
C	4.27871200	-1.05182300	-0.31050500
C	3.12025200	-1.21194500	1.83165800
C	5.05778100	-2.15342000	0.03815700
H	4.40421000	-0.53697400	-1.26365800
C	3.90107700	-2.31729800	2.16381700
H	2.37603800	-0.81510900	2.52450200
C	4.86509500	-2.78482000	1.26848500
H	5.81765900	-2.52020800	-0.65269000
H	3.76206600	-2.80925700	3.12700000
H	5.47592100	-3.64867800	1.53453700

Py-4-2356-R1-Nu4

C	1.13261500	-0.70662100	0.10245400
C	1.53409800	-0.94640500	1.59444900
C	1.15233600	0.76857400	-0.28792300
C	0.67323300	-0.22329600	2.60200300
H	1.47804100	-2.03255100	1.76626100
H	2.58041100	-0.63257200	1.70891600
C	-0.02670600	1.44617200	-0.34247400
C	-0.52731000	-0.95956600	3.13313700
C	0.99227700	1.00833100	3.01505700
C	-1.22083500	-0.61230900	-0.25972700
C	-1.28444600	0.80614600	-0.23302200
H	-0.20854600	-1.87307600	3.66099100
H	-1.10332000	-0.33715500	3.83093300
H	-1.18903100	-1.28120200	2.31746700
H	1.87944300	1.51746200	2.63116800
H	0.36872800	1.54294900	3.73616800

C	2.42096400	1.52328500	-0.45530700
C	-2.51298200	1.61826000	-0.32910900
C	-2.49193200	-1.44588500	-0.39902100
C	2.12395200	-1.53299400	-0.73862400
F	1.90028900	-1.39285400	-2.04516400
F	3.39922500	-1.18330000	-0.51419500
F	2.04338100	-2.83787000	-0.46345400
F	-3.35671700	-0.91153600	-1.26740200
F	-2.24632800	-2.68436900	-0.81043500
F	-3.12844800	-1.54683700	0.77783700
F	-3.02169600	1.75891000	-1.57719700
F	-2.29922100	2.88008400	0.09367400
F	-3.53018100	1.13681600	0.41046000
F	3.10184200	1.20973800	-1.57705100
F	2.22579200	2.84953000	-0.51311400
F	3.29811500	1.32347100	0.55533900
N	-0.15234600	-1.32980400	-0.17022600
H	-0.00534600	2.52657000	-0.49535500

Py-4-2356-R2

C	0.00000000	1.19839900	-0.72721600
C	0.00000000	0.00000000	-1.44030400
C	0.00000000	-1.13882900	0.68141500
C	0.00000000	-1.19839900	-0.72721600
C	0.00000000	2.46488500	-1.40904000
N	0.00000000	3.48127200	-1.95388500
C	0.00000000	-2.46488500	-1.40904000
N	0.00000000	-3.48127200	-1.95388500
C	0.00000000	-2.36210100	1.46016400
N	0.00000000	-3.34132700	2.06798500
C	0.00000000	2.36210100	1.46016400
N	0.00000000	3.34132700	2.06798500
N	0.00000000	0.00000000	1.35860300
H	0.00000000	0.00000000	-2.52968300
C	0.00000000	1.13882900	0.68141500

Py-4-2356-R2-Nu1

C	0.13214600	-0.08590900	0.18976600
C	-0.34100200	-1.47163500	-0.25660600
C	-1.68510100	-1.72528800	-0.17558200
C	-2.07067800	0.63662100	-0.16793500
C	-2.60332500	-0.67059300	0.05617700
C	0.55081300	-2.48860300	-0.69783800
N	1.27093100	-3.30984200	-1.08837100
C	-4.00014400	-0.89851500	0.17011900
N	-5.14002400	-1.08676100	0.27551100
C	-3.00472100	1.71987400	-0.46453800
N	-3.74216800	2.57736100	-0.69283100
C	0.10647300	-0.04744500	1.69123000
N	-0.07412900	0.00360600	2.82974300
N	-0.81391500	0.94128800	-0.24798500

H	-2.06226000	-2.73247100	-0.36363500
C	1.54131700	0.31821300	-0.35605800
C	2.64310300	-0.56728000	0.25169600
C	1.84694500	1.75113400	0.16348100
C	1.54440300	0.26306400	-1.88769500
O	2.41710100	-1.31870600	1.16570700
C	4.01673300	-0.44732000	-0.35869100
O	2.29047000	1.87688900	1.27962200
C	1.59325100	2.92971800	-0.73548700
H	1.45991400	-0.76939000	-2.25005900
H	0.69500000	0.83676000	-2.27907700
H	2.47061600	0.68904300	-2.29489900
H	4.75395300	-0.87909100	0.32667000
H	4.02911300	-1.01628300	-1.30121600
H	4.27222800	0.59559300	-0.59168100
H	2.26293300	2.89569400	-1.60659200
H	0.55808500	2.88902300	-1.09763400
H	1.77369500	3.84988300	-0.16920600

Py-4-2356-R2-Nu3

C	0.26049800	0.56067000	0.27879100
C	1.45572400	1.48890200	0.06381000
C	2.69269000	0.90945200	0.21238200
C	1.68041400	-1.19755100	-0.26924100
C	2.81705700	-0.49966300	0.25277100
C	1.36227900	2.87568200	-0.23445100
N	1.31738400	4.00937000	-0.47603100
C	4.07102600	-1.14257900	0.43690800
N	5.09093900	-1.66901800	0.60243300
C	1.88447700	-2.53044100	-0.82852900
N	2.04832400	-3.58840200	-1.25857800
C	0.15677900	0.28052700	1.75038900
N	0.10052700	0.07102000	2.88375200
N	0.49470500	-0.69680100	-0.41025300
H	3.59080100	1.53011000	0.21384900
C	-1.14831300	1.11753600	-0.17937200
C	-2.20629200	0.01008900	-0.04572800
C	-1.00609000	1.47775900	-1.60450200
C	-1.56895300	2.38029600	0.60018900
C	-2.92916400	-0.13090200	1.14395000
C	-2.45491400	-0.88418800	-1.09350400
N	-0.90767000	1.78624500	-2.71148700
H	-0.96222400	3.24827300	0.32654200
H	-2.62012300	2.60585700	0.38099600
H	-1.46638000	2.21252600	1.67996500
C	-3.87657800	-1.14597000	1.28347000
H	-2.75676100	0.54537900	1.98075500
C	-3.40657000	-1.89190800	-0.95740400
H	-1.89302500	-0.79433400	-2.02353300
C	-4.12095900	-2.02768200	0.23319800
H	-4.42735600	-1.24048200	2.22034100
H	-3.58729600	-2.57673300	-1.78711600

H	-4.86576000	-2.81765700	0.34059200

Py-4-2356-R2-Nu2

C	1.00634600	0.32683900	0.68526600
C	1.82794300	1.57554900	0.35583400
C	3.17564500	1.45213100	0.16438800
C	2.89263700	-0.93079400	0.04341500
C	3.78999100	0.17934400	0.09454400
C	1.17937600	2.84117500	0.38396800
N	0.61070900	3.85108000	0.42522400
C	5.18569600	0.02456400	-0.10672000
N	6.32901500	-0.09934300	-0.26130100
C	3.44330300	-2.23199100	-0.33502700
N	3.88587600	-3.25589200	-0.62810200
C	0.87887600	0.29647700	2.18161100
N	0.88787300	0.36524800	3.33332600
N	1.61246200	-0.90772800	0.23032200
H	3.77998400	2.34968700	0.02199100
C	-0.42603100	0.41288000	0.09082800
Br	-0.39897900	0.82167500	-1.78951400
S	-1.35202700	-1.16895500	0.33208100
H	-1.01570600	1.19259400	0.59091500
O	-1.10533600	-2.07651900	-0.77656900
O	-1.09226100	-1.57808300	1.70847400
C	-3.04286700	-0.62032000	0.22771700
C	-3.71554500	-0.73426800	-0.98716500
C	-3.65127300	-0.10859800	1.37337100
C	-5.03998800	-0.30479900	-1.05518500
H	-3.20562100	-1.15948500	-1.85243200
C	-4.97484300	0.31733200	1.28886700
H	-3.09656300	-0.05832600	2.31186100
C	-5.66365200	0.22033600	0.07771200
H	-5.58730900	-0.38488900	-1.99489100
H	-5.47194100	0.71945200	2.17212200
H	-6.70061500	0.55375100	0.01807300

Py-4-2356-R2-Nu4

C	0.65074100	-0.06329600	0.78806200
C	2.03279100	0.63315600	0.83181300
C	0.54496900	-1.12355200	-0.30566500
C	2.44628800	1.31358600	-0.45344400
H	1.97646100	1.37681200	1.64296900
H	2.78940100	-0.11446400	1.11066400
C	-0.69570800	-1.45680200	-0.77785800
C	1.62435600	2.47797800	-0.93939600
C	3.54006600	0.90730000	-1.10689800
C	-1.54312200	0.56228700	0.21273900
C	-1.83209500	-0.69016900	-0.42010900
H	1.41819600	3.18267300	-0.12013200
H	2.14374700	3.00814100	-1.74895100
H	0.64519500	2.14190100	-1.30930900

H	4.12495900	0.05220200	-0.76090100
H	3.87266100	1.41346700	-2.01671100
C	1.70137100	-1.85565800	-0.69497000
N	2.64348700	-2.46867400	-0.98262300
C	-3.13437500	-1.01037900	-0.88094000
N	-4.20233500	-1.28054500	-1.24727700
C	-2.60091400	1.57281500	0.23835600
N	-3.43523400	2.36925400	0.26515200
C	0.47636000	-0.74077800	2.11719700
N	0.33709800	-1.26815800	3.13464800
N	-0.40403700	0.94359300	0.69094400
H	-0.80783500	-2.28793600	-1.47684800

Py-4-2356-R3

C	-1.17362200	-0.76051100	-0.21552100
C	-1.29417100	0.62926600	-0.07603300
C	-2.38251800	-1.66224600	-0.34692400
C	1.10761400	-0.66828700	-0.13023500
C	-2.64438200	1.27499400	-0.10981200
C	-0.12563100	1.37838300	0.03064900
O	-3.04790600	-1.73262400	0.79537200
O	-2.65360700	-2.26295900	-1.34765300
C	1.10417300	0.72806800	-0.01337600
C	2.38674700	-1.47467300	-0.09823400
O	-2.60376000	2.53728900	0.29005500
O	-3.64335700	0.71259000	-0.47751300
C	-4.26763000	-2.46864800	0.75705800
C	2.34812900	1.56245200	0.02869600
O	3.09036100	-1.19806500	0.99252000
O	2.69228900	-2.28093300	-0.92972000
C	-3.84011500	3.24837200	0.25804300
H	-4.67274900	-2.44486300	1.77218100
H	-4.96188900	-1.98967700	0.05387500
H	-4.08285600	-3.50235800	0.43944700
O	3.34055700	1.01058000	-0.65869800
O	2.41307300	2.62871600	0.57885800
C	4.35630200	-1.84458200	1.09561700
H	-3.61916800	4.25860700	0.61169500
H	-4.57198800	2.76095400	0.91419900
H	-4.23453200	3.27421800	-0.76556900
C	4.58172500	1.71310400	-0.65057500
H	4.22979900	-2.93438500	1.09097000
H	4.99380100	-1.55297600	0.24998400
H	4.79475000	-1.51229400	2.04027400
H	4.96582700	1.78535900	0.37531100
H	4.44698600	2.72270400	-1.05787400
H	5.26390300	1.13283200	-1.27732100
N	-0.00376300	-1.38755200	-0.24264000
H	-0.16646400	2.46383600	0.12942000

Py-4-2356-R3-Nu1

C	-0.84641900	-0.29960700	0.19730900
C	-0.74160500	1.21297000	0.02403900
C	-1.83704800	-0.60692300	1.33832300
C	1.53196300	-0.31206000	0.24835100
C	-1.89572000	2.07872300	0.27696500
C	0.46546400	1.75623400	-0.31298700
O	-1.35219100	-0.18903700	2.50527500
O	-2.91110700	-1.13483700	1.22477700
C	1.65685800	0.99240800	-0.32927000
C	2.76223200	-1.10777500	0.62826500
O	-1.79059500	3.28658300	-0.31582400
O	-2.85260000	1.80533600	0.97125500
C	-2.26237600	-0.23593100	3.59152000
C	2.89187600	1.60112300	-0.75972300
O	3.40178600	-0.56617700	1.66173500
O	3.09627100	-2.13845200	0.10328100
C	-2.83475900	4.20057800	-0.03759100
H	-1.72743900	0.15318000	4.46335900
H	-3.13540800	0.39096200	3.36368100
H	-2.59748500	-1.26491900	3.77731700
O	3.93146200	0.72510400	-0.80370300
O	3.04637700	2.76274800	-1.09216100
C	4.60182000	-1.21528000	2.05427900
H	-2.59715800	5.11904400	-0.58405700
H	-2.89952600	4.40794600	1.03971600
H	-3.80134800	3.80026800	-0.37191300
C	5.18523100	1.26303100	-1.17335100
H	4.40301900	-2.25225300	2.35555100
H	5.31802500	-1.21875300	1.22084100
H	5.00216500	-0.64451000	2.89736400
H	5.50833600	2.03640400	-0.46205300
H	5.14116900	1.71169400	-2.17522000
H	5.89528200	0.42903000	-1.16783000
N	0.43199200	-0.90010500	0.57793300
H	0.53625000	2.82631300	-0.51832800
C	-1.32828100	-1.06699800	-1.11946800
C	-2.82549900	-0.80843400	-1.39590900
C	-0.99662600	-2.56640000	-0.91041700
C	-0.60346100	-0.50384400	-2.35264600
O	-3.19315200	0.33306700	-1.55833600
C	-3.77187400	-1.96255100	-1.60540200
O	-1.65793100	-3.25863900	-0.17273200
C	0.18414000	-3.16180300	-1.64318300
H	-0.86829800	0.55222500	-2.47271600
H	0.48583700	-0.58632700	-2.25302600
H	-0.91439100	-1.04304700	-3.26035500
H	-4.73657500	-1.56650100	-1.94197900
H	-3.36468400	-2.65699400	-2.35602300
H	-3.87297800	-2.51530400	-0.66524300
H	0.10185600	-3.02006400	-2.72899200
H	1.10980200	-2.67635900	-1.30017800
H	0.22752600	-4.23164300	-1.40861800

Py-4-2356-R3-Nu3

C	-0.84502800	0.38822200	0.15774000
C	-0.23700800	1.74559500	-0.17752600
C	-1.88538300	0.54251000	1.28481500
C	1.38291300	-0.40287700	0.37277700
C	-1.07108300	2.93526300	-0.11550700
C	1.10030200	1.81805400	-0.47423200
O	-1.32884000	1.13760700	2.33842300
O	-3.02411500	0.15586700	1.28126600
C	1.95915900	0.71480200	-0.31667700
C	2.25352400	-1.52444100	0.89685700
O	-0.44099200	4.06625400	-0.49711800
O	-2.23413400	2.96001000	0.24854200
C	-2.20424200	1.41758100	3.41808100
C	3.33546700	0.81497900	-0.75168400
O	3.04477200	-1.12070800	1.88804900
O	2.19779100	-2.66134000	0.50707600
C	-1.21223300	5.25196800	-0.44712400
H	-1.60343300	1.91795800	4.18352900
H	-3.01688200	2.07495900	3.07934000
H	-2.63806500	0.49178700	3.81862400
O	4.01448000	-0.35454300	-0.64482700
O	3.87417300	1.80941400	-1.20071400
C	3.94554500	-2.09507700	2.39281100
H	-0.56098600	6.05965400	-0.79627500
H	-1.55091400	5.45685700	0.57780500
H	-2.09606100	5.17277000	-1.09503900
C	5.37556300	-0.32256000	-1.02710900
H	3.39999500	-2.95844200	2.79582500
H	4.61398300	-2.44042200	1.59168600
H	4.52130700	-1.60580900	3.18398900
H	5.94190300	0.38765800	-0.40824500
H	5.47997200	-0.02535000	-2.07947300
H	5.76212000	-1.33724900	-0.88456300
N	0.14025200	-0.55087500	0.68494300
H	1.53005000	2.76734900	-0.79942100
C	-1.54141300	-0.29144800	-1.11219600
C	-2.08197300	-1.70423700	-0.82611800
C	-0.49786400	-0.44802200	-2.15082000
C	-2.60437500	0.63454300	-1.73347500
C	-3.45584800	-1.94417300	-0.71604200
C	-1.20432500	-2.79084200	-0.70689600
N	0.25531800	-0.57957400	-3.01549500
H	-2.13164900	1.55260700	-2.10324400
H	-3.08651600	0.12831600	-2.58074600
H	-3.35513500	0.90718400	-0.98526300
C	-3.94135900	-3.23040400	-0.48219700
H	-4.15969700	-1.11732600	-0.79139400
C	-1.68907900	-4.07633500	-0.46831200
H	-0.12811600	-2.63288400	-0.78499300
C	-3.06019800	-4.30326900	-0.35542900
H	-5.01751900	-3.39121100	-0.39764000
H	-0.98493400	-4.90469600	-0.37381100

H	-3.43987400	-5.31006500	-0.17283800

Py-4-2356-R3-Nu2

C	-0.27755500	1.04204800	-0.51394400
C	-0.61914300	1.47697300	0.89704000
C	0.13189500	2.21923600	-1.40786500
C	-2.21118000	-0.29014000	-0.45205000
C	0.09957000	2.60333000	1.47626800
C	-1.45785100	0.66781500	1.61067100
O	-0.82300500	3.14839300	-1.40589300
O	1.12110100	2.28587900	-2.08270200
C	-2.16970600	-0.37828900	0.97909300
C	-3.33507000	-0.94457800	-1.22858100
O	-0.18124500	2.81442100	2.77371200
O	0.89022100	3.30266100	0.87358000
C	-0.52725800	4.32496400	-2.13945800
C	-2.97553500	-1.28358300	1.77272500
O	-4.52098500	-0.44751100	-0.87550500
O	-3.18708000	-1.76739300	-2.09092500
C	0.56632000	3.83960600	3.40275600
H	-1.39365500	4.98468200	-2.03182500
H	0.37207900	4.80560800	-1.72990900
H	-0.35528100	4.09160000	-3.19876600
O	-3.53040900	-2.27751600	1.03564700
O	-3.14320100	-1.22843500	2.97566500
C	-5.64728100	-1.03795800	-1.50595300
H	0.24222900	3.86300300	4.44808800
H	0.37787000	4.81162600	2.92663300
H	1.64185000	3.62388100	3.34286500
C	-4.38037300	-3.16878600	1.73138600
H	-5.59149400	-0.91739600	-2.59590900
H	-5.69259400	-2.11042100	-1.27013600
H	-6.52905500	-0.52468000	-1.11100800
H	-5.24262600	-2.63684700	2.15810200
H	-3.84059300	-3.66651500	2.54820200
H	-4.72050000	-3.90887500	0.99952400
N	-1.43269300	0.43520600	-1.18418800
H	-1.61757200	0.84426400	2.67628300
C	0.82245500	-0.06381700	-0.44903100
Br	1.21408800	-0.88217100	-2.16535500
S	2.41062300	0.31780900	0.40703500
H	0.40384700	-0.86854600	0.17253800
O	3.26643100	1.17596400	-0.39915600
O	2.08487000	0.68971300	1.78466700
C	3.18477800	-1.29163600	0.49499900
C	4.21458100	-1.59800900	-0.39091600
C	2.76859100	-2.17985700	1.48497400
C	4.83333000	-2.84259900	-0.29010800
H	4.51577900	-0.86538700	-1.14058100
C	3.39075100	-3.42419500	1.57012200
H	1.97896700	-1.89289300	2.18172700
C	4.41850500	-3.75269200	0.68412800

H	5.64177200	-3.10301600	-0.97419700
H	3.07783200	-4.13511600	2.33557500
H	4.90442100	-4.72678000	0.75673400

Py-4-2356-R3-Nu4

C	-1.14534500	-0.53823300	-0.23760300
C	-1.18373100	-0.98944100	-1.72882600
C	-0.48452700	-2.29151700	-2.04371000
H	-2.24349400	-1.03800200	-2.02714100
H	-0.70120200	-0.19452100	-2.31318700
C	-1.22805100	0.98284100	-0.16037600
C	-2.30370600	-1.17755600	0.53862800
C	1.14134000	-0.33269200	0.25990400
C	-0.98420900	-3.55067900	-1.39274700
C	0.56560800	-2.30417500	-2.87249000
C	-2.52655600	1.62852900	-0.13962500
C	-0.05515000	1.67979300	-0.27983100
O	-2.32437700	-0.75573300	1.80406400
O	-3.08503400	-1.98790900	0.11104300
C	1.19124900	1.01799000	-0.24088900
C	2.38179900	-0.98490800	0.83071200
H	-0.83322200	-3.48205800	-0.30493800
H	-2.06380000	-3.67850600	-1.55373300
H	-0.44635700	-4.43075000	-1.77091700
H	0.94544000	-1.38166600	-3.31948800
H	1.08397000	-3.23473700	-3.11785300
O	-2.46634300	2.97914200	-0.14861000
O	-3.59992900	1.05033500	-0.12305800
C	-3.41327000	-1.21277100	2.58706200
C	2.40579500	1.75537100	-0.50245500
O	2.84755500	-0.31614600	1.88798700
O	2.86691600	-2.00790900	0.42725300
C	-3.70892900	3.65454800	-0.16582200
H	-3.29111800	-0.76913600	3.58010600
H	-4.36162000	-0.88655600	2.13761400
H	-3.41232000	-2.30884300	2.65774900
O	3.51021200	0.96331100	-0.52917100
O	2.49471500	2.95053800	-0.71939500
C	4.05143900	-0.81494900	2.44805000
H	-3.47969100	4.72510600	-0.18253400
H	-4.30179800	3.41093300	0.72675000
H	-4.29401400	3.38118100	-1.05507800
C	4.74424700	1.62288300	-0.72653100
H	3.92205100	-1.84830700	2.79657600
H	4.85446600	-0.79377600	1.69779100
H	4.29959500	-0.15909600	3.28803700
H	4.94271700	2.33909900	0.08372600
H	4.74981100	2.16946000	-1.67944300
H	5.51525900	0.84513800	-0.73621100
N	0.07802600	-1.04385500	0.39354400
H	-0.06859500	2.76677500	-0.38424200

Py-4-2356-R4

C	0.00000000	1.12854200	0.69761300
C	0.00000000	1.18779800	-0.69638300
C	0.00000000	0.00000000	-1.41231500
C	0.00000000	-1.12854200	0.69761300
C	0.00000000	-1.18779800	-0.69638300
F	0.00000000	-2.25426700	1.38794500
F	0.00000000	-2.36100700	-1.30750300
F	0.00000000	2.36100700	-1.30750300
N	0.00000000	0.00000000	1.35911400
H	0.00000000	0.00000000	-2.50262600
F	0.00000000	2.25426700	1.38794500

Py-4-2356-R4-Nu1

C	0.13736400	-0.04893200	-0.24225500
C	0.73362900	-1.35222300	0.13018400
C	2.07614900	-1.55277300	0.15280100
C	2.30932600	0.79468500	-0.06862600
C	2.92372700	-0.43890800	-0.03484000
F	3.09390100	1.88943600	-0.04667100
F	4.27282300	-0.57459500	-0.02940300
F	-0.11481300	-2.37420800	0.42206000
F	-0.17996700	-0.08908300	-1.68323800
N	1.01983800	1.01216100	-0.03834900
H	2.48717900	-2.54476000	0.34809900
C	-1.26425000	0.20085300	0.41323500
C	-2.29927100	-0.91744600	0.12690700
C	-1.86019400	1.49923000	-0.17726000
C	-1.08151600	0.32404900	1.92632700
O	-3.03907700	-1.26041100	1.02154000
C	-2.43063200	-1.52489800	-1.24656900
O	-2.67361100	1.46567800	-1.07294300
C	-1.43158100	2.81169400	0.42806900
H	-0.75353700	-0.64339300	2.33212500
H	-0.31594500	1.07585400	2.15091800
H	-2.02621600	0.59074400	2.41683700
H	-3.29330800	-2.20196000	-1.23784700
H	-2.55522500	-0.72717900	-1.98577700
H	-1.51656300	-2.07290300	-1.50259100
H	-1.79532300	2.88033800	1.46350300
H	-0.33508200	2.85103200	0.44679500
H	-1.85734400	3.62903800	-0.16497600

Py-4-2356-R4-Nu3

C	-0.61181100	0.35355500	-0.46542500
C	-1.86525600	1.11069300	-0.26372800
C	-3.05348100	0.48449800	-0.06476000
C	-1.82102400	-1.52041400	0.20448400
C	-3.05521900	-0.91628600	0.11243300
F	-1.78061100	-2.82036500	0.55319700
F	-4.21016500	-1.59608400	0.31142300

F	-1.81713500	2.46475700	-0.34135800
F	-0.41802400	0.26240600	-1.95268700
N	-0.66379500	-0.92527400	0.06030900
H	-3.97965000	1.05895300	-0.00968900
C	0.70265800	1.10943900	-0.03121200
C	1.90437600	0.15759400	0.01873000
C	0.43780300	1.64316500	1.31918800
C	1.05305900	2.29533900	-0.95615000
C	2.28984800	-0.51668700	-1.14961700
C	2.67248100	-0.01418900	1.17224500
N	0.23867200	2.08922600	2.36484700
H	0.22450500	3.00833600	-1.01719100
H	1.94757100	2.80668800	-0.57499500
H	1.26188100	1.90623600	-1.95809200
C	3.41567400	-1.33430500	-1.15762300
H	1.67744200	-0.41256000	-2.04435300
C	3.80421600	-0.83424700	1.16334500
H	2.39207100	0.49468200	2.09557900
C	4.18260900	-1.49541400	-0.00099500
H	3.69484900	-1.85492200	-2.07541400
H	4.38892800	-0.95213100	2.07723300
H	5.06607700	-2.13582100	-0.00983000

Py-4-2356-R4-Nu2

C	1.46211500	0.68826600	0.05370500
C	2.35648200	0.89195900	-1.09889500
C	3.63468300	0.43327400	-1.11734400
C	3.14632300	-0.66129700	0.92831300
C	4.08439000	-0.34880800	-0.03345600
F	3.51475200	-1.50334700	1.91094000
F	5.34616100	-0.84035800	0.00176800
F	1.85841300	1.60882100	-2.13659900
F	1.32032300	2.00049900	0.73345300
N	1.90123600	-0.26469500	0.94909600
H	4.29151500	0.65698500	-1.95958400
C	-0.01714200	0.50057900	-0.40526600
Br	-0.31114900	-1.30218900	-1.02124700
S	-1.23284100	1.01102200	0.88033600
H	-0.23193500	1.17593800	-1.24070400
O	-0.86604300	0.45044900	2.17378500
O	-1.41816800	2.45115100	0.71823000
C	-2.77140800	0.24654700	0.38815000
C	-3.17425200	-0.92892800	1.01702700
C	-3.54796700	0.87011100	-0.58559600
C	-4.38747800	-1.50363300	0.64361100
H	-2.53955900	-1.37518100	1.78353000
C	-4.75700200	0.28233800	-0.95404600
H	-3.21111100	1.80608800	-1.03356000
C	-5.17251000	-0.90162400	-0.34192200
H	-4.72174300	-2.42420700	1.12348600
H	-5.37994900	0.75420500	-1.71477200
H	-6.12074900	-1.35674700	-0.63168600

Py-4-2356-R4-Nu4

C	0.36589300	-0.22983400	0.82777200
C	1.88277300	0.00851900	0.84523200
C	-0.05491800	-1.31349000	-0.08977100
C	2.42453500	0.58127100	-0.44187800
H	2.07243800	0.69518600	1.68353900
H	2.36148700	-0.95346700	1.07168100
C	-1.28682800	-1.34840000	-0.65391500
C	2.35805500	2.07646100	-0.57752400
C	2.90652500	-0.20867300	-1.40754200
C	-1.58550600	0.86354400	0.16179500
C	-2.13487700	-0.22844600	-0.47372700
H	2.98459500	2.55662700	0.19186800
H	2.69315200	2.41478300	-1.56734600
H	1.32420600	2.40576300	-0.39796100
H	2.92671100	-1.29409700	-1.28578400
H	3.27466800	0.20728900	-2.34910600
F	-2.31883500	1.99731200	0.19984900
F	-3.38159100	-0.20204300	-1.01408000
F	0.81989500	-2.33565700	-0.27448400
F	0.14677000	-0.75035200	2.20478300
N	-0.38299900	0.94027400	0.67404900
H	-1.60632600	-2.20304100	-1.25250100

Py-4-2356-R5

C	0.66258700	0.02113300	1.12481900
C	-0.72811300	-0.02713500	1.18433200
C	-1.44809700	-0.04340400	0.00000000
C	0.66258700	0.02113300	-1.12481900
C	-0.72811300	-0.02713500	-1.18433200
N	1.49765800	0.14438600	-2.35124500
O	2.42384700	-0.60813600	-2.44287000
O	1.17921200	1.02177900	-3.10916800
N	-1.46114800	-0.14487200	-2.45798500
O	-0.90733300	-0.74529700	-3.34049200
O	-2.56047200	0.33915800	-2.48847600
N	-1.46114800	-0.14487200	2.45798500
O	-2.56047200	0.33915800	2.48847600
O	-0.90733300	-0.74529700	3.34049200
N	1.49765800	0.14438600	2.35124500
O	1.17921200	1.02177900	3.10916800
O	2.42384700	-0.60813600	2.44287000
N	1.33407700	0.04294400	0.00000000
H	-2.53885700	-0.08144100	0.00000000

Py-4-2356-R5-Nu1

C	-0.51009600	-0.06841700	0.40543000
C	-0.08149700	1.37405400	0.28095800
C	1.18981000	1.72142500	-0.07159400

C	1.78339700	-0.56385000	0.23128700
C	2.17482200	0.73414500	-0.19162800
N	2.81545900	-1.62718800	0.44659400
O	2.74148300	-2.60013700	-0.25683400
O	3.59743900	-1.44378200	1.34049700
N	3.46079000	1.03409200	-0.70575100
O	4.21244500	0.10165500	-0.94050800
O	3.74904600	2.19964000	-0.90371400
N	-1.03627500	2.40770400	0.52839200
O	-0.73440500	3.56530400	0.33456600
O	-2.12953600	2.05673500	0.95174700
N	-1.25633400	-0.23317100	1.78130200
O	-0.71695200	0.26956800	2.72655000
O	-2.25952300	-0.89959500	1.81842500
N	0.61727000	-0.94074600	0.56777800
H	1.43711300	2.76760100	-0.24935100
C	-1.41216800	-0.59153100	-0.77381700
C	-2.52463100	0.44272600	-1.13002000
C	-2.00927400	-2.00607600	-0.61397400
C	-0.50231700	-0.70407200	-2.01785100
O	-2.24579400	1.31297900	-1.92066200
C	-3.90774100	0.36071000	-0.53575700
O	-3.06423000	-2.23284300	-1.15934400
C	-1.22257800	-3.12324400	0.02795000
H	-0.02106600	0.26032500	-2.22180800
H	0.26393800	-1.47736400	-1.88368500
H	-1.12082700	-0.96402100	-2.88608000
H	-4.29233800	1.38292400	-0.44846400
H	-4.53591100	-0.21170600	-1.23132700
H	-3.91826400	-0.14515000	0.43358500
H	-0.21177500	-3.18698100	-0.39489300
H	-1.10091500	-2.94574700	1.10271900
H	-1.77246800	-4.05614200	-0.14060100

Py-4-2356-R5-Nu3

C	-0.19540300	0.74583300	0.33323700
C	0.99696300	1.62063900	0.01547000
C	2.21603900	1.07764700	-0.28136400
C	1.35998900	-1.00904200	0.47457800
C	2.42516500	-0.29627200	-0.13846100
N	1.58576500	-2.39858800	0.98381200
O	0.96070300	-3.27879700	0.45429400
O	2.33549600	-2.51039800	1.91655300
N	3.62828900	-0.90847500	-0.57021600
O	3.69073600	-2.12656600	-0.53871600
O	4.53315300	-0.19609800	-0.96319700
N	0.87088800	3.04679700	0.01976900
O	1.79572900	3.72614000	-0.37754800
O	-0.17259700	3.51686100	0.44510700
N	-0.91551400	1.32926800	1.59582000
O	-0.21200400	1.44286000	2.56234200
O	-2.08650700	1.58805100	1.54412400

N	0.20722700	-0.56025900	0.76677200
H	3.03050700	1.72157100	-0.61153900
C	-1.23041200	0.58333600	-0.86636600
C	-2.39516400	-0.37615200	-0.55029200
C	-0.42575300	-0.05453400	-1.93455100
C	-1.70567100	1.92797600	-1.44890900
C	-3.69091800	0.11202100	-0.35161000
C	-2.18471000	-1.76051800	-0.50668100
N	0.19598300	-0.53041400	-2.78159300
H	-0.85610100	2.50235500	-1.83866800
H	-2.39165900	1.72650500	-2.28185000
H	-2.21361900	2.52229300	-0.68455100
C	-4.74886200	-0.76049500	-0.10267800
H	-3.88237600	1.18351100	-0.37396300
C	-3.24259700	-2.63245100	-0.25299000
H	-1.18558800	-2.16789100	-0.66525000
C	-4.52915600	-2.13625900	-0.04999000
H	-5.75118000	-0.35819700	0.05131500
H	-3.05507600	-3.70658900	-0.22007500
H	-5.35811200	-2.81865900	0.14400700

Py-4-2356-R5-Nu2

C	-0.79965000	0.80934700	-0.56648000
C	-1.35829700	1.39288400	0.70567600
C	-2.21652300	0.66891600	1.47773800
C	-2.51641100	-0.76627600	-0.40029800
C	-2.74794100	-0.52959700	0.98120100
N	-3.40064000	-1.71671400	-1.14654700
O	-2.87126500	-2.66368900	-1.66102300
O	-4.56436000	-1.41831300	-1.20952200
N	-3.53792900	-1.38336700	1.79123500
O	-3.87002700	-2.46128200	1.32523900
O	-3.82751900	-1.01431500	2.91377900
N	-0.88218900	2.65940300	1.17144400
O	-1.29221400	3.09386000	2.22529500
O	-0.08121000	3.23967300	0.45982200
N	-0.67839300	1.92688800	-1.64356700
O	-1.70042900	2.51784400	-1.86886600
O	0.37574700	2.09277500	-2.18605200
N	-1.71639600	-0.12876700	-1.15802100
H	-2.49852300	1.03015900	2.46638000
C	0.55104700	0.06217900	-0.36108400
Br	1.20629400	-0.82518900	-1.94599100
S	1.92515400	0.93050200	0.52235500
H	0.30842700	-0.74238900	0.34830700
O	2.54851200	1.93952700	-0.31425100
O	1.37484000	1.26196700	1.83565000
C	3.09894700	-0.38693600	0.76738400
C	4.20395000	-0.47089500	-0.07707300
C	2.90249600	-1.26781600	1.82964600
C	5.13226800	-1.48576300	0.14355500
H	4.32415400	0.25089300	-0.88576200

C	3.83662900	-2.28152900	2.03392900
H	2.04219400	-1.14941700	2.49032700
C	4.94511700	-2.38882100	1.19210700
H	6.00531400	-1.57107300	-0.50409600
H	3.70316400	-2.98289800	2.85802500
H	5.67494000	-3.18231500	1.35863900

Py-4-2356-R5-Nu4

C	1.08380500	-0.57410400	-0.23840800
C	1.70701400	-1.27796300	0.97983400
C	1.04887700	0.92735900	-0.15214000
C	0.87248600	-1.07024800	2.22638700
H	1.77665200	-2.34563500	0.73724800
H	2.72335600	-0.88738200	1.11196200
C	-0.11545100	1.62479900	-0.03533600
C	-0.13213800	-2.14215400	2.55129400
C	1.03707000	0.01704800	2.98654300
C	-1.25497500	-0.43815200	-0.42712100
C	-1.34003300	0.95025000	-0.11938500
H	0.38017500	-3.10474200	2.70812700
H	-0.70013600	-1.89636900	3.45808500
H	-0.83708200	-2.28943100	1.71912200
H	1.78428100	0.77679600	2.74070600
H	0.42947300	0.18112000	3.87962000
N	-2.49589900	-1.19093500	-0.79891000
O	-2.84223400	-2.07685100	-0.06345100
O	-3.01257000	-0.88078400	-1.83951200
N	-2.55823500	1.61283700	0.15476900
O	-3.57160900	0.93662300	0.25048400
O	-2.54401400	2.82325500	0.29657600
N	2.29095000	1.62754700	-0.09785200
O	2.29929300	2.82463000	0.11480700
O	3.30667700	0.97099200	-0.28054800
N	1.93521000	-0.96005900	-1.50761500
O	1.72157700	-0.30695200	-2.49535300
O	2.68294000	-1.89797800	-1.43764100
N	-0.20393800	-1.14366300	-0.53310600
H	-0.09374100	2.70106100	0.13124800

Py-4-3256-R1

C	0.00000000	1.20549900	-0.73290200
C	0.00000000	0.00000000	-1.42697600
C	0.01431700	1.14570900	0.66584900
C	0.00000000	-1.20549900	-0.73290200
C	-0.01431700	-1.14570900	0.66584900
C	-0.00702500	-2.49877300	-1.52878600
C	0.00227800	-2.38717300	1.55750100
C	-0.00227800	2.38717300	1.55750100
C	0.00702500	2.49877300	-1.52878600
F	1.21642800	3.04684600	-1.55906700
F	-0.35927400	2.27045300	-2.78781100

F	-0.83996000	3.38637000	-1.01731600
F	-1.23300100	2.88366700	1.64334300
F	0.79286100	3.33602400	1.06805900
F	0.40998300	2.09818400	2.77880100
F	1.23300100	-2.88366700	1.64334300
F	-0.79286100	-3.33602400	1.06805900
F	-0.40998300	-2.09818400	2.77880100
F	-1.21642800	-3.04684600	-1.55906700
F	0.35927400	-2.27045300	-2.78781100
F	0.83996000	-3.38637000	-1.01731600
N	0.00000000	0.00000000	1.32436000
H	0.00000000	0.00000000	-2.51676800

Py-4-3256-R1-Nu1

C	-0.80848500	-0.24922000	-0.32963600
C	0.40907800	-1.09022500	-0.62233200
C	-0.30840100	1.19631200	-0.28040100
C	1.66150500	-0.69468200	-0.28382200
C	1.89532600	0.64125100	0.17289100
C	2.77501500	-1.69620900	-0.43739400
C	3.22684600	1.20074000	0.53382700
C	-1.19022200	2.37018600	-0.61454600
C	-1.75975000	-0.48878900	-1.51812100
F	-1.22995000	-0.03786500	-2.65421000
F	-1.98732200	-1.80008200	-1.70989400
F	-2.95559800	0.08330200	-1.38210800
F	-2.44381900	2.26516700	-0.13210400
F	-1.34503200	2.54899600	-1.94232100
F	-0.69966700	3.51354000	-0.13792000
F	3.98753300	1.56840600	-0.52083400
F	3.97598900	0.32397700	1.22731100
F	3.11388000	2.29784600	1.29135300
F	3.13614800	-2.21533400	0.74335500
F	2.42945700	-2.72858000	-1.21344800
F	3.87240500	-1.15986400	-0.98215700
N	0.91275700	1.52576800	0.03634600
H	0.24967300	-2.07801700	-1.05024300
C	-1.50468800	-0.74858500	1.07901400
C	-2.02038400	-2.19955400	0.88975700
C	-2.59552200	0.22714200	1.56783400
C	-0.38706800	-0.80846800	2.13363200
O	-1.21632500	-3.10048200	0.98086800
C	-3.46918100	-2.52284700	0.61064900
O	-3.76432600	0.08363500	1.29865500
C	-2.16761100	1.36326600	2.47426400
H	0.33256700	-1.58687200	1.86869700
H	0.14095500	0.14943800	2.20863900
H	-0.81144600	-1.05778200	3.11697900
H	-3.51528900	-3.56929000	0.28696400
H	-4.04567400	-2.39512300	1.53787300
H	-3.91537000	-1.85109200	-0.12744300
H	-1.96086600	0.96671000	3.47912700

H	-1.25252800	1.85592800	2.12099900
H	-2.99105400	2.08301500	2.53090700

Py-4-3256-R1-Nu3

C	-0.77285400	-0.55638800	0.33535400
C	0.11584200	0.55015200	0.83742200
C	0.15762800	-1.71205100	-0.01250800
C	1.42053500	0.65663000	0.48332500
C	2.06958500	-0.41958000	-0.20242900
C	2.13183200	1.93741100	0.83273300
C	3.51457700	-0.44574800	-0.55985200
C	-0.38236100	-3.10311600	-0.17044300
C	-1.72063800	-0.89878400	1.49764000
F	-1.03299200	-1.25951800	2.58114700
F	-2.46466200	0.16174200	1.84499200
F	-2.57447500	-1.88662200	1.23137700
F	-1.52322300	-3.12313100	-0.89242400
F	-0.68444800	-3.69625800	1.00171300
F	0.47215900	-3.91444100	-0.78813100
F	4.34156500	-0.69587700	0.47994300
F	3.93508700	0.72631800	-1.07279100
F	3.78614200	-1.38635100	-1.46904700
F	2.28743800	2.71770100	-0.24687000
F	1.45945300	2.66708100	1.72837200
F	3.34885700	1.72795400	1.34312100
N	1.41609500	-1.57196100	-0.31663300
H	-0.35300500	1.33658900	1.42612600
C	-1.64298100	-0.00341200	-0.95481400
C	-1.93576200	1.49647300	-0.81079000
C	-2.93134900	-0.69551200	-1.14286700
C	-0.81415900	-0.28068200	-2.22539200
C	-0.96167300	2.43569600	-1.17706500
C	-3.14572000	1.96608800	-0.28701500
N	-3.94784300	-1.19902200	-1.35585700
H	-0.78277300	-1.35650400	-2.42966400
H	-1.26487100	0.23596200	-3.08322900
H	0.21674800	0.06980700	-2.09140700
C	-1.18187200	3.80101600	-1.00051200
H	-0.00795400	2.10791800	-1.58910800
C	-3.37163000	3.33166900	-0.11908000
H	-3.92332600	1.25685000	-0.00032100
C	-2.38790100	4.25563500	-0.46928700
H	-0.40100700	4.50966100	-1.28107200
H	-4.32410300	3.67221700	0.28986500
H	-2.56219100	5.32402000	-0.33291200

Py-4-3256-R1-Nu2

C	-0.25873400	0.83203100	0.80807900
C	-1.14176200	-0.31808800	1.22156100
C	-0.88905500	1.47999300	-0.43021900
C	-2.16032300	-0.78257600	0.45814200

C	-2.51784500	-0.13420600	-0.76694200
C	-2.87338300	-2.02194700	0.93757700
C	-3.69165900	-0.49325000	-1.60806300
C	-0.30696500	2.74873400	-0.98019100
C	-0.21908400	1.85966100	1.97354800
F	-1.35671000	2.54469700	2.03078900
F	-0.06336100	1.28259400	3.16791400
F	0.78051800	2.73988700	1.83325500
F	1.04805400	2.74182900	-0.98805400
F	-0.63511600	3.84297400	-0.25924700
F	-0.69648100	2.99181600	-2.22526600
F	-4.87974300	-0.08100200	-1.10527600
F	-3.82698700	-1.82257500	-1.77496700
F	-3.61436000	0.04423400	-2.82850700
F	-2.54719000	-3.09666200	0.21455200
F	-2.58353500	-2.31871600	2.20950600
F	-4.20378600	-1.89122400	0.87260800
N	-1.88531900	0.98761800	-1.09542600
H	-0.89439300	-0.81069600	2.16000100
C	1.21900200	0.37276400	0.54673900
Br	2.02624800	-0.59606200	2.02854300
S	1.36536800	-0.70539400	-0.94255300
H	1.87483700	1.22587400	0.34670500
O	0.77487200	-2.00593300	-0.65216700
O	0.92016100	0.06625000	-2.09764700
C	3.12879200	-0.90620300	-1.10222400
C	3.71377600	-2.11423200	-0.73329400
C	3.86618000	0.14869100	-1.63814800
C	5.09015100	-2.26261300	-0.89566700
H	3.09147200	-2.91343600	-0.32877700
C	5.24240400	-0.01061000	-1.78588000
H	3.36459100	1.07038000	-1.94010600
C	5.84940900	-1.21272700	-1.41543800
H	5.57106100	-3.20034500	-0.61538800
H	5.84096500	0.80144100	-2.19979600
H	6.92669500	-1.33380700	-1.53755400

Py-4-3256-R1-Nu4

C	-1.18664800	-0.76109800	0.08111200
C	-1.61591100	-1.00646600	1.56922200
C	0.15176400	-1.40480500	-0.19741300
C	-0.71153600	-0.35488700	2.58785600
H	-2.64764100	-0.64887800	1.70209300
H	-1.61234500	-2.09664600	1.72465800
C	-1.05126900	0.71312000	-0.30199500
C	1.30084300	-0.69073100	-0.23976900
C	-0.97633100	1.08821600	2.92899200
C	0.27629400	-1.04716600	3.16594700
C	1.26318600	0.74563600	-0.21926300
H	-2.02799700	1.23030400	3.22252600
H	-0.33065200	1.42422100	3.75134200
H	-0.79594900	1.73743600	2.05940700

H	0.45723400	-2.09472600	2.91218800
H	0.94159500	-0.58224200	3.89789700
C	2.59596000	-1.45407100	-0.31320700
C	2.45228800	1.62648400	-0.33707700
C	-2.27560500	1.56096800	-0.45336600
C	-2.22141400	-1.48975000	-0.78744300
F	-2.00595000	-1.30841500	-2.09000200
F	-2.18854400	-2.81611100	-0.57170000
F	-3.47874900	-1.10715900	-0.53506100
F	-3.16186700	1.37325600	0.55316800
F	-2.97092200	1.30772300	-1.58241600
F	-1.99893100	2.86356700	-0.47034800
F	2.93455400	1.76191200	-1.59706200
F	3.49832800	1.18477000	0.38842400
F	2.19603500	2.87375800	0.07637700
F	3.24547800	-1.45022300	0.85764800
F	2.41460700	-2.74099800	-0.63518800
F	3.43360700	-0.94989200	-1.22739900
N	0.08112200	1.34860500	-0.34323700
H	0.17936000	-2.49210100	-0.26698300

Py-4-3256-R2

C	0.00000000	1.19838300	-0.72714400
C	0.00000000	0.00000000	-1.44018000
C	0.00000000	1.13882000	0.68155200
C	0.00000000	-1.19838300	-0.72714400
C	0.00000000	-1.13882000	0.68155200
C	0.00000000	-2.46478600	-1.40920500
N	0.00000000	-3.48105500	-1.95427000
C	0.00000000	-2.36205100	1.46038300
N	0.00000000	-3.34139500	2.06801700
C	0.00000000	2.36205100	1.46038300
N	0.00000000	3.34139500	2.06801700
C	0.00000000	2.46478600	-1.40920500
N	0.00000000	3.48105500	-1.95427000
N	0.00000000	0.00000000	1.35873600
H	0.00000000	0.00000000	-2.52957900

Py-4-3256-R2-Nu1

C	-0.42504000	0.45073100	-0.53997500
C	0.54968200	-0.55927300	-1.10965100
C	0.40476900	1.59820500	0.06916100
C	1.84453900	-0.58694700	-0.70752300
C	2.39687200	0.44856300	0.13447900
C	2.71338200	-1.61799000	-1.21996000
N	3.41331600	-2.44458600	-1.62126400
C	3.76070000	0.42144400	0.55464100
N	4.86770600	0.38119200	0.89983400
C	-0.23448400	2.87010200	0.24593700
N	-0.76607300	3.89416000	0.36449600
C	-1.21704200	1.00892800	-1.66517200

N	-1.82809000	1.44068500	-2.54428900
N	1.66557300	1.52345800	0.40579700
H	0.17732200	-1.28461200	-1.83119800
C	-1.49054800	-0.21670300	0.45593900
C	-0.77238500	-0.98587700	1.58252600
C	-2.32948600	-1.20771700	-0.37006200
C	-2.33904100	0.85604800	1.15297800
O	0.33001400	-0.67068400	1.94803000
C	-1.54303000	-2.09407900	2.26152600
O	-1.82447400	-2.25301300	-0.71324900
C	-3.74678700	-0.85641800	-0.73897700
H	-1.71023100	1.46819900	1.81208100
H	-2.81916000	1.52388800	0.42350200
H	-3.12445400	0.39604400	1.76818400
H	-1.15235700	-2.23635300	3.27503600
H	-2.62312600	-1.89419700	2.28676600
H	-1.39083900	-3.01497200	1.68090400
H	-4.36636100	-0.81649600	0.16926800
H	-3.79687000	0.13267900	-1.21367600
H	-4.13787800	-1.62306300	-1.41633000

Py-4-3256-R2-Nu3

C	-0.12999000	0.64432200	0.49223500
C	-0.60036200	-0.72563100	0.92071700
C	-1.38158100	1.49484000	0.24460500
C	-1.81275200	-1.18538000	0.51188200
C	-2.76103500	-0.30902200	-0.13248000
C	-2.19736900	-2.53946600	0.82569500
N	-2.50232700	-3.62822400	1.06093200
C	-4.02668400	-0.78759800	-0.59009100
N	-5.04203300	-1.19435700	-0.97515600
C	-1.28931900	2.92044900	0.39190100
N	-1.21922000	4.06758000	0.53865300
C	0.67260500	1.23630500	1.58354500
N	1.29024200	1.69958800	2.44045400
N	-2.53194200	1.00440100	-0.13561200
H	0.06077800	-1.34188900	1.52889200
C	0.82009500	0.55075900	-0.81235500
C	1.99727000	-0.38109000	-0.52232100
C	1.32091800	1.90841900	-1.10058600
C	-0.01288100	0.10539700	-2.02439800
C	1.88326900	-1.75733900	-0.75717100
C	3.18322700	0.10983400	0.03808700
N	1.69826900	2.97294400	-1.33634900
H	-0.77650900	0.85788100	-2.25858900
H	0.64169700	-0.02342900	-2.89621700
H	-0.51776500	-0.84511400	-1.81690900
C	2.92932300	-2.62083600	-0.43271200
H	0.97407000	-2.16963600	-1.19390600
C	4.22973800	-0.75347100	0.35804100
H	3.29327000	1.17956400	0.22566500
C	4.10565100	-2.12256500	0.12522300

H	2.82153800	-3.68962000	-0.62254400
H	5.14664200	-0.35008600	0.78975100
H	4.92460600	-2.79877300	0.37448900

Py-4-3256-R2-Nu2

C	-1.19118800	0.72958100	0.71067300
C	-1.93096700	-0.44973200	1.29383000
C	-2.18866900	1.42409100	-0.22780100
C	-2.74878000	-1.14406400	0.45790000
C	-3.15438000	-0.57314100	-0.80448200
C	-3.31907500	-2.39529600	0.88751600
N	-3.76736900	-3.40569500	1.22187700
C	-3.98437300	-1.29878100	-1.71586700
N	-4.63752700	-1.90357000	-2.45802600
C	-2.17802400	2.85210200	-0.34832600
N	-2.17696500	4.00777100	-0.42996500
C	-0.75096800	1.65245400	1.77376800
N	-0.46731700	2.40871700	2.59567100
N	-2.97684000	0.73598400	-1.00916800
H	-1.71289000	-0.77644600	2.30905100
C	0.02355400	0.23841600	-0.14176100
Br	1.06238900	1.71541800	-0.82138100
S	1.02744800	-0.98187300	0.81590100
H	-0.35763200	-0.32816000	-1.00178500
O	1.11015300	-0.47961500	2.18551600
O	0.42767700	-2.28520000	0.54483600
C	2.65700100	-0.96995500	0.11270200
C	3.64346700	-0.18914600	0.71153900
C	2.90480100	-1.76705500	-1.00300600
C	4.92239600	-0.20276600	0.16012200
H	3.40412600	0.40944300	1.59131800
C	4.18835700	-1.76568400	-1.54529500
H	2.10904600	-2.38218900	-1.42540600
C	5.19006500	-0.98488400	-0.96560000
H	5.71280700	0.39800500	0.61090200
H	4.40831700	-2.38130300	-2.41792500
H	6.19361100	-0.98977100	-1.39324500

Py-4-3256-R2-Nu4

C	-1.00634900	-0.02502100	0.71704000
C	-2.06954000	0.92806400	0.08236100
C	0.25892900	0.73215100	1.06871000
C	-1.52116300	1.81672400	-1.00747900
H	-2.88369200	0.29657900	-0.31487600
H	-2.49965000	1.55131000	0.87996800
C	-0.63457000	-1.21129200	-0.19072900
C	1.47192700	0.35324600	0.60322900
C	-0.90075100	1.15480500	-2.20856100
C	-1.60179600	3.14668900	-0.89569200
C	1.63453800	-0.81156700	-0.24622000
H	-1.54000900	0.34185800	-2.58608200

H	-0.73410300	1.88201800	-3.01393100
H	0.07034500	0.70177600	-1.95150800
H	-2.04111300	3.61785900	-0.01308800
H	-1.23303500	3.80302600	-1.68773500
C	2.63144300	1.13963600	0.94911700
N	3.56291300	1.76519900	1.22269600
C	2.91552800	-1.21113300	-0.72575700
N	3.96584200	-1.52387700	-1.10879500
C	-1.72589600	-2.02208200	-0.64848600
N	-2.63782900	-2.64610300	-1.00088100
C	-1.61227300	-0.55938400	1.96662900
N	-2.07515400	-0.98104100	2.93677100
N	0.56509100	-1.52872200	-0.59102500
H	0.15504500	1.62237100	1.69092300

Py-4-3256-R3

C	-1.19875900	-0.94865000	0.06046800
C	-1.13812700	0.45172900	0.10661400
C	0.00009000	-1.65196300	-0.00004900
C	-2.48966400	-1.71387100	0.08140300
C	-2.39086500	1.24501300	0.39559100
C	1.19888500	-0.94851900	-0.06057900
O	-3.34583700	-1.24718400	-0.81624100
O	-2.68525600	-2.66357000	0.78993900
O	-2.36364200	2.45381400	-0.13237500
O	-3.29141500	0.80719700	1.06356400
C	1.13809200	0.45183600	-0.10667900
C	2.48983900	-1.71368100	-0.08154400
C	-4.65039200	-1.81976200	-0.79226500
C	-3.49195000	3.28055100	0.14583600
C	2.39079200	1.24519100	-0.39553400
O	3.34580800	-1.24732800	0.81642200
O	2.68557500	-2.66305300	-0.79049900
H	-5.20742400	-1.34647300	-1.60510500
H	-4.59526100	-2.90501200	-0.94257100
H	-5.12746600	-1.60923600	0.17414200
H	-3.30299900	4.23288200	-0.35613200
H	-3.59475500	3.42807700	1.22838300
H	-4.40707400	2.81476500	-0.24150900
O	2.36329900	2.45412300	0.13210900
O	3.29159400	0.80728400	-1.06311500
C	4.65037300	-1.81994100	0.79258900
C	3.49170600	3.28082400	-0.14578500
H	5.12819200	-1.60786900	-0.17311300
H	4.59505300	-2.90541400	0.94113200
H	5.20678600	-1.34793900	1.60660000
H	3.59425900	3.42911100	-1.22825700
H	4.40683900	2.81458500	0.24098400
H	3.30307100	4.23284900	0.35688200
N	-0.00007000	1.12435600	-0.00003000
H	0.00015200	-2.74362900	-0.00005300

Py-4-3256-R3-Nu1

C	0.83444300	-0.39925200	0.33679200
C	0.44248300	1.07861900	0.17756600
C	-0.42441700	-1.11635100	0.79637500
C	1.89434300	-0.55161500	1.43584600
C	1.52376200	2.06063200	0.26488500
C	-1.65266800	-0.63470900	0.51481300
O	1.48930100	0.03909100	2.56648300
O	2.93131000	-1.15186300	1.35569800
O	1.15655900	3.32982200	0.03068300
O	2.68327500	1.76892100	0.50540500
C	-1.82532900	0.69746700	-0.02498200
C	-2.83632600	-1.50110600	0.81690600
C	2.42411100	0.02512700	3.63167500
C	2.19893100	4.28684100	0.08362800
C	-3.16059800	1.22256700	-0.26382200
O	-3.30239500	-2.07733000	-0.29679200
O	-3.28841900	-1.72120000	1.91031300
H	1.95524900	0.55858600	4.46420200
H	2.66441300	-1.00518300	3.92668200
H	3.35071600	0.53042200	3.32640000
H	1.73803600	5.25751000	-0.12561700
H	2.97258700	4.06581900	-0.66531200
H	2.67159900	4.29870400	1.07538100
O	-3.18174400	2.48383700	-0.73961200
O	-4.19619300	0.60645500	-0.06884700
C	-4.52317900	-2.78442300	-0.15364300
C	-4.46694700	3.03444400	-0.94921800
H	-5.30907600	-2.09712200	0.18913200
H	-4.42104200	-3.60404500	0.56997800
H	-4.77166800	-3.18091100	-1.14302100
H	-5.04908900	3.04587900	-0.01714800
H	-5.02804300	2.45670900	-1.69735800
H	-4.31160100	4.05786200	-1.30664200
N	-0.76831400	1.50787200	-0.07062100
H	-0.30765200	-2.08466900	1.28030600
C	1.39619500	-1.03004400	-1.01432400
C	1.79544400	-2.50927500	-0.84098900
C	2.59624100	-0.23012000	-1.57018600
C	0.26729500	-1.04568000	-2.06887600
O	1.38686600	-3.18469400	0.07265600
C	2.63413400	-3.15078700	-1.92834100
O	3.73315500	-0.59738900	-1.39505000
C	2.32002100	0.96682400	-2.45827000
H	-0.53368700	-1.72829000	-1.75666500
H	-0.17186400	-0.04465600	-2.17560400
H	0.64090800	-1.36843600	-3.05096200
H	2.35531600	-4.20821100	-2.00731900
H	2.53886100	-2.64999400	-2.89977600
H	3.68584200	-3.07387900	-1.62167900
H	2.13706700	0.59319100	-3.47843300
H	1.43675000	1.54391700	-2.16104100
H	3.21321600	1.60153800	-2.47367100

Py-4-3256-R3-Nu3

C	0.72754100	0.46728200	0.26540500
C	-0.29150300	1.57454600	-0.02816300
C	-0.03304000	-0.66132500	0.92117300
C	1.79551000	0.95708200	1.24586200
C	0.20263200	2.95900000	-0.09009000
C	-1.34292300	-0.85629900	0.65137700
O	1.23527400	1.39911100	2.37221500
O	2.98615700	0.90827500	1.08095300
O	-0.70309500	3.85004800	-0.51996800
O	1.32605300	3.29920800	0.23151200
C	-2.11454800	0.15783900	-0.02791600
C	-1.95993900	-2.15258100	1.07489200
C	2.11647500	2.02061300	3.29364900
C	-0.25875900	5.19330400	-0.57670100
C	-3.54325700	-0.03820500	-0.23052100
O	-2.08905300	-2.96850800	0.02015700
O	-2.23141300	-2.48081100	2.19944600
H	1.50902300	2.31164300	4.15590900
H	2.91213600	1.32997200	3.60213200
H	2.56831800	2.90733900	2.82761900
H	-1.10867200	5.78497800	-0.93166000
H	0.58901700	5.29687700	-1.26813300
H	0.05864000	5.54430400	0.41497900
O	-4.15680200	0.98464300	-0.85455300
O	-4.16320900	-1.03143900	0.11401600
C	-2.79618800	-4.17466100	0.26141300
C	-5.55005700	0.83580500	-1.04716700
H	-3.81858800	-3.94280200	0.58985700
H	-2.29673900	-4.77361500	1.03418600
H	-2.81673300	-4.71867500	-0.68779700
H	-6.06970500	0.70517300	-0.08766400
H	-5.77025500	-0.03600000	-1.67934200
H	-5.89445000	1.75133600	-1.53929800
N	-1.56280600	1.35523700	-0.23727600
H	0.52138600	-1.37809300	1.53001800
C	1.43614100	-0.06322300	-1.07544800
C	2.28299800	-1.32293100	-0.84000800
C	2.28433600	1.00160500	-1.64793400
C	0.36479600	-0.34679700	-2.15191000
C	1.65750500	-2.57471100	-0.73659800
C	3.67940300	-1.27188100	-0.74894900
N	2.90560400	1.80871300	-2.19097700
H	-0.16143600	0.57749000	-2.42117100
H	0.84464900	-0.76216600	-3.04824500
H	-0.37349300	-1.06506800	-1.77703900
C	2.40651600	-3.73331900	-0.52790300
H	0.57296000	-2.65665300	-0.80796700
C	4.42851300	-2.43097200	-0.55255300
H	4.18421500	-0.30909600	-0.82101100
C	3.79558500	-3.66801400	-0.43610500

H	1.89517400	-4.69379100	-0.44439200
H	5.51575300	-2.36278000	-0.48846800
H	4.38149900	-4.57520600	-0.27980700

Py-4-3256-R3-Nu2

C	1.05331800	-0.11366900	0.68918400
C	1.72858700	1.04581800	-0.05114800
C	-0.02186700	0.53659800	1.53140800
C	1.99916600	-0.93309800	1.55439200
C	3.17144700	0.97835600	-0.31497100
C	-0.74758200	1.53344900	0.96961900
O	2.49655100	-0.20511000	2.54985000
O	2.22624100	-2.10344100	1.39845400
O	3.62778900	1.97928200	-1.08199300
O	3.91331300	0.11515700	0.11424200
C	-0.27911200	2.14260100	-0.25448700
C	-1.81505200	2.19095100	1.78300900
C	3.50613200	-0.83668800	3.32232000
C	5.01230900	1.93994500	-1.37660100
C	-1.16078300	3.01665000	-1.02513400
O	-2.64274400	1.29212200	2.33949600
O	-1.91456100	3.37243800	1.98487600
H	3.80724000	-0.11615600	4.08856400
H	3.12313900	-1.75398500	3.78776500
H	4.35778300	-1.08678800	2.67515500
H	5.21857400	2.80956800	-2.00860400
H	5.27378800	1.01472100	-1.90863000
H	5.61015900	1.99066200	-0.45627300
O	-0.52648500	3.75468600	-1.95452600
O	-2.36798600	3.06605500	-0.88717500
C	-3.64430500	1.82012700	3.19792800
C	-1.35742000	4.57419000	-2.75412300
H	-4.29345500	2.51401200	2.64785400
H	-3.18597700	2.35682400	4.03906700
H	-4.22102300	0.96438700	3.56150500
H	-1.89220500	5.31199800	-2.13959500
H	-2.10113500	3.97259000	-3.29529200
H	-0.69883600	5.08494900	-3.46422300
N	1.00277000	1.98686800	-0.59798200
H	-0.21812100	0.16899900	2.53674800
C	0.36959000	-1.02577900	-0.36025600
Br	1.52343800	-1.66348500	-1.77401900
S	-0.56394700	-2.44994300	0.38491300
H	-0.39552200	-0.42408500	-0.86713600
O	-0.02885100	-3.72439800	-0.07741500
O	-0.70339600	-2.20345900	1.82023000
C	-2.17081100	-2.23239000	-0.36133300
C	-2.51634800	-3.02141500	-1.45479700
C	-3.02053600	-1.25383700	0.15423900
C	-3.76118500	-2.82160300	-2.05215400
H	-1.81858500	-3.77613300	-1.81954100
C	-4.25713800	-1.06246200	-0.45833000

H	-2.71519700	-0.64721100	1.01054900
C	-4.62524600	-1.84459800	-1.55601300
H	-4.05525800	-3.43019500	-2.90808100
H	-4.93453100	-0.29759300	-0.07639400
H	-5.59533400	-1.68911600	-2.03036700

Py-4-3256-R3-Nu4

C	-0.81466400	-0.84516200	0.34834500
C	-1.00216900	-0.60261500	1.88063000
C	-0.79123700	0.48628200	-0.39787800
C	-2.11960300	0.33597500	2.27682900
H	-1.12675200	-1.58435900	2.36319300
H	-0.04987900	-0.17199800	2.22315600
C	0.53306400	-1.49393700	0.10505200
C	-1.87684500	-1.79724600	-0.18076500
C	-2.08313000	1.03856400	-0.80834100
C	-3.50413600	-0.22615400	2.44879200
C	-1.86464900	1.64039800	2.43898100
C	1.63131500	-0.71026300	0.00658000
O	-1.78812800	-1.94508100	-1.50562300
O	-2.65235400	-2.42858900	0.49257200
O	-2.02916800	2.29032400	-1.29121400
O	-3.14029100	0.43532900	-0.72614600
C	1.47681700	0.72549900	-0.11269800
H	-3.89525800	-0.55159200	1.47502000
H	-3.49075300	-1.11143000	3.10293300
H	-4.18350900	0.52458600	2.87594400
H	-0.85864300	2.04067800	2.28414300
H	-2.65479600	2.34501800	2.71272600
C	2.94399500	-1.36129000	-0.28818300
C	-2.80189700	-2.73066100	-2.10767100
C	-3.27429400	2.86388300	-1.63986600
C	2.62033600	1.60609900	0.06279600
O	3.19958100	-2.38107000	0.54474800
O	3.67592200	-1.07477800	-1.20018400
H	-2.57438500	-2.76694200	-3.17751600
H	-2.81639400	-3.74439800	-1.68590800
H	-3.78171000	-2.26052100	-1.94404100
H	-3.05921700	3.87210400	-2.00870200
H	-3.93899800	2.91611800	-0.76570100
H	-3.77613300	2.27522900	-2.42046600
O	2.39313700	2.88449600	-0.31272100
O	3.69175900	1.27184200	0.53794100
C	4.38812900	-3.10838900	0.27580700
C	3.46357600	3.78070200	-0.09853200
H	5.26435200	-2.44932400	0.33654500
H	4.35322700	-3.55124500	-0.72883200
H	4.44991900	-3.89386300	1.03521400
H	4.35064800	3.48602900	-0.67723800
H	3.74504600	3.81698100	0.96365900
H	3.11523800	4.76599500	-0.42667800
N	0.28121900	1.22775800	-0.44910500

H	0.60814900	-2.58125000	0.03201900

Py-4-3256-R4

C	0.00000000	1.18796500	-0.69651000
C	0.00000000	0.00000000	-1.41276500
C	0.00000000	1.12860800	0.69745700
C	0.00000000	-1.18796500	-0.69651000
C	0.00000000	-1.12860800	0.69745700
F	0.00000000	2.25375000	1.38794200
F	0.00000000	-2.25375000	1.38794200
F	0.00000000	-2.36123100	-1.30707600
N	0.00000000	0.00000000	1.35895000
H	0.00000000	0.00000000	-2.50303200
F	0.00000000	2.36123100	-1.30707600

Py-4-3256-R4-Nu1

C	-0.09861600	0.05486900	-0.20152700
C	-0.75889800	-1.06933400	0.28793600
C	-0.93330600	1.05487900	-0.72711600
C	-2.14546200	-1.13490600	0.27006900
C	-2.83950300	-0.05942400	-0.25905400
F	-0.41748400	2.09459300	-1.36106300
F	-4.16764300	-0.08848400	-0.28910300
F	-2.80079800	-2.19630300	0.74151700
F	-0.30033300	2.37329900	1.30605300
N	-2.24195100	0.99003800	-0.76244200
H	-0.20099400	-1.91111400	0.70175400
C	1.42130500	0.13193000	-0.13205200
C	2.04847500	-0.84407300	-1.14459000
C	1.79222800	-0.38906000	1.27926500
C	1.98664900	1.55550800	-0.29909000
O	1.38324400	-1.60871500	-1.79888900
C	3.55039900	-0.77941300	-1.29559400
O	2.16627600	-1.53424800	1.41997400
C	1.60995200	0.57434900	2.40971400
H	1.95434500	1.86946600	-1.35033500
H	1.34170700	2.21674100	0.30511300
H	3.03134900	1.58738000	0.04254100
H	3.89633400	-1.67365100	-1.82608100
H	3.81512600	0.11277500	-1.88217800
H	4.04515800	-0.69777800	-0.31912500
H	2.50550700	1.21664600	2.45139900
H	0.74967000	1.25496800	2.18855100
H	1.53626900	0.01809500	3.35239200

Py-4-3256-R4-Nu3

C	0.54882500	0.38826300	-0.27161300
C	0.79204400	-1.03440800	-0.12138200
C	1.79682200	1.10286800	0.00331900
C	2.08417500	-1.53389800	-0.12080000

C	3.17706000	-0.68569900	-0.06811500
F	1.67782100	2.44589300	0.17733500
F	4.44300200	-1.15950100	-0.03685600
F	2.28154200	-2.86266800	-0.11211900
F	0.13953100	0.72083600	-1.70349600
N	2.99135600	0.61888400	0.03036500
H	-0.03877000	-1.73976200	-0.11681400
C	-0.71501700	0.93961300	0.49484500
C	-1.91395100	0.01459300	0.25128500
C	-1.07288200	2.27232000	-0.03353300
C	-0.38379800	1.10368000	1.98480000
C	-2.36466500	-0.86455800	1.24030800
C	-2.55462400	0.00762800	-0.99610900
N	-1.39749100	3.32009600	-0.39249500
H	0.39838400	1.86295100	2.10575300
H	-1.27286800	1.41122800	2.55178100
H	-0.00543000	0.15379500	2.38383100
C	-3.42880000	-1.73470900	0.98917600
H	-1.89017300	-0.88424900	2.22089700
C	-3.61755200	-0.85602700	-1.24432000
H	-2.19482300	0.67743700	-1.77697200
C	-4.05934900	-1.73387800	-0.25189900
H	-3.76393600	-2.41395600	1.77472400
H	-4.10402900	-0.84471000	-2.22108100
H	-4.89219700	-2.41136100	-0.44676000

Py-4-3256-R4-Nu2

C	1.49159300	0.26719300	-0.27451500
C	2.11647800	-1.05105700	-0.33760000
C	2.35769600	1.12286900	0.55647100
C	3.47370000	-1.18818400	-0.10605300
C	4.21935000	-0.15201000	0.43008800
F	1.77641700	2.24559900	1.05214400
F	5.53756100	-0.28932100	0.69965300
F	4.06616700	-2.37088000	-0.34003900
F	1.38474900	0.85285700	-1.63072400
N	3.61434800	0.96459500	0.79403100
H	1.57407600	-1.91537000	-0.71403600
C	0.01670500	0.30445600	0.22804100
Br	-0.95508300	1.89376800	-0.33284900
S	-0.94044800	-1.18116900	-0.27972400
H	-0.01318200	0.29008900	1.32341900
O	-0.83199300	-1.33425100	-1.72683700
O	-0.51877300	-2.26513800	0.60746800
C	-2.64072000	-0.82427800	0.11710800
C	-3.51650800	-0.48057200	-0.90934800
C	-3.05538800	-0.94356500	1.44202700
C	-4.85028800	-0.23390500	-0.59069600
H	-3.14744700	-0.41136800	-1.93324000
C	-4.38972700	-0.68273300	1.74786000
H	-2.34660400	-1.24507300	2.21487500
C	-5.28182600	-0.32872000	0.73387200

H	-5.55479700	0.03455000	-1.37860200
H	-4.73545700	-0.76481700	2.77882100
H	-6.32627100	-0.12951300	0.97799400

Py-4-3256-R4-Nu4

C	-0.39690600	-0.15568500	0.83852400
C	-1.92543600	-0.02088200	0.81747200
C	0.35220100	1.06285000	0.58340900
C	-2.50784300	0.53128900	-0.45832400
H	-2.34203100	-1.01968600	1.02127100
H	-2.19312300	0.62833500	1.66206800
C	0.17897200	-1.22398300	0.01968800
C	1.64259000	0.99053800	0.08686200
C	-2.32178200	-0.28282600	-1.70990700
C	-3.16801400	1.69481600	-0.46656100
C	2.14712700	-0.20435300	-0.40222200
H	-2.63002300	-1.32609000	-1.54626800
H	-2.89274400	0.13668500	-2.54881900
H	-1.25724400	-0.31192300	-1.99135200
H	-3.29208900	2.28106900	0.44757500
H	-3.60332600	2.09505300	-1.38596100
F	-0.60575800	-2.32123600	-0.16277300
F	3.39750600	-0.28453700	-0.92014300
F	2.39418400	2.10443700	-0.00274500
F	-0.22237300	-0.60406800	2.30091400
N	1.37055000	-1.27427700	-0.47269800
H	-0.06075100	2.03453500	0.85945700

Py-4-3256-R5

C	0.00000000	1.18462200	-0.72696100
C	0.00000000	0.00000000	-1.44658000
C	-0.02906600	1.12470800	0.66441900
C	0.00000000	-1.18462200	-0.72696100
C	0.02906600	-1.12470800	0.66441900
N	0.18152500	-2.34900600	1.49842400
O	-0.54925000	-2.44825000	2.44091000
O	1.05845500	-3.09914700	1.15993000
N	-0.09670200	-2.45898000	-1.46128200
O	0.41628200	-2.49127200	-2.54733300
O	-0.71174700	-3.34024100	-0.92182000
N	0.09670200	2.45898000	-1.46128200
O	-0.41628200	2.49127200	-2.54733300
O	0.71174700	3.34024100	-0.92182000
N	-0.18152500	2.34900600	1.49842400
O	-1.05845500	3.09914700	1.15993000
O	0.54925000	2.44825000	2.44091000
N	0.00000000	0.00000000	1.33443700
H	0.00000000	0.00000000	-2.53804800

Py-4-3256-R5-Nu1

C	0.78850500	0.18578200	0.45432400
C	-0.27903800	-0.75116200	0.95724700
C	0.08009000	1.49179900	0.13329700
C	-1.56267000	-0.58860400	0.59118700
C	-1.99170800	0.59190900	-0.09928800
N	-3.29210000	0.69512800	-0.69735300
O	-3.71868100	1.78677600	-1.00142900
O	-3.88614600	-0.35295400	-0.89353900
N	-2.52952700	-1.54455900	1.15308400
O	-3.49675800	-1.08917500	1.70759000
O	-2.26185100	-2.71948400	1.06144500
N	1.74610600	0.39794400	1.67795000
O	1.21795900	0.77398300	2.68894600
O	2.91883200	0.16168600	1.54895000
N	0.85503400	2.71197600	0.07545000
O	1.97544900	2.67646200	0.56398000
O	0.36993800	3.70284800	-0.41871700
N	-1.16497900	1.59815900	-0.21097600
H	0.01591200	-1.59309400	1.57994300
C	1.62869700	-0.41989000	-0.73585700
C	0.55065500	-0.91052900	-1.76642600
C	2.44202300	-1.63077600	-0.23438200
C	2.48197200	0.62531700	-1.46158300
O	0.02472000	-0.08696900	-2.47029600
C	0.20046300	-2.37219400	-1.85370600
O	1.95461500	-2.41657700	0.54484200
C	3.82507800	-1.83839400	-0.79015400
H	1.83863700	1.42624900	-1.84498700
H	3.24169800	1.05112800	-0.79853700
H	2.96854200	0.16836400	-2.33349900
H	-0.58773700	-2.49678800	-2.60388800
H	1.08902800	-2.95430700	-2.14165600
H	-0.13203800	-2.75094400	-0.87776300
H	3.79124300	-1.92231100	-1.88635800
H	4.46046500	-0.97737700	-0.54370000
H	4.24350700	-2.75377700	-0.35877800

Py-4-3256-R5-Nu3

C	0.24772700	0.79328200	0.33348400
C	-0.18433700	-0.59111500	0.71295300
C	-0.97028100	1.53117800	-0.14744800
C	-1.41083700	-1.06359200	0.41808800
C	-2.41194100	-0.22959000	-0.17155600
N	-3.77482200	-0.66096400	-0.32969700
O	-4.54014200	0.03546400	-0.95625800
O	-4.08195100	-1.71737500	0.19800700
N	-1.60154900	-2.51339600	0.60203800
O	-1.49697000	-2.95637500	1.71676800
O	-1.78351700	-3.15446700	-0.40165400
N	0.73135300	1.43358400	1.68104800
O	-0.04392400	2.13329500	2.27125000
O	1.82009500	1.09860000	2.07453700

N	-0.82863600	2.95575700	-0.37410300
O	0.30257100	3.40570200	-0.26165600
O	-1.79673500	3.61788200	-0.65612300
N	-2.14214100	1.03515800	-0.37908700
H	0.55296200	-1.24378600	1.17849100
C	1.49837500	0.73039000	-0.69187100
C	2.24029800	-0.61355100	-0.58236600
C	2.48251400	1.80778600	-0.47530600
C	0.93828500	0.91724900	-2.11812600
C	1.75423900	-1.73520400	-1.26811300
C	3.37372700	-0.76032200	0.22405400
N	3.30594900	2.60956200	-0.38010300
H	0.61624500	1.95304400	-2.27085900
H	1.71953200	0.67789500	-2.85059000
H	0.07857000	0.25596500	-2.28686900
C	2.37828000	-2.97493500	-1.13836700
H	0.86794600	-1.66014300	-1.89838800
C	4.00309100	-1.99893700	0.34628100
H	3.76560000	0.09976700	0.76652900
C	3.50602900	-3.11174900	-0.33017200
H	1.97697200	-3.83607900	-1.67434900
H	4.88815900	-2.09070500	0.97737800
H	3.99650200	-4.08103100	-0.23021800

Py-4-3256-R5-Nu2

C	-0.58115900	0.21540200	0.58425200
C	-1.45893400	-1.00336800	0.72617900
C	-1.50848600	1.39166400	0.34663100
C	-2.69705700	-1.02602300	0.19828200
C	-3.31064200	0.16047500	-0.31939600
N	-4.69265400	0.20580900	-0.72276800
O	-5.10602000	1.20395000	-1.26664900
O	-5.37307400	-0.77666200	-0.48127500
N	-3.34997600	-2.34636500	0.12593500
O	-3.57892600	-2.91346600	1.16342300
O	-3.54858500	-2.78112000	-0.97878700
N	0.18657500	0.37420800	1.92078800
O	-0.48633800	0.40708900	2.91312400
O	1.38585900	0.43587700	1.87229400
N	-1.04350200	2.73711400	0.64746300
O	-0.02516300	2.83087100	1.31095400
O	-1.68769200	3.67994300	0.25732100
N	-2.70292700	1.30727000	-0.14103100
H	-1.01690800	-1.90433100	1.14776600
C	0.42935800	-0.01117000	-0.57170100
Br	1.35168600	1.55045400	-1.22622700
S	1.58054000	-1.47357000	-0.38058200
H	-0.16412500	-0.35935800	-1.42603400
O	1.18010600	-2.20755400	0.81994300
O	1.50290300	-2.12022900	-1.68787600
C	3.25947600	-0.92789500	-0.16496200
C	3.81281700	-0.92209600	1.11277400

C	4.01228100	-0.64954600	-1.30595800
C	5.16090100	-0.59546000	1.24962300
H	3.19380700	-1.16920900	1.97415700
C	5.35558300	-0.31901200	-1.15182900
H	3.54911500	-0.69874600	-2.29188300
C	5.92543600	-0.29008900	0.12305900
H	5.61518500	-0.58455500	2.24074000
H	5.96094300	-0.09137600	-2.02962300
H	6.97972300	-0.03432400	0.23790500

Py-4-3256-R5-Nu4

C	-0.99313400	0.38625900	0.56118500
C	-2.20611300	1.25278600	0.13861300
C	0.25895000	1.20071300	0.44737300
C	-2.20526200	1.59740900	-1.33212900
H	-3.13305500	0.73561400	0.41010600
H	-2.13898800	2.17243600	0.73374600
C	-0.74736900	-0.94252600	-0.08803600
C	1.44154400	0.62661700	0.15234100
C	-2.59591200	0.51523300	-2.30296800
C	-1.88398600	2.83077400	-1.73708000
C	1.51618700	-0.76038400	-0.19307500
H	-3.53094500	0.02829200	-1.98771600
H	-2.72604800	0.92261800	-3.31369600
H	-1.82910900	-0.27428800	-2.35937900
H	-1.61251700	3.61131800	-1.02195300
H	-1.89008500	3.09777000	-2.79656900
N	2.77582200	-1.44610300	-0.30774500
O	2.81491200	-2.51707700	-0.87078900
O	3.74183000	-0.90545200	0.20506300
N	2.58122600	1.53305700	-0.07272500
O	2.80369600	2.36728800	0.77017000
O	3.17591300	1.41300800	-1.11396700
N	-1.22537400	0.12217500	2.11859400
O	-1.21969200	-1.01461200	2.50581200
O	-1.36091700	1.09963200	2.81217900
N	-1.87245300	-1.83400600	-0.30498300
O	-2.94867700	-1.50041500	0.16461500
O	-1.70399300	-2.85120000	-0.93715900
N	0.41694300	-1.45287100	-0.34969200
H	0.19358800	2.27981400	0.58658500

Py-5-23456-R1

C	0.96456400	-0.03902100	0.00000000
C	0.25864700	0.03686100	1.21094000
C	0.25864700	0.03686100	-1.21094000
C	-1.14122200	-0.01937500	1.14367700
C	-1.14122200	-0.01937500	-1.14367700
C	2.46919200	-0.36924100	0.00000000
C	0.96800900	0.27601200	2.54793300
C	0.96800900	0.27601200	-2.54793300

C	-2.06455500	-0.08669400	2.36964300
C	-2.06455500	-0.08669400	-2.36964300
F	1.21938200	-0.84140200	-3.20788000
F	2.11152000	0.92607800	-2.35763300
F	0.21347100	1.05588000	-3.31596500
F	-2.34194200	1.12381400	-2.83619400
F	-1.50864300	-0.80361800	-3.34297100
F	-3.20534200	-0.66822100	-2.04291400
F	-3.20534200	-0.66822100	2.04291400
F	-1.50864300	-0.80361800	3.34297100
F	-2.34194200	1.12381400	2.83619400
F	0.21347100	1.05588000	3.31596500
F	1.21938200	-0.84140200	3.20788000
F	2.11152000	0.92607800	2.35763300
F	2.75956900	-1.11083200	1.06443300
F	2.75956900	-1.11083200	-1.06443300
F	3.24886700	0.69563000	0.00000000
N	-1.79388400	-0.08047500	0.00000000

Py-5-23456-R1-Nu1

C	-1.02436600	-0.34968100	-0.42649000
C	-0.31989900	1.03308000	-0.40103100
C	1.15923200	-1.27064300	-0.51577500
C	1.02976400	1.07822200	-0.13956200
C	1.81287400	-0.10691500	-0.03121000
C	1.94402900	-2.54310600	-0.83769900
C	3.06551500	-0.19867900	0.76520100
C	1.77020200	2.41068000	-0.02495000
C	-1.13006300	2.29156000	-0.60057200
C	-2.10440900	-0.39152100	-1.58786100
F	-1.86531100	0.59683900	-2.46927500
F	-2.06901000	-1.49908000	-2.31049400
F	-3.35463400	-0.22970700	-1.16013500
F	-2.44057700	2.04079300	-0.42792300
F	-1.01400700	2.87514000	-1.79693100
F	-0.83371400	3.24841300	0.29861500
F	3.07754700	2.26559500	-0.24624300
F	1.35531300	3.30607000	-0.92245100
F	1.63425900	2.95802700	1.18510100
F	4.22583400	-0.04483400	0.10617600
F	3.15709500	-1.39980100	1.37215400
F	3.08495900	0.71054800	1.76056000
F	1.85625700	-3.42932800	0.16124100
F	1.47469600	-3.13998100	-1.92832800
F	3.24085700	-2.30743800	-1.05212200
C	-1.64643800	-0.75026500	0.99986700
C	-2.41577500	-2.09076500	0.95220600
C	-2.65644500	0.27083700	1.58034000
C	-0.50347500	-0.91013200	2.02581200
O	-2.72128000	-2.63487300	-0.07486000
C	-2.75425000	-2.72879800	2.28560000
O	-3.84311300	0.05778200	1.51328800

C	-2.13219300	1.44941600	2.36785200
H	0.11294200	-1.78681300	1.78836700
H	0.14493700	-0.02729000	2.04124000
H	-0.90952700	-1.04604800	3.03670500
H	-1.87112200	-3.26887500	2.65828400
H	-3.04863800	-1.99239100	3.04351900
H	-3.56365600	-3.44954000	2.12488600
H	-2.07578000	1.13963400	3.42321800
H	-1.13374500	1.77546900	2.06130800
H	-2.84923700	2.27447000	2.28805200
N	-0.08898800	-1.38899200	-0.79612200

Py-5-23456-R1-Nu3

C	-1.34856400	-0.65328300	-0.23689000
C	-1.15258600	0.86810000	-0.19553000
C	0.85928100	-0.76567100	-1.08686800
C	0.13350700	1.36042500	-0.30276700
C	1.23718900	0.51789400	-0.61289300
C	1.83391800	-1.65701500	-1.86348100
C	2.66767500	0.85960200	-0.35850600
C	0.41979800	2.85471400	-0.14080800
C	-2.33889800	1.77621700	0.00332200
C	-2.66607400	-1.07371000	-1.00363900
F	-2.99895400	-0.13634200	-1.89715300
F	-2.50750100	-2.20526900	-1.67939600
F	-3.73113900	-1.27926800	-0.22089700
F	-3.43623400	1.09522300	0.36239200
F	-2.69971800	2.47980500	-1.07661600
F	-2.13695800	2.66442600	0.99653600
F	1.53876300	3.21534300	-0.76989100
F	-0.53211800	3.63184900	-0.65684000
F	0.55619000	3.18904800	1.14433300
F	3.32318700	1.46627400	-1.36532400
F	3.38384000	-0.24607100	-0.08788000
F	2.81527700	1.66231300	0.71035000
F	2.44096000	-2.54273600	-1.06811200
F	1.20132500	-2.35343500	-2.80300500
F	2.78512100	-0.95276900	-2.48310100
C	-1.34381600	-1.31680000	1.24223600
C	0.06796200	-1.22401200	1.83855100
C	-1.69595400	-2.74261000	1.09484100
C	-2.35458300	-0.69663900	2.22985500
C	0.45903700	-0.08099900	2.54730900
C	0.99367300	-2.25943400	1.67382700
N	-1.99958500	-3.85276400	1.01231500
H	-3.38793900	-0.87998500	1.92614100
H	-2.18296100	-1.13277800	3.22276500
H	-2.20411500	0.38608600	2.30366700
C	1.74692500	0.02400500	3.06749200
H	-0.22837000	0.75533200	2.67086300
C	2.28174400	-2.15404500	2.19460300
H	0.71268500	-3.15227800	1.11367000

C	2.66317800	-1.01226000	2.89524200
H	2.03765500	0.93138800	3.59883000
H	2.99181600	-2.96762300	2.03898700
H	3.67380800	-0.92485400	3.29710200
N	-0.31632900	-1.28404800	-1.01745500

Py-5-23456-R1-Nu2

C	-0.08190600	0.51563900	0.99778800
C	1.04222900	1.34978200	0.37424700
C	2.11778000	0.69691500	-0.17796900
C	1.35504900	-1.35158500	0.78241200
C	2.25934400	-0.71768400	-0.11051200
C	3.24120600	1.49967800	-0.83349800
C	0.78752600	2.82123600	0.17341600
C	3.16021900	-1.50220700	-1.00218500
C	1.56767700	-2.79751500	1.24643000
C	-0.59943800	1.19312100	2.34353200
F	0.25376200	2.14606800	2.73846900
F	-1.79508200	1.78189400	2.21751300
F	-0.68547500	0.33785600	3.34448100
F	2.86287300	-3.13300600	1.25284900
F	1.12178300	-2.97578500	2.48517000
F	0.92740800	-3.67084300	0.47089000
F	4.39581000	-1.75045400	-0.53358800
F	3.33438300	-0.90048400	-2.19248700
F	2.63258400	-2.70707500	-1.28000000
F	3.02182600	1.72986500	-2.12661000
F	3.42089400	2.68326900	-0.24393800
F	4.41199800	0.86765000	-0.73083500
F	1.43282800	3.65501800	0.99388500
F	1.07326200	3.22363700	-1.07362100
F	-0.52486900	3.08408200	0.34506600
C	-1.31642900	0.38117300	0.02688000
Br	-0.81426900	0.13012500	-1.82085500
S	-2.42258700	-1.01155800	0.50461800
H	-1.94843000	1.27379700	0.05256400
O	-1.92146700	-2.27121200	-0.02693600
O	-2.69508400	-0.85487500	1.92923700
C	-3.92094400	-0.59846700	-0.36990800
C	-4.22381500	-1.25895100	-1.55749100
C	-4.76150200	0.36818900	0.18149300
C	-5.40538500	-0.92754500	-2.21963100
H	-3.54212400	-2.01844800	-1.94234100
C	-5.93570700	0.69280900	-0.49380700
H	-4.49706600	0.84637000	1.12666500
C	-6.25386200	0.04618500	-1.69048700
H	-5.66438800	-1.43255800	-3.15078000
H	-6.60783800	1.44627500	-0.08193400
H	-7.17612700	0.30205900	-2.21401500
N	0.35444100	-0.79830100	1.37400300

Py-5-23456-R1-Nu4

C	1.10320400	-1.13472200	0.14819100
C	1.23073700	-1.47075700	1.66864800
C	1.19615300	0.36948100	-0.15827200
C	0.27878500	-0.70533200	2.55921100
H	1.04870000	-2.55234400	1.76018600
H	2.26690400	-1.27197100	1.97101400
C	0.02370300	1.06733500	-0.29059100
C	-1.00965700	-1.38970000	2.93009300
C	0.59453200	0.51374800	3.01065500
C	-1.17510600	-1.00019500	-0.43271200
C	-1.24284600	0.40578200	-0.24703000
H	-0.80098400	-2.26095800	3.57258900
H	-1.68325800	-0.71154900	3.47091800
H	-1.53254700	-1.77012200	2.04227300
H	1.54821800	0.98250500	2.75597600
H	-0.09938700	1.08233700	3.63489000
C	0.00386300	2.55535100	-0.64520400
C	2.55478100	1.00767100	-0.10270600
C	-2.48967900	1.06610400	0.21588500
C	-2.41180600	-1.80259100	-0.84006900
C	2.19136600	-1.95616900	-0.58540800
F	2.16054000	-1.72980200	-1.89862300
F	3.43884600	-1.70732300	-0.17215800
F	2.00174400	-3.26464400	-0.40030700
F	-3.36173300	-1.04315900	-1.38960700
F	-2.10648700	-2.74431000	-1.72879700
F	-2.96122500	-2.42649000	0.21024700
F	-3.31931100	1.56306500	-0.72033200
F	-2.22482100	2.09489500	1.04581700
F	-3.24736300	0.20317400	0.92691600
F	0.25141200	3.35242700	0.39360600
F	0.88103800	2.84222900	-1.61321800
F	-1.18328600	2.92423400	-1.13342000
F	3.30063400	0.78831000	-1.19799700
F	2.54027200	2.32881900	0.08731200
F	3.26915100	0.52896100	0.93690200
N	-0.11293300	-1.72106200	-0.38700800

Py-5-23456-R2

C	0.00000000	0.00000000	1.02001000
C	0.00000000	1.20670400	0.30565300
C	0.00000000	-1.20670400	0.30565300
C	0.00000000	1.14017600	-1.10217300
C	0.00000000	-1.14017600	-1.10217300
N	0.00000000	0.00000000	-1.77552500
C	0.00000000	-2.47159000	0.98549000
N	0.00000000	-3.48879000	1.52843700
C	0.00000000	-2.36285900	-1.88012600
N	0.00000000	-3.34205900	-2.48756600
C	0.00000000	0.00000000	2.45862000
N	0.00000000	0.00000000	3.61122500

C	0.00000000	2.47159000	0.98549000
N	0.00000000	3.48879000	1.52843700
C	0.00000000	2.36285900	-1.88012600
N	0.00000000	3.34205900	-2.48756600

Py-5-23456-R2-Nu1

C	0.68173900	0.38145200	-0.74993500
C	-0.43213000	1.39365100	-0.45381200
C	-1.65828800	0.90033400	-0.06447800
C	-1.00992100	-1.23212200	-0.94326600
C	-1.94548600	-0.48421500	-0.14994000
C	-2.68407700	1.80890300	0.38759400
N	-3.50063200	2.53068400	0.76693100
C	-0.19199200	2.79182700	-0.54378200
N	0.02553100	3.92890400	-0.60203000
C	-3.16655800	-1.04011600	0.30782400
N	-4.15786800	-1.50036600	0.69535200
C	-1.43886900	-2.53864000	-1.43764500
N	-1.77857000	-3.57387900	-1.81541800
C	1.59122600	1.00042900	-1.75368500
N	2.23481800	1.55806800	-2.53010000
C	1.54551800	0.05451600	0.54727300
C	2.72050000	-0.88071600	0.18053700
C	0.67672800	-0.72238100	1.55844800
C	2.12769900	1.34262800	1.14512300
O	2.97661000	-1.17054300	-0.95803300
C	3.60763400	-1.33782500	1.31905300
O	0.62059700	-1.92628600	1.46516600
C	-0.01079200	0.02104700	2.67391700
H	2.77550200	1.84805600	0.41400000
H	1.33712700	2.04309900	1.44260000
H	2.72834500	1.11926000	2.03720000
H	4.48464400	-0.67336300	1.34888000
H	3.11608700	-1.31188100	2.29826000
H	3.96056900	-2.35308400	1.10265200
H	0.74383200	0.26989800	3.43736600
H	-0.45579600	0.96646800	2.34050900
H	-0.76880900	-0.63059700	3.12255100
N	0.15147100	-0.83710400	-1.33479600

Py-5-23456-R2-Nu3

C	-0.06158900	-0.62763400	-0.52634800
C	1.31530400	-1.25858000	-0.32006700
C	2.38624800	-0.43855700	-0.03480300
C	1.06053300	1.44218000	-0.69833900
C	2.27938500	0.97096200	-0.11646400
C	3.66580500	-1.02927300	0.27731200
N	4.68939300	-1.49616300	0.53297300
C	1.47445000	-2.66807300	-0.42889700
N	1.57770500	-3.81884800	-0.51857300
C	3.35435300	1.84074000	0.19517600

N	4.22820600	2.55665000	0.45623200
C	0.99661400	2.85056500	-1.08554200
N	0.93964200	3.96416400	-1.37943200
C	-0.79352000	-1.39882100	-1.56883900
N	-1.33489000	-2.00599000	-2.38521800
C	-0.96796300	-0.69379800	0.79920300
C	-2.34154900	-0.07528100	0.52561400
C	-0.27595000	0.10260000	1.83114200
C	-1.05185700	-2.14014400	1.31725800
C	-3.41885100	-0.88394700	0.14773300
C	-2.53268400	1.30941000	0.61096900
N	0.25307400	0.70139000	2.66296800
H	-0.07789500	-2.47632200	1.69305500
H	-1.78096400	-2.19601400	2.13543800
H	-1.36566900	-2.82239500	0.51562900
C	-4.66065900	-0.31852900	-0.14226000
H	-3.29938800	-1.96446900	0.06948600
C	-3.77384800	1.87287200	0.32463200
H	-1.70416800	1.95426200	0.90880600
C	-4.84238800	1.06016200	-0.05422900
H	-5.48933400	-0.96435200	-0.43561000
H	-3.90462700	2.95317500	0.39946700
H	-5.81504300	1.50099200	-0.27733200
N	0.01345300	0.74365400	-0.98729700

Py-5-23456-R2-Nu2

C	0.81525000	-0.10475100	-0.17964300
C	1.70665100	1.11967700	-0.33542800
C	2.96759300	0.98801600	0.20754500
C	2.74887500	-1.36742200	-0.10182300
C	3.44687300	-0.30519100	0.55826500
C	3.85016200	2.12401300	0.29145000
N	4.55948300	3.03111100	0.36849700
C	1.31668500	2.27227700	-1.07040900
N	1.01157700	3.19125700	-1.70595600
C	4.70740500	-0.50592100	1.18328800
N	5.72872700	-0.67445000	1.70333800
C	3.46919200	-2.60900900	-0.35400900
N	4.03907700	-3.59403600	-0.54180800
C	0.46125700	-0.30858900	1.26588100
N	0.21649200	-0.53106600	2.37051500
C	-0.48399100	0.00992900	-1.01001200
Br	-1.36402200	-1.68264000	-1.24355700
S	-1.67038500	1.30779700	-0.37682600
H	-0.24584400	0.38409700	-2.01390400
O	-0.94890600	2.09177600	0.62316400
O	-2.18985000	1.93016900	-1.58843300
C	-3.02334700	0.49999700	0.44566700
C	-2.96095600	0.30818000	1.82395400
C	-4.14689700	0.15865900	-0.30726600
C	-4.05566200	-0.27584000	2.46054800
H	-2.07562000	0.61231900	2.38237500

C	-5.23159400	-0.42059800	0.34409700
H	-4.16207200	0.34962800	-1.38078600
C	-5.18201500	-0.64083400	1.72295300
H	-4.02862100	-0.43814500	3.53834900
H	-6.11977900	-0.69748700	-0.22452100
H	-6.03518900	-1.09558300	2.22832200
N	1.56053600	-1.27685700	-0.61234600

Py-5-23456-R2-Nu4

C	-0.82140200	-0.39002900	0.81855300
C	-2.30961100	-0.77963600	0.65385200
C	-0.47211900	0.94015700	0.15969200
C	-2.78203500	-0.92824300	-0.77548900
H	-2.43322400	-1.73699900	1.18463200
H	-2.92256300	-0.02705800	1.17020500
C	0.85420400	1.18050400	-0.12018000
C	-2.19899500	-2.04027000	-1.60712600
C	-3.73101300	-0.11801900	-1.25646600
C	1.25558500	-1.17949000	0.06135300
C	1.81561800	0.13777000	-0.02962200
H	-2.17996400	-2.98373800	-1.04162500
H	-2.78316200	-2.18263300	-2.52601400
H	-1.15704800	-1.82510100	-1.88260300
H	-4.14572300	0.69440300	-0.65591600
H	-4.11159500	-0.24070900	-2.27351700
C	1.26918100	2.48297100	-0.58238600
N	1.60583600	3.52502200	-0.94635100
C	-1.46096600	1.95050900	0.00631500
N	-2.26547700	2.78039600	-0.08043900
C	3.19437500	0.35127800	-0.28316700
N	4.32461900	0.52345900	-0.47738900
C	2.11552400	-2.30593600	-0.29673900
N	2.79339700	-3.19702800	-0.57357800
C	-0.57047500	-0.25709300	2.29194500
N	-0.37577300	-0.15123500	3.42445500
N	0.02969200	-1.47561300	0.33639100

Py-5-23456-R3

C	-0.07721900	-0.98843700	0.03978000
C	1.20812500	-0.45483300	-0.06044300
C	-1.17342300	-0.12797400	0.11660600
C	1.33338600	0.94061400	-0.11250800
C	-0.92102000	1.25194700	0.10263800
N	0.29574000	1.75990600	-0.02273900
C	-2.06992800	2.21426800	0.29326100
O	-3.12344100	1.87677100	0.76970800
O	-1.78317500	3.43795100	-0.10232400
C	-2.56657600	-0.68348600	0.29008400
O	-2.91169200	-1.27958400	1.27204600
O	-3.31324800	-0.45368400	-0.77469100
C	-0.25027200	-2.48333300	0.11065600

O	0.48390400	-3.19903200	0.73433500
O	-1.29398400	-2.88601900	-0.59345800
C	2.40160000	-1.37405100	-0.17156300
O	2.59895200	-2.07254100	-1.12566900
O	3.15360000	-1.31622600	0.91250800
C	2.69820800	1.55144000	-0.32682000
O	3.63354300	0.91528900	-0.74045200
O	2.73735900	2.83384600	-0.02649400
C	-2.81239500	4.40889100	0.07889400
H	-3.07423600	4.48813400	1.14156700
H	-2.40627900	5.35438800	-0.28930100
H	-3.70569100	4.12371100	-0.49116700
C	-4.68117600	-0.83791200	-0.66289300
H	-5.15584800	-0.27160800	0.14919100
H	-5.14641900	-0.59779100	-1.62247400
H	-4.75956100	-1.91220800	-0.45328400
C	-1.61192200	-4.27610100	-0.51514000
H	-1.85997100	-4.54154400	0.52041400
H	-2.47421600	-4.42709400	-1.16933600
H	-0.75834800	-4.87609300	-0.85289800
C	4.35881900	-2.07596000	0.86696300
H	4.84947700	-1.92985000	1.83278200
H	4.13245200	-3.13726000	0.70507600
H	4.99457300	-1.70832000	0.05074500
C	3.98914700	3.48501100	-0.23515300
H	4.27997600	3.41492900	-1.29092900
H	3.84106500	4.52823900	0.05507700
H	4.76618600	3.01835400	0.38346600

Py-5-23456-R3-Nu1

C	-1.02421300	-0.66967800	0.20200000
C	-0.69828100	0.81473200	0.35161400
C	-2.09300700	-1.05784200	1.24506100
C	1.31477100	-1.05116900	0.25293400
C	-1.77476700	1.75220400	0.72277900
C	0.58556200	1.23733900	0.14731200
O	-1.58317100	-1.03185800	2.47139900
O	-3.23915500	-1.33568500	1.01129000
C	1.65687600	0.31013600	-0.02631200
C	2.39657000	-2.09158400	0.45795800
O	-1.64781900	2.95476100	0.14080000
O	-2.68066500	1.50930700	1.48861600
C	0.92686300	2.70649400	0.18033400
C	-2.52034300	-1.19386200	3.52381100
C	2.99759400	0.75105500	-0.33183900
O	3.14559100	-1.82728200	1.52504100
O	2.52638900	-3.07646800	-0.22167900
C	-2.59397300	3.93465900	0.52954700
O	1.02002500	3.20022100	-1.05223600
O	1.09409700	3.35102800	1.18068900
H	-1.95342200	-1.12947900	4.45745100
H	-3.27104300	-0.39348200	3.47261200

H	-3.02487400	-2.16619500	3.44972600
O	3.81314000	-0.27117800	-0.70032100
O	3.41403400	1.89592400	-0.31240500
C	4.21775500	-2.72695800	1.76472800
H	-2.34009500	4.84569900	-0.02160800
H	-2.54253200	4.11821100	1.61147900
H	-3.61060400	3.60600200	0.27503600
C	1.46970600	4.54172500	-1.13579700
C	5.17305100	0.05972600	-0.90341800
H	3.84159700	-3.74580300	1.92513100
H	4.90367700	-2.73305800	0.90610800
H	4.73186800	-2.36533600	2.66004300
H	2.46909000	4.62845700	-0.68816800
H	0.78020400	5.21563600	-0.60911600
H	1.50586900	4.79271800	-2.20031000
H	5.62188000	0.45950700	0.01715500
H	5.27868100	0.81220700	-1.69624900
H	5.67756700	-0.86729200	-1.19593300
C	-1.57011900	-1.04921100	-1.25046000
C	-2.99995300	-0.50877400	-1.46168800
C	-1.48081600	-2.59194800	-1.36764400
C	-0.72602200	-0.35133300	-2.32962700
O	-3.19165100	0.67999100	-1.33714700
C	-4.09184700	-1.42311100	-1.95200900
O	-2.26333000	-3.30702900	-0.78718800
C	-0.38373500	-3.19813900	-2.21070800
H	-0.80873100	0.73477800	-2.20780000
H	0.33196800	-0.63331000	-2.25780900
H	-1.09101700	-0.61544100	-3.33372200
H	-4.96792400	-0.81739000	-2.20913900
H	-3.75124900	-1.99294900	-2.82955800
H	-4.32629300	-2.14431200	-1.16103400
H	-0.38496300	-2.79863500	-3.23334800
H	0.58947900	-2.97002500	-1.74986300
H	-0.52881600	-4.28426400	-2.23149900
N	0.13139500	-1.52241100	0.44915700

Py-5-23456-R3-Nu3

C	-0.48080000	-1.09602500	0.17404500
C	-0.45040700	0.37760500	0.55206000
C	-1.23112800	-1.91341800	1.24307900
C	1.87182800	-0.97597500	-0.04511000
C	-1.66956300	0.93180700	1.15050800
C	0.69650000	1.09824000	0.32623500
O	-0.70694200	-1.68955900	2.44475200
O	-2.10444700	-2.71496700	1.04225600
C	1.89853900	0.45925900	-0.08455900
C	3.15391800	-1.77948300	-0.13038600
O	-1.73151100	2.27336100	1.13070700
O	-2.57088100	0.26624500	1.61836400
C	0.74790500	2.58601100	0.57905700
C	-1.36482900	-2.32429000	3.52884900

C	3.05807900	1.23281600	-0.47709200
O	3.92126000	-1.58859100	0.94088300
O	3.41754800	-2.54512200	-1.01790700
C	-2.88782400	2.85280500	1.71045800
O	0.41554300	3.26371700	-0.51864300
O	1.05364200	3.09320100	1.62252300
H	-0.83022000	-2.02421300	4.43509200
H	-2.41140300	-1.99263100	3.57460000
H	-1.33944900	-3.41617100	3.41501600
O	4.06040100	0.46741400	-0.97148600
O	3.17226200	2.44401800	-0.42561300
C	5.17152700	-2.26109100	0.93004100
H	-2.77054400	3.93682500	1.61373700
H	-2.97562400	2.57344500	2.76886000
H	-3.78945400	2.51605200	1.18021100
C	0.52863200	4.67342500	-0.41509900
C	5.24820800	1.14795200	-1.32978300
H	5.02890600	-3.34765800	0.86366800
H	5.77013300	-1.92888000	0.07028800
H	5.67319900	-1.99927400	1.86632200
H	1.56101500	4.94859200	-0.16041000
H	-0.14669900	5.05837900	0.36140400
H	0.25433300	5.08090500	-1.39290500
H	5.69556300	1.64193100	-0.45588700
H	5.04623800	1.90936500	-2.09466300
H	5.93177500	0.38924800	-1.72485700
C	-1.16476800	-1.30545200	-1.25892800
C	-2.55110600	-0.65718800	-1.33579100
C	-1.26179000	-2.75055000	-1.55270400
C	-0.23834100	-0.73801800	-2.35775200
C	-2.66283500	0.70861500	-1.63022000
C	-3.72114800	-1.38651500	-1.09596600
N	-1.29913100	-3.85442200	-1.88775500
H	0.68470400	-1.32625000	-2.41803400
H	-0.75293000	-0.77887300	-3.32698800
H	0.02852300	0.30219200	-2.13730100
C	-3.90928500	1.33447500	-1.64865500
H	-1.77121100	1.30597500	-1.81916100
C	-4.96764200	-0.76400700	-1.12828300
H	-3.64829700	-2.44810400	-0.86113600
C	-5.06747700	0.60147700	-1.39485700
H	-3.97102900	2.40206500	-1.86749200
H	-5.86700100	-1.35172300	-0.93704200
H	-6.04355900	1.08920000	-1.41322900
N	0.83497200	-1.71886200	0.12751200

Py-5-23456-R3-Nu2

C	-0.17919000	-1.41385300	0.38792900
C	0.01792300	-0.35677700	1.46920200
C	-0.97794100	-2.62605100	0.87332800
C	1.97128800	-0.94772100	-0.34614900
C	-0.79752500	-0.38625700	2.69114500

C	0.96037500	0.60163200	1.18862700
O	-0.24405600	-3.44460900	1.60837200
O	-2.13146800	-2.81655300	0.58853700
C	1.85932700	0.41271700	0.09930900
C	3.24640200	-1.43777800	-0.99951800
O	-0.85217200	0.80229700	3.31155900
O	-1.36472200	-1.35989400	3.13350100
C	1.18484800	1.79790800	2.08181700
C	-0.95738400	-4.49354800	2.24570300
C	2.74452600	1.47407700	-0.34222200
O	4.31120300	-1.21820200	-0.22883500
O	3.29246600	-1.99996500	-2.05966800
C	-1.60667900	0.84392900	4.51060500
O	0.43214100	2.83394400	1.71281400
O	1.95574300	1.82053800	3.00104900
H	-0.22150500	-5.06503800	2.81915100
H	-1.71722200	-4.06449700	2.91277300
H	-1.44748900	-5.13742500	1.50380100
O	3.43154700	1.14479400	-1.45989200
O	2.86688100	2.57061100	0.16900400
C	5.56291400	-1.58414900	-0.78984700
H	-1.54214600	1.87228200	4.87974600
H	-1.19677900	0.14712800	5.25393100
H	-2.65262300	0.57464700	4.31253900
C	0.64429600	4.03127200	2.44375900
C	4.36394700	2.10397100	-1.92368300
H	5.58745600	-2.65715100	-1.02108000
H	5.74150500	-1.01833100	-1.71499200
H	6.32215600	-1.33847300	-0.04159600
H	1.68574800	4.36113900	2.32951300
H	0.43181200	3.87385200	3.50983400
H	-0.04037800	4.77570500	2.02580800
H	5.13734500	2.29516100	-1.16670500
H	3.86255500	3.05256400	-2.15763600
H	4.81608800	1.68374100	-2.82778600
C	-0.93420100	-0.75020700	-0.80245800
Br	-0.96209400	-1.82071700	-2.41306800
S	-2.64672800	-0.12831700	-0.47096100
H	-0.36928700	0.15134500	-1.06753800
O	-3.65154300	-0.92620500	-1.15951600
O	-2.78208500	0.13084500	0.96070000
C	-2.55934500	1.45507900	-1.29184500
C	-3.16519600	1.60968000	-2.53461500
C	-1.86008100	2.48722200	-0.66545100
C	-3.06803100	2.84693300	-3.17259100
H	-3.70188400	0.77201900	-2.98147300
C	-1.76439800	3.71317700	-1.31985300
H	-1.39115200	2.33164600	0.30999900
C	-2.36883800	3.89162400	-2.56735100
H	-3.53814500	2.99327600	-4.14578100
H	-1.21525300	4.53203600	-0.85319600
H	-2.29151200	4.85519900	-3.07314100
N	1.10684900	-1.87867500	-0.12135300

Py-5-23456-R3-Nu4

C	1.04801200	-1.11098300	0.26858400
C	0.98044500	-1.32391800	1.81152100
C	0.15408600	-2.49937500	2.28175200
H	2.01786200	-1.41249000	2.17174500
H	0.54839200	-0.40982800	2.24088300
C	1.25436800	0.36998400	-0.03762600
C	2.17984200	-1.95039900	-0.33909500
C	-1.19064900	-0.83296600	-0.33301600
C	0.57731700	-3.88344300	1.87614900
C	-0.93893300	-2.29430200	3.02516900
C	2.62001000	0.86844200	-0.11156200
C	0.13048100	1.16179600	-0.03294800
O	2.29592800	-1.72253900	-1.64831700
O	2.85585200	-2.75749600	0.24403900
C	-1.15751100	0.56610800	0.01707800
C	-2.46057200	-1.45462200	-0.87564400
H	0.48422800	-3.98852500	0.78441100
H	1.63363800	-4.06339800	2.12043500
H	-0.04982000	-4.64472800	2.35969800
H	-1.26490800	-1.28365700	3.28575600
H	-1.54930900	-3.12886900	3.37970300
O	2.71775800	2.17882600	-0.40468600
O	3.61446500	0.19183000	0.07697000
C	0.20092800	2.66432700	-0.15259800
C	3.36700800	-2.38915000	-2.29416700
C	-2.33989400	1.35061100	0.29182700
O	-2.90315500	-0.80783800	-1.95538800
O	-2.98378100	-2.44108700	-0.43197400
C	4.02577200	2.72088600	-0.42397000
O	0.43093700	3.23700100	1.02800000
O	0.05509800	3.27178900	-1.17776700
H	3.33144500	-2.09000500	-3.34624200
H	4.32269500	-2.08741700	-1.84337500
H	3.26027300	-3.47849200	-2.20428100
O	-3.45501100	0.58782600	0.41745300
O	-2.39220200	2.55756300	0.44829900
C	-4.12699700	-1.28055000	-2.49503400
H	3.91671300	3.78066000	-0.67574400
H	4.64465100	2.21506700	-1.17699200
H	4.50887300	2.61115200	0.55665400
C	0.42966700	4.65443400	1.02085300
C	-4.66614800	1.28397400	0.63646700
H	-4.03818500	-2.33092000	-2.80286300
H	-4.92698700	-1.19893400	-1.74565200
H	-4.35070900	-0.64850500	-3.35959500
H	-0.54269600	5.02893900	0.67279100
H	1.21727300	5.03794000	0.35736800
H	0.61369900	4.97207900	2.05176900
H	-4.89615100	1.94778400	-0.20912900
H	-4.60925300	1.89191000	1.54938800

H	-5.44669900	0.52283600	0.73933900
N	-0.17650700	-1.61924700	-0.35450200

Py-5-23456-R4

C	0.00000000	0.00000000	1.12703600
C	0.00000000	1.20158700	0.42264800
C	0.00000000	-1.20158700	0.42264800
C	0.00000000	1.12910900	-0.96852500
C	0.00000000	-1.12910900	-0.96852500
N	0.00000000	0.00000000	-1.62998200
F	0.00000000	-2.25233000	-1.65680100
F	0.00000000	-2.35659800	1.05941100
F	0.00000000	0.00000000	2.43902200
F	0.00000000	2.35659800	1.05941100
F	0.00000000	2.25233000	-1.65680100

Py-5-23456-R4-Nu1

C	0.01042700	-0.17660400	-0.41741200
C	0.81420100	1.05369000	-0.21588900
C	2.00556300	-1.33223600	0.07417000
C	2.16036300	1.01352400	-0.03687900
C	2.83060800	-0.22417500	0.10021700
F	0.19692500	2.24994300	-0.38988800
F	2.87053000	2.14267200	0.02038000
F	4.16482000	-0.27654200	0.31555000
F	2.59040100	-2.52656400	0.28786600
F	-0.34811900	-0.18443500	-1.81261500
C	-1.37868900	-0.15258700	0.32314700
C	-2.27423900	-1.33684000	-0.10762900
C	-2.19278900	1.09987300	-0.05862600
C	-1.12233800	-0.25010300	1.83168300
O	-2.00222000	-2.12191900	-0.97490700
C	-3.56349800	-1.48788400	0.68368200
O	-2.81507600	1.11646700	-1.09508900
C	-2.28759000	2.24618600	0.92158000
H	-0.68918200	-1.23352400	2.05796600
H	-0.39384500	0.51118900	2.14406200
H	-2.03900700	-0.11586000	2.42122600
H	-3.34244700	-2.02286500	1.61970600
H	-4.01416100	-0.52168300	0.94829300
H	-4.27037400	-2.08711600	0.09862100
H	-3.03316100	1.98313900	1.68875200
H	-1.33693500	2.44465500	1.42688100
H	-2.63299800	3.13926500	0.38832800
N	0.71533800	-1.34518800	-0.08768100

Py-5-23456-R4-Nu3

C	-0.38665800	1.07198200	-0.19728700
C	-1.07153000	0.19651400	-1.16653800
C	-1.76988200	0.09341300	1.43104300

C	-2.15152300	-0.54650800	-0.80078900
C	-2.57385900	-0.59784100	0.54635700
F	-0.69027900	0.20446800	-2.46806100
F	-2.81981700	-1.25384800	-1.71267900
F	-3.63583500	-1.34481300	0.91678700
F	-2.06568700	-0.00836600	2.73719100
F	-0.77439600	2.45492800	-0.50668300
C	1.18415800	1.16114100	-0.34737700
C	1.80170500	-0.21018300	-0.07654400
C	1.62441400	2.12696500	0.67947700
C	1.64073100	1.75102200	-1.69800500
C	1.74308600	-1.19892800	-1.06843300
C	2.38808500	-0.52994400	1.15158500
N	2.02162200	2.90942400	1.42888200
H	1.14946500	2.71829700	-1.85374700
H	2.72981000	1.89412500	-1.68845500
H	1.37785500	1.08911200	-2.52761200
C	2.27184400	-2.46791400	-0.84050500
H	1.26520200	-0.98293000	-2.02459800
C	2.91925900	-1.80021500	1.37988000
H	2.42843900	0.21982500	1.94271600
C	2.86662000	-2.77321500	0.38418700
H	2.21529400	-3.22311100	-1.62612800
H	3.37500300	-2.02698800	2.34506700
H	3.28282400	-3.76611900	0.56160300
N	-0.71711800	0.79753500	1.12966900

Py-5-23456-R4-Nu2

C	1.35591900	0.28135300	-0.47635200
C	2.04070100	-1.02751900	-0.37375600
C	3.30342900	-1.13133500	0.12019400
C	3.26689200	1.17251100	0.58450600
C	3.97853300	-0.00689800	0.64781700
F	3.86033200	2.27246200	1.07576200
F	5.21814900	-0.11706500	1.17043700
F	3.93237100	-2.30595100	0.10064400
F	1.47432100	-2.08921800	-0.97992100
F	1.16840200	0.53608800	-1.87824400
C	-0.11412300	0.31999200	0.08100000
Br	-1.01359300	1.94412900	-0.47399900
S	-1.16724300	-1.13023700	-0.30282500
H	-0.06330600	0.33872800	1.17557900
O	-1.23477500	-1.31735000	-1.74693800
O	-0.70940100	-2.20065400	0.58200100
C	-2.79613300	-0.67233600	0.26682100
C	-3.75824300	-0.29113600	-0.66478300
C	-3.07232100	-0.74882100	1.63047700
C	-5.03372800	0.03734400	-0.21006400
H	-3.49869200	-0.25583400	-1.72341300
C	-4.34878800	-0.40678500	2.07324000
H	-2.30172700	-1.08073000	2.32803900
C	-5.32445100	-0.01489700	1.15464600

H	-5.80289200	0.33647900	-0.92297300
H	-4.58475000	-0.45505000	3.13685800
H	-6.32337200	0.24804500	1.50568100
N	2.05878800	1.33085800	0.12204200

Py-5-23456-R4-Nu4

C	0.53425500	0.03577000	0.90385400
C	2.06647600	0.00698300	0.81387100
C	-0.10535400	-1.12486200	0.24777100
C	2.60154100	0.22632200	-0.58040200
H	2.42220800	0.79566300	1.49293300
H	2.39680500	-0.96313100	1.20896600
C	-1.36746100	-1.03478100	-0.24281400
C	2.79463300	1.65857600	-0.99204500
C	2.85741100	-0.80012900	-1.39856300
C	-1.24882000	1.30244400	0.08329900
C	-2.02473200	0.22070400	-0.28332600
H	3.55008800	2.14068100	-0.35037900
H	3.11322900	1.74599900	-2.03949800
H	1.85172600	2.20414700	-0.84056800
H	2.69216600	-1.83054500	-1.07515300
H	3.22111500	-0.63616600	-2.41630000
F	-1.78574100	2.52567400	-0.09616700
F	-3.28385900	0.32724500	-0.76764000
F	-1.99967600	-2.10821300	-0.72779800
F	0.55023800	-2.30943600	0.23933800
F	0.32591100	-0.13254200	2.35562600
N	-0.02241100	1.26751800	0.52570000

Py-5-23456-R5

C	0.00000000	1.19990300	0.24807200
C	0.00000000	0.00000000	0.94578600
C	-0.01951100	1.12530400	-1.14338700
C	0.00000000	-1.19990300	0.24807200
C	0.01951100	-1.12530400	-1.14338700
N	0.12317000	-2.36534300	-1.96961400
O	-0.38841200	-2.33422700	-3.04814300
O	0.73878900	-3.26900500	-1.46521500
N	-0.01687700	-2.48980300	0.97654900
O	-0.94986500	-3.20378600	0.74852600
O	0.90020800	-2.67505100	1.72589800
N	0.00000000	0.00000000	2.43005400
O	-0.76939300	-0.75637500	2.95207700
O	0.76939300	0.75637500	2.95207700
N	0.01687700	2.48980300	0.97654900
O	-0.90020800	2.67505100	1.72589800
O	0.94986500	3.20378600	0.74852600
N	-0.12317000	2.36534300	-1.96961400
O	-0.73878900	3.26900500	-1.46521500
O	0.38841200	2.33422700	-3.04814300
N	0.00000000	0.00000000	-1.80695300

Py-5-23456-R5-Nu1

C	0.76677700	0.22722500	0.49961700
C	-0.04843100	-1.05757000	0.45844900
C	-1.36213700	-1.01403800	0.05874100
C	-1.29271100	1.33789300	0.37143000
C	-2.03652600	0.20610200	-0.08686400
N	-2.03112700	2.61186000	0.66430000
O	-1.63021600	3.60882800	0.13066800
O	-2.93449400	2.52685800	1.45421800
N	-3.24067700	0.36364600	-0.82261500
O	-3.58359800	1.50044500	-1.09460100
O	-3.85934400	-0.62852700	-1.15269300
N	-2.09738000	-2.26611100	-0.26141700
O	-2.84493300	-2.68064400	0.57680500
O	-1.88338000	-2.72950100	-1.34730600
N	0.58023300	-2.29794400	0.79720600
O	-0.06142500	-3.32405900	0.74365800
O	1.74610400	-2.24846500	1.14916600
N	1.62582300	0.19928000	1.81277100
O	0.98337400	0.12898000	2.82574100
O	2.81998300	0.25047200	1.73674300
C	1.67354600	0.44748600	-0.76136900
C	2.54594800	-0.80972300	-1.06483100
C	2.52762000	1.72842300	-0.67279800
C	0.73875100	0.63294200	-1.97778000
O	2.04594900	-1.70323900	-1.70232500
C	3.97592500	-0.89983300	-0.59330600
O	2.48210200	2.49196500	0.25448400
C	3.37511800	2.03355900	-1.89274800
H	0.07345700	-0.23350700	-2.07682800
H	0.15021400	1.55539800	-1.88669200
H	1.33190600	0.68806200	-2.89845000
H	3.97722800	-1.53851400	0.30084800
H	4.56659700	-1.39227500	-1.37621100
H	4.41306400	0.06498400	-0.31659300
H	3.65607800	1.14107400	-2.46687700
H	2.79457800	2.69450900	-2.55412500
H	4.27128300	2.57744600	-1.57138300
N	-0.07382500	1.37543000	0.69812500

Py-5-23456-R5-Nu3

C	-1.40485200	-0.32536600	-0.33656400
C	-0.95981700	1.11798900	-0.27416700
C	0.67050500	-0.77769600	-1.29199700
C	0.37873800	1.39904200	-0.38567300
C	1.28733700	0.41816100	-0.80495400
N	0.89050000	2.75479400	-0.05066700
O	0.86677700	3.05895700	1.11056400
O	1.28919100	3.41472200	-0.96729600
N	2.67776200	0.46923600	-0.52116900

O	3.12256000	1.46324800	0.01872900
O	3.35110300	-0.49833600	-0.82459700
N	1.47036500	-1.70852100	-2.15233400
O	1.92021400	-1.23625700	-3.16238100
O	1.55193000	-2.84958300	-1.78908700
N	-2.75725300	-0.38071300	-1.11891600
O	-2.76269200	0.21909100	-2.15877400
O	-3.65090800	-1.05209100	-0.68376800
N	-1.93087500	2.15754600	-0.10990300
O	-3.07001500	1.82187300	0.16109000
O	-1.58263700	3.30917700	-0.25964400
C	-1.52187000	-1.03795100	1.07864800
C	-0.10332600	-1.03404800	1.68289900
C	-1.93279200	-2.44257500	0.87205600
C	-2.53962600	-0.37812400	2.02653600
C	0.34782800	0.07321100	2.41020900
C	0.79655500	-2.07101600	1.40536400
N	-2.23330600	-3.55189200	0.78165800
H	-3.55263500	-0.45131200	1.62063000
H	-2.48927400	-0.89041600	2.99610900
H	-2.30667000	0.68047500	2.17716100
C	1.68218000	0.16495400	2.80437200
H	-0.31952000	0.90465400	2.63386200
C	2.12830200	-1.98216600	1.80662600
H	0.47049200	-2.93816800	0.82915800
C	2.57835000	-0.85778300	2.49763900
H	2.02188800	1.05258100	3.33932800
H	2.81902500	-2.78824200	1.55625100
H	3.62587200	-0.77588100	2.79078100
N	-0.54553900	-1.11186200	-1.17837300

Py-5-23456-R5-Nu2

C	0.53750900	0.22631600	-1.16680100
C	1.29342700	-1.02296900	-0.77833800
C	2.09857900	-0.96779100	0.32592100
C	2.09936200	1.39858800	0.09040700
C	2.43549500	0.27314800	0.89968900
N	2.85945500	2.67858300	0.27293300
O	2.21255200	3.67700000	0.42525700
O	4.05682800	2.59474500	0.19660900
N	3.00979600	0.42491200	2.18918700
O	3.10642900	1.55603800	2.62821400
O	3.35296200	-0.57229400	2.79225100
N	2.65520300	-2.20982500	0.92326600
O	3.79628100	-2.45974200	0.65759600
O	1.91259500	-2.83423700	1.62707600
N	1.02774500	-2.24901400	-1.47284500
O	1.68128500	-3.22855800	-1.19430900
O	0.15347600	-2.22158800	-2.31517800
N	0.34842300	0.25632200	-2.70747500
O	1.36764500	0.16425100	-3.33662300
O	-0.75410600	0.40999900	-3.14443400

C	-0.80386900	0.37934500	-0.39994600
Br	-1.72622900	2.03479400	-0.75211400
S	-1.97665600	-1.05301600	-0.40411100
H	-0.51008800	0.42291400	0.65954300
O	-2.67539800	-1.15821900	-1.67149600
O	-1.20102600	-2.16794100	0.13596500
C	-3.14746000	-0.56866300	0.84422800
C	-4.36659400	-0.02117000	0.44954300
C	-2.82566000	-0.79419000	2.18177400
C	-5.28551400	0.32916000	1.43600300
H	-4.58082800	0.11933700	-0.61052300
C	-3.75367400	-0.43358300	3.15664300
H	-1.87440400	-1.25818600	2.44783000
C	-4.97623800	0.12746100	2.78276000
H	-6.24652500	0.75919100	1.15248200
H	-3.52494900	-0.60009200	4.20957900
H	-5.69991100	0.40518900	3.55031400
N	1.32407000	1.40653500	-0.91338300

Py-5-23456-R5-Nu4

C	1.10048400	-1.02416800	-0.07636800
C	1.46278300	-1.53378800	1.32764300
C	1.12912400	0.47815900	-0.22728700
C	0.43264300	-1.10593400	2.35487300
H	1.52175700	-2.62790500	1.27076800
H	2.45926600	-1.14620900	1.57409200
C	-0.03993700	1.18733300	-0.22455500
C	-0.63962500	-2.10750800	2.68941200
C	0.49455700	0.10178300	2.92488200
C	-1.18332000	-0.87882000	-0.53911800
C	-1.28027300	0.52962800	-0.30549100
H	-0.18727300	-3.01341700	3.12341200
H	-1.35840200	-1.69648600	3.41008500
H	-1.18515700	-2.42777700	1.78864300
H	1.28964000	0.81106800	2.68021800
H	-0.25456200	0.42191900	3.65289100
N	-2.39973800	-1.61347400	-1.02222300
O	-2.73893600	-2.58170500	-0.39796100
O	-2.89829900	-1.19855600	-2.03473900
N	-2.50532800	1.12620500	0.08023700
O	-3.47054600	0.39420000	0.21718700
O	-2.53728800	2.32828100	0.26417500
N	-0.01868800	2.66864700	-0.11469900
O	-0.21102000	3.28092700	-1.12607200
O	0.17963900	3.11410100	0.98209100
N	2.40666500	1.12021700	-0.24617700
O	2.47016300	2.31001700	-0.47310300
O	3.37967000	0.41555600	-0.03783000
N	2.12373100	-1.61502200	-1.10614300
O	2.12059400	-1.10431900	-2.19537600
O	2.78285900	-2.56042600	-0.77288600
N	-0.14358100	-1.59884500	-0.51213100

Py-5-32456-R1

C	0.96456400	-0.03902100	0.00000000
C	0.25864700	0.03686100	1.21094000
C	0.25864700	0.03686100	-1.21094000
C	-1.14122200	-0.01937500	1.14367700
C	-1.14122200	-0.01937500	-1.14367700
C	2.46919200	-0.36924100	0.00000000
C	0.96800900	0.27601200	2.54793300
C	0.96800900	0.27601200	-2.54793300
C	-2.06455500	-0.08669400	2.36964300
C	-2.06455500	-0.08669400	-2.36964300
F	1.21938200	-0.84140200	-3.20788000
F	2.11152000	0.92607800	-2.35763300
F	0.21347100	1.05588000	-3.31596500
F	-2.34194200	1.12381400	-2.83619400
F	-1.50864300	-0.80361800	-3.34297100
F	-3.20534200	-0.66822100	-2.04291400
F	-3.20534200	-0.66822100	2.04291400
F	-1.50864300	-0.80361800	3.34297100
F	-2.34194200	1.12381400	2.83619400
F	0.21347100	1.05588000	3.31596500
F	1.21938200	-0.84140200	3.20788000
F	2.11152000	0.92607800	2.35763300
F	2.75956900	-1.11083200	1.06443300
F	2.75956900	-1.11083200	-1.06443300
F	3.24886700	0.69563000	0.00000000
N	-1.79388400	-0.08047500	0.00000000

Py-5-32456-R1-Nu1

C	0.84575000	-0.05368100	-0.29527400
C	0.43309500	1.43430700	-0.40331000
C	-0.47748000	-0.86258300	-0.29197000
C	-1.70682900	-0.26531100	-0.09674900
C	-1.80553600	1.13488400	0.11011000
C	1.43914300	2.54541000	-0.64306500
C	-2.99461800	1.89056400	0.61803700
C	-3.01401700	-1.04670900	-0.22698200
C	-0.38358000	-2.35307200	-0.53401900
C	1.56432700	-0.38970700	-1.65191600
F	0.64208200	-0.31273500	-2.62681400
F	2.50261300	0.49304200	-1.98558000
F	2.10209000	-1.59787200	-1.74552300
F	0.79473500	-2.85895400	-0.13676800
F	-0.51558300	-2.70798500	-1.81873800
F	-1.29592100	-3.05124400	0.15856100
F	-4.02866700	-0.23827200	-0.53742200
F	-2.97653600	-1.94471900	-1.21409000
F	-3.35073400	-1.67937400	0.89817300
F	-3.87040200	2.30375800	-0.31748900
F	-2.60874000	3.00438800	1.25285900

F	-3.70589800	1.17531300	1.50945900
F	1.57981500	2.87824800	-1.93320800
F	1.07909900	3.66618300	-0.01311900
F	2.67023900	2.25143500	-0.18262500
C	1.72147300	-0.22947300	1.06724400
C	3.24876000	-0.03306600	0.89290600
C	1.65661000	-1.60907700	1.79476200
C	1.13535200	0.74844800	2.11174800
O	3.83538100	-0.49942600	-0.04693500
C	3.99626900	0.66046500	2.00907600
O	2.65345700	-2.27542100	1.92935500
C	0.38884600	-2.00819000	2.52225000
H	1.37416500	1.79056100	1.87342900
H	0.04135900	0.65727700	2.14801500
H	1.52942000	0.53507300	3.11426800
H	3.65926300	1.69844900	2.12488200
H	3.82199000	0.13765200	2.96037500
H	5.06513700	0.64022400	1.77014600
H	0.48539300	-1.66533400	3.56352000
H	-0.52792700	-1.58091500	2.10600700
H	0.33138000	-3.10273400	2.52700100
N	-0.73428500	1.89691300	-0.13045100

Py-5-32456-R1-Nu3

C	-1.17368800	0.42537900	-0.04022900
C	-0.85210800	-0.68806800	-1.06633500
C	0.05271900	1.36426600	-0.06615300
C	1.31223000	0.85717700	-0.35323500
C	1.44433100	-0.45955900	-0.85027900
C	-1.86343100	-1.60310400	-1.74854800
C	2.69986200	-1.27639500	-0.95957600
C	2.58695500	1.69142600	-0.24674100
C	-0.08088800	2.82006900	0.32451900
C	-2.47130700	1.19639200	-0.46811600
F	-2.17829200	2.19081000	-1.30755900
F	-3.33930600	0.41501700	-1.10718500
F	-3.16854900	1.70802600	0.54960200
F	-1.25254600	3.11296300	0.89839600
F	0.03920900	3.67667300	-0.69721400
F	0.84332300	3.17716400	1.23956400
F	3.55023700	1.20959200	-1.03433300
F	2.43694900	2.95949000	-0.62832500
F	3.06569600	1.69710300	1.00070200
F	3.40421900	-1.10427800	-2.09421000
F	2.41779400	-2.58242200	-0.91682100
F	3.55011900	-1.03785200	0.05452300
F	-2.41365900	-1.07305500	-2.85299400
F	-1.28295000	-2.73511700	-2.14580500
F	-2.87458100	-1.98043100	-0.94839400
C	-1.39018600	-0.21300300	1.45111300
C	-0.37241100	-1.34136400	1.68222100
C	-2.74560800	-0.78221500	1.58976200

C	-1.26881000	0.83699300	2.57690200
C	0.88472000	-1.06305500	2.23156900
C	-0.67208900	-2.66209100	1.32964200
N	-3.80574000	-1.19950100	1.77229700
H	-2.09331700	1.55393300	2.54645700
H	-1.27591300	0.31679000	3.54362300
H	-0.32503900	1.38791800	2.49263500
C	1.81944300	-2.08037500	2.41727100
H	1.15862700	-0.04219900	2.49713100
C	0.26278900	-3.67886600	1.51376000
H	-1.64748500	-2.89997500	0.90158700
C	1.51260000	-3.39125200	2.05902300
H	2.79809000	-1.83928400	2.83416700
H	0.00929800	-4.70054300	1.22722100
H	2.24711400	-4.18563400	2.20001900
N	0.35415400	-1.08739400	-1.28541000

Py-5-32456-R1-Nu2

C	-0.19953700	0.34383000	0.81910500
C	-1.10472800	-0.88290000	0.65425500
C	-0.96545900	1.56308900	0.28571200
C	-2.28477000	-0.82271700	-0.07232700
C	-2.70086400	0.38087800	-0.68150900
C	-3.23748100	-2.01624600	-0.14863500
C	-0.59614200	-2.20726600	1.18435000
C	-3.84275000	0.55832800	-1.63792000
C	-0.42681300	2.96234900	0.50237200
C	0.03734400	0.60284300	2.34234700
F	-1.10861900	1.03567700	2.87567600
F	0.38675500	-0.46514400	3.04488000
F	0.95386000	1.54741200	2.55308400
F	0.90701500	3.01093500	0.44343900
F	-0.77043200	3.46792200	1.70024300
F	-0.89244100	3.81031700	-0.40997900
F	-5.03967000	0.78421700	-1.06533300
F	-4.00481000	-0.50512200	-2.44428400
F	-3.62815400	1.60816600	-2.43753900
F	-2.99800100	-2.80037300	-1.19784100
F	-3.20069100	-2.77655000	0.94630000
F	-4.50407600	-1.60413800	-0.24133400
F	-1.09573400	-2.57105200	2.37044400
F	-0.84721600	-3.21538100	0.33361800
F	0.74222300	-2.21733400	1.33853900
C	1.14927000	0.10008000	-0.02372200
Br	1.08793700	0.90237500	-1.78977400
S	2.83863400	0.36703500	0.68610800
H	1.20183900	-0.96586800	-0.24675900
O	3.25216600	1.76008600	0.62255100
O	2.85647100	-0.35737200	1.95287500
C	3.84515600	-0.56011900	-0.45918500
C	4.60053200	0.12296000	-1.40863300
C	3.87484800	-1.94912400	-0.34061800

C	5.40208900	-0.61581100	-2.27723100
H	4.55410100	1.21164800	-1.45550300
C	4.67554800	-2.67468300	-1.22027000
H	3.28577400	-2.44613800	0.43267500
C	5.43508500	-2.00836200	-2.18432200
H	6.00291800	-0.10233900	-3.02857900
H	4.71273700	-3.76204300	-1.14762000
H	6.06252100	-2.58128200	-2.86853400
N	-2.03613800	1.51427000	-0.41665000

Py-5-32456-R1-Nu4

C	-1.20356500	-0.48173100	0.05695400
C	-1.85422300	-0.75712100	1.46015800
C	0.24485700	-1.00260700	-0.07154200
C	-1.03325700	-0.23404900	2.61698400
H	-2.84351100	-0.28466000	1.46990400
H	-2.01065800	-1.83485200	1.57284200
C	-1.18221500	1.02765600	-0.25098400
C	1.31320900	-0.13809200	-0.20356300
C	-1.19466500	1.22027200	2.97872800
C	-0.20971300	-1.04043700	3.29610900
C	1.11763800	1.26705300	-0.19479900
H	-2.25774100	1.46260600	3.13368200
H	-0.64125200	1.46248200	3.89575700
H	-0.83250700	1.87829900	2.17457400
H	-0.10385500	-2.09530700	3.03394700
H	0.38478200	-0.66364300	4.13226400
C	2.72601900	-0.63803700	-0.50727100
C	0.49838600	-2.48250400	0.10373300
C	2.17650000	2.31605100	-0.08652100
C	-2.45907400	1.81668800	-0.45730300
C	-2.09083900	-1.16599700	-1.00879700
F	-1.72239400	-0.81737600	-2.24174200
F	-2.05041900	-2.50011900	-0.95759500
F	-3.37945200	-0.83271700	-0.86471700
F	-3.43488500	1.51511200	0.42098300
F	-2.99349000	1.62704100	-1.67798700
F	-2.25145600	3.12601600	-0.34226400
F	2.67637600	2.76405100	-1.25648000
F	3.23434100	1.90651600	0.63647700
F	1.70052000	3.40716100	0.52944100
F	3.45969300	-0.85032300	0.58375300
F	2.73023200	-1.76181200	-1.22861200
F	3.38662900	0.25144200	-1.25647000
F	0.55112700	-3.17892300	-1.04317500
F	1.65019700	-2.72643000	0.75050900
F	-0.43604900	-3.09374400	0.85156000
N	-0.12547600	1.75844900	-0.27677500

Py-5-32456-R2

C	0.00000000	0.00000000	1.02001000

C	0.00000000	1.20670400	0.30565300
C	0.00000000	-1.20670400	0.30565300
C	0.00000000	1.14017600	-1.10217300
C	0.00000000	-1.14017600	-1.10217300
N	0.00000000	0.00000000	-1.77552500
C	0.00000000	-2.47159000	0.98549000
N	0.00000000	-3.48879000	1.52843700
C	0.00000000	-2.36285900	-1.88012600
N	0.00000000	-3.34205900	-2.48756600
C	0.00000000	0.00000000	2.45862000
N	0.00000000	0.00000000	3.61122500
C	0.00000000	2.47159000	0.98549000
N	0.00000000	3.48879000	1.52843700
C	0.00000000	2.36285900	-1.88012600
N	0.00000000	3.34205900	-2.48756600

Py-5-32456-R2-Nu1

C	-0.54616300	0.58165000	0.43657200
C	0.82218000	1.22684000	0.24662500
C	-0.30523900	-0.52797300	1.47652500
C	1.92144000	0.38564800	0.21018200
C	1.81235500	-0.94045100	0.69645400
C	3.20119100	0.88085300	-0.22569900
N	4.22348400	1.26863400	-0.59657600
C	0.94757000	2.62563300	0.02854500
N	1.03422300	3.76492700	-0.16735200
C	2.90212100	-1.86264200	0.60207700
N	3.78064800	-2.60927400	0.49373500
C	-1.25643300	-0.76080500	2.53565900
N	-1.97036800	-0.94145000	3.42660300
C	-1.47481600	1.59539900	0.97510300
N	-2.15601100	2.43210000	1.38076700
C	-1.15286900	0.05083200	-0.93816300
C	-2.56106200	-0.52807800	-0.69572400
C	-0.32038800	-1.14005900	-1.46851300
C	-1.23806700	1.19782500	-1.95269700
O	-3.03947100	-0.57282400	0.40860900
C	-3.33213800	-0.98275100	-1.91115200
O	-0.59382800	-2.24891600	-1.07438300
C	0.72952600	-0.90544400	-2.52359400
H	-1.92015300	1.98226100	-1.59309100
H	-0.25434300	1.65402000	-2.12220700
H	-1.60978800	0.83842500	-2.92134000
H	-3.84557700	-0.10799000	-2.33902200
H	-2.68497100	-1.41198900	-2.68622900
H	-4.08894100	-1.71113500	-1.59961800
H	0.22009900	-0.82100800	-3.49719100
H	1.29049100	0.02355200	-2.36817300
H	1.40406800	-1.76891900	-2.55338000
N	0.75377600	-1.26953300	1.47042900

Py-5-32456-R2-Nu3

C	0.10727200	0.64376300	0.41392200
C	1.57877200	1.00201600	0.27695900
C	0.06325100	-0.71811100	1.12757100
C	2.50199900	-0.01752900	0.12336800
C	2.14374700	-1.36272300	0.38877400
C	3.86911900	0.29804000	-0.20328800
N	4.96456400	0.54355600	-0.47153700
C	1.97352400	2.36903900	0.24906300
N	2.27114200	3.48867200	0.22235500
C	3.07052400	-2.43579800	0.19687300
N	3.82174100	-3.29999600	0.02501800
C	-1.06591400	-1.00943700	1.97409600
N	-1.96583600	-1.21970800	2.67022500
C	-0.56983000	1.65653700	1.24877700
N	-1.08390200	2.45064600	1.90761300
C	-0.66089000	0.60257600	-0.99706900
C	-2.07957900	0.06781300	-0.79369700
C	0.08263200	-0.31729800	-1.88099700
C	-0.62273000	1.98905200	-1.66494300
C	-3.11429100	0.93657200	-0.42585900
C	-2.35132300	-1.30175900	-0.90079100
N	0.65108300	-1.00360300	-2.61303700
H	0.40811800	2.26728800	-1.91935600
H	-1.21888700	1.97093100	-2.58618500
H	-1.03002400	2.75652000	-0.99407900
C	-4.38889000	0.44109100	-0.15638400
H	-2.93293000	2.00745200	-0.33588500
C	-3.62694500	-1.79489000	-0.63391100
H	-1.55774400	-1.99379400	-1.18951200
C	-4.64862600	-0.92528400	-0.25672300
H	-5.18265900	1.13082100	0.13318700
H	-3.81888900	-2.86513800	-0.71922600
H	-5.64687500	-1.31042600	-0.04431000
N	0.97105800	-1.63180500	0.99378000

Py-5-32456-R2-Nu2

C	-0.95026100	0.31318800	-0.08210600
C	-1.90780500	-0.76291900	-0.55739900
C	-1.85281200	1.55583700	-0.19813500
C	-3.12019700	-0.79615800	0.11616300
C	-3.54522700	0.38633500	0.78735100
C	-4.02046700	-1.90664600	-0.02665400
N	-4.74348600	-2.80119500	-0.13143400
C	-1.61698300	-1.62247800	-1.65108200
N	-1.39420300	-2.29753200	-2.56564600
C	-4.74149400	0.40835500	1.57853900
N	-5.69577400	0.41200600	2.23244800
C	-1.51176900	2.70189500	-0.99330600
N	-1.29338200	3.61851200	-1.66460000
C	-0.62973800	0.13021400	1.36472300
N	-0.38277100	0.02682300	2.48613900

C	0.36826400	0.37447100	-0.86568400
Br	1.45007300	1.89499100	-0.37067000
S	1.37034700	-1.21835400	-0.70977700
H	0.18175300	0.43759600	-1.94598100
O	0.56622700	-2.15716700	0.06705700
O	1.77212800	-1.51630700	-2.07816000
C	2.83434700	-0.86828000	0.23343200
C	2.79239300	-1.00508900	1.61938100
C	3.99914600	-0.52980400	-0.45305100
C	3.96111500	-0.76762300	2.34068500
H	1.86473300	-1.28884500	2.11792100
C	5.15853100	-0.30080200	0.28289400
H	3.98820300	-0.45202500	-1.54072300
C	5.13601700	-0.41517400	1.67473900
H	3.95373000	-0.86501400	3.42658000
H	6.08252000	-0.03496600	-0.23133700
H	6.04711400	-0.23317700	2.24639700
N	-3.00772100	1.55870700	0.40351100

Py-5-32456-R2-Nu4

C	-0.90399400	-0.00700900	0.67720900
C	-2.14652200	0.57601400	-0.06441900
C	0.33208200	0.86527700	0.47345700
C	-1.90358600	0.88459000	-1.52313500
H	-2.97087900	-0.14868700	0.04445900
H	-2.45018300	1.49301200	0.46088200
C	-0.55175500	-1.43928000	0.23408100
C	1.55238900	0.31642300	0.15409700
C	-1.64323000	-0.26860200	-2.45620800
C	-1.93506900	2.14969500	-1.95440200
C	1.70324900	-1.07931200	-0.07788300
H	-2.36800300	-1.08136900	-2.29440600
H	-1.70340200	0.05596400	-3.50308800
H	-0.64095500	-0.69553800	-2.29295900
H	-2.10370000	2.98084400	-1.26572300
H	-1.78397300	2.38861000	-3.00989500
C	2.70016500	1.17809000	0.01002000
N	3.62350400	1.86065600	-0.10581400
C	0.17500600	2.26829400	0.66416900
N	0.00817700	3.40096900	0.84353200
C	2.97130400	-1.64943800	-0.40392000
N	4.00567000	-2.10379200	-0.66278500
C	-1.65257200	-2.36685700	0.17036600
N	-2.56324100	-3.07969900	0.12013200
C	-1.24810400	-0.04778600	2.12538100
N	-1.50926800	-0.08767300	3.24898700
N	0.62443300	-1.88666400	-0.06007400

Py-5-32456-R3

C	-0.07721900	-0.98843700	0.03978000
C	1.20812500	-0.45483300	-0.06044300

C	-1.17342300	-0.12797400	0.11660600
C	1.33338600	0.94061400	-0.11250800
C	-0.92102000	1.25194700	0.10263800
N	0.29574000	1.75990600	-0.02273900
C	-2.06992800	2.21426800	0.29326100
O	-3.12344100	1.87677100	0.76970800
O	-1.78317500	3.43795100	-0.10232400
C	-2.56657600	-0.68348600	0.29008400
O	-2.91169200	-1.27958400	1.27204600
O	-3.31324800	-0.45368400	-0.77469100
C	-0.25027200	-2.48333300	0.11065600
O	0.48390400	-3.19903200	0.73433500
O	-1.29398400	-2.88601900	-0.59345800
C	2.40160000	-1.37405100	-0.17156300
O	2.59895200	-2.07254100	-1.12566900
O	3.15360000	-1.31622600	0.91250800
C	2.69820800	1.55144000	-0.32682000
O	3.63354300	0.91528900	-0.74045200
O	2.73735900	2.83384600	-0.02649400
C	-2.81239500	4.40889100	0.07889400
H	-3.07423600	4.48813400	1.14156700
H	-2.40627900	5.35438800	-0.28930100
H	-3.70569100	4.12371100	-0.49116700
C	-4.68117600	-0.83791200	-0.66289300
H	-5.15584800	-0.27160800	0.14919100
H	-5.14641900	-0.59779100	-1.62247400
H	-4.75956100	-1.91220800	-0.45328400
C	-1.61192200	-4.27610100	-0.51514000
H	-1.85997100	-4.54154400	0.52041400
H	-2.47421600	-4.42709400	-1.16933600
H	-0.75834800	-4.87609300	-0.85289800
C	4.35881900	-2.07596000	0.86696300
H	4.84947700	-1.92985000	1.83278200
H	4.13245200	-3.13726000	0.70507600
H	4.99457300	-1.70832000	0.05074500
C	3.98914700	3.48501100	-0.23515300
H	4.27997600	3.41492900	-1.29092900
H	3.84106500	4.52823900	0.05507700
H	4.76618600	3.01835400	0.38346600

Py-5-32456-R3-Nu1

C	-0.86739900	-0.26525100	-0.09320100
C	-0.80122700	1.23368800	-0.41235500
C	0.52688100	-0.82027500	-0.39755300
C	-1.89496900	-0.95230000	-1.00330400
C	-2.05974600	1.92751400	-0.76229000
C	0.64954600	-2.23352900	-0.81856700
C	1.62599400	-0.01649300	-0.24781500
O	-1.70735100	-0.61604300	-2.27712200
O	-2.74494400	-1.71659800	-0.63191900
O	-1.92471600	3.24767000	-0.93577600
O	-3.13836800	1.37750400	-0.85425900

C	1.47624100	1.39856200	-0.09384400
O	1.82040200	-2.78607100	-0.47319500
O	-0.18719700	-2.85787400	-1.43115000
C	3.02879500	-0.55211800	-0.38553800
C	-2.55768400	-1.26512700	-3.20439400
C	-3.12132800	3.95303100	-1.21737800
C	2.63279000	2.26706700	0.10657500
C	2.01500500	-4.13083000	-0.87313800
O	3.53126800	-0.88116500	0.80290400
O	3.62668100	-0.65423600	-1.42276700
H	-2.27271100	-0.90235300	-4.19677700
H	-2.41887300	-2.35258100	-3.13802900
H	-3.60828100	-1.02055000	-2.99533700
H	-2.84016500	5.00484800	-1.32839300
H	-3.84344000	3.83808900	-0.39689300
H	-3.58475000	3.58453300	-2.14260500
O	2.31749700	3.57380200	0.18609700
O	3.78564100	1.88969100	0.21696600
H	3.01092000	-4.41702600	-0.52056700
H	1.95641600	-4.22694400	-1.96560200
H	1.25035300	-4.77619400	-0.42010500
C	4.89539100	-1.26307500	0.79635800
C	3.40769600	4.45459300	0.37670900
H	5.50909500	-0.44422400	0.39678500
H	5.04538400	-2.15876100	0.17774400
H	5.16630000	-1.47159300	1.83597500
H	4.12580500	4.37921900	-0.45172400
H	3.93738700	4.23017300	1.31319700
H	2.98631200	5.46444000	0.41672200
C	-1.30687600	-0.52252500	1.43633300
C	-1.44788500	-2.04226500	1.70568000
C	-2.59037200	0.27833700	1.76223100
C	-0.17749600	-0.06729000	2.38251700
O	-0.48396600	-2.74553300	1.49295500
C	-2.67423700	-2.64125500	2.34613100
O	-3.69330700	-0.20614600	1.69702800
C	-2.44089500	1.70482000	2.26180100
H	0.71578100	-0.67619400	2.20341800
H	0.08112000	0.98668100	2.22292300
H	-0.48309700	-0.19710000	3.43232800
H	-2.44289700	-3.68276800	2.59717300
H	-2.96022000	-2.08277800	3.24779200
H	-3.51218300	-2.57508200	1.64518400
H	-2.16330900	1.67194800	3.32697400
H	-1.66274000	2.27612900	1.74186500
H	-3.41366000	2.20086500	2.16580500
N	0.27128500	1.95364900	-0.30224600

Py-5-32456-R3-Nu3

C	0.25809600	0.93363800	0.12272100
C	-1.17045700	1.49942400	0.11487500
C	0.16889100	-0.50623700	0.61731200

C	1.12851900	1.77059500	1.07441000
C	-1.36504400	2.96739900	0.24613400
C	1.37136300	-1.07708900	1.24885600
C	-0.97848600	-1.22898400	0.38384900
O	0.62010600	1.74446600	2.30591700
O	2.11537900	2.38386100	0.77304900
O	-2.61239200	3.36619800	-0.02644800
O	-0.49927600	3.74959100	0.57240400
C	-2.16815200	-0.57491000	-0.04831800
O	1.36435900	-2.41816900	1.31494200
O	2.31005200	-0.43409200	1.66717700
C	-1.06461400	-2.70559400	0.67557000
C	1.35288200	2.46915900	3.27673400
C	-2.84722400	4.75891600	0.09309500
C	-3.37796500	-1.33462300	-0.36240300
C	2.50605400	-3.01935600	1.90126300
O	-0.68909400	-3.41683100	-0.38836700
O	-1.43299500	-3.18317200	1.71273600
H	0.81628400	2.35344800	4.22351700
H	2.36992100	2.06180400	3.36111600
H	1.41040200	3.52978500	2.99615600
H	-3.89651800	4.91844000	-0.17386600
H	-2.19190600	5.32326500	-0.58416400
H	-2.66146200	5.09926900	1.12098800
O	-4.43817700	-0.56054700	-0.65221700
O	-3.45436800	-2.54930600	-0.39273600
H	2.32904300	-4.09940900	1.88217300
H	2.64100000	-2.67449600	2.93496700
H	3.40691100	-2.76795100	1.32399600
C	-0.81860800	-4.82205700	-0.25184400
C	-5.62700600	-1.25103600	-0.98742600
H	-1.86541800	-5.08467900	-0.04774500
H	-0.19072900	-5.18864900	0.57210000
H	-0.49328000	-5.25900100	-1.20087900
H	-5.95497800	-1.89569200	-0.16038100
H	-5.47982800	-1.87878100	-1.87741400
H	-6.38265300	-0.48495400	-1.18925200
C	0.89052300	0.96496300	-1.35328600
C	2.21913800	0.19886800	-1.42572600
C	1.09481400	2.36399500	-1.78392400
C	-0.10832000	0.38375200	-2.38018200
C	2.21225300	-1.19684900	-1.56321800
C	3.45362300	0.85343400	-1.34532300
N	1.19188800	3.42662500	-2.22397300
H	-1.01673400	0.99710700	-2.42483100
H	0.36031700	0.36637800	-3.37319000
H	-0.39609600	-0.63724800	-2.10714000
C	3.40664700	-1.91778200	-1.57910800
H	1.27161000	-1.74386600	-1.62498600
C	4.64680000	0.13430900	-1.37702400
H	3.47680700	1.93700800	-1.23569500
C	4.62963400	-1.25626300	-1.48284300
H	3.37475100	-3.00510600	-1.67049300

H	5.59693500	0.66693700	-1.31067500
H	5.56418000	-1.81961000	-1.49881800
N	-2.22581200	0.77108400	-0.04921100

Py-5-32456-R3-Nu2

C	1.12547900	-0.06352200	0.52452800
C	1.82314400	1.02610500	-0.29619200
C	0.00499600	0.64176500	1.29059900
C	2.08411600	-0.79483200	1.45787900
C	3.27790500	0.91751200	-0.58156500
C	-0.32959200	0.21916500	2.66569700
C	-0.70379600	1.59751500	0.60026000
O	2.79074200	0.05478800	2.18833700
O	2.16858900	-1.99229300	1.52143600
O	3.76186100	1.97590400	-1.23958100
O	3.97462200	-0.02360000	-0.27500600
C	-0.16292800	2.14630700	-0.60219700
O	-1.58854500	0.53372000	3.00934500
O	0.41233700	-0.34118000	3.43763100
C	-1.94725400	2.24388300	1.16018800
C	3.62969200	-0.53555700	3.16436200
C	5.13468400	1.90454200	-1.58597100
C	-0.92660100	3.11637400	-1.39053400
C	-1.99054600	0.14529200	4.31138700
O	-3.04723100	1.57361300	0.81634900
O	-1.95247300	3.24694400	1.82000400
H	4.14571400	0.28638200	3.66985700
H	3.01903400	-1.10235000	3.88036700
H	4.35676700	-1.20798900	2.68908800
H	5.36612900	2.83029200	-2.12162400
H	5.32923200	1.03494700	-2.22845100
H	5.75958700	1.81993000	-0.68639300
O	-0.22426300	3.66732300	-2.39289500
O	-2.09166800	3.40627000	-1.19570500
H	-3.03006900	0.46941500	4.42263600
H	-1.36014400	0.62202400	5.07378400
H	-1.91632100	-0.94493000	4.42127800
C	-4.26777400	2.16212600	1.23395400
C	-0.92406600	4.60915400	-3.18512100
H	-4.37328400	3.16094100	0.78934300
H	-4.29742500	2.25179200	2.32843900
H	-5.06783200	1.50202200	0.88475900
H	-1.26755700	5.45623800	-2.57542100
H	-1.79979500	4.14784300	-3.66229000
H	-0.21915800	4.95606100	-3.94735100
C	0.46190600	-1.05010400	-0.47866700
Br	1.62356600	-1.75434200	-1.85845700
S	-0.49864100	-2.46857400	0.23622400
H	-0.29416600	-0.47232700	-1.02476000
O	0.17692000	-3.73949600	0.01598000
O	-0.93513600	-2.10684600	1.58270500
C	-1.93834800	-2.42865400	-0.81882400

C	-2.07635700	-3.38910500	-1.81555400
C	-2.87729400	-1.41657000	-0.61645400
C	-3.19955500	-3.33289300	-2.64168900
H	-1.31464500	-4.16126700	-1.92991300
C	-3.98792000	-1.36908800	-1.45576800
H	-2.73622700	-0.67411000	0.17452600
C	-4.14795800	-2.32546900	-2.46255500
H	-3.33176600	-4.07662000	-3.42835000
H	-4.73094200	-0.58165100	-1.32310500
H	-5.02137000	-2.28274000	-3.11493300
N	1.14286400	1.95434000	-0.88577700

Py-5-32456-R3-Nu4

C	-0.86560000	-0.62442200	0.33568100
C	-1.00963000	-0.41131400	1.87531600
C	-1.17853700	0.68391100	-0.39613700
C	-2.24123500	0.34056400	2.33495300
H	-0.95141400	-1.40278000	2.34940100
H	-0.12303800	0.16572300	2.17612900
C	0.58425900	-0.95784300	-0.00952700
C	-1.78723600	-1.75232300	-0.13156900
C	-2.58504900	0.96169500	-0.76805900
C	-3.49944000	-0.43177400	2.62271600
C	-2.19857000	1.67398000	2.45282500
C	0.98765300	-2.36364300	-0.03944900
C	1.47566100	0.08721500	-0.10531100
O	-1.67107700	-1.94678600	-1.44961400
O	-2.51447200	-2.40662800	0.56629700
O	-2.78535300	2.19256600	-1.24934900
O	-3.49252600	0.16495600	-0.64471200
C	0.98983700	1.42020000	-0.21007600
H	-3.91245200	-0.83092500	1.68640800
H	-3.28933500	-1.29594800	3.27103100
H	-4.24994200	0.20746800	3.10815000
H	-1.28908600	2.23333800	2.21472100
H	-3.07476700	2.24469000	2.77241800
O	2.23955500	-2.56368700	-0.49264500
O	0.28777900	-3.29737100	0.29802100
C	2.96167100	-0.13122400	-0.23032000
C	-2.48089600	-2.97769500	-1.98336800
C	-4.12800200	2.51370200	-1.56733900
C	1.90524900	2.55441200	-0.13089200
C	2.71158900	-3.89841900	-0.45826900
O	3.50718500	-0.45035700	0.94454800
O	3.58560500	-0.02696000	-1.25109400
H	-2.27032300	-3.01770500	-3.05673800
H	-2.23508800	-3.93809500	-1.50958500
H	-3.54321100	-2.75497500	-1.81117600
H	-4.11809100	3.53854100	-1.95133300
H	-4.76467000	2.45044000	-0.67373600
H	-4.52546400	1.82726600	-2.32726000
O	1.34269000	3.73421600	-0.45137100

O	3.07022000	2.48312500	0.21637400
H	3.72893400	-3.87620800	-0.86178300
H	2.07537900	-4.55088200	-1.07092700
H	2.71901500	-4.28351900	0.57080200
C	4.91024800	-0.64874400	0.92463900
C	2.19076600	4.86185700	-0.34891000
H	5.41885500	0.26355100	0.58440700
H	5.17280200	-1.47405900	0.24798800
H	5.20625600	-0.88991500	1.95023200
H	3.05331800	4.76918400	-1.02350900
H	2.56706500	4.97997900	0.67691900
H	1.58640200	5.73061300	-0.62973300
N	-0.31348700	1.63411600	-0.51226800

Py-5-32456-R4

C	0.00000000	0.00000000	1.12703600
C	0.00000000	1.20158700	0.42264800
C	0.00000000	-1.20158700	0.42264800
C	0.00000000	1.12910900	-0.96852500
C	0.00000000	-1.12910900	-0.96852500
N	0.00000000	0.00000000	-1.62998200
F	0.00000000	-2.25233000	-1.65680100
F	0.00000000	-2.35659800	1.05941100
F	0.00000000	0.00000000	2.43902200
F	0.00000000	2.35659800	1.05941100
F	0.00000000	2.25233000	-1.65680100

Py-5-32456-R4-Nu1

C	0.12897000	-0.19628900	-0.34289400
C	0.83645300	-1.40703000	0.11256400
C	1.07370600	0.88718500	-0.10483000
C	2.44233300	0.70856000	-0.03472600
C	2.94647600	-0.57938100	0.09235700
F	0.60667300	2.16290600	-0.16271000
F	3.25152900	1.77538500	-0.02255300
F	4.27655200	-0.80152500	0.19519600
F	0.06032600	-2.48495800	0.40526600
F	-0.17034800	-0.24640000	-1.78612200
C	-1.28693600	-0.00516900	0.30552400
C	-2.31313500	-1.06059800	-0.17855100
C	-1.91517200	1.33251800	-0.16281700
C	-1.15914200	-0.07987100	1.83331600
O	-2.11735400	-1.85540400	-1.05637200
C	-3.64261500	-1.04371700	0.55666200
O	-2.39871800	1.41308200	-1.26612600
C	-2.01900500	2.48102900	0.81468800
H	-0.86552700	-1.09599600	2.13042400
H	-0.38107400	0.60992300	2.18499400
H	-2.09978400	0.17355100	2.33937200
H	-3.52953100	-1.58199200	1.50975100
H	-3.98604300	-0.02553600	0.78662800

H	-4.38876800	-1.56475600	-0.05349200
H	-2.85649400	2.27925900	1.50111800
H	-1.11136100	2.60522900	1.41320100
H	-2.23835600	3.39712200	0.25482800
N	2.11590400	-1.59004700	0.21481600

Py-5-32456-R4-Nu3

C	0.50948600	0.92019500	0.15420800
C	0.73709800	0.57761300	-1.24482100
C	1.37753700	0.06161500	0.92148200
C	2.48545200	-0.57507700	0.38230300
C	2.66125700	-0.57908500	-0.99207500
F	1.20966200	-0.04554400	2.26218600
F	3.32965200	-1.22162000	1.19227300
F	3.71687800	-1.19869800	-1.55603200
F	-0.20453900	1.02404800	-2.11294600
F	0.89874000	2.40606200	0.39223900
C	-1.00420000	1.02229300	0.57099100
C	-1.73180600	-0.28369300	0.24756600
C	-1.61132200	2.14673200	-0.17227600
C	-1.18096800	1.41246600	2.05825300
C	-1.43036300	-1.42982100	0.99705200
C	-2.66466100	-0.38381900	-0.78734400
N	-2.15046200	3.03570800	-0.67233700
H	-0.55995200	2.29034400	2.27003300
H	-2.23551900	1.65529900	2.24545700
H	-0.88475300	0.59464000	2.71977300
C	-2.05734500	-2.64247800	0.72255200
H	-0.69224600	-1.37505400	1.79908600
C	-3.29362500	-1.59996900	-1.06228500
H	-2.90169400	0.49387700	-1.38991700
C	-2.99472700	-2.73196300	-0.30841100
H	-1.80936400	-3.52369600	1.31626000
H	-4.02066600	-1.65759700	-1.87389100
H	-3.48571100	-3.68221400	-0.52390100
N	1.75410200	-0.03054700	-1.76845700

Py-5-32456-R4-Nu2

C	1.44987000	0.15370800	-0.48059900
C	2.05544500	-1.17625500	-0.59852000
C	2.28095200	0.89350300	0.45500300
C	3.54323700	0.50086900	0.85824800
C	4.00314100	-0.75678000	0.49384800
F	1.79939800	2.07028500	0.91570100
F	4.26736600	1.30879000	1.63827600
F	5.23073400	-1.18152800	0.85786400
F	1.36294800	-2.05327900	-1.36002800
F	1.42993400	0.75352100	-1.84118900
C	-0.05186400	0.27289600	-0.03861300
Br	-0.93030700	1.87520100	-0.70824700
S	-1.13019100	-1.17081600	-0.38595500

H	-0.08856600	0.34989000	1.05425100
O	-1.31623500	-1.31979100	-1.82299600
O	-0.61781700	-2.26134500	0.44091200
C	-2.70223100	-0.70325400	0.32019100
C	-3.71744800	-0.25182100	-0.51997600
C	-2.88717300	-0.85428300	1.69316100
C	-4.94984900	0.07713500	0.04044900
H	-3.53183700	-0.16512400	-1.59108700
C	-4.12247000	-0.51419800	2.24189800
H	-2.07983800	-1.24544500	2.31424000
C	-5.14800400	-0.04883700	1.41701300
H	-5.75848500	0.43266200	-0.59907900
H	-4.28735500	-0.62241200	3.31440800
H	-6.11428700	0.21257100	1.85076400
N	3.23450200	-1.54974800	-0.21587500

Py-5-32456-R4-Nu4

C	-0.39473700	-0.15153500	0.82570800
C	-1.91233700	0.04768200	0.77181100
C	0.39732600	0.95242700	0.34215100
C	-2.46744000	0.29142600	-0.61048700
H	-2.36693600	-0.85013100	1.21607900
H	-2.13976600	0.89912000	1.42543000
C	0.15328200	-1.35326400	0.21989500
C	1.67959900	0.79364300	-0.15338000
C	-2.47118500	-0.86905700	-1.56891900
C	-2.93430000	1.49506500	-0.95847300
C	2.13725800	-0.49069500	-0.42577000
H	-2.94119000	-1.75198000	-1.11014000
H	-3.00445700	-0.61780100	-2.49537500
H	-1.44190100	-1.16141200	-1.82819700
H	-2.91166400	2.33291100	-0.25756800
H	-3.34373400	1.67894700	-1.95524300
F	-0.64649300	-2.45245800	0.27718200
F	3.37765800	-0.68778200	-0.92601800
F	2.43027900	1.86733200	-0.43304700
F	-0.08033700	2.20678800	0.51178600
F	-0.23432500	-0.28199200	2.37329800
N	1.34340200	-1.52725600	-0.26843000

Py-5-32456-R5

C	0.00000000	1.19990300	0.24807200
C	0.00000000	0.00000000	0.94578600
C	-0.01951100	1.12530400	-1.14338700
C	0.00000000	-1.19990300	0.24807200
C	0.01951100	-1.12530400	-1.14338700
N	0.12317000	-2.36534300	-1.96961400
O	-0.38841200	-2.33422700	-3.04814300
O	0.73878900	-3.26900500	-1.46521500
N	-0.01687700	-2.48980300	0.97654900
O	-0.94986500	-3.20378600	0.74852600

O	0.90020800	-2.67505100	1.72589800
N	0.00000000	0.00000000	2.43005400
O	-0.76939300	-0.75637500	2.95207700
O	0.76939300	0.75637500	2.95207700
N	0.01687700	2.48980300	0.97654900
O	-0.90020800	2.67505100	1.72589800
O	0.94986500	3.20378600	0.74852600
N	-0.12317000	2.36534300	-1.96961400
O	-0.73878900	3.26900500	-1.46521500
O	0.38841200	2.33422700	-3.04814300
N	0.00000000	0.00000000	-1.80695300

Py-5-32456-R5-Nu1

C	0.63438700	-0.01579600	0.35558100
C	-0.63777600	-0.85313500	0.45663800
C	0.14334700	1.41488000	0.54503200
C	-1.84491100	-0.33740700	0.06023300
C	-1.99000000	1.05551900	-0.13717700
N	-3.21988300	1.68115600	-0.56452800
O	-3.27061400	2.88762500	-0.58556400
O	-4.13157100	0.94803100	-0.89945600
N	-3.03175800	-1.21031600	-0.12213100
O	-3.77288300	-1.32284100	0.81355700
O	-3.14889100	-1.70328400	-1.20917600
N	-0.57337100	-2.22484200	0.94031400
O	-1.48074000	-2.97247000	0.66122400
O	0.37413500	-2.53712400	1.62270700
N	1.54157100	-0.34096500	1.58166000
O	1.10653600	-0.01705800	2.65262900
O	2.59089000	-0.87702500	1.37017200
N	1.08988700	2.42834100	1.03850300
O	2.24474100	2.07149300	1.18024900
O	0.68336200	3.53842100	1.25417500
C	1.45104400	-0.24069800	-0.98755400
C	1.90434200	-1.72639100	-1.10541900
C	2.61269000	0.78600000	-1.13657200
C	0.47206900	-0.05005900	-2.16896600
O	1.03595400	-2.56529500	-1.04409900
C	3.32067100	-2.12273800	-1.41515300
O	3.74997600	0.50252300	-0.86676500
C	2.29751300	2.13514800	-1.75125500
H	-0.29894100	-0.82875700	-2.14348500
H	-0.01137600	0.93525300	-2.14962600
H	1.01754200	-0.14193600	-3.11892700
H	3.32647900	-3.20263200	-1.59902300
H	3.69317300	-1.58026700	-2.29443700
H	3.97265200	-1.85662200	-0.57652400
H	2.28327400	2.01828500	-2.84590800
H	1.32399300	2.55133200	-1.46390800
H	3.10237500	2.82742000	-1.48119500
N	-1.01167400	1.86405500	0.23811100

Py-5-32456-R5-Nu3

C	-1.20288400	0.37909800	-0.08018600
C	-0.85225000	-0.60044700	-1.20219400
C	-0.07651900	1.39107500	-0.10359000
C	1.19292700	1.00662600	-0.46909100
C	1.38342200	-0.22120900	-1.11724200
N	2.35510700	1.89406500	-0.22371500
O	2.67353500	2.04374400	0.92525600
O	2.88218200	2.37027100	-1.18912500
N	2.68362000	-0.81944600	-1.30699500
O	3.61642800	-0.30675100	-0.71700400
O	2.76952600	-1.79808500	-2.00833200
N	-1.92363000	-1.42202000	-1.80852300
O	-1.60243200	-2.40034500	-2.42920100
O	-3.06316900	-1.04418300	-1.64064700
N	-2.50605400	1.13351900	-0.48128500
O	-2.43452900	1.76177700	-1.50285700
O	-3.46499300	1.03491600	0.22653600
N	-0.33661300	2.75196000	0.28522600
O	-1.39544700	2.99398300	0.83237500
O	0.50357100	3.59001900	0.03816000
C	-1.35645700	-0.36446600	1.32033600
C	-0.06068000	-1.17866000	1.52920600
C	-2.48667200	-1.31544400	1.27392500
C	-1.60317400	0.59278200	2.50228500
C	1.05399200	-0.58588500	2.13491600
C	0.06434700	-2.47295100	1.01173200
N	-3.33415800	-2.09644600	1.30807500
H	-2.56754500	1.09738600	2.40074600
H	-1.58403400	0.00556500	3.42959700
H	-0.82064000	1.35599000	2.55725700
C	2.27753700	-1.25225700	2.17479000
H	1.00003000	0.42478900	2.53740000
C	1.28753800	-3.14011200	1.05187800
H	-0.79054300	-2.95903700	0.53720100
C	2.40113600	-2.52722100	1.62483500
H	3.14088900	-0.75893200	2.62264600
H	1.36865200	-4.13929200	0.62258500
H	3.36332000	-3.04091700	1.64038500
N	0.33014400	-0.90076100	-1.56402800

Py-5-32456-R5-Nu2

C	0.65983900	-0.93421700	0.33120900
C	1.09653000	-0.33566900	1.66834600
C	1.80028400	-0.58206900	-0.60781100
C	2.41445100	0.64262900	-0.46974500
C	2.24490500	1.36758700	0.71885000
N	3.32334500	1.16959600	-1.51794300
O	2.79878900	1.55952500	-2.52412500
O	4.49371400	1.17609800	-1.25764200
N	2.75930200	2.70664200	0.89915700
O	3.05930500	3.31799700	-0.10899100

O	2.83678000	3.14552600	2.02003700
N	0.62330100	-0.95883500	2.92087700
O	1.02549600	-0.51234500	3.95870700
O	-0.15308700	-1.88192600	2.80227500
N	0.58503300	-2.46951600	0.47645500
O	1.59424400	-2.98751000	0.87632700
O	-0.44107300	-3.00847900	0.19680500
N	2.18551000	-1.48086100	-1.66713200
O	1.49942200	-2.46005600	-1.86570700
O	3.18753000	-1.21502400	-2.29669100
C	-0.67107200	-0.30460100	-0.15910200
Br	-1.22668900	-0.86050100	-1.91768900
S	-2.10617800	-0.27929600	1.01334000
H	-0.45294200	0.76993400	-0.25066200
O	-2.72365200	-1.58635500	1.13685400
O	-1.59830300	0.43832000	2.18264900
C	-3.24490400	0.80262700	0.17937400
C	-4.31354600	0.24468600	-0.51940400
C	-3.05606700	2.17987000	0.28444900
C	-5.21375600	1.10208600	-1.14769700
H	-4.42880800	-0.83904600	-0.56099200
C	-3.96218400	3.02378800	-0.35447700
H	-2.22529900	2.57924400	0.86846700
C	-5.03430800	2.48463400	-1.06793600
H	-6.05861200	0.68969700	-1.69993700
H	-3.83553000	4.10455800	-0.28674100
H	-5.74215400	3.15063100	-1.56325800
N	1.72664500	0.76111400	1.78836900

Py-5-32456-R5-Nu4

C	-1.09687700	-0.48350000	-0.16317100
C	-1.85271100	-0.90269500	1.11600900
C	0.33217000	-0.97214200	-0.17849400
C	-1.27801500	-0.20727600	2.33508100
H	-2.91353500	-0.65961500	0.98542100
H	-1.77791500	-1.99340000	1.20919700
C	-1.03340200	1.01415200	-0.37592600
C	1.41468100	-0.13344600	-0.18232600
C	-1.97259300	1.04988500	2.78539500
C	-0.19542400	-0.68655900	2.95597600
C	1.22636800	1.26011400	-0.28642800
H	-3.03819200	0.85334900	2.98009300
H	-1.51543300	1.45112200	3.69879000
H	-1.92801200	1.83864500	2.01693400
H	0.30427000	-1.60071800	2.62266200
H	0.23099200	-0.17482500	3.82169300
N	2.31769500	2.19995100	-0.37157800
O	2.06145500	3.38005700	-0.34848200
O	3.44231300	1.74192500	-0.46472700
N	2.78507300	-0.68386400	-0.04874700
O	3.28573400	-1.13219200	-1.04202900
O	3.26651000	-0.62440200	1.04807500

N	0.50990900	-2.39881400	-0.05929800
O	1.54530700	-2.83212000	0.40002900
O	-0.41246400	-3.09579400	-0.43222600
N	-1.83713100	-1.09523700	-1.41179200
O	-1.35778300	-0.80457000	-2.47873500
O	-2.79795600	-1.78459300	-1.23386600
N	-2.32016800	1.71367100	-0.52293700
O	-3.29064400	1.01114000	-0.72989300
O	-2.34427900	2.91426100	-0.43748100
N	-0.00007900	1.75922900	-0.38804900

Py-5-42356-R1

C	0.96456400	-0.03902100	0.00000000
C	0.25864700	0.03686100	1.21094000
C	0.25864700	0.03686100	-1.21094000
C	-1.14122200	-0.01937500	1.14367700
C	-1.14122200	-0.01937500	-1.14367700
C	2.46919200	-0.36924100	0.00000000
C	0.96800900	0.27601200	2.54793300
C	0.96800900	0.27601200	-2.54793300
C	-2.06455500	-0.08669400	2.36964300
C	-2.06455500	-0.08669400	-2.36964300
F	1.21938200	-0.84140200	-3.20788000
F	2.11152000	0.92607800	-2.35763300
F	0.21347100	1.05588000	-3.31596500
F	-2.34194200	1.12381400	-2.83619400
F	-1.50864300	-0.80361800	-3.34297100
F	-3.20534200	-0.66822100	-2.04291400
F	-3.20534200	-0.66822100	2.04291400
F	-1.50864300	-0.80361800	3.34297100
F	-2.34194200	1.12381400	2.83619400
F	0.21347100	1.05588000	3.31596500
F	1.21938200	-0.84140200	3.20788000
F	2.11152000	0.92607800	2.35763300
F	2.75956900	-1.11083200	1.06443300
F	2.75956900	-1.11083200	-1.06443300
F	3.24886700	0.69563000	0.00000000
N	-1.79388400	-0.08047500	0.00000000

Py-5-42356-R1-Nu1

C	-0.13121800	-0.59279900	0.28640200
C	-1.22932600	0.49334900	0.14455400
C	1.24427300	0.10953600	0.19920600
C	-0.90326300	1.79535400	-0.14484400
C	1.34057100	1.44306600	-0.12573200
C	-1.93515200	2.91951100	-0.32406400
C	-2.68503900	0.10829700	0.24759300
C	2.68111600	2.17530200	-0.29943500
C	2.47602900	-0.64563900	0.63288900
C	-0.26532900	-1.09764900	1.77349200
F	0.07230400	-0.07269400	2.57455400

F	-1.50879000	-1.42035500	2.12470100
F	0.52313300	-2.10694000	2.11704900
F	2.35079600	-1.97620200	0.51719600
F	2.79784900	-0.41956700	1.91662600
F	3.56834000	-0.34565000	-0.08432700
F	2.50941800	3.48628200	-0.41212000
F	3.50434600	1.99513900	0.73844000
F	3.31983800	1.78321800	-1.40971000
F	-2.42580300	2.93705900	-1.56809400
F	-2.96542700	2.83734500	0.52116000
F	-1.38367500	4.11088000	-0.11554300
F	-3.26090700	0.41257400	1.41598400
F	-3.42962300	0.70428100	-0.70397400
F	-2.89970100	-1.20446500	0.05996300
C	-0.31220500	-1.70015500	-0.90171900
C	-1.20777600	-2.91215200	-0.54161900
C	0.96300100	-2.41927500	-1.45027400
C	-0.82611800	-0.94533500	-2.14891400
O	-1.14663500	-3.43627500	0.53827600
C	-2.08159200	-3.48189400	-1.63632100
O	1.05940900	-3.62021300	-1.37429000
C	1.96465200	-1.64972300	-2.28761900
H	-1.86623000	-0.62133100	-2.04095600
H	-0.21488200	-0.04926300	-2.32251500
H	-0.76523400	-1.57909900	-3.04413300
H	-2.82450900	-2.74628200	-1.97027600
H	-1.46372600	-3.76155700	-2.50126200
H	-2.58771200	-4.37092000	-1.24436400
H	1.67879400	-1.78390100	-3.34180200
H	2.01137500	-0.57821000	-2.07624600
H	2.94924900	-2.10871700	-2.14169400
N	0.32515100	2.28060900	-0.34247900

Py-5-42356-R1-Nu3

C	0.26094700	0.99738800	-0.19196900
C	-0.90284800	0.19027200	-0.80579100
C	1.50975700	0.09478000	-0.21233900
C	-0.79879500	-1.17410200	-0.95305800
C	1.36892200	-1.27502500	-0.30644100
C	-1.91097700	-2.07079800	-1.51629200
C	-2.24344400	0.84258000	-1.06642500
C	2.49344900	-2.26603100	0.03282000
C	2.90722400	0.66179100	-0.15243500
C	0.54063700	2.26560200	-1.08802000
F	1.47314100	2.00059500	-2.00584400
F	-0.52613400	2.66920700	-1.77659800
F	0.94815400	3.32885600	-0.39174700
F	2.95130500	1.98014800	0.06775900
F	3.59700400	0.46208600	-1.28519800
F	3.65151100	0.11652300	0.83106800
F	2.16659400	-3.50805600	-0.29865600
F	3.65177500	-2.00685700	-0.57565200

F	2.72554700	-2.27227700	1.35549000
F	-2.76493700	-2.45678200	-0.56445800
F	-2.61392500	-1.49285000	-2.49379800
F	-1.40055600	-3.18178400	-2.04155100
F	-2.48260700	1.13250500	-2.35224100
F	-3.26454000	0.06662200	-0.66739500
F	-2.40208100	1.98635400	-0.38684400
C	-0.07966900	1.44585800	1.36381400
C	-0.91233800	0.32632000	2.00598500
C	-0.81778100	2.71934500	1.47461900
C	1.18186800	1.69329600	2.22249600
C	-0.28377000	-0.86346700	2.39945300
C	-2.29295400	0.44490400	2.19644400
N	-1.33514300	3.73346700	1.66250300
H	1.72016000	2.58393400	1.88469600
H	0.86608500	1.83978300	3.26377800
H	1.85824000	0.83196500	2.19087000
C	-1.02497200	-1.91856200	2.92649900
H	0.79256100	-0.98389200	2.28455000
C	-3.03344600	-0.60821500	2.73306400
H	-2.80357400	1.36498800	1.91135300
C	-2.40479100	-1.79847300	3.09078000
H	-0.51534600	-2.84081900	3.20962800
H	-4.11046400	-0.49393200	2.86484400
H	-2.98450800	-2.62616800	3.50204800
N	0.25549500	-1.92478400	-0.62821600

Py-5-42356-R1-Nu2

C	0.39372800	0.00161300	0.68735300
C	0.89201200	1.40129700	0.30674300
C	1.40382400	-1.04227400	0.19751800
C	2.07005000	1.55795400	-0.39441100
C	2.55144700	-0.66057500	-0.44567600
C	2.58384400	2.92265000	-0.87807000
C	0.08414200	2.61219300	0.71162900
C	3.61458900	-1.66074500	-0.91727800
C	1.13102300	-2.48901500	0.51864500
C	0.36913700	-0.10267100	2.25336900
F	1.63155300	-0.17464200	2.68494900
F	-0.16592700	0.94877900	2.86069400
F	-0.25656900	-1.19116100	2.69775300
F	-0.16661400	-2.72198300	0.72521000
F	1.76269600	-2.90763100	1.62834400
F	1.49794800	-3.31817100	-0.46585600
F	4.77213100	-1.05399900	-1.15505500
F	3.86850200	-2.60970600	-0.01131700
F	3.25653700	-2.26456500	-2.05394000
F	1.94475400	3.31555200	-1.98415600
F	2.44977800	3.88877500	0.03438600
F	3.87596000	2.87256800	-1.17898300
F	0.59545400	3.27790600	1.75450800
F	-0.02997600	3.50251200	-0.28654100

F	-1.18354000	2.31746800	1.05756600
C	-1.04629300	-0.18216300	-0.01262900
Br	-0.96016700	-1.12068300	-1.70712400
S	-2.56381000	-0.73806700	0.89882600
H	-1.37968700	0.80871800	-0.31863600
O	-2.68701400	-2.18564900	0.97462300
O	-2.62047400	0.07486800	2.10932400
C	-3.84630000	-0.14185700	-0.19146000
C	-4.52224400	-1.04839500	-1.00373300
C	-4.16355800	1.21552900	-0.16414900
C	-5.54120700	-0.57274700	-1.82719000
H	-4.24691300	-2.10338300	-0.98089300
C	-5.18000100	1.67772100	-0.99782200
H	-3.62507800	1.89096700	0.50320000
C	-5.86449000	0.78531300	-1.82556000
H	-6.08449600	-1.26484500	-2.47142700
H	-5.44339600	2.73586200	-0.99528000
H	-6.66136300	1.15226200	-2.47395600
N	2.89646500	0.58876700	-0.77688500

Py-5-42356-R1-Nu4

C	-0.03999800	-0.84021700	0.07309200
C	-0.10048700	-0.99285700	1.63960600
C	1.33616700	-0.24143500	-0.25470200
C	-1.36832000	-1.52439200	2.26795200
H	0.09188200	0.01860600	2.02207200
H	0.76003300	-1.61545500	1.92634900
C	-1.05470000	0.20102500	-0.42542100
C	1.46846600	1.07600300	0.13722400
C	-1.35970400	-2.96640500	2.70144100
C	-2.42376500	-0.72919400	2.47012800
C	-0.76770500	1.48429000	-0.01306900
N	0.44042600	1.89518300	0.39409700
H	-2.33153800	-3.26780500	3.11444800
H	-0.59012700	-3.12265200	3.47474500
H	-1.10986300	-3.63517600	1.86588400
H	-2.41036700	0.32215400	2.17389000
H	-3.34417500	-1.11574200	2.91548500
C	-1.78968700	2.61872200	0.06402000
C	-2.32660600	-0.10238400	-1.16603200
C	-0.27108100	-2.22732900	-0.53312100
C	2.53800000	-1.08699200	-0.55252300
C	2.80726300	1.78807700	0.35214200
F	-3.01175300	2.18236300	0.39982000
F	-1.90221400	3.30333300	-1.07699400
F	-1.44423800	3.49869700	1.00221400
F	-3.32973700	-0.55902400	-0.40021900
F	-2.79950300	0.99101100	-1.78605300
F	-2.16997100	-1.00965400	-2.13998200
F	-1.52052800	-2.64964700	-0.29397900
F	-0.08323100	-2.29968400	-1.84544500
F	0.53736300	-3.13684600	0.03463100

F	2.32432400	-2.04690900	-1.45939200
F	3.00756900	-1.72210300	0.54240300
F	3.55848500	-0.37002800	-1.03932900
F	3.38469700	2.16289900	-0.79298900
F	2.65012100	2.89372000	1.07475300
F	3.68351800	1.02946400	1.02322900

Py-5-42356-R2

C	0.00000000	0.00000000	1.02001000
C	0.00000000	1.20670400	0.30565300
C	0.00000000	-1.20670400	0.30565300
C	0.00000000	1.14017600	-1.10217300
C	0.00000000	-1.14017600	-1.10217300
N	0.00000000	0.00000000	-1.77552500
C	0.00000000	-2.47159000	0.98549000
N	0.00000000	-3.48879000	1.52843700
C	0.00000000	-2.36285900	-1.88012600
N	0.00000000	-3.34205900	-2.48756600
C	0.00000000	0.00000000	2.45862000
N	0.00000000	0.00000000	3.61122500
C	0.00000000	2.47159000	0.98549000
N	0.00000000	3.48879000	1.52843700
C	0.00000000	2.36285900	-1.88012600
N	0.00000000	3.34205900	-2.48756600

Py-5-42356-R2-Nu1

C	-0.14874600	-0.33677700	-0.70647100
C	1.37731200	-0.44610500	-0.70170700
C	-0.43395100	1.16240400	-0.69700400
C	2.09455500	0.48751200	0.02778700
C	0.41391300	1.98139100	0.02346500
C	3.50714700	0.24004900	0.27648000
N	4.62292600	0.03675900	0.48887100
C	2.02899500	-1.53522100	-1.34199700
N	2.54818900	-2.43370600	-1.85877700
C	0.02757800	3.36467500	0.25123400
N	-0.27095800	4.46255400	0.44316300
C	-1.53850500	1.73043300	-1.39644900
N	-2.39385300	2.24439300	-1.98372900
C	-0.65507600	-0.94698800	-1.95521300
N	-0.98100900	-1.45284700	-2.93836700
C	-0.78792300	-1.12854500	0.52167400
C	-2.32528600	-1.04346100	0.46538000
C	-0.37591900	-0.43373400	1.83749100
C	-0.37627000	-2.60631400	0.48612600
O	-2.89109100	-0.49073500	-0.44258600
C	-3.09407500	-1.75224700	1.55575700
O	-1.03537900	0.50345200	2.22124600
C	0.77997700	-0.97430100	2.63768700
H	-0.74626900	-3.08838000	-0.43059900
H	0.71437400	-2.72185200	0.51497700

H	-0.79056000	-3.14633900	1.34803100
H	-3.26277900	-2.79107100	1.23270100
H	-2.56044500	-1.77253200	2.51380800
H	-4.06957700	-1.26702200	1.67340200
H	0.43049900	-1.86361100	3.18647100
H	1.61938200	-1.29280300	2.00816400
H	1.09989400	-0.21422100	3.35869900
N	1.61968800	1.63991400	0.51450000

Py-5-42356-R2-Nu3

C	-0.91991300	0.82059200	-0.11291800
C	-1.16386000	-0.37630700	-1.02033200
C	0.31183000	1.52444500	-0.66120400
C	-0.08111300	-0.98299300	-1.63179900
C	1.28369500	0.77131400	-1.30030100
C	-0.28583400	-2.26476500	-2.28978300
N	-0.43743900	-3.28771700	-2.80122600
C	-2.47682600	-0.90675900	-1.17327700
N	-3.54629200	-1.32877500	-1.31596100
C	2.56307200	1.40180400	-1.59187900
N	3.58315600	1.89446800	-1.81029800
C	0.49468400	2.91801100	-0.44296400
N	0.63221500	4.05340100	-0.25431100
C	-2.08463700	1.73060100	-0.16244600
N	-2.99241100	2.44059300	-0.18900400
C	-0.71110100	0.38510300	1.43190800
C	0.47143700	-0.57838400	1.52562500
C	-1.94421000	-0.30258400	1.86023300
C	-0.54715500	1.62672000	2.32523500
C	1.77051100	-0.08755100	1.70406100
C	0.28741600	-1.95667400	1.35795900
N	-2.91992000	-0.81149100	2.20608700
H	-1.45673300	2.24108400	2.30260900
H	-0.35606300	1.31273000	3.35938200
H	0.29291000	2.24555500	1.98957300
C	2.86296100	-0.95320300	1.68068400
H	1.94554000	0.97902900	1.84602600
C	1.37951700	-2.82200700	1.33759400
H	-0.71964700	-2.36012300	1.23276500
C	2.67166600	-2.32092300	1.48979400
H	3.86900300	-0.55218300	1.81056700
H	1.21677300	-3.89151800	1.19830800
H	3.52812600	-2.99615000	1.46727200
N	1.16458500	-0.49434400	-1.71580700

Py-5-42356-R2-Nu2

C	1.02825700	-0.03509100	0.03954600
C	1.85829000	1.21391200	-0.24556500
C	2.07111300	-1.13782200	-0.13870300
C	3.05120100	1.29925100	0.45620600
C	3.25342400	-0.93177000	0.56771900

C	3.76241200	2.56367900	0.50399300
N	4.32900600	3.56837100	0.54234200
C	1.48581600	2.20481100	-1.19424100
N	1.22723900	2.99481900	-2.00091600
C	4.17334100	-2.04066600	0.75176800
N	4.90514600	-2.92046900	0.90100500
C	1.92661900	-2.28790600	-0.96108200
N	1.88611600	-3.22402500	-1.64297900
C	0.58692000	-0.04564700	1.46803900
N	0.27000700	-0.09559500	2.57568600
C	-0.22439500	-0.15970900	-0.84623000
Br	-1.13132400	-1.84892900	-0.62005900
S	-1.44182100	1.25961700	-0.58078200
H	0.03584600	-0.06175700	-1.90854100
O	-0.82573500	2.18469700	0.36624500
O	-1.79301200	1.67673200	-1.93187400
C	-2.90376000	0.61093900	0.19342500
C	-2.96786500	0.56697000	1.58463900
C	-3.96903300	0.22877900	-0.61989400
C	-4.13921000	0.09660900	2.17567900
H	-2.11844100	0.89115000	2.18703100
C	-5.13329700	-0.23362800	-0.01254400
H	-3.87866900	0.29743600	-1.70444700
C	-5.21403400	-0.30312900	1.38006900
H	-4.21321000	0.04890900	3.26239400
H	-5.98058600	-0.53809400	-0.62755100
H	-6.12817300	-0.66784500	1.85053400
N	3.67161400	0.25403200	1.03103900

Py-5-42356-R2-Nu4

C	-0.11112900	-0.66536100	-0.17668800
C	-0.67665100	-1.16254800	1.19297500
C	1.41570600	-0.65510200	-0.12644400
C	-2.16641800	-1.00855300	1.38010900
H	-0.14911000	-0.57244800	1.95577300
H	-0.37386500	-2.21709200	1.31252700
C	-0.54554900	0.76397100	-0.48679700
C	2.07518300	0.51018900	0.21447600
C	-3.07725200	-1.89986600	0.57773600
C	-2.63964900	-0.09528000	2.23470100
C	0.26849100	1.81591000	-0.11710600
N	1.53998500	1.73664700	0.30794700
H	-4.10333300	-1.85955900	0.96559100
H	-2.72830300	-2.94349000	0.59848800
H	-3.10537300	-1.59087700	-0.47851900
H	-1.96744700	0.55361700	2.80154100
H	-3.71386800	0.02925800	2.39120900
C	-0.54489600	-1.57220300	-1.26460100
N	-0.86450700	-2.29204500	-2.10704600
C	-1.83427800	0.98425500	-1.04789400
N	-2.88117400	1.12694600	-1.52474100
C	-0.25806700	3.16872400	-0.23339700

N	-0.67102100	4.24256200	-0.31978100
C	2.11308400	-1.88642900	-0.26994900
N	2.65855400	-2.90214700	-0.39048400
C	3.50856000	0.44328100	0.46326600
N	4.64366100	0.39584700	0.66425700

Py-5-42356-R3

C	-0.07721900	-0.98843700	0.03978000
C	1.20812500	-0.45483300	-0.06044300
C	-1.17342300	-0.12797400	0.11660600
C	1.33338600	0.94061400	-0.11250800
C	-0.92102000	1.25194700	0.10263800
N	0.29574000	1.75990600	-0.02273900
C	-2.06992800	2.21426800	0.29326100
O	-3.12344100	1.87677100	0.76970800
O	-1.78317500	3.43795100	-0.10232400
C	-2.56657600	-0.68348600	0.29008400
O	-2.91169200	-1.27958400	1.27204600
O	-3.31324800	-0.45368400	-0.77469100
C	-0.25027200	-2.48333300	0.11065600
O	0.48390400	-3.19903200	0.73433500
O	-1.29398400	-2.88601900	-0.59345800
C	2.40160000	-1.37405100	-0.17156300
O	2.59895200	-2.07254100	-1.12566900
O	3.15360000	-1.31622600	0.91250800
C	2.69820800	1.55144000	-0.32682000
O	3.63354300	0.91528900	-0.74045200
O	2.73735900	2.83384600	-0.02649400
C	-2.81239500	4.40889100	0.07889400
H	-3.07423600	4.48813400	1.14156700
H	-2.40627900	5.35438800	-0.28930100
H	-3.70569100	4.12371100	-0.49116700
C	-4.68117600	-0.83791200	-0.66289300
H	-5.15584800	-0.27160800	0.14919100
H	-5.14641900	-0.59779100	-1.62247400
H	-4.75956100	-1.91220800	-0.45328400
C	-1.61192200	-4.27610100	-0.51514000
H	-1.85997100	-4.54154400	0.52041400
H	-2.47421600	-4.42709400	-1.16933600
H	-0.75834800	-4.87609300	-0.85289800
C	4.35881900	-2.07596000	0.86696300
H	4.84947700	-1.92985000	1.83278200
H	4.13245200	-3.13726000	0.70507600
H	4.99457300	-1.70832000	0.05074500
C	3.98914700	3.48501100	-0.23515300
H	4.27997600	3.41492900	-1.29092900
H	3.84106500	4.52823900	0.05507700
H	4.76618600	3.01835400	0.38346600

Py-5-42356-R3-Nu1

C	-0.05091700	-0.72655500	0.09700400

C	1.21684000	0.09875400	0.24289600
C	-1.20650500	0.24984700	0.33511700
C	-0.10623300	-1.82956300	1.16727800
C	2.43186500	-0.56269100	0.68138400
C	1.20112000	1.43790800	-0.11796400
C	-2.49747100	-0.25229400	0.84504100
C	-1.04451900	1.57531300	0.03901500
O	0.16076400	-1.35184700	2.37975900
O	-0.38844500	-2.97941400	0.95703500
O	3.44444700	0.28698300	0.96358800
O	2.57171800	-1.76471800	0.80565900
C	2.47575500	2.17270100	-0.45760800
O	-3.54589900	0.43110400	0.35840100
O	-2.64047400	-1.15978100	1.63218600
C	-2.16350800	2.57731600	0.18553100
C	0.03477400	-2.28335000	3.43924600
C	4.68948700	-0.31143000	1.27519500
O	2.74626200	3.15991000	0.38704500
O	3.12951700	1.94139600	-1.44450300
C	-4.81953200	0.02603600	0.82630500
O	-2.61974500	2.96583200	-1.00439000
O	-2.54601300	3.03937500	1.22784000
H	0.26721500	-1.74030300	4.36050500
H	-0.99144900	-2.67361200	3.47245700
H	0.73518300	-3.11883000	3.30223600
H	5.38972700	0.50934600	1.46100800
H	5.04530100	-0.93109500	0.44013700
H	4.60330000	-0.94476400	2.16815100
C	3.91330000	3.91724900	0.09933500
H	-5.55056600	0.68039800	0.34077300
H	-4.88652400	0.12524400	1.91820400
H	-5.00591100	-1.02141200	0.55327800
C	-3.64635300	3.94700700	-0.98097600
H	4.79822100	3.26629100	0.09295100
H	3.82624600	4.40421600	-0.88090200
H	3.99809500	4.66757000	0.89081100
H	-3.29758100	4.86025000	-0.48102700
H	-4.52681500	3.56577900	-0.44567400
H	-3.89840500	4.15685800	-2.02470000
C	-0.15084300	-1.44131800	-1.34956100
C	-1.41110400	-2.33706100	-1.43063700
C	1.15742200	-2.21096100	-1.64496400
C	-0.36723500	-0.36313400	-2.42960500
O	-2.48974100	-1.82016400	-1.23057800
C	-1.35884600	-3.77729000	-1.87626800
O	1.28236500	-3.38617600	-1.39942600
C	2.28371100	-1.47581400	-2.34497200
H	-1.32603600	0.13849100	-2.25396700
H	0.43183400	0.38852300	-2.41156100
H	-0.39213700	-0.82010800	-3.43090500
H	-2.39071700	-4.11034300	-2.03718100
H	-0.77042700	-3.88657200	-2.79714800
H	-0.86306700	-4.37199100	-1.10235200

H	2.03398500	-1.39936000	-3.41522700
H	2.44233600	-0.45443000	-1.97687300
H	3.19584500	-2.07400500	-2.23826700
N	0.11529200	2.18569000	-0.31005800

Py-5-42356-R3-Nu3

C	0.14334600	-0.67040200	0.12817100
C	1.62266200	-0.30933900	0.03524900
C	-0.59599200	0.58378000	0.57607500
C	-0.04725500	-1.79840600	1.15791300
C	2.62456700	-1.35485000	0.24001100
C	1.96490900	0.98930500	-0.28154500
C	-1.85790400	0.43388500	1.30500300
C	-0.09254700	1.81178900	0.20421100
O	0.41811600	-1.43078600	2.35146300
O	-0.52793100	-2.87645900	0.93944500
O	3.89338200	-0.92170100	0.09085100
O	2.39101400	-2.50705800	0.53891800
C	3.34860100	1.38846500	-0.73758500
O	-2.55675100	1.58077600	1.41699700
O	-2.27353600	-0.59874100	1.78526500
C	-0.88478700	3.09510300	0.29877900
C	0.31524900	-2.41411500	3.36516300
C	4.90965700	-1.89557500	0.25277100
O	4.05984300	1.95491500	0.23156500
O	3.72100400	1.29748400	-1.87824800
C	-3.80432000	1.48557000	2.08258500
O	-1.71331600	3.20566000	-0.74464500
O	-0.73361400	3.95530900	1.12171300
H	0.71198700	-1.96277800	4.27988400
H	-0.73377600	-2.70778400	3.50590400
H	0.90169700	-3.30203900	3.09117500
H	5.86027700	-1.38053800	0.08171900
H	4.78469600	-2.71196700	-0.47105300
H	4.88447900	-2.31825000	1.26597200
C	5.36529900	2.38008200	-0.13193500
H	-4.22181500	2.49736900	2.10167500
H	-3.67315400	1.10927800	3.10560400
H	-4.47514500	0.80462100	1.53989100
C	-2.51835300	4.37540600	-0.77027800
H	5.95746900	1.52583600	-0.48815300
H	5.31946900	3.13501600	-0.92789100
H	5.81538600	2.80520000	0.76995700
H	-1.89209100	5.27683700	-0.78780600
H	-3.16365300	4.40885800	0.11858400
H	-3.12503200	4.31495000	-1.67868100
C	-0.43349200	-1.15369800	-1.29691300
C	-1.96228400	-1.29792400	-1.28560300
C	0.18463300	-2.43830800	-1.68570000
C	-0.01385700	-0.16843900	-2.41332400
C	-2.76943300	-0.16099400	-1.43962800
C	-2.58795800	-2.53925300	-1.12362600

N	0.68417300	-3.39182000	-2.10259500
H	1.07516500	-0.16532200	-2.54104500
H	-0.48435900	-0.47139900	-3.35831800
H	-0.33257400	0.85194000	-2.17255700
C	-4.16011900	-0.26148500	-1.38898400
H	-2.32097800	0.82377000	-1.56976300
C	-3.97764500	-2.64153000	-1.08912700
H	-1.97619700	-3.43246400	-1.00336100
C	-4.77157100	-1.50127000	-1.20926600
H	-4.76629000	0.64028100	-1.49585200
H	-4.44114400	-3.62103000	-0.96007100
H	-5.85945800	-1.58031900	-1.17377200
N	1.13310000	2.04295600	-0.29928100

Py-5-42356-R3-Nu2

C	0.61500000	-0.32234100	0.78029100
C	1.88094000	0.19736000	0.12882700
C	-0.22152300	0.90776900	1.13614700
C	0.89718200	-1.19393300	2.00088200
C	3.13668600	-0.52948500	0.27896700
C	1.71858700	1.24949000	-0.75375100
C	-1.07430400	0.93183900	2.33832500
C	-0.21300400	1.93215300	0.22294600
O	1.70499700	-0.59027400	2.85782000
O	0.42240100	-2.28742700	2.16075600
O	4.18679000	0.13532400	-0.24933900
O	3.28055400	-1.60615100	0.81830100
C	2.73550100	1.55644900	-1.82760400
O	-2.18198800	1.67586400	2.15449200
O	-0.84787600	0.37788100	3.38659100
C	-1.09371200	3.15231700	0.35481500
C	1.96797000	-1.30378000	4.05218600
C	5.42195800	-0.55799400	-0.25613700
O	3.38314100	2.69487400	-1.60071000
O	2.88106400	0.88820700	-2.81730700
C	-3.06845200	1.74542100	3.25800000
O	-2.13348300	3.10036100	-0.47921100
O	-0.85329100	4.09655900	1.05760700
H	2.64452500	-0.68048500	4.64467000
H	1.03069800	-1.47734100	4.59783900
H	2.43958300	-2.26859100	3.82192900
H	6.14924000	0.11004200	-0.72847200
H	5.34014800	-1.49332300	-0.82657500
H	5.73748600	-0.79815000	0.76783200
C	4.33286800	3.07140600	-2.58758300
H	-3.89789600	2.38911900	2.94847600
H	-2.56615900	2.16943800	4.13761300
H	-3.43609100	0.74080500	3.50632100
C	-2.98822000	4.23621600	-0.46378000
H	5.10683300	2.29807900	-2.68757900
H	3.84438400	3.21025100	-3.56114900
H	4.77692100	4.01147900	-2.24735500

H	-2.42788300	5.14304800	-0.72592100
H	-3.42905400	4.36795100	0.53366600
H	-3.77102200	4.04646700	-1.20404600
C	-0.18529300	-1.12495800	-0.29252600
Br	0.82170400	-2.47488500	-1.24480300
S	-1.80337300	-1.86796100	0.24059700
H	-0.46989200	-0.40033300	-1.06528000
O	-1.75557500	-3.32333700	0.19897600
O	-2.26169300	-1.19585200	1.45457100
C	-2.85918800	-1.31108100	-1.08613800
C	-3.21596800	-2.20140200	-2.09343400
C	-3.28166900	0.01876500	-1.07477600
C	-4.02920700	-1.74106000	-3.12983800
H	-2.86169300	-3.23222200	-2.05295800
C	-4.08542900	0.46433400	-2.12120200
H	-2.97689400	0.69177900	-0.26772400
C	-4.45905200	-0.41381400	-3.14234900
H	-4.32591400	-2.42089200	-3.92936200
H	-4.42239600	1.50134500	-2.13914400
H	-5.09084000	-0.05758200	-3.95733400
N	0.64426700	2.04960100	-0.81726900

Py-5-42356-R3-Nu4

C	0.05614500	-0.90275000	0.28939700
C	-0.01560500	-1.14200300	1.83350600
C	-1.07089400	0.02010200	-0.15395200
C	-1.38169900	-1.44877300	2.40869300
H	0.70308800	-1.93832400	2.07931900
H	0.34288300	-0.20523200	2.28473300
C	1.35346200	-0.17794700	-0.05449500
C	0.00798200	-2.24401300	-0.44855700
C	-2.36546600	-0.58221500	-0.44403000
C	-0.87930900	1.37430300	-0.00628300
C	-1.83048900	-2.88304300	2.48210400
C	-2.17289700	-0.44976000	2.82274500
C	2.54981200	-0.95106000	-0.35760800
C	1.37905400	1.19382700	0.09701200
O	0.08644300	-2.07504000	-1.77303600
O	-0.05655500	-3.32834900	0.06592700
O	-3.36353800	0.30811600	-0.61805700
O	-2.57312500	-1.77696900	-0.52949200
C	-2.00502300	2.37837900	-0.04999400
H	-1.97847800	-3.27898400	1.46832400
H	-1.06243900	-3.51019000	2.96083000
H	-2.76804300	-2.97281200	3.04843200
H	-1.86344900	0.59601400	2.73988800
H	-3.16696100	-0.64980500	3.23300500
O	3.54596000	-0.21132400	-0.89482700
O	2.67955600	-2.14837500	-0.19492200
C	2.67353600	1.97030200	0.16440000
C	0.06584200	-3.26537800	-2.53910400
C	-4.65407500	-0.23564800	-0.82711500

O	-2.22196100	2.84457300	-1.27746200
O	-2.57671200	2.78413600	0.92812400
C	4.78162600	-0.87489600	-1.08502300
O	3.41875000	1.59122800	1.20760000
O	2.97813800	2.87070300	-0.57067600
H	0.13756500	-2.96299700	-3.58865100
H	0.91408900	-3.90983200	-2.26938200
H	-0.86821100	-3.81718000	-2.36493200
H	-5.33352100	0.61488700	-0.94268100
H	-4.95912500	-0.84971800	0.03160200
H	-4.67240400	-0.86146900	-1.72925400
C	-3.26848000	3.79607500	-1.39659500
H	5.46023100	-0.14301300	-1.53520800
H	4.66079100	-1.73888200	-1.75167000
H	5.18745900	-1.22763800	-0.12596400
C	4.67979000	2.23019600	1.32425100
H	-4.21856100	3.35962200	-1.05827000
H	-3.05448600	4.68722800	-0.79191300
H	-3.32810600	4.06154500	-2.45626900
H	4.55839400	3.31549600	1.43970400
H	5.28699300	2.03700400	0.42843900
H	5.16315100	1.80567700	2.20939100
N	0.30051700	1.98354600	0.22699500

Py-5-42356-R4

C	0.00000000	0.00000000	1.12703600
C	0.00000000	1.20158700	0.42264800
C	0.00000000	-1.20158700	0.42264800
C	0.00000000	1.12910900	-0.96852500
C	0.00000000	-1.12910900	-0.96852500
N	0.00000000	0.00000000	-1.62998200
F	0.00000000	-2.25233000	-1.65680100
F	0.00000000	-2.35659800	1.05941100
F	0.00000000	0.00000000	2.43902200
F	0.00000000	2.35659800	1.05941100
F	0.00000000	2.25233000	-1.65680100

Py-5-42356-R4-Nu1

C	0.19170700	-0.07336900	-0.45894200
C	1.02645700	-1.23588100	-0.09349400
C	1.04468800	1.11163200	-0.24052500
C	2.36416400	-1.14729700	0.17071400
C	2.38718300	1.05094900	-0.00303300
F	0.46589500	2.32131600	-0.47602700
F	3.06338700	2.20964500	0.04648200
F	3.01186100	-2.28910200	0.45563500
F	0.41953800	-2.44252600	0.03367000
F	-0.18988900	-0.07306300	-1.83986400
C	-1.18331600	-0.05681100	0.31174500
C	-2.16721100	-1.13934800	-0.19228300
C	-1.91580600	1.27473400	0.03872900

C	-0.92281900	-0.27946200	1.80811600
O	-1.96508900	-1.86661700	-1.12632700
C	-3.44785600	-1.26663900	0.61497700
O	-2.58674700	1.39630500	-0.95889600
C	-1.85095500	2.36668700	1.07942400
H	-0.58583100	-1.31211400	1.97384200
H	-0.12791200	0.39182000	2.15870700
H	-1.82170900	-0.10271100	2.41332200
H	-3.24527100	-1.88732800	1.50096800
H	-3.82858800	-0.29720100	0.96339800
H	-4.20276000	-1.77135400	0.00147300
H	-2.56215700	2.12179200	1.88394700
H	-0.85364900	2.45669400	1.52360100
H	-2.15247600	3.31343600	0.61738900
N	3.10007300	-0.04544600	0.20764300

Py-5-42356-R4-Nu3

C	-0.70811300	0.88880400	-0.28981100
C	-0.86209200	0.60805700	1.14416700
C	-1.47856800	-0.14203300	-0.99427400
C	-1.76099700	-0.28879100	1.64520400
C	-2.34632600	-0.99032300	-0.36235000
F	-1.37334200	-0.21328800	-2.34835200
F	-3.05857300	-1.82633800	-1.13234400
F	-1.85331700	-0.39837800	2.97850600
F	-0.06970000	1.30316600	1.99671300
F	-1.23352100	2.21211600	-0.61309400
C	0.80343700	1.06102600	-0.72961200
C	1.60068100	-0.17979700	-0.33417500
C	1.34178800	2.25996400	-0.05310300
C	0.94254300	1.38273400	-2.23670500
C	1.40584800	-1.36935500	-1.04968500
C	2.48257700	-0.18214300	0.74952200
N	1.80788100	3.21707900	0.39170900
H	0.27422200	2.21380400	-2.49176400
H	1.97961100	1.67319100	-2.45157300
H	0.68087300	0.51861700	-2.85195000
C	2.08776600	-2.53025300	-0.69276400
H	0.70416400	-1.39195000	-1.88516600
C	3.16649200	-1.34569000	1.10635300
H	2.63327300	0.73113500	1.32686700
C	2.97332100	-2.52227700	0.38628000
H	1.92298100	-3.44761000	-1.26002100
H	3.85223800	-1.32793400	1.95487400
H	3.50722000	-3.43199100	0.66529800
N	-2.54340200	-1.09312300	0.94061900

Py-5-42356-R4-Nu2

C	1.51090500	0.21406800	-0.42787000
C	2.13572700	-1.11658700	-0.35141200
C	2.34168300	1.10917800	0.39663400

C	3.36295900	-1.33278600	0.21035500
C	3.54427300	0.74349300	0.93134200
F	1.87017500	2.36294600	0.60213800
F	4.22233300	1.67386200	1.61862500
F	3.87591400	-2.56631700	0.10406500
F	1.54867900	-2.10697400	-1.05928300
F	1.47317300	0.62464600	-1.80038800
C	0.01121500	0.32066100	0.03046000
Br	-0.89102600	1.86634600	-0.72176600
S	-1.02309400	-1.16565400	-0.27902300
H	-0.01921600	0.43389500	1.12048400
O	-1.11059100	-1.40620300	-1.71357500
O	-0.53951000	-2.18971400	0.64393700
C	-2.64634100	-0.69566500	0.29576700
C	-3.62147400	-0.35006900	-0.63671500
C	-2.90811600	-0.73632300	1.66393500
C	-4.89454900	-0.01822600	-0.17821800
H	-3.37370900	-0.34565300	-1.69865200
C	-4.18308300	-0.39238200	2.10974000
H	-2.12881400	-1.04504000	2.36245600
C	-5.17082600	-0.03412600	1.19044700
H	-5.67340100	0.25400300	-0.89133400
H	-4.40839700	-0.41397300	3.17648200
H	-6.16847500	0.23008500	1.54409700
N	4.10307700	-0.45407400	0.86807900

Py-5-42356-R4-Nu4

C	0.17599100	-0.42499800	0.23417000
C	1.36165600	-1.04861400	-0.52442500
C	-1.09213700	-1.11154200	-0.05002400
C	2.70365900	-0.36940500	-0.39664700
H	1.08814300	-1.11768600	-1.58658800
H	1.44587500	-2.07803100	-0.13836800
C	-0.07478100	1.00718100	0.03065600
C	-2.28985500	-0.46243300	-0.12972400
C	3.23292600	-0.11504500	0.98778300
C	3.40752200	-0.04944100	-1.48825300
C	-1.33539100	1.53111000	-0.04187600
N	-2.47094400	0.85136100	-0.10972400
H	4.26458400	0.26020400	0.95186400
H	3.20093100	-1.03384400	1.59227100
H	2.59737200	0.61831500	1.50180600
H	3.01433800	-0.22925800	-2.49193500
H	4.39772300	0.40634000	-1.40909900
F	0.54297100	-0.66186500	1.64352000
F	0.99161200	1.84853500	0.06570700
F	-1.45204400	2.86924100	-0.09600100
F	-3.40013600	-1.20437200	-0.28540900
F	-1.04350400	-2.46558700	-0.14802100

Py-5-42356-R5

C	0.00000000	1.19990300	0.24807200
C	0.00000000	0.00000000	0.94578600
C	-0.01951100	1.12530400	-1.14338700
C	0.00000000	-1.19990300	0.24807200
C	0.01951100	-1.12530400	-1.14338700
N	0.12317000	-2.36534300	-1.96961400
O	-0.38841200	-2.33422700	-3.04814300
O	0.73878900	-3.26900500	-1.46521500
N	-0.01687700	-2.48980300	0.97654900
O	-0.94986500	-3.20378600	0.74852600
O	0.90020800	-2.67505100	1.72589800
N	0.00000000	0.00000000	2.43005400
O	-0.76939300	-0.75637500	2.95207700
O	0.76939300	0.75637500	2.95207700
N	0.01687700	2.48980300	0.97654900
O	-0.90020800	2.67505100	1.72589800
O	0.94986500	3.20378600	0.74852600
N	-0.12317000	2.36534300	-1.96961400
O	-0.73878900	3.26900500	-1.46521500
O	0.38841200	2.33422700	-3.04814300
N	0.00000000	0.00000000	-1.80695300

Py-5-42356-R5-Nu1

C	0.39296200	-0.20472900	0.34100200
C	-0.90970700	-1.00147400	0.29347700
C	-0.04886900	1.23511300	0.46157900
C	-2.04476000	-0.41906200	-0.18689500
C	-1.24553700	1.63981000	-0.08992300
N	-1.49739900	3.07736000	-0.40595100
O	-0.76477300	3.57165300	-1.22763200
O	-2.44313500	3.59672700	0.11332900
N	-3.29552100	-1.19240500	-0.41882600
O	-3.99991700	-1.39293200	0.53303200
O	-3.52469500	-1.47689600	-1.56318200
N	-0.96017600	-2.39074800	0.72065800
O	-1.79684900	-3.10443600	0.21954000
O	-0.18143900	-2.74402600	1.57335700
N	1.12451200	-0.56960100	1.67623200
O	0.58598900	-0.21815300	2.68841100
O	2.15422600	-1.17592800	1.59064700
N	0.80936300	2.19225300	1.08055500
O	1.94601800	1.84457400	1.34832700
O	0.36820500	3.29807200	1.31971300
C	1.36790000	-0.47602000	-0.88398600
C	1.78649900	-1.97236000	-0.94561200
C	2.57569900	0.50822700	-0.87969500
C	0.55383400	-0.26042000	-2.18048900
O	0.89513600	-2.78924000	-0.96971000
C	3.21583500	-2.40843200	-1.10901900
O	3.64994000	0.18690000	-0.44311800
C	2.40367400	1.85441300	-1.55177400
H	-0.25360700	-0.99988400	-2.24029900

H	0.12538600	0.74855000	-2.23562500
H	1.20566300	-0.39891100	-3.05484500
H	3.21021300	-3.48487200	-1.31212900
H	3.70212900	-1.86299100	-1.92864700
H	3.77675700	-2.17824800	-0.19688100
H	2.52325000	1.71189500	-2.63728100
H	1.42198400	2.31981100	-1.39873400
H	3.20122500	2.51500900	-1.19490900
N	-2.22475200	0.86724600	-0.49186200

Py-5-42356-R5-Nu3

C	0.01640800	1.10280500	-0.01159100
C	-0.97935200	0.35464300	-0.87838300
C	1.35859200	0.52922600	-0.41977900
C	-0.72294100	-0.93643100	-1.25606100
C	1.43682400	-0.78879800	-0.81078400
N	2.74230000	-1.51629000	-0.81607500
O	3.29802800	-1.62545500	0.24718400
O	3.09140500	-1.99166800	-1.85969300
N	-1.79997300	-1.81741100	-1.80033300
O	-1.60107100	-2.29473100	-2.88292400
O	-2.74392100	-2.02741100	-1.08372700
N	-2.20906800	0.99279600	-1.27376300
O	-2.84743300	0.49689000	-2.17603900
O	-2.52065300	2.01244800	-0.69517100
N	-0.02542100	2.59409800	-0.47724500
O	0.16083600	2.77011600	-1.65163900
O	-0.24476600	3.44074100	0.33829000
N	2.53920500	1.34522000	-0.40374800
O	2.45831800	2.45723900	0.08109800
O	3.55070200	0.89762000	-0.90238200
C	-0.24017400	0.98482300	1.55501500
C	-0.38044500	-0.51765500	1.87737500
C	-1.49480100	1.64995400	1.96557500
C	0.88509200	1.61914800	2.39945300
C	0.74490100	-1.28274500	2.20482700
C	-1.62277300	-1.15458400	1.78209400
N	-2.44535200	2.13035900	2.40721300
H	0.91898500	2.70181700	2.25187000
H	0.69100600	1.39254600	3.45583000
H	1.86202000	1.20335200	2.13245200
C	0.63244400	-2.65760100	2.40669500
H	1.73063900	-0.82575500	2.27273600
C	-1.73638400	-2.52778500	1.98895600
H	-2.51402600	-0.57806500	1.52612100
C	-0.60731500	-3.28497300	2.29774300
H	1.52537200	-3.23758400	2.64378000
H	-2.71246900	-3.00500400	1.89529600
H	-0.69373000	-4.36134000	2.45271700
N	0.43262300	-1.57397700	-1.14427700

Py-5-42356-R5-Nu2

C	-0.84675200	-0.16393200	-0.75161200
C	-1.35379000	1.24650300	-0.53102700
C	-1.81349600	-1.05179200	0.01346600
C	-1.93738500	1.55443800	0.66650600
C	-2.35369800	-0.56484400	1.18123600
N	-2.97969800	-1.46609600	2.19268800
O	-2.22270200	-2.16727800	2.81072600
O	-4.16233200	-1.35669500	2.35722600
N	-2.15201200	2.96265200	1.10169600
O	-3.28219500	3.36498300	1.08079400
O	-1.18211100	3.54240300	1.51062300
N	-1.06588500	2.26874200	-1.50220800
O	-1.52119800	3.37449900	-1.31709400
O	-0.39449000	1.95352800	-2.46038100
N	-0.95537900	-0.48932200	-2.26344200
O	-2.05141200	-0.35446800	-2.73695200
O	0.03647600	-0.83694500	-2.82783100
N	-2.10812500	-2.38951300	-0.42421500
O	-1.56017000	-2.80180000	-1.42323400
O	-2.91780100	-3.02704500	0.21753500
C	0.59958600	-0.34509300	-0.20535600
Br	1.29856700	-2.13929200	-0.30615300
S	1.90052400	0.87869300	-0.70813700
H	0.51276200	-0.13266200	0.87019600
O	2.37978500	0.62196800	-2.05384300
O	1.35936100	2.18166900	-0.32490800
C	3.20995600	0.50470500	0.43735100
C	4.29255500	-0.25675600	0.00270400
C	3.13288900	1.02961800	1.72657400
C	5.32450200	-0.51449800	0.90225900
H	4.31616500	-0.63160600	-1.02121400
C	4.16993700	0.75724000	2.61642800
H	2.28403300	1.65130600	2.01655300
C	5.25890500	-0.01295900	2.20386300
H	6.18327700	-1.10721200	0.58593000
H	4.13192400	1.15597300	3.63050200
H	6.06963500	-0.21982600	2.90379000
N	-2.35389600	0.69151200	1.58464700

Py-5-42356-R5-Nu4

C	-0.23805200	-0.79001900	-0.04411100
C	-0.80650400	-1.46205600	1.23565800
C	1.26970400	-0.66742700	0.00132600
C	-2.25878900	-1.19172600	1.57539100
H	-0.18696900	-1.05468700	2.04745700
H	-0.60823700	-2.53764400	1.16941800
C	-0.77455600	0.60134200	-0.29406800
C	1.87784800	0.54735900	0.19021600
C	-3.27984200	-2.21285800	1.15775900
C	-2.58701100	-0.08496800	2.25201900
C	0.00377100	1.70666000	-0.08582100

N	1.29887300	1.73450900	0.19382200
H	-4.28377600	-1.92844500	1.49875600
H	-3.02740100	-3.19660600	1.58392700
H	-3.28767600	-2.32659500	0.06573500
H	-1.82959100	0.64046000	2.56328900
H	-3.62675700	0.13478500	2.50561600
N	-0.57012600	-1.67139300	-1.30795000
O	-0.11637400	-1.27284400	-2.34908700
O	-1.22381200	-2.66331400	-1.16053500
N	2.04047500	-1.87443700	-0.04929400
O	3.25200400	-1.79747400	-0.03941400
O	1.43038000	-2.92414400	-0.12381800
N	3.33851500	0.66401200	0.46565100
O	4.03145600	1.01601400	-0.44862000
O	3.67986200	0.47572700	1.60285600
N	-2.15660000	0.74138400	-0.66383700
O	-2.70337200	-0.23503800	-1.13326000
O	-2.69913300	1.81463700	-0.50964300
N	-0.55051300	3.08597600	-0.17971700
O	-0.81834600	3.61534600	0.86487300
O	-0.60683600	3.57490600	-1.27524400

TS-Ph-4-1234-R1-Nu4

C	-1.37117300	-0.58280600	-0.59915900
C	-1.92157000	-1.93766200	1.45254400
C	-0.68615200	-1.65830000	-1.15302100
C	-0.57963700	-2.11973600	1.77365300
H	-2.52517500	-1.18517100	1.96075800
H	-2.45717300	-2.70010600	0.88105400
C	-0.60499300	0.57759200	-0.16539900
C	0.67271700	-1.81416400	-0.92349500
C	0.10020700	-1.12030300	2.69227100
C	0.21312000	-3.07053600	1.13697200
C	0.79903800	0.52636000	-0.25572100
C	1.45931200	-0.66010000	-0.59276100
H	-0.47798000	-0.18896500	2.76400700
H	0.21157000	-1.52759300	3.71052100
H	1.10718300	-0.86985600	2.32435800
H	-0.24822300	-3.89974500	0.59549100
H	1.27273600	-3.17191700	1.38247200
C	2.93457500	-0.88079100	-0.48637500
C	1.62209800	1.80542900	-0.13781200
C	-1.33174100	1.64993100	0.58295100
C	-2.81210900	-0.44381900	-1.03211200
F	-2.91222400	0.36450600	-2.10440600
F	-3.33547900	-1.62070400	-1.40369600
F	-3.64169100	0.05896800	-0.11667900
F	-2.11306800	1.13529700	1.55449400
F	-2.15731200	2.38830300	-0.18563800
F	-0.53558200	2.51371500	1.22235400
F	2.75065500	1.72578600	-0.85280800
F	0.97406700	2.86099800	-0.64202600

F	1.98448700	2.10162900	1.11153900
F	3.65598000	-0.54854800	-1.57254100
F	3.19832300	-2.18461200	-0.28072700
F	3.49064800	-0.22948600	0.55108300
H	-1.25237200	-2.47815400	-1.59444300
H	1.17806900	-2.69773400	-1.30797600

TS-Ph-4-1234-R3-Nu4

C	-1.19809400	-1.23813700	-0.25893900
C	-1.70806800	-1.29582200	2.26503800
C	-0.42293200	-0.04728200	-0.22614400
C	-1.99839700	0.05008000	2.33085700
H	-2.49522800	-2.04885700	2.32890500
H	-0.67216000	-1.63920200	2.30405400
C	-0.55918700	-2.49892200	-0.27903100
C	-2.61478900	-1.22160500	-0.77175500
C	-1.16631600	1.23243500	-0.20242100
C	0.98367800	-0.16270300	-0.10182000
C	-3.44230700	0.49901400	2.38180200
C	-1.01494600	1.03902300	2.15941700
C	0.80248500	-2.59630200	-0.15087900
O	-2.62563200	-0.86227000	-2.06535600
O	-3.60483400	-1.60202300	-0.20397900
O	-0.35990400	2.33214500	-0.32880100
O	-2.37197700	1.32177800	-0.42887500
C	1.59110000	-1.42052400	-0.05383100
C	1.82533800	1.08108200	0.05176700
H	-3.65703900	1.14064800	1.51505700
H	-4.12875700	-0.35801100	2.35512200
H	-3.64206100	1.08172800	3.29590000
H	0.04295400	0.80262700	2.28125100
H	-1.28887200	2.09114400	2.28128200
C	-3.90943400	-0.67718000	-2.63295700
C	-1.02531100	3.57114700	-0.29229500
C	3.05736800	-1.64522500	0.05965500
O	2.19115900	1.58256500	-1.12532500
O	2.14477500	1.55567900	1.10937000
H	-3.75088500	-0.36408400	-3.67002000
H	-4.49331800	-1.60712900	-2.60254200
H	-4.45105400	0.10320400	-2.08021400
H	-0.26049700	4.34587100	-0.42017700
H	-1.53568700	3.71442400	0.67367500
H	-1.77424700	3.64540000	-1.09255000
O	3.79553000	-0.54430000	-0.11606100
O	3.56081800	-2.72358100	0.27272500
C	2.94394400	2.78047900	-1.06247400
C	5.19939600	-0.69929700	0.02229500
H	2.37116600	3.56265800	-0.54493600
H	3.88582000	2.61690200	-0.52011500
H	3.14714400	3.07747200	-2.09594700
H	5.58321800	-1.40893900	-0.72244400
H	5.44943300	-1.06993500	1.02513500

H	5.63532500	0.29158600	-0.13644000
H	-1.17282700	-3.39733000	-0.37650100
H	1.30692100	-3.56169900	-0.13779000

TS-Ph-4-1234-R5-Nu2

C	-1.30641000	-0.20972200	1.43122500
C	-2.05979100	-0.84926600	0.44243100
C	-1.53132500	1.12083900	1.75433000
C	-2.97381000	-0.11628800	-0.28726600
C	-2.43883000	1.86383200	1.00346700
C	-3.13435200	1.24822200	-0.02072600
N	-3.99896800	2.07948000	-0.87245200
O	-4.51893900	3.03626000	-0.35751900
O	-4.09131500	1.76183700	-2.03230300
N	-3.81728900	-0.80533400	-1.28289700
O	-5.00522100	-0.66502800	-1.16283700
O	-3.24971700	-1.46729800	-2.11117800
N	-1.90144200	-2.28438000	0.11518600
O	-2.86754600	-2.97405600	0.33251800
O	-0.86140400	-2.62282200	-0.36270400
N	-0.41785300	-0.97795400	2.32650800
O	-0.55001800	-2.17624000	2.32975900
O	0.32297200	-0.34598600	3.02952300
C	0.86783500	0.35975400	-0.43571400
Br	1.37943900	1.88351300	0.64315100
S	2.00258000	-0.92596700	-0.36766800
H	0.77744200	0.69087600	-1.47571800
O	2.04663600	-1.49633200	0.98785000
O	1.70613200	-1.81356200	-1.50423900
C	3.68481800	-0.33890800	-0.66132900
C	4.48780600	0.04147000	0.41305000
C	4.14200400	-0.23202000	-1.97458700
C	5.76603000	0.54264400	0.16517600
H	4.10659300	-0.06996300	1.42908300
C	5.42124400	0.26745900	-2.21429700
H	3.49535100	-0.55535400	-2.79206400
C	6.23188300	0.65687700	-1.14560300
H	6.40293900	0.84103400	0.99953000
H	5.79031700	0.34755500	-3.23810400
H	7.23318500	1.04681300	-1.33563400
H	-0.96855400	1.56264500	2.57286700
H	-2.60531100	2.92267000	1.19611500

TS-Ph-4-1235-R1-Nu4

C	-0.24388500	-1.14299900	-0.37129300
C	0.28980600	-1.67287700	2.19512500
C	1.13962500	-1.07099300	-0.55405400
C	1.04810800	-0.52403700	2.42630300
H	-0.77887100	-1.68831100	2.41590800
H	0.77350900	-2.63208500	1.99610400
C	-1.03349000	0.04995100	-0.41760100

C	1.79448800	0.14200400	-0.51371300
C	0.30594500	0.72279700	2.87978400
C	2.40610500	-0.42247800	2.17006400
C	-0.33206500	1.28794600	-0.38241200
C	1.04356300	1.34196300	-0.46295000
H	0.22932300	0.75897600	3.97910800
H	0.81944100	1.63903300	2.55193400
H	-0.71652600	0.74247300	2.47506500
H	2.99652500	-1.30280900	1.90402200
H	2.94663400	0.51057700	2.34069200
C	3.26270900	0.22594200	-0.79006900
C	-1.02083300	2.62941100	-0.21513000
C	-2.51657000	-0.02749200	-0.23700800
C	-0.83340700	-2.53437100	-0.49004700
F	-1.33124800	-2.75348500	-1.71855700
F	0.09180000	-3.48485700	-0.30610800
F	-1.81404700	-2.78838700	0.37763300
F	-2.87749500	-0.21034500	1.05020000
F	-3.07082800	-1.03870100	-0.92134400
F	-3.15863600	1.06989100	-0.65243600
F	-0.13982600	3.60740300	0.03171200
F	-1.70070700	3.01632500	-1.29750700
F	-1.86659900	2.63159900	0.82259800
F	3.86956300	1.17804000	-0.07359400
F	3.90557000	-0.92307500	-0.57166000
F	3.49126300	0.54421100	-2.08033700
H	1.71895800	-1.99155400	-0.60815600
H	1.56113400	2.29753800	-0.42207700

TS-Ph-4-1235-R4-Nu4

C	-0.07657400	0.19235500	1.35194500
C	2.17741100	-0.96763400	0.64334300
C	-1.08630100	-0.75089000	1.16709600
C	1.72211800	-1.31932800	-0.63465700
H	2.83550800	-0.10652100	0.78517300
H	2.05039100	-1.64469900	1.49269300
C	0.04116900	1.26298000	0.45187900
C	-1.90743600	-0.63806500	0.05100900
C	2.05699700	-0.33366400	-1.74544500
C	0.96896100	-2.42867700	-0.97324800
C	-0.77836300	1.31556800	-0.65580900
C	-1.80949600	0.39425900	-0.86645400
H	3.13413600	-0.35823200	-1.98019300
H	1.50184900	-0.55190100	-2.66975300
H	1.82427800	0.69924400	-1.44058700
H	0.72190100	-3.19392300	-0.23148400
H	0.59237500	-2.56837600	-1.98910000
F	1.03863300	2.13991700	0.59027900
F	-0.56575100	2.28607600	-1.54980500
F	-2.86387000	-1.55833900	-0.12325100
F	0.52016500	0.29575800	2.54484400
H	-2.45606000	0.45980400	-1.73878300

H	-1.21788100	-1.56752700	1.87547600

TS-Ph-4-1245-R1-Nu4

C	-1.21389300	-0.63884300	-0.31463800
C	-1.22027900	-1.13299400	2.18618700
C	0.00000300	-1.30800800	-0.43270300
C	0.00001600	-0.51376900	2.43791000
H	-2.16105500	-0.61091000	2.37030200
H	-1.27302700	-2.21231700	2.02681300
C	-1.22201000	0.79470400	-0.46421800
C	1.21390800	-0.63884900	-0.31462200
C	-0.00007700	0.94925200	2.84105500
C	1.22041300	-1.13280500	2.18620500
C	-0.00000200	1.45478800	-0.50597000
C	1.22199600	0.79472400	-0.46417700
H	0.00024800	1.05487300	3.93851000
H	0.89219000	1.46314300	2.45581700
H	-0.89271400	1.46290300	2.45635700
H	1.27337400	-2.21211400	2.02681400
H	2.16107600	-0.61053200	2.37035700
C	2.46104600	-1.45565700	-0.52719800
C	2.46441200	1.59838700	-0.34322200
C	-2.46442400	1.59836600	-0.34324600
C	-2.46104600	-1.45565200	-0.52716800
F	-2.87278000	-1.39777700	-1.80553500
F	-2.26901000	-2.75380300	-0.26049400
F	-3.49593400	-1.05751900	0.21802600
F	-3.00940200	1.54771500	0.89302000
F	-3.44457900	1.20886400	-1.17929600
F	-2.25376000	2.89905700	-0.59476100
F	3.44453700	1.20891700	-1.17932000
F	3.00942300	1.54767800	0.89302500
F	2.25374000	2.89908700	-0.59467300
F	3.49595900	-1.05753400	0.21796500
F	2.26900600	-2.75381000	-0.26052700
F	2.87274700	-1.39778800	-1.80558000
H	-0.00000100	-2.39745800	-0.43979400
H	-0.00000700	2.54278000	-0.56699700

TS-Ph-4-1245-R3-Nu4

C	-0.88128700	-0.83409600	-0.45668900
C	-1.03149100	-1.42019000	2.45313400
C	-0.86353800	0.57663500	-0.26249900
C	-1.88964500	-0.36316300	2.39398200
H	-1.39054800	-2.43486200	2.63669400
H	0.04538600	-1.27451000	2.33759700
C	0.30891200	-1.55457600	-0.41750400
C	-2.13420400	-1.56000700	-0.83515400
C	-2.11836600	1.34311000	-0.25031500
C	0.41117800	1.22056500	-0.31011800
C	-3.37971700	-0.58452700	2.52474800

C	-1.45758800	0.95081500	2.05022100
C	1.54967200	-0.93002100	-0.29457100
O	-2.56684600	-1.15477500	-2.03827500
O	-2.66710400	-2.45310200	-0.22662400
O	-1.90605100	2.68647800	-0.27948800
O	-3.24552500	0.88338100	-0.35024700
C	1.58215800	0.49992800	-0.30603700
H	-3.61759300	-1.64442900	2.68756700
H	-3.79480600	0.00124500	3.36085200
H	-3.87920400	-0.25116700	1.60324100
H	-0.40315700	1.20480300	2.17643900
H	-2.15902600	1.77908200	2.19086800
C	2.72821700	-1.78560000	-0.09480900
C	-3.84819200	-1.62101500	-2.41912300
C	-3.06744800	3.48191700	-0.17593300
C	2.85789300	1.23730900	-0.54912700
O	3.69902900	-1.17818600	0.60988500
O	2.82860700	-2.93946200	-0.45345200
H	-4.04697900	-1.21080500	-3.41451900
H	-3.87738900	-2.71845200	-2.44828900
H	-4.60215900	-1.25976500	-1.70527300
H	-2.73825200	4.52559000	-0.22264400
H	-3.58515400	3.29716500	0.77790100
H	-3.76857100	3.26981300	-0.99460700
O	2.89522800	2.41844800	0.08265800
O	3.74543000	0.86630900	-1.27586100
C	4.90858000	-1.89912200	0.75302600
C	4.02806100	3.22914800	-0.19001600
H	5.34237600	-2.12736300	-0.23065700
H	4.74059300	-2.84301900	1.28914800
H	5.58750100	-1.25693800	1.32308000
H	4.08991400	3.46036400	-1.26202700
H	4.95081600	2.71592700	0.11168800
H	3.89763500	4.14774400	0.38981400
H	0.28055100	-2.64453900	-0.48877200
H	0.44673600	2.30872300	-0.34616600

TS-Ph-4-2134-R1-Nu4

C	-0.61560100	0.35805100	-0.13926100
C	-1.32855600	1.06679700	2.09791500
C	-1.35106000	-0.91200600	-0.13045800
C	-0.91912300	-0.17434400	2.55081900
H	-0.77718700	1.97059500	2.36115100
H	-2.33907700	1.20175700	1.71154900
C	0.83031100	0.23721100	-0.16254900
C	-0.68440200	-2.07021400	-0.61764100
C	0.47992300	-0.30110800	3.11059600
C	-1.61250300	-1.32141600	2.14321500
C	1.45237000	-0.97315700	-0.45725000
C	0.67164600	-2.11921700	-0.76619300
H	1.21668900	-0.44980600	2.30384600
H	0.77310400	0.61019100	3.65136100

H	0.56882400	-1.15529500	3.79759700
H	-2.69733600	-1.29684700	2.06073800
H	-1.18333100	-2.30741400	2.34965000
C	-2.83734200	-0.95839700	-0.39858300
C	2.94896600	-1.18694300	-0.51974400
C	1.69676000	1.44071600	0.19042900
C	-1.19686900	1.52413700	-0.89668700
F	-1.19421600	1.27995100	-2.22910800
F	-2.46754200	1.81296800	-0.59410200
F	-0.51543300	2.66646900	-0.75432000
F	1.10999200	2.30003400	1.02161800
F	2.09315600	2.13481500	-0.88459400
F	2.81476200	1.06778000	0.83438800
F	3.24586700	-2.30111600	-1.20576900
F	3.60377300	-0.19982500	-1.14379200
F	3.51624500	-1.34744800	0.68446500
F	-3.13307200	-0.60713400	-1.66779700
F	-3.31677700	-2.20521100	-0.25062400
F	-3.60622100	-0.18385400	0.37651000
H	1.15388600	-3.03370100	-1.10110800
H	-1.27749300	-2.95700800	-0.84431300

TS-Ph-4-2134-R3-Nu4

C	-0.80855100	0.01595500	-0.09497000
C	-0.90678700	0.64633200	2.36345400
C	0.60592400	-0.15839100	-0.14185200
C	0.16790800	1.50906200	2.29498000
H	-1.92847000	1.02044700	2.44997600
H	-0.74333200	-0.42576700	2.49800100
C	-1.64204700	-1.13528000	0.00535100
C	-1.39411100	1.29716600	-0.66249700
C	1.45986100	1.03426400	-0.30648700
C	1.12506900	-1.45060200	0.09706200
C	-0.06630500	3.00188900	2.21451800
C	1.47869000	1.05515200	2.07761600
C	-3.11618800	-1.00456400	-0.15390600
C	-1.09867700	-2.37915800	0.27912700
O	-1.28339000	1.26719000	-2.00054500
O	-1.91567100	2.20200900	-0.07409600
O	2.79128500	0.72083900	-0.43841500
O	1.04870800	2.13702200	-0.65912900
C	0.29439500	-2.53282500	0.34364300
C	2.59797900	-1.74362500	0.11469500
H	0.17235400	3.34785600	1.19774300
H	-1.11268900	3.26008600	2.42384100
H	0.58126500	3.54026200	2.92492300
H	1.74584600	0.01350200	2.26437200
H	2.30340600	1.77366500	2.09550300
O	-3.78566800	-2.06974000	0.30863300
O	-3.68294500	-0.06589100	-0.65920100
C	-1.67837400	2.45323400	-2.66252700
C	3.65513700	1.82504700	-0.55265500

O	3.07757400	-1.95438500	-1.11132600
O	3.25684300	-1.87511500	1.11364800
C	-5.19439600	-2.03640800	0.14097800
H	-1.53860600	2.27605800	-3.73412500
H	-2.73072000	2.68769800	-2.45092000
H	-1.05040900	3.29294000	-2.33207100
H	4.67150000	1.42552800	-0.64760300
H	3.59326000	2.46530300	0.34214200
H	3.40803200	2.43659000	-1.43139900
C	4.47980300	-2.15429200	-1.19346000
H	-5.57761500	-2.96927800	0.56514500
H	-5.45578100	-1.96546700	-0.92339100
H	-5.62510300	-1.17359600	0.66604500
H	5.00836800	-1.27983200	-0.78895400
H	4.78230600	-3.04432200	-0.62574300
H	4.71426700	-2.28256600	-2.25447300
H	-1.75645600	-3.23640500	0.41243500
H	0.72870100	-3.51588700	0.53358900

TS-Ph-4-2134-R4-Nu4

C	-0.42531100	-0.86302100	0.75372900
C	-1.41342600	1.57880400	0.98278000
C	0.91740100	-0.49221500	0.73159500
C	-0.66988300	2.10827500	-0.07890500
H	-2.47070400	1.33030400	0.85784100
H	-1.00871300	1.55376700	1.99776100
C	-0.97582400	-1.39283900	-0.42463900
C	1.64497400	-0.55487100	-0.45226200
C	-1.38581600	2.16382900	-1.42035600
C	0.65742800	2.49973500	-0.04820700
C	-0.25217800	-1.46940300	-1.59474000
C	1.09292900	-1.06757300	-1.61467300
H	-2.25579500	2.83968900	-1.37913400
H	-0.72286000	2.50588000	-2.22835100
H	-1.77025300	1.16671500	-1.69129300
H	1.24043300	2.45623400	0.87612100
H	1.16794500	2.83787300	-0.95292400
F	-2.26690400	-1.75139300	-0.39108000
F	2.91214300	-0.13476600	-0.42722000
F	1.47862700	-0.02518600	1.84528000
F	-1.04299700	-1.06609300	1.92031900
H	1.69369700	-1.10590700	-2.52181400
H	-0.74334400	-1.85193100	-2.48983700

TS-Ph-4-2134-R5-Nu2

C	-1.95531600	0.54974600	0.34990100
C	-2.22412100	-0.81310800	0.20284300
C	-2.15157800	1.39828100	-0.74190200
C	-2.48137500	-1.32080800	-1.05633100
C	-2.40042000	0.87660200	-2.00790000
C	-2.55608000	-0.49009300	-2.17370200

N	-2.57848900	-2.77270900	-1.29492600
O	-3.43873300	-3.13471100	-2.05749900
O	-1.76384100	-3.47438500	-0.75569300
N	-2.15243100	-1.69065700	1.38154500
O	-3.00753200	-2.53701700	1.47081500
O	-1.26794700	-1.48010800	2.16482800
N	-1.79418300	1.08760800	1.72920900
O	-2.74031300	0.88891300	2.45428100
O	-0.78990100	1.66756500	1.99933300
N	-2.12507300	2.85844700	-0.59980600
O	-2.36796300	3.31124500	0.49338400
O	-1.91983700	3.50808400	-1.59487600
C	0.63001400	0.08485300	-0.27739000
Br	1.10848600	1.90985200	-0.66880700
S	1.67306100	-0.70253800	0.83356000
H	0.63540600	-0.48860600	-1.21219200
O	1.74175800	0.01789200	2.11019600
O	1.26706000	-2.11935700	0.82716900
C	3.36646600	-0.69446900	0.21191800
C	4.21995400	0.35474600	0.55136300
C	3.78122900	-1.71220400	-0.64706100
C	5.50614500	0.38787900	0.01186800
H	3.87014000	1.12413800	1.24108700
C	5.06925000	-1.67375400	-1.17869900
H	3.09420800	-2.52835300	-0.87639200
C	5.92979000	-0.62289700	-0.85258800
H	6.18251100	1.20385100	0.27160300
H	5.40607900	-2.46947400	-1.84518500
H	6.93747000	-0.59518900	-1.27037100
H	-2.49043200	1.56340800	-2.84787900
H	-2.75590100	-0.92641500	-3.15097100

TS-Ph-4-2135-R1-Nu4

C	-0.92553000	0.00736000	-0.20202100
C	-0.89908100	-0.31614100	2.31818200
C	-0.18321000	-1.16703800	-0.40633300
C	0.48645100	-0.36587900	2.39069100
H	-1.43434700	0.60366500	2.56418500
H	-1.48666900	-1.23127000	2.23789500
C	-0.25154900	1.27744300	-0.43510100
C	1.21586500	-1.12420300	-0.33165800
C	1.24505600	0.90989300	2.70267400
C	1.21938500	-1.50156100	2.02950500
C	1.12972000	1.28361100	-0.60350300
C	1.87785100	0.12194400	-0.57377600
H	1.43525200	0.99606000	3.78529400
H	2.21767400	0.92527600	2.19133900
H	0.67145900	1.79467600	2.39145800
H	0.73037700	-2.47695500	1.98023000
H	2.30851600	-1.50058700	2.11427600
C	-0.79004600	-2.54981900	-0.49831000
C	3.35098200	0.14669800	-0.63398800

C	-0.92562800	2.59966800	-0.33326000
C	-2.43928500	-0.01680100	-0.24043300
F	-2.89815300	0.12182800	-1.49432300
F	-2.97111000	-1.14512200	0.23461900
F	-2.98285000	0.96936200	0.47787400
F	-1.31556700	2.91650600	0.92241500
F	-2.02906600	2.71956200	-1.09593500
F	-0.11470400	3.60199700	-0.71322000
F	3.86096200	-0.82904600	-1.41110400
F	3.93747700	-0.04264400	0.57607500
F	3.83675700	1.30757000	-1.09468400
F	-1.75911100	-2.61387600	-1.42050300
F	0.12703300	-3.45620400	-0.86517700
F	-1.30518300	-3.00901400	0.64971400
H	1.63715200	2.23405000	-0.76927700
H	1.77633700	-2.04423100	-0.48315800

TS-Ph-4-2135-R3-Nu4

C	0.62047600	0.35119900	-0.15530200
C	0.90360300	0.00176700	2.41786000
C	-0.03371400	-0.89480500	-0.34077600
C	1.35059900	-1.28953600	2.26488500
H	1.58774100	0.81097300	2.67920000
H	-0.16634600	0.22438800	2.40343800
C	-0.17318300	1.53302900	-0.08202900
C	2.09479600	0.46020500	-0.50013500
C	0.76286300	-2.13867300	-0.45027200
C	-1.43277900	-0.94176700	-0.31455100
C	2.82924200	-1.59238200	2.35049700
C	0.51277300	-2.33476200	1.81780900
C	0.48490600	2.86876100	-0.04218100
C	-1.55324800	1.45845000	-0.03718200
O	2.21973900	0.48716900	-1.83612200
O	3.03046500	0.54417400	0.24372500
O	-0.02509400	-3.22243100	-0.78141400
O	1.97232700	-2.16021100	-0.68506800
C	-2.19394900	0.20951600	-0.15125900
H	3.19244400	-1.87625600	1.35126100
H	3.40256000	-0.71808500	2.68500900
H	3.01866800	-2.43103900	3.03971800
H	-0.57024600	-2.24119300	1.92341800
H	0.90211500	-3.35728200	1.81921700
O	-0.34727500	3.84702600	0.33605900
O	1.63714900	3.08416400	-0.33048200
C	3.54979200	0.48714700	-2.31976900
C	0.67466000	-4.43018700	-0.93948500
C	-3.67608000	0.17985400	-0.12828100
C	0.20100000	5.15606800	0.35625300
H	3.48581500	0.53433600	-3.41181900
H	4.10611600	1.35245400	-1.93421200
H	4.06218200	-0.43475200	-2.01045000
H	-0.06578600	-5.19658200	-1.19769300

H	1.18787900	-4.71632400	-0.00651300
H	1.43050400	-4.35667400	-1.73405400
O	-4.17996200	-1.05265500	-0.27136300
O	-4.38064600	1.15299100	0.00633400
H	-0.60382500	5.82193700	0.68133600
H	0.55337200	5.44317900	-0.64342700
H	1.04579600	5.20922300	1.05562700
C	-5.59537800	-1.15162300	-0.25335800
H	-6.03463900	-0.55963000	-1.06734200
H	-5.99621300	-0.78672300	0.70180700
H	-5.83251900	-2.21157100	-0.38419900
H	-2.15282900	2.36160300	0.06176600
H	-1.92469300	-1.90736200	-0.42176700

TS-Ph-4-2135-R5-Nu2

C	2.01268600	-0.41102700	0.68157300
C	1.75135800	0.82987900	1.26247700
C	2.49018000	-0.44991100	-0.63205900
C	1.82996600	1.99647200	0.52430700
C	2.56925800	0.69840600	-1.39800400
C	2.22316000	1.90145700	-0.80109600
N	2.29882600	3.13429200	-1.60744000
O	2.65517700	3.02304900	-2.75441900
O	1.99996300	4.16875200	-1.06518300
N	1.24708200	0.94015400	2.64045400
O	1.27213700	2.03427600	3.14395300
O	0.85410200	-0.06797200	3.16651500
N	1.97445500	-1.65120200	1.50069500
O	2.88558100	-1.76328200	2.28046400
O	1.08437300	-2.41966400	1.30509600
N	2.85589400	-1.71984200	-1.27778700
O	3.14996200	-2.64296900	-0.55866100
O	2.86612800	-1.73930700	-2.48249100
C	-0.58431400	-0.26215400	-0.53090300
Br	-0.91220500	-1.90697500	-1.47119800
S	-1.60616300	0.05149500	0.78679600
H	-0.60963200	0.58181600	-1.22846000
O	-1.70415600	-1.10509500	1.68560800
O	-1.13739400	1.34494100	1.34142200
C	-3.30251800	0.36383100	0.25983900
C	-4.18451100	-0.71013800	0.13451100
C	-3.68768600	1.65624800	-0.09859000
C	-5.46696400	-0.48453700	-0.36609400
H	-3.85997200	-1.70550000	0.44046600
C	-4.97321200	1.87370100	-0.59190200
H	-2.97997300	2.47746000	0.02554200
C	-5.86117500	0.80417900	-0.72955600
H	-6.16403700	-1.31803000	-0.46708900
H	-5.28610400	2.88251800	-0.86631100
H	-6.86628100	0.97739200	-1.11739100
H	2.88959800	0.65099900	-2.43702600
H	1.56403200	2.94947500	0.97656100

TS-Ph-4-5123-R1-Nu4

C	-1.72939000	0.35827400	-0.36179000
C	-2.44456900	0.26783500	2.12288800
C	-1.17027600	-0.94112700	-0.51937600
C	-1.55935100	-0.79201200	2.26815600
H	-2.20543300	1.25408400	2.52948700
H	-3.48205300	0.08953200	1.83715500
C	-0.87438700	1.46839900	-0.31322500
C	0.23456600	-1.07506700	-0.48948600
C	-0.19805300	-0.53410900	2.88913500
C	-1.77148300	-2.02435400	1.66172100
C	0.49243600	1.32526500	-0.21460100
C	1.07118600	0.00681900	-0.23430500
H	-0.21625500	-0.70776300	3.97775500
H	0.56361200	-1.20141800	2.45882700
H	0.12830100	0.50411100	2.72314600
H	-2.76030700	-2.27861700	1.27151100
H	-1.05526100	-2.84041000	1.77705500
C	0.74875400	-2.48185500	-0.71601400
C	2.54893600	-0.17305400	0.01133200
C	1.29692000	2.58832600	-0.10575900
C	-3.14250300	0.57054500	-0.78512100
F	-3.25037600	0.68746900	-2.12917100
F	-3.94367900	-0.45240800	-0.45712400
F	-3.68292000	1.68794800	-0.28547800
F	2.42399600	2.54862100	-0.83039500
F	0.60511000	3.64880500	-0.54912700
F	1.65444300	2.89492200	1.15170000
F	3.29225600	-0.07673800	-1.09771800
F	2.83595400	-1.35724600	0.56438200
F	3.01662500	0.74119800	0.86999200
F	1.02396500	-3.13768400	0.41657700
F	-0.15401600	-3.22836600	-1.36309300
F	1.85587200	-2.50346900	-1.46846200
H	-1.30979100	2.46797400	-0.33438700
H	-1.77459000	-1.74826500	-0.92060100

TS-Ph-4-5123-R5-Nu2

C	1.15193400	-1.10695300	1.09984000
C	1.37909000	0.18148200	1.58544800
C	1.92659600	-1.64956500	0.07587300
C	2.25994200	0.98404200	0.89013700
C	2.83346300	-0.82209300	-0.56257900
C	3.00053500	0.51295900	-0.19674200
N	3.69842300	-1.43156400	-1.59027400
O	3.24135400	-2.35315100	-2.21508300
O	4.81154100	-0.98154900	-1.70725100
N	3.94752600	1.38896800	-0.90532300
O	3.75678100	1.55095500	-2.08122500
O	4.83182100	1.86989400	-0.24696100

N	2.31221900	2.40649700	1.27117900
O	2.15102000	2.66908600	2.43386900
O	2.48235800	3.20299800	0.38116300
N	0.29525000	-2.01352400	1.88599800
O	-0.17449300	-1.57858600	2.90855000
O	0.16474900	-3.14170400	1.47618400
C	-1.08319100	-0.70012300	-0.10779500
Br	-2.02037700	-2.23741300	-0.75256900
S	-2.03745800	0.37261700	0.82912300
H	-0.60635700	-0.16141400	-0.93374400
O	-2.73428800	-0.36202000	1.89067800
O	-1.13170800	1.48254900	1.19639100
C	-3.33562400	1.12128600	-0.16426000
C	-4.58379700	0.50325000	-0.23399500
C	-3.05182400	2.27016700	-0.90280400
C	-5.56276200	1.04580800	-1.06645800
H	-4.77627500	-0.38430800	0.37014300
C	-4.03790700	2.80670000	-1.72871600
H	-2.06915600	2.73594900	-0.81252800
C	-5.29017800	2.19339300	-1.81279600
H	-6.54423700	0.57333200	-1.12881100
H	-3.83087200	3.70910300	-2.30597900
H	-6.05946800	2.61558700	-2.46122800
H	1.80407600	-2.68600700	-0.23251000
H	0.79014100	0.58278000	2.40612200

TS-Ph-5-12345-R3-Nu4

C	-0.91504700	-1.21818600	-0.29751000
C	-1.23658200	-1.73517500	2.26003700
C	-0.87787900	0.20566900	-0.23395900
C	-2.09064600	-0.65609100	2.36949500
H	-1.61356500	-2.75913900	2.26337500
H	-0.15605000	-1.58976300	2.31637900
C	0.27552700	-1.95879800	-0.27809900
C	-2.13336700	-1.94008500	-0.80023700
C	-2.15717300	0.92987900	-0.22102400
C	0.40588300	0.82506600	-0.13819500
C	-3.58350500	-0.90129700	2.44148000
C	-1.62524800	0.65707400	2.25343600
C	1.50905700	-1.35178300	-0.11789500
O	-2.43281700	-1.53408100	-2.04203500
O	-2.72049600	-2.84262400	-0.25988900
O	-2.02254600	2.28258500	-0.23023600
O	-3.25624900	0.41010800	-0.33501700
C	1.55914400	0.07469000	-0.01910100
C	0.54947300	2.32060400	-0.05842300
H	-3.84653000	-1.82143800	1.90089500
H	-3.91675900	-1.00598600	3.48702300
H	-4.13683800	-0.07027700	1.98489000
H	-0.55950800	0.88011000	2.33203100
H	-2.31341500	1.49815200	2.37140600
C	2.75576200	-2.12941800	-0.22892100

C	-3.66118000	-2.00982300	-2.56252400
C	-3.22080000	3.01277600	-0.08413000
C	2.82790800	0.76589200	0.37325600
O	0.45246100	2.89557100	-1.25305100
O	0.79343900	2.92522400	0.95517700
O	2.56824100	-3.44792200	-0.05028900
O	3.84478200	-1.66628400	-0.48412100
H	-3.76438100	-1.57708100	-3.56272900
H	-3.66813100	-3.10652000	-2.62121600
H	-4.48949600	-1.67886600	-1.91991200
H	-2.95023700	4.07317300	-0.13197600
H	-3.69458800	2.79422400	0.88573200
H	-3.93411100	2.76768600	-0.88215100
O	3.15431700	1.75031300	-0.47211900
O	3.45061600	0.52733200	1.37402000
C	0.53543700	4.31087100	-1.25765100
C	3.71540300	-4.26449100	-0.21848900
C	4.21211900	2.59825900	-0.04938700
H	-0.25799100	4.74009400	-0.63024000
H	1.50954900	4.64229700	-0.87198400
H	0.41154600	4.62681200	-2.29779100
H	4.49316400	-3.99317300	0.50772300
H	4.12739900	-4.15325500	-1.23068100
H	3.38752600	-5.29544000	-0.05410400
H	5.13006100	2.01923400	0.11364100
H	3.93577000	3.10342500	0.88686100
H	4.35934500	3.33108200	-0.84858100
H	0.22753500	-3.04093500	-0.41103900

TS-Ph-5-12345-R4-Nu4

C	0.45849600	-0.67252200	-1.27494400
C	2.42539400	-0.88587100	0.31352800
C	0.34574700	0.70835900	-1.05531900
C	1.87670300	-0.25510500	1.44239500
H	2.50163000	-1.97588000	0.27114400
H	3.02785900	-0.32029000	-0.40195400
C	-0.61116600	-1.50958700	-0.91516900
C	-0.69742100	1.20879900	-0.30038800
C	1.17486600	-1.16689300	2.43602200
C	1.81379700	1.10574600	1.66658300
C	-1.66191700	-0.98090000	-0.18400100
C	-1.74026900	0.37413200	0.11582300
H	1.85953900	-1.93968000	2.82129300
H	0.76879400	-0.60832900	3.29164900
H	0.34078500	-1.69662600	1.94626200
H	2.27162900	1.81408300	0.97077100
H	1.29949800	1.51218100	2.54048900
F	-2.65666900	-1.77419100	0.22617200
F	-2.74790800	0.87041000	0.84341600
F	-0.75357500	2.50589900	-0.01861800
F	1.31540000	1.52020500	-1.46989700
F	1.31040000	-1.09672700	-2.22087200

H	-0.58394200	-2.57277800	-1.14864600

TS-Ph-5-21345-R3-Nu4

C	1.18862800	-0.00887200	-0.11773600
C	1.45208000	-0.36486700	2.38714300
C	-0.10002900	-0.61613700	-0.18391700
C	1.04817000	-1.67852100	2.33944900
H	2.49808300	-0.10020700	2.55304200
H	0.71296700	0.43917800	2.42370000
C	1.28071300	1.41109500	-0.09456100
C	2.40257500	-0.80025700	-0.58009400
C	-0.14591300	-2.10244300	-0.20960600
C	-1.24779300	0.19683800	-0.07096300
C	2.06511800	-2.79310100	2.39609400
C	-0.27907400	-2.03932000	2.01411000
C	2.60417700	2.08747500	-0.21263400
C	0.14648500	2.18085500	0.05164200
O	2.38305500	-0.86463100	-1.92016700
O	3.29308500	-1.25132000	0.08236000
O	-1.41583200	-2.59088300	-0.44593000
O	0.83404800	-2.79434700	-0.50527100
C	-1.12901800	1.58584400	0.06564900
C	-2.62356000	-0.42788400	-0.03771300
H	2.07057800	-3.31360700	1.42668700
H	3.07553000	-2.41143100	2.59083100
H	1.80061200	-3.52232800	3.17877300
H	-1.09269500	-1.32865500	2.16868800
H	-0.56165500	-3.09402300	2.08891100
O	2.56214000	3.37260600	0.15902500
O	3.61633700	1.57013000	-0.61691900
C	3.43155900	-1.61743200	-2.49995600
C	-1.50725100	-3.99120800	-0.51809500
C	-2.26990000	2.53679400	0.18287800
O	-3.12241100	-0.57866200	-1.26039500
O	-3.20851100	-0.71895900	0.97093800
C	3.77376700	4.09947000	0.02034200
H	3.27715800	-1.58753600	-3.58353600
H	4.40894600	-1.18576200	-2.24279000
H	3.39373900	-2.65446700	-2.13909400
H	-2.56185200	-4.23298600	-0.69635600
H	-1.17977700	-4.45873200	0.42488600
H	-0.88763900	-4.39142100	-1.33259100
O	-3.46120700	1.99557000	-0.08018600
O	-2.14420400	3.70327200	0.47173300
C	-4.39317800	-1.20155700	-1.32253000
H	3.56493300	5.11464100	0.37030400
H	4.09629000	4.11648300	-1.02940300
H	4.56808400	3.64272600	0.62514600
C	-4.58534300	2.85275300	0.05476300
H	-4.34300800	-2.20821700	-0.88463600
H	-5.13892300	-0.61258000	-0.77093300
H	-4.66152900	-1.26001000	-2.38171200

H	-4.50529200	3.70182400	-0.63650300
H	-4.65465800	3.23688000	1.08091800
H	-5.46406500	2.24698600	-0.18515900
H	0.21889100	3.26391000	0.12373600

TS-Ph-5-21345-R4-Nu4

C	0.54437800	0.73769600	0.96741200
C	2.38603400	-0.90558800	0.09680000
C	-0.44365300	-0.21894400	1.20558500
C	1.58256500	-1.43812800	-0.91944900
H	3.16238700	-0.17071500	-0.13120100
H	2.38642500	-1.33853600	1.10047800
C	0.30894500	1.65818500	-0.06329700
C	-1.57167200	-0.30985000	0.39833400
C	1.76205600	-0.81064400	-2.29392800
C	0.60551100	-2.40743200	-0.77973500
C	-0.80738800	1.58984700	-0.87469500
C	-1.77027900	0.61724100	-0.61572000
H	2.75516300	-1.04614500	-2.71093800
H	0.99961400	-1.15390600	-3.00828400
H	1.70027500	0.28790500	-2.22551600
H	0.42112200	-2.89147700	0.18392600
H	-0.02142600	-2.70452800	-1.62361200
F	1.25632300	2.57190400	-0.30253300
F	-2.86470300	0.51865000	-1.38082700
F	-2.46244000	-1.27134000	0.62405000
F	-0.27136100	-1.09617900	2.18986700
F	1.44830400	1.01561900	1.91189800
H	-0.93765300	2.29520800	-1.69479500

TS-Ph-5-31245-R3-Nu4

C	0.14691100	-0.68876800	-0.02177900
C	0.10623300	-1.06651200	2.49112500
C	-1.09869300	0.01324000	-0.12758500
C	-1.23068100	-1.40983300	2.46039700
H	0.88708100	-1.81399900	2.64025800
H	0.40089400	-0.01476800	2.52492200
C	1.35592500	0.04166700	-0.02834300
C	0.24137600	-2.14283100	-0.44455400
C	-2.31865800	-0.75735200	-0.36957200
C	-1.05915800	1.43608400	-0.06503700
C	-1.65082200	-2.86246400	2.53376200
C	-2.22199700	-0.46901600	2.15356100
C	2.63433400	-0.71357100	-0.23992500
C	1.36115800	1.42437600	0.11899300
O	0.46113200	-2.19130700	-1.77346200
O	0.22596500	-3.12868500	0.23430800
O	-3.43372700	0.01081000	-0.50531900
O	-2.35089800	-1.95529600	-0.61208900
C	0.12352300	2.11472700	0.11023100
C	-2.30226900	2.27571900	-0.11812600

H	-2.07216700	-3.17454600	1.56672300
H	-0.79876700	-3.51721900	2.75627300
H	-2.42087400	-3.00610900	3.30859200
H	-2.03134500	0.60179400	2.23443800
H	-3.27287700	-0.77345500	2.14754300
O	3.33794600	-0.20815600	-1.25706000
O	2.97684900	-1.68919700	0.37567600
C	2.58768600	2.22716400	0.31788600
C	0.57370000	-3.49402600	-2.32248800
C	-4.64512000	-0.69949900	-0.63854400
O	-2.67193400	2.55081600	-1.36925000
O	-2.86290600	2.72386500	0.84873600
C	4.61166600	-0.79580700	-1.47289500
O	3.59813600	1.52212400	0.85009500
O	2.68084100	3.41035200	0.08727900
H	0.75146700	-3.36627800	-3.39499000
H	1.40771200	-4.03856500	-1.85851100
H	-0.35397400	-4.05828100	-2.15436000
H	-5.43762700	0.04830400	-0.75144300
H	-4.83676100	-1.31254000	0.25565500
H	-4.62201600	-1.36213600	-1.51405300
C	-3.88593400	3.27311200	-1.50472100
H	5.05602100	-0.26898500	-2.32253400
H	4.51282600	-1.86634000	-1.69587900
H	5.23959300	-0.67766700	-0.57848800
C	4.82531800	2.21487500	1.01295000
H	-4.70980000	2.71667800	-1.03650400
H	-3.80981900	4.25920500	-1.02733500
H	-4.06345000	3.38385600	-2.57868800
H	4.69229400	3.08695600	1.66637200
H	5.20706100	2.55759000	0.04108400
H	5.52309700	1.50412900	1.46605900
H	0.13323400	3.20205300	0.20154100

TS-Ph-6-123456-R3-Nu4

C	-1.15994300	-0.80788600	-0.01095200
C	-1.45082400	-0.69022500	2.46042600
C	0.20104500	-1.29365600	-0.08611500
C	-0.10293700	-0.94467400	2.69049900
H	-2.15453600	-1.51029600	2.33623100
H	-1.86694000	0.30402200	2.64229800
C	-2.28135000	-1.78439300	-0.19336100
C	-1.41319900	0.57857500	-0.21653900
C	1.24225300	-0.31980500	-0.12586700
C	0.45586800	-2.67170500	-0.55487600
C	0.46140300	-2.14259700	2.27838400
C	0.81425300	0.17596800	3.13980700
O	-3.07310400	-1.43365200	-1.22541600
O	-2.50843300	-2.76363300	0.47106000
C	-2.82095500	1.06476100	-0.41012800
C	-0.39030500	1.49668200	-0.20874700
C	0.96593800	1.03351500	-0.10292300

C	2.68321500	-0.76269800	-0.09360500
O	1.68562000	-3.13463800	-0.25724600
O	-0.32879500	-3.34445900	-1.18717700
H	-0.18273200	-2.98698500	2.01916400
H	1.53417100	-2.32706700	2.36282000
H	0.41171200	1.16114200	2.86027000
H	1.80963400	0.07059100	2.68380700
H	0.94231700	0.16407000	4.23514200
C	-4.22689800	-2.23406100	-1.41716600
O	-3.63798800	0.63930500	0.55655400
O	-3.18545500	1.75777700	-1.32320000
C	-0.67911100	2.95267400	-0.28138100
C	2.03245300	2.02947400	0.17769400
O	3.14099800	-1.04206800	-1.31147900
O	3.35382900	-0.83101800	0.90324500
C	2.03665000	-4.37907300	-0.83386500
H	-3.94234500	-3.27208000	-1.63454800
H	-4.85687600	-2.22421100	-0.51631500
H	-4.76699400	-1.80194700	-2.26565100
C	-5.00548300	0.98674300	0.40031400
O	0.12528500	3.58170700	-1.14872500
O	-1.52953100	3.52761300	0.35286300
O	3.18188200	1.77017000	-0.45564500
O	1.90136300	2.97075500	0.92379600
C	4.48384900	-1.49470700	-1.36904200
H	1.34293900	-5.16665300	-0.51149000
H	2.01595300	-4.32068500	-1.93144400
H	3.04990800	-4.60616200	-0.48679100
H	-5.12835500	2.07779400	0.40341700
H	-5.39322000	0.59007900	-0.54847600
H	-5.53615600	0.54071100	1.24670700
C	0.07515000	4.99873700	-1.11955200
C	4.29396400	2.57158900	-0.08497000
H	5.16500900	-0.72543400	-0.98004600
H	4.60489800	-2.40783900	-0.76993200
H	4.69956300	-1.69718600	-2.42237300
H	-0.93918400	5.35728000	-1.33645400
H	0.37992900	5.36379200	-0.12850300
H	0.77482900	5.35041100	-1.88398300
H	4.08576700	3.63166400	-0.27836100
H	4.51894100	2.44112700	0.98229300
H	5.13619600	2.23156200	-0.69502100

TS-Ph-6-123456-R4-Nu4

C	0.75730700	-0.27180100	-1.19164500
C	2.51044000	-0.31379200	0.86963900
C	0.33139600	1.04239800	-1.01615400
C	1.51357200	0.08910900	1.76485900
H	2.77656400	-1.36842200	0.76353000
H	3.14116600	0.41949500	0.35906700
C	-0.11811600	-1.30945100	-0.85336400
C	-0.88477800	1.31648600	-0.40432200

C	0.77093400	-1.03285300	2.47976400
C	1.10378300	1.38676600	2.02794200
C	-1.32923000	-1.03442600	-0.24244000
C	-1.75112100	0.28330900	-0.06859600
H	1.37156900	-1.44410600	3.30821100
H	-0.18781400	-0.68743500	2.89550500
H	0.56120200	-1.86908300	1.79296000
H	1.60155600	2.24341500	1.56335200
H	0.26136900	1.58938900	2.69333600
F	0.29482700	-2.57180300	-0.96159400
F	-2.10102700	-2.03388000	0.17362600
F	-2.91237600	0.54308600	0.53289300
F	-1.24278900	2.57471200	-0.18603900
F	1.14227800	2.04090000	-1.34770300
F	1.82456200	-0.53276100	-1.94118900

TS-Py-3-246-R1-Nu4

C	0.64885900	1.08206100	-0.41528100
C	0.08699400	1.82230900	2.03853000
C	-0.73447400	0.98370200	-0.60792900
C	-0.64246000	0.68316700	2.39320100
H	1.14728400	1.90151700	2.28696600
H	-0.42537400	2.74447100	1.75342000
C	-1.31783700	-0.26076600	-0.48229600
C	0.13381600	-0.48378500	2.98008400
C	-1.99224700	0.51206900	2.14032400
C	0.88300800	-1.17194900	-0.32903100
C	-0.47376400	-1.40445900	-0.37738000
H	0.35415600	-0.31640500	4.04714700
H	-0.43042200	-1.42418600	2.89759100
H	1.09542600	-0.61774600	2.46294200
H	-2.60556900	1.33909200	1.77364800
H	-2.50854700	-0.40950900	2.41586800
C	-2.77598300	-0.46222600	-0.74950500
C	1.85053400	-2.32411600	-0.21370800
C	1.34274400	2.40067100	-0.64850400
F	1.85274200	2.44498800	-1.89353900
F	0.51458200	3.44540500	-0.54806400
F	2.36593000	2.61064000	0.17720100
F	1.23365700	-3.51027300	-0.22394000
F	2.73495500	-2.33138400	-1.21473200
F	2.55534300	-2.26084300	0.92198800
F	-3.32307100	-1.38873500	0.04400100
F	-3.49261900	0.65686500	-0.63010400
F	-2.97412500	-0.90228600	-2.00885000
N	1.47626300	0.02466000	-0.36881000
H	-1.34015700	1.88208300	-0.72593700
H	-0.88235000	-2.41139000	-0.31665400

TS-Py-3-246-R4-Nu4

C	-0.58804500	-0.91532100	-1.05208900

C	1.86015900	-1.43191200	-0.18169700
C	-0.26273200	0.36424100	-1.50982900
C	2.06107700	-0.26124900	0.55820200
H	1.55593000	-2.35887600	0.31010700
H	2.19907500	-1.50533500	-1.21936100
C	-0.55557700	1.39967800	-0.64736400
C	1.68695900	-0.32781000	2.03340200
C	2.52354900	0.95318400	0.07730900
C	-1.59483000	-0.13673700	0.78919100
C	-1.25055200	1.18630100	0.54749400
H	2.47339900	-0.83106900	2.62037900
H	1.54087900	0.67541100	2.46105300
H	0.75795900	-0.90058600	2.18332100
H	2.85825600	1.06211400	-0.95862000
H	2.57447800	1.83469800	0.72069800
F	-2.28374000	-0.40819500	1.90093100
F	-0.23676300	2.64603500	-0.98364300
F	-0.45112000	-1.94109500	-1.89328200
N	-1.32708400	-1.16318900	0.02518100
H	0.28493600	0.52264100	-2.43595200
H	-1.49076000	1.99037000	1.23834300

TS-Py-3-345-R1-Nu4

C	-1.19949700	0.41008000	-0.52196300
C	-1.41664600	2.09309200	0.93117500
C	0.02514700	-0.27190200	-0.11615000
C	-0.20197500	1.88737300	1.61520200
H	-1.54275500	3.00520700	0.33852000
H	-2.33733700	1.69871100	1.35982600
C	-1.13660200	1.12684200	-1.76686200
C	1.20848200	0.26419800	-0.72985300
C	0.95134300	2.84787700	1.41601800
C	0.03472100	0.66609200	2.20350500
C	1.12315000	1.23197600	-1.70389600
H	0.81005600	3.46138200	0.51419500
H	1.04504000	3.53549700	2.27305300
H	1.90582400	2.30973500	1.31955000
H	-0.79518400	-0.01257300	2.41456700
H	1.00127200	0.42445800	2.65049800
C	2.59706900	-0.15574300	-0.32274900
C	0.06319800	-1.69033400	0.36302600
C	-2.55951400	-0.19129700	-0.27063200
F	-2.72288200	-1.36686300	-0.91038800
F	-2.86507200	-0.42378500	1.01169000
F	-3.53443400	0.61150800	-0.73134600
F	2.83289200	-0.01131000	0.98800200
F	2.86431100	-1.43565100	-0.62683600
F	3.54269700	0.56848800	-0.94103100
F	0.18362000	-2.57338600	-0.65693600
F	1.10013100	-1.94816600	1.17483500
F	-1.02841500	-2.07184500	1.03531600
N	-0.04625700	1.58107900	-2.31038300

H	-2.07924600	1.35911300	-2.27483000
H	2.02723500	1.70048500	-2.09339000

TS-Py-3-345-R4-Nu4

C	0.45431500	1.30278800	-0.14140400
C	-2.06687700	0.88849400	-0.40866800
C	0.81513100	0.11139700	-0.77866100
C	-2.06974600	-0.44586900	0.02399100
H	-2.39549100	1.68939900	0.25892400
H	-1.98454900	1.12982900	-1.47174700
C	0.80523000	1.44366200	1.21620500
C	1.31009400	-0.92147400	-0.00419900
C	-2.26083700	-0.65619100	1.51729000
C	-1.77346100	-1.55717900	-0.74146500
C	1.56228500	-0.71774300	1.35317500
H	-3.14607500	-0.11650900	1.88954500
H	-2.37242100	-1.72025600	1.77164000
H	-1.38914400	-0.26412600	2.06754200
H	-1.57977600	-1.46979100	-1.81395700
H	-1.73059300	-2.55384800	-0.29607900
F	1.59816600	-2.08886700	-0.57777900
F	0.62062500	-0.03102400	-2.08272700
F	0.23983800	2.40208900	-0.87677800
N	1.31815700	0.45836400	1.93757000
H	1.97207600	-1.53680100	1.94908300
H	0.61442000	2.40646100	1.70002400

TS-Py-3-426-R4-Nu4

C	-0.14822800	-0.79296300	0.79549400
C	1.58935800	-0.81221200	-1.35512200
C	-1.25655100	-1.41131900	0.20061900
C	2.45046100	0.10509100	-0.75264700
H	0.74275100	-0.47742200	-1.96198400
H	1.81348600	-1.88150300	-1.34719500
C	-0.11206400	0.59068600	0.96405900
C	-2.21431400	-0.56085300	-0.31074400
C	3.57256700	-0.48711000	0.08788800
C	2.32694400	1.48942200	-0.73586500
C	-1.15159700	1.28401600	0.36705000
N	-2.18468800	0.75868800	-0.25355500
H	4.40074500	0.22451600	0.22351400
H	3.97798100	-1.40214100	-0.37111000
H	3.19542800	-0.76400300	1.08598000
H	1.52570800	1.99642200	-1.28250000
H	3.05230600	2.11304300	-0.20762500
F	0.72951300	-1.54900800	1.45047100
F	-1.13570700	2.61450900	0.42398000
F	-3.27896300	-1.08353700	-0.92173500
H	0.74331200	1.10618600	1.39429400
H	-1.33194800	-2.49213300	0.10267200

TS-Py-3-435-R1-Nu4

C	0.25085700	0.10655500	-0.11721500
C	0.02867400	-1.29578300	1.90349800
C	1.48299200	0.77964400	0.18774600
C	-1.31988500	-1.01062400	2.00517100
H	0.77022200	-0.58584800	2.26892700
H	0.37559800	-2.32211100	1.75228100
C	-0.95496700	0.91855100	0.05212000
C	1.49169000	2.08173800	0.62687000
C	-2.33481800	-2.11041700	1.77313200
C	-1.71769100	0.33513600	2.05878800
C	-0.73596700	2.32321400	0.26329800
N	0.38851500	2.87946000	0.61121600
H	-3.35758000	-1.71236200	1.74146900
H	-2.28562500	-2.85770600	2.58038000
H	-2.14689200	-2.63128800	0.82261400
H	-1.03706700	1.05957400	2.51271500
H	-2.77991400	0.59510600	2.10782400
C	-2.22692100	0.63795700	-0.71944900
C	0.27776000	-1.06844000	-1.04748400
C	2.82226200	0.09010800	0.12294000
F	-2.89183800	-0.47142400	-0.39291300
F	-3.11980500	1.63407600	-0.57264500
F	-1.99121700	0.55016300	-2.04499600
F	-0.87874600	-1.74024000	-1.08271300
F	0.52531700	-0.70656000	-2.32862900
F	1.22129300	-1.97389200	-0.75265200
F	3.13410200	-0.31468400	-1.11768600
F	2.90417600	-0.98362300	0.91784300
F	3.82316100	0.89901700	0.50137400
H	-1.60326900	2.98614500	0.19263500
H	2.42300400	2.55093500	0.94328600

TS-Py-3-435-R3-Nu4

C	-0.30946400	-0.14076100	-0.17696200
C	0.11805700	-0.33364900	2.35807300
C	0.84545200	-0.87401500	-0.54153100
C	1.45123000	-0.02015000	2.23060800
H	-0.59608200	0.38422500	2.76517500
H	-0.22942900	-1.35576600	2.18865000
C	-1.54888100	-0.83769100	-0.23658100
C	-0.29143500	1.37663600	-0.24732900
C	2.17127600	-0.22309300	-0.55412200
C	0.69981200	-2.25006600	-0.78369100
C	1.93862300	1.37441400	2.55058600
C	2.37520900	-0.88417200	1.60259600
C	-2.82765300	-0.11549400	-0.01633100
C	-1.55379800	-2.20257100	-0.48131100
O	-0.39238700	1.74034300	-1.53552900
O	-0.23665500	2.16967300	0.64847000
O	3.14676200	-1.05662000	-1.06718600
O	2.32572600	1.00032500	-0.58090700

H	2.29306800	1.84694600	1.62224000
H	1.13791100	1.99836100	2.96717600
H	2.77793000	1.34061000	3.26398400
H	2.15586700	-1.95187600	1.53447700
H	3.43656100	-0.62061300	1.62321400
O	-3.88231300	-0.93502200	0.08231400
O	-2.93897100	1.08458800	0.05649700
C	-0.35171200	3.13556000	-1.77366900
C	4.42419600	-0.47985700	-1.16336000
C	-5.14310500	-0.30698100	0.25845000
H	-0.45195100	3.27122000	-2.85545500
H	-1.17279200	3.64268200	-1.24834400
H	0.60321600	3.55326200	-1.42576300
H	5.08408800	-1.23807700	-1.60159300
H	4.80845900	-0.19341700	-0.17011000
H	4.41409700	0.41858900	-1.79643500
H	-5.88131700	-1.11173600	0.32350500
H	-5.36955000	0.35038700	-0.59168300
H	-5.15044400	0.29267600	1.17815100
N	-0.44576600	-2.91326500	-0.75065200
H	-2.50188700	-2.74445100	-0.47931800
H	1.59848000	-2.82512600	-1.02512800

TS-Py-3-435-R5-Nu3

C	1.15877600	0.29156800	0.57345300
C	0.76989000	1.62771000	0.41756700
C	2.35877800	-0.08240300	-0.04782700
C	1.49886900	2.48139700	-0.39095700
C	3.02759000	0.85404300	-0.85034900
N	2.62414100	2.10035300	-1.01220700
N	0.58118000	-0.55662100	1.65686200
O	0.95129300	-0.24632000	2.76108700
O	-0.14988600	-1.45492000	1.37213900
N	-0.50883100	2.10298500	0.96325600
O	-0.88100000	3.20294100	0.62848300
O	-1.10955300	1.36437800	1.70160000
N	2.98267800	-1.37947100	0.15306600
O	2.56137600	-2.07148200	1.05387800
O	3.93189300	-1.65818900	-0.54372900
H	3.94583700	0.53686100	-1.34876100
H	1.14533800	3.50309700	-0.53746600
C	-0.76426700	-0.25136400	-1.50448400
C	-2.05408600	-0.50413500	-0.89585700
C	0.17729100	-1.27244400	-1.65488400
C	-0.55352500	0.99289000	-2.32535200
C	-3.02501500	0.52174600	-0.80809000
C	-2.41881300	-1.77338000	-0.38193900
N	1.05279200	-2.04363400	-1.76643100
H	0.44575500	1.00228600	-2.78279600
H	-1.29965100	1.08793300	-3.13478200
H	-0.64151400	1.91941400	-1.72711100
C	-4.27122200	0.29471100	-0.22732000

H	-2.79469000	1.51942000	-1.18671000
C	-3.66235700	-1.99168400	0.19343300
H	-1.69527400	-2.58878400	-0.43975500
C	-4.60603300	-0.96049000	0.28035700
H	-4.99036400	1.11509400	-0.17139900
H	-3.90480700	-2.98450000	0.57915500
H	-5.58259100	-1.13635600	0.73363500

TS-Py-3-435-R5-Nu2

C	2.13348000	0.37768800	-0.03679000
C	2.56420800	-0.93245200	0.17029200
C	1.81860200	1.09971300	1.11113500
C	2.64766600	-1.45074500	1.45744200
C	1.93701400	0.50250200	2.36792900
N	2.37232100	-0.73542600	2.54496500
N	2.36518100	1.06411500	-1.34282800
O	3.53644100	1.26265100	-1.56018400
O	1.44432200	1.38527100	-2.02252900
N	2.82699000	-1.82994700	-0.95826000
O	3.19769200	-2.95049000	-0.70084600
O	2.64882000	-1.39487500	-2.06768900
N	1.43836000	2.51357200	1.06571000
O	1.79071800	3.14798900	0.10250600
O	0.84482200	2.95393800	2.01609500
H	1.68846000	1.09792300	3.24901900
H	2.95796700	-2.48951900	1.58279400
C	-0.38896400	-0.84778200	-0.87609700
Br	-0.59362700	-1.80299000	0.80073900
S	-1.31615700	0.59712900	-0.91053200
H	-0.73330600	-1.49779600	-1.68765900
O	-0.79781000	1.55481300	0.08710200
O	-1.41281300	1.02006500	-2.31637700
C	-3.02063000	0.30961400	-0.39090500
C	-3.38247500	0.48269200	0.94392500
C	-3.94501200	-0.13160600	-1.33743000
C	-4.69183400	0.20313200	1.33631300
H	-2.63852800	0.84587600	1.65408100
C	-5.25175300	-0.40707500	-0.93797100
H	-3.63456500	-0.24012100	-2.37805700
C	-5.62416800	-0.24254100	0.39824600
H	-4.98641600	0.33803500	2.37831900
H	-5.98450600	-0.74553700	-1.67229500
H	-6.64797000	-0.45818300	0.70815900

TS-Py-4-2356-R3-Nu4

C	1.15727400	-0.57138300	-0.10454700
C	1.11616200	-0.95646600	2.18751400
C	-0.14218800	-1.54184000	2.29521900
H	2.01729600	-1.56227100	2.28774900
H	1.22052500	0.12053100	2.33061100
C	1.34061000	0.88282800	-0.13898000

C	2.31838700	-1.46327700	-0.49966900
C	-1.12348300	-0.39660400	-0.16677000
C	-0.25100800	-3.03821400	2.15633900
C	-1.29702800	-0.78498800	2.12702500
C	2.65591100	1.42355700	0.10363000
C	0.18653000	1.65473900	-0.20606300
O	2.87475400	-1.02716400	-1.63473800
O	2.67136800	-2.47409900	0.04862800
C	-1.07292300	1.06923300	-0.18734800
C	-2.36882800	-1.11700100	-0.63793900
H	-0.33891300	-3.26242800	1.08012600
H	0.63825700	-3.55311500	2.54485200
H	-1.14578200	-3.43281500	2.65825400
H	-1.25974100	0.29659300	2.26578300
H	-2.27894500	-1.25833900	2.17411500
O	2.72829500	2.77136500	-0.01974300
O	3.63535600	0.76687900	0.42111000
C	4.03257200	-1.72500600	-2.06097600
C	-2.24358800	1.90196300	-0.00847300
O	-2.87455800	-0.53484200	-1.73149400
O	-2.82671700	-2.12758400	-0.17249000
C	3.98314300	3.35041900	0.27844800
H	4.36912100	-1.23759000	-2.98122100
H	4.81328600	-1.66219200	-1.29030700
H	3.80541900	-2.78241000	-2.25144400
O	-3.32364500	1.18624500	0.40013600
O	-2.30908400	3.11047000	-0.14270900
C	-4.07008900	-1.10541500	-2.23651800
H	3.86668000	4.43123900	0.14601900
H	4.76325500	2.97153100	-0.39656400
H	4.28505400	3.12900000	1.31196200
C	-4.52225300	1.91514800	0.57293500
H	-3.91013900	-2.15172400	-2.52964000
H	-4.86051200	-1.07104900	-1.47340200
H	-4.35699900	-0.50710100	-3.10675000
H	-4.82012400	2.40794100	-0.36334800
H	-4.40534000	2.68624100	1.34699200
H	-5.28629500	1.19185800	0.87708600
N	-0.02511100	-1.12210900	-0.37233000
H	0.25831900	2.74454800	-0.21711400

TS-Py-4-2356-R4-Nu4

C	-0.57340900	-1.33979100	-0.35148300
C	-1.71131200	0.62641100	-1.27524900
C	-0.76756400	-0.83979100	0.95171900
C	-1.11596500	1.71932600	-0.62041400
H	-1.40303600	0.35917300	-2.28896900
H	-2.68828600	0.25893000	-0.94744400
C	0.22072200	-0.09431900	1.53684200
C	0.11706100	2.33267700	-1.26782500
C	-1.49786900	2.21354500	0.61029900
C	1.57721200	-0.68594500	-0.35441300

C	1.44449600	-0.01213700	0.84135200
H	-0.13281700	3.26513600	-1.80096600
H	0.87886900	2.57848800	-0.51143100
H	0.57332700	1.64335700	-1.99413900
H	-2.39084200	1.83889100	1.11804600
H	-0.94869600	3.02985400	1.08587000
F	2.75617700	-0.64911000	-0.98455700
F	2.44783000	0.74197300	1.31976600
F	-1.97941000	-0.94029800	1.49721100
F	-1.50098000	-2.16476700	-0.85069200
N	0.62502900	-1.40006200	-0.91514600
H	0.06350100	0.41655800	2.48452500

TS-Py-4-3256-R3-Nu4

C	-0.77866600	-0.87270200	-0.13328800
C	-1.21368000	-1.21857100	2.29232900
C	-0.80342800	0.56230400	-0.22079700
C	-2.08649800	-0.14022000	2.32143000
H	-1.58263100	-2.24348200	2.35407500
H	-0.14458800	-1.05724800	2.44303800
C	0.48045800	-1.48953700	-0.09449500
C	-1.91892100	-1.69317800	-0.67007000
C	-2.08760500	1.24051600	-0.43589200
C	-3.57808400	-0.40064000	2.33694300
C	-1.61945800	1.15790800	2.13874000
C	1.63727300	-0.73413000	-0.00106000
O	-2.09252500	-1.42931100	-1.97361600
O	-2.55173200	-2.54355500	-0.09885800
O	-1.97771000	2.57454100	-0.61289500
O	-3.16212600	0.66991400	-0.53838200
C	1.48863500	0.69083200	-0.04836600
H	-3.81547000	-1.29434400	1.74281500
H	-3.93824300	-0.56683900	3.36543100
H	-4.13091100	0.44729500	1.91145300
H	-0.55784600	1.38656300	2.24497000
H	-2.31075700	2.00460800	2.13667900
C	2.97351200	-1.34531100	-0.05634900
C	-3.24872300	-1.99453000	-2.56554700
C	-3.20149300	3.27008800	-0.71552600
C	2.66809700	1.60080800	0.17019700
O	2.95719200	-2.66618900	0.18380500
O	3.99742500	-0.75193400	-0.31487100
H	-3.24846200	-1.67944200	-3.61386100
H	-3.23211700	-3.09047000	-2.49769800
H	-4.14654600	-1.61633500	-2.05687900
H	-2.94958000	4.32558400	-0.86494000
H	-3.79517800	3.15647500	0.20489900
H	-3.80182000	2.90248300	-1.55930200
O	2.92233400	2.36989800	-0.88783700
O	3.27620300	1.67729500	1.20613900
C	4.20577400	-3.33101000	0.07674400
C	3.98853100	3.29579800	-0.73246200

H	4.92361700	-2.92545300	0.80215200
H	4.62314200	-3.21302300	-0.93239700
H	4.01188500	-4.38739400	0.28582100
H	4.93006600	2.76450900	-0.53803900
H	3.78892700	3.97792600	0.10447900
H	4.05350400	3.85374800	-1.67132900
N	0.34070000	1.29102300	-0.19948300
H	0.55129300	-2.57814000	-0.14805200

TS-Py-4-3256-R4-Nu4

C	-1.35596200	-0.45931800	-0.78025000
C	1.61611000	-2.33117000	-0.47573300
C	-0.14173600	-0.12436800	-1.38444800
C	1.67499500	-1.45388400	0.58510500
H	1.02099200	-3.24585800	-0.42453600
H	2.22926900	-2.17683000	-1.36865900
C	-1.80599200	0.25766700	0.30691500
C	0.61069000	0.89435900	-0.78733500
C	0.83708400	-1.76672400	1.81300900
C	2.33072900	-0.21122000	0.57047300
C	-0.01184600	1.61582400	0.25463000
H	0.11112200	-2.56954100	1.61749000
H	1.47063200	-2.07998100	2.65914100
H	0.28024800	-0.87501800	2.14174100
H	3.07344600	0.00323500	-0.20185400
H	2.39589200	0.38115200	1.48817900
F	-2.97137800	-0.05560600	0.88931700
F	0.65257500	2.65649700	0.75829000
F	1.58850000	1.49626800	-1.48971900
F	-2.09019800	-1.46643100	-1.26296600
N	-1.13477300	1.29076400	0.80969100
H	0.22188900	-0.67604200	-2.24783700

TS-Py-4-3256-R5-Nu1

C	-0.70155500	0.26362600	-0.79595500
C	0.26433300	-0.69794400	-1.18053800
C	-0.19139900	1.50661700	-0.33100100
C	1.55331600	-0.49709200	-0.75739900
C	1.88868100	0.68044800	-0.06577500
N	3.19037100	0.84439200	0.58922400
O	3.63050000	1.95813400	0.70182700
O	3.70754200	-0.16903200	1.00555500
N	2.57352000	-1.46519400	-1.18352100
O	3.61638500	-1.01576000	-1.58672700
O	2.27467100	-2.63243800	-1.14146300
N	-2.02828400	0.16252900	-1.40493600
O	-2.21322800	-0.75500100	-2.17270900
O	-2.86008300	0.98556400	-1.09595400
N	-0.97568500	2.76771800	-0.36479200
O	-1.29615200	3.15196100	-1.46118500
O	-1.15860300	3.34599800	0.67292600

N	1.03750800	1.66904100	0.07066500
H	-0.02548900	-1.57608700	-1.75313700
C	-1.70366400	-0.75552600	1.21372900
C	-0.40907200	-0.63982300	1.89936200
C	-2.09183700	-1.96500500	0.51670000
C	-2.71300900	0.26374600	1.65937200
O	-0.12322700	0.41661900	2.45767100
C	0.55681200	-1.80609300	1.98376500
O	-1.26893400	-2.78118100	0.09353100
C	-3.56574000	-2.17672000	0.21856100
H	-2.29230000	1.27618900	1.57181400
H	-3.64404900	0.22288900	1.08449500
H	-2.95902200	0.12899100	2.72764200
H	1.45716600	-1.46641200	2.51099600
H	0.08040400	-2.62461800	2.54579900
H	0.80410800	-2.22754300	1.00440300
H	-4.17246600	-2.11499000	1.13322800
H	-3.93440300	-1.40411800	-0.47499400
H	-3.68811700	-3.16002400	-0.24943700

TS-Py-5-23456-R2-Nu2

C	1.31348200	1.20900300	1.36885200
C	1.90267500	1.68782100	0.18009400
C	2.88975700	0.89284700	-0.41651500
C	2.65792200	-0.62583300	1.43869000
C	3.26028100	-0.30991900	0.20296400
C	3.52320800	1.30518300	-1.64132300
N	4.03729900	1.63419800	-2.61926700
C	1.56043000	2.96800500	-0.37394900
N	1.37572000	4.02430100	-0.79803800
C	4.24966400	-1.17108500	-0.37994600
N	5.04682200	-1.86355400	-0.84423600
C	3.06801300	-1.82051900	2.15026700
N	3.40783100	-2.77103900	2.70657700
C	0.26456700	1.97520000	2.00917800
N	-0.53837300	2.59749000	2.55361800
C	-0.38327200	-0.72421700	-0.20624200
Br	-0.96642000	-2.56750900	-0.54237900
S	-1.39730700	0.39031600	-1.06242400
H	0.57374300	-0.62524600	-0.74592200
O	-0.81697400	1.72649000	-0.77674600
O	-1.67598100	0.10161200	-2.48216500
C	-2.99496600	0.32047900	-0.25161500
C	-3.23582300	1.12578900	0.85900000
C	-3.97803700	-0.52137100	-0.76825800
C	-4.48428200	1.06825800	1.47729400
H	-2.45455800	1.79415700	1.22222200
C	-5.22162800	-0.57602000	-0.13962300
H	-3.75928100	-1.11651500	-1.65534500
C	-5.47292100	0.21580300	0.98199200
H	-4.68705000	1.69527000	2.34675600
H	-5.99864500	-1.23452500	-0.53056200

H	-6.44811000	0.17306800	1.46977400
N	1.72566600	0.12005800	2.00152800

TS-Py-5-23456-R3-Nu4

C	0.98827400	-1.32273800	0.39656000
C	1.11058400	-1.00978400	2.21263500
C	0.16995100	0.02559800	2.44987000
H	0.88822700	-1.99252400	2.63985900
H	2.17547700	-0.75696800	2.25153600
C	1.27273800	-0.02988400	-0.16390400
C	2.02134900	-2.42688100	0.17947900
C	-1.25573500	-1.05810600	-0.08048300
C	-1.18621300	-0.31993900	2.99356300
C	0.42291300	1.30335700	1.88182500
C	2.67272600	0.34030900	-0.27270900
C	0.22644400	0.92959600	0.06639400
O	2.21784500	-2.63480700	-1.12291900
O	2.52797800	-3.10582600	1.03330000
C	-1.15070900	0.34768600	-0.10607100
C	-2.57912400	-1.74535300	-0.33131400
H	-1.56156300	-1.25336800	2.54587300
H	-1.12681500	-0.47878900	4.08355500
H	-1.91215800	0.48279400	2.80688900
H	1.44852700	1.68068300	1.89437800
H	-0.32519700	2.08312900	2.05231000
O	2.87814300	1.60870200	-0.67630600
O	3.61168700	-0.39112100	-0.00586300
C	0.35764900	2.26160400	-0.67215800
C	3.17372600	-3.63180800	-1.44482800
C	-2.25179000	1.24602800	-0.00395300
O	-2.95420700	-1.65989800	-1.60659900
O	-3.19170100	-2.36272300	0.50100900
C	4.22726500	2.03195800	-0.76052400
O	0.73149000	3.28848800	0.08996500
O	0.14178100	2.36815300	-1.85004500
H	3.21134000	-3.68445400	-2.53715000
H	4.15843900	-3.35448200	-1.04314100
H	2.88073200	-4.60374400	-1.02605400
O	-3.47908200	0.64868900	-0.03481200
O	-2.16819200	2.46233300	0.14198600
C	-4.21429400	-2.23340300	-1.91457800
H	4.20265800	3.08026700	-1.07475700
H	4.77849300	1.42967000	-1.49525100
H	4.72687400	1.93922300	0.21318900
C	0.85578900	4.52517500	-0.58735900
C	-4.59172900	1.50685900	0.09897600
H	-4.22649000	-3.30489700	-1.67393700
H	-5.00644200	-1.73308600	-1.33978100
H	-4.37130100	-2.08287100	-2.98690600
H	-0.10064800	4.80611200	-1.04853500
H	1.61982700	4.45746900	-1.37465700
H	1.14873700	5.26476900	0.16449400

H	-4.64088700	2.22766300	-0.72953400
H	-4.54680000	2.06957100	1.04192000
H	-5.48107500	0.86682100	0.08877200
N	-0.29782500	-1.90864900	0.16295200

TS-Py-5-23456-R4-Nu4

C	0.84661200	-1.35711200	-0.00116800
C	1.56750200	0.27991900	1.76136900
C	0.95510100	-0.37318900	-0.99604700
C	0.72317600	1.36101900	1.47983800
H	1.34732200	-0.40064100	2.58682000
H	2.58267400	0.25222500	1.35435500
C	-0.16224600	0.38141600	-1.27832100
C	-0.59414300	1.44183200	2.23712000
C	0.94202300	2.29964000	0.48641800
C	-1.38289900	-1.11298700	0.13076500
C	-1.38894400	-0.01104700	-0.70157300
H	-0.53957300	2.17495600	3.05873600
H	-1.41329700	1.75499000	1.57068700
H	-0.86679800	0.46959500	2.67477200
H	1.88291900	2.32484800	-0.06980200
H	0.20711500	3.07923800	0.27387900
F	-2.54265900	-1.50258500	0.66240000
F	-2.47777000	0.74768700	-0.87280600
F	-0.14476900	1.33563200	-2.19721900
F	2.14442800	-0.04334800	-1.49026900
F	1.91124000	-2.12962000	0.21914600
N	-0.32167000	-1.82600700	0.42126800

TS-Py-5-32456-R3-Nu4

C	-0.80467400	-0.58701200	0.04003100
C	-1.14071900	-0.69227300	2.50582500
C	-1.22319000	0.79480900	-0.17390800
C	-2.27236500	0.08197900	2.36905800
H	-1.20127000	-1.75098800	2.76255000
H	-0.15644800	-0.22014300	2.55077200
C	0.57841100	-0.82711900	0.01977000
C	-1.70621500	-1.74961300	-0.31522900
C	-2.58986400	1.05906700	-0.65504900
C	-3.65134600	-0.53521500	2.43592700
C	-2.16166600	1.40000400	1.89686500
C	1.04656800	-2.24959100	-0.07555000
C	1.49473900	0.22077500	0.05490900
O	-1.45189100	-2.12968200	-1.58403700
O	-2.49220300	-2.33296000	0.37585600
O	-2.83318700	2.35072000	-0.93726200
O	-3.42848000	0.19550200	-0.83709500
C	0.96508600	1.53320000	-0.11524000
H	-4.11488500	-0.52289900	1.43810700
H	-3.60601300	-1.57951800	2.76943800
H	-4.29834400	0.03040600	3.12520200

H	-1.22872900	1.94971900	2.02709400
H	-3.06087600	2.01014200	1.76752800
O	2.01755100	-2.39531700	-0.97997000
O	0.58287300	-3.17058400	0.54562600
C	2.95447000	-0.01860200	0.10318300
C	-2.18636800	-3.25314300	-2.04184200
C	-4.16243500	2.64526800	-1.31932500
C	1.86959800	2.72724500	0.00343000
C	2.56792000	-3.70037100	-1.09106800
O	3.27988400	-0.95989500	1.00518900
O	3.78004400	0.54026100	-0.57579100
H	-1.87121300	-3.43180100	-3.07477900
H	-1.97005400	-4.13209300	-1.41846400
H	-3.26491200	-3.04810700	-2.00201400
H	-4.19372900	3.71892000	-1.53198300
H	-4.86468000	2.40357000	-0.50764800
H	-4.45491900	2.07407000	-2.21088900
O	1.59201100	3.68195700	-0.88195300
O	2.72963300	2.83333200	0.84139100
H	3.34920300	-3.64137000	-1.85444400
H	1.79415000	-4.41979700	-1.38946400
H	2.99372400	-4.01663300	-0.12869700
C	4.63819800	-1.36846300	1.00242600
C	2.36036800	4.87098800	-0.76545700
H	5.29782600	-0.52331800	1.23916000
H	4.91599000	-1.76551900	0.01540200
H	4.72967900	-2.14730500	1.76576400
H	3.42900200	4.65329300	-0.89460100
H	2.21146600	5.33076900	0.22071000
H	2.01025500	5.54298000	-1.55444300
N	-0.30202800	1.78105100	-0.30892500

TS-Py-5-32456-R4-Nu4

C	0.93151800	-0.62117600	-0.74289000
C	2.09502100	1.03296000	0.75784300
C	-0.14139600	0.11661300	-1.25882000
C	0.91779700	1.73311900	1.06513300
H	2.57188700	0.39696100	1.50900700
H	2.71018900	1.33725500	-0.09331100
C	0.59049500	-1.59313400	0.21680900
C	-1.39659000	0.00322200	-0.65931500
C	0.24706300	1.39698200	2.38800000
C	0.26283100	2.60935900	0.22267200
C	-1.54933900	-0.90313500	0.37074600
H	0.66120700	2.00202700	3.21157300
H	-0.83698000	1.58293700	2.34843600
H	0.39988100	0.33926100	2.65243600
H	0.70857800	2.91729000	-0.72697900
H	-0.68477300	3.07037900	0.51135900
F	1.56738800	-2.39370100	0.63753400
F	-2.72456800	-0.99427000	1.00083100
F	-2.40425000	0.76144200	-1.07694700

F	0.03293000	0.88068300	-2.32997900
F	2.05222000	-0.74571100	-1.46004000
N	-0.56969500	-1.69585300	0.78340400

TS-Py-5-42356-R3-Nu4

C	0.14642700	-0.75883700	0.03499200
C	0.21668800	-1.27546100	2.47095900
C	-1.14304600	-0.09773100	0.00707700
C	-1.14328800	-1.50096200	2.46299700
H	0.93062800	-2.09215100	2.58929900
H	0.60286600	-0.25784900	2.56281600
C	1.29574600	0.05944100	-0.00269400
C	0.32750600	-2.18507700	-0.43817800
C	-2.32003000	-0.84422200	-0.44933300
C	-1.09619000	1.33759700	-0.02808700
C	-1.69950100	-2.90786700	2.46873100
C	-2.01973200	-0.44332100	2.17680300
C	2.62212300	-0.60533900	-0.21335600
C	1.18830400	1.43692300	0.13353000
O	0.79676500	-2.17129000	-1.70375200
O	0.17273300	-3.20909600	0.16480500
O	-3.43455300	-0.08998200	-0.54884500
O	-2.32535500	-2.01855500	-0.75871500
C	-2.35494600	2.17247300	-0.06693200
H	-2.12855100	-3.14951900	1.48517300
H	-0.91539200	-3.64635800	2.67858900
H	-2.49236500	-3.00940500	3.22655900
H	-1.72561100	0.58785400	2.37641700
H	-3.09840000	-0.62212500	2.12666100
O	3.30184300	-0.05017500	-1.21885200
O	3.01852000	-1.56357800	0.39953000
C	2.35765600	2.32556600	0.32815600
C	1.11506300	-3.44059300	-2.25032800
C	-4.61452400	-0.77305900	-0.92912100
O	-2.65931100	2.56556500	-1.30198100
O	-2.96676600	2.51511700	0.91037600
C	4.60088900	-0.57511800	-1.44472200
O	3.39271500	1.68374500	0.90379700
O	2.40518800	3.49890600	0.05171000
H	1.47996200	-3.25951400	-3.26630700
H	1.89088700	-3.93306900	-1.64716500
H	0.22508900	-4.08379900	-2.27294900
H	-5.40471200	-0.01769400	-0.98759100
H	-4.87821700	-1.53541000	-0.18230500
H	-4.48608600	-1.26511100	-1.90220000
C	-3.83615000	3.35083000	-1.42383700
H	5.02533600	-0.00407200	-2.27577900
H	4.54831700	-1.64170400	-1.70158900
H	5.22034000	-0.45874900	-0.54440800
C	4.59481000	2.42490100	1.01982800
H	-4.70629100	2.79003800	-1.05508400
H	-3.74474600	4.28012500	-0.84621300

H	-3.95126900	3.57478300	-2.48849200
H	4.43640200	3.32881700	1.62260200
H	4.96148700	2.72468500	0.02766300
H	5.31964400	1.76633400	1.50852900
N	-0.02148300	2.06254100	0.09175600

TS-Py-5-42356-R4-Nu4

C	0.10806000	-0.70391200	0.32713200
C	1.16737300	-1.36575200	-1.16411000
C	-1.28139100	-1.01099700	0.23117300
C	2.18800500	-0.40843900	-1.03395600
H	0.42693600	-1.21363000	-1.96103600
H	1.36879100	-2.42162500	-0.94595400
C	0.48677300	0.76256600	0.23331000
C	-2.17459100	-0.07112300	-0.20185800
C	3.38226400	-0.66477700	-0.14768200
C	1.56534500	0.91157400	-1.03793900
C	-0.69647200	1.60354900	0.09072400
N	-1.87966400	1.23687700	-0.25291500
H	4.27270700	-0.11331700	-0.49308700
H	3.64055400	-1.73451700	-0.14420800
H	3.20306500	-0.36527800	0.89967800
H	0.94305300	1.09997500	-1.92649000
H	2.20427500	1.78086000	-0.82930700
F	0.77018400	-1.36698000	1.33403400
F	1.25346400	1.15961900	1.33023400
F	-0.43459100	2.92586800	0.11454200
F	-3.42901000	-0.42084900	-0.55028600
F	-1.64603800	-2.29840700	0.37037200
